# Supplementary material for: Rational enzyme design for enabling biocatalytic Baldwin cyclization and asymmetric synthesis of chiral heterocycles
Source: Nat Commun. 2022 Dec 19;13:7813. doi: 10.1038/s41467-022-35468-y (PMC9763437; doi:10.1038/s41467-022-35468-y)
Supplement: Supplementary file 1 — Supplementary Information [file 41467_2022_35468_MOESM1_ESM.pdf]

Supplementary Information

Rational enzyme design for enabling biocatalytic Baldwin cyclization and asymmetric synthesis of chiral heterocycles

Jun-Kuan Li,[1,2#] Ge Qu,[2,3#] Xu Li,[2,3#] Yuchen Tian,[1] Chengsen Cui,[2,3] Fa-Guang Zhang,[1] Wuyuan Zhang,[2,3] Jun-An Ma,[1✉] Manfred T. Reetz,[2,4✉] and Zhoutong Sun[2,3✉]

[1] Department of Chemistry, Frontiers Science Center for Synthetic Biology (Ministry of Education), Tianjin University, Tianjin 300072, China

[2] Tianjin Institute of Industrial Biotechnology, Chinese Academy of Sciences, Tianjin 300308, China

[3] National Technology Innovation Center of Synthetic Biology, Tianjin 300308, China

[4] Biocatalysis Section, Max-Planck-Institut für Kohlenforschung, Kaiser-Wilhelm-Platz 1, 45470 Mülheim an der Ruhr, Germany

[#] These authors contributed equally.

[✉] e-mails of corresponding authors: majun_an68@tju.edu.cn; reetz@mpi-muelheim.mpg.de; sunzht@tib.cas.cn.

1. Supplementary Figures 3
2. Supplementary Tables 11
3. Supplementary Methods 18
3.1 Preparation and characterization of starting materials 18
3.1.1 General procedure for the synthesis of epoxy alcohols 19
3.1.2 General procedure for the synthesis of oxetane alcohols 21
3.1.3 General procedure for the synthesis of oxetane amines 27
3.2 General procedure for the synthesis of chiral heterocycles using *Re*LEH mutants 34
3.2.1 General procedure for the synthesis of racemic products 34
3.2.2 General procedure for Baldwin/anti-Baldwin and desymmetrization reactions 35
3.2.3 General procedure for Baldwin and anti-Baldwin reactions catalyzed by *Re*LEH mutants 35
3.2.4 General procedure for scaling-up enantioselective intramolecular openings of oxetanes 36
3.2.5 General procedure for asymmetric desymmetrization of oxetanes using *Re*LEH mutants to synthesize chiral tetrahydrofurans and tetrahydropyrans 37
3.2.6 General procedure for asymmetric desymmetrization of oxetanes using *Re*LEH mutants to synthesize chiral pyrrolidines and piperidines 42
3.3 Synthetic transformations and derivatizations 48
3.4 NMR spectra of the relevant compounds 52
3.5 HPLC chromatograms 152
4. Supplementary References 189

1. Supplementary Figures

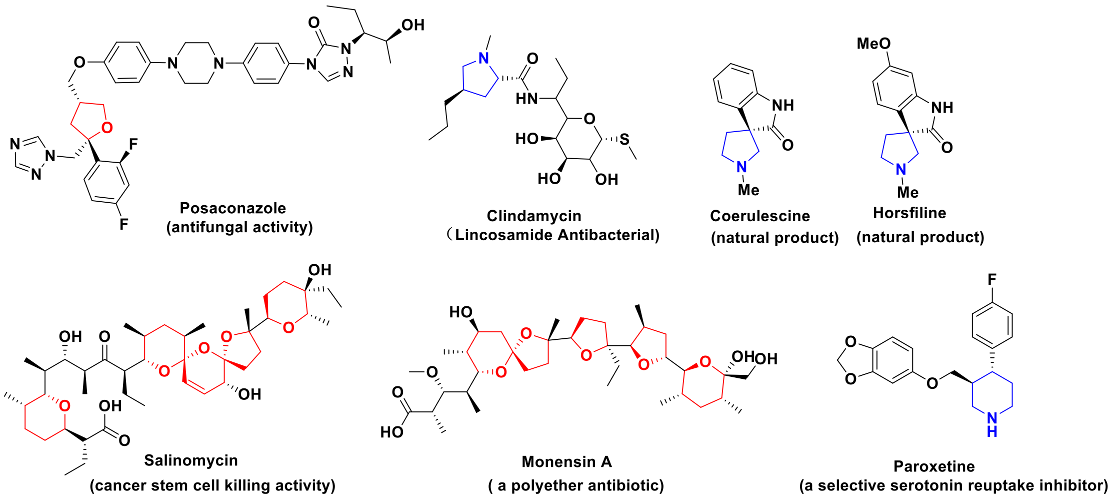

Supplementary Figure 1. Chiral *N*- and *O*-heterocycles are ubiquitous in important natural products and medicinally relevant molecules.

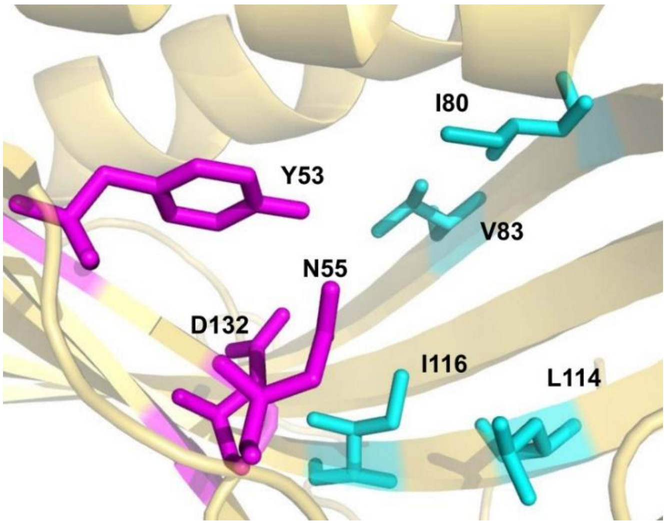

Supplementary Figure 2. The residues of *Re*LEH mutated in this study.

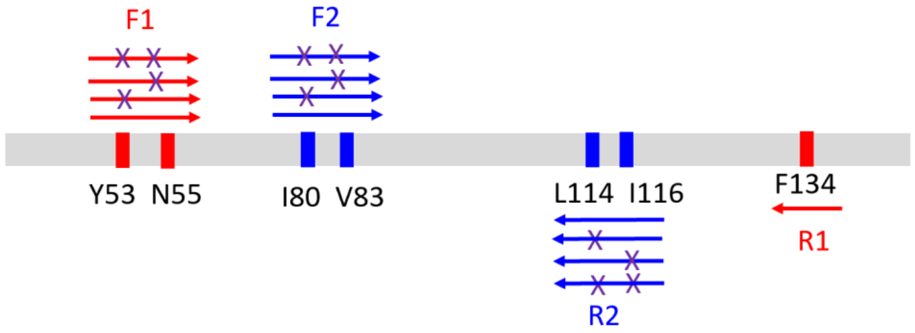

Supplementary Figure 3. Primer design for creation of *Re*LEH mutant libraries A (red), B (light blue)

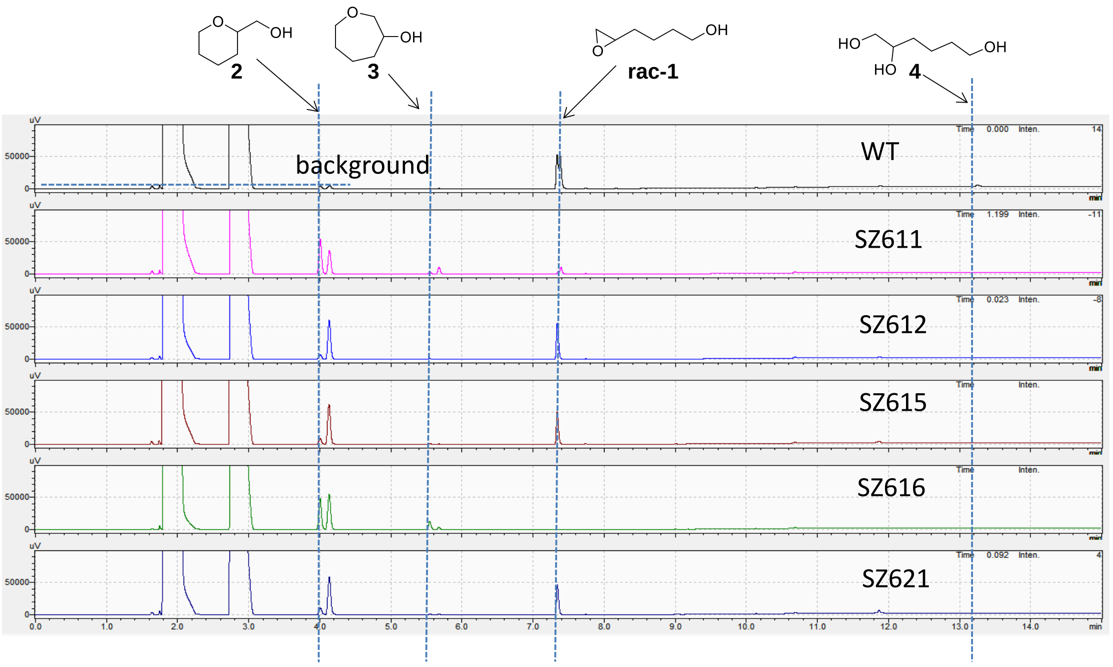

Supplementary Figure 4. GC profiles of transformation of ***rac*-1** to **2, 3** and **4** catalyzed by WT *Re*LEH and mutants.

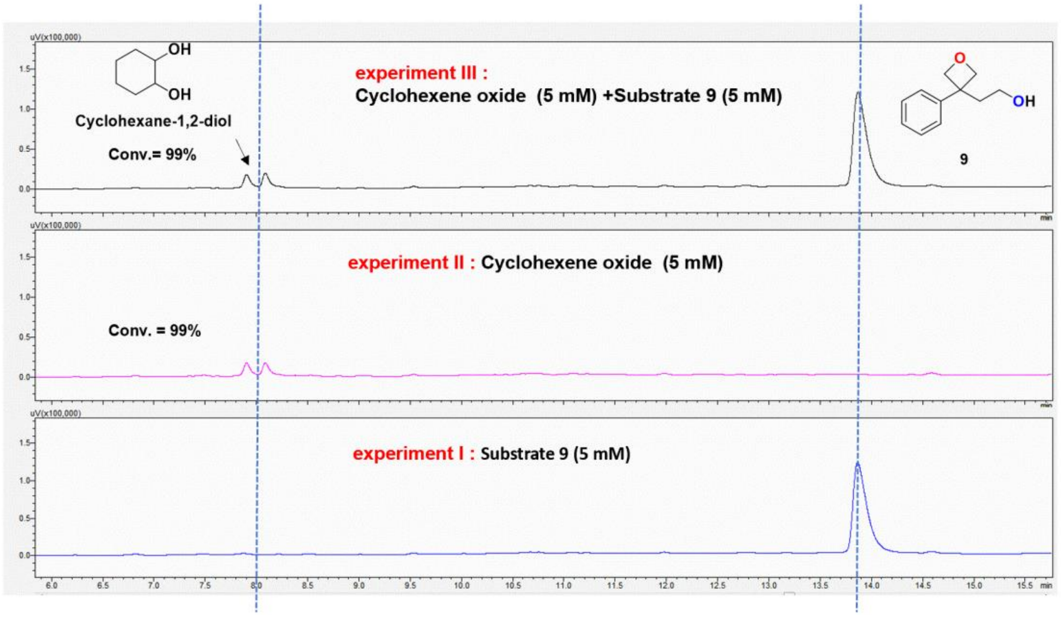

Reaction conditions: LEH (0.1 g/mL wet cell), Substrates (5 mM), PBK buffer (50 mM, pH 7.4), Lysozyme (1 mg/mL), Dnase I (6 U/mL), 0.5 mLtotal volume, 30 °C, 1,000 rpm, 24 h. GC analysis by chiral column Hydrodex-β-TBDAc, 25 m x 0.25 mm ID as the follows condition: 110 °C, 5 °C/min, 135 °C, 20 °C/min, 220 °C hold 2 min. N_2: 1.5 bar

Supplementary Figure 5. GC profiles of the transformation of cyclohexene oxide to the vicinal diol**,** and substrate **9** for WT *Re*LEH. The results show that WT *Re*LEH catalyzes the complete conversion of cyclohexene oxide to the vicinal diol with or without the addition of substrate **9**. The three experiments I, II and III share the same reaction conditions, except that in experiment I only substrate **9** was added, in II only the natural substrate cyclohexene oxide was used, while in the case of III substrate **9** and cyclohexene oxide were simultaneously added.

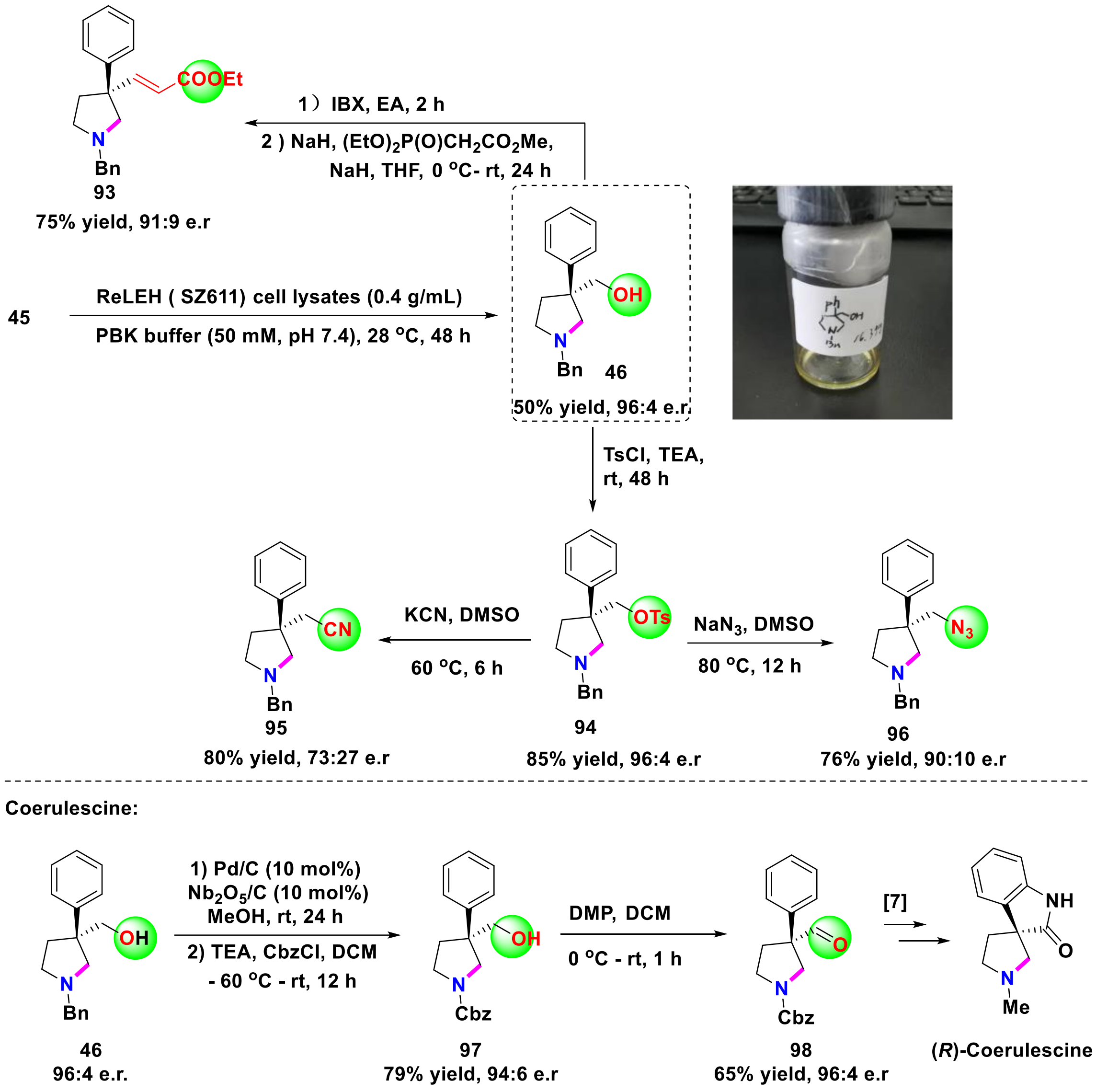

Supplementary Figure 6. Synthetic transformations and derivatizations of products obtained by enzymatic reactions of the present study.

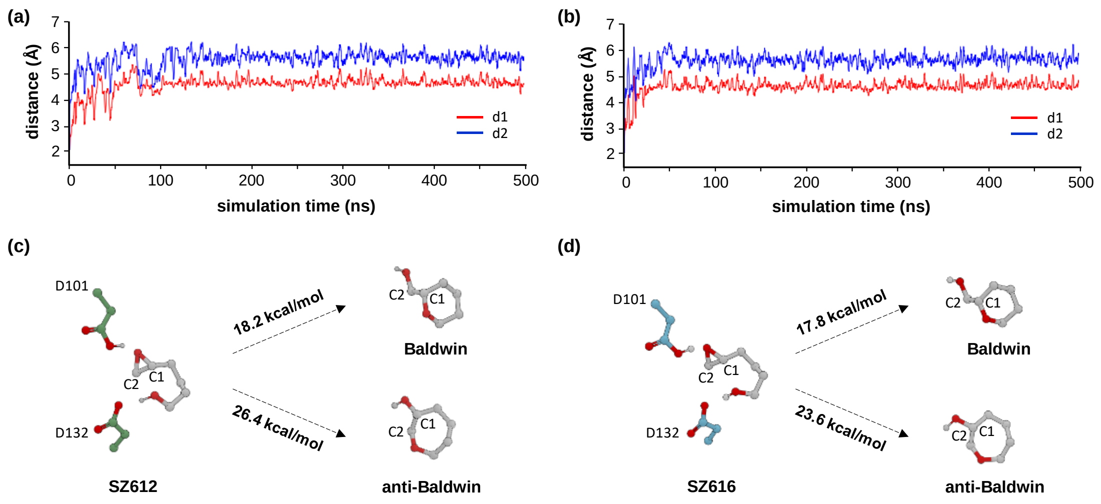

Supplementary Figure 7. Distances of d1 (O_N-C1) and d2 (O_N-C2) along MD simulations of SZ612–(*R*)-**1** (a) and SZ616–(*R*)-**1** (b). Calucated activation enery barriers of models SZ612 (c) and SZ616 (d) toward Baldwin and anti-Baldwin products using QM/MM scheme.

.

2. Supplementary Tables

Supplementary Table 1. Primer list used for the site-directed mutagenesis towards residues Y53 and N55.

Primer mix	Name of primers	Oligo nucleotides 5' to 3'
	LEH-53P-F	GCAGAAGACACGATGCCGCAGAACATGCCACTCCCCCCTGCATAC
	LEH-55P-F	GCAGAAGACACGATGTACCAGCCGATGCCACTCCCCCCTGCATAC
	LEH-5355P-F	GCAGAAGACACGATGCCGCAGCCGATGCCACTCCCCCCTGCATAC
	LEH-53GBA-F	GCAGAAGACACGATGGBACAGAACATGCCACTCCCCCCTGCATAC
	LEH-55GBA-F	GCAGAAGACACGATGTACCAGGBAATGCCACTCCCCCCTGCATAC
	LEH-5355GBA-F	GCAGAAGACACGATGGBACAGGBAATGCCACTCCCCCCTGCATAC
	LEH-53WTK-F	GCAGAAGACACGATGWTKCAGAACATGCCACTCCCCCCTGCATAC
F1	LEH-55WTK-F	GCAGAAGACACGATGTACCAGWTKATGCCACTCCCCCCTGCATAC
	LEH-5355WTK-F	GCAGAAGACACGATGWTKCAGWTKATGCCACTCCCCCCTGCATAC
	LEH-53WTK55P-F	GCAGAAGACACGATGWTKCAGCCGATGCCACTCCCCCCTGCATAC
	LEH-53P55WTK-F	GCAGAAGACACGATGCCGCAGWTKATGCCACTCCCCCCTGCATAC
	LEH53WTK55GBA-F	GCAGAAGACACGATGWTKCAGGBAATGCCACTCCCCCCTGCATAC
	LEH53GBA55WTK-F	GCAGAAGACACGATGGBACAGWTKATGCCACTCCCCCCTGCATAC
	LEH-53GBA55P-F	GCAGAAGACACGATGGBACAGCCGATGCCACTCCCCCCTGCATAC
	LEH-53P55GBA-F	GCAGAAGACACGATGCCGCAGGBAATGCCACTCCCCCCTGCATAC
R1	Leh134-wt-R	TTCGCGCAGATCGAAGTAGTCACGCCAACCCG

Supplementary Table 2. Primer list of *Re*LEH for I80/V83-L114/I116 using SZ611 as template

Primer mix	Name of primers	Oligo nucleotides (5' to 3')
	LEH-8083VFY-wt-F	GCCTGTTCACCGTCATGAGCATCGATGCGGTGGAGACGTTCCATATCGGCTC
	LEH-8083VFY-80V-F	GCCTGTTCACCGTCATGAGCGTGGATGCGGTGGAGACGTTCCATATCGGCTC
	LEH-8083VFY-80 TWT-F	GCCTGTTCACCGTCATGAGCTWTGATGCGGTGGAGACGTTCCATATCGGCTC
F2	LEH-8083VFY-83 TWT-F	GCCTGTTCACCGTCATGAGCATCGATGCGTWTGAGACGTTCCATATCGGCTC
	LEH-8083VFY-80TWT 83TWT-F	GCCTGTTCACCGTCATGAGCTWTGATGCGTWTGAGACGTTCCATATCGGCTC
	LEH-8083VFY-80V83TWT-F	GCCTGTTCACCGTCATGAGCGTGGATGCGTWTGAGACGTTCCATATCGGCTC
	LEH-114116VFY-wt-R	CGGTGAGCTGGAAGACTCCGAGGATTGACAGGTTGTAGCTCTTGCCGGTGG
	LEH-114116VFY-114V-R	CGGTGAGCTGGAAGACTCCGAGGATTGACACGTTGTAGCTCTTGCCGGTGG
	LEH-114116VFY-116V-R	CGGTGAGCTGGAAGACTCCGAGCACTGACAGGTTGTAGCTCTTGCCGGTGG
	LEH-114116VFY-114V116V-R	CGGTGAGCTGGAAGACTCCGAGCACTGACACGTTGTAGCTCTTGCCGGTGG
R2	LEH-114116VFY-114AWA-R	CGGTGAGCTGGAAGACTCCGAGGATTGAAWAGTTGTAGCTCTTGCCGGTGG
	LEH-114116VFY-116AWA-R	CGGTGAGCTGGAAGACTCCGAGAWATGACAGGTTGTAGCTCTTGCCGGTGG
	LEH-114116VFY-114AWA116AWA-R	CGGTGAGCTGGAAGACTCCGAGAWATGAAWAGTTGTAGCTCTTGCCGGTGG
	LEH-114116VFY-114V116AWA-R	CGGTGAGCTGGAAGACTCCGAGAWATGACACGTTGTAGCTCTTGCCGGTGG
	LEH-114116VFY-114AWA116V-R	CGGTGAGCTGGAAGACTCCGAGCACTGAAWAGTTGTAGCTCTTGCCGGTGG

Supplementary Table 3. Hydrolytic desymmetrization catalyzed by the wildtype (WT) *Re*LEH and variants.

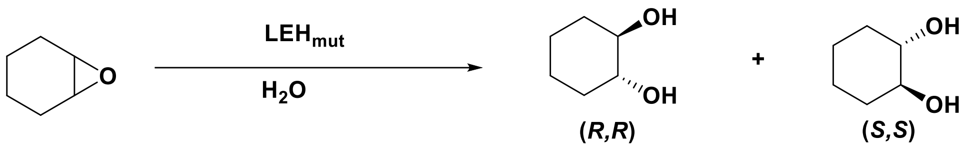

Code	Mutation	c%[a]	e.r[b]
WT	–	99	–
SZ532	Y53L/N55P	77	67:33 (*R,R*)
SZ533	Y53I/N55P	63	73:27 (*R,R*)
SZ534	Y53V/N55P	50	64:32 (*R,R*)
SZ609	Y53I/N55A	90	74:26 (*R,R*)
SZ610	Y53F/N55P	46	52:48 (*R,R*)
SZ611	Y53F/N55A	55	52:48 (*R,R*)

Reaction conditions: LEH (0.1 g/mL wet cell), Substrates (5 mM), PBK buffer (50 mM, pH 7.4), Lysozyme (1 mg/mL), Dnase I (6 U/mL), 0.5 mL total volume, 30 °C, 1,000 rpm, 24 h. [a]: c = Conversion; [b]: The er value was determined by chiral GC column; –, not detectable.

Supplementary Table 4. Screening of *Re*LEH mutants in the intramolecular desymmetrization of substrate **9**.

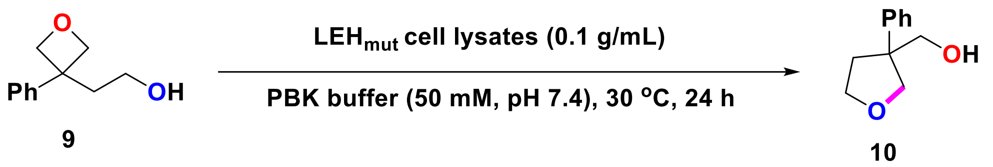

Code	Mutation	10	
		c%[a]	e.r[b]
WT	–	–	–
SZ532	Y53L/N55P	33	76:24 (*S*)
SZ533	Y53I/N55P	5	53:47 (*S*)
SZ534	Y53V/N55P	9	56:44 (*S*)
SZ609	Y53I/N55A	47	56:44 (*S*)
SZ610	Y53F/N55P	80	64:36 (*S*)
SZ611	Y53F/N55A	83	66:34 (*S*)
SZ612	Y53F/N55A/I80F/L114V/I116V	8	86:14 (*S*)
SZ616	Y53F/N55A/I116V	96	72:28 (*S*)
SZ621	Y53F/N55A/I80F/I116V	68	87:13 (*S*)

Reaction conditions: LEH (0.1 g/mL wet cell), Substrates (5 mM), PBK buffer (50 mM, pH 7.4), Lysozyme (1 mg/mL), Dnase I (6 U/mL), 0.5 mL total volume, 30 °C, 1,000 rpm, 24 h. [a]: c = Conversion; [b]: The er value was determined by chiral GC column; –, not detectable.

Supplementary Table 5. Determination of absolute configurations of the chiral heterocycles by the X-ray crystallographic structures for derivative of **54**.

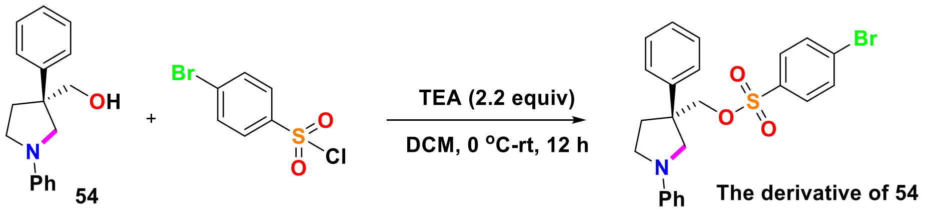

The crystallized derivative of **54** was obtained by two phases recrystallization with 1 mL of ethyl acetate and 15 mL of hexane at room temperature. ORTEP representation with 50% probability thermal ellipsoids. Solvent is omitted for clarity. Crystal data have been deposited to CCDC, number 2162958.

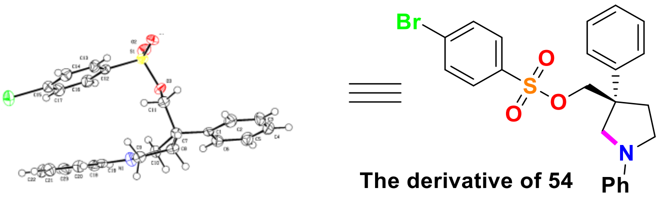

Empirical formula	$C_{23}H_{22}BrNO_3S$
Identification code	1219Y_0m
Formula weight	472.38
Temperature/K	150.0
Wavelength	0.71073 Å
Crystal system, space group	monoclinic, $P2_1$
Unit cell dimensions	a = 11.3309(10) Å = 90°.
	b = 6.2613(6) Å = 90°.
	c = 15.3055(17) Å = 90°.
Volume	1029.52(18) Å3
Z, Calculated density	2, 1.524 g/m3
Absorption coefficient	2,123 μ/mm^{-1}
F (000)	484.0
Crystal size	0.15 × 0.08 × 0.05 mm3
Theta range for data collection	3.936 to 52.848°.
Limiting indices	-14<=h<=14, -7<=k<=7, -19<=l<=19
Reflections collected	11810
Independent reflections	3798 [R_{int} = 0.0613, R_{sigma} = 0.0783]
Completeness to theta = 24.999°	100.0 %
Absorption correction	Semi-empirical from equivalents
Refinement method	Full-matrix least-squares on F2
Data / restraints / parameters	3798/1/262
Goodness-of-fit on F^2	1.009
Final R indices [I>2sigma(I)]	R_1 = 0.0393, wR_2 = 0.0579
R indices (all data)	R_1 = 0.0611, wR2 = 0.0664
Extinction coefficient	n/a
Largest diff. peak and hole	0.28/-0.38 e.Å-3

Supplementary Table 6. Data collection and refinement statistics of the solved X-ray structures in this study.

Name	SZ611	SZ612	SZ616	SZ611-substrate **59**	SZ611-product **46**
PDB ID	7VWD	7VX2	7VWM	7XEE	7XEF
**Data collection**					
Wavelength (Å)	1.0331	1.0331	0.9785	1.5418	1.5418
Space group	$P1$	$P2_12_12_1$	$P2_12_12_1$	$P12_11$	$P1$
Unit cell parameters					
a, b, c (Å)	49.3, 50.4, 64.7	74.2, 84.0, 93.4	71.5, 75.2, 104.7	42.8, 49.0, 62.2	49.9, 51.0, 65.1
α, β, γ (°)	69.8, 79.9, 63.9	90.0, 90.0, 90.0	90.0, 90.0, 90.0	90.0, 93.0, 90.0	69.9, 79.2, 63.8
Resolution (Å)[a]	50.00-2.15 (2.23-2.15)	50.00-2.49 (2.57-2.49)	50.00-1.98 (2.05-1.98)	38.50-1.88 (1.95-1.88)	43.73-1.82 (1.88-1.82)
No of measured reflections[a]	106080 (10611)	138108 (12287)	549734 (47124)	157237 (13625)	170958 (18450)
No of unique reflections[a]	26435 (2620)	20046 (1838)	39840 (3864)	21088 (1982)	45725 (4212)
Redundancy[a]	4.0 (4.0)	6.9 (6.7)	13.8 (12.2)	7.5 (6.9)	3.7 (4.4)
Completeness (%)[a]	93.1 (92.6)	94.6 (89.5)	99.4 (98.1)	99.3 (93.3)	93.8 (86.3)
Average (I/σ)[a]	10.3 (6.4)	9.6 (3.4)	21.1 (5.1)	8.6 (2.6)	12.2 (6.3)
R_{merge} (%)[a, b]	10.59 (19.83)	15.69 (53.79)	9.65 (44.92)	16.78 (69.22)	6.88 (21.54)
**Refinement**					
No of reflections[a]	26426 (2620)	20014 (1836)	39780 (3864)	21084 (1982)	45719 (4212)
R_{work}/R_{free}[c]	0.1853/0.2186	0.2320/0.2781	0.1637/0.2125	0.1585/0.2066	0.1410/0.1898
**No of non-H atoms**					
protein	4602	4630	4562	2247	4733
solvent	179	58	621	277	701
ligand	32	21	45	50	121
Average B factor (Å^2)	20.35	26.49	24.02	22.00	14.63
**RMS deviations**					
Bond lengths (Å)	0.006	0.005	0.009	0.008	0.012
Bond angles (°)	0.65	0.65	0.99	0.90	1.07

Ramachandran plot favored (%)	99.48	98.46	99.65	99.64	99.66
Ramachandran plot allowed (%)	0.52	1.54	0.35	0.36	0.34
Ramachandran plot outliers (%)	0.00	0.00	0.00	0.00	0.00

[a] Numbers in parentheses are values for the highest-resolution shell.

[b] $R_{merge} = \sum_{hkl}\sum_i|I_i - \langle I\rangle|/\sum_{hkl}\sum_i|\langle I\rangle|$, where I_i is the intensity of the ith observation of an equivalent reflection with indices h, k, and l.

[c] R_{free} was calculated with the 5% of reflections set that was not included in refinement.

3. Supplementary Methods

3.1 Preparation and characterization of starting materials

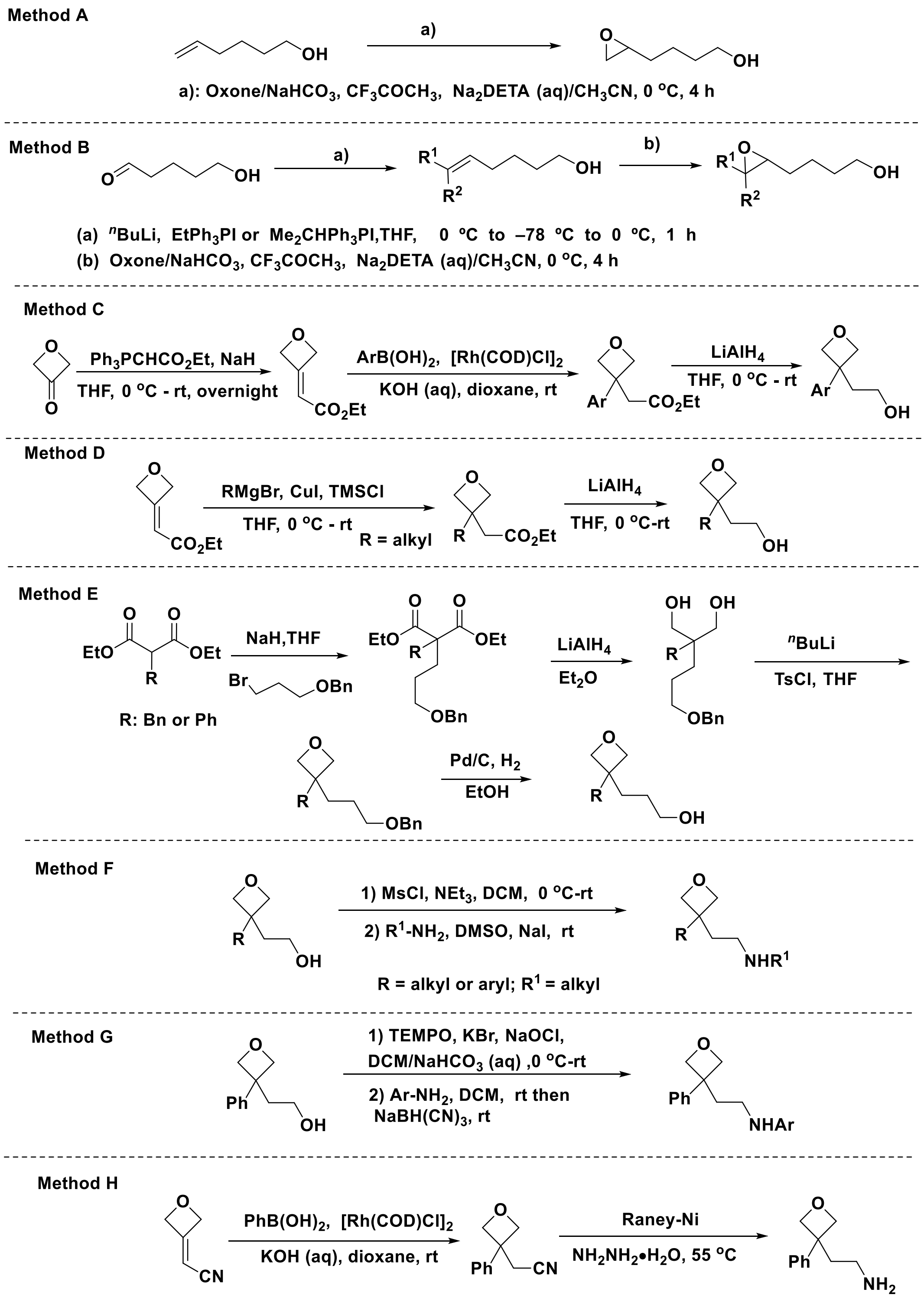

Supplementary Figure 8 Synthesis scheme of starting materials.

3.1.1 General procedure for the synthesis of epoxy alcohols

Synthesis of substrates **1** and 4,5-epoxy alcohols (According to literature procedures with minor revision[1]):

Method A

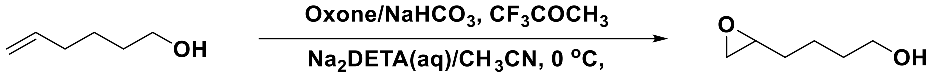

A solution of 5-hexen-1-ol (3.0 g, 30 mmol, 1.0 equiv) in 60 mL of CH_3CN and 60 mL 4.0 mM NaEDTA aqueous buffer was cooled to 0 °C, and 0.84 g (7.5 mmol, 0.25 equiv) of (1,1,1)-trifluoro acetone was added to the precooled solution. A mixture of 40 g (0.48 mol, 16.2 equiv) of $NaHCO_3$ and 62.4 g (105 mmol, 3.5 equiv) of oxone was added to a vigorously stirred biphasic mixture of the solution over three hours. After four hours the solids were filtered off and washed with CH_2Cl_2 (60 mL twice), the combined organic phases were washed with saturated $Na_2S_2O_3$ solution (50 mL twice), saturated $NaHCO_3$ solution (50 mL) and brine (2 x 50 mL) and dried over anhydrous $MgSO_4$. After filtration, the solution was concentrated under reduced pressure and the residue was purified by silica gel column chromatography (eluent: PE/EA = 10:1 to 2:1), and the racemic epoxide was obtained as a colorless liquid.

4-(Oxiran-2-yl)butan-1-ol (1)

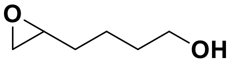

1

1 was prepared according to Method A and isolated as a colorless liquid. The product was purified by silica gel column chromatography by using PE/EA = 10:1 to 2:1 as the eluent (R_f value = 0.12, PE/EA = 2:1). The spectral data were consistent with a previous literature report[1]. **^{1}H NMR** (400 MHz, $CDCl_3$) δ 3.66–3.60 (m, 2H), 2.93–2.88 (m, 1H), 2.76–2.71 (m, 1H), 2.48–2.44 (m, 1H), 1.65–1.49 (m, 6H). **HRMS** (ESI): calcd for $[M + H]^+$ $C_6H_{13}O_2$ 117.0916; found 117.0911.

3-(Oxiran-2-yl)propan-1-ol

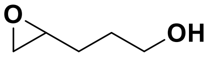

3-(Oxiran-2-yl)propan-1-ol was prepared according to Method A and isolated as a colorless liquid. The product was purified by silica gel pretreated with triethylamine column chromatography by using PE/EA = 10:1 to 2:1 as the eluent (R_f value = 0.12, PE/EA = 2:1). The spectral data were consistent with a previous literature report[1]**^{1}H NMR** (400 MHz, $CDCl_3$) δ 3.73 (t, J = 6.0 Hz, 2H), 3.00 (dd, *J* = 6.8, 3.0 Hz, 1H), 2.82 (t, *J* = 4.4 Hz, 1H), 2.56 (dd, *J* = 4.7, 2.9 Hz, 1H), 2.00 (s, 1H), 1.85 – 1.71 (m, 3H), 1.57 (dt, *J* = 14.0, 6.9 Hz, 1H). **^{13}C NMR** (100 MHz, $CDCl_3$) δ 62.2, 52.4, 47.3, 29.0(2).

3-(3-Methyloxiran-2-yl)propan-1-ol

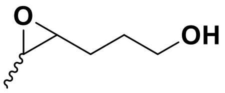

3-(3-Methyloxiran-2-yl)propan-1-ol was prepared according to Method A and isolated as a colorless liquid. The product was purified by silica gel pretreated with triethylamine column chromatography by using PE/EA = 10:1 to 2:1 as the eluent (R_f value = 0.12, PE/EA = 2:1). The spectral data were consistent with a previous literature report[2]**^{1}H NMR** (400 MHz, $CDCl_3$) δ 3.78 – 3.63 (m, 2H), 2.84 (tt, *J* = 5.0, 2.5 Hz, 1H), 2.76 – 2.69 (m, 1H), 1.85 – 1.71 (m, 4H), 1.67 (s, 1H), 1.54 (td, *J* = 13.4, 7.0 Hz, 1H), 1.34 (d, *J* = 5.2 Hz, 4H). **^{13}C NMR** (100 MHz, CDCl3) δ 62.1, 59.7, 54.9, 29.0, 28.5, 17.5.

Synthesis of substrates **5, 7** (According to literature procedures with minor revision[3]):

Method B

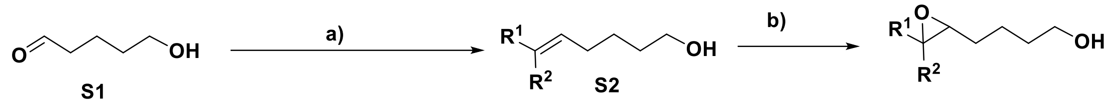

(a) ^{n}BuLi, $EtPh_3PI$ or Me_2CHPh_3PI,THF, 0 ºC to –78 ºC to 0 ºC, 1 h (b) Oxone/$NaHCO_3$, CF_3COCH_3, Na_2DETA (aq)/CH_3CN, 0 ºC, 4 h

To a solution of ethyltriphenylphosphonium iodide (10.0 g, 24 mmol, 2.4 equiv) in dry THF (50 mL) at 0 ºC was added dropwise ^{n}BuLi (15 mL, 24 mmol, 2.4 equiv, 1.6 M in hexane) under atmosphere of nitrogen. The resulting solution was stirred at 0 ºC for 15 min before being cooled to –78 ºC. To this mixture was added another solution of S1 (1.02 g, 10 mmol,1.0 equiv) in dry THF (50 mL) over the course of 15 min. After being stirred at –78 ºC for 30 min, the reaction mixture was transferred to an ice-water bath and was allowed to warm to 0 ºC over the course of 1 h. The reaction was quenched by the addition of saturated aqueous NH_4Cl (50 mL) and diluted with EtOAc (50 mL). The organic layer was separated and the aqueous layer was extracted with EtOAc (3 x 50mL). The combined organic layers were dried over Na_2SO_4, filtered and concentrated under reduced pressure. The crude product was purified by flash chromatography (SiO_2, PE/EA = 6:1, R_f = 0.15) to give **S2** (mixture of stereoisomers Z/E = 2:1) as a colorless oil (0.8 g, 70%).

A solution of **S2** (0.8 g, 7 mmol, 1.0 equiv) in 20 mL of CH_3CN and 20 mL 4.0 mM NaEDTA aqueous buffer was cooled to 0 °C, and 196 mg (1.75 mmol 0.25 equiv) of (1,1,1)-trifluoro acetone was added to the precooled solution. A mixture of 9.8 g (113 mmol, 16.2 equiv) of $NaHCO_3$ and 15.4 g (24.5 mmol, 3.5 equiv) of oxone was added to a vigorously stirred biphasic mixture of the solution over three hours. After four hours the solids were filtered off and washed with CH_2Cl_2 (20 mL twice), the combined organic phases were washed with saturated $Na_2S_2O_3$ solution (25 mL twice), saturated $NaHCO_3$ solution (25 mL) and brine (25 mL twice) and dried over anhydrous $MgSO_4$. After filtration, the solution was concentrated under reduced pressure and the residue was purified by silica gel column chromatography (eluent: PE/EA = 10:1 to 2:1), and the racemic epoxide was obtained as a colorless liquid.

4-(3-Methyloxiran-2-yl)butan-1-ol (5)

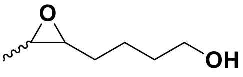

5

5 was prepared according to Method B and isolated as a colorless liquid. The product was purified by silica gel pretreated with triethylamine column chromatography by using PE/EA = 10:1 to 2:1 as the eluent (R_f value = 0.12, PE/EA = 2:1). The spectral data were consistent with a previous literature report[3]. **^{1}H NMR** (400 MHz, $CDCl_3$) δ 3.73 – 3.60 (m, 2H), 3.07 (m, *J* = 5.5, 2.9 Hz, 1H), 2.96 – 2.89 /2.69 – 2.63 (m, 1H), 2.81 – 2.73/2.02 – 1.76 (m, 1H), 1.71 – 1.49 (m, 6H), 1.35 – 1.24 (m, 3H). **HRMS** (ESI): calcd for $[M + H]^+$ $C_7H_{15}O_2$ 131.1072; found 131.1071.

4-(3,3-Dimethyloxiran-2-yl)butan-1-ol (7)

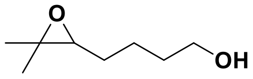

7

7 was prepared according to Method B and isolated as a colorless liquid. The product was purified by silica gel pretreated with triethylamine column chromatography by using PE/EA = 10:1 to 2:1 as the eluent (R_f value = 0.12, PE/EA = 2:1). The spectral data were consistent with a previous literature report[3]. **^{1}H NMR** (400 MHz, $CDCl_3$) δ 3.66 (t, *J* = 6.2 Hz, 2H), 2.74 (t, *J* = 5.4 Hz, 1H), 2.01 (s, 1H), 1.68

– 1.52 (m, 6H), 1.29 (d, *J* = 17.2 Hz, 6H). **HRMS** (ESI): calcd for $[M + H]^+$ $C_8H_{17}O_2$ 145.1227; found 145.1229.

3.1.2 General procedure for the synthesis of oxetane alcohols

Method C :

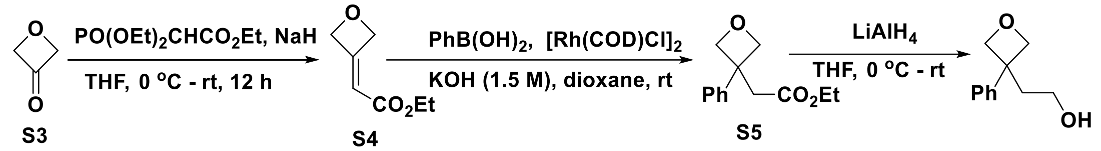

(According to literature procedures with minor revision[4,5]):

Ethyl 2-(oxetan-3-ylidene)acetate (S4)

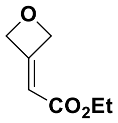

S4

Neat triethyl phosphonoacetate was added to a suspension of NaH (1.1 equiv, 60% dispersion in mineral oil) dry THF. The reaction mixture was stirred at room temperature for 30 minutes and the corresponding ketone (1.0 equiv) was added. The reaction mixture was stirred at room temperature overnight and then carefully quenched with a saturated $NaHCO_3$ solution. The reaction mixture was transferred to a separation funnel using EtOAc/water. The aqueous phase was extracted with EtOAc (three times). The combined organic phases were washed with brine, dried over $MgSO_4$, filtered and concentrated under reduced pressure. Final purification was accomplished by silica gel column chromatography (by using PE/EA = 50:1 to 10:1) to give the desired product **S4** in 85% yield.

Ethyl 2-(3-phenyloxetan-3-yl) acetate (S5)

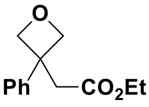

S5

To a solution of $[Rh(COD)Cl]_2$ (0.5 mmol, 0.05 equiv) in dry dioxane (40 mL) was added aqueous KOH (1.5 M, 8.7 mL, 13 mmol, 1.3 equiv), followed by the *α*,*β*-unsaturated ester **S4** (10.0 mmol, 1.0 equiv) and a solution of phenylboronic acid (15.0 mmol, 1.5 equiv) in dry dioxane (60 mL). After stirring for 14 h until full conversion of the starting material (monitored by TLC or GC/MS), the solution was concentrated under reduced pressure, and then EtOAc (50 mL) and brine (50 mL) were added. The aqueous phase was extracted three times with EtOAc (30 mL). The combined organic phases were dried over anhydrous Na_2SO_4 and concentrated under reduced pressure. The residue was purified by silica gel column chromatography to afford S3 in 92% yield. The spectral data were consistent with a previous literature report[4]. **^{1}H NMR** (400 MHz, $CDCl_3$) δ 7.38 (t, *J* = 7.6 Hz, 2H), 7.32 – 7.25 (m, 1H), 7.20 (d, *J* = 7.9 Hz, 2H), 5.06 (d, J = 6.1 Hz, 2H), 4.91 (d, *J* = 6.1 Hz, 2H), 4.04 (q, *J* = 7.1 Hz, 2H), 3.15 (s, 2H), 1.15 (t, *J* = 7.1 Hz, 3H).

2-(3-Phenyloxetan-3-yl)ethan-1-ol (9)

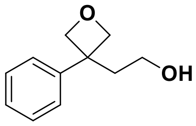

9

Under N_2, to a suspension of $LiAlH_4$ (9.2 mmol, 1.0 equiv) in THF at 0 °C was added dropwise of S3 (3.0 g, 9.2 mmol, 1.0 equiv) in THF (20 mL), the mixture was allowed to warm to room temperature and stirred for another 1 h, then quenched by $Na_2SO_4 \cdot 10H_2O$. The mixture was filtered, and washed with ethyl acetate for several times. The organic layers were combined, dried with Na_2SO_4 and concentrated under reduced pressure, followed by flash column chromatography (PE/EA = 15:1→5:1) to afford the pure alcohol substrate 1.4 g in 90%. The spectral data were consistent with a previous literature report[4]. **^{1}H NMR** (400 MHz, $CDCl_3$) δ

7.50 (t, *J* = 7.5 Hz, 2H), 7.39 (t, *J* = 6.4 Hz, 1H), 7.21 (d, *J* = 7.6 Hz, 2H), 5.16 (d, *J* = 5.7 Hz, 2H), 4.94 (d, *J* = 5.7 Hz, 2H), 3.67 (t, *J* = 6.2 Hz, 2H), 2.49 (t, *J* = 6.5 Hz, 2H), 1.51 (s, 1H). **HRMS** (ESI): calcd for $[M + H]^+$ $C_{11}H_{15}O_2$ 179.1072; found 179.1076.

2-(3-(*p*-Tolyl)oxetan-3-yl)ethan-1-ol (11)

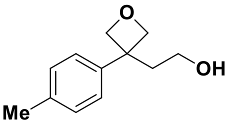

11

From 1.42 g of *α*, *β*-unsaturated ester, 1.46 g of **11** was obtained as a colorless oil in 76% yield with two steps according to Method C, column chromatography (silica gel, PE/EA = 5:1→1:1).

^{1}H NMR (400 MHz, $CDCl_3$) δ 7.17 (d, *J* = 8.1 Hz, 2H), 6.96 (d, *J* = 8.0 Hz, 2H), 4.99 (d, *J* = 5.7 Hz, 2H), 4.77 (d, *J* = 5.7 Hz, 2H), 3.53 (t, *J* = 6.5 Hz, 2H), 2.48 – 2.17 (m, 5H), 1.33 (s, 1H). **^{13}C NMR** (100 MHz, $CDCl_3$) δ 141.6, 136.2, 129.4, 125.7, 82.5, 59.8, 46.0, 43.3, 21.1; **HRMS** (ESI): calcd for $[M + H]^+$ $C_{12}H_{17}O_2$ 193.1229; found 193.1224.

2-(3-(*m*-Tolyl)oxetan-3-yl)ethan-1-ol (13)

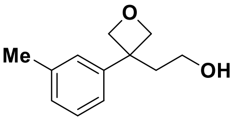

13

From 1.42 g of *α*, *β*-unsaturated ester, 1.44 g of **13** was obtained as a colorless oil in 75% yield with two steps according to Method C, column chromatography (silica gel, PE/EA = 5:1→1:1). **^{1}H NMR** (400 MHz, $CDCl_3$) δ 7.35 – 7.20 (m, 1H), 7.11 (d, *J* = 7.6 Hz, 1H), 6.90 (d, *J* = 5.8 Hz, 2H), 5.05 (d, *J* = 5.7 Hz, 2H), 4.81 (d, *J* = 5.7 Hz, 2H), 3.58 (t, *J* = 5.8 Hz, 2H), 2.47 – 2.30 (m, 5H),1.32 (S, 1H). **^{13}C NMR** (100 MHz, $CDCl_3$) δ 144.5, 138.3, 128.5, 127.3, 126.4, 122.7, 82.3, 59.5, 43.2, 21.6; **HRMS** (ESI): calcd for $[M + H]^+$ $C_{12}H_{17}O_2$ 193.1229; found 193.1228.

2-(3-(3,5-Dimethylphenyl)oxetan-3-yl)ethan-1-ol (15).

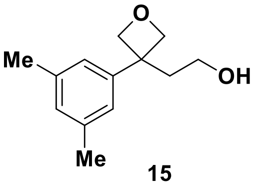

15

From 1.42 g of *α*, *β*-unsaturated ester, 1.69 g of **15** of was obtained as a white solid in 82% yield with two steps according to Method C, column chromatography (silica gel, PE/EA = 5:1→1:1). **^{1}H NMR** (400 MHz, $CDCl_3$) δ 6.88 (s, 1H), 6.65 (s, 2H), 4.99 (d, *J* = 5.7 Hz, 2H), 4.74 (d, *J* = 5.7 Hz, 2H), 3.54 (t, *J* = 5.8 Hz, 2H), 2.57 – 2.12 (m, 9H), 1.48 (s, 1H). **^{13}C NMR** (100 MHz, $CDCl_3$) δ 144.6, 138.2, 128.2, 123.4, 82.3, 59.7, 46.1, 43.3, 21.4; **HRMS** (ESI): calcd for $[M + H]^+$ $C_{13}H_{19}O_2$ 207.1385; found 207.1386.

2-(3-(4-Ethylphenyl)oxetan-3-yl)ethan-1-ol (17)

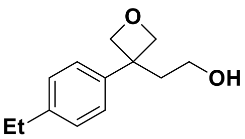

17

From 1.42 g of *α*, *β*-unsaturated ester, 1.70 g of **17** was obtained as a white solid in 83% yield with two steps according to Method C, column chromatography (silica gel, PE/EA = 5:1→1:1). **^{1}H NMR** (400 MHz, $CDCl_3$) δ 7.19 (d, *J* = 7.8 Hz, 2H), 6.98 (d, *J* = 7.9 Hz, 2H), 5.00 (d, *J* = 5.6 Hz, 2H), 4.77 (d, *J* = 5.6 Hz, 2H), 3.53 (t, *J* = 5.8 Hz, 2H), 2.64 (q, *J* = 7.6 Hz, 2H), 2.33 (t, *J* = 6.5 Hz, 2H), 1.46 (s, 1H), 1.24 (t, *J* = 7.6 Hz, 3H). **^{13}C NMR** (100 MHz, $CDCl_3$) δ 142.6 141.8, 128.2125.7 82.5 59.7, 46.0, 43.3 28.5, 15.8, 15.7; **HRMS** (ESI): calcd for $[M + H]^+$ $C_{13}H_{19}O_2$ 207.1385; found 207.1387.

2-(3-(4-Methoxyphenyl)oxetan-3-yl)ethan-1-ol (19)

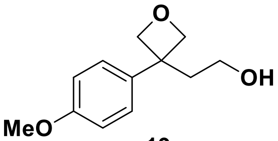

19

From 1.42 g of *α*, *β*-unsaturated ester,1.64 g of **19** was obtained as a colorless oil in 79% yield with two steps according to Method C, column chromatography (silica gel, PE/EA = 5:1→1:1). **^{1}H NMR** (400 MHz, $CDCl_3$) δ 7.04 (d, *J* = 8.6 Hz, 2H), 6.93 (d, *J* = 8.6 Hz, 2H), 5.01 (d, *J* = 5.7 Hz, 2H), 4.80 (d, *J* = 5.7 Hz, 2H), 3.84 (s, 3H), 3.55 (t, *J* = 5.5 Hz, 2H), 2.35 (t, *J* = 6.5 Hz, 2H), 1.49 (s, 1H). **^{13}C NMR** (100 MHz, $CDCl_3$) δ 136.6, 126.8, 114.0, 82.5, 82.5, 59.7, 59.6, 55.3, 45.6, 45.6, 45.6, 43.2; **HRMS** (ESI): calcd for $[M + H]^+$ $C_{12}H_{17}O_3$ 209.1178; found 209.1178.

2-(3-(4-(Methylthio)phenyl)oxetan-3-yl)ethan-1-ol (21)

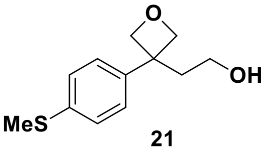

21

From 1.42 g of *α*, *β*-unsaturated ester, 1.93 g of **21** was obtained as a a colorless oil in 86% yield with two steps according to Method C, column chromatography (silica gel, PE/EA = 5:1→1:1). **^{1}H NMR** (400 MHz, $CDCl_3$) δ 7.40 – 7.22 (m, 2H), 7.23 – 7.00 (m, 2H), 5.00 (d, *J* = 5.8 Hz, 2H), 4.81 (d, *J* = 5.8 Hz, 2H), 3.54 (dd, *J* = 11.3, 6.4 Hz, 2H), 2.51 (s, 3H), 2.35 (t, *J* = 6.5 Hz, 2H), 1.44 (s, 1H). **^{13}C NMR** (100 MHz, $CDCl_3$) δ 141.4, 136.5, 126.9, 126.3, 82.3, 59.4, 45.9, 43.0, 16.0; **HRMS** (ESI): calcd for $[M + H]^+$ $C_{12}H_{17}O_2S$ 225.0949; found 225.0947.

2-(3-(Naphthalen-2-yl)oxetan-3-yl)ethan-1-ol (23)

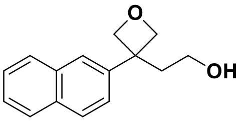

23

From 1.42 g of *α, β*-unsaturated ester, 1.76 g of **23** was obtained as a colorless oil in 77% yield with two steps according to Method C, column chromatography (silica gel, PE/EA = 5:1→1:1). **^{1}H NMR** (400 MHz, $CDCl_3$) δ 7.92 – 7.66 (m, 3H), 7.56 – 7.37 (m, 3H), 7.24 – 7.04 (m, 1H), 5.11 (d, *J* = 5.7 Hz, 2H), 4.87 (d, *J* = 5.7 Hz, 2H), 3.51 (t, *J* = 6.5 Hz, 2H), 2.41 (t, *J* = 6.6 Hz, 2H), 1.66 (s, 1H). **^{13}C NMR** (100 MHz, $CDCl_3$) δ 141.8, 133.2, 132.1, 128.6, 127.7, 127.7, 126.5, 125.9, 124.3, 124.0, 82.3, 59.7, 59.7, 59.7, 46.4, 43.0; **HRMS** (ESI): calcd for $[M + H]^+$ $C_{15}H_{17}O_2$ 229.1229; found 229.1225.

2-(3-(4-Fluorophenyl)oxetan-3-yl)ethan-1-ol (25)

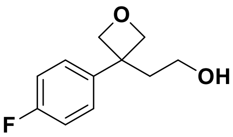

25

From 1.42 g of *α*, *β*-unsaturated ester, 1.07 g of **25** was obtained as a colorless oil in 55% yield with two steps according to Method C, column chromatography (silica gel, PE/EA = 5:1→1:1). **^{1}H NMR** (400 MHz, $CDCl_3$) δ 7.30 – 6.85 (m, 4H), 5.00 (d, *J* = 5.9 Hz, 2H), 4.82 (d, *J* = 5.9 Hz, 2H), 3.55 (t, *J* = 6.5 Hz, 2H), 2.35 (t, *J* = 6.5 Hz, 2H). **^{19}F NMR** (376 MHz, $CDCl_3$) δ -115.7– -115.8 (m). **^{13}C NMR** (100 MHz, $CDCl_3$) δ 161.8 d, *J* = 245.7 Hz), 138.9 (d, *J* = 3.4 Hz), 128.9(d, *J* = 8.0 Hz), 115.5 (d, *J* = 21.2 Hz), 74.4, 69.25, 67.7, 53.0, 34.2; **HRMS** (ESI): calcd for $[M + H]^+$ $C_{11}H_{14}O_2F$ 197.0978; found 197.0979.

2-(3-(4-Chlorophenyl)oxetan-3-yl)ethan-1-ol (27)

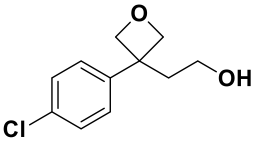

27

From 1.42 g of *α*, *β*-unsaturated ester, 0.98 g of **27** was obtained as a colorless oil in 46% yield with two steps according to Method C, column chromatography (silica gel, PE/EA = 5:1→1:1). **^{1}H NMR** (400 MHz, $CDCl_3$) δ 7.31 (d, *J* = 8.6 Hz, 2H), 7.01 (d, *J* = 8.6 Hz, 2H), 4.94 (d, *J* = 5.9 Hz, 2H), 4.78 (d, *J* = 5.9 Hz, 2H), 3.47 (t, *J* = 6.5 Hz, 2H), 2.29 (t, *J* = 6.5 Hz, 2H), 1.76 (s, 1H). **^{13}C NMR** (100 MHz, $CDCl_3$) δ 143.0, 132.3, 128.7, 127.3, 82.3, 59.3, 45.9, 42.8. **HRMS** (ESI):

calcd for [M + H]+ $C_{11}H_{14}O_2Cl$ 213.0682; found 213.0679.

2-(3-(4-Bromophenyl)oxetan-3-yl)ethan-1-ol (29)

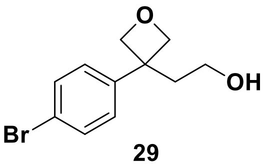

29

From 1.42 g of *α*, *β*-unsaturated ester, 0.97 g of **29** was obtained as a colorless oil in 38% yield with two steps according to Method C, column chromatography (silica gel, PE/EA = 5:1→1:1). **^{1}H NMR** (400 MHz, $CDCl_3$) δ 7.48 (d, *J* = 8.4 Hz, 2H), 6.96 (d, *J* = 8.4 Hz, 2H), 4.95 (d, *J* = 5.8 Hz, 2H), 4.79 (d, *J* = 5.8 Hz, 2H), 3.50 (t, *J* = 6.5 Hz, 2H), 2.30 (t, J = 6.5 Hz, 2H), 1.49 (s, 1H) . **^{13}C NMR** (100 MHz, $CDCl_3$) δ143.7, 143.6, 131.8, 127.7, 120.4, 82.3, 59.5, 46.1, 42.8. **HRMS** (ESI): calcd for [M + H]+ $C_{11}H_{14}O_2Br$ 257.0177; found 257.0172.

2-(3-(4-(Trifluoromethyl)phenyl)oxetan-3-yl)ethan-1-ol (31)

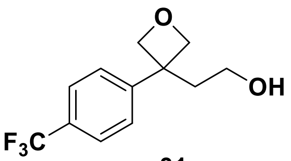

31

From 1.42 g of *α*, *β*-unsaturated ester, 1.18 g of **31** was obtained as a colorless oil in 48% yield with two steps according to Method C, column chromatography (silica gel, PE/EA = 5:1→1:1). **^{1}H NMR** (400 MHz, $CDCl_3$) δ 7.74 (d, *J* = 8.0 Hz, 2H), 7.33 (d, *J* = 8.0 Hz, 2H), 5.12 (d, *J* = 5.7 Hz, 2H), 4.96 (d, *J* = 5.7 Hz, 2H), 3.85 – 3.39 (m, 2H), 2.47 (t, *J* = 6.3 Hz, 2H), 1.51 (s, 1H). **^{19}F NMR** (376 MHz, $CDCl_3$) δ -62.35 (s). **^{13}C NMR** (100 MHz, $CDCl_3$) δ 148.6, 128.9 (q, *J* = 32.5 Hz), 126.3 (s), 125.6 (q, *J* = 3.8 Hz), 124.1 (q, *J* = 272.1 Hz), 82.1, 59.5, 46.5, 42.7; **HRMS** (ESI): calcd for [M + H]+ $C_{12}H_{14}O_2F_3$ 247.0946; found 247.0948.

Method D

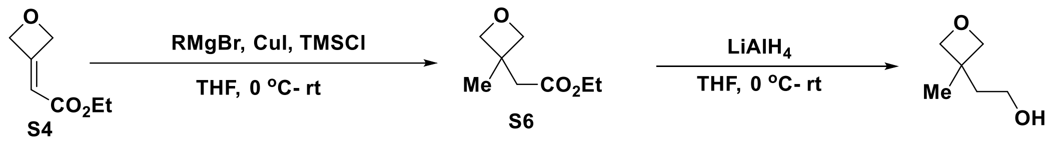

Under N_2, to a solution of ethyl 2-(oxetan-3-ylidene)acetate **S4** (1.42 g, 10 mmol, 1.0 equiv) and CuI (165 mg, 1 mmol, 0.1 equiv) in 10 mL THF was added TMSCl (1.0 mL, 20 mmol, 2.0 equiv). After stirring for 20 min at room temperature, the mixture was cooled to –15 °C, then a solution of Methyl magnesium Bromide (12 mL, 1 M in Et_2O) was added dropwise over 1 h. The resulting solution was stirred at room temperature for another 2 h and then quenched by saturated aqueous NH_4Cl solution (15 mL). The mixture was diluted with diethyl ether (15 mL). The organic layer was separated, and the aqueous layer was extracted with diethyl ether (10 mL) for three times. The combined organic layers were dried over anhydrous Na_2SO_4, and concentrated under reduced pressure. The residue was purified by silica gel column chromatography to give **S6**. The spectral data were consistent with a previous literature report[3].**^{1}H NMR** (400 MHz, $CDCl_3$) δ 4.62 (d, *J* = 6.0 Hz, 1H), 4.45 (d, *J* = 6.0 Hz, 2H), 4.17 (q, *J* = 7.1 Hz, 2H), 2.70 (s, 2H), 1.44 (s, 3H), 1.29 (t, *J* = 7.1 Hz, 3H).

2-(3-Methyloxetan-3-yl)ethan-1-ol (37)

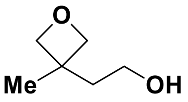

37

The reduction of the ester group is consistent with method C. From 1.42 g of *α*, *β*-unsaturated ester, 0.42 g of **37** was obtained as a colorless oil in 36% yield with two steps according to Method D**,** column chromatography (silica gel, PE/EA = 5:1→1:1). **^{1}H NMR** (400 MHz, $CDCl_3$) δ 4.58 (d, *J* = 5.7 Hz, 2H), 4.40 (d, *J* = 5.7 Hz, 2H), 3.77

(t, J = 6.8 Hz, 2H), 1.96 (t, J = 6.8 Hz, 2H), 1.72 (s, 1H), 1.39 (s, 3H). **^{13}C NMR** (100 MHz, $CDCl_3$) δ 83.3, 59.4, 41.0, 38.1, 23.3; **HRMS** (ESI): calcd for $[M + H]^+$ $C_6H_{13}O_2$ 399.1873; found 399.1878.

2-(3-Ethyloxetan-3-yl)ethan-1-ol (35)

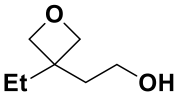

35

From 1.42 g of *α*, *β*-unsaturated ester, 0.52 g of **35** was obtained as a colorless oil in 40% yield with two steps according to Method D**,** column chromatography (silica gel, PE/EA = 5:1→1:1).

^{1}H NMR (400 MHz, $CDCl_3$) δ 4.52 (d, J = 5.8 Hz, 2H), 4.42 (d, J = 5.8 Hz, 2H), 3.75 (t, J = 6.9 Hz, 2H), 1.97 (t, J = 6.9 Hz, 2H), 1.78 (q, J = 7.5 Hz, 2H), 1.67 (s, 1H), 0.92 (t, J = 7.5 Hz, 3H). **^{13}C NMR** (100 MHz, $CDCl_3$) δ 81.9, 59.6, 41.9, 37.7, 28.8, 8.6; **HRMS** (ESI): calcd for $[M + H]^+$ $C_7H_{15}O_2$ 131.1072; found 131.1073.

2-(3-Benzyloxetan-3-yl)ethan-1-ol (33)

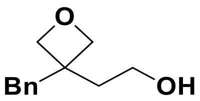

33

From 1.42 g of *α*, *β*-unsaturated ester, 0.92 g of **33** was obtained as a colorless oil in 48% yield with two steps according to Method D**,** column chromatography (silica gel, PE/EA = 5:1→1:1). **^{1}H NMR** (400 MHz, $CDCl_3$) δ 7.35 – 7.26 (m, 3H), 7.17 (d, J = 7.4 Hz, 2H), 4.67 (d, J = 5.9 Hz, 2H), 4.55 (d, J = 5.9 Hz, 2H), 3.87 (t, J = 6.8 Hz, 2H), 3.07 (s, 2H), 1.94 (t, J = 6.8 Hz, 2H), 1.59 (s, 1H). **^{13}C NMR** (100 MHz, $CDCl_3$) δ 137.8, 129.5, 128.5, 126.6, 81.2, 59.1, 42.3, 42.1, 37.7; **HRMS** (ESI): calcd for $[M + H]^+$ $C_{12}H_{17}O_2$ 193.1229; found 193.1227.

Synthesis of substrates 41 and 43:

Method E

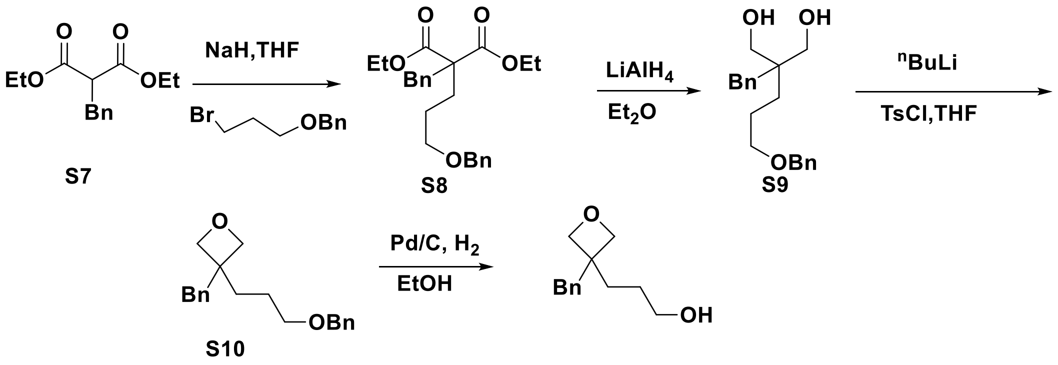

(According to literature procedures with minor revision[4])

Diethyl 2-benzyl-2-(3-(benzyloxy)propyl)malonate (S8)

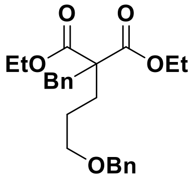

S8

An oven-dried round-bottomed flask charged with of the corresponding diethyl 2-benzylmalonate **S7** (14.3 g, 57 mmol, 1.9 equiv), NaH (1.4 g, 36 mmol, 1.2 equiv) and anhydrous THF (200 mL), The resulting mixture was stirred at rt for 30 min, the solution benzyl 3-bromopropyl ether (6.9 g, 30 mmol, 1.0 equiv) was added dropwise via a syringe under a dry N_2 atmosphere. The reaction mixture was heated to reflux for next 48 h, then quenched by water. The aqueous layer was extracted with ethyl acetate (100 mL) for two times, and then the organic layers were combined, dried with Na_2SO_4 and concentrated under reduced pressure, The product was purified via column

chromatography (1:1 CH_2Cl_2: hexanes) to yield the product **S8** as a colorless oil (8.5 g; 72% yield). ^{1}**H NMR** (400 MHz, $CDCl_3$) δ 7.43 – 7.30 (m, 5H), 7.26 – 7.22 (m, 3H), 7.15 – 7.11 (m, 2H), 4.52 (s, 2H), 4.20 (q, *J* = 7.1 Hz, 4H), 3.50 (t, *J* = 6.5 Hz, 2H), 3.28 (s, 2H), 1.90 (dd, *J* = 11.1, 5.9 Hz, 2H), 1.75 – 1.61 (m, 2H), 1.26 (t, *J* = 7.1 Hz, 6H). ^{13}**C NMR** (101 MHz, $CDCl_3$) δ 171.2, 138.5, 136.2, 130.0, 128.4, 128.3, 127.6, 127.5, 126.9, 72.9, 70.2, 61.3, 58.6, 38.1, 28.6, 24.7, 14.1; **HRMS** (ESI): calcd for $[M + H]^+$ $C_{24}H_{31}O_5$ 399.2171; found 399.2168.

2-Benzyl-2-(3-(benzyloxy)propyl)propane-1,3-diol (S9)

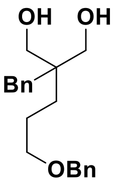

S9

Under N_2, to a suspension of $LiAlH_4$ (42 mmol, 2.1 equiv) in 100 mL THF at 0 ^{o}C was added dropwise of diethyl 2-benzyl-2-(3-(benzyloxy)propyl)malonate **S8** (8.0 g, 20 mmol, 1.0 equiv) in THF (30 mL), the mixture was allowed to warm to room temperature and stirred for another 2 h, then quenched by $Na_2SO_4 \cdot 10H_2O$. The mixture was filtered, and washed with ethyl acetate for several times. The organic layers were combined, dried with Na_2SO_4 and concentrated under reduced pressure, followed by flash column chromatography (PE/EA = 5:1→1:1) to afford the diol product **S9** (5 g; 79% yield). ^{1}**H NMR** (400 MHz, $CDCl_3$) δ 7.45 – 7.33 (m, 5H), 7.31 – 7.19 (m, 5H), 4.55 (s, 2H), 3.64 – 3.46 (m, 6H), 3.21 – 3.07 (m, 3H), 2.65 (s, 2H), 1.81 – 1.62 (m, 2H), 1.38 – 1.14 (m, 2H). ^{13}**C NMR** (100 MHz, $CDCl_3$) δ 138.2, 137.7, 130.5, 128.5, 128.1, 127.8, 127.7, 126.2, 73.2, 71.1, 67.4, 42.6, 37.2, 27.0, 23.3; **HRMS** (ESI): calcd for $[M + H]^+$ $C_{20}H_{26}O_3$ 337.1780; found 337.1781.

3-Benzyl-3-(3-(benzyloxy)propyl)oxetane (S10)

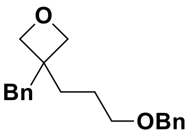

S10

To a solution of 2-(2-(benzyloxy)ethyl)propane-1,3-diol **S9** (5 g, 16 mmol, 1.0 equiv) in THF (100 mL) was added dropwise ^{n}BuLi (6.4 mL, 2.5 M in hexanes, 1.0 equiv) at 0 °C, and the resulting suspension was stirred at 0 °C for 30 minutes before *p*-toluenesulfonyl chloride (3.1g, 16 mmol, 1 equiv) in the THF (25 mL) was added via cannula. The reaction was allowed to continue at 0 °C and gradually turning clear. After 1 hour, a solution of ^{n}BuLi (6.4 mL, 2.5 M in hexanes, 16 mmol, 1 equiv) was added over 15 minutes, and the reaction was then warmed to 60 °C for 8 h. The reaction was cooled to room temperature, diluted with Et_2O and quenched by the addition of water. The organic layer was removed, and the aqueous layer was extracted 3x with Et_2O. The combined organic layers were washed with brine, dried over $MgSO_4$, filtered, concentrated under vacuum, and purified by flash column chromatography (PE/EA = 5:1→1:1) to afford the corresponding oxetane (2.8 g, 59% yield). ^{1}**H NMR** (400 MHz, $CDCl_3$) δ 7.47 – 7.28 (m, 8H), 7.20 (d, *J* = 7.3 Hz, 2H), 4.66 (d, *J* = 5.8 Hz, 2H), 4.56 (s, 2H), 4.45 (d, *J* = 5.8 Hz, 2H), 3.62 – 3.49 (m, 2H), 1.82 – 1.72 (m, 4H). ^{13}**C NMR** (100 MHz, $CDCl_3$) δ 138.5, 138.0, 129.5, 128.4, 127.6, 127.6, 126.5, 80.9, 73.0, 70.5, 43.2, 41.7, 32.1, 24.7; **HRMS** (ESI): calcd for $[M + H]^+$ $C_{20}H_{24}O_2$ 319.1680; found 319.1674.

3-(3-Benzyloxetan-3-yl)propan-1-ol (43)

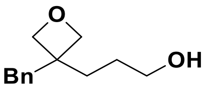

43

To a solution of 3-benzyl-3-(3-(benzyloxy)propyl)oxetane (2.8 g, 9.4 mmol, 1.0 equiv) in EtOH (30 mL) was added 10% Pd/C (0.6 g, 0.56 mmol, 0.06 equiv). The round-bottomed flask was then evacuated and backfilled with hydrogen for 3 times and stirred under an atmosphere of hydrogen (balloon) for 24 h. The black suspension was passed through a plug of celite eluting with ethyl acetate, concentrated and the residue was purified by by flash column chromatography (PE/EA = 5:1→1:1) to afford the corresponding

product as a colorless oil (1.5 g, 76%). **^{1}H NMR** (400 MHz, $CDCl_3$) δ 7.37 – 7.31 (m, 2H), 7.28 (dd, *J* = 8.7, 5.9 Hz, 1H), 7.18 (d, *J* = 7.0 Hz, 2H), 4.67 (d, *J* = 5.9 Hz, 2H), 4.44 (d, *J* = 5.9 Hz, 2H), 3.68 (t, *J* = 5.2 Hz, 2H), 3.04 (s, 2H), 2.07 (s, 1H), 1.74 – 1.63 (m, 2H). **^{13}C NMR** (100 MHz, $CDCl_3$) δ 137.9, 129.4, 128.5, 126.5, 80.9, 62.9, 43.1, 41.7, 31.7, 27.4; **HRMS** (ESI): calcd for $[M + H]^+$ $C_{13}H_{19}O_2$ 207.1380; found 207.1385.

3-(3-Phenyloxetan-3-yl)propan-1-ol (41)

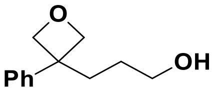

41

3-(3-phenyloxetan-3-yl)propan-1-ol was synthesized from diethyl phenylmalonate (13.5 g, 57 mmol, 1.0 equiv) and benzyl 3-bromopropyl ether (6.9 g, 30 mmol, 1.0 equiv) using method E. The corresponding product **41** was obtained as a colorless oil in 25% yield with four steps. **^{1}H NMR** (400 MHz, $CDCl_3$) δ 7.38 (t, *J* = 7.7 Hz, 2H), 7.27 (t, *J* = 7.4 Hz, 1H), 7.05 (d, *J* = 8.1 Hz, 2H), 5.03 (d, *J* = 5.6 Hz, 2H), 4.70 (d, *J* = 5.6 Hz, 2H), 3.61 (t, *J* = 6.5 Hz, 2H), 2.27 – 2.11 (m, 2H), 1.76 (s, 1H), 1.51 – 1.39 (m, 2H). **^{13}C NMR** (100 MHz, $CDCl_3$) δ 144.8, 128.5, 126.4, 125.7, 81.8, 62.8, 47.2, 37.5, 27.8; **HRMS** (ESI): calcd for $[M + H]^+$ $C_{12}H_{17}O_2$ 193.1232; found 193.1229.

3.1.3 General procedure for the synthesis of oxetane amines

Method F

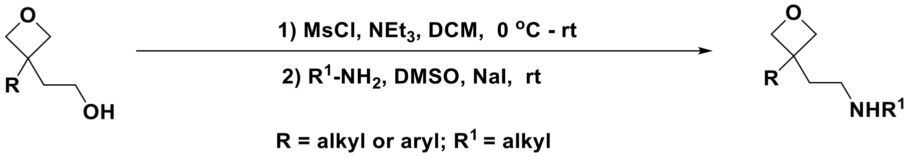

(According to literature procedures with minor revision[6])

To a solution of oxetane alcohols（4 mmol, 1.0 equiv）in CH_2Cl_2 (15 mL) was added Et_3N (10 mmol, 2.5 equiv) and MsCl (6 mmol, 1.5 equiv) at 0 °C, and the mixture was stirred for 10 min at room temperature. Then, saturated aqueous $NaHCO_3$ was added at 0 °C, and the mixture was extracted three times with CH_2Cl_2. The combined organic layer was dried over anhydrous $MgSO_4$, filtered, and concentrated under reduced pressure. The resulting crude product was used immediately in the next reaction without purification. To a solution of the above crude product in DMSO (0.5 M) were added alkyl amine (12 mmol 3.0 eq) and NaI (10 mol%) at room temperature, and the mixture was stirred for 2 days at ambient temperature. Then, the mixture was diluted with H_2O, and extracted with Et_2O. The combined organic layer was washed with brine three times, dried over anhydrous Na_2SO_4, filtered, and concentrated under reduced pressure. The resulting residue was purified by column chromatography to give oxetane amines. (Note that a small amount of the substrate may be spontaneously cyclized to the tetrahydropyrrole product.）

***N*-Benzyl-2-(3-phenyloxetan-3-yl)ethan-1-amine (45)**

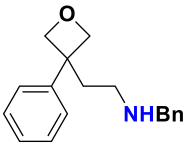

45

From 4 mmol of the oxetane alcohols according to Method F. Light yellow liquid; 75% yield; column chromatography (silica gel, PE/EA = 3:1→1:1 and then MeOH/DCM = 75:1→15:1)**.** **^{1}H NMR** (400 MHz, $CDCl_3$) δ 7.43 – 7.32 (m, 4H), 7.30 – 7.21 (m, 4H), 7.11 – 7.04 (m, 2H), 5.03 (d, *J* = 5.7 Hz, 2H), 4.77 (d, *J* = 5.7 Hz, 2H), 3.72 (s, 2H), 2.53 (dd, *J* = 8.5, 6.4 Hz, 2H), 2.39 – 2.31 (m, 2H), 1.44 (s, 1H).

^{13}C NMR (100 MHz, $CDCl_3$) δ 144.8, 140.2, 128.5, 128.4, 128.1, 127.0, 126.4, 125.7, 82.2, 54.1, 46.7, 45.4, 41.2. **HRMS** (ESI): calcd for $[M + H]^+$ 268.1701; found 268.1706.

***N*-(2-(3-Phenyloxetan-3-yl)ethyl)prop-2-en-1-amine (47)**

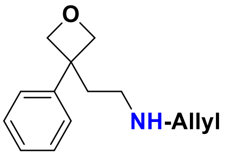

47

From 4 mmol of the oxetane alcohols according to Method F. Light yellow liquid; 64% yield; column chromatography (silica gel, PE/EA = 3:1→1:1 and then MeOH/DCM = 75:1→15:1). **^{1}H NMR** (400 MHz, $CDCl_3$) δ 7.40 – 7.32 (m, 2H), 7.27 – 7.21 (m, 1H), 7.11 – 7.02 (m, 2H), 5.89 – 5.73 (m, 1H), 5.16 – 5.02 (m, 2H), 5.00 (d, *J* = 5.7 Hz, 2H), 4.73 (d, *J* = 5.7 Hz, 2H), 3.15 (d, *J* = 6.0 Hz, 2H), 2.46 (dd, *J* = 9.0, 6.2 Hz, 2H), 2.36 – 2.25 (m, 2H), 1.71 – 1.42 (m, 1H). **^{13}C NMR** (100 MHz, $CDCl_3$) δ 144.7, 136.6, 128.5, 128.5, 128.5, 126.4, 125.6, 125.6, 115.8, 82.1, 52.4, 46.6, 45.1, 41.2. **HRMS** (ESI): calcd for $[M + H]^+$ $C_{14}H_{20}NO$ 218.1545; found 218.1547.

***N*-(2-(3-Phenyloxetan-3-yl)ethyl)prop-2-yn-1-amine (49)**

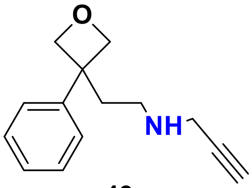

49

From 4 mmol of the oxetane alcohols according to Method F. Light yellow liquid; 57% yield; column chromatography (silica gel, PE/EA = 3:1→1:1 and then MeOH/DCM = 75:1→15:1). **^{1}H NMR** (400 MHz, $CDCl_3$) δ 7.39 (t, *J* = 7.6 Hz, 2H), 7.31 – 7.25 (m, 1H), 7.09 (d, *J* = 7.2 Hz, 2H), 5.04 (d, *J* = 5.7 Hz, 2H), 4.76 (d, *J* = 5.7 Hz, 2H), 3.37 (d, *J* = 2.4 Hz, 2H), 2.61 – 2.50 (m, 2H), 2.43 – 2.25 (m, 2H). **^{13}C NMR** (100 MHz, $CDCl_3$) δ 144.6, 128.6, 126.5, 125.7, 82.1, 81.9, 71.5, 46.6, 44.5, 40.9, 38.1. **HRMS** (ESI): calcd for $[M + H]^+$ $C_{14}H_{18}NO$ 216.1388; found 216.1386.

***N*-(2-(3-Phenyloxetan-3-yl)ethyl)butan-1-amine (51)**

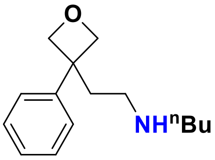

51

From 4 mmol of the oxetane alcohols according to Method F. Light yellow liquid; 68% yield; column chromatography (silica gel, PE/EA = 3:1→1:1 and then MeOH/DCM = 75:1→15:1). **^{1}H NMR** (400 MHz, $CDCl_3$) δ 7.36 (t, *J* = 7.5 Hz, 2H), 7.26 (dd, *J* = 8.3, 6.5 Hz, 1H), 7.10 – 7.03 (m, 2H), 5.00 (d, *J* = 5.7 Hz, 2H), 4.73 (d, *J* = 5.7 Hz, 2H), 2.58 – 2.44 (m, 5H), 2.34 (dd, *J* = 9.2, 6.1 Hz, 2H), 1.49 – 1.39 (m, 2H), 1.36 – 1.24 (m, 2H), 0.90 (t, *J* = 7.3 Hz, 3H). **^{13}C NMR** (100 MHz, $CDCl_3$) δ 144.6, 128.6, 126.4, 125.6, 82.0, 49.6, 46.5, 45.6, 40.8, 31.8, 20.4, 14.0. **HRMS** (ESI): calcd for $[M + H]^+$ $C_{15}H_{24}NO$ 234.1858; found 234.1859.

Method G

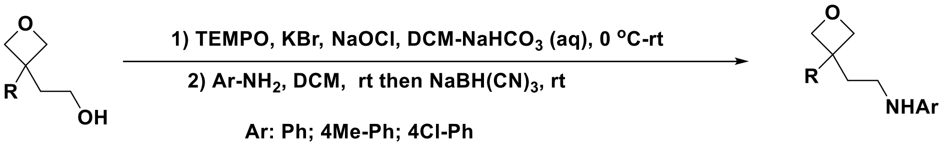

To a cooled and well stirred mixture of oxetane alcohols and TEMPO (1 mol%) in CH_2Cl_2 (0.2 M) and saturated aqueous $NaHCO_3$ containing KBr (10 mol%) was added dropwise a pre-mixed solution of $NaOCl·5H_2O$ (1.5 eq) in saturated aqueous $NaHCO_3$, and the resulting mixture was stirred for 20 min at 0 °C. Then, saturated aqueous $Na_2S_2O_3$ was added at 0 °C, and the mixture was extracted with CH_2Cl_2. The combined organic layer was washed with brine, dried over $MgSO_4$, filtered, and concentrated under reduced pressure. The resulting crude product was used immediately in the next reaction without

purification. To a solution of the above crude product in CH_2Cl_2 were added $ArNH_2$ (1 eq). After the mixture was stirred at room temperature, $NaBH(CN)_3$ (1.2 eq) was added at 0 °C and stirred at room temperature. Then saturated aqueous $NaHCO_3$ was added, and the resulting mixture extracted with CH_2Cl_2. The combined organic layer was dried over anhydrous Na_2SO_4, filtered, and concentrated under reduced pressure. The resulting residue was purified by silica gel column chromatography to give oxetane amines.

(Note that these substrates cyclize spontaneously and require cryopreservation).

_N_-(2-(3-Phenyloxetan-3-yl)ethyl)aniline (53)

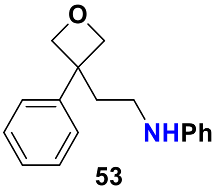

53

From 4 mmol of the oxetane alcohols according to Method G. white solid; 42% yield; column chromatography (silica gel pretreated with 1% Et_3N, PE/Et_2O = 15:1→3:1). **^{1}H NMR** (400 MHz, $CDCl_3$) δ 7.49 (t, *J* = 7.6 Hz, 2H), 7.39 (t, *J* = 7.4 Hz, 1H), 7.24 (t, *J* = 7.8 Hz, 2H), 7.20 – 7.13 (m, 2H), 6.79 (t, *J* = 7.3 Hz, 1H), 6.54 (d, *J* = 8.3 Hz, 2H), 5.13 (d, *J* = 5.7 Hz, 2H), 4.83 (d, *J* = 5.7 Hz, 2H), 3.51 (s, 1H), 3.15 – 2.97 (m, 2H), 2.85 – 2.41 (m, 2H).**^{13}C NMR** (100 MHz, $CDCl_3$) δ 148.1, 144.5, 129.4, 128.9, 126.8, 125.8, 125.7, 117.6, 112.9, 82.0, 46.7, 40.6, 40.1. **HRMS** (ESI): calcd for $[M + H]^+$ $C_{17}H_{20}NO$ 254.1545; found 254.1542.

4-Methyl-_N_-(2-(3-phenyloxetan-3-yl)ethyl)aniline (55)

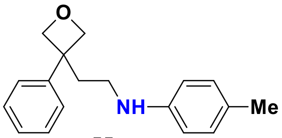

55

From 4 mmol of the oxetane alcohols according to Method G. white solid; 47% yield; column chromatography (silica gel pretreated with 1% Et_3N, PE/Et_2O = 15:1→3:1). **^{1}H NMR** (400 MHz, $CDCl_3$) δ 7.45 – 7.39 (m, 2H), 7.34 – 7.30 (m, 1H), 7.14 – 7.05 (m, 5H), 6.41 – 6.31 (m, 2H), 5.06 (d, *J* = 5.7 Hz, 2H), 4.75 (d, *J* = 5.8 Hz, 2H), 3.42 (s, 1H), 3.04 – 2.89 (m, 2H), 2.47 – 2.37 (m, 2H). **^{13}C NMR** (101 MHz, $CDCl_3$) δ 145.7, 144.5, 129.8, 129.7, 128.7, 126.8, 126.7, 125.6, 113.1, 81.9, 46.6, 40.6, 40.4, 20.4. **HRMS** (ESI): calcd for $[M + H]^+$ $C_{18}H_{22}NO$ 268.1701; found 268.1705.

4-Chloro-_N_-(2-(3-phenyloxetan-3-yl)ethyl)aniline (57)

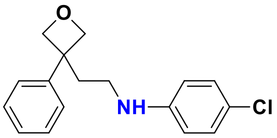

57

From 4 mmol of the oxetane alcohols according to Method G. white solid; 36% yield; column chromatography (silica gel pretreated with 1% Et_3N, PE/Et_2O = 15:1→3:1). **^{1}H NMR** (400 MHz, $CDCl_3$) δ 7.45 – 7.39 (m, 2H), 7.34 – 7.30 (m, 1H), 7.14 – 7.05 (m, 5H), 6.41 – 6.31 (m, 2H), 5.06 (d, *J* = 5.7 Hz, 2H), 4.75 (d, *J* = 5.8 Hz, 2H), 3.42 (s, 1H), 3.04 – 2.89 (m, 2H), 2.47 – 2.37 (m, 2H). **^{13}C NMR** (101 MHz, $CDCl_3$) δ 146.5, 144.3, 129.0, 126.8, 125.6, 122., 113.9, 113.9, 113.8, 81.8, 46.6, 40.3, 40.1. **HRMS** (ESI): calcd for $[M + H]^+$ $C_{17}H_{19}NOCl$ 288.1155 ; found 288.1157.

Method H

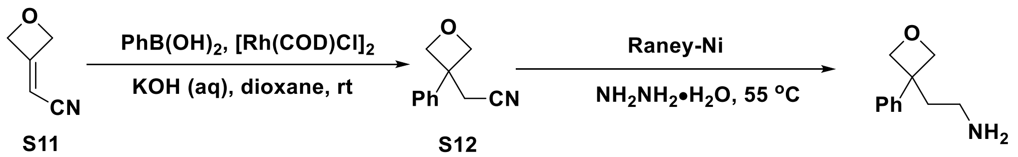

2-(3-Phenyloxetan-3-yl)acetonitrile (S12)

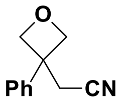

S12

From 0.95 g of **S11**[4] (10 mmol) according to Method C. column chromatography (silica gel, PE/EA = 10:1→3:1). white solid;780 mg, 45% yield; **^{1}H NMR** (400 MHz, $CDCl_3$) δ 7.45 – 7.39 (m, 2H), δ 7.48 – 7.40 (m, 2H), 7.38 – 7.34 (m, 1H), 7.18 – 7.10 (m, 2H), 5.08 (d, *J* = 6.5 Hz, 2H), 4.74 (d, *J* = 6.6 Hz, 2H), 3.11 (s, 2H). **^{13}C NMR** δ 141.5, 129.0, 127.8, 125.5, 117.1, 80.4, 45.4, 29.9. **HRMS** (ESI): calcd for $[M + H]^+$ $C_{11}H_{12}NO$ 174.0915; found 174.0919.

2-(3-Phenyloxetan-3-yl)ethan-1-amine (59)

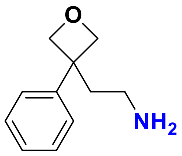

59

To a solution of **S12**（696 mg, 4mmol, 1.0 equiv）in ethanol (15 mL) and warmed to 55 °C was added Raney-nickel (1.6 mL slurry in water) followed by addition of hydrazine monohydrate (2.4 mL). The resulting mixture was allowed to stir at 55°C for 6 h or until the evolution of gas had stopped. The cooled reaction mixture was filtered through diatomaceous earth, rinsed with methanol and dichloromethane. The filtrate was diluted with saturated sodium bicarbonate (50 mL) and extracted with dichloromethane (50 mL × 3). The combined organic layers were dried with sodium sulfate and concentrated. Chromatography with NH_4OH: MeOH: EtOAc (5:10:85) afforded the product (400 mg, 57% yield) as a colorless oil. **^{1}H NMR** (400 MHz, $CDCl_3$) δ 7.37 (t, *J* = 7.6 Hz, 2H), 7.30 – 7.23 (m, 1H), 7.11 – 7.01 (m, 2H), 5.02 (d, *J* = 5.7 Hz, 2H), 4.74 (d, *J* = 5.7 Hz, 2H), 2.57 (dd, *J* = 9.0, 6.4 Hz, 2H), 2.28 (dd, *J* = 9.0, 6.5 Hz, 2H), 1.47 (s, 2H). **^{13}C NMR** (101 MHz, $CDCl_3$) δ 144.75, 128.56, 126.43, 125.60, 82.08, 46.56, 44.95, 38.12. **HRMS** (ESI): calcd for $[M + H]^+$ $C_{11}H_{16}NO$ 178.1232; found 178.1231.

***N*-Benzyl-2-(3-(*p*-tolyl)oxetan-3-yl)ethan-1-amine (61)**

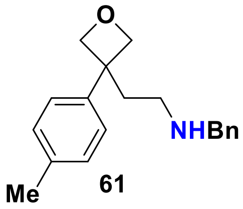

61

From 4 mmol of the oxetane alcohols according to Method F. Light yellow liquid; 58% yield; column chromatography (silica gel, PE/EA = 3:1→1:1 and then MeOH/DCM = 75:1→15:1). **^{1}H NMR** (400 MHz, $CDCl_3$) δ 7.42 – 7.32 (m, 2H), 7.29 (t, *J* = 6.0 Hz, 3H), 7.20 (d, *J* = 8.0 Hz, 2H), 6.99 (d, *J* = 8.1 Hz, 2H), 5.01 (d, *J* = 5.7 Hz, 2H), 4.75 (d, *J* = 5.7 Hz, 2H), 3.72 (s, 2H), 2.58 – 2.51 (m, 2H), 2.40 (s, 3H), 2.38 – 2.32 (m, 2H), 2.14 (s, 1H). **^{13}C NMR** (100 MHz, $CDCl_3$) δ 141.7, 139.9, 136.0, 129.3, 128.5, 128.2, 127.1, 125.6, 82.2, 54.0, 46.3, 45.3, 40.9, 21.1. **HRMS** (ESI): calcd for $[M + H]^+$ $C_{19}H_{24}NO$ 282.1858; found 282.1858.

***N*-Benzyl-2-(3-(*m*-tolyl)oxetan-3-yl)ethan-1-amine (63)**

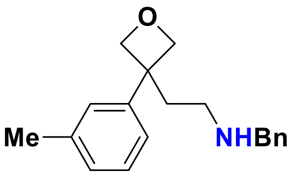

63

From 4 mmol of the oxetane alcohols according to Method F. Light yellow liquid; 55% yield; column chromatography (silica gel, PE/EA = 3:1→1:1 and then MeOH/DCM = 75:1→15:1). **^{1}H NMR** (400 MHz, $CDCl_3$) δ 7.41 – 7.31 (m, 2H), 7.31 – 7.25 (m, 4H), 7.10 (d, *J* = 7.5 Hz, 1H), 6.88 (d, *J* = 7.8 Hz, 2H), 5.03 (d, *J* = 5.7 Hz, 2H), 4.75 (d, *J* = 5.7 Hz, 2H), 3.73 (s, 2H), 2.58 – 2.50 (m, 2H), 2.40 (s, 3H), 2.37 – 2.31 (m, 2H), 1.71 (s, 1H). **^{13}C NMR** (100 MHz, $CDCl_3$) δ 144.7, 140.2, 138.2, 128.4, 128.4, 128.1, 127.2, 127.0, 126.4, 122.8, 82.2, 54.1, 46.6, 45.4, 41.1, 21.6. **HRMS** (ESI): calcd for $[M + H]^+$ $C_{19}H_{24}NO$ 282.1858; found 282.1857.

***N*-Benzyl-2-(3-(3,5-dimethylphenyl)oxetan-3-yl)ethan-1-amine (65)**

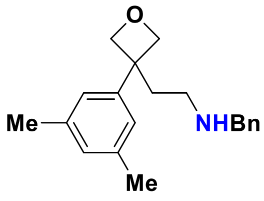

65

From 4 mmol of the oxetane alcohols according to Method F. Light yellow liquid; 62% yield; column chromatography (silica gel, PE/EA = 3:1→1:1 and then MeOH/DCM = 75:1→15:1). **^{1}H NMR** (400 MHz, $CDCl_3$) δ 7.38 – 7.32 (m, 2H), 7.29 (t, *J* = 5.9 Hz, 3H), 6.93 (s, 1H), 6.69 (s, 2H), 5.02 (d, *J* = 5.6 Hz, 2H), 4.73 (d, *J* = 5.6 Hz, 2H), 3.73 (s, 2H), 2.58 – 2.53 (m, 2H), 2.39 – 2.28 (m, 8H), 1.74 (s, 1H). **^{13}C NMR** (100 MHz, $CDCl_3$) δ 144.7, 140.2, 138.0, 128.4, 128.1, 128.1, 127.0, 123.5, 123.5, 82.2, 54.1, 46.5, 45.4, 41.1, 21.4. **HRMS** (ESI): calcd for $[M + H]^+$ $C_{20}H_{26}NO$ 296.2014; found 296.2014.

***N*-Benzyl-2-(3-(4-ethylphenyl)oxetan-3-yl)ethan-1-amine (67)**

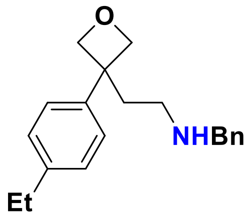

67

From 4 mmol of the oxetane alcohols according to Method F. Light yellow liquid; 65% yield; column chromatography (silica gel, PE/EA = 3:1→1:1 and then MeOH/DCM = 75:1→15:1). **^{1}H NMR** (400 MHz, $CDCl_3$) δ 7.38 – 7.30 (m, 2H), 7.30 – 7.23 (m, 3H), 7.21 (d, *J* = 7.6 Hz, 2H), 7.01 (d, *J* = 7.4 Hz, 2H), 5.02 (d, *J* = 5.3 Hz, 2H), 4.75 (d, *J* = 5.5 Hz, 2H), 3.72 (s, 2H), 2.69 (q, *J* = 7.5 Hz, 2H), 2.55 (t, *J* = 7.3 Hz, 2H), 2.34 (t, *J* = 7.3 Hz, 2H), 1.39 (s, 1H), 1.29 (t, *J* = 7.6 Hz, 3H). **^{13}C NMR** (100 MHz, $CDCl_3$) δ 142.4, 142.0, 140.2, 128.5, 128.2, 128.1, 127.1, 125.7, 82.3, 54.1, 46.4, 45.4, 41.1, 28.6. **HRMS** (ESI): calcd for $[M + H]^+$ $C_{20}H_{26}NO$ 296.2014; found 296.2010.

***N*-(2-(3-(4-Ethylphenyl)oxetan-3-yl)ethyl)prop-2-en-1-amine (69)**

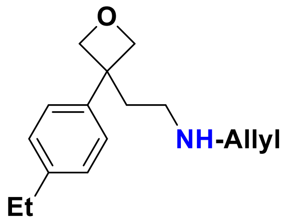

69

From 4 mmol of the oxetane alcohols according to Method F. Light yellow liquid; 38% yield; column chromatography (silica gel, PE/EA = 3:1→1:1 and then MeOH/DCM = 75:1→15:1). **^{1}H NMR** (400 MHz, $CDCl_3$) δ 7.29 (d, *J* = 7.9 Hz, 2H), 7.08 (d, *J* = 7.9 Hz, 2H), 5.94 (dq, *J* = 11.5, 6.0 Hz, 1H), 5.18 (dd, *J* = 22.5, 13.7 Hz, 2H), 5.08 (d, *J* = 5.6 Hz, 2H), 3.26 (d, *J* = 5.9 Hz, 2H), 2.75 (q, *J* = 7.6 Hz, 2H), 2.62 – 2.53 (m, 2H), 2.42 – 2.35 (m, 2H), 1.78 (s, 1H), 1.35 (t, *J* = 7.6 Hz, 3H). **^{13}C NMR** (100 MHz, $CDCl_3$) δ 142.3, 141.9, 136.5, 128.0, 125.6, 115.9, 82.2, 52.4, 46.3, 45.1, 41.1, 28.5, 15.6. **HRMS** (ESI): calcd for $[M + H]^+$ $C_{16}H_{24}NO$ 246.1858; found 246.1862.

***N*-Benzyl-2-(3-(4-methoxyphenyl)oxetan-3-yl)ethan-1-amine (71)**

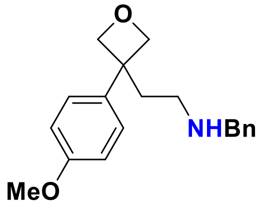

71

From 4 mmol of the oxetane alcohols according to Method F. Light yellow liquid; 58% yield; column chromatography (silica gel, PE/EA = 3:1→1:1 and then MeOH/DCM = 75:1→15:1). **^{1}H NMR** (400 MHz, $CDCl_3$) δ 7.38 – 7.31 (m, 2H), 7.28 (d, *J* = 6.7 Hz, 3H), 7.02 (d, *J* = 8.7 Hz, 2H), 6.91 (d, *J* = 8.7 Hz, 2H), 4.99 (d, *J* = 5.7 Hz, 2H), 4.74 (d, *J* = 5.6 Hz, 2H), 3.83 (s, 3H), 3.72 (s, 2H), 2.58 – 2.46 (m, 2H), 2.38 – 2.24 (m, 2H), 1.30 (s, 1H). **^{13}C NMR** (100 MHz, $CDCl_3$) δ 158.2, 140.5, 136.9, 128.5, 128.2, 127.1, 126.9, 114.1, 82.5, 55.4, 54.2, 46.2, 45.6, 41.3. **HRMS** (ESI): calcd for $[M + H]^+$ $C_{19}H_{24}NO_2$ 298.1807; found 298.1811.

N-Benzyl-2-(3-(4-(methylthio)phenyl)oxetan-3-yl)ethan-1-amine (73)

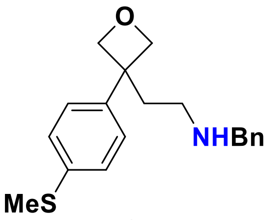

73

From 4 mmol of the oxetane alcohols according to Method F. Light yellow liquid; 63% yield; column chromatography (silica gel, PE/EA = 3:1→1:1 and then MeOH/DCM = 75:1→15:1). **^{1}H NMR** (400 MHz, $CDCl_3$) δ 7.36 – 7.31 (m, 2H), 7.28 – 7.22 (m, 5H), 7.00 (d, *J* = 8.2 Hz, 2H), 4.97 (d, *J* = 5.7 Hz, 2H), 4.73 (d, *J* = 5.7 Hz, 2H), 3.69 (s, 2H), 2.54 – 2.46 (m, 5H), 2.38 – 2.23 (m, 2H), 1.94 (s, 1H). **^{13}C NMR** (100 MHz, $CDCl_3$) δ 141.5, 140.0, 136.4, 128.5, 128.1, 127.1, 126.9, 126.3, 82.1, 54.0, 46.3, 45.2, 40.9, 16.0. **HRMS** (ESI): calcd for $[M + H]^+$ $C_{19}H_{24}NOS$ 314.1579; found 314.1582.

N-(2-(3-(4-(Methylthio)phenyl)oxetan-3-yl)ethyl)prop-2-en-1-amine (75)

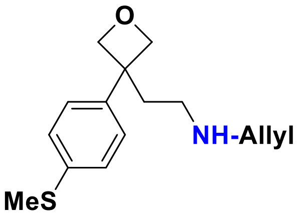

75

From 4 mmol of the oxetane alcohols according to Method F. Light yellow liquid; 42% yield; column chromatography (silica gel, PE/EA = 3:1→1:1 and then MeOH/DCM = 75:1→15:1).**^{1}H NMR** (400 MHz, $CDCl_3$) δ 7.28 – 7.22 (m, 2H), 7.00 (d, *J* = 8.3 Hz, 2H), 5.84 (m, 1H), 5.15 – 5.03 (m, 2H), 4.95 (d, *J* = 5.7 Hz, 2H), 4.70 (d, *J* = 5.7 Hz, 2H), 3.16 (d, *J* = 5.9, 1.2 Hz, 2H), 2.49 (s, 3H), 2.47 – 2.41 (m, 2H), 2.30 – 2.23 (m, 2H), 1.86 (s, 1H). **^{13}C NMR** (100 MHz, $CDCl_3$) δ 141.5, 136.5, 136.4, 126.9, 126.2, 116.0, 82.0, 52.4, 46.2, 45.1, 40.9, 16.0. **HRMS** (ESI): calcd for $[M + H]^+$ $C_{15}H_{12}NOS$ 264.1422; found 264.1426.

N-Benzyl-2-(3-(4-fluorophenyl)oxetan-3-yl)ethan-1-amine (77)

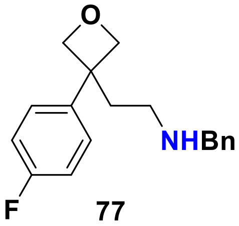

77

From 4 mmol of the oxetane alcohols according to Method F. Light yellow liquid; 54% yield; column chromatography (silica gel, PE/EA = 3:1→1:1 and then MeOH/DCM = 75:1→15:1). **^{1}H NMR** (400 MHz, $CDCl_3$) δ 7.39 – 7.33 (m, 4H), 7.29 (dd, *J* = 8.9, 7.4 Hz, 3H), 7.05 – 7.00 (m, 2H), 4.97 (d, *J* = 5.8 Hz, 2H), 4.77 (d, *J* = 5.8 Hz, 2H), 3.73 (s, 2H), 2.55 – 2.42 (m, 2H), 2.36 – 2.24 (m, 2H), 1.36 (s, 1H). **^{19}F NMR** (376 MHz, $CDCl_3$) -116.24 – -116.34 (m). **^{13}C NMR** (100 MHz, $CDCl_3$) δ 161.4 (d, *J* = 245.1 Hz), 140.51 (d, *J* = 3.3 Hz), 140.1, 128.5, 128.1, 127.4 (d, *J* = 7.9 Hz), 127.1, 115.4 (d, *J* = 21.3 Hz), 82.3, 54.1, 46.3, 45.3, 41.1. **HRMS** (ESI): calcd for $[M + H]^+$ $C_{18}H_{21}NOF$ 286.1607; found 286.1610.

N-Benzyl-2-(3-(4-chlorophenyl)oxetan-3-yl)ethan-1-amine (79)

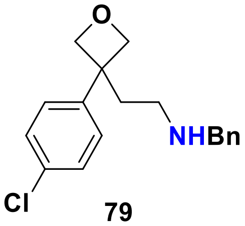

79

From 4 mmol of the oxetane alcohols according to Method F. Light yellow liquid; 44% yield; column chromatography (silica gel, PE/EA = 3:1→1:1 and then MeOH/DCM = 75:1→15:1). **^{1}H NMR** (400 MHz, $CDCl_3$) δ 7.39 – 7.31 (m, 2H), 7.28 (t, *J* = 7.2 Hz, 3H), 7.05 (d, *J* = 7.0 Hz, 4H), 4.98 (d, *J* = 5.7 Hz, 2H), 4.76 (d, *J* = 5.7 Hz, 2H), 3.72 (s, 2H), 2.63 – 2.44 (m, 2H), 2.37 – 2.24 (m, 2H), 1.33 (s, 1H).**^{13}C NMR** (100 MHz, $CDCl_3$) δ 143.3, 140.1, 132.3, 128.7, 128.5, 128.32, 127.3, 127.1, 82.1, 54.1, 46.4, 45.2, 40.9. **HRMS** (ESI): calcd for $[M + H]^+$ $C_{18}H_{21}NOCl$ 302.1312; found 302.1314.

***N*-Benzyl-2-(3-(4-bromophenyl)oxetan-3-yl)ethan-1-amine (81)**

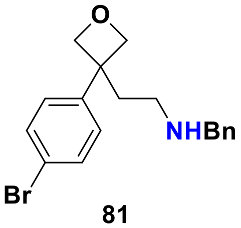

81

From 4 mmol of the oxetane alcohols according to Method F. Light yellow liquid; 46% yield; column chromatography (silica gel, PE/EA = 3:1→1:1 and then MeOH/DCM = 75:1→15:1). **^{1}H NMR** (400 MHz, $CDCl_3$) δ 7.49 (d, *J* = 8.4 Hz, 2H), 7.40 – 7.32 (m, 2H), 7.28 (t, *J* = 8.7 Hz, 3H), 6.97 (d, *J* = 8.4 Hz, 2H), 4.97 (d, *J* = 5.8 Hz, 2H), 4.76 (d, *J* = 5.7 Hz, 2H), 3.72 (s, 2H), 2.70 – 2.44 (m, 2H), 2.33 – 2.25 (m, 2H), 1.39 (s, 1H). **^{13}C NMR** (100 MHz, $CDCl_3$) δ 143.8, 140.1, 131.6, 128.5, 128.1, 127.6, 127.0, 120.3, 82.0, 54.1, 46.5, 45.2, 40.9. **HRMS** (ESI): calcd for $[M + H]^+$ $C_{18}H_{21}NOBr$ 346.0807; found 346.0811.

***N*-Benzyl-2-(3-(4-(trifluoromethyl)phenyl)oxetan-3-yl)ethan-1-amine (83)**

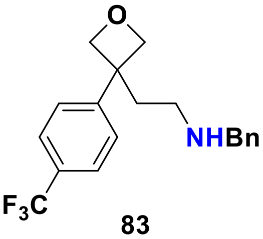

83

From 4 mmol of the oxetane alcohols according to Method F. Light yellow liquid; 53% yield; column chromatography (silica gel, PE/EA = 3:1→1:1 and then MeOH/DCM = 75:1→15:1). **^{1}H NMR** (400 MHz, $CDCl_3$) δ 7.63 (d, *J* = 8.0 Hz, 2H), 7.38 – 7.31 (m, 2H), 7.28 (dd, *J* = 11.6, 7.7 Hz, 3H), 7.21 (d, *J* = 8.0 Hz, 2H), 5.01 (d, *J* = 5.8 Hz, 2H), 4.81 (d, *J* = 5.8 Hz, 2H), 3.72 (s, 2H), 2.50 (t, *J* = 7.3 Hz, 2H), 2.35 (t, *J* = 7.3 Hz, 2H), 1.42 (s, 1H). **^{19}F NMR** (376 MHz, $CDCl_3$) δ -62.33 (s). **^{13}C NMR** (100 MHz, $CDCl_3$) δ 148.8, 134.0 128.5, 128.1, 127.1, 126.2, 125.5 (q, *J* = 3.8 Hz), 81.9, 54.0, 46.8, 45.1, 40.8. **HRMS** (ESI): calcd for $[M + H]^+$ $C_{19}H_{21}NOF_3$ 336.1575; found 336.1578.

***N*-(2-(3-(4-(Trifluoromethyl)phenyl)oxetan-3-yl)ethyl)prop-2-en-1-amine (85)**

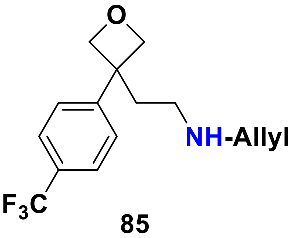

85

From 4 mmol of the oxetane alcohols according to Method F. Light yellow liquid; 39% yield; column chromatography (silica gel, PE/EA = 3:1→1:1 and then MeOH/DCM = 75:1→15:1). **^{1}H NMR** (400 MHz, $CDCl_3$) δ 7.56 (d, J = 8.1 Hz, 0.73H)/7.29 (t, J = 7.6 Hz, 1.27H), 7.15 (d, J = 7.9 Hz, 0.75H)/7.07 – 6.97 (m, 1.23H), 5.85 – 5.67 (m, 1H), 5.09 – 4.97 (m, 2H), 4.93 (d, J = 5.7 Hz, 1.23H), 4.90 (d, J = 5.9 Hz, 0.77H), 4.70 (d, J = 5.9 Hz, 0.75H)/4.66 (d, J = 5.7 Hz, 1.25H), 3.13 – 3.05 (m, 2H), 2.46 – 2.31 (m, 2H), 2.26 – 2.19 (m, 2H), 1.53 (s, 1H). **^{19}F NMR** (376 MHz, $CDCl_3$) δ -62.37 (s). **^{13}C NMR** (100 MHz, $CDCl_3$) δ 144.7, 136.6/136.6(2), 128.4, 126.3/126.2, 125.6, 125.41 (q, *J* = 3.7 Hz), 82.0/81.7, 52.3/52.3(2), 46.7/46.5, 45.0/44.9, 41.1/40.8. **HRMS** (ESI): calcd for $[M + H]^+$ $C_{15}H_{19}NOF_3$ 286.1419; found 286.1421.

***N*-Benzyl-2-(3-benzyloxetan-3-yl)ethan-1-amine (87)**

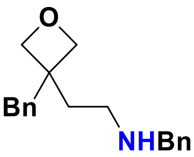

87

From 4 mmol of the oxetane alcohols according to Method F. Light yellow liquid; 75% yield; column chromatography (silica gel, PE/EA = 3:1→1:1 and then MeOH/DCM= 75:1→15:1). **^{1}H NMR** (400 MHz, $CDCl_3$) δ 7.41 – 7.27 (m, 8H), 7.11 (d, *J* = 6.8 Hz, 2H), 4.64 (d, *J* = 5.9 Hz, 2H), 4.48 (d, *J* = 5.9 Hz, 2H), 3.84 (s, 2H), 3.00 (s, 2H), 2.82 – 2.73 (m, 2H), 1.98 (s, 1H), 1.90 – 1.84 (m, 2H). **^{13}C NMR** (100 MHz, $CDCl_3$) δ 140.2, 138.0, 129.6, 128.7, 128.6, 128.3, 127.3, 126.7, 81.3, 54.2, 44.9, 42.8, 42.2, 35.9. **HRMS** (ESI): calcd for $[M + H]^+$ $C_{19}H_{24}NO$ 282.1858; found 282.1862.

***N*-Benzyl-3-(3-phenyloxetan-3-yl)propan-1-amine (89)**

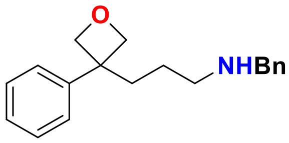

89

From 4 mmol of the oxetane alcohols according to Method F. Light yellow liquid; 65% yield; column chromatography (silica gel, PE/EA = 3:1→1:1 and then MeOH/DCM = 75:1→15:1). **^{1}H NMR** (400 MHz, $CDCl_3$) δ 7.41 – 7.26 (m, 8H), 7.10 – 6.98 (m, 2H), 5.02 (d, *J* = 5.6 Hz, 2H), 4.70 (d, *J* = 5.6 Hz, 2H), 3.76 (s, 2H), 2.63 (t, *J* = 7.2 Hz, 2H), 2.20 – 2.09 (m, 2H), 1.55 (s, 1H), 1.37 (m, 2H). **^{13}C NMR** (100 MHz, $CDCl_3$) δ 144.9, 140.3, 128.5, 128.4, 128.1, 127.0, 126.3, 125.8, 125.7, 81.9, 54.1, 49.5, 47.3, 39.0, 25.2. **HRMS** (ESI): calcd for $[M + H]^+$ $C_{19}H_{24}NO$ 282.1858; found 282.1862.

***N*-Benzyl-3-(3-benzyloxetan-3-yl)propan-1-amine (91)**

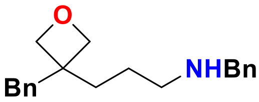

91

From 4 mmol of the oxetane alcohols according to Method F. Light yellow liquid; 45% yield; column chromatography (silica gel, PE/EA = 3:1→1:1 and then MeOH/DCM = 75:1→15:1). **^{1}H NMR** (400 MHz, $CDCl_3$) δ 7.42 – 7.26 (m, 8H), 7.18 – 7.10 (m, 2H), 4.64 (d, *J* = 5.8 Hz, 2H), 4.41 (d, *J* = 5.8 Hz, 2H), 3.82 (s, 2H), 3.02 (s, 2H), 2.68 (t, *J* = 6.8 Hz, 2H), 1.73 – 1.55 (m, 5H). **^{13}C NMR** (100 MHz, $CDCl_3$) δ 140.4, 138.0, 129.4, 128.5, 128.4, 128.1, 127.0, 126.5, 81.0, 54.0, 49.7, 43.2, 41.6, 33.2, 24.8. **HRMS** (ESI): calcd for $[M + H]^+$ $C_{20}H_{26}NO$ 296.2014; found 296.2015.

3.2 General procedure for the synthesis of chiral heterocycles using *Re*LEH mutants

3.2.1 General procedure for the synthesis of racemic products

Method H:

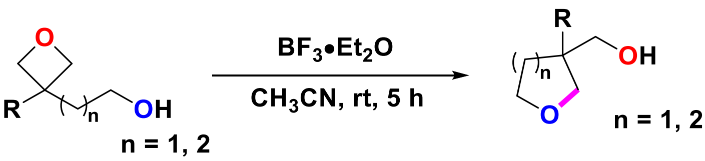

Method I:

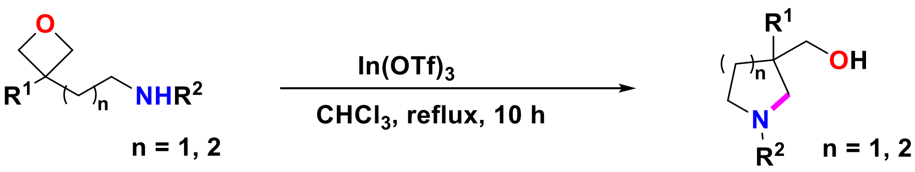

Method H: To a solution of oxetane alcohols (1.0 mmol) in CH_3CN (2 mL) was added $BF_3·Et_2O$ (10 mol%). The solution was stirred 5 h at rt and concentrated in vacuo. The residue was purified by flash chromatography to afford desired racemic products.

Method I: To a solution of oxetane amines (1.0 mmol) in $CHCl_3$ (2 mL) was added $In(OTf)_3$ (10 mol%). The solution was stirred 10 h for reflux and concentrated in vacuo. The residue was purified by flash chromatography to afford desired racemic products.

3.2.2 General procedure for Baldwin/anti-Baldwin and desymmetrization reactions

Baldwin/anti-Baldwin:

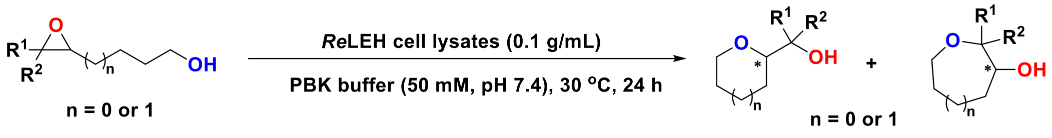

Desymmetrization:

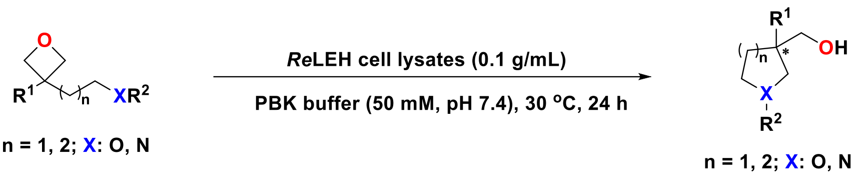

To a solution of 475 μL enzyme (50 mM PBK buffer, pH 7.4, 6 U DNase I and 1 mg/mL lysozyme for breaking the cell) was added substrates (100 mM) in 25 μL CH_3CN. The mixture was shaken at 1000 rpm for 24 h at 30 °C, then extracted with ethyl acetate for three times and concentrated in vacuo. The conversion rate and e.r. value for product were determined by gas chromatography (GC) and high performance liquid chromatography (HPLC).

3.2.3 General procedure for Baldwin and anti-Baldwin reactions catalyzed by *Re*LEH mutants

Baldwin/anti-Baldwin:

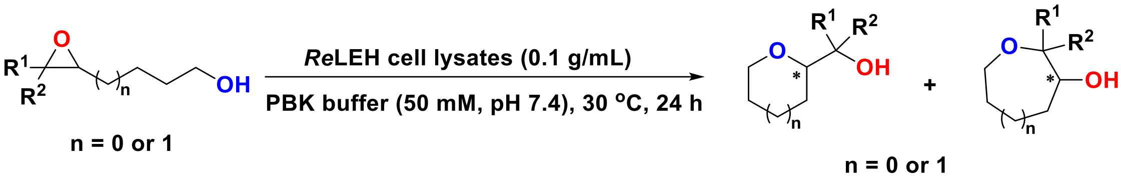

(*S*)-(Tetrahydro-2*H*-pyran-2-yl)methanol (2)

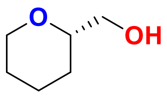

2

SZ612 (Y53F/N55A/I80F/L114V/I116V): Conversion = 60%; e.r = 90:10; SZ616 (Y53F/N55A/I116V): Conversion = 87%; e.r = 45:55; The enantioselectivity was determined by GC analysis using chiral column Hydrodex-*β*-TBDAc, 25 m x 0.25 mm ID as the follows condition: 110 °C, 5 °C/min, 135 °C; 20 °C/min, 220 °C hold 2 min. N_2: 1.5 bar. The spectral data were consistent with a previous literature report[2]. **^{1}H NMR** (400 MHz, $CDCl_3$) δ 4.02 (d, *J* = 11.5 Hz, 1H), 3.61 – 3.31 (m, 4H), 2.44 (s, 1H), 1.87 (d, *J* = 7.8 Hz, 1H), 1.66 – 1.43 (m, 4H), 1.34 (td, *J* = 14.0, 8.1 Hz, 1H). **^{13}C NMR** (101 MHz, CDCl3) δ 78.2, 68.3, 66.3, 27.4, 26.0, 23.0.

(*S*)-1-(Tetrahydro-2*H*-pyran-2-yl)ethan-1-ol (6)

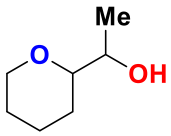

6

SZ612 (Y53F/N55A/I80F/L114V/I116V): Conversion = 69%; d.r = 90:10; SZ616 (Y53F/N55A/I116V): Conversion = 99%; d.r = 86:14; The enantioselectivity was determined by GC analysis using chiral column Hydrodex-*β*-TBDAc, 25 m x 0.25 mm ID as the follows condition: 80 °C, 5 °C/min, 110 °C, hold 2 min, 5 °C/min, 135 °C, hold 2 min, 20 °C/min, 220 °C hold 2 min. N_2: 1.5 bar. The spectral data were consistent with a previous literature report[2]. **^{1}H NMR** (400 MHz, $CDCl_3$) δ 4.08 – 3.94 (m, 1H), 3.84 – 3.73/ 3.64 – 3.54 (m, 1H), 3.58 (p, *J* = 6.5 Hz, 1H), 3.52 – 3.38/ 3.28 – 3.21 (m, 1H), 3.09 – 3.01 (m, 1H), 2.93 (s, 1H), 1.98 – 1.81 (m, 1H), 1.65 – 1.43 (m, 4H), 1.33 – 1.22 (m, 1H), 1.13 (d, *J* = 6.3 Hz, 3H). **^{13}C NMR** (101 MHz, $CDCl_3$) δ 82.4, 81.1*, 70.6, 69.6*, 68.7*, 68.4, 27.5, 26.2*, 25.9, 25.1*, 23.1*, 23.0, 18.1, 17.7*. (Signal for mixture of stereoisomers denoted with asterisk).

(*S*)-2-(Tetrahydro-2*H*-pyran-2-yl)propan-2-ol (8)

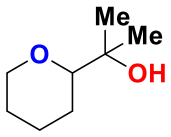

8

SZ612 (Y53F/N55A/I80F/L114V/I116V): Conversion = 76%; e.r = 73:27; SZ616 (Y53F/N55A/I116V): Conversion = 96%; e.r = 52:48; The enantioselectivity was determined by GC analysis using chiral column LIPODEX-E, 25 m x 0.25 mm ID as the follows condition: 80 °C, hold 1 min. 3 °C/min, 100 °C, hold 2 min, 20 °C/min, 175 °C hold 2 min. N_2: 1.5 bar. The spectral data were consistent with a previous literature report[2]. **^{1}H NMR** (400 MHz, $CDCl_3$) δ 4.24 – 3.95 (m, 1H), 3.44 (t, *J* = 11.1 Hz, 1H), 3.08 (d, *J* = 11.3 Hz, 1H), 2.66 (s, 1H), 1.88 (d, *J* = 10.0 Hz, 1H), 1.67 – 1.41 (m, 4H), 1.38 – 1.26 (m, 1H), 1.15 (d, *J* = 10.4 Hz, 6H). **^{13}C NMR** (101 MHz, $CDCl_3$) δ 84.5, 71.9, 68.8, 26.1, 26.0, 24.0, 23.5.

3.2.4 General procedure for scaling-up enantioselective intramolecular openings of oxetanes.

Desymmetrization:

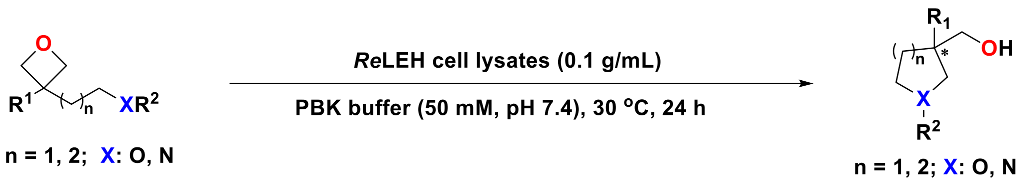

To a solution of mutants SZ616 or SZ611 (0.2 g/mL wet cell) in 19 mL PBK buffer (50 mM, pH 7.4), Lysozyme (1 mg/mL), DNase I (6 U/mL), was added substrates (1M) in 1 mL CH_3CN. The mixture was shaken at 1000 rpm for 24 h at 30 °C. After 24 hours of reaction, the reaction system was added 2 g wet cells and stirred for another 24 hours. The suspension was heated for 5 minutes, then centrifuged for 10 minutes. The mixture was passed through a plug of celite eleuting with ethyl acetate. the resulting mixture extracted with ethyl acetate for several times. The organic layers were combined, dried with Na_2SO_4 and concentrated under reduced pressure, followed by flash column chromatography.

3.2.5 General procedure for asymmetric desymmetrization of oxetanes using *Re*LEH mutants to synthesize chiral tetrahydrofurans and tetrahydropyrans

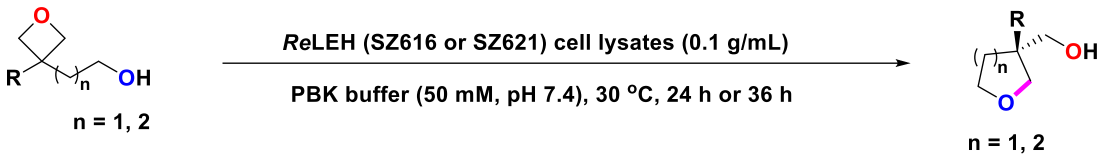

(*S*)-(3-Phenyltetrahydrofuran-3-yl)methanol (10)

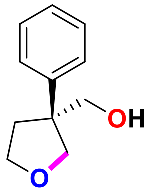

10

Obtained as a colorless oil after column chromatography (PE/EA = 4:1, R_f = 0.32). SZ616 (Y53F/N55A/I116V): Conversion = 96%; yield = 78%; e.r = 72:28; SZ621 (Y53F/N55A/I80F/I116V): Conversion = 68%; e.r = 87:13; The enantioselectivity was determined by HPLC using a Daicel CHIRALCEL AD-H column (25 cm × 0.46 cm ID), [hexane/iso-propanol = 98:2, λ = 220 nm, 1.0 mL/min flow rate] t_S = 22.8 min (major) and t_R = 24.0 min (minor); Absolute configuration assigned based on correlation in a previous literature report[4]. **^{1}H NMR** (400 MHz, $CDCl_3$) δ 7.38 (t, *J* = 7.5 Hz, 2H), 7.29 (t, *J* = 7.3 Hz, 1H), 7.22 (d, *J* = 7.3 Hz, 2H), 4.25 (d, *J* = 8.6 Hz, 1H), 4.05 (dd, *J* = 15.9, 7.9 Hz, 1H), 4.04 – 3.89 (m, 1H), 3.85 (d, *J* = 8.6 Hz, 1H), 3.74 – 3.52 (m, 2H), 2.45 – 2.29 (m, 1H), 2.28 – 2.12 (m, 1H), 1.92 (s, 1H). **^{13}C NMR** (100 MHz, $CDCl_3$) δ143.11, 128.66, 127.28, 126.92, 74.26, 69.34, 67.59, 53.47, 33.90. **HRMS** (ESI): calcd for $[M + H]^+$ $C_{11}H_{15}O_2$ 179.1072; found 179.1069.

(*S*)-(3-(4-Tolyl)tetrahydrofuran-3-yl)methanol (12)

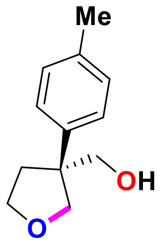

12

Obtained as a colorless oil after column chromatography (PE/EA = 4:1, R_f = 0.25). SZ616 (Y53F/N55A/I116V): Conversion = 98%; yield = 82%; e.r = 70:30; SZ621 (Y53F/N55A/I80F/I116V): Conversion = 79%; e.r = 82:18; The enantioselectivity was determined by HPLC using a Daicel CHIRALCEL AD-H column (25 cm × 0.46 cm ID), [hexane/iso-propanol = 97:3, λ = 220 nm, 1.0 mL/min flow rate] t_S = 30.8 min (major) and t_R = 32.3 min (minor); **^{1}H NMR** (400 MHz, $CDCl_3$) δ 7.19 (d, *J* = 7.9 Hz, 2H), 7.11 (d, *J* = 8.1 Hz, 2H), 4.18 (d, *J* = 8.5 Hz, 1H), 4.01 (q, *J* = 7.8 Hz, 1H), 3.91 (dd, *J* = 13.0, 8.2 Hz, 1H), 3.80 (d, *J* = 8.5 Hz, 1H), 3.62 (s, 2H), 2.44 (s, 1H), 2.37 (s, 3H), 2.34 – 2.24 (m, 1H), 2.17 (dd, *J* = 20.2, 8.2 Hz, 1H). **^{13}C NMR** (100 MHz, $CDCl_3$) δ 134.0, 136.6, 129.4, 127.2, 74.4, 69.4, 67.6, 53.1, 34.0, 21.0. **HRMS** (ESI): calcd for $[M + H]^+$ $C_{12}H_{17}O_2$ 193.1229; found 193.1226.

(*S*)-(3-(3-Tolyl)tetrahydrofuran-3-yl)methanol (14)

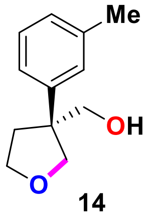

14

Obtained as a colorless oil after column chromatography (PE/EA = 4:1, R_f = 0.35). SZ616 (Y53F/N55A/I116V): Conversion = 98%; yield = 88%; e.r = 60:40; SZ621 (Y53F/N55A/I80F/I116V): Conversion = 91%; e.r = 74:26; The enantioselectivity was determined by HPLC using a Daicel CHIRALCEL AD-H column (25 cm × 0.46 cm ID), [hexane/iso-propanol = 95:5, λ = 220 nm, 1.0 mL/min flow rate] t_S = 13.4 min (major) and t_R = 15.2 min (minor); **^{1}H NMR** (400 MHz, $CDCl_3$) δ 7.26 (t, *J* = 7.5 Hz, 1H), 7.10 (d, *J* = 7.5 Hz, 1H), 7.02 (d, *J* = 9.3 Hz, 2H), 4.20 (d, *J* = 8.5 Hz, 1H), 4.01 (t, *J* = 7.6 Hz, 1H), 3.93 (dd, *J* = 9.0, 4.9 Hz, 1H), 3.82 (d, *J* = 8.5 Hz, 1H), 2.40 (s, 3H), 2.36 – 2.27 (m, 1H), 2.24 – 2.13 (m, 1H). **^{13}C NMR** (100 MHz, $CDCl_3$) δ 138.1, 128.4, 128.1, 127.5, 124.4, 74.2, 69.0, 67.5, 53.3, 33.9, 21.6. **HRMS** (ESI): calcd for $[M + H]^+$ $C_{12}H_{17}O_2$ 193.1229; found 193.1228.

(*S*)-(3-(3,5-Dimethylphenyl)tetrahydrofuran-3-yl)methanol (16)

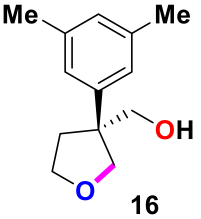

16

Obtained as a colorless oil after column chromatography (PE/EA = 4:1, R_f = 0.34). SZ616 (Y53F/N55A/I116V): Conversion = 83%; yield = 62%; e.r = 78:22; SZ621 (Y53F/N55A/I80F/I116V): Conversion = 37%; e.r = 84:16; The enantioselectivity was determined by HPLC using a Daicel CHIRALCEL AD-H column (25 cm × 0.46 cm ID), [hexane/iso-propanol = 98:2, λ = 220 nm, 1.0 mL/min flow rate] t_S = 17.9 min (major) and t_R = 19.7 min (minor); **^{1}H NMR** (400 MHz, $CDCl_3$) δ 6.95 (s, 1H), 6.84 (s, 2H), 4.24 (d, *J* = 8.5 Hz, 1H), 4.06 (q, *J* = 7.9 Hz, 1H), 3.95 (td, *J* = 8.9, 5.0 Hz, 1H), 3.84 (d, *J* = 8.5 Hz, 1H), 3.67 (s, 2H), 2.37 (s, 6H), 2.36 – 2.28 (m, 1H), 2.27 – 2.10 (m, 1H), 1.80 (s, 1H). **^{13}C NMR** (100 MHz, $CDCl_3$) δ 143.0, 143.0, 138.1, 128.6, 125.1, 74.3, 69.3, 67.5, 53.3, 33.9, 21.5. **HRMS** (ESI): calcd for $[M + H]^+$ $C_{13}H_{19}O_2$ 207.1385; found 207.1386.

(*S*)-(3-(4-Ethylphenyl)tetrahydrofuran-3-yl)methanol (18)

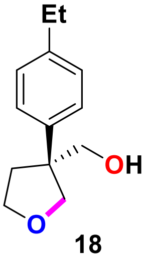

18

Obtained as a colorless oil after column chromatography (PE/EA = 4:1, R_f = 0.37). SZ616 (Y53F/N55A/I116V): Conversion = 98%; yield = 79%; e.r = 76:24; SZ621 (Y53F/N55A/I80F/I116V): Conversion = 78%; e.r = 90:10; The enantioselectivity was determined by HPLC using a Daicel CHIRALCEL IC column (25 cm × 0.46 cm ID), [hexane/ethanol = 97:3, λ = 220 nm, 1.0 mL/min flow rate] t_S = 14.4 min (major) and t_R = 12.2 min (minor) of the benzoylated product; **^{1}H NMR** (400 MHz, $CDCl_3$) δ 7.22 (d, *J* = 8.2 Hz, 2H), 7.15 (d, *J* = 8.2 Hz, 2H), 4.26 (d, *J* = 8.5 Hz, 1H), 4.07 (dd, *J* = 15.9, 7.9 Hz, 1H), 3.95 (td, *J* = 8.9, 5.1 Hz, 1H), 3.85 (d, *J* = 8.6 Hz, 1H), 3.69 (s, 2H), 2.67 (q, *J* = 7.6 Hz, 2H), 2.35 (ddd, *J* = 12.8, 7.8, 5.1 Hz, 1H), 2.21 (ddd, *J* = 12.4, 9.2, 7.3 Hz, 1H), 1.58 (s, 1H), 1.27 (t, *J* = 7.6 Hz, 3H). **^{13}C NMR** (100 MHz, $CDCl_3$) δ 142.8, 140.3, 128.1, 127.2, 74.4, 69.3, 67.6, 53.1, 34.0, 28.4, 15.6. **HRMS** (ESI): calcd for $[M + H]^+$ $C_{13}H_{19}O_2$ 207.1385; found 207.1382.

(*S*)-(3-(4-Methoxyphenyl)tetrahydrofuran-3-yl)methanol (20)

Obtained as a colorless oil after column chromatography (PE/EA = 4:1, R_f = 0.25).

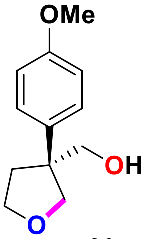

20

SZ616 (Y53F/N55A/I116V): Conversion = 96%; yield = 78%; e.r = 79:21; SZ621 (Y53F/N55A/I80F/I116V): Conversion = 90%; e.r = 89:11; The enantioselectivity was determined by HPLC using a Daicel CHIRALCEL AD-H column (25 cm × 0.46 cm ID), [hexane/iso-propanol = 98:2, λ = 220 nm, 1.0 mL/min flow rate] t_S = 47.0min (major) and t_R = 51.0 min (minor). **^{1}H NMR** (400 MHz, $CDCl_3$) δ 7.15 (d, *J* = 8.7 Hz, 2H), 6.92 (d, *J* = 8.7 Hz, 2H), 4.22 (d, *J* = 8.5 Hz, 1H), 4.10 – 4.00 (m, 1H), 3.94 (td, *J* = 8.9, 5.0 Hz, 1H), 3.83 (s, 3H), 3.82 (d, *J* = 7.7 Hz, 1H), 3.69 – 3.61 (m, 2H), 2.32 (ddd, *J* = 12.6, 7.8, 5.1 Hz, 1H), 2.24 – 2.11 (m, 1H), 1.82 (s, 1H). **^{13}C NMR** (100 MHz, $CDCl_3$) δ 158.4, 135.2, 128.3, 114.0, 74.4, 69.1, 67.6, 55.3, 52.7, 34.1. **HRMS** (ESI): calcd for $[M + H]^+$ $C_{12}H_{17}O_3$ 209.1178; found 209.1178.

(*S*)-(3-(4-(Methylthio)phenyl)tetrahydrofuran-3-yl)methanol (22)

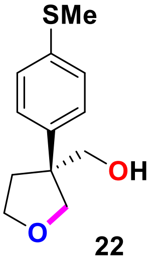

22

Obtained as a colorless oil after column chromatography (PE/EA = 4:1, R_f = 0.27). SZ616 (Y53F/N55A/I116V): Conversion = 97%; yield = 77%; e.r = 79:21; SZ621 (Y53F/N55A/I80F/I116V): Conversion = 98%; e.r = 89:11; The enantioselectivity was determined by HPLC using a Daicel CHIRALCEL IC column (25 cm × 0.46 cm ID), [hexane/ethanol = 97:3, λ = 220 nm, 1.0 mL/min flow rate] t_S = 23.0 min (major) and t_R = 21.0 min (minor) of the benzoylated product. **^{1}H NMR** (400 MHz, $CDCl_3$) δ 7.27 (d, *J*

= 8.3 Hz, 1H), 7.15 (d, *J* = 8.4 Hz, 1H), 4.22 (d, *J* = 8.6 Hz, 1H), 4.05 (q, *J* = 7.9 Hz, 1H), 3.94 (td, *J* = 8.9, 5.1 Hz, 1H), 3.83 (d, *J* = 8.6 Hz, 1H), 3.67 (s, 1H), 2.51 (s, 1H), 2.33 (ddd, *J* = 12.7, 7.8, 5.1 Hz, 1H), 2.24 – 2.06 (m, 1H), 1.67 (s, 1H). **^{13}C NMR** (100 MHz, $CDCl_3$) δ 140.0, 140.0, 137.0, 137.0, 127.8, 126.9, 74.3, 69.1, 67.6, 53.1, 34.0, 15.9. **HRMS** (ESI): calcd for $[M + H]^+$ $C_{12}H_{17}O_2S$ 225.0949; found 225.0947.

(*S*)-(3-(Naphthalen-2-yl)tetrahydrofuran-3-yl)methanol (24)

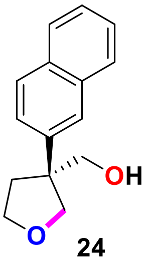

24

Obtained as a colorless oil after column chromatography (PE/EA = 4:1, R_f = 0.36). SZ616 (Y53F/N55A/I116V): Conversion = 92%; yield = 86%; e.r =74:26; SZ621 (Y53F/N55A/I80F/I116V): Conversion = 54%; e.r =91:9; The enantioselectivity was determined by HPLC using a Daicel CHIRALCEL IC column (25 cm × 0.46 cm ID), [hexane : EtOH = 97:3, λ = 220 nm, 1.0 mL/min flow rate] t_S = 19.246 min (major) and t_R = 16.870 min (minor) of the benzoylated product. **^{1}H NMR** (400 MHz, $CDCl_3$) δ 7.87 (dd, *J* = 8.2, 2.8 Hz, 3H), 7.67 (s, 1H), 7.58 – 7.47 (m, 2H), 7.36 (d, *J* = 8.5 Hz, 1H), 4.33 (d, *J* = 8.6 Hz, 1H), 4.10 (dd, *J* = 15.9, 7.9 Hz, 1H), 4.03 – 3.94 (m, 2H), 3.77 (s, 2H), 2.50 – 2.39 (m, 1H), 2.36 – 2.26 (m, 1H), 1.82 (s, 1H). **^{13}C NMR** (100 MHz, $CDCl_3$) δ 140.4, 133.2, 132.3, 128.5, 128.5, 127.9, 127.6, 126.4, 126.0, 125.9, 125.5, 74.3, 69.1, 69.1, 67.7, 53.6, 34.1. **HRMS** (ESI): calcd for $[M + H]^+$ $C_{15}H_{17}O_2$ 229.1229; found 229.1225.

(*S*)-(3-(4-Fluorophenyl)tetrahydrofuran-3-yl)methanol (26)

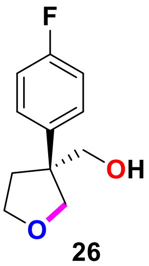

26

Obtained as a colorless oil after column chromatography (PE/EA = 4:1, Rf = 0.30). SZ616 (Y53F/N55A/I116V): Conversion = 96%; yield = 72%; e.r = 73:27; SZ621 (Y53F/N55A/I80F/I116V): Conversion = 90%; e.r = 86:14; The enantioselectivity was determined by HPLC using a Daicel CHIRALCEL IC column (25 cm × 0.46 cm ID), [hexane/iso-propanol = 97:3, λ = 220 nm, 1.0 mL/min flow rate] t_S = 16.5 min (major) and t_R = 17.9 min (minor). **^{1}H NMR** (400 MHz, $CDCl_3$) δ 7.25 – 7.16 (m, 2H), 7.08 (t, *J* = 8.6 Hz, 2H), 4.25 (d, *J* = 8.6 Hz, 1H), 4.07 (q, *J* = 7.9 Hz, 1H), 3.98 (dd, *J* = 8.9, 5.0 Hz, 1H), 3.83 (d, *J* = 8.5 Hz, 1H), 3.74 – 3.64 (m, 2H), 2.46 – 2.28 (m, 1H), 2.26 – 2.13 (m, 1H), 1.74 (s, 1H). **^{19}F NMR** (100 MHz, $CDCl_3$) -115.54 – -115.85 (m). **^{13}C NMR** (100 MHz, $CDCl_3$) δ 161.6 (d, *J* = 245.6 Hz), 139.0 (d, *J* = 3.3 Hz), 128.8 (d, *J* = 7.9 Hz), 115.4 (d, *J* = 21.1 Hz), 74.3, 68.9 (d, *J* = 0.6 Hz), 67.6, 52.9, 34.2. **HRMS** (ESI): calcd for $[M + H]^+$ $C_{11}H_{14}O_2F$ 197.0978; found 197.0979.

(*S*)-(3-(4-Chlorophenyl)tetrahydrofuran-3-yl)methanol (28)

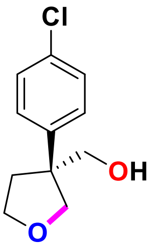

28

Obtained as a colorless oil after column chromatography (PE/EA = 4:1, Rf = 0.35). SZ616 (Y53F/N55A/I116V): Conversion = 94%; yield = 77%; e.r = 73:27; SZ621 (Y53F/N55A/I80F/I116V): Conversion = 68%; e.r = 88:12; The enantioselectivity was determined by HPLC using a Daicel CHIRALCEL IC column (25 cm × 0.46 cm ID), [hexane/iso-propanol = 97:3, λ = 220 nm, 1.0 mL/min flow rate] t_S = 16.2 min (major) and t_R = 18.0 min (minor). **^{1}H NMR** (400 MHz, $CDCl_3$) δ 7.32 (d, *J* = 7.3 Hz, 2H), 7.13 (d, *J* = 7.3 Hz, 2H), 4.19 – 4.08 (m, 1H), 4.01 – 3.93 (m, 1H), 3.92 – 3.84 (m, 1H), 3.79 – 3.70 (m, 1H), 3.58 (s, 2H), 2.53 (s, 1H), 2.30 – 2.18 (m, 1H), 2.17 – 2.03 (m, 1H). **^{13}C NMR** (100 MHz, $CDCl_3$) δ142.0, 132.6, 128.7, 128.6, 74.0, 68.5, 67.5, 53.0, 34.0. **HRMS** (ESI): calcd for $[M + H]^+$ $C_{11}H_{14}O_2Cl$ 213.0682; found 213.0685.

(*S*)-(3-(4-Bromophenyl)tetrahydrofuran-3-yl)methanol (30)

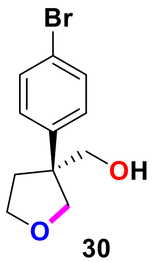

30

Obtained as a colorless oil after column chromatography (PE/EA = 4:1, R_f = 0.34). SZ616 (Y53F/N55A/I116V): Conversion = 84%; yield = 75%; e.r = 73:27; SZ621 (Y53F/N55A/I80F/I116V): Conversion = 59%; e.r = 89:11; The enantioselectivity was determined by HPLC using a Daicel CHIRALCEL IC column (25 cm × 0.46 cm ID), [hexane/iso-propanol = 97:3, λ = 220 nm, 1.0 mL/min flow rate] t_S = 17.1 min (major) and t_R = 19.3 min (minor). **^{1}H NMR** (400 MHz, $CDCl_3$) δ 7.51 (d, *J* = 8.3 Hz, 2H), 7.12 (d, *J* = 8.4 Hz, 2H), 4.22 (d, *J* = 8.6 Hz, 1H), 4.05 (q, *J* = 7.9 Hz, 1H), 4.00 – 3.91 (m, 1H), 3.81 (d, *J* = 8.6 Hz, 1H), 3.68 (s, 2H), 2.48 – 2.24 (m, 1H), 2.22 – 2.10 (m, 1H), 1.83 (s, 1H). **^{13}C NMR** (100 MHz, $CDCl_3$) δ 142.5, 131.7, 129.2, 120.9, 74.1, 68.8, 67.6, 53.2, 34.1. **HRMS** (ESI): calcd for $[M + H]^+$ $C_{11}H_{14}O_2Br$ 257.0177; found 257.0173.

(*S*)-(3-(4-(Trifluoromethyl)phenyl)tetrahydrofuran-3-yl)methanol (32)

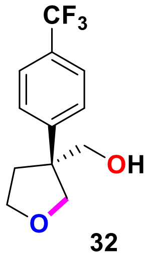

32

Obtained as a colorless oil after column chromatography (PE/EA = 4:1, R_f = 0.25). SZ616 (Y53F/N55A/I116V): Conversion = 87%; yield = 62%; e.r = 71:29; SZ621 (Y53F/N55A/I80F/I116V): Conversion = 53%; e.r = 88:12; The enantioselectivity was determined by HPLC using a Daicel CHIRALCEL IC column (25 cm × 0.46 cm ID), [hexane/iso-propanol = 93:7, λ = 220 nm, 1.0 mL/min flow rate] t_S = 13.9 min (major) and t_R = 13.2 min (minor). **^{1}H NMR** (400 MHz, $CDCl_3$) δ 7.61 (d, *J* = 8.1 Hz, 2H), 7.32 (d, *J* = 8.0 Hz, 2H), 4.18 (d, *J* = 8.6 Hz, 1H), 3.98 (d, *J* = 7.7 Hz, 1H), 3.89 (d, *J* = 4.9 Hz, 1H), 3.78 (d, *J* = 8.6 Hz, 1H), 3.62 (s, 2H), 2.71 (s, 1H), 2.34 – 2.24 (m, 1H), 2.20 – 2.07 (m, 1H). **^{19}F NMR** (100 MHz, $CDCl_3$) -62.43 (s). **^{13}C NMR** (100 MHz, $CDCl_3$) δ 147.7 (d, *J* = 1.3 Hz), 129.0 (q, *J* = 32.5 Hz), 125.3 (q, *J* = 3.8 Hz), 124.2 (q, *J* = 271.9 Hz), 73.8 , 68.3, 67.4, 53.4, 34.0. **HRMS** (ESI): calcd for $[M + H]^+$ $C_{12}H_{14}O_2F_3$ 247.0946; found 247.0944.

(*R*)-(3-Benzyltetrahydrofuran-3-yl)methanol (34)

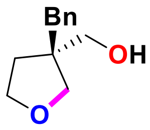

34

Obtained as a colorless oil after column chromatography (PE/EA = 4:1, R_f = 0.26). SZ616 (Y53F/N55A/I116V): Conversion = 97%; yield = 69%; e.r = 96: 4; SZ621 (Y53F/N55A/I80F/I116V): Conversion = 84%; e.r =95: 5; The enantioselectivity was determined by HPLC using a Daicel CHIRALCEL IC column (25 cm × 0.46 cm ID), [hexane/ethanol = 97:3, λ = 220 nm, 1.0 mL/min flow rate] t_S = 11.7 min (major) and t_R = 13.3 min (minor) of the benzoylated product. **^{1}H NMR** (400 MHz, $CDCl_3$) δ 7.34 (t, *J* = 7.1 Hz, 2H), 7.27 (t, *J* = 8.5 Hz, 3H), 4.02 – 3.81 (m, 2H), 3.73 (d, *J* = 8.8 Hz, 1H), 3.64 (d, *J* = 8.8 Hz, 1H), 3.50 (s, 2H), 2.86 (dd, *J* = 35.4, 13.4 Hz, 2H), 1.97 (s, 1H), 1.92 – 1.81 (m, 1H), 1.80 – 1.70 (m, 1H). **^{13}C NMR** (100 MHz, $CDCl_3$) δ 138.4, 130.1, 128.3, 126.4, 75.0, 67.7, 65.7, 49.1, 39.9, 33.8. **HRMS** (ESI): calcd for $[M + H]^+$ $C_{12}H_{17}O_2$ 193.1229; found 193.1231.

(*R*)-(3-Ethyltetrahydrofuran-3-yl)methanol (36)

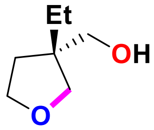

36

Obtained as a colorless oil after column chromatography (PE/EA = 4:1, R_f = 0.32). SZ616 (Y53F/N55A/I116V): Conversion = 72%; yield = 53%; e.r = 87:13; SZ621 (Y53F/N55A/I80F/I116V): Conversion = 30%; e.r =92:8; The enantioselectivity was determined by a chiral GC analysis (CP7503, 25 m x 0.25 mm ID) as the follows condition: 90 °C, hold 2 min ; 3 °C/min, 140 °C; hold 3 min; 20 °C/min, 200 °C hold 2

min. N_2: 1.5 bar, t_S = 21.5 min (major) and t_R = 21.8 min (minor). **^{1}H NMR** (400 MHz, $CDCl_3$) δ 3.99 – 3.78 (m, 2H), 3.73 (d, *J* = 8.8 Hz, 1H), 3.61 – 3.49 (m, 2H), 3.44 (d, *J* = 8.8 Hz, 1H), 2.45 (s, 1H), 1.84 – 1.64 (m, 2H), 1.66 – 1.40 (m, 2H), 0.93 (t, *J* = 7.5 Hz, 3H). **^{13}C NMR** (100 MHz, $CDCl_3$) δ 75.3, 68.0, 66.1, 48.2, 33.9, 26.9, 9.2. **HRMS** (ESI): calcd for $[M + H]^+$ $C_7H_{15}O_2$ 131.1072; found 131.1073.

(*R*)-(3-Methyltetrahydrofuran-3-yl)methanol (38)

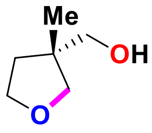

38

Obtained as a colorless oil after column chromatography (PE/EA = 4:1, R_f = 0.35). SZ616 (Y53F/N55A/I116V): Conversion = 37%; e.r = 76 : 24; The enantioselectivity was determined by a chiral GC analysis (Hydrodex-*β*-TBDAc, 25 m x 0.25 mm ID, 25 m x 0.25 mm ID) as the follows condition: 110 °C, 5 °C/min, 135 °C; hold 2 min; 20 °C/min, 200 °C hold 4 min. N_2: 1.5 bar, t_S = 5.6 min (major) and t_R = 5.8 min (minor). **^{1}H NMR** (400 MHz, $CDCl_3$) δ 3.96 – 3.83 (m, 2H), 3.75 (d, *J* = 8.6 Hz, 1H), 3.53 (s, 2H), 3.41 (d, *J* = 8.6 Hz, 1H), 1.95 (s, 1H), 1.89 (ddd, *J* = 12.4, 8.1, 6.8 Hz, 1H), 1.65 (ddd, *J* = 12.5, 8.1, 5.9 Hz, 1H), 1.16 (s, 3H). **^{13}C NMR** (100 MHz, $CDCl_3$) δ 76.6, 69.2, 68.0, 44.7, 36.0, 21.4. **HRMS** (ESI): calcd for $[M + H]^+$ $C_6H_{13}O_2$ 117.0921; found 117.0916.

(*R*)-(Tetrahydrofuran-3-yl)methanol (40)

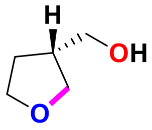

40

Obtained as a colorless oil after column chromatography (PE/EA = 4:1, Rf = 0.32). SZ616 (Y53F/N55A/I116V): Conversion = 20%; e.r = 60:40; The enantioselectivity was determined by a chiral GC analysis (Hydrodex-*β*-TBDAc, 25 m x 0.25 mm ID, 25 m x 0.25 mm ID) as the follows condition: 110 °C, 5 °C/min, 135 °C, hold 2 min; 20 °C/min, 200 °C hold 4 min. N_2: 1.5 bar, t_S = 5.6 min (major) and t_R = 5.8 min (minor) t_S = 24.5 min (major) and $t_{R =}$ 24.9 min (minor). This compound is directly commercially available.

(*S*)-(3-Phenyltetrahydro-2H-pyran-3-yl)methanol (42)

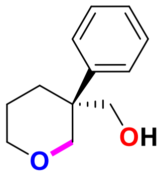

42

Obtained as a colorless oil after column chromatography (PE/EA = 4:1, R_f = 0.32). SZ616 (Y53F/N55A/I116V): Conversion = 20%; e.r = 89:11; The enantioselectivity was determined HPLC using a Daicel CHIRALCEL AD-H column (25 cm × 0.46 cm ID), [hexane: isopropanol = 98.5:1.5, λ = 220 nm, 1.0 mL/min flow rate] t_S = 39.0 min (major) and $t_{R=}$ 37.0 min (minor). **^{1}H NMR** (400 MHz, $CDCl_3$) δ 7.45 – 7.37 (m, 4H), 7.31 – 7.22 (m, 1H), 4.04 (d, *J* = 11.7 Hz, 1H), 3.93 (d, *J* = 11.7 Hz, 1H), 3.85 – 3.74 (m, 3H), 3.64 (ddd, *J* = 11.1, 7.4, 3.6 Hz, 1H), 2.11 – 1.90 (m, 2H), 1.78 – 1.67 (m, 1H), 1.63 – 1.47 (m, 2H). **^{13}C NMR** (100 MHz, $CDCl_3$) δ 142.7, 128.7, 126.9, 126.7, 72.8, 68.6, 68.4, 42.9, 30.5, 22.4. **HRMS** (ESI): calcd for $[M + H]^+$ $C_{12}H_{17}O_2$ 193.1232; found 193.1229.

(*S*)-(3-Benzyltetrahydro-2H-pyran-3-yl)methanol (44)

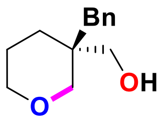

44

Obtained as a colorless oil after column chromatography (PE/EA = 4:1, Rf = 0.35). SZ616 (Y53F/N55A/I116V): Conversion = 56%; e.r = 77:23; The enantioselectivity was determined by HPLC using a Daicel CHIRALCEL AD-H column (25 cm × 0.46 cm ID), [hexane/iso-propanol = 90:10, λ = 220 nm, 1.0 mL/min flow rate] t_S = 47.0 min (major) and t_R = 51.0 min (minor). **^{1}H NMR** (400 MHz, $CDCl_3$) δ 7.32 (t, *J* = 7.1 Hz, 2H), 7.28 – 7.21 (m, 3H), 3.84 – 3.72 (m, 1H), 3.69 – 3.54 (m, 3H), 3.44 (t, *J* = 9.8 Hz, 2H), 2.78 (d, *J* = 13.3 Hz, 1H), 2.62 (d, *J* = 13.3 Hz, 1H), 2.05 (s, 1H), 1.76 – 1.62 (m, 2H), 1.60 – 1.44 (m, 2H). **^{13}C NMR** (100 MHz, $CDCl_3$) δ 137.6,

130.5, 128.1, 126.2, 73.3, 68.7, 64.3, 39.6, 38.6, 29.4, 22.1. **HRMS** (ESI): calcd for $[M + H]^+$ $C_{13}H_{19}O_2$ 207.1390; found 207.1385.

3.2.6 General procedure for asymmetric desymmetrization of oxetanes using *Re*LEH mutants to synthesize chiral pyrrolidines and piperidines

Desymmetrization:

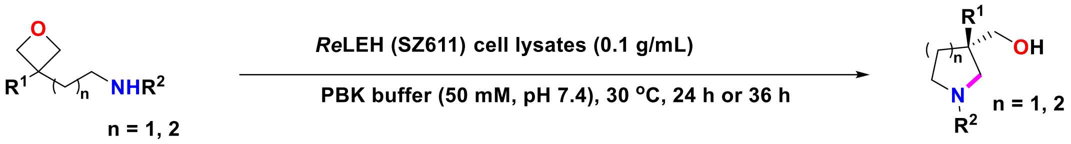

(*R*)-(1-Benzyl-3-phenylpyrrolidin-3-yl)methanol (46)

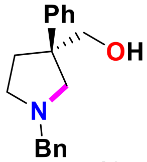

46

Obtained as a yellow oil after column chromatography (silica gel, PE/EA = 3:1→1:1). SZ611 (Y53F/N55A): Conversion = 64%; yield = 50%; e.r = 96:4; The enantioselectivity was determined by HPLC using a Daicel CHIRALCEL AD-H column (25 cm × 0.46 cm ID), [hexane/iso-propanol (0.1% diethylamine) = 98:2, λ = 220 nm, 1.0 mL/min flow rate] t_R = 17.2 min (major) and t_S = 18.5 min (minor). **^{1}H NMR** (400 MHz, $CDCl_3$) δ 7.44 – 7.33 (m, 7H), 7.27 (t, *J* = 7.3 Hz, 1H), 7.22 (d, *J* = 7.2 Hz, 2H), 3.79 – 3.62 (m, 3H), 3.56 (s, 1H), 3.46 (d, *J* = 8.7 Hz, 1H), 3.33 – 3.21 (m, 1H), 2.73 (dd, *J* = 8.8, 1.7 Hz, 1H), 2.63 – 2.52 (m, 1H), 2.46 – 2.25 (m, 2H), 1.31 (s, 1H). **^{13}C NMR** (100 MHz, $CDCl_3$) δ δ 145.0, 138.2, 128.7, 128.6, 128.4, 127.4, 126.7, 126.5, 75.6, 63.6, 60.1, 53.8, 50.8, 33.5. **HRMS** (ESI): calcd for $[M + H]^+$ $C_{18}H_{22}NO$ 268.1701; found 268.1703.

(*R*)-(1-Allyl-3-phenylpyrrolidin-3-yl)methanol (48)

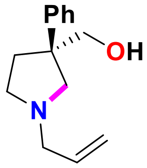

48

Obtained as a yellow oil after column chromatography (silica gel, PE/EA = 3:1→1:1). SZ611 (Y53F/N55A): Conversion = 94%; e.r = 90:10; The enantioselectivity was determined by HPLC using a Daicel CHIRALCEL AD-H column (25 cm × 0.46 cm ID), [hexane/iso-propanol (0.1% diethylamine) = 98:2, λ = 220 nm, 1.0 mL/min flow rate] t_R = 10.7 min (major) and t_S = 11.9 min (minor). **^{1}H NMR** (400 MHz, $CDCl_3$) δ δ 7.35 (t, *J* = 7.5 Hz, 2H), 7.30 – 7.25 (m, 1H), 7.22 (d, *J* = 7.2 Hz, 2H), 5.96 (tt, *J* = 12.8, 6.5 Hz, 1H), 5.38 – 5.12 (m, 2H), 4.39 (s, 1H), 3.75 (d, *J* = 10.1 Hz, 1H), 3.65 – 3.48 (m, 2H), 3.31 (td, *J* = 8.9, 3.4 Hz, 1H), 3.20 (d, *J* = 6.4 Hz, 2H), 2.70 (d, *J* = 9.2 Hz, 1H), 2.58 – 2.47 (m, 1H), 2.44 – 2.35 (m, 1H), 2.34 – 2.24 (m, 1H). **^{13}C NMR** (100 MHz, $CDCl_3$) δ 144.8, 134.5, 128.5, 128.4, 126.7, 126.6, 118.1, 75.0, 63.2, 58.4, 53.5, 50.9, 33.5. **HRMS** (ESI): calcd for $[M + H]^+$ $C_{14}H_{20}NO$ 218.1545; found 218.1549.

(*R*)-(3-Phenyl-1-(prop-2-yn-1-yl)pyrrolidin-3-yl)methanol (50)

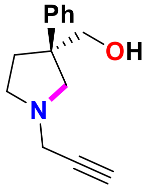

50

Obtained as a yellow oil after column chromatography (silica gel, PE/EA = 3:1→1:1). SZ611 (Y53F/N55A): Conversion = 85%; e.r = 94:6; The enantioselectivity was determined by HPLC using a Daicel CHIRALCEL AD-H column (25 cm × 0.46 cm ID), [hexane/iso-propanol (0.1% diethylamine) = 98:2, λ = 220 nm, 1.0 mL/min flow rate] t_R = 20.1 min (major) and t_S = 21.3 min (minor). **^{1}H NMR** (400 MHz, $CDCl_3$) δ δ7.37 (t, *J* = 7.5 Hz, 2H), 7.30 – 7.22 (m, 3H), 4.03 (s, 1H), 3.74 (d, *J* = 10.1 Hz, 1H), 3.59 (d, *J* = 10.2 Hz, 1H), 3.56 – 3.45 (m, 2H), 3.38 (d, *J* = 8.8 Hz, 1H), 3.16 (td, *J* = 8.8, 3.8 Hz, 1H), 2.97 (d, *J* = 8.8 Hz, 1H), 2.67 (dd, *J* = 16.8, 9.5 Hz, 1H), 2.49 (dt, *J* = 15.8, 8.1 Hz, 1H), 2.39 – 2.22 (m, 2H). **^{13}C NMR** (100 MHz, $CDCl_3$) δ 145.0, 128.4, 126.8, 126.6, 78.3, 74.5, 73.5, 61.4, 51.6, 51.2, 42.4, 33.7. **HRMS** (ESI): calcd for $[M + H]^+$ $C_{14}H_{18}NO$ 216.1388; found 216.1386.

(*R*)-(1-Butyl-3-phenylpyrrolidin-3-yl)methanol (52)

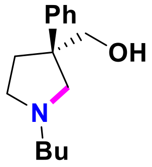

52

Obtained as a yellow oil after column chromatography (silica gel, PE/EA = 3:1→1:1). SZ611 (Y53F/N55A): Conversion = 98%; e.r = 96:4; The enantioselectivity was determined by HPLC using a Daicel CHIRALCEL AD-H column (25 cm × 0.46 cm ID), [hexane/iso-propanol (0.1% diethylamine) = 98:2, λ = 220 nm, 1.0 mL/min flow rate] t_R = 14.7 min (major) and t_S = 13.4 min (minor). **^{1}H NMR** (400 MHz, $CDCl_3$) δ δ 7.36 (t, *J* = 7.5 Hz, 2H), 7.27 (d, *J* = 7.1 Hz, 1H), 7.23 (d, *J* = 7.4 Hz, 2H), 4.83 (s, 1H), 3.76 (d, *J* = 10.0 Hz, 1H), 3.56 (d, *J* = 9.4 Hz, 2H), 3.43 – 3.31 (m, 1H), 2.72 (dd, *J* = 8.9, 1.8 Hz, 1H), 2.62 (dt, *J* = 11.8, 7.5 Hz, 1H), 2.58 – 2.48 (m, 2H), 2.41 – 2.25 (m, 2H), 1.67 – 1.55 (m, 2H), 1.48 – 1.36 (m, 2H), 0.98 (t, *J* = 7.3 Hz, 3H). **^{13}C NMR** (100 MHz, $CDCl_3$) δ 144.7, 128.4, 126.6, 126.6, 75.2, 63.7, 55.4, 53.7, 50.7, 33.4, 30.3, 20.6, 14.0. **HRMS** (ESI): calcd for $[M + H]^+$ $C_{15}H_{24}NO$ 234.1858; found 234.1857.

(*R*)-(1,3-Diphenylpyrrolidin-3-yl)methanol (54)

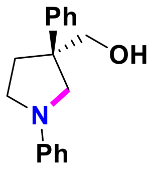

54

Obtained as a yellow oil after column chromatography (silica gel, PE/EA = 25:1→8:1). SZ611 (Y53F/N55A): Conversion = 99%; yield = 88%; e.r = 99:1; The enantioselectivity was determined by HPLC using a Daicel CHIRALCEL AD-H column (25 cm × 0.46 cm ID), [hexane/iso-propanol (0.1% diethylamine) = 90:10, λ = 220 nm, 1.0 mL/min flow rate] t_R = 8.3 min (major) and t_S = 10.9 min (minor). **^{1}H NMR** (400 MHz, $CDCl_3$) δ 7.45 (t, *J* = 7.5 Hz, 2H), 7.34 (dd, *J* = 15.9, 7.8 Hz, 5H), 6.78 (t, *J* = 7.2 Hz, 1H), 6.70 (d, *J* = 8.0 Hz, 2H), 3.84 (d, *J* = 9.3 Hz, 1H), 3.59 (t, *J* = 7.6 Hz, 2H), 3.46 (t, *J* = 8.1 Hz, 1H), 2.72 – 2.51 (m, 1H), 2.41 – 2.19 (m, 1H), 1.60 (s, 1H). **^{13}C NMR** (100 MHz, $CDCl_3$) δ 147.7, 143.8, 129.3, 128.7, 127.0, 126.9, 116.0, 111.8, 69.3, 54.8, 51.8, 46.6, 32.3. **HRMS** (ESI): calcd for $[M + H]^+$ $C_{17}H_{20}NO$ 254.1545; found 254.1541.

(*R*)-(3-Phenyl-1-(*p*-tolyl)pyrrolidin-3-yl)methanol (56)

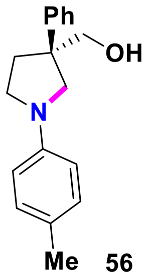

56

Obtained as a yellow oil after column chromatography (silica gel , PE/EA = 25:1→8:1). SZ611 (Y53F/N55A): Conversion = 99%; e.r = 98:2; The enantioselectivity was determined by HPLC using a Daicel CHIRALCEL AD-H column (25 cm × 0.46 cm ID), [hexane/iso-propanol (0.1% diethylamine) = 90:10, λ = 220 nm, 1.0 mL/min flow rate] t_R = 8.1 min (major) and t_S = 10.9 min (minor). **^{1}H NMR** (400 MHz, $CDCl_3$δ 7.44 – 7.41 (m, 2H), 7.38 – 7.32 (m, 3H), 7.12 (d, *J* = 8.1 Hz, 2H), 6.62 (d, *J* = 8.3 Hz, 2H), 3.87 – 3.69 (m, 3H), 3.63 – 3.49 (m, 2H), 3.41 (td, *J* = 9.1, 2.8 Hz, 1H), 2.63 – 2.53 (m, 1H), 2.36 – 2.28 (m, 4H), 1.58 (s, 1H). **^{13}C NMR** (100 MHz, $CDCl_3$) δ 145.8, 144.0, 129.8, 128.7, 127.0, 126.9, 125.2, 111.9, 69.5, 55.2, 51.8, 46.9, 32.4, 20.4. **HRMS** (ESI): calcd for $[M + H]^+$ $C_{18}H_{22}NO$ 268.1701; found 268.1706.

(*R*)-(1-(4-Chlorophenyl)-3-phenylpyrrolidin-3-yl)methanol (58)

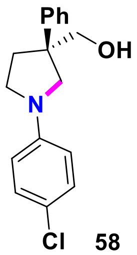

58

Obtained as a yellow oil after column chromatography (silica gel, PE/EA = 25:1→8:1). SZ611 (Y53F/N55A): Conversion = 99%; e.r = 98:2; The enantioselectivity was determined by HPLC using a Daicel CHIRALCEL AD-H column (25 cm × 0.46 cm ID), [hexane/iso-propanol (0.1% diethylamine)= 98:2, λ = 220 nm, 1.0 mL/min flow rate] t_R = 10.1 min (major) and t_S = 13.7 min (minor). **^{1}H NMR** (400 MHz, $CDCl_3$) δ 7.46 (t, J = 7.5 Hz, 2H), 7.38 – 7.19 (m, 5H), 6.60 (d, *J* = 8.8 Hz, 2H), 3.75 (d, *J* = 9.3 Hz, 1H), 3.62 (q, *J* = 11.0 Hz, 2H), 3.49 (dd, *J* = 15.0, 8.8 Hz, 2H), 3.38 (dd, *J* = 9.1, 6.6 Hz, 1H), 2.50 (dd, *J*

= 10.0, 7.1 Hz, 1H), 2.39 – 2.11 (m, 2H), 1.44 (s, 1H). **^{13}C NMR** (100 MHz, $CDCl_3$) δ 146.4, 143.9, 129.1, 128.7, 127.0, 120.7, 112.8, 68.9, 54.8, 51.8, 46.7, 32.4. **HRMS** (ESI): calcd for $[M + H]^+$ $C_{17}H_{19}NOCl$ 288.1155; found 288.1157.

(*R*)-(1-Benzyl-3-(*p*-tolyl)pyrrolidin-3-yl)methanol (62)

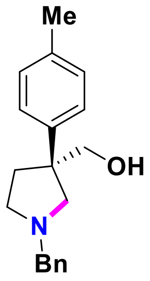

62

Obtained as a yellow oil after column chromatography (silica gel, PE/EA = 3:1→1:1). SZ611 (Y53F/N55A): Conversion = 53%; e.r = 94:6; The enantioselectivity was determined by HPLC using a Daicel CHIRALCEL AD-H column (25 cm × 0.46 cm ID), [hexane/iso-propanol (0.1% diethylamine) = 90:10, λ = 220 nm, 1.0 mL/min flow rate] t_R = 6.5 min (major) and t_S = 7.3 min (minor). **^{1}H NMR** (400 MHz, $CDCl_3$) δ 7.45 – 7.32 (m, 5H), 7.19 (d, *J* = 8.0 Hz, 2H), 7.13 (d, *J* = 8.2 Hz, 2H), 3.79 – 3.69 (m, 3H), 3.58 (dd, *J* = 9.9, 1.8 Hz, 1H), 3.51 (s, 1H), 3.46 (d, *J* = 8.8 Hz, 1H), 3.27 (td, *J* = 8.7, 2.7 Hz, 1H), 2.72 (dd, *J* = 8.8, 1.7 Hz, 1H), 2.55 (dt, *J* = 12.1, 8.1 Hz, 1H), 2.46 – 2.37 (m, 4H), 2.36 – 2.27 (m, 1H). **^{13}C NMR** (100 MHz, $CDCl_3$) δ 141.9, 138.2, 136.1, 129.1, 128.7, 128.6, 127.4, 126.6, 75.5, 63.7, 60.1, 53.8, 50.5, 33.6, 21.0. **HRMS** (ESI): calcd for $[M + H]^+$ $C_{19}H_{24}NO$ 282.1858; found 282.1856.

(*R*)-(1-Benzyl-3-(*m*-tolyl)pyrrolidin-3-yl)methanol (64)

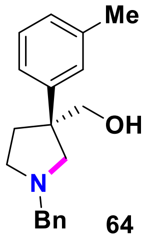

64

Obtained as a yellow oil after column chromatography (silica gel, PE/EA = 3:1→1:1). SZ611 (Y53F/N55A): Conversion = 89%; e.r = 97:3; The enantioselectivity was determined by HPLC using a Daicel CHIRALCEL AD-H column (25 cm × 0.46 cm ID), [hexane/iso-propanol (0.1% diethylamine) = 90:10, λ = 220 nm, 1.0 mL/min flow rate] t_R = 6.0 min (major) and t_S = 7.6 min (minor). **^{1}H NMR** (400 MHz, $CDCl_3$) δ 7.45 – 7.29 (m, 5H), 7.24 (t, *J* = 7.9 Hz, 1H), 7.08 (d, *J* = 7.5 Hz, 1H), 7.02 (d, *J* = 6.4 Hz, 2H), 4.43 (s, 1H), 3.80 – 3.67 (m, 3H), 3.56 (d, *J* = 9.9 Hz, 1H), 3.45 (d, *J* = 8.8 Hz, 1H), 3.26 (dd, *J* = 12.3, 5.3 Hz, 1H), 2.72 (d, *J* = 8.8 Hz, 1H), 2.59 – 2.48 (m, 1H), 2.43 – 2.26 (m, 5H). **^{13}C NMR** (100 MHz, $CDCl_3$) δ 144.8, 138.0, 128.8, 128.6, 128.3, 127.5, 127.4, 127.3, 123.7, 75.4, 63.5, 60.1, 53.7, 50.8, 33.5, 21.6. **HRMS** (ESI): calcd for $[M + H]^+$ $C_{19}H_{24}NO$ 282.1858; found 282.1857.

(*R*)-(1-Benzyl-3-(3,5-dimethylphenyl)pyrrolidin-3-yl)methanol (66)

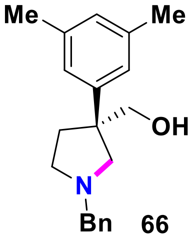

66

Obtained as a yellow oil after column chromatography (silica gel, PE/EA = 3:1→1:1). SZ611 (Y53F/N55A): Conversion = 69%; e.r = 93:7; The enantioselectivity was determined by HPLC using a Daicel CHIRALCEL AD-H column (25 cm × 0.46 cm ID), [hexane/iso-propanol (0.1% diethylamine)= 90:10, λ = 220 nm, 1.0 mL/min flow rate] t_R = 5.1 min (major) and t_S = 5.9 min (minor). **^{1}H NMR** (400 MHz, $CDCl_3$) δ 7.46 – 7.38 (m, 4H), 7.37 – 7.32 (m, 1H), 6.93 (s, 1H), 6.84 (s, 2H), 3.78 – 3.69 (m, 3H), 3.57 (dd, *J* = 9.9, 1.9 Hz, 1H), 3.44 (d, *J* = 8.8 Hz, 1H), 3.29 – 3.22 (m, 1H), 2.71 (dd, *J* = 8.8, 1.9 Hz, 1H), 2.59 – 2.48 (m, 1H), 2.43 – 2.37 (m, 1H), 2.36 (s, 6H), 2.34 – 2.26 (m, 1H). **^{13}C NMR** (100 MHz, $CDCl_3$) δ 144.9, 138.3, 137.9, 128.7, 128.6, 128.2, 127.4, 124.6, 75.6, 63.6, 60.1, 53.8, 50.7, 33.5, 21.5. **HRMS** (ESI): calcd for $[M + H]^+$ $C_{20}H_{26}NO$ 296.2014; found 296.2017.

(*R*)-(1-Allyl-3-(4-ethylphenyl)pyrrolidin-3-yl)methanol (70)

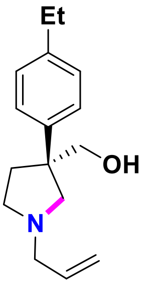

70

Obtained as a yellow oil after column chromatography (silica gel, PE/EA = 3:1→1:1). SZ611 (Y53F/N55A): Conversion = 73%; e.r = 85:15; The enantioselectivity was determined by HPLC using a Daicel CHIRALCEL AD-H column (25 cm × 0.46 cm ID), [hexane/iso-propanol (0.1% diethylamine) = 98:2, λ = 220 nm, 1.0 mL/min flow rate] t_R = 10.0 min (major) and t_S = 11.9 min (minor). **^{1}H NMR** (400 MHz, $CDCl_3$) δ 7.17 (dd, *J* = 20.3, 8.2 Hz, 4H), 6.13 – 5.83 (m, 1H), 5.35 – 5.12 (m, 2H), 4.52 (s, 1H), 3.75 (d, *J* = 10.0 Hz, 1H), 3.64 – 3.46 (m, 2H), 3.37 – 3.25 (m, 1H), 3.19 (d, *J* = 6.4 Hz, 2H), 2.74 – 2.63 (m, 3H), 2.56 – 2.48 (m, 1H), 2.44 – 2.21 (m, 2H), 1.27 (t, *J* = 7.6 Hz, 3H). **^{13}C NMR** (100 MHz, $CDCl_3$) δ 142.5, 142.1, 134.8, 127.9, 126.6, 117.8, 75.2, 63.4, 58.4, 53.6, 50.5, 33.5, 28.4, 15.6. **HRMS** (ESI): calcd for $[M + H]^+$ $C_{16}H_{24}NO$ 246.1858; found 246.1859.

(*R*)-(1-Benzyl-3-(4-methoxyphenyl)pyrrolidin-3-yl)methanol (72)

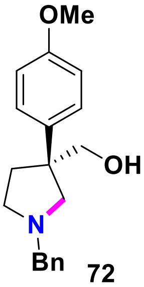

72

Obtained as a yellow oil after column chromatography (silica gel, PE/EA (0.1% Et_3N)= 3 : 1→1:1). SZ611 (Y53F/N55A): Conversion = 74%; e.r = 98:2; The enantioselectivity was determined by HPLC using a Daicel CHIRALCEL AD-H column (25 cm × 0.46 cm ID), [hexane/iso-propanol (0.1% diethylamine) = 90:10, λ = 220 nm, 1.0 mL/min flow rate] t_R = 9.6 min (major) and t_S = 10.3 min (minor). **^{1}H NMR** (400 MHz, $CDCl_3$) δ 7.48 – 7.31 (m, 2H), 7.19 – 7.10 (m, 1H), 6.94 – 6.86 (m, 1H), 3.83 (s, 1H), 3.75 – 3.66 (m, 1H), 3.54 (dd, *J* = 9.9, 1.9 Hz, 1H), 3.49 (s, 1H), 3.42 (d, *J* = 8.8 Hz, 1H), 3.24 (td, *J* = 8.7, 3.0 Hz, 1H), 2.69 (dd, *J* = 8.8, 1.8 Hz, 1H), 2.58 – 2.47 (m, 1H), 2.44 – 2.36 (m, 1H), 2.32 – 2.24 (m, 1H). **^{13}C NMR** (100 MHz, $CDCl_3$) δ 158.1, 138.2, 137.1, 128.7, 128.6, 127.7, 127.4, 113.8, 75.4, 63.8, 60.1, 55.3, 53.8, 50.1, 33.7. **HRMS** (ESI): calcd for $[M + H]^+$ $C_{19}H_{24}NO_2$ 218.1545; found 218.1547.

(*R*)-(1-Allyl-3-(4-(methylthio)phenyl)pyrrolidin-3-yl)methanol (76)

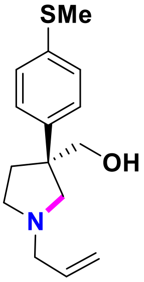

76

Obtained as a yellow oil after column chromatography (silica gel, PE/EA = 3:1→1:1). SZ611 (Y53F/N55A): Conversion = 73%; e.r = 89:11; The enantioselectivity was determined by HPLC using a Daicel CHIRALCEL AD-H column (25 cm × 0.46 cm ID), [hexane/iso-propanol (0.1% diethylamine) = 98:2, λ = 220 nm, 1.0 mL/min flow rate] t_R = 21.5 min (major) and t_S = 27.4 min (minor). **^{1}H NMR** (400 MHz, $CDCl_3$) δ 7.26 (d, *J* = 8.2 Hz, 2H), 7.15 (d, *J* = 8.2 Hz, 2H), 5.94 (tt, *J* = 12.5, 6.1 Hz, 1H), 5.23 (dd, *J* = 29.3, 13.6 Hz, 2H), 3.73 (d, *J* = 9.9 Hz, 1H), 3.73 (d, *J* = 9.9 Hz, 1H), 3.53 (d, *J* = 10.0 Hz, 1H), 3.45 (d, *J* = 8.5 Hz, 1H), 3.27 (t, *J* = 8.7 Hz, 1H), 3.16 (d, *J* = 6.2 Hz, 2H), 2.62 (d, *J* = 8.9 Hz, 1H), 2.57 – 2.43 (m, 4H), 2.40 – 2.11 (m, 2H). **^{13}C NMR** (100 MHz, $CDCl_3$) δ 141.8, 136.5, 134.9, 127.2, 126.8, 117.6, 75.0, 63.3, 58.4, 53.5, 50.4, 33.5, 16.0. **HRMS** (ESI): calcd for $[M + H]^+$ $C_{15}H_{22}NOS$ 264.1422; found 264.1426.

(*R*)-(1-Benzyl-3-(4-fluorophenyl)pyrrolidin-3-yl)methanol (78)

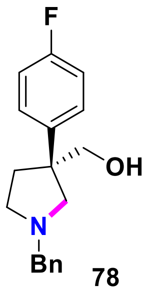

78

Obtained as a yellow oil after column chromatography (silica gel, PE/EA= 3:1→1:1). SZ611 (Y53F/N55A): Conversion = 61%; e.r = 94:6; The enantioselectivity was determined by HPLC using a Daicel CHIRALCEL IC column (25 cm × 0.46 cm ID), [hexane/iso-propanol (0.1% diethylamine) = 97:3, λ = 220 nm, 1.0 mL/min flow rate] t_{S} = 14.9 min (minor) and t_{R} = 16.5 min (major). **^{1}H NMR** (400 MHz, $CDCl_3$) δ 7.44 – 7.32 (m, 5H), 7.21 – 7.14 (m, 2H), 7.03 (t, *J* = 8.7 Hz, 2H), 3.75 – 3.65 (m, 3H), 3.56 – 3.49 (m, 1H), 3.41 (d, *J* = 8.8 Hz, 1H), 3.25 (td, *J* = 8.9, 3.0 Hz, 1H), 2.69 (dd, *J* = 8.8, 1.8 Hz, 1H), 2.53 (dt, *J* = 12.2, 8.1 Hz, 1H), 2.41 (dd, *J* = 17.0, 9.3 Hz, 1H), 2.31 – 2.22 (m, 1H). **^{19}F NMR** (100 MHz, $CDCl_3$) -116.24 – -116.37 (m). **^{13}C NMR** (100 MHz, $CDCl_3$) δ 161.4 (d, *J* = 245.2 Hz), 140.7 (d, *J* = 3.2 Hz), 138.0, 128.7, 128.6, 128.2 (d, *J* = 7.9 Hz), 127.4, 115.1 (d, *J* = 21.1 Hz), 75.4, 63.6, 60.0, 53.7, 50.3, 33.6. **HRMS** (ESI): calcd for $[M + H]^+$ $C_{18}H_{21}NOF$ 286.1607; found 286.1606.

(*R*)-(1-Benzyl-3-(4-chlorophenyl)pyrrolidin-3-yl)methanol (80)

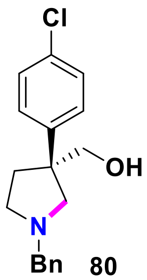

80

Obtained as a yellow oil after column chromatography (silica gel, PE/EA= 3:1→1:1). SZ611 (Y53F/N55A): Conversion = 57%; e.r = 91:9; The enantioselectivity was determined by HPLC using a Daicel CHIRALCEL IC column (25 cm × 0.46 cm ID), [hexane/iso-propanol (0.1% diethylamine) = 97:3, λ = 220 nm, 1.0 mL/min flow rate] t_{S}= 15.5 min (minor) and t_{R} = 18.6 min (major). **^{1}H NMR** (400 MHz, $CDCl_3$) δ 7.43 – 7.29 (m, 7H), 7.14 (d, *J* = 8.4 Hz, 2H), 3.76 – 3.64 (m, 3H), 3.53 (dd, *J* = 10.2, 1.7 Hz, 1H), 3.40 (d, *J* = 8.8 Hz, 1H), 3.25 (td, *J* = 8.8, 3.0 Hz, 1H), 2.67 (dd, *J* = 8.8, 1.5 Hz, 1H), 2.52 (dt, *J* = 12.2, 8.1 Hz, 1H), 2.41 (dd, *J* = 17.0, 9.2 Hz, 1H), 2.29 – 2.20 (m, 1H). **^{13}C NMR** (100 MHz, $CDCl_3$) δ 143.5, 138.1, 132.4, 128.7, 128.6, 128.5, 128.1, 127.4, 75.2, 63.4, 60.0, 53.7, 50.5, 33.5. **HRMS** (ESI): calcd for $[M + H]^+$ $C_{18}H_{21}NOCl$ 302.1312; found 302.1314.

(*R*)-(1-Benzyl-3-(4-bromophenyl)pyrrolidin-3-yl)methanol (82)

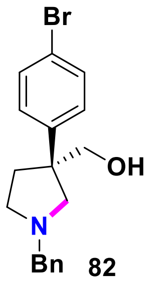

82

Obtained as a yellow oil after column chromatography (silica gel, PE/EA= 3:1→1:1). SZ611 (Y53F/N55A): Conversion = 25%; e.r = 88:12; The enantioselectivity was determined by HPLC using a Daicel CHIRALCEL AD-H column (25 cm × 0.46 cm ID), [hexane/iso-propanol (0.1% diethylamine) = 90:10, λ = 220 nm, 1.0 mL/min flow rate] t_{S} = 7.7 min (minor) and t_{R} = 8.0 min (major). **^{1}H NMR** (400 MHz, $CDCl_3$) δ 7.46 (d, *J* = 8.5 Hz, 2H), 7.37 (dd, *J* = 11.9, 6.8 Hz, 5H), 7.08 (d, *J* = 8.5 Hz, 2H), 3.75 – 3.67 (m, 3H), 3.54 – 3.50 (m, 2H), 3.39 (d, *J* = 8.8 Hz, 1H), 3.24 (td, *J* = 8.9, 3.1 Hz, 1H), 2.66 (dd, *J* = 8.8, 2.0 Hz, 1H), 2.56 – 2.47 (m, 1H), 2.45 – 2.36 (m, 1H), 2.29 – 2.20 (m, 1H). **^{13}C NMR** (100 MHz, $CDCl_3$) δ 143.9, 138.0, 131.4, 128.7, 128.6, 128.5, 127.4, 120.4, 75.1, 63.4, 60.0, 53.7, 50.5, 33.4. **HRMS** (ESI): calcd for $[M + H]^+$ $C_{18}H_{21}NOBr$ 346.0807; found 346.0803.

(*R*)-(1-Allyl-3-(4-(trifluoromethyl)phenyl)pyrrolidin-3-yl)methanol (86)

Obtained as a colorless oil after column chromatography (silica gel, PE/EA= 3:1→1:1). SZ611 (Y53F/N55A): Conversion = 85%; e.r = 80:20; The enantioselectivity was determined by HPLC using a Daicel CHIRALCEL AD-H column (25 cm × 0.46 cm ID), [hexane/iso-propanol (0.1% diethylamine) = 98:2, λ = 220 nm, 1.0 mL/min flow rate] t_{R} = 10.7 min (major) and t_{S} = 11.9 min (minor). **^{1}H NMR** (400

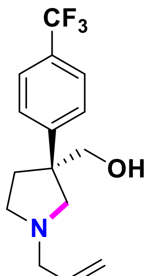

86

MHz, $CDCl_3$) δ 7.61 (d, *J* = 8.2 Hz, 1H), 7.40 – 7.32 (m, 2H), 7.29 – 7.21 (m, 2H), 6.08 – 5.86 (m, 1H), 5.43 – 5.04 (m, 2H), 4.58 (s, 1H), 3.74 (dd, *J* = 10.0, 2.9 Hz, 1H), 3.61 – 3.54 (m, 1H), 3.48 (t, *J* = 8.1 Hz, 1H), 3.36 – 3.20 (m, 1H), 3.18 (d, *J* = 6.4 Hz, 2H), 2.72 – 2.63 (m, 1H), 2.57 – 2.45 (m, 1H), 2.42 – 2.20 (m, 2H). **^{19}F NMR** (100 MHz, $CDCl_3$) -62.37 (s). **^{13}C NMR** (100 MHz, $CDCl_3$) δ 145.0, 135.0/134.9, 128.78 (q, 32.7 Hz), 128.39/127.2, 126.69/126.5, 125.28 (q, *J* = 3.9 Hz), 124.18 (q, *J* = 271.4 Hz), 117.7/117.6, 75.1/74.5, 63.3/63.0, 58.42 (2), 53.54/53.4, 51.0/50.8, 33.5(2). **HRMS** (ESI): calcd for $[M + H]^+$ $C_{15}H_{19}NOF_3$ 286.1419; found 286.1421.

(*S*)-(1,3-Dibenzylpyrrolidin-3-yl)methanol (88)

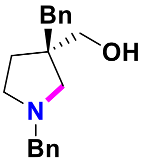

88

Obtained as a colorless oil after column chromatography (silica gel, PE/EA = 3:1→1:1). SZ611 (Y53F/N55A): Conversion = 98%; e.r = 85:15; The enantioselectivity was determined by HPLC using a Daicel CHIRALCEL AD-H column (25 cm × 0.46 cm ID), [hexane/iso-propanol (0.1% diethylamine) = 98:2, λ = 220 nm, 1.0 mL/min flow rate] t_R = 20.2 min (major) and t_S = 23.1 min (minor). **^{1}H NMR** (400 MHz, $CDCl_3$) δ 7.40 – 7.25 (m, 8H), 7.19 – 7.14 (m, 2H), 3.69 – 3.57 (m, 3H), 3.44 (dd, *J* = 9.8, 1.5 Hz, 1H), 3.04 (td, *J* = 8.8, 4.0 Hz, 1H), 2.87 (d, *J* = 9.1 Hz, 1H), 2.79 – 2.70 (m, 2H), 2.45 – 2.40 (m, 1H), 2.33 (dd, *J* = 17.2, 9.4 Hz, 1H), 2.01 – 1.85 (m, 2H). **^{13}C NMR** (100 MHz, $CDCl_3$) δ 138.23, 129.95, 128.68, 128.49, 128.21, 127.31, 126.38, 71.53, 63.73, 59.99, 53.76, 46.43, 42.70, 32.49. **HRMS** (ESI): calcd for $[M + H]^+$ $C_{19}H_{24}NO$ 282.1858; found 282.1861.

(*R*)-(1-Benzyl-3-phenylpiperidin-3-yl)methanol (90)

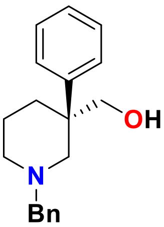

90

Obtained as a colorless oil after column chromatography (silica gel, PE/EA= 3:1→1:1). SZ611 (Y53F/N55A): Conversion = 99%; e.r = 99:1; The enantioselectivity was determined by HPLC using a Daicel CHIRALCEL AD-H column (25 cm × 0.46 cm ID), [hexane/iso-propanol (0.1% diethylamine) = 98:2, λ = 220 nm, 1.0 mL/min flow rate] t_R = 16.8 min (major) and t_S = 17.8 min (minor). **^{1}H NMR** (400 MHz, $CDCl_3$) δ 7.62 – 7.50 (m, 10H), 7.47 – 7.41 (m, 1H), 4.04 (dd, *J* = 24.2, 10.4 Hz, 2H), 3.89 – 3.73 (m, 2H), 3.42 – 3.19 (m, 1H), 3.08 – 2.82 (m, 2H), 2.54 – 2.41 (m, 1H), 2.34 – 2.22 (m, 2H), 2.08 – 1.97 (m, 1H), 1.94 – 1.80 (m, 1H). **^{13}C NMR** (100 MHz, $CDCl_3$) δ 144.7, 138.0, 129.1, 128.4 (2), 127.3, 126.4, 126.2, 73.1, 63.6, 61.4, 54.0, 50.8, 42.3, 33.0, 22.9. **HRMS** (ESI): calcd for $[M + H]^+$ $C_{19}H_{24}NO$ 282.1858; found 282.1861.

(*R*)-(1,3-Dibenzylpiperidin-3-yl)methanol (92)

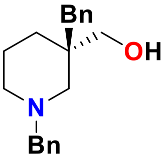

92

Obtained as a colorless oil after column chromatography (silica gel, PE/EA (0.1% Et_3N)= 3:1→1:1). SZ611 (Y53F/N55A): Conversion = 47%; e.r = 71:29; The enantioselectivity was determined by HPLC using a Daicel CHIRALCEL AD-H column (25 cm × 0.46 cm ID), [hexane/iso-propanol (0.1% diethylamine) = 90:10, λ = 220 nm, 1.0 mL/min flow rate] t_R = 6.1 min (major) and t_S = 9.0 min (minor). **^{1}H NMR** (400 MHz, $CDCl_3$) δ 7.41 – 7.27 (m, 8H), 7.16 – 7.11 (m, 2H), 3.65 (dd, *J* = 25.3, 10.2 Hz, 2H), 3.50 (dd, *J* = 17.0, 7.7 Hz, 2H), 2.75 (s, 2H), 2.54 (dd, *J* = 36.8, 13.3 Hz, 2H), 2.19 (d, *J* = 11.2 Hz, 1H), 2.13 – 1.93 (m, 2H), 1.73 – 1.57 (m, 2H), 1.47 – 1.33 (m, 1H). **^{13}C NMR** (100 MHz, $CDCl_3$) δ 137.7, 137.6, 130.5, 129.1, 128.4, 128.0, 127.3, 126.2, 71.1, 63.4, 62.3, 53.7, 50.9, 38.0, 32.7, 22.7. **HRMS** (ESI): calcd for $[M + H]^+$ $C_{20}H_{26}NO$ 296.2014; found 296.2011.

3.3 Synthetic transformations and derivatizations

Ethyl (*R*, *E*)-3-(1-benzyl-3-phenylpyrrolidin-3-yl)acrylate (93)

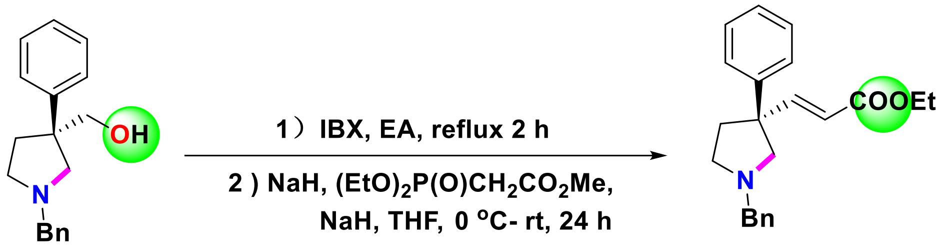

46 **93**

To a solution of **46** (53 mg, 0.2 mmol) in EtOAc (1 mL) was added IBX (56 mg, 0.2 mmol). The reaction was refluxed for 2 h until complete conversion (TLC). The suspension was cooled to room temperature and passed through a plug of celite eleuting with EtOAc. The filtrates were combined, concentrated under reduced pressure. Neat triethyl phosphonoacetate (56 mg, 0.25 mml, 1.25 equiv) was added to a suspension of NaH (10 mg, 1.25 equiv, 60% dispersion in mineral oil) dry THF. The reaction mixture was stirred at room temperature for 10 minutes and the above crude product in THF (0.2 mL) was added. The reaction mixture was stirred at room temperature overnight and then carefully quenched with a saturated $NaHCO_3$ solution. The reaction mixture was transferred to a separation funnel using EtOAc/water. The aqueous phase was extracted with EtOAc (three times). The combined organic phases were washed with brine, dried over $MgSO_4$, filtered and concentrated under reduced pressure. Final purification was accomplished by silica gel column chromatography to yield the product (23 mg, 34%) as a yellow oil.

The enantioselectivity was determined by HPLC using a Daicel CHIRALCEL OJ-H column (25 cm × 0.46 cm ID), [hexane/iso-propanol (0.1% diethylamine) = 97:3, λ = 220 nm, 1.0 mL/min flow rate] t_R = 6.9 min (major) and t_S = 8.0 min (minor);

^{1}H NMR (400 MHz, $CDCl_3$)δ 7.43 – 7.21 (m, 11H), 5.57 (d, *J* = 15.8 Hz, 1H), 4.20 (q, *J* = 7.2 Hz, 2H), 3.75 (s, 2H), 3.20 (d, *J* = 9.2 Hz, 1H), 3.05 (dd, *J* = 14.9, 8.0 Hz, 1H), 2.90 (d, *J* = 9.1 Hz, 1H), 2.66 (dd, *J* = 14.2, 7.5 Hz, 1H), 2.48 – 2.31 (m, 2H), 1.31 (t, *J* = 7.1 Hz, 5H).**^{13}C NMR** (100 MHz, $CDCl_3$) δ 167.2, 156.3, 145.0, 139.3, 128.6, 128.5, 128.3, 127.1, 127.0, 126.6, 118.7, 64.1, 60.3, 60.2, 52.8, 52.2, 36.9, 14.3. **HRMS** (ESI): calcd for $[M + H]^+$ $C_{18}H_{21}NI$ 378.0721; found 378.0719.

(*R*)-(1-Benzyl-3-phenylpyrrolidin-3-yl)methyl 4-methylbenzenesulfonate (94)

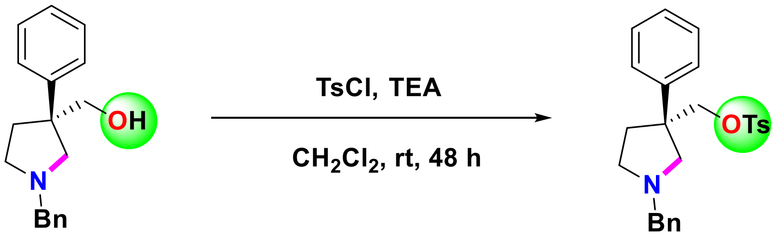

46 **94**

To a solution of **46** (160 mg, 0.6 mmol) in DCM (6 mL) was added Et_3N (168 µL, 1.2 mmol, 2.0 equiv) at 0 °C, followed by TsCl (229 mg,1.2 mmol, 2.0 equiv) in portions. The mixture was allowed to warm to room temperature and stirred for 12 h. Then, saturated aqueous $NaHCO_3$ was added at 0 °C, and the mixture was extracted three times with CH_2Cl_2. The combined organic layer was dried over anhydrous $MgSO_4$, filtered, and concentrated under reduced pressure. The residue was purified by column chromatography (silica gel, PE/EA = 20:1→6:1) to give the desired product (225 mg, 89%) as a white solid. The enantioselectivity was determined by HPLC using a Daicel CHIRALCEL AD-H column (25 cm × 0.46 cm ID), [hexane/iso-propanol (0.1% diethylamine) = 98:2, λ = 220 nm, 1.0 mL/min flow rate] t_R = 18.8 min (major) and t_S = 20.2 min (minor);

^{1}H NMR (400 MHz, $CDCl_3$) δ 7.54 (d, *J* = 8.1 Hz, 2H), 7.40 – 7.23 (m, 10H), 7.14 (d, *J* = 7.3 Hz, 2H), 4.29 (d, *J* = 8.8 Hz, 1H), 4.11 (d, *J* = 8.8 Hz, 1H), 3.66 (dd, *J* = 41.8, 13.1 Hz, 2H), 3.15 (d, *J* = 9.4 Hz, 1H), 2.98 – 2.83 (m, 1H), 2.66 (d, *J* = 9.4 Hz, 1H), 2.53 – 2.34 (m, 4H), 2.24 – 2.00 (m, 2H). **^{13}C NMR** (100 MHz, $CDCl_3$) δ 144.4, 144.2, 139.1, 132.7, 129.7, 128.5, 128.3, 128.2, 127.8, 127.0, 126.5, 76.3, 61.3, 59.8, 52.7, 49.7, 34.2, 21.7. **HRMS** (ESI): calcd for $[M + H]^+$ $C_{25}H_{28}NO_3S$ 422.1794; found 422.1790.

(*R*)-2-(1-Benzyl-3-phenylpyrrolidin-3-yl)acetonitrile (95)

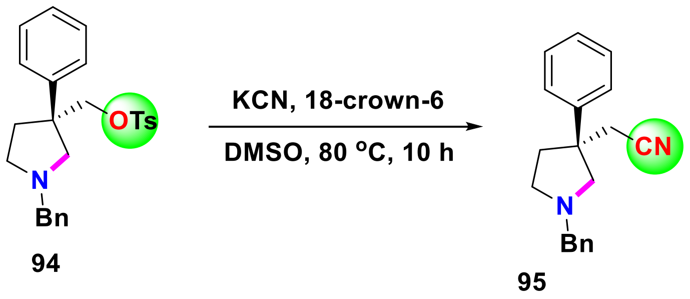

To a solution of **94** (42.2 mg, 0.1 mmol, 1.0 equiv) in DMSO (1 mL) was added KCN (12.2 mg, 0.2 mmol, 2.0 equiv) and 18-crown-6 (53 mg, 0.2 mmol, 2.0 equiv) at room temperature. The mixture was stirred at 80 °C for 10 h. Then, the mixture was diluted with EtOAc/water (1:1). The aqueous phase was extracted with EtOAc (two times). The combined organic phases were washed with water (two times) and brine, dried over $MgSO_4$, filtered and concentrated under reduced pressure. Final purification was accomplished by silica gel column chromatography (by using PE/EA = 20:1 to 4:1) to give the desired product **95** (20.2 mg, 73%) as a yellow oil.

The enantioselectivity was determined by HPLC using a Daicel CHIRALCEL AS-H column (25 cm × 0.46 cm ID), [hexane/iso-propanol (0.1% diethylamine)= 99:1, λ = 220 nm, 1.0 mL/min flow rate] t_R = 21.8 min (major) and t_S = 23.8 min (minor);

^{1}H NMR (400 MHz, $CDCl_3$)δ 7.43 – 7.36 (m, 6H), 7.34 – 7.28 (m, 4H), 3.75 (q, *J* = 13.0 Hz, 2H), 3.22 (d, *J* = 9.2 Hz, 1H), 3.19 – 3.10 (m, 1H), 2.91 (q, *J* = 16.4 Hz, 2H), 2.70 (d, *J* = 9.3 Hz, 1H), 2.50 (dd, *J* = 16.6, 9.2 Hz, 1H), 2.39 – 2.21 (m, 2H). **^{13}C NMR** (100 MHz, $CDCl_3$) δ 144.63, 139.0, 128.7, 128.5, 128.4, 127.1, 126.5, 118.6. 63.7. 59.7, 52.5, 48.0, 36.2, 32.0. **HRMS** (ESI): calcd for $[M + H]^+$ $C_{19}H_{21}N_2$ 277.1709; found 277.1705.

(*R*)-3-(Azidomethyl)-1-benzyl-3-phenylpyrrolidine (96)

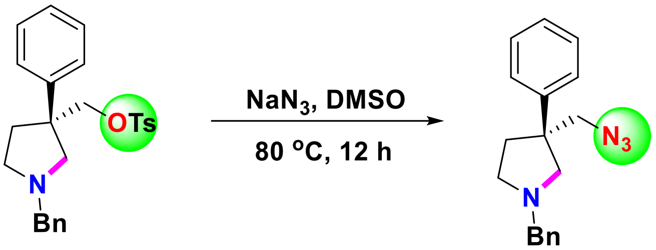

46 96

To a solution of **46** (42.2 mg, 0.1 mmol, 1.0 equiv) in DMSO (1 mL) was added NaN_3 (13 mg, 0.2 mmol, 2.0 equiv) at room temperature. The mixture was stirred at 80 °C for 12 h. Then, the mixture was diluted with EtOAc/water (1:1). The aqueous phase was extracted with EtOAc (two times). The combined organic phases were washed with water (two times) and brine, dried over $MgSO_4$, filtered and concentrated under reduced pressure. Final purification was accomplished by silica gel column chromatography (by using PE/EA = 20:1 to 5:1) to give the desired product **96** (23 mg, 78%) as a yellow oil. The enantioselectivity was determined by HPLC using a Daicel CHIRALCEL OJ-H column (25 cm × 0.46 cm ID), [hexane/iso-propanol (0.1% diethylamine) = 98:2, λ = 220 nm, 1.0 mL/min flow rate] t_R = 13.8 min (major) and t_{S} = 16.3 min (minor); **^{1}H NMR** (400 MHz, $CDCl_3$) δ 7.42 – 7.35 (m, 6H), 7.32 – 7.25 (m, 4H), 3.71 (d, *J* = 10.9 Hz, 3H), 3.56 (d, *J* = 11.6 Hz, 1H), 3.21 (d, *J* = 9.3 Hz, 1H), 3.05 (td, *J* = 8.6, 4.7 Hz, 1H), 2.66 (d, *J* = 9.3 Hz, 1H), 2.48 (dt, *J* = 12.8, 6.4 Hz, 1H), 2.30 – 2.13 (m, 2H). **^{13}C NMR** (100 MHz, $CDCl_3$) δ 145.4, 139.3, 128.5, 128.4, 128.3, 127.1, 127.0, 126.7, 62.0, 62.0, 60.1, 53.0, 50.7, 34.9. **HRMS** (ESI): calcd for $[M + H]^+$ $C_{18}H_{21}N_4$ 293.1775; found 293.1766.

Synthetic Natural Products Coerulescine[7]:

Benzyl (*R*)-3-(hydroxymethyl)-3-phenylpyrrolidine-1-carboxylate (97)

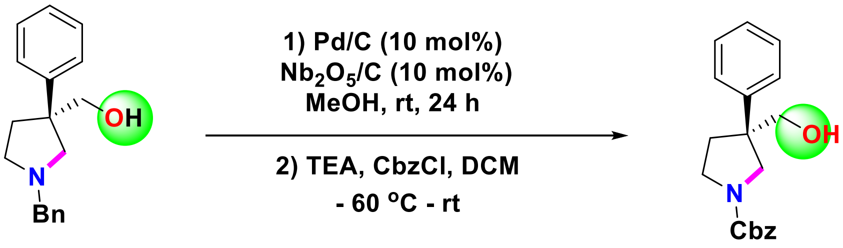

46 97

To a solution of **46** (106.8 mg, 0.4 mmol, 1.0 equiv) in MeOH (5mL) was added 10% Pd/C (42.6 mg, 0.04 mmol) and 10% Nb_2O_5/C (106.3 mg, 0.04 mmol) at room temperature[8]. The mixture was stirred at 25 °C for 16 h. Then, the mixture was filtered and concentrated under reduced pressure. The resulting crude product was used immediately in the next reaction without purification. To this mixture was added triethylamine (3 equiv), then cooled to -60 °C and benzyl chloroformate (1.2 equiv) was added dropwise. The reaction mixture was allowed to warm to room temp and stirred for 16 h. The solvent was removed by evaporation in vacuo and the resulting oil was purified by flash column chromatography to give the Cbz protected pyrrolidine **97** (98 mg,79%).

The enantioselectivity was determined by HPLC using a Daicel CHIRALCEL AD-H column (25 cm × 0.46 cm ID), [hexane/iso-propanol (0.1% diethylamine) = 98:2, λ = 220 nm, 1.0 mL/min flow rate] t_R =

17.8 min (major) and t_{S} = 18.7 min (minor); **^{1}H NMR** (400 MHz, $CDCl_3$) δ 7.45 – 7.30 (m, 8H), 7.25 (t, *J* = 7.4 Hz, 2H), 5.27 – 5.14 (m, 2H), 3.96 (t, *J* = 10.2 Hz, 1H), 3.73 – 3.53 (m, 5H), 2.55 – 2.34 (m, 1H), 2.15 (dq, *J* = 19.3, 9.5 Hz, 1H), 1.68 (s, 1H). **^{13}C NMR** (100 MHz, $CDCl_3$) δ 155.0, 142.9, 136.9, 128.7, 128.5, 128.0, 127.9, 127.1, 126.9, 126.8*, 68.3, 68.2*, 67.0, 66.9*, 53.1, 52.5*, 52.0, 51.1*, 44.8, 44.5*, 32.1, 31.5*(Signal for the rotational isomer denoted with asterisk). **HRMS** (ESI): calcd for $[M + H]^+$ $C_{19}H_{21}NO_3Na$ 334.1419; found 334.1418.

Benzyl (*R*)-3-formyl-3-phenylpyrrolidine-1-carboxylate (98)

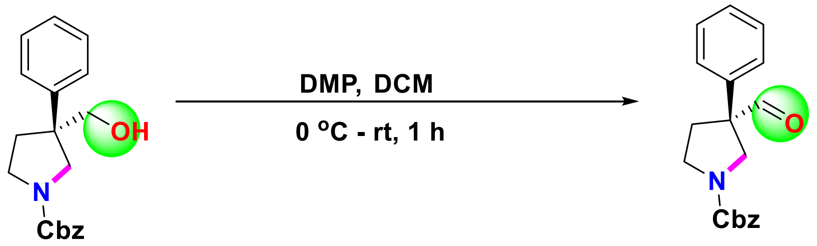

97
79% yield, 94:6 e.r

98
65% yield, 96:4 e.r

To a solution of pyrrolidine **97** (62.2 mg, 0.2 mmol, 1.0 equiv) in DCM (2 mL) was added Dess-Martin periodinane (85.6 mg, 0.2 mmol) at 0 °C. The solution was stirred 1 h at room temperature and concentrated in vacuo. The mixture was filtered and concentrated under reduced pressure. The residue was purified by flash chromatography to afford the desired product **98** (40 mg, 65%).

The enantioselectivity was determined by HPLC using a Daicel CHIRALCEL OD-H column (25 cm × 0.46 cm ID), [hexane/iso-propanol (0.1% diethylamine) = 90:10, λ = 220 nm, 1.0 mL/min flow rate] t_{S} = 17.8 min (minor) and t_{R} = 19.6 min (major); **^{1}H NMR** (400 MHz, CDCl3) δ 9.47 (d, *J* = 6.2 Hz, 1H), 7.47 – 7.32 (m, 8H), 7.22 (t, *J* = 7.5 Hz, 2H), 5.30 – 5.09 (m, 2H), 4.57 – 4.35 (m, 1H), 3.75 – 3.55 (m, 2H), 3.48 – 3.35 (m, 1H), 2.83 (ddd, *J* = 28.2, 12.5, 6.8 Hz, 1H), 2.32 – 2.13 (m, 1H). **^{13}C NMR** (100 MHz, $CDCl_3$) δ 197.8, 197.6*, 154.7, 154.6*, 136.7, 136.1*, 129.3, 128.5, 128.2, 128.0, 127.8*, 127.2, 127.2*, 67.1, 67.0*, 61.9, 61.1*, 50.7, 50.1*, 44.9, 44.7*, 30.8, 30.0* (Signal for the rotational isomer denoted with asterisk). **HRMS** (ESI): calcd for $[M + H]^+$ $C_{19}H_{19}NO_3Na$ 332.1263; found 332.1271.

3.4 NMR spectra of the relevant compounds

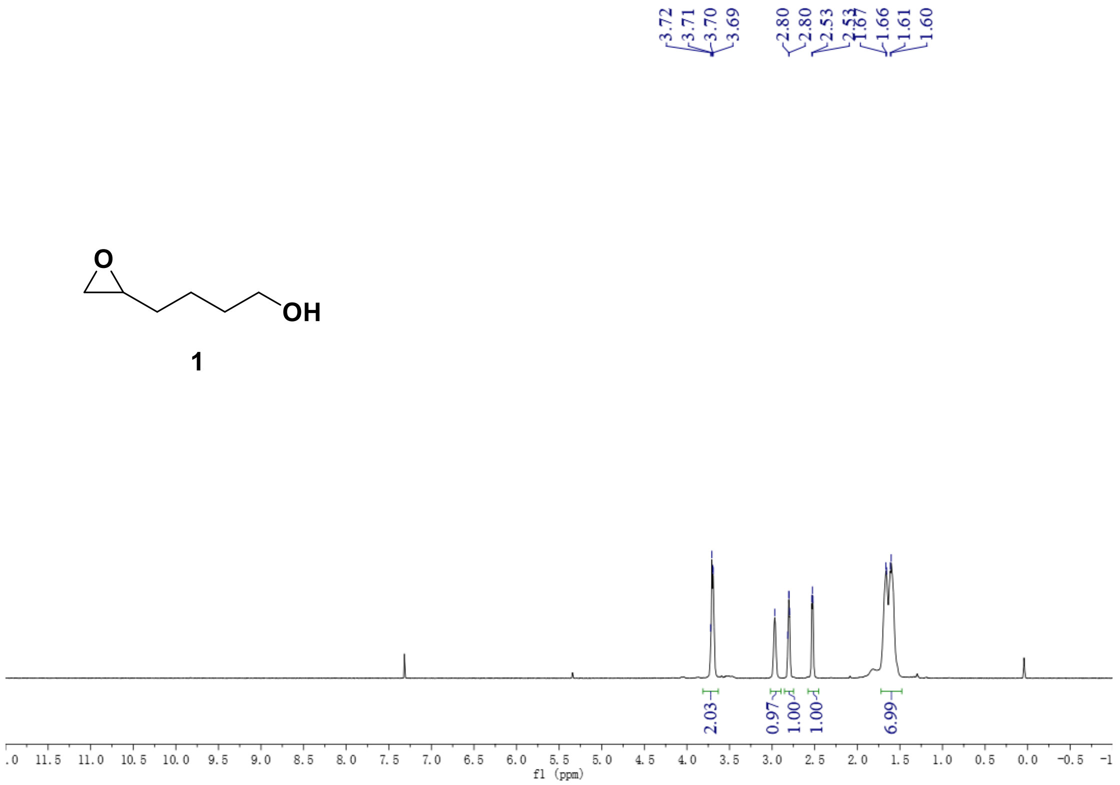

Supplementary Figure 9. ^{1}H NMR (400 MHz, $CDCl_3$) spectrum of **1.**

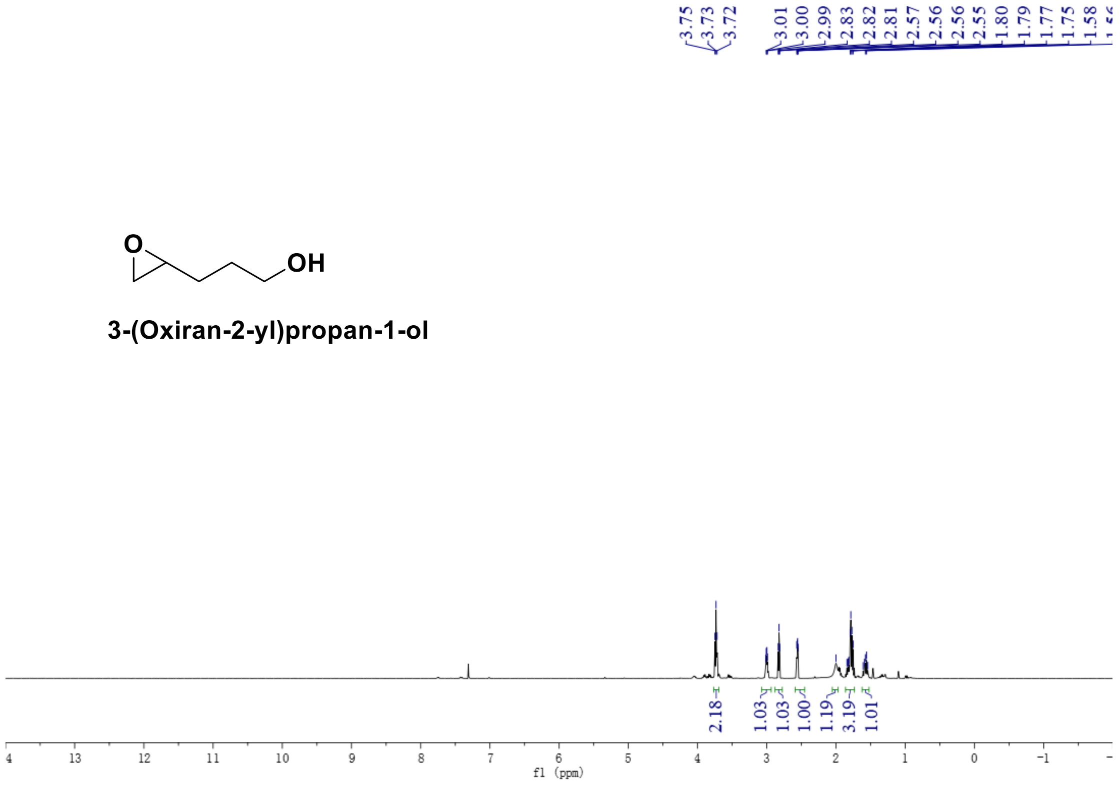

Supplementary Figure 10. ^{1}H NMR (400 MHz, $CDCl_3$) spectrum of **3-(Oxiran-2-yl)propan-1-ol**

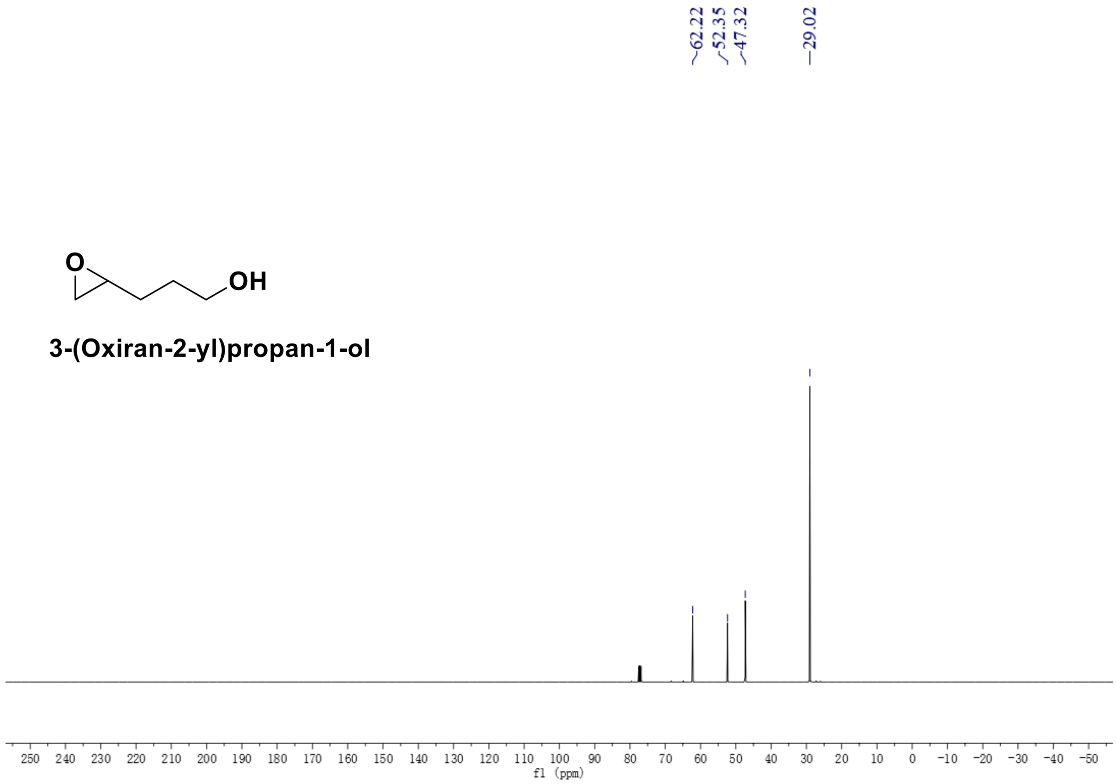

Supplementary Figure 11. ^{13}**C NMR (101 MHz,** $CDCl_3$**)** spectrum of **3-(Oxiran-2-yl)propan-1-ol**

.

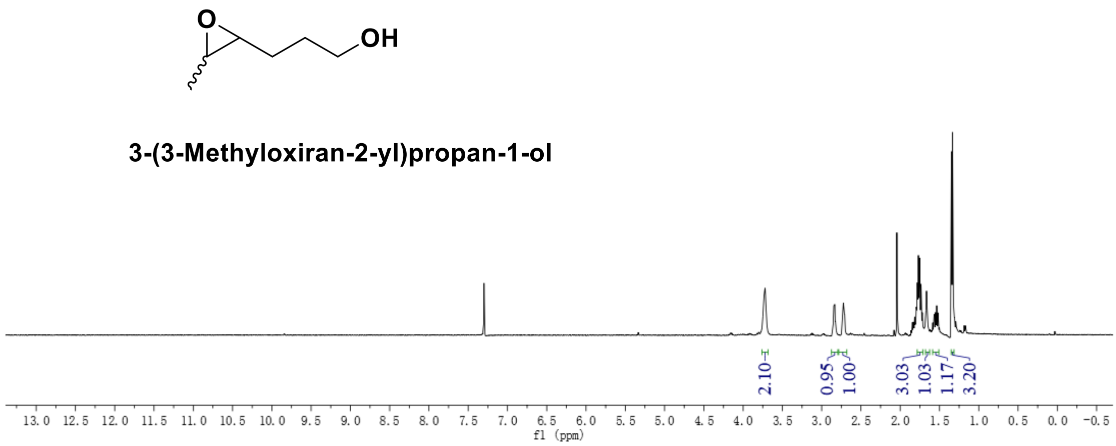

Supplementary Figure 12. ^{1}**H NMR (400 MHz,** $CDCl_3$**)** spectrum of **3-(3-Methyloxiran-2-yl)propan-1-ol.**

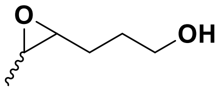

3-(3-Methyloxiran-2-yl)propan-1-ol

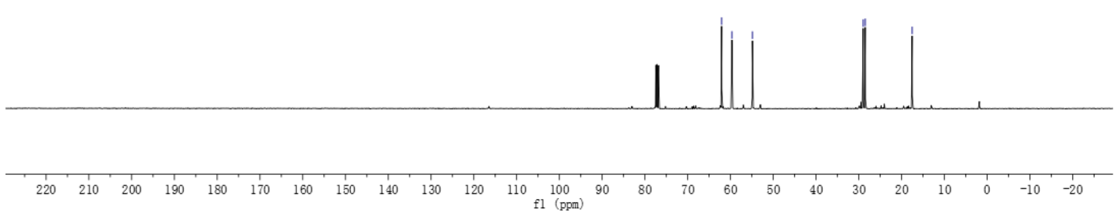

Supplementary Figure 13. ^{13}**C NMR (101 MHz,** $CDCl_3$**)** spectrum of **3-(3-Methyloxiran-2-yl)propan-1-ol.**

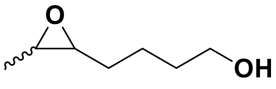

5

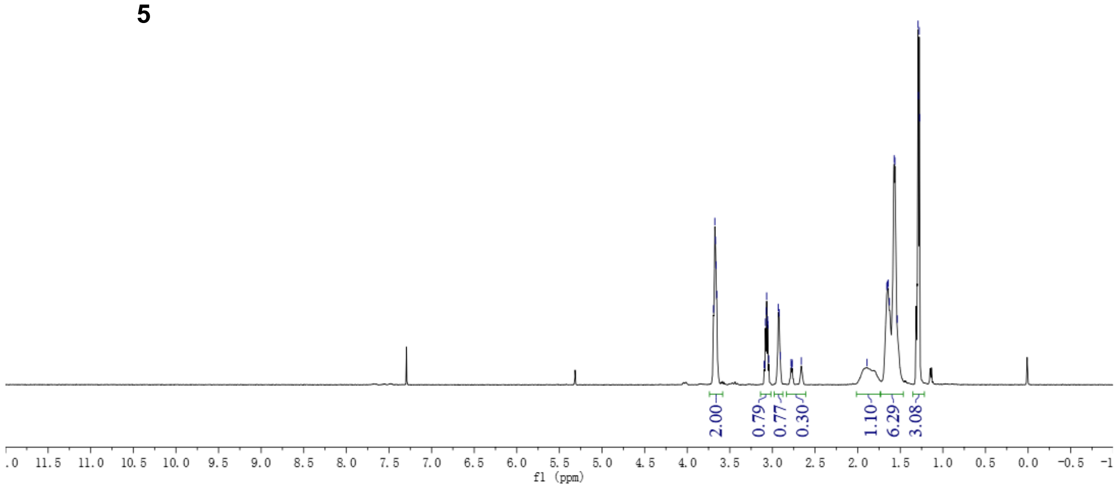

Supplementary Figure 14. ^{1}**H NMR (400 MHz,** $CDCl_3$**)** spectrum of **5.**

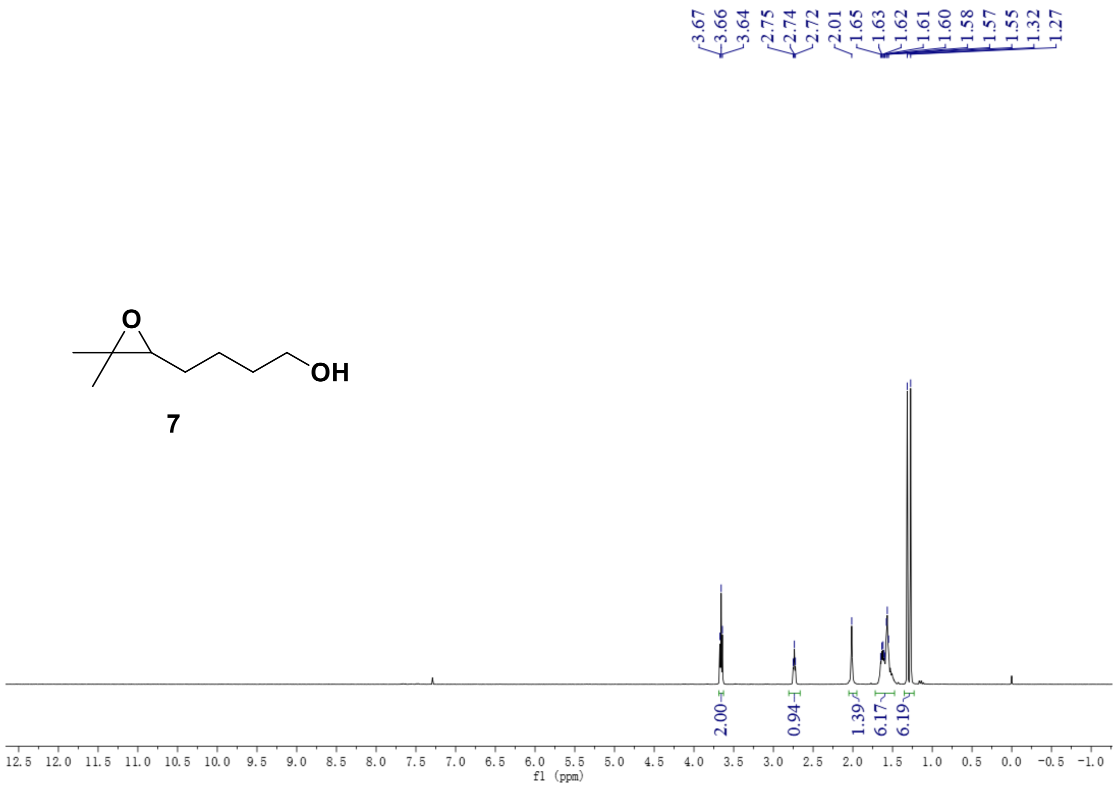

Supplementary Figure 15. **^{1}H NMR (400 MHz, $CDCl_3$)** spectrum of **7.**

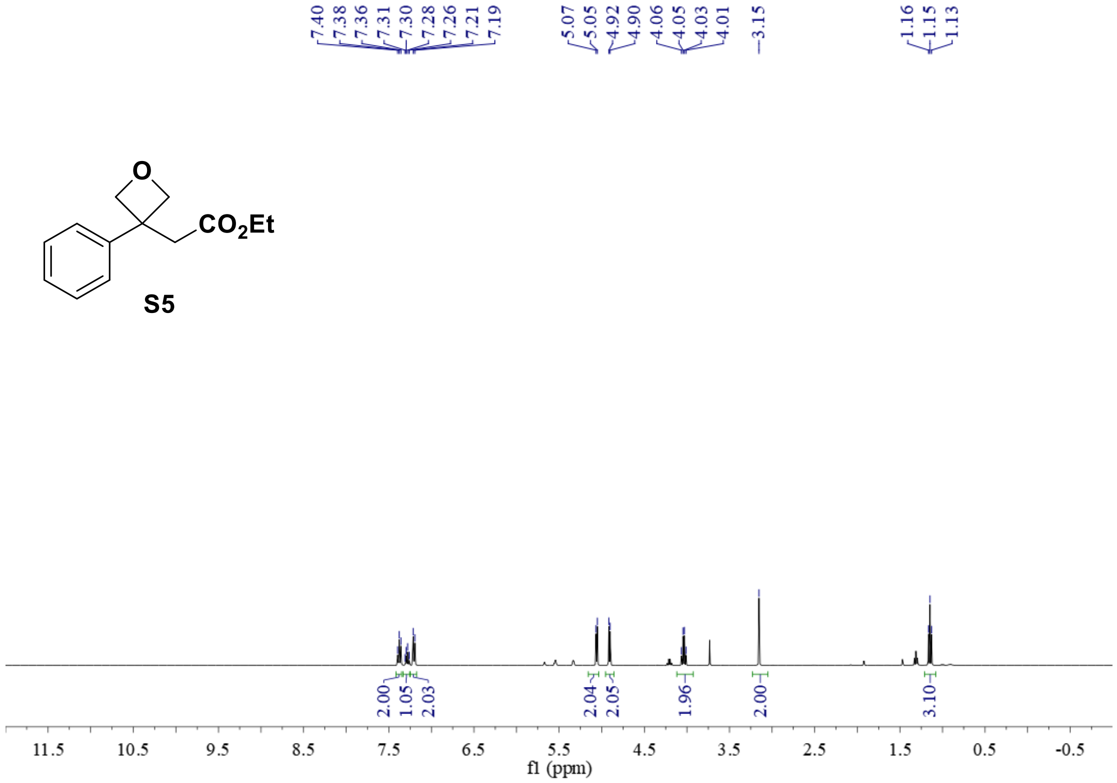

Supplementary Figure 16. **^{1}H NMR (400 MHz, $CDCl_3$)** spectrum of **S5.**

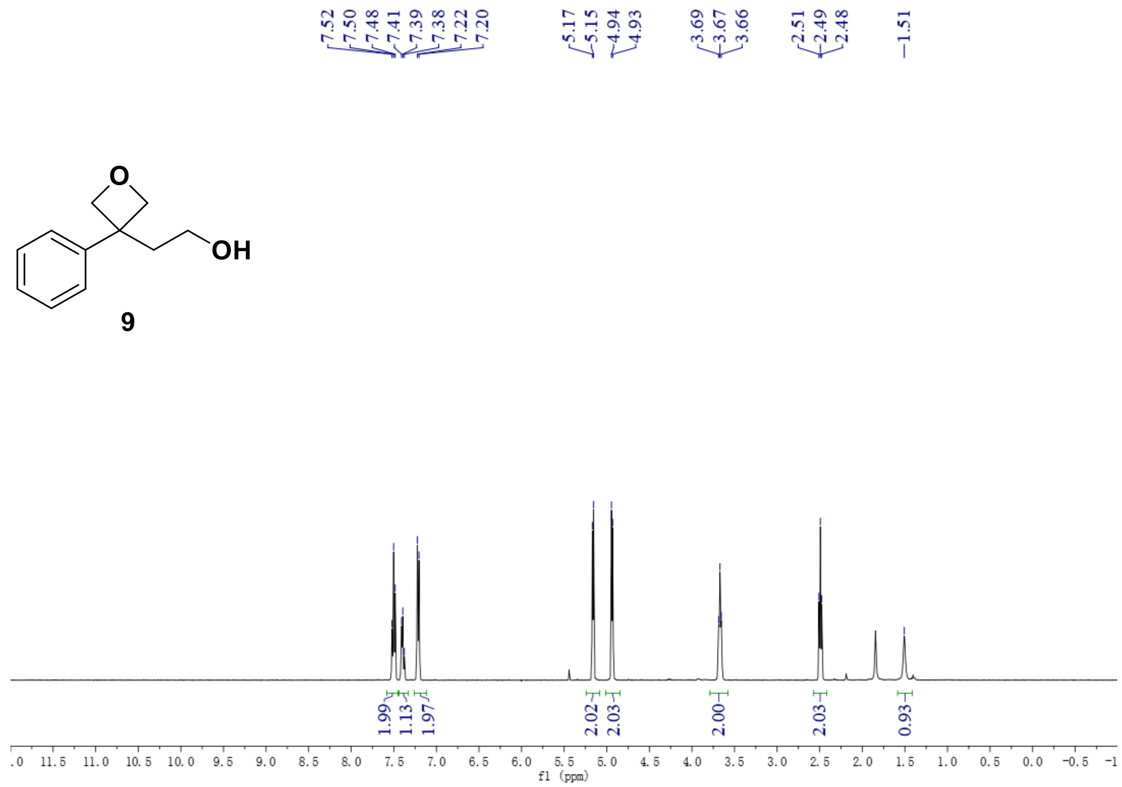

Supplementary Figure 17.^{1}**H NMR (400 MHz, CDCl$_3$)** spectrum of **9.**

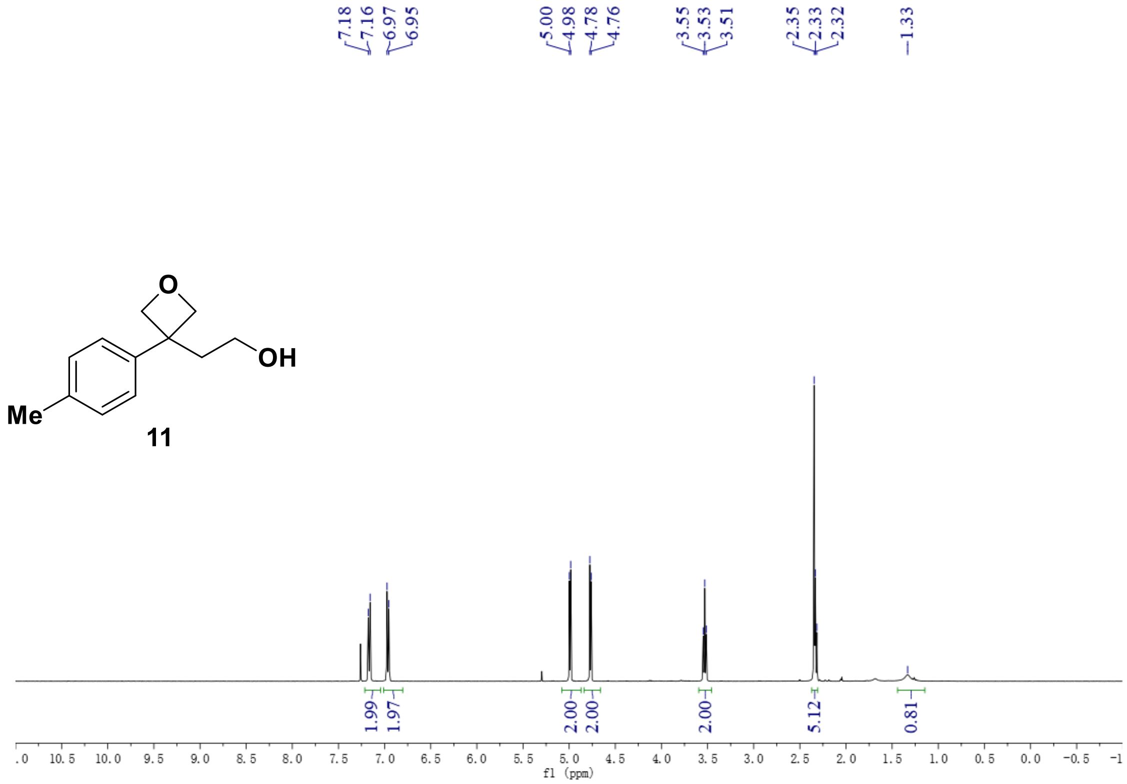

Supplementary Figure 18. ^{1}**H NMR (400 MHz, CDCl$_3$)** spectrum of **11.**

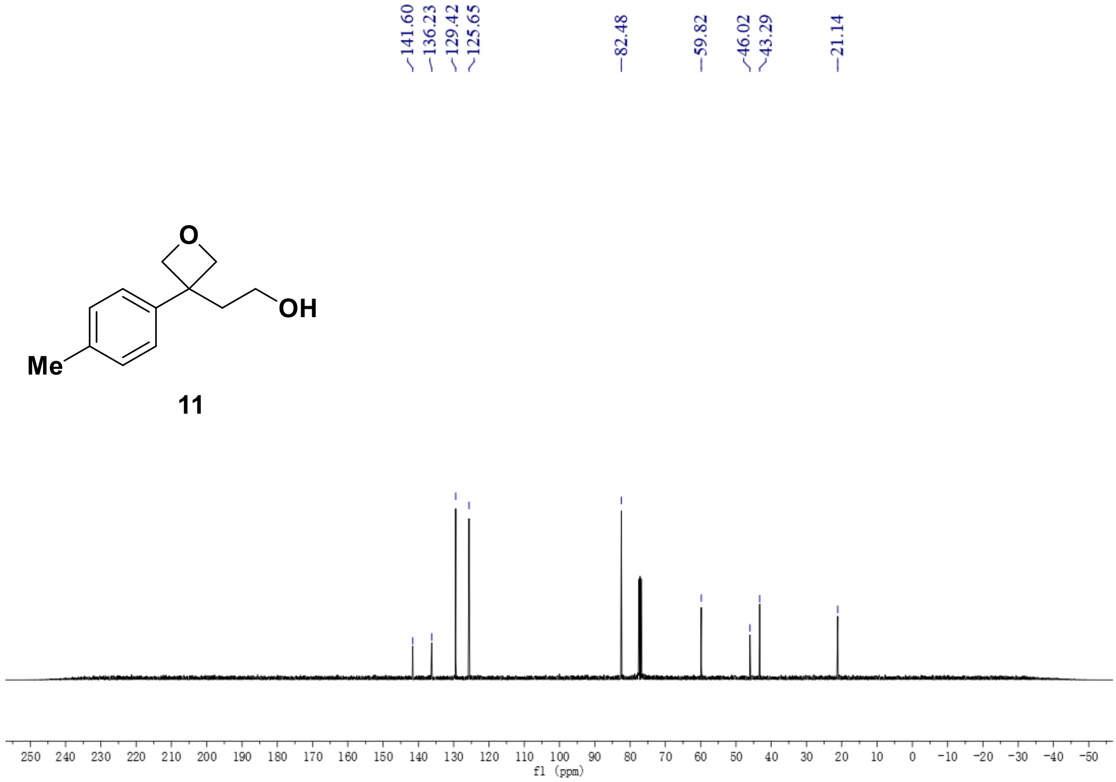

Supplementary Figure 19. ^{13}C NMR (101 MHz, CDCl$_3$) spectrum of **11.**

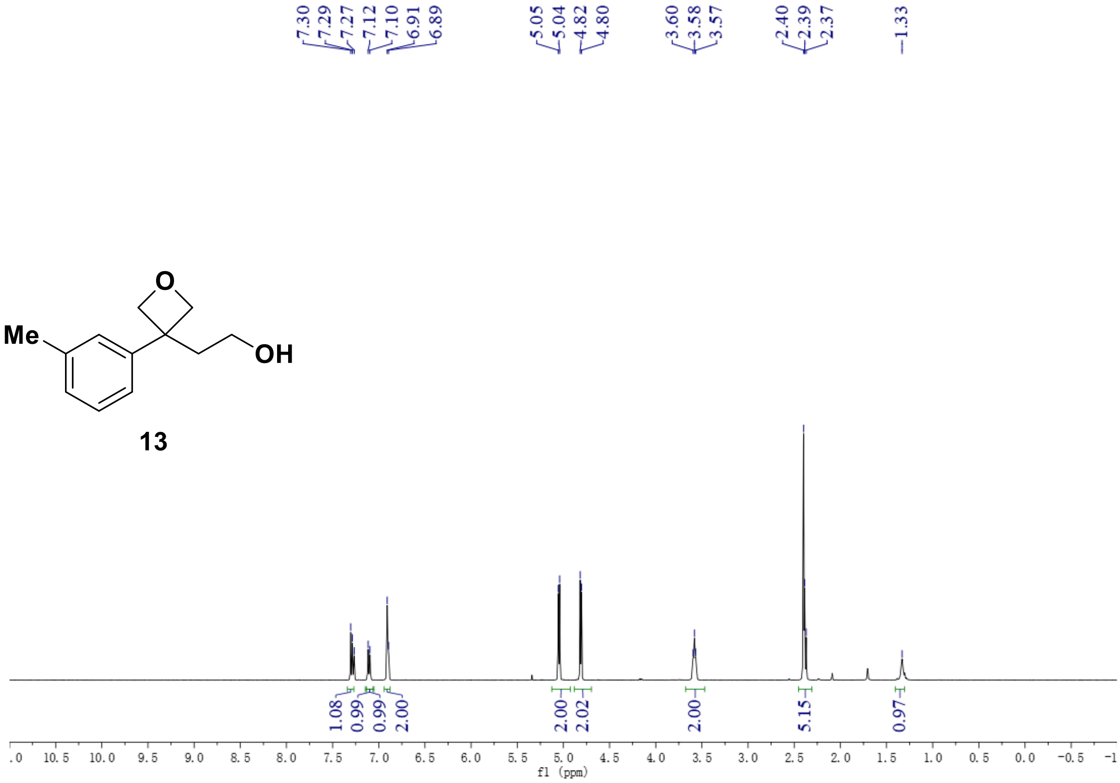

Supplementary Figure 20.^{1}H NMR (400 MHz, CDCl$_3$) spectrum of **13.**

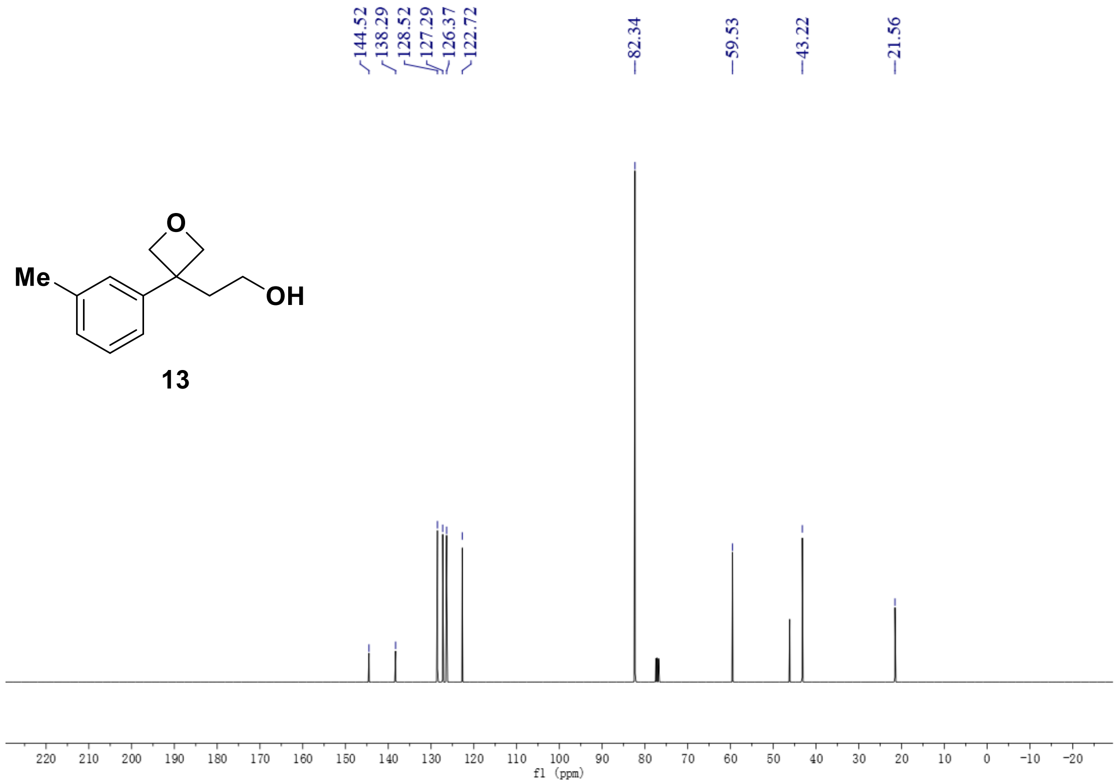

Supplementary Figure 21. ^{13}C NMR (101 MHz, $CDCl_3$) spectrum of **13.**

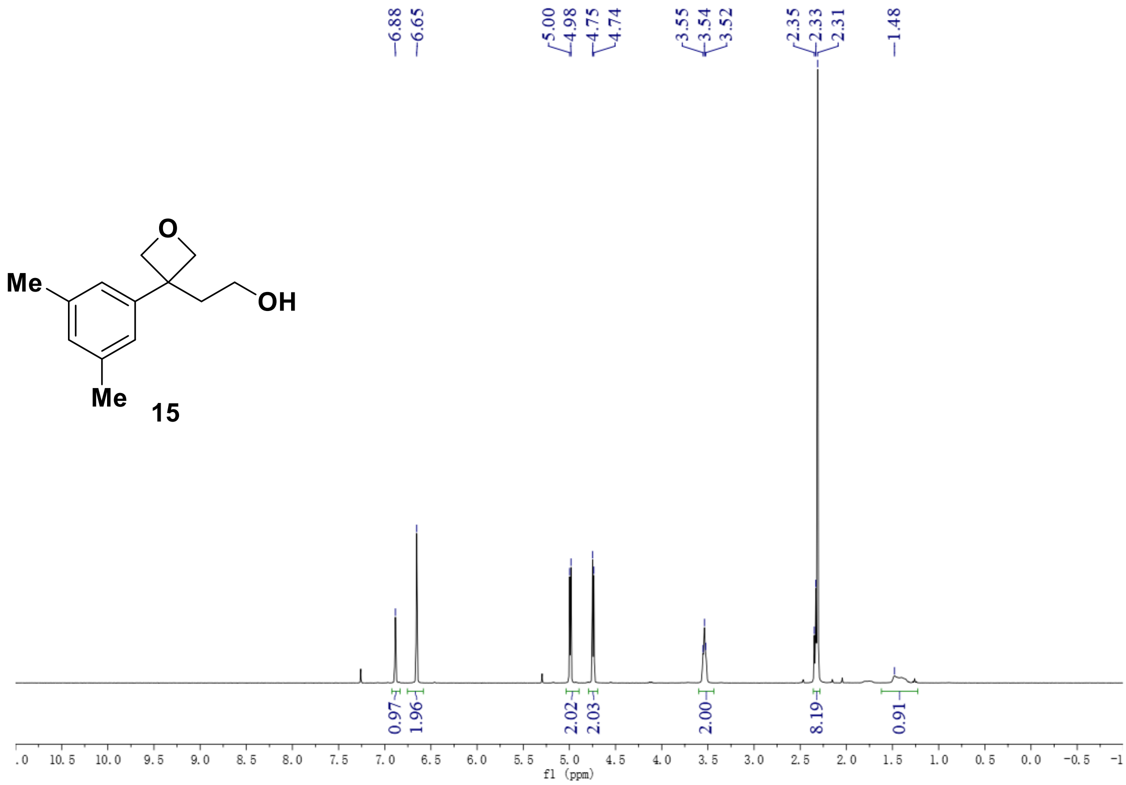

Supplementary Figure 22. ^{1}H NMR (400 MHz, $CDCl_3$) spectrum of **15.**

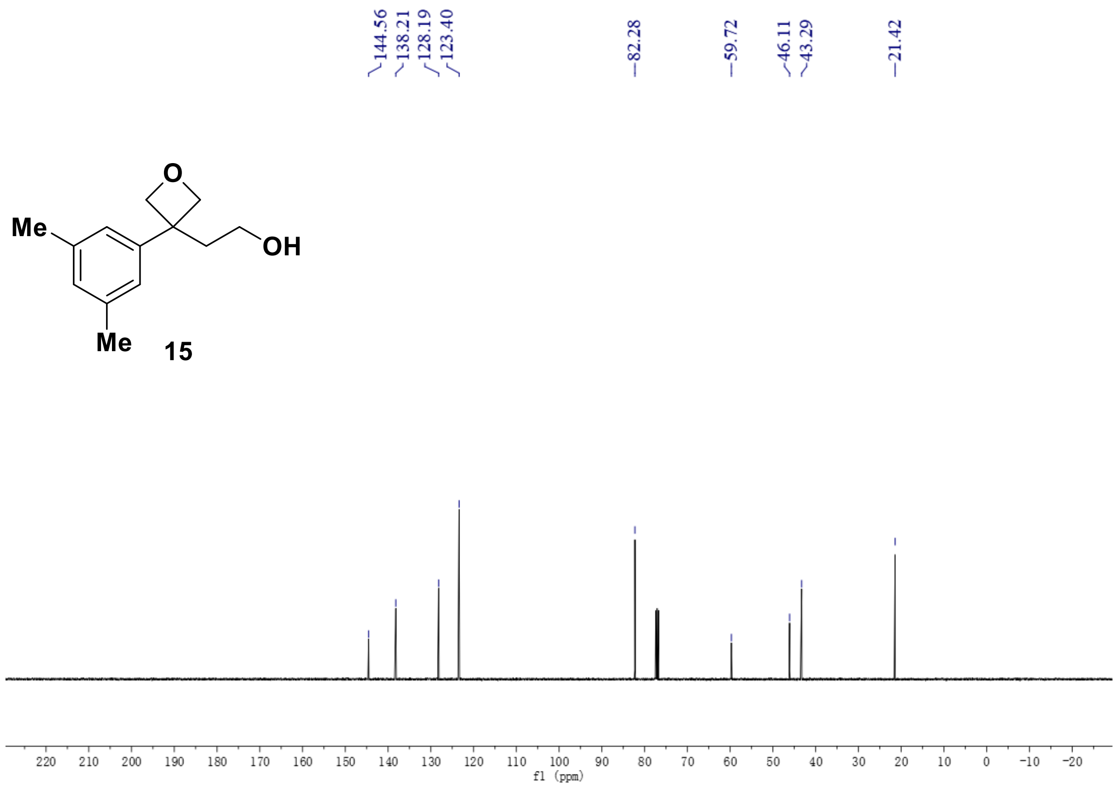

Supplementary Figure 23. ^{13}**C NMR (101 MHz, CDCl$_3$)** spectrum of **15.**

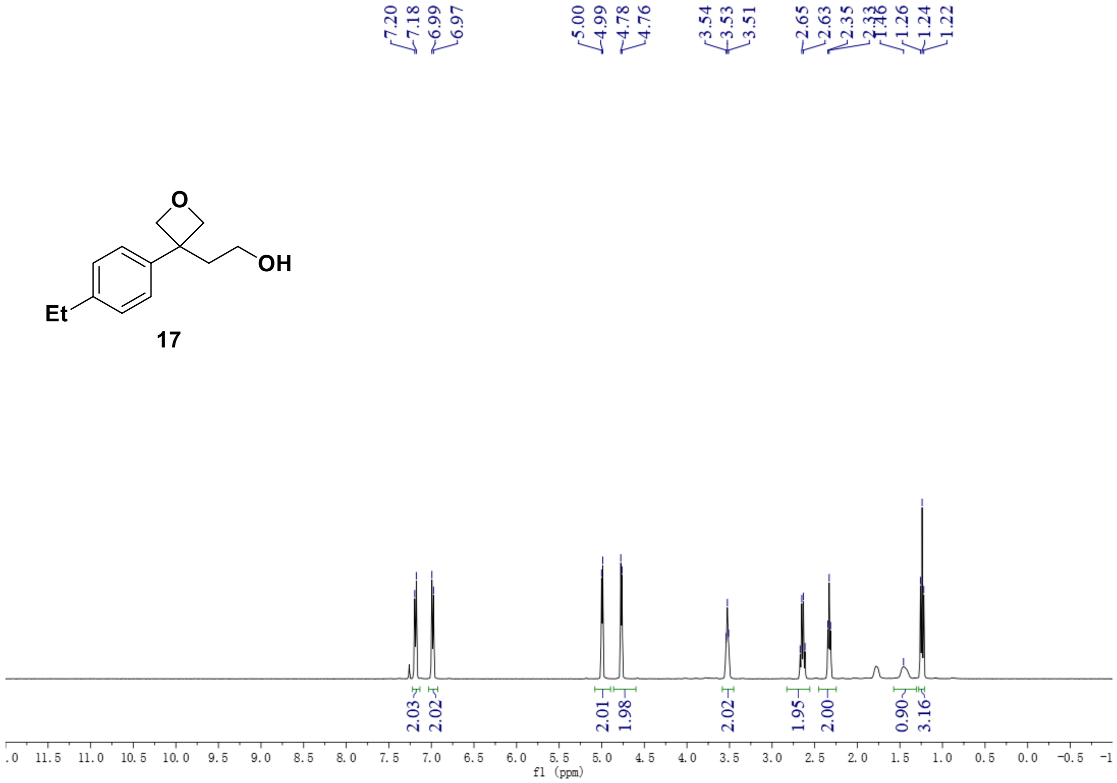

Supplementary Figure 24. ^{1}**H NMR (400 MHz, CDCl$_3$)** spectrum of **17.**

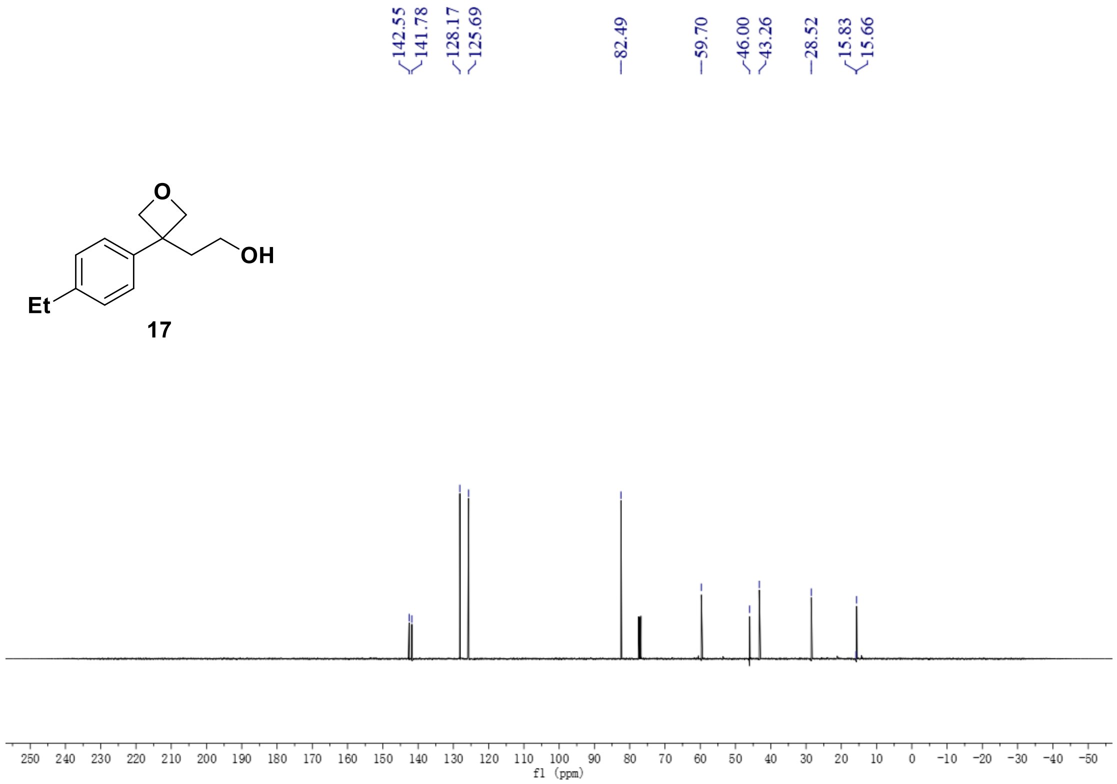

Supplementary Figure 25. ^{13}**C NMR (101 MHz, CDCl$_3$)** spectrum of **17.**

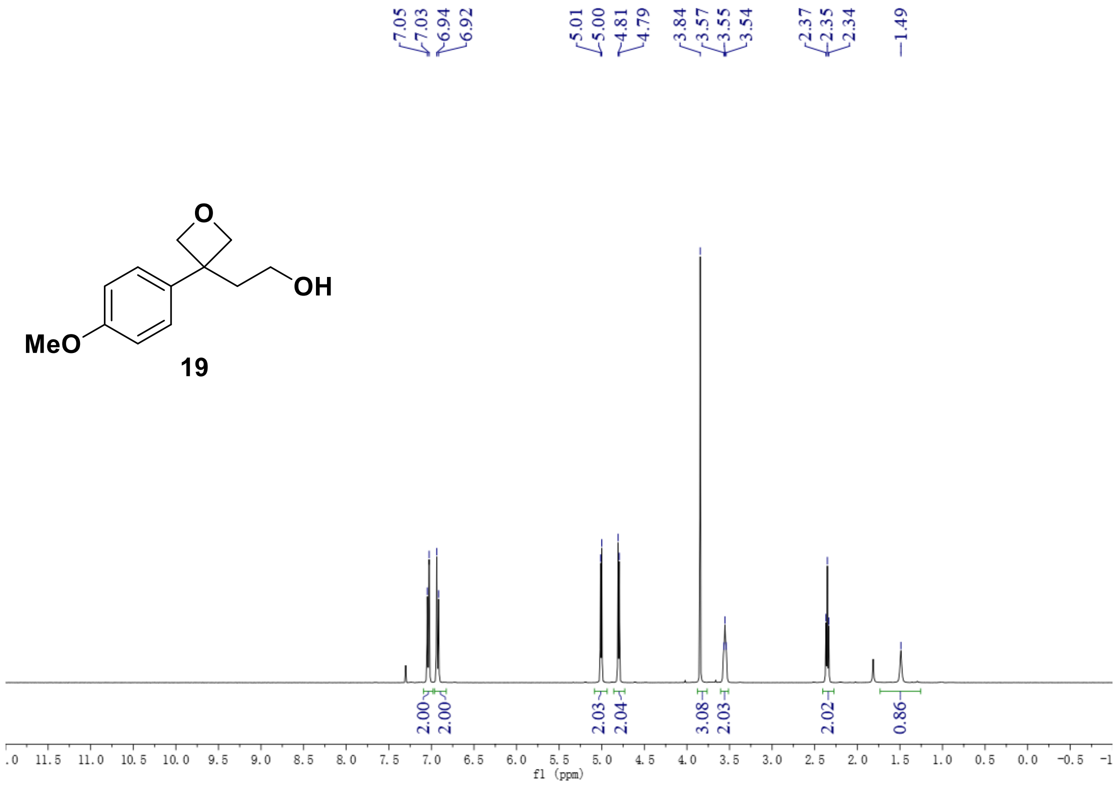

Supplementary Figure 26. ^{1}**H NMR (400 MHz, CDCl$_3$)** spectrum of **19.**

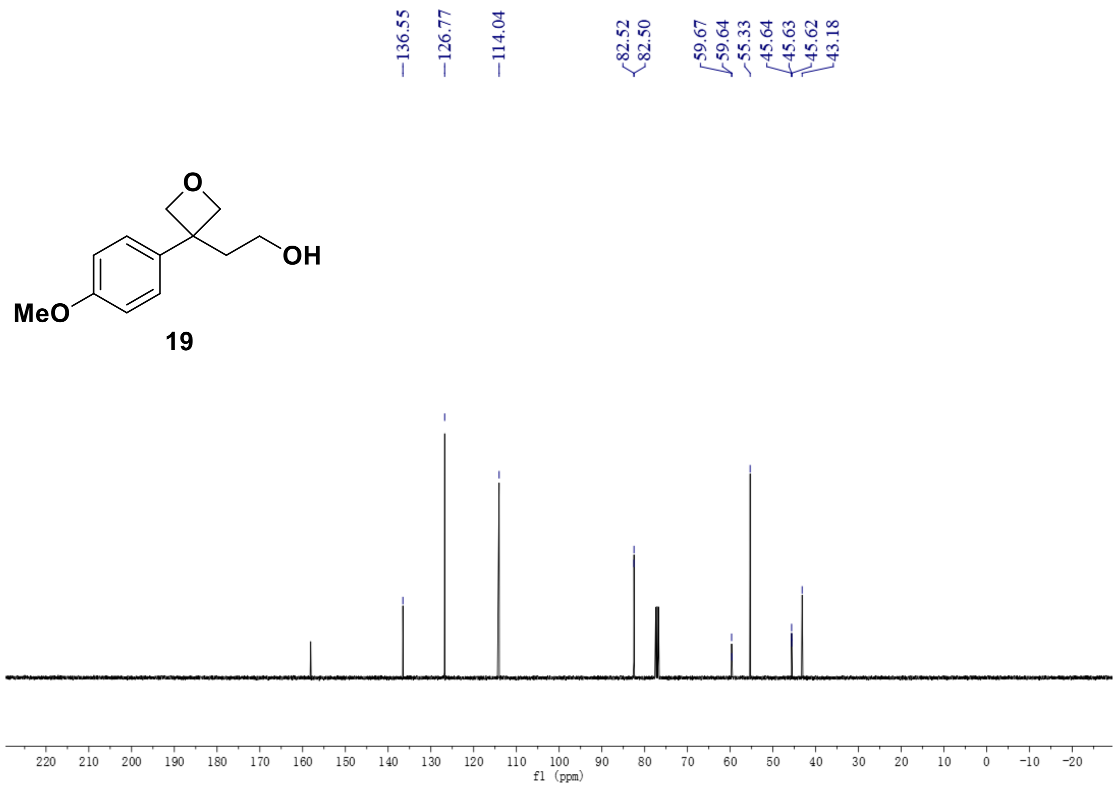

Supplementary Figure 27. ^{13}**C NMR (101 MHz, CDCl$_3$)** spectrum of **19.**

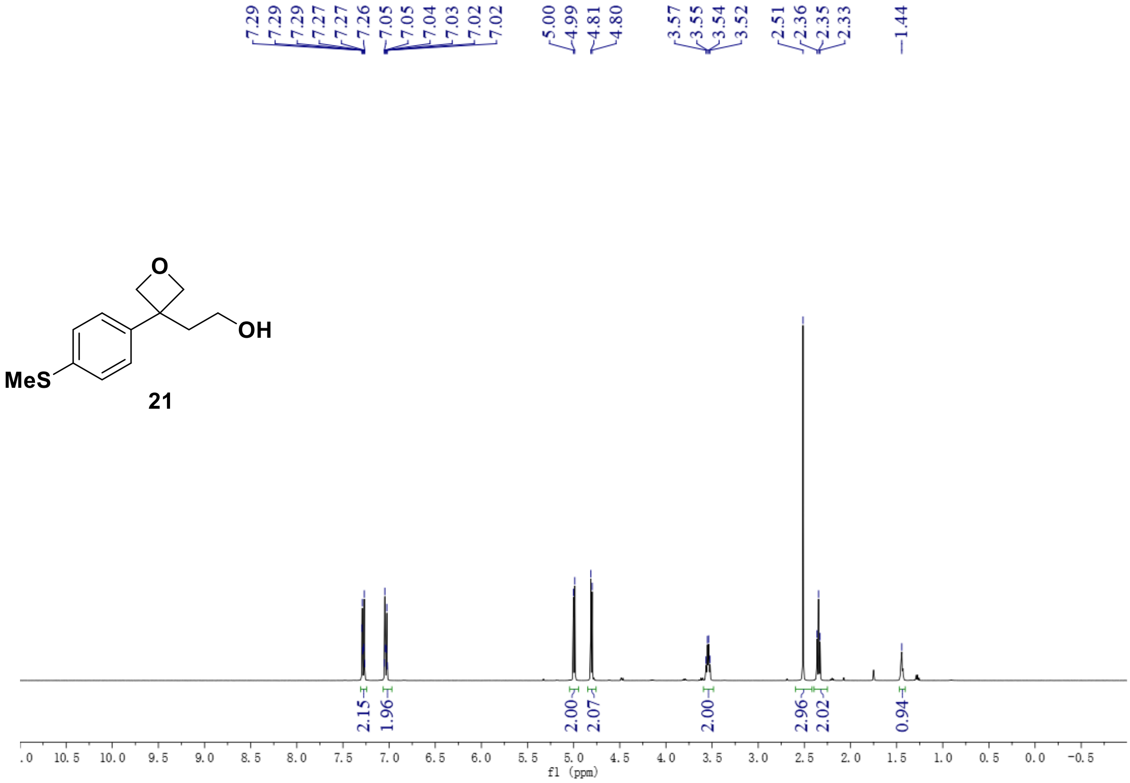

Supplementary Figure 28. ^{1}**H NMR (400 MHz, CDCl$_3$)** spectrum of **21.**

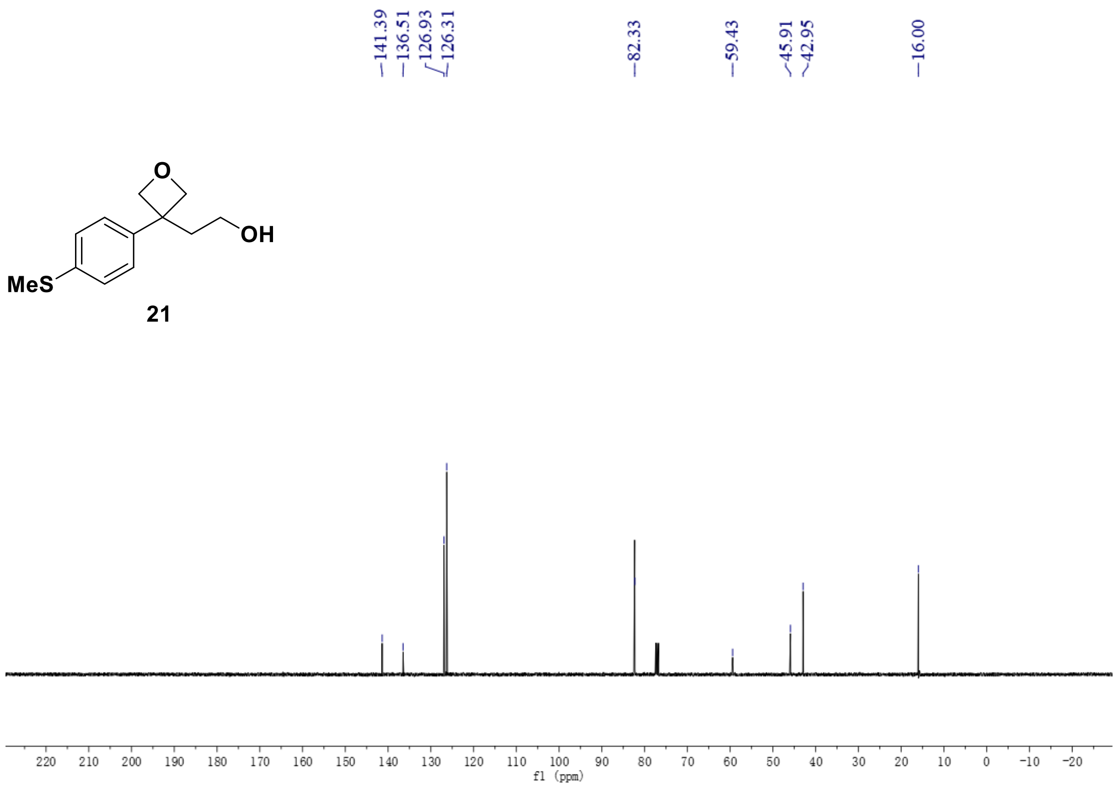

Supplementary Figure 29. ^{13}**C NMR (101 MHz, CDCl$_3$)** spectrum of **21.**

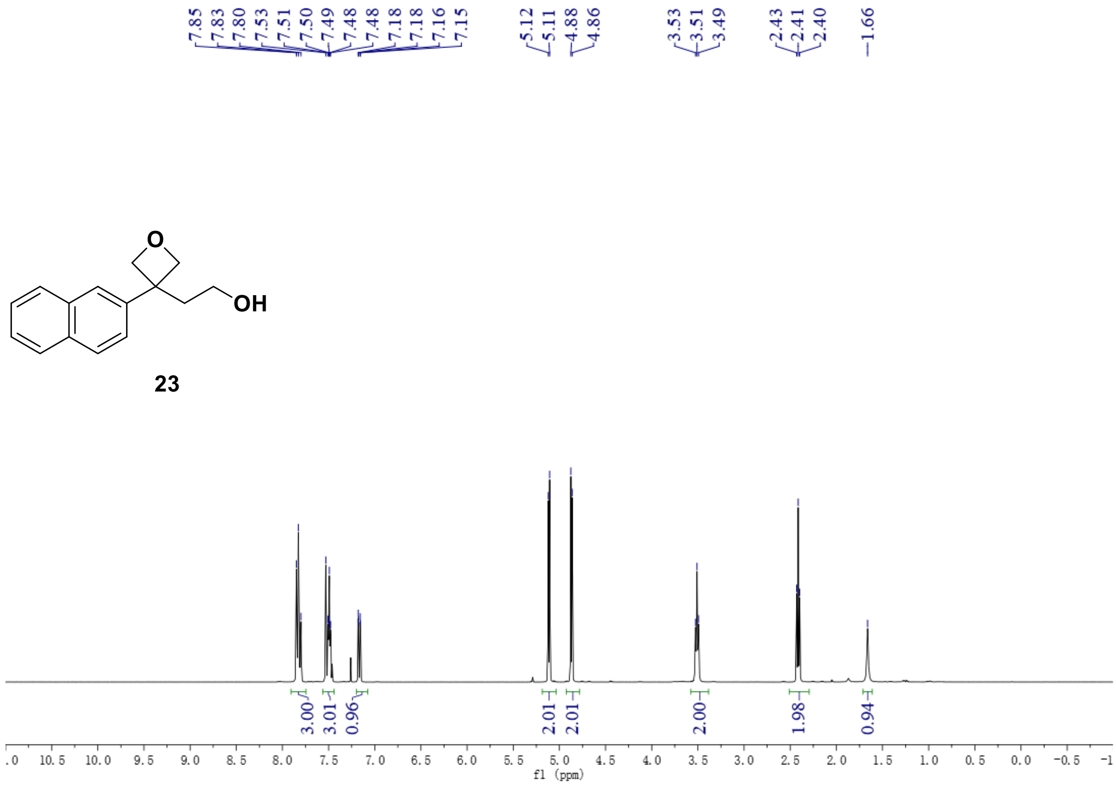

Supplementary Figure 30. ^{1}**H NMR (400 MHz, CDCl$_3$)** spectrum of **23.**

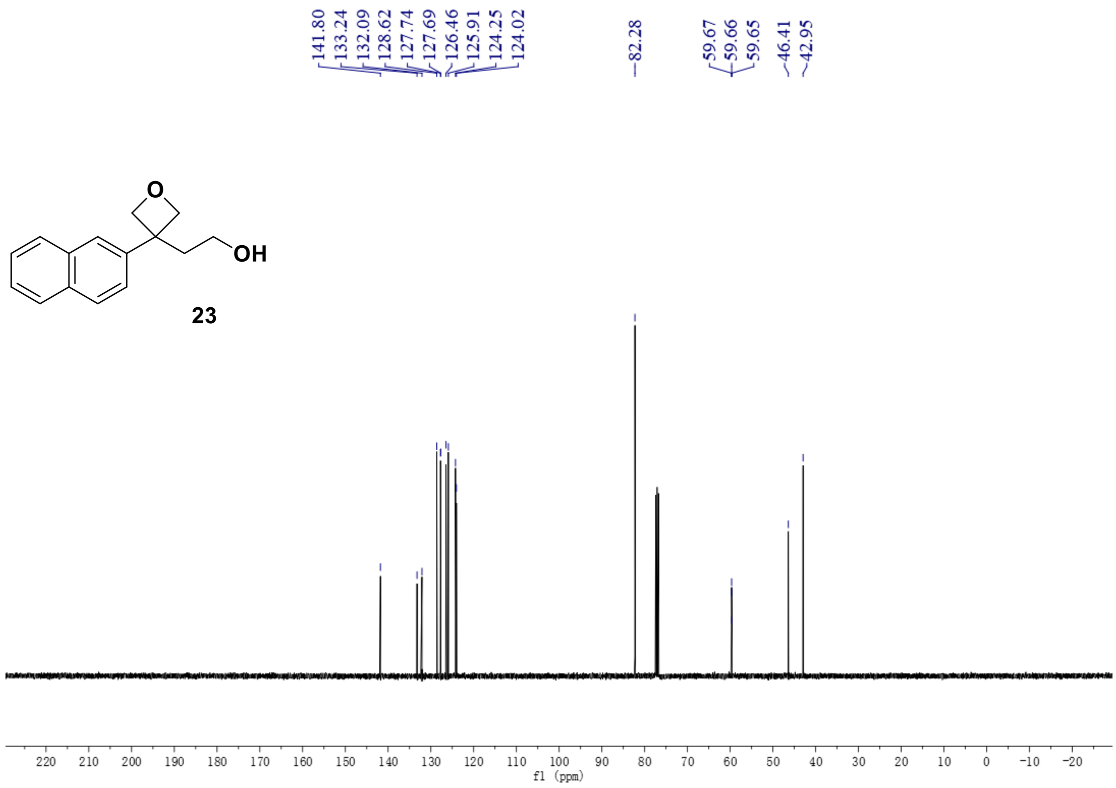

Supplementary Figure 31. ^{13}**C NMR (101 MHz, CDCl$_3$)** spectrum of **23.**

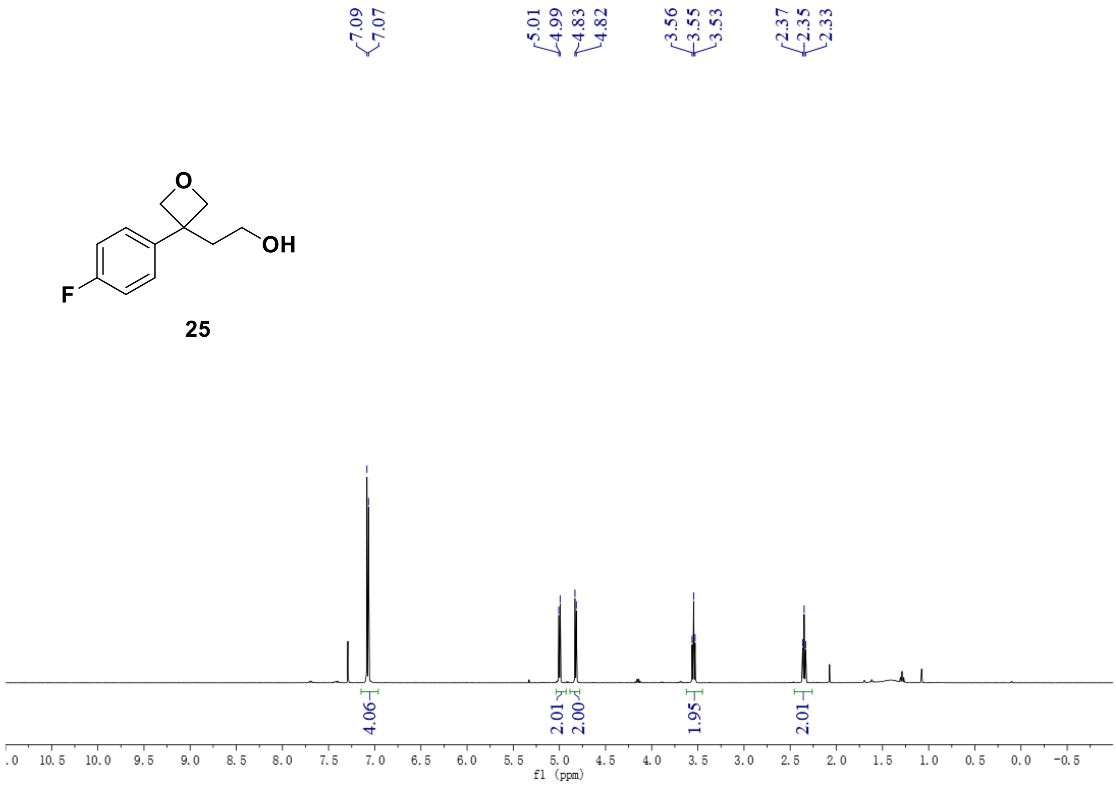

Supplementary Figure 32. ^{1}**H NMR (400 MHz, CDCl$_3$)** spectrum of **25.**

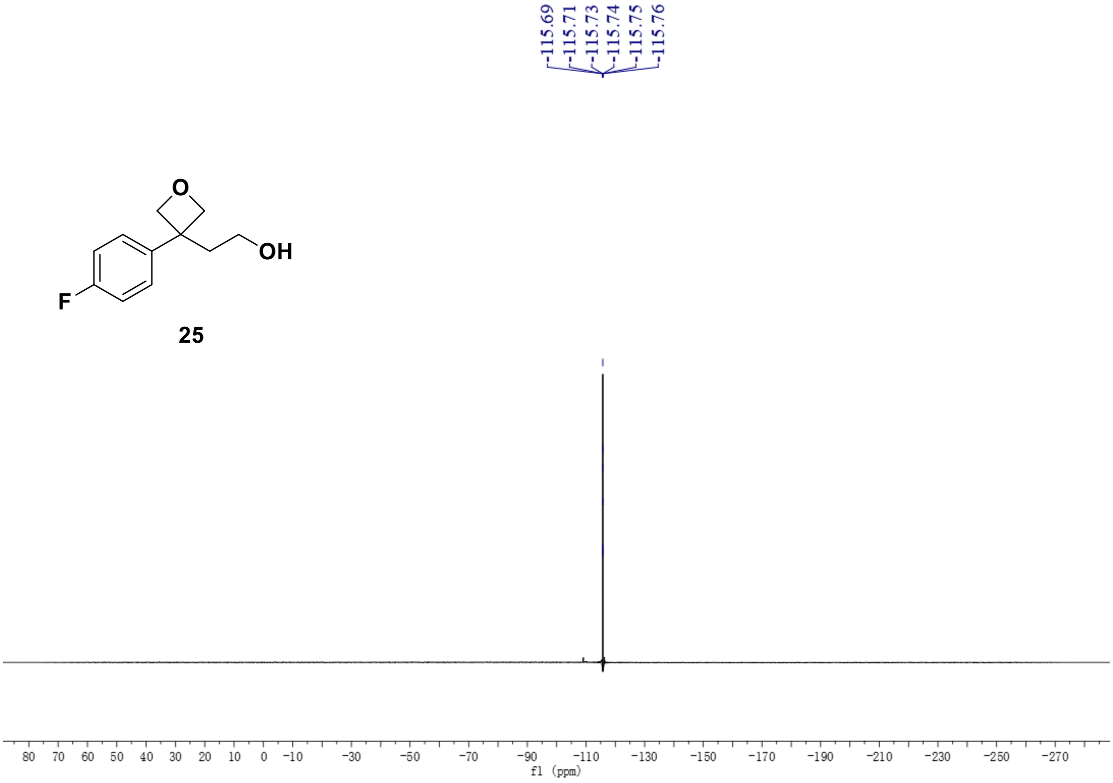

Supplementary Figure 33. ^{19}**F NMR (376 MHz,** $CDCl_3$**)** spectrum of **25.**

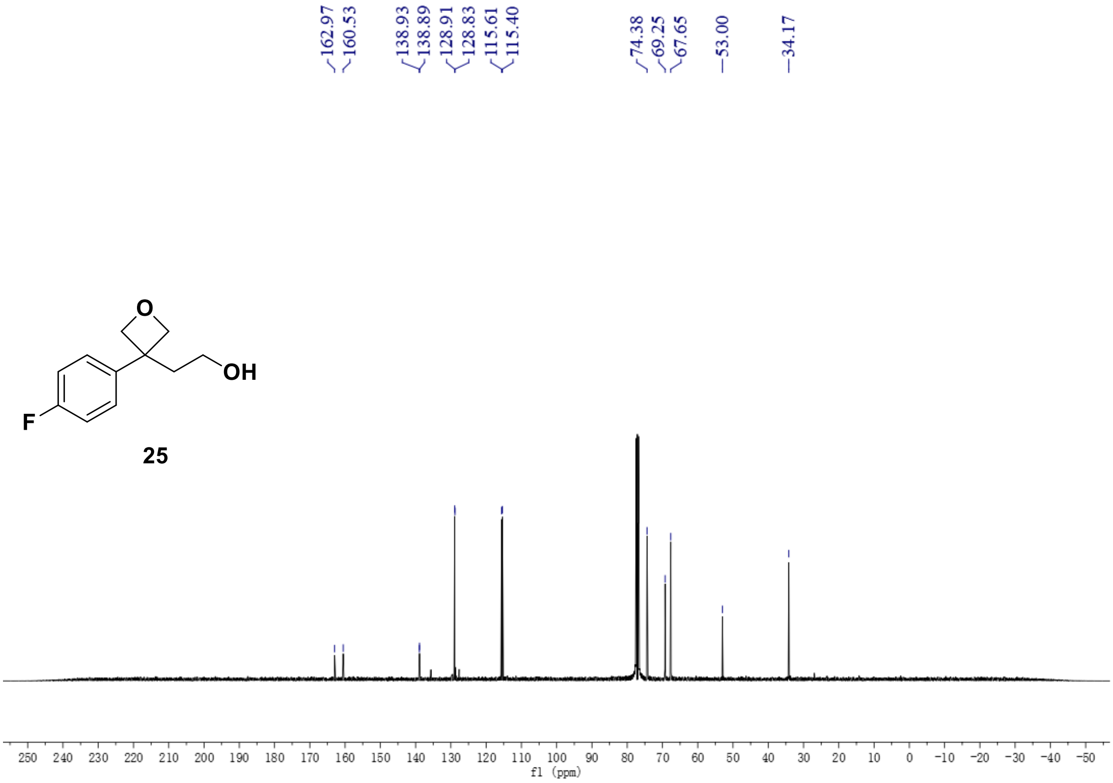

Supplementary Figure 34. ^{13}**C NMR (101 MHz,** $CDCl_3$**)** spectrum of **25.**

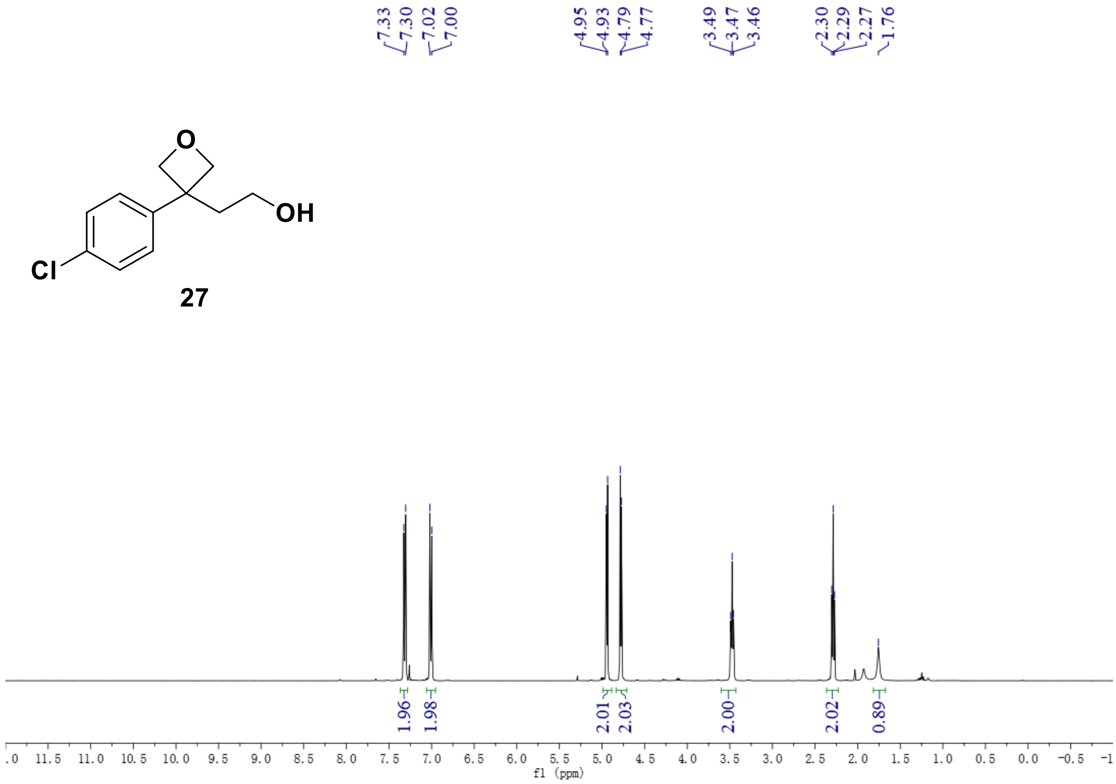

Supplementary Figure 35. ^{1}H NMR (400 MHz, CDCl$_3$) spectrum of **27.**

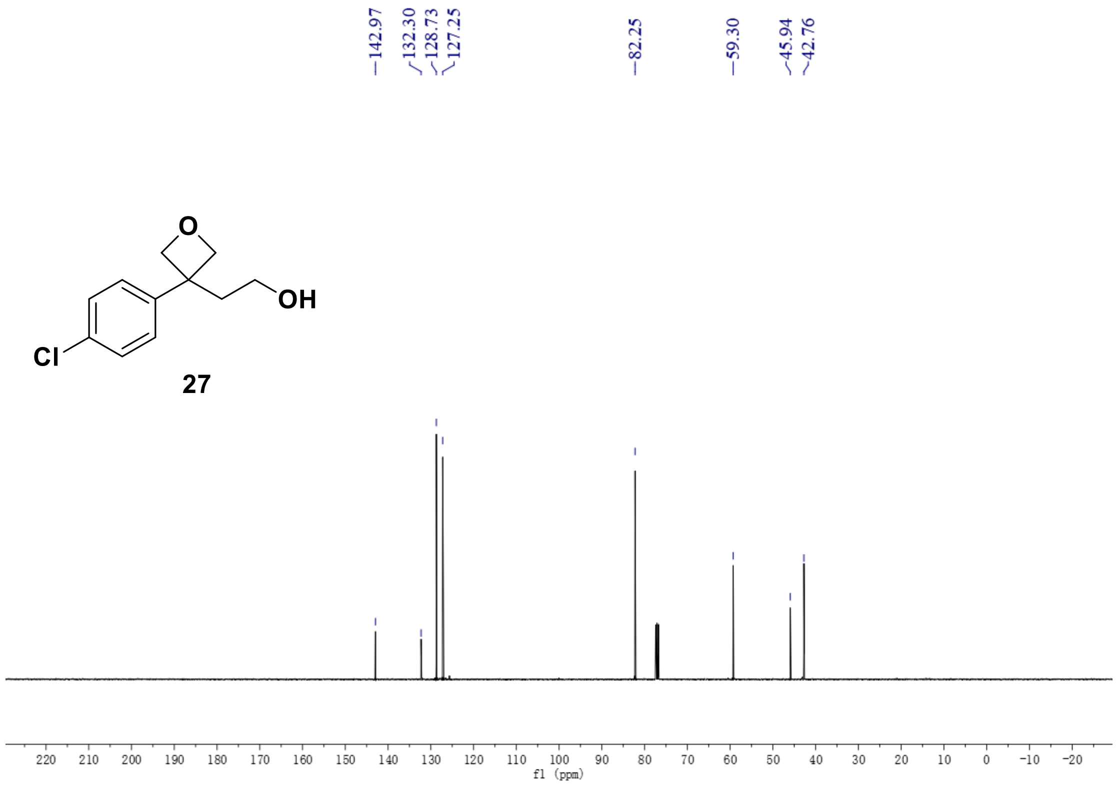

Supplementary Figure 36. ^{13}C NMR (101 MHz, CDCl$_3$) spectrum of **27.**

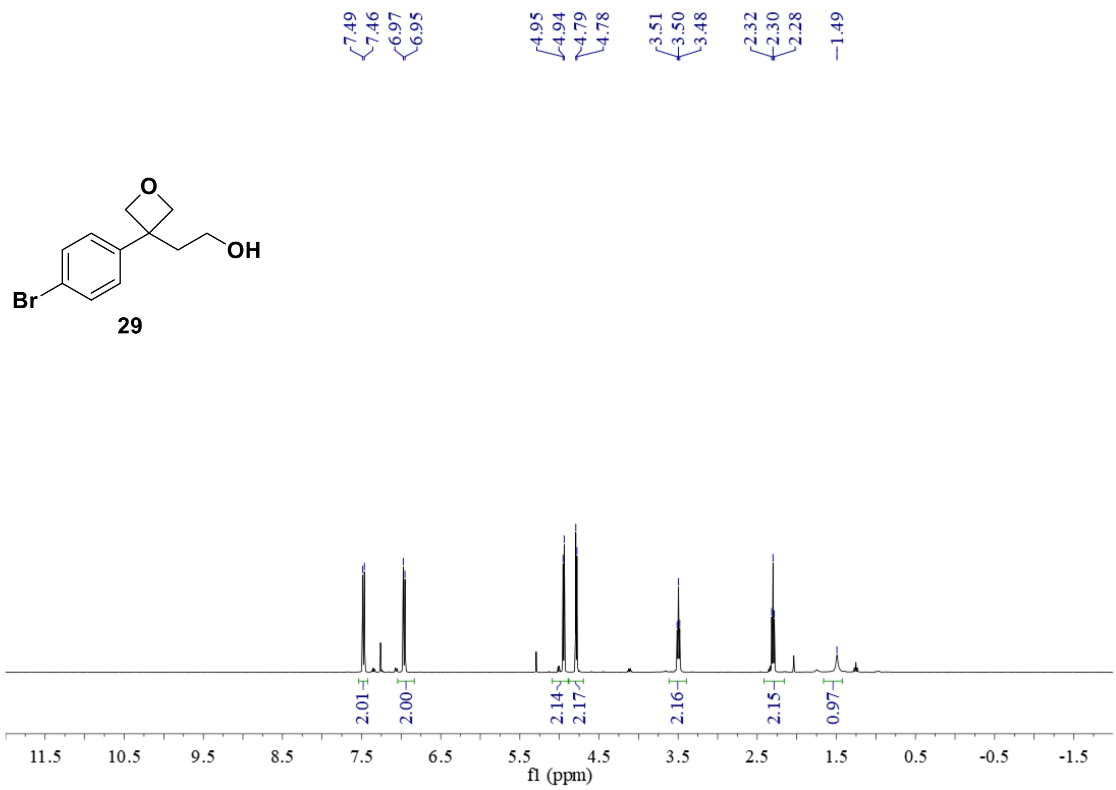

Supplementary Figure 37. ^{1}H NMR (400 MHz, CDCl$_3$) spectrum of 29.

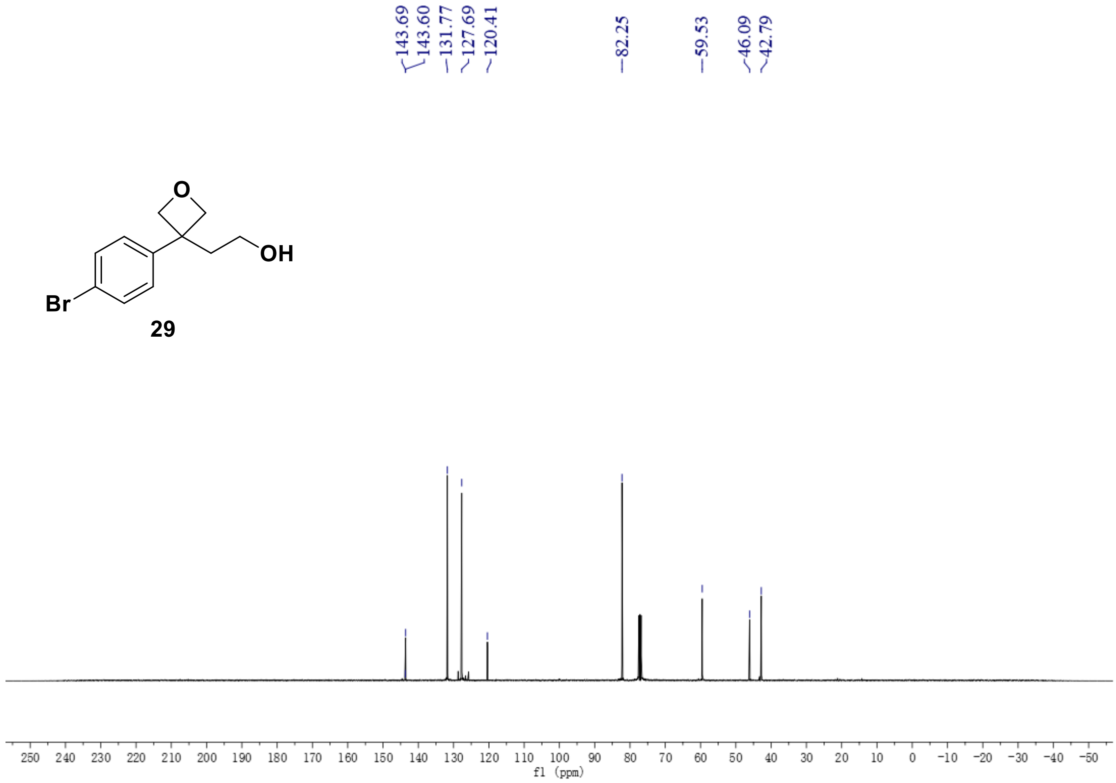

Supplementary Figure 38. ^{13}C NMR (101 MHz, CDCl$_3$) spectrum of 29.

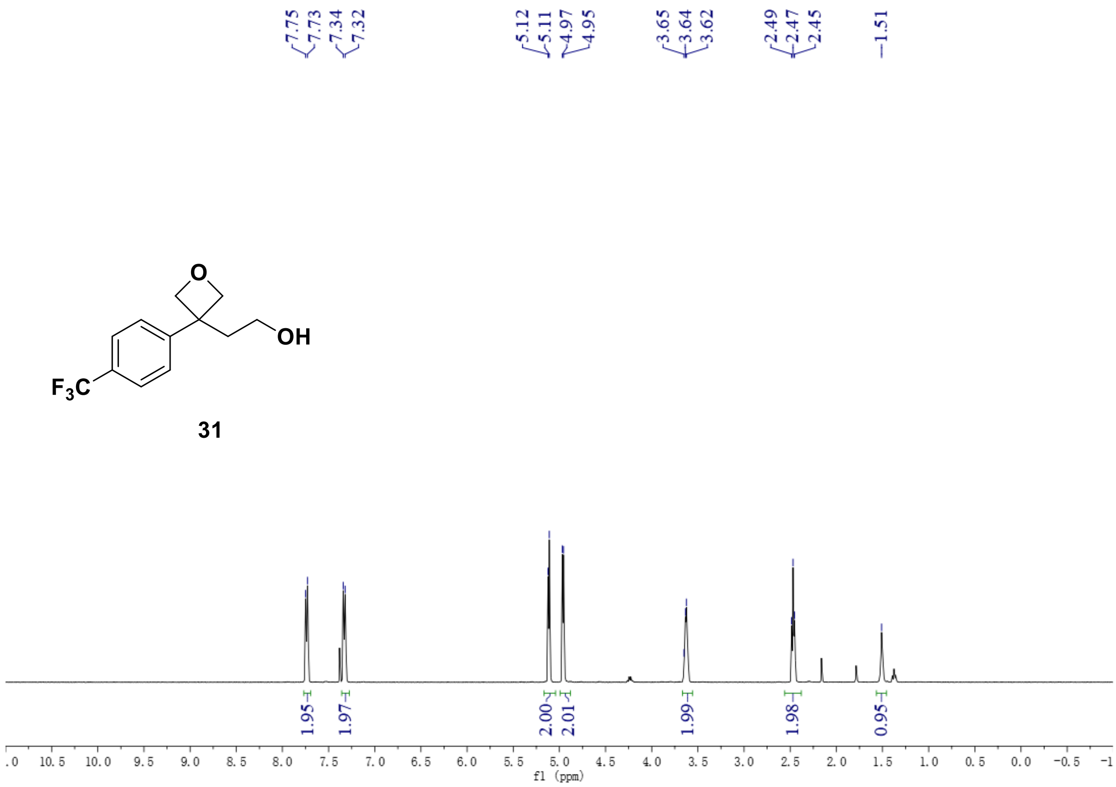

Supplementary Figure 39. ^{1}**H NMR (400 MHz, CDCl**$_3$**)** spectrum of **31.**

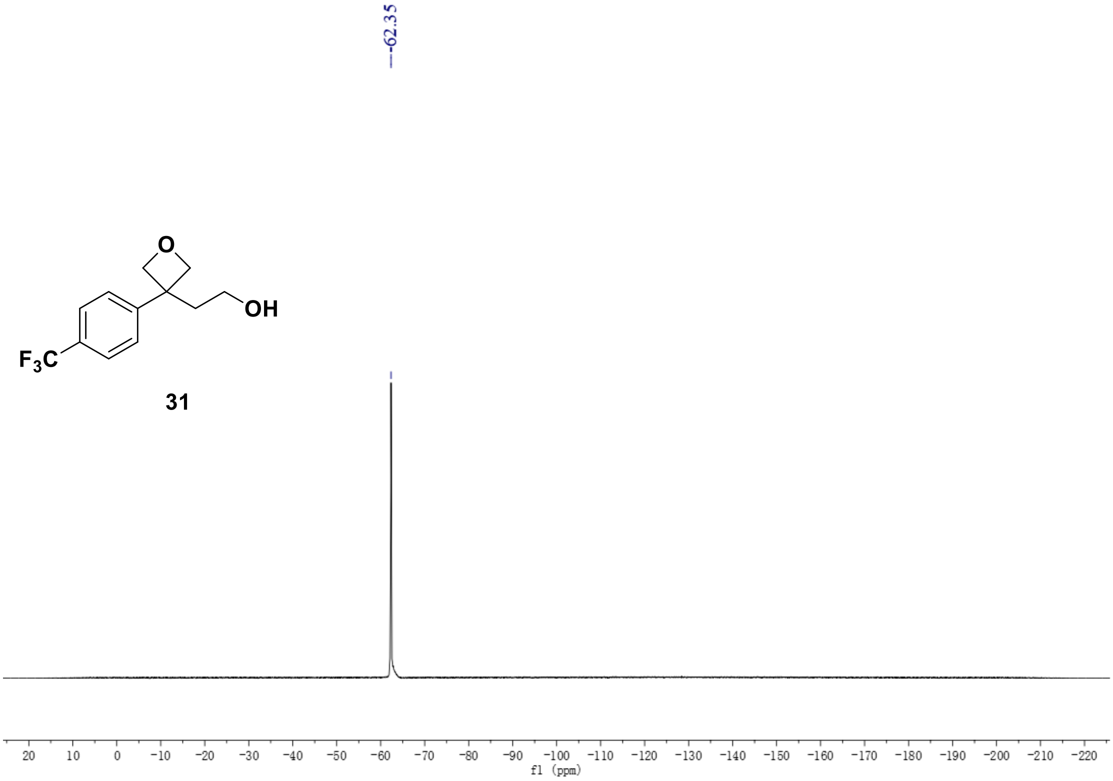

Supplementary Figure 40. ^{19}**F NMR (376 MHz, CDCl**$_3$**)** spectrum of **31.**

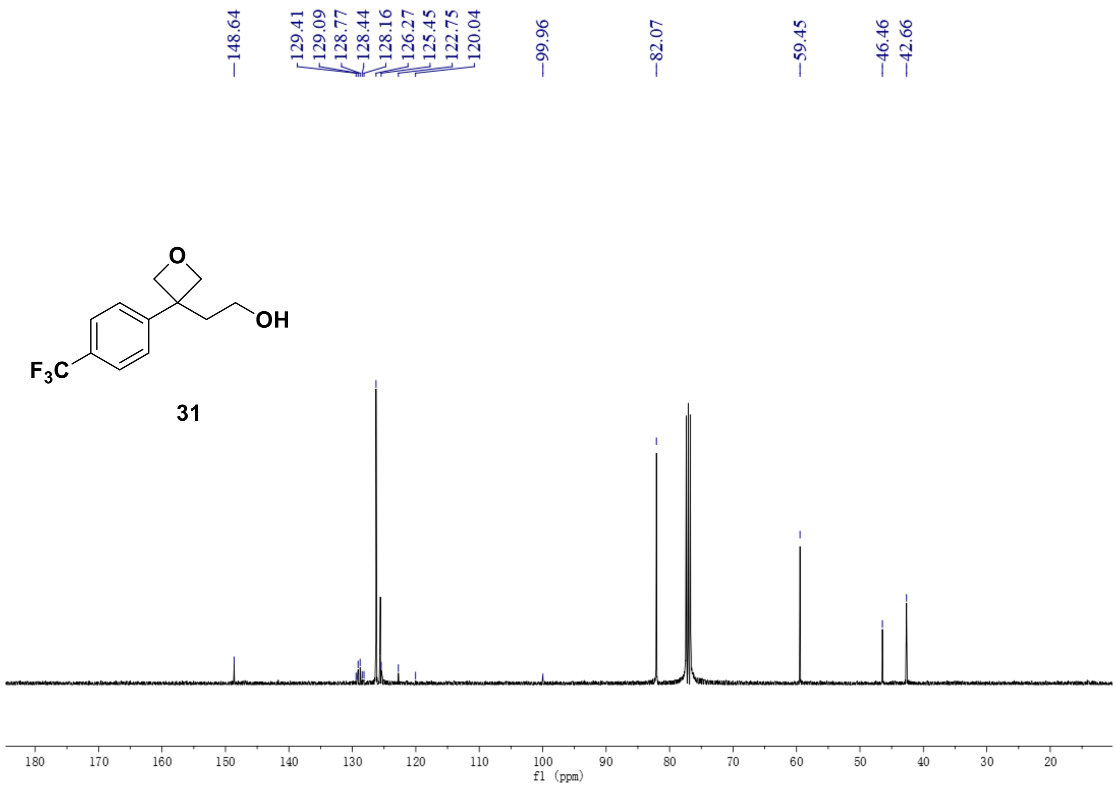

Supplementary Figure 41. ^{13}**C NMR (101 MHz, CDCl**$_3$**)** spectrum of **31.**

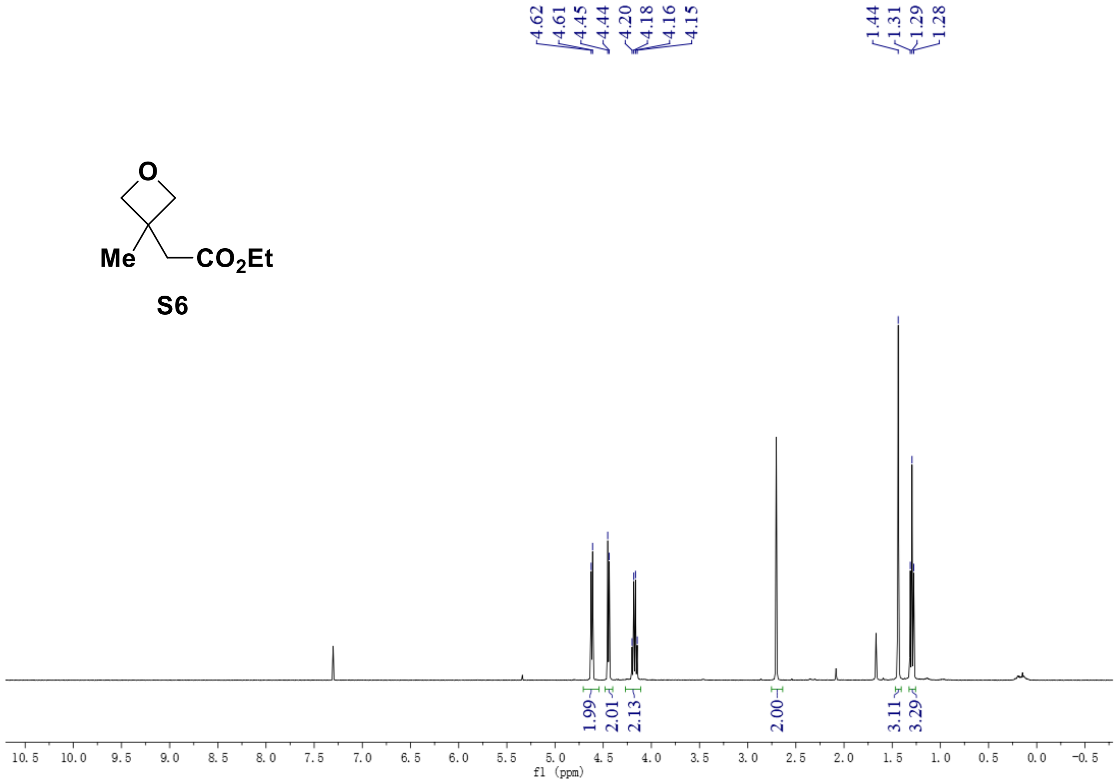

Supplementary Figure 42. ^{1}**H NMR (400 MHz, CDCl**$_3$**)** spectrum of **S6.**

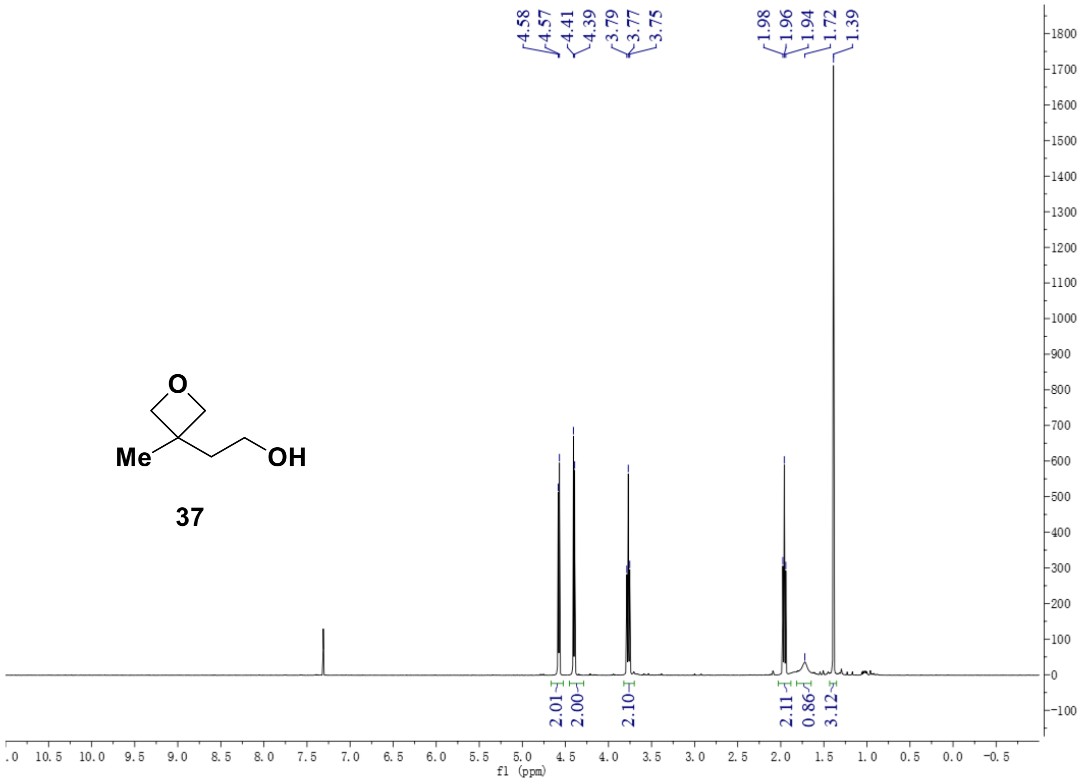

Supplementary Figure 43. **^{1}H NMR (400 MHz, $CDCl_3$)** spectrum of **37.**

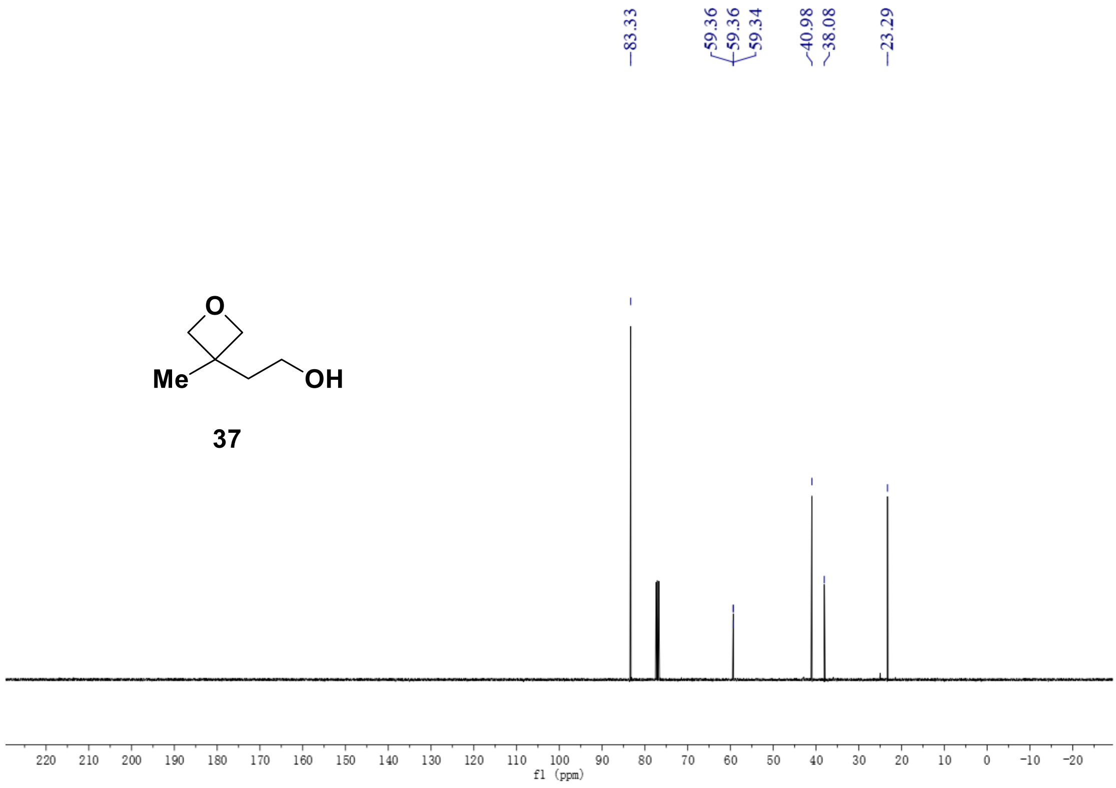

Supplementary Figure 44. **^{13}C NMR (101 MHz, $CDCl_3$)** spectrum of **37.**

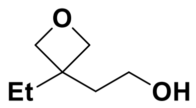

35

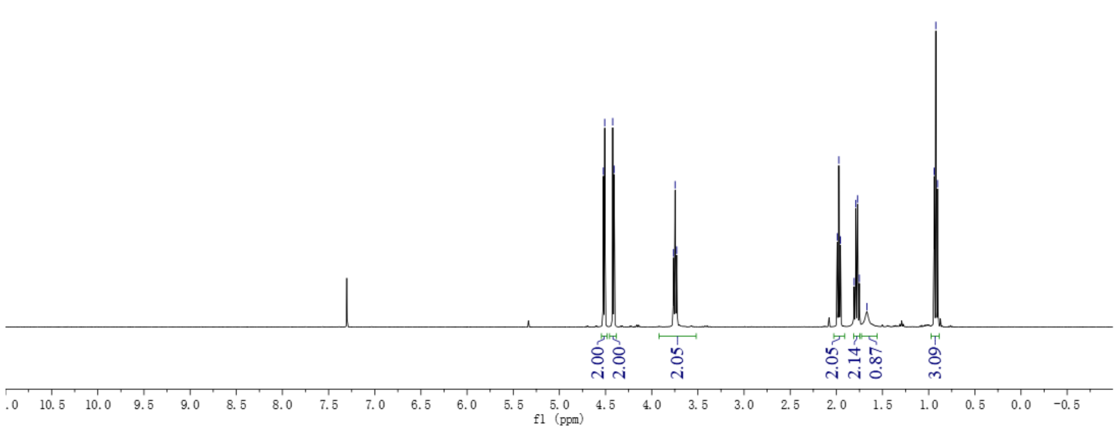

Supplementary Figure 45. ^{1}H NMR (400 MHz, $CDCl_3$) spectrum of **35.**

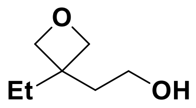

35

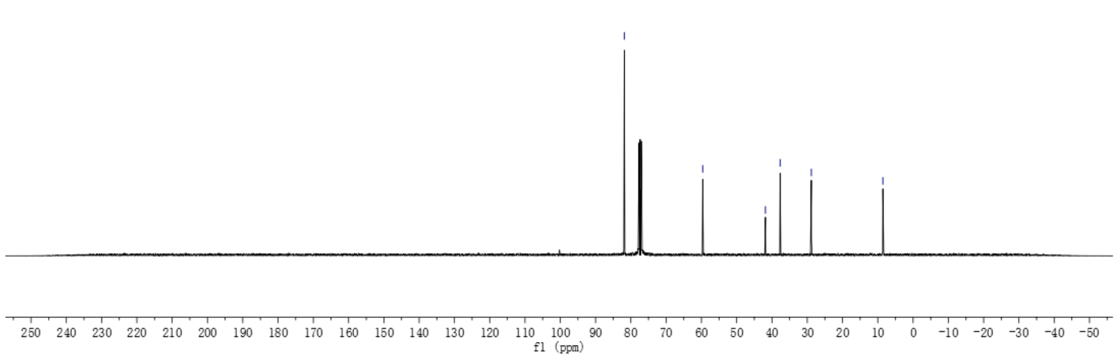

Supplementary Figure 46. ^{13}C NMR (101 MHz, $CDCl_3$) spectrum of **35.**

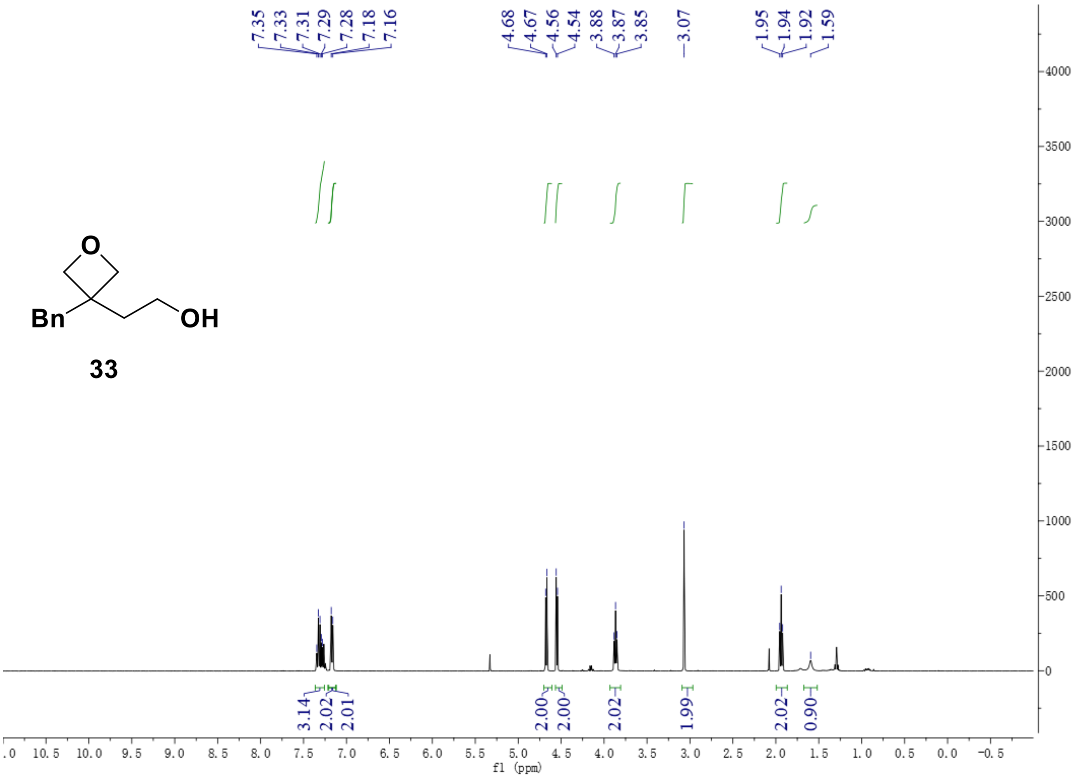

Supplementary Figure 47. ^{1}**H NMR (400 MHz, CDCl$_3$)** spectrum of **33.**

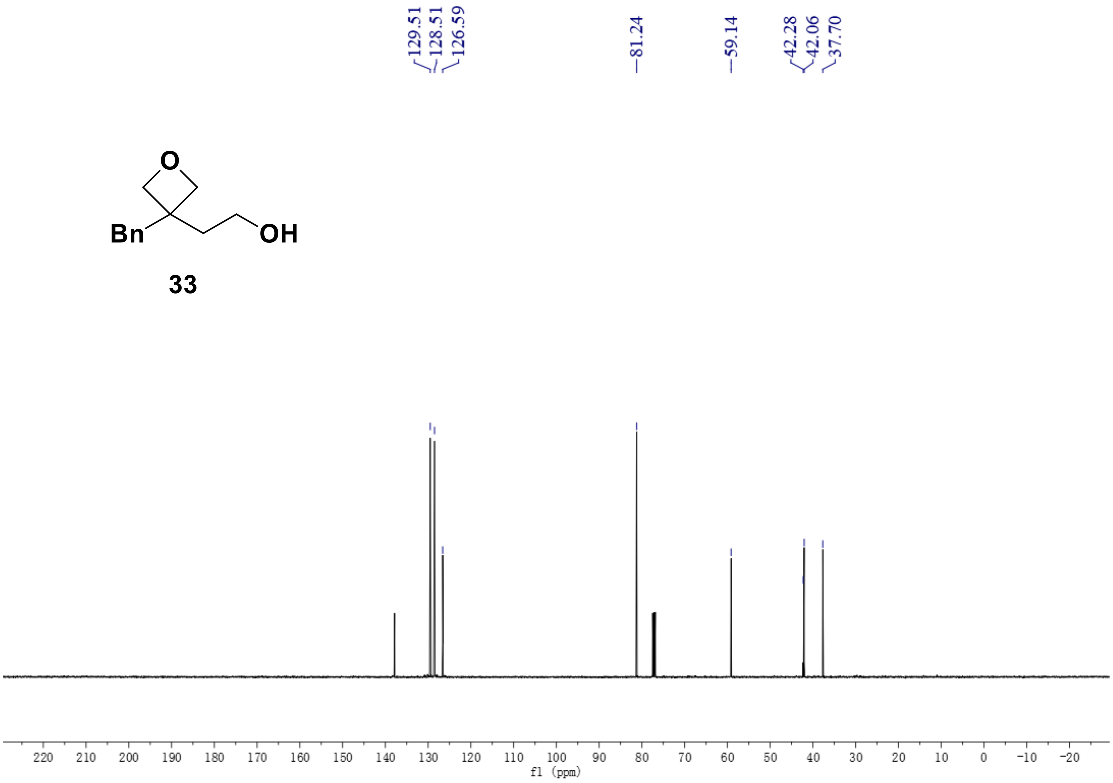

Supplementary Figure 48. ^{13}**C NMR (101 MHz, CDCl$_3$)** spectrum of **33.**

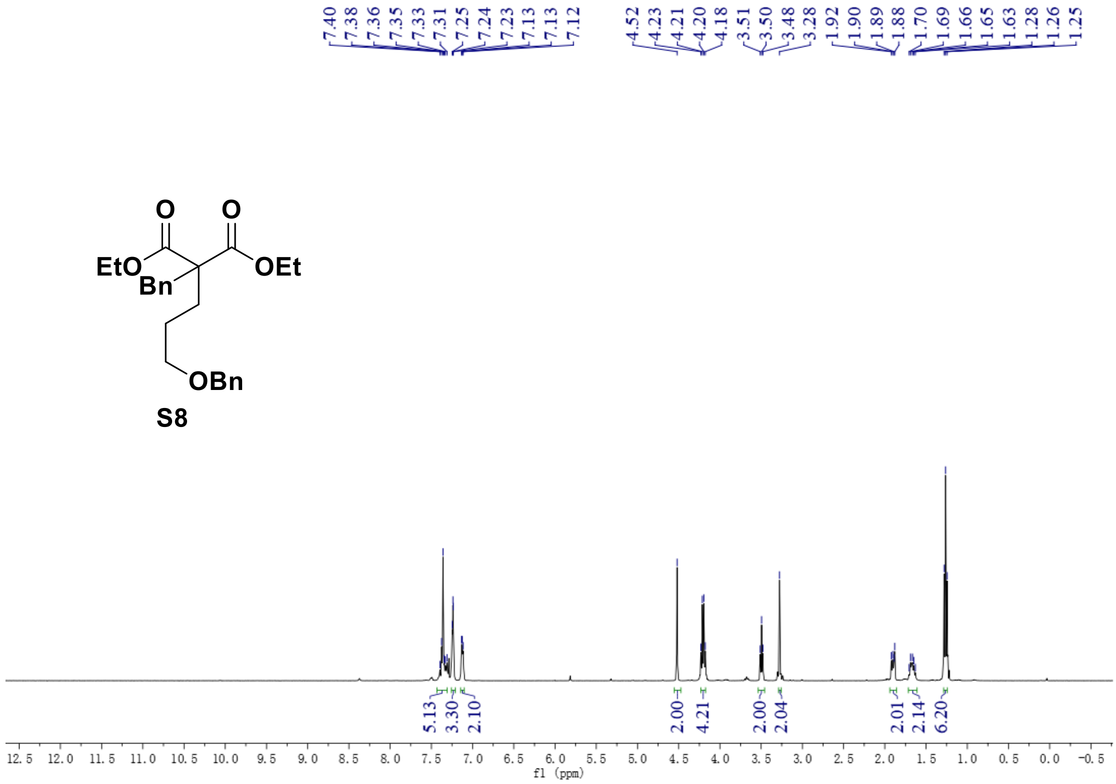

Supplementary Figure 49. **^{1}H NMR (400 MHz, $CDCl_3$)** spectrum of **S8.**

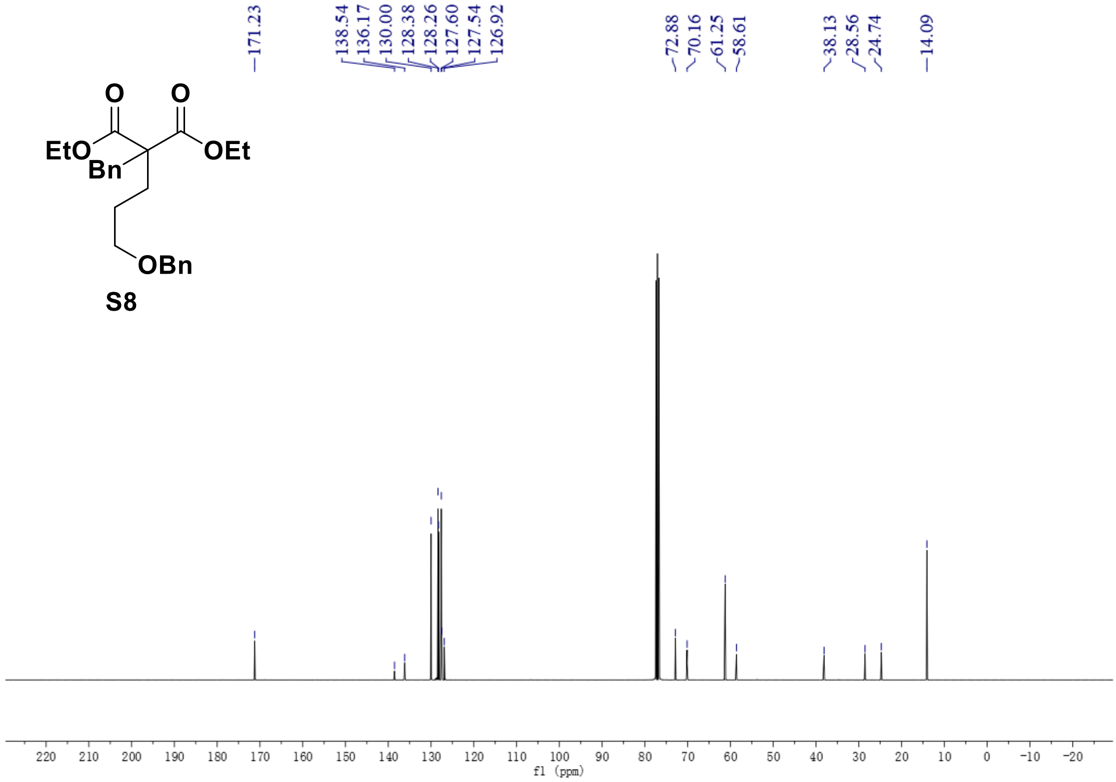

Supplementary Figure 50. **^{13}C NMR (101 MHz, $CDCl_3$)** spectrum of **S8.**

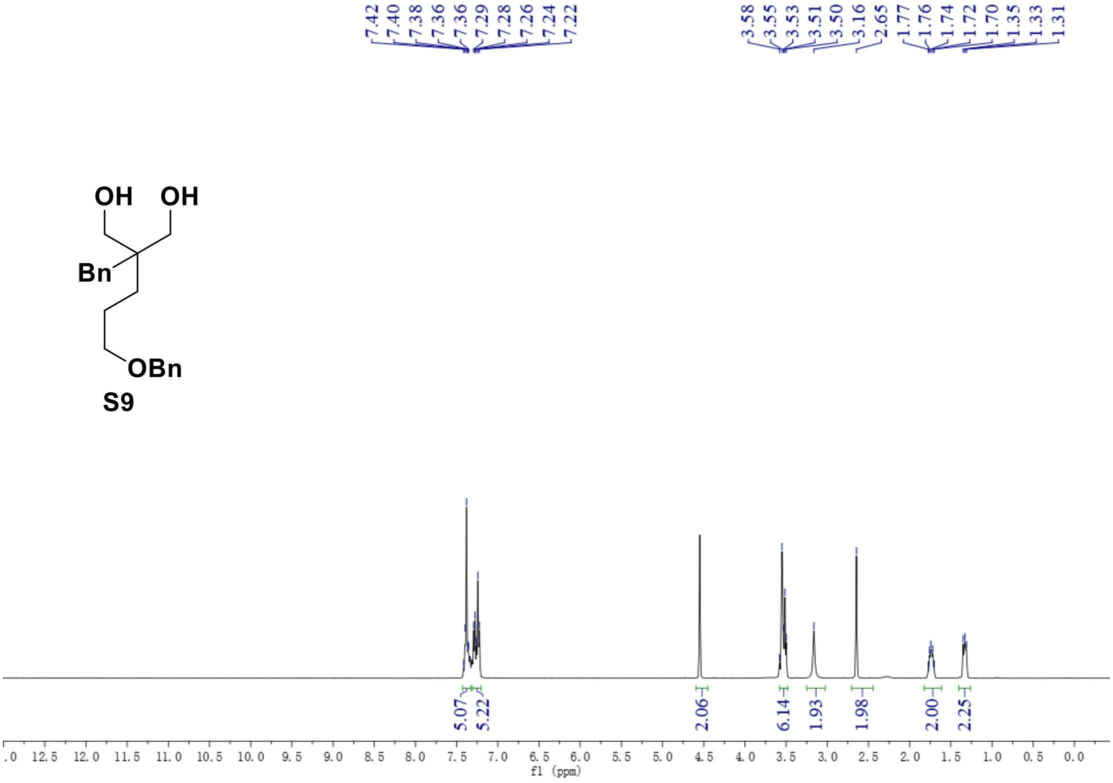

Supplementary Figure 51. **^{1}H NMR (400 MHz, $CDCl_3$)** spectrum of **S9.**

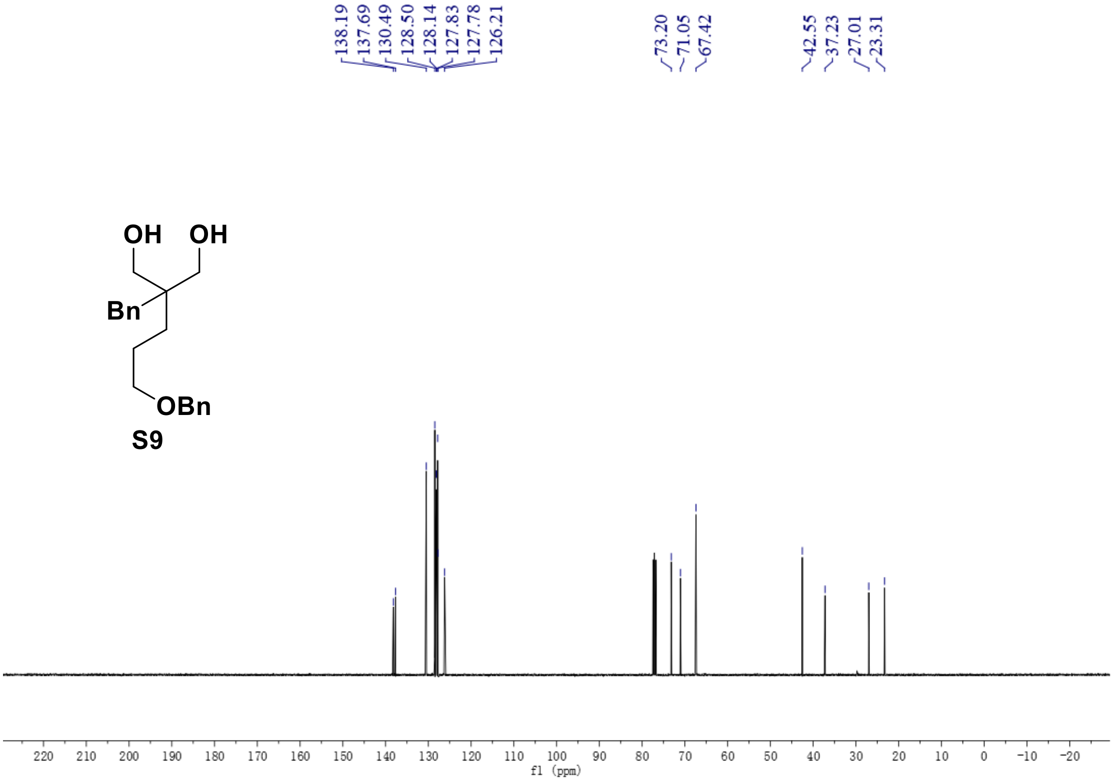

Supplementary Figure 52. **^{13}C NMR (101 MHz, $CDCl_3$)** spectrum of **S9.**

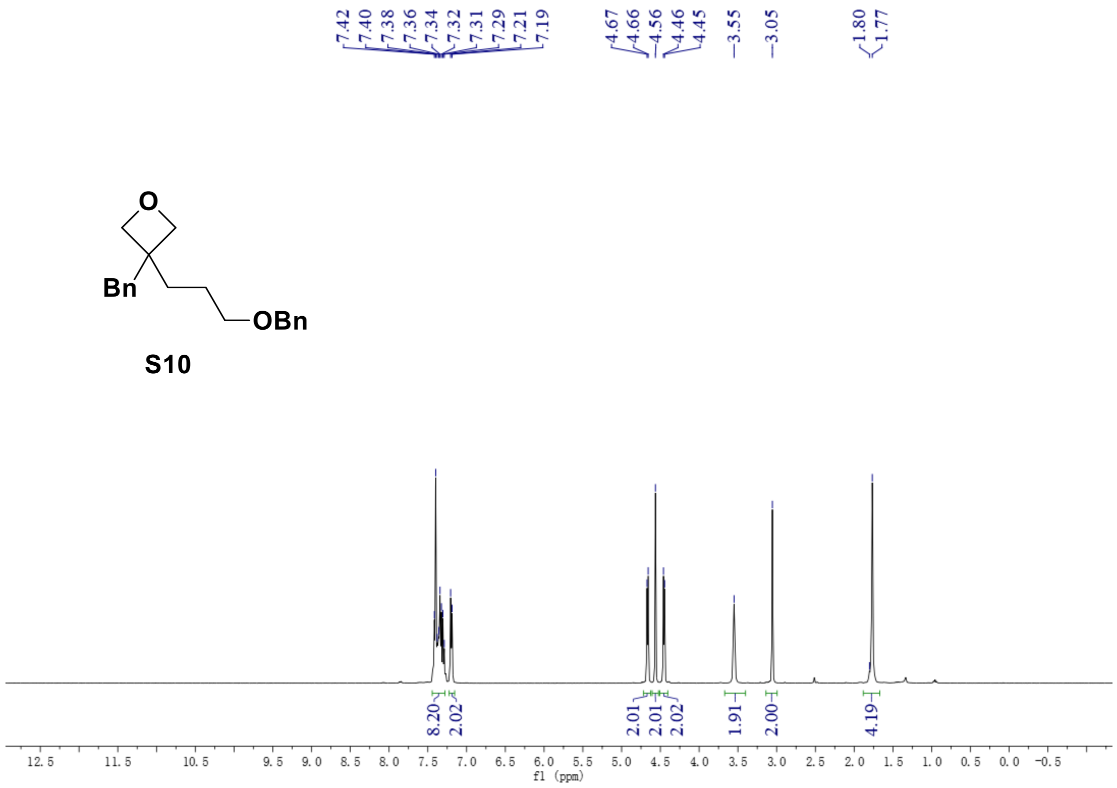

Supplementary Figure 53. ^{1}**H NMR (400 MHz, CDCl$_3$)** spectrum of **S10.**

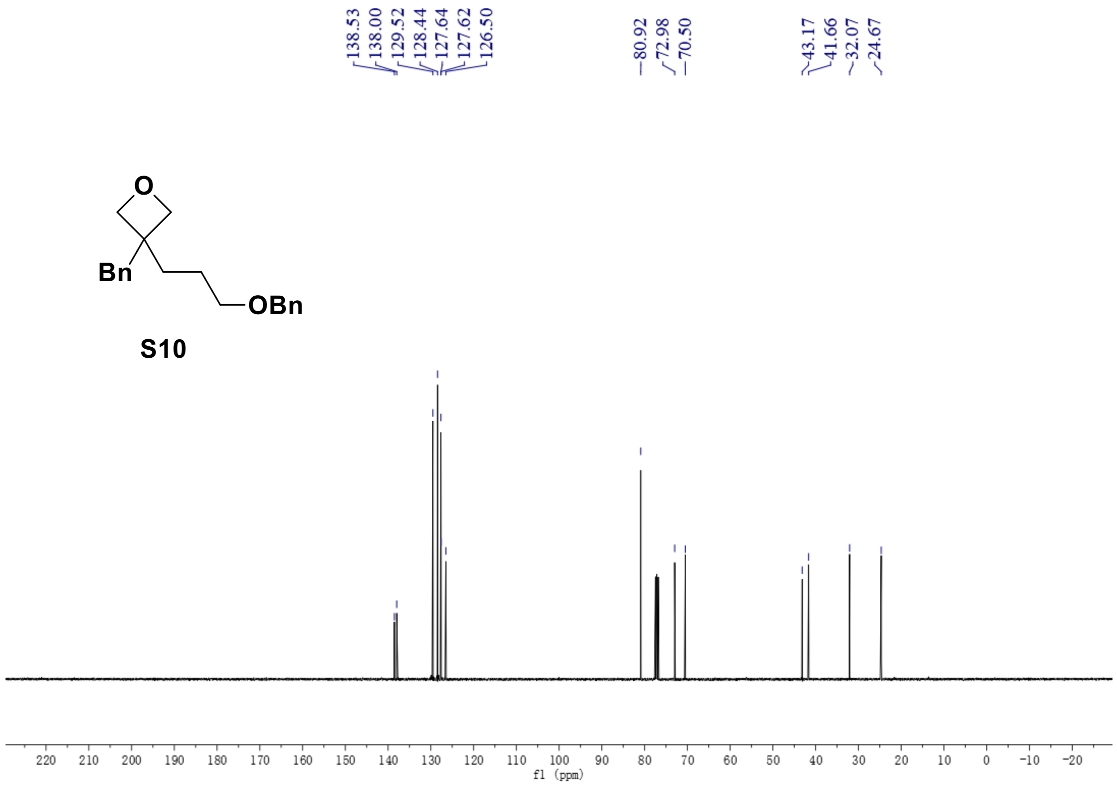

Supplementary Figure 54. ^{13}**C NMR (101 MHz, CDCl$_3$)** spectrum of **S10.**

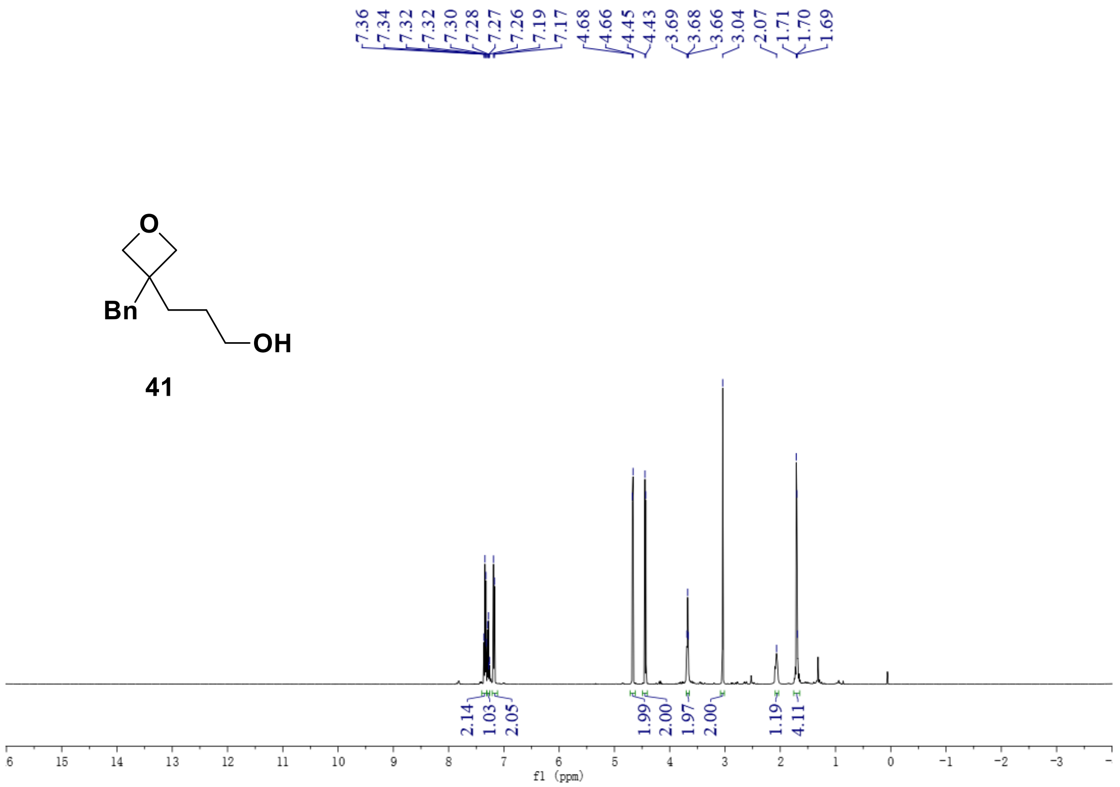

Supplementary Figure 55. ^{1}H NMR (400 MHz, $CDCl_3$) spectrum of **41.**

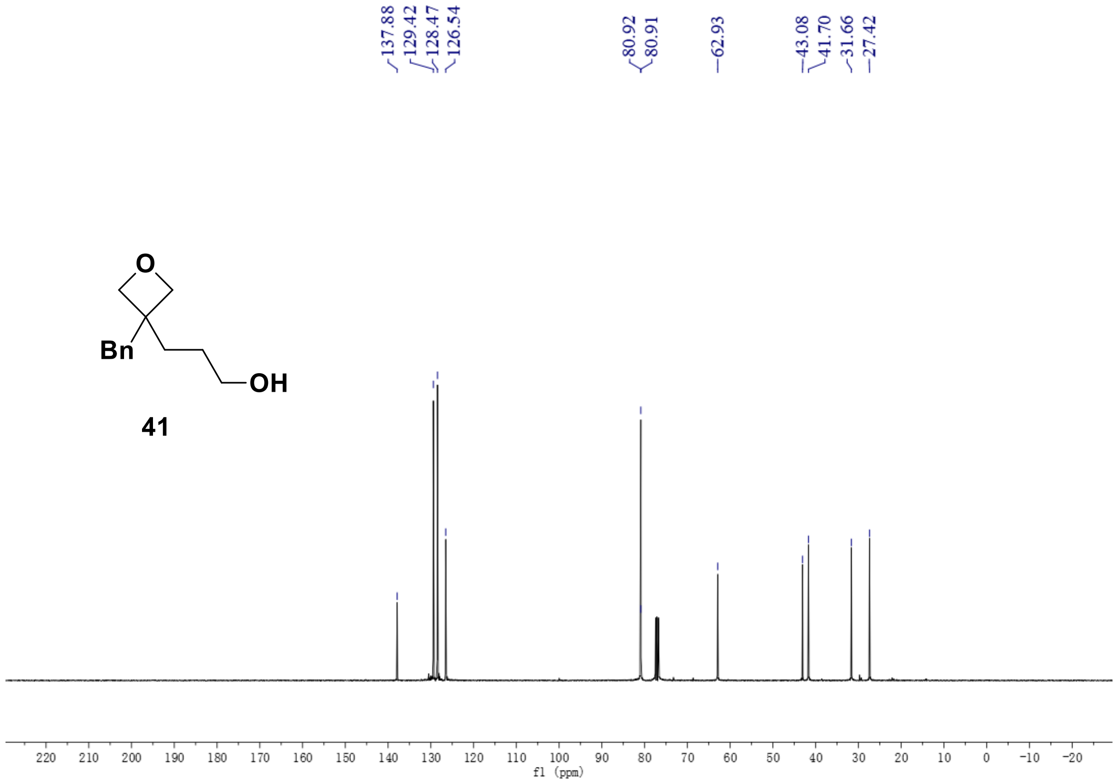

Supplementary Figure 56. ^{13}C NMR (101 MHz, $CDCl_3$) spectrum of **41.**

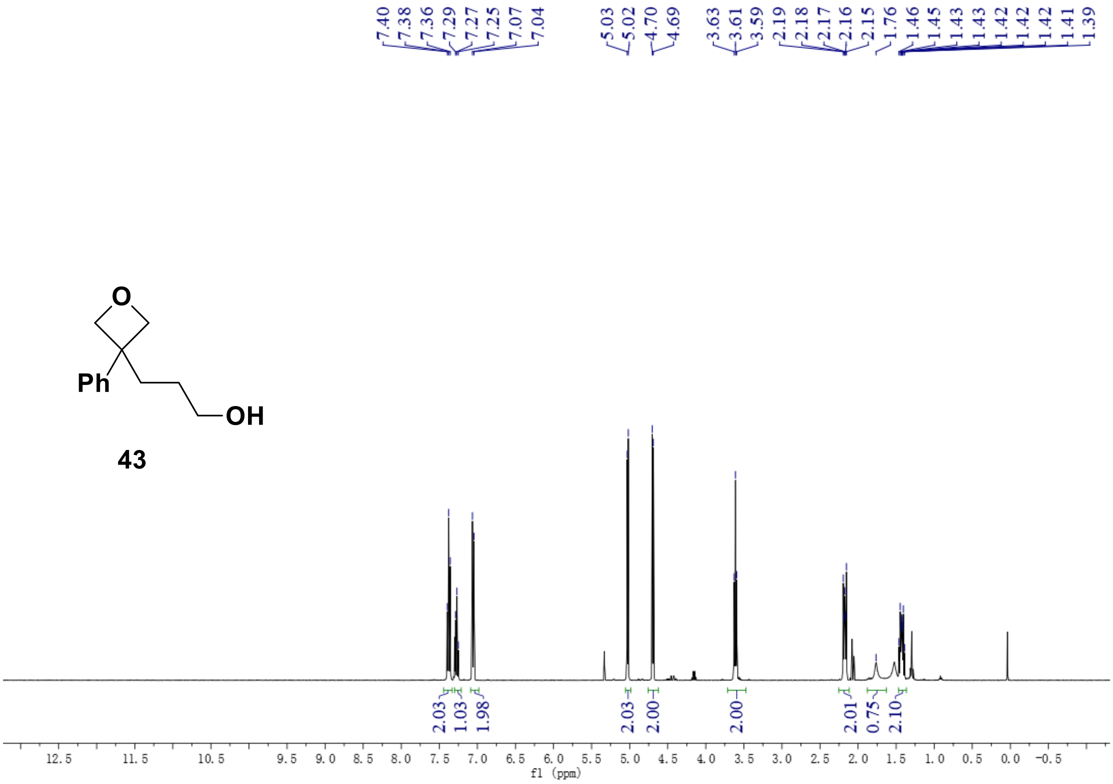

Supplementary Figure 57. **^{1}H NMR (400 MHz, $CDCl_3$)** spectrum of **43.**

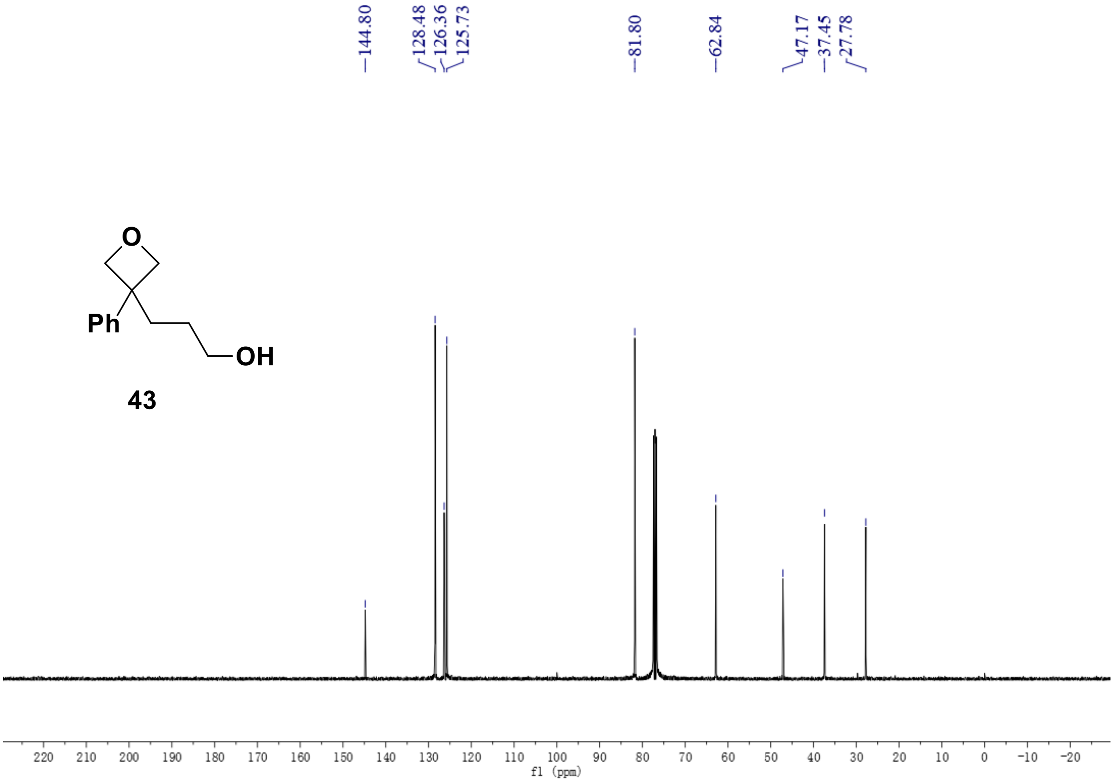

Supplementary Figure 58. **^{13}C NMR (101 MHz, $CDCl_3$)** spectrum of **43.**

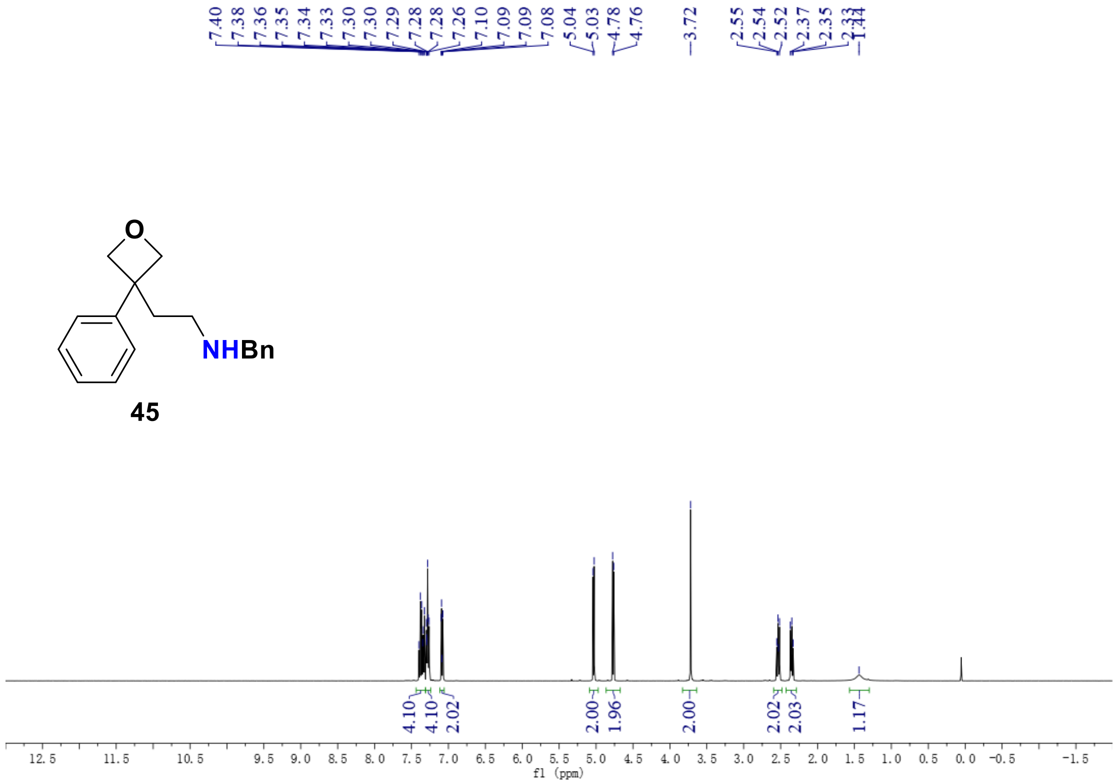

Supplementary Figure 59. ^{1}H NMR (400 MHz, $CDCl_3$) spectrum of **45.**

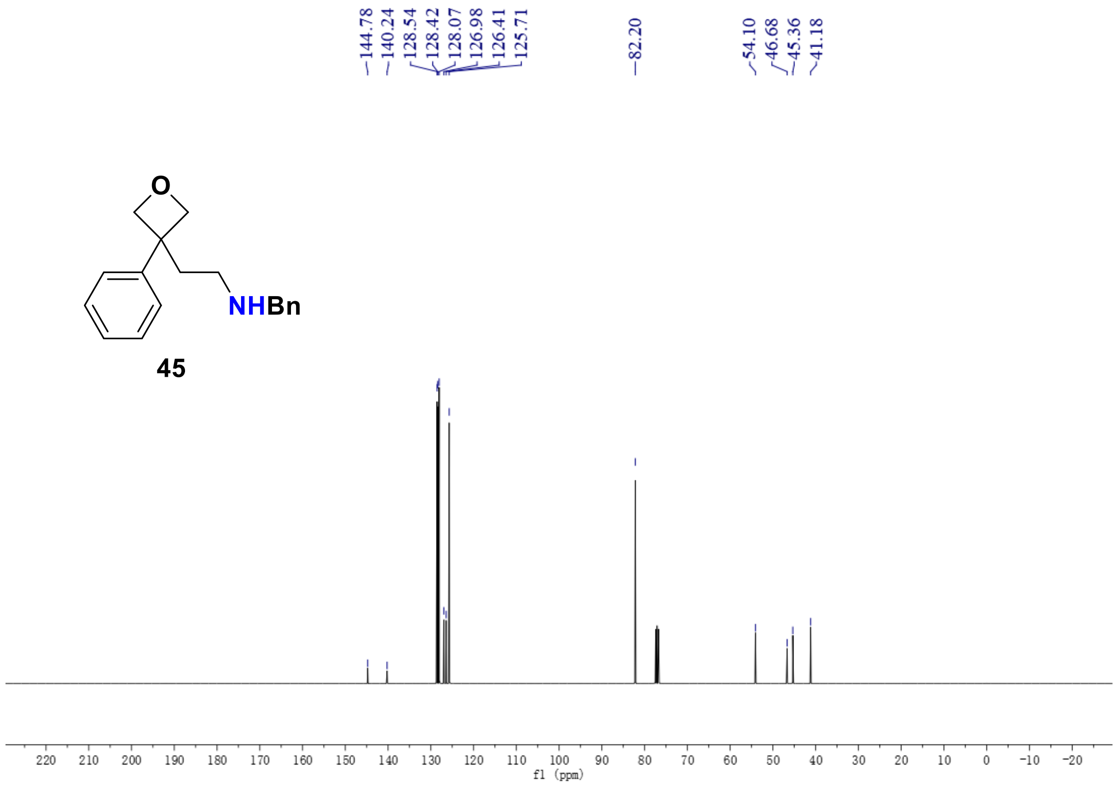

Supplementary Figure 60. ^{13}C NMR (101 MHz, $CDCl_3$) spectrum of **45.**

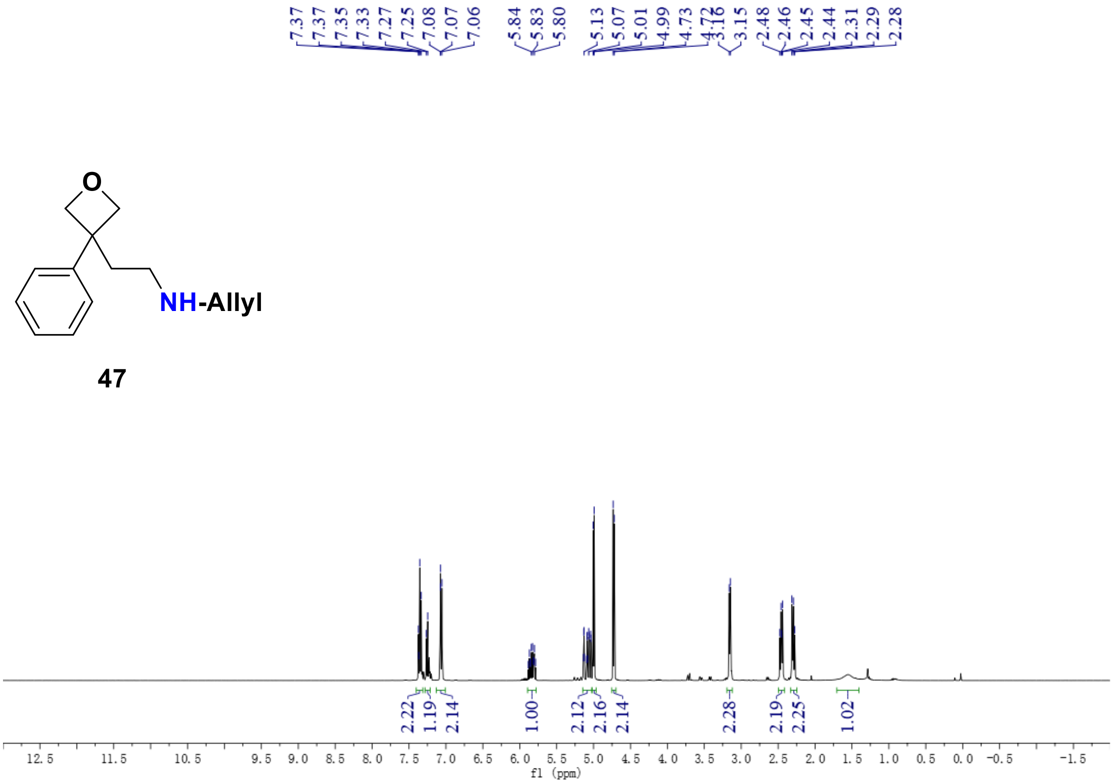

Supplementary Figure 61. **^{1}H NMR (400 MHz, $CDCl_3$)** spectrum of **47.**

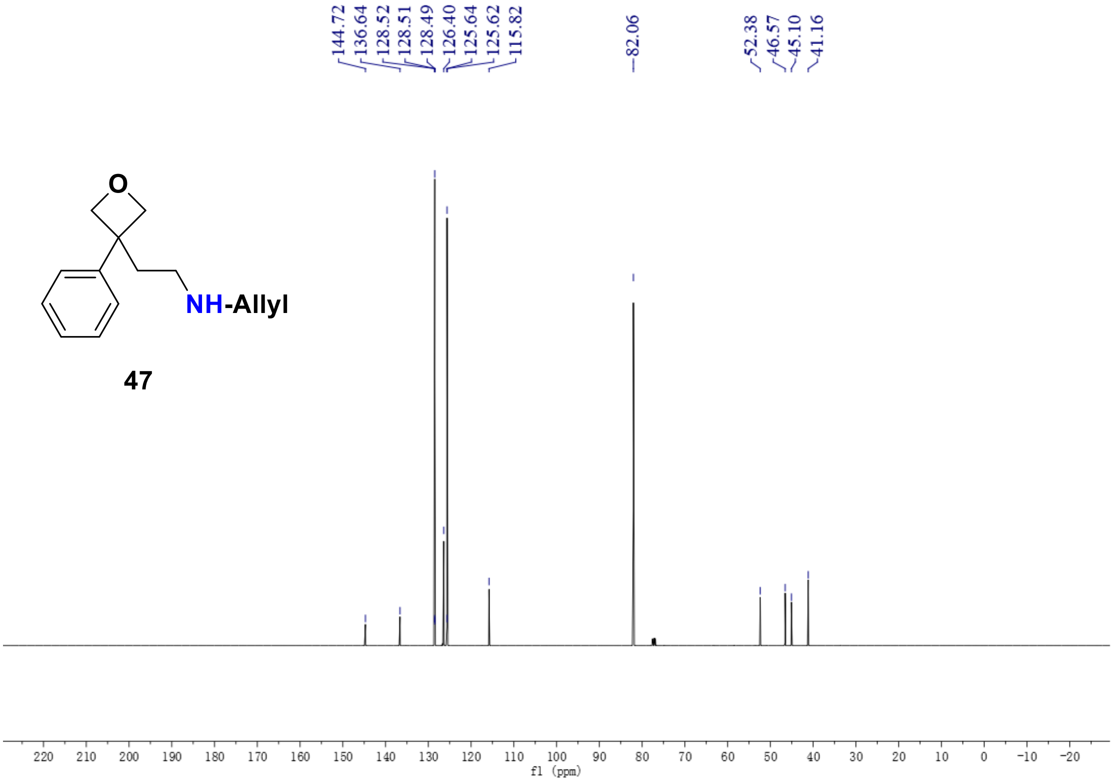

Supplementary Figure 62. **^{13}C NMR (101 MHz, $CDCl_3$)** spectrum of **47.**

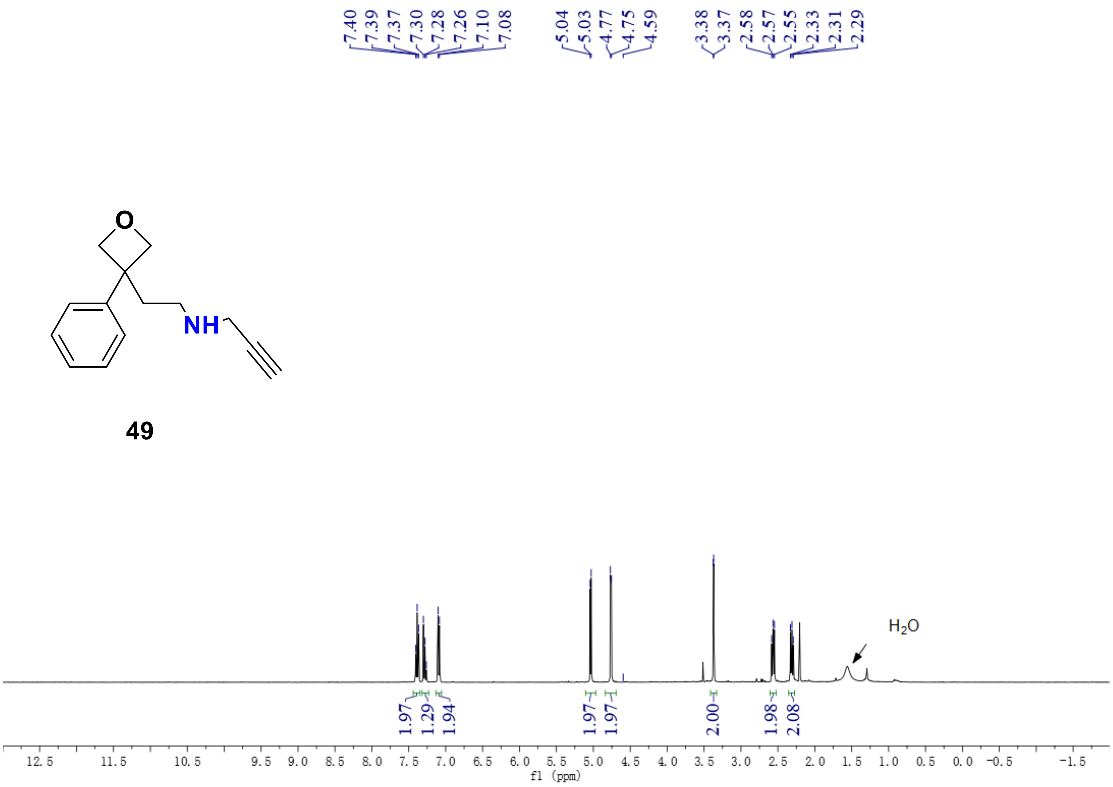

Supplementary Figure 63. ^{1}**H NMR (400 MHz, CDCl$_3$)** spectrum of **49.**

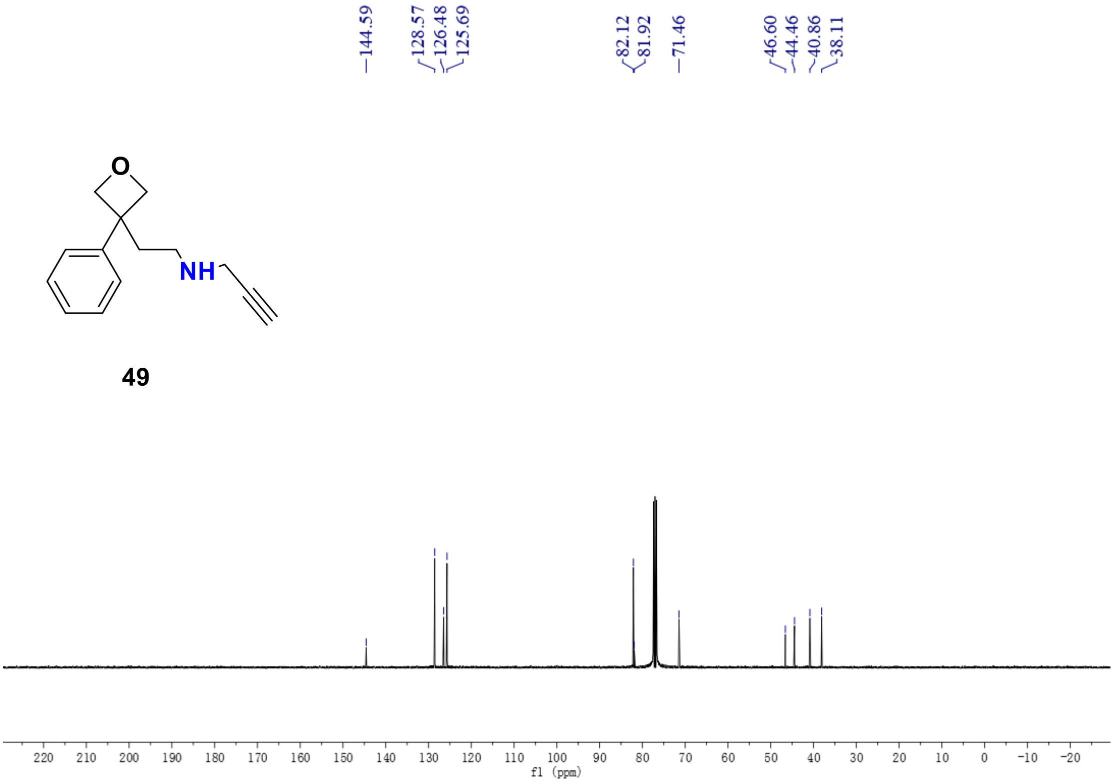

Supplementary Figure 64. ^{13}**C NMR (101 MHz, CDCl$_3$)** spectrum of **49.**

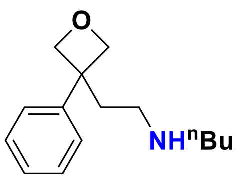

51

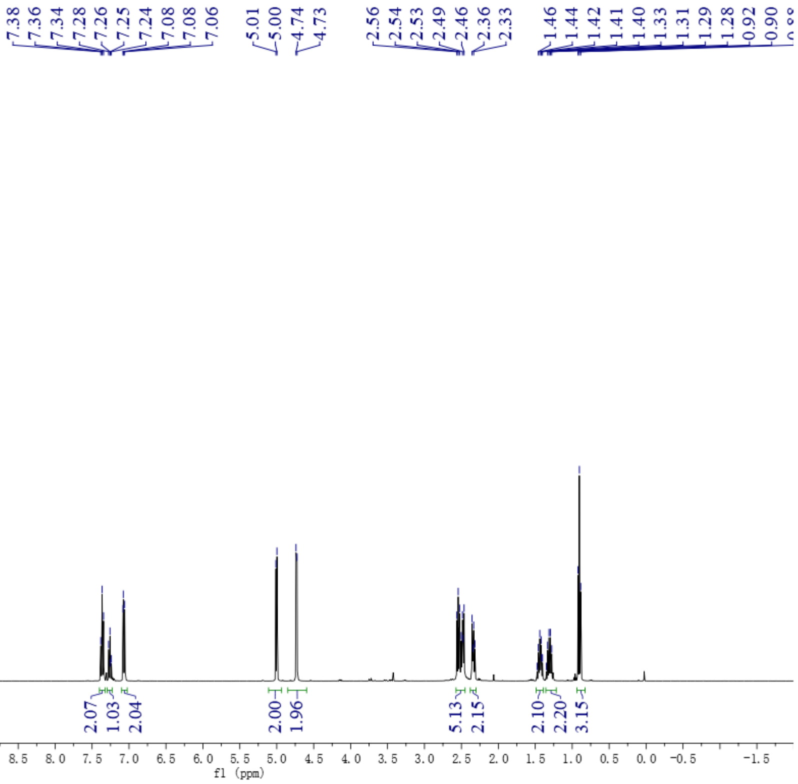

Supplementary Figure 65. ^{1}H NMR (400 MHz, $CDCl_3$) spectrum of **51.**

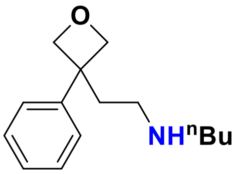

51

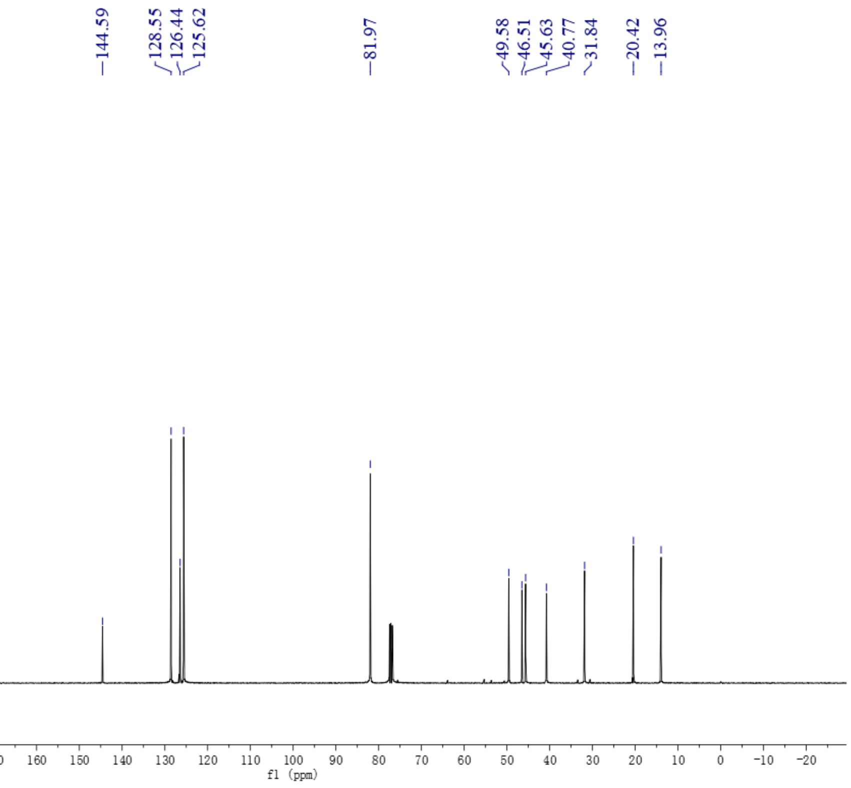

Supplementary Figure 66. ^{13}C NMR (101 MHz, $CDCl_3$) spectrum of **51.**

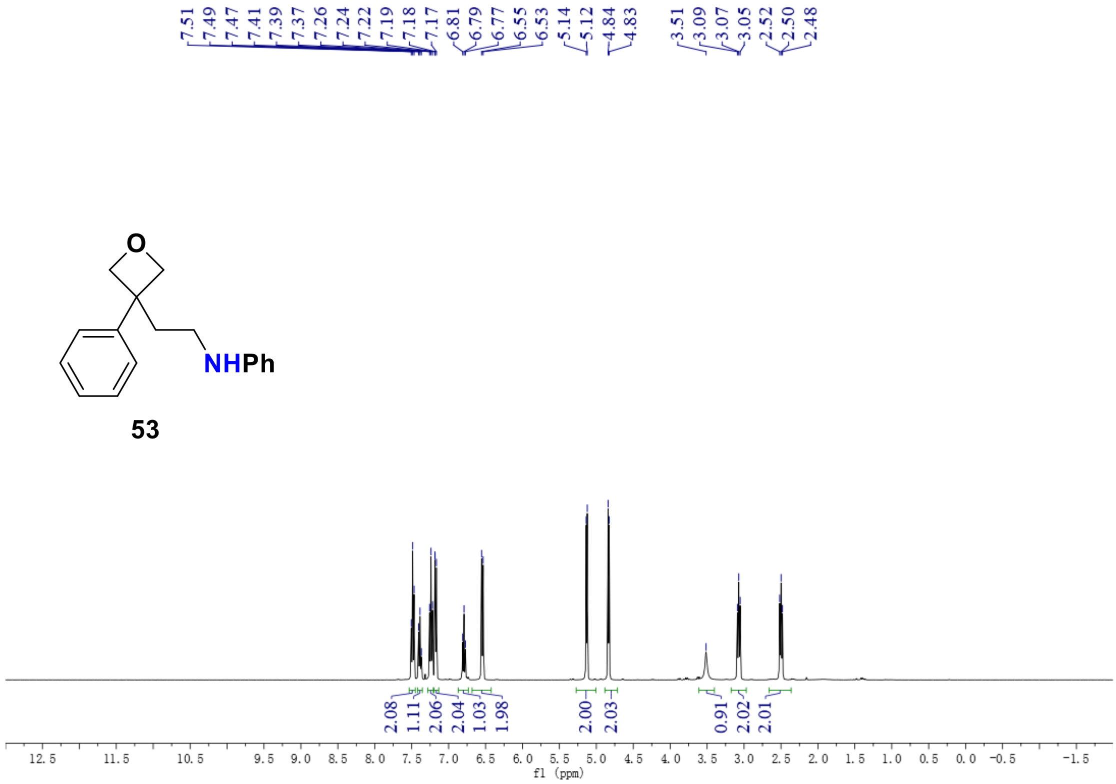

Supplementary Figure 67. **^{1}H NMR (400 MHz, $CDCl_3$)** spectrum of **53.**

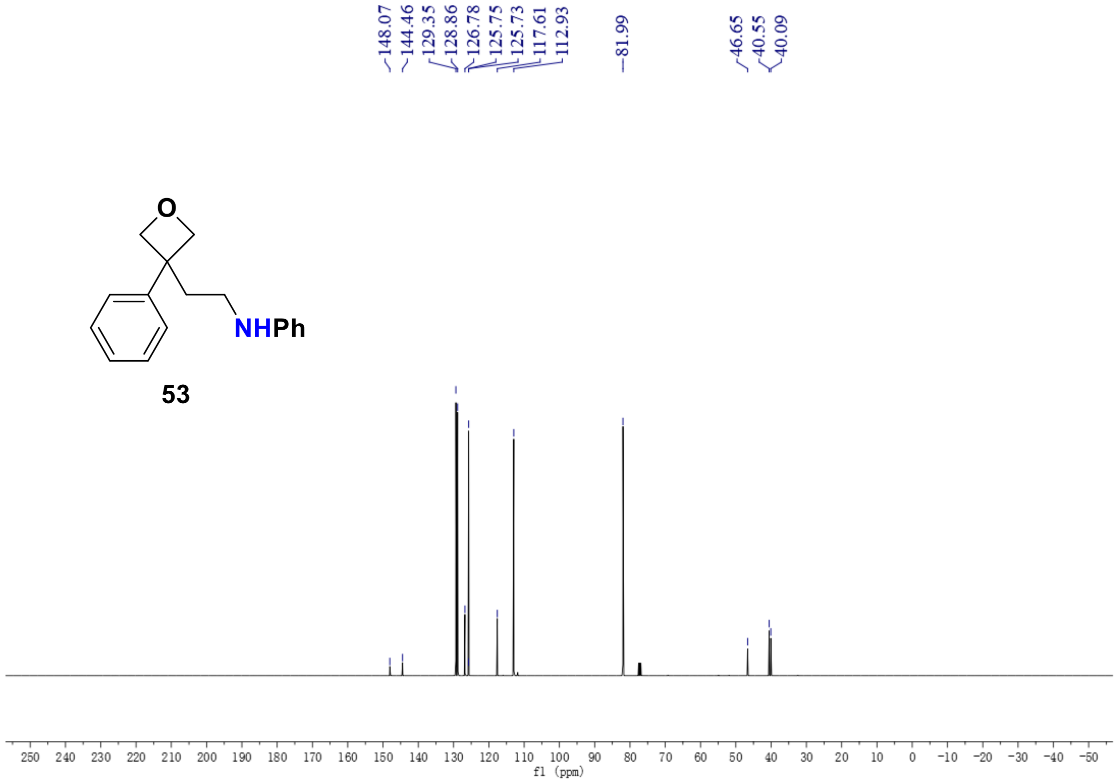

Supplementary Figure 68. **^{13}C NMR (101 MHz, $CDCl_3$)** spectrum of **53.**

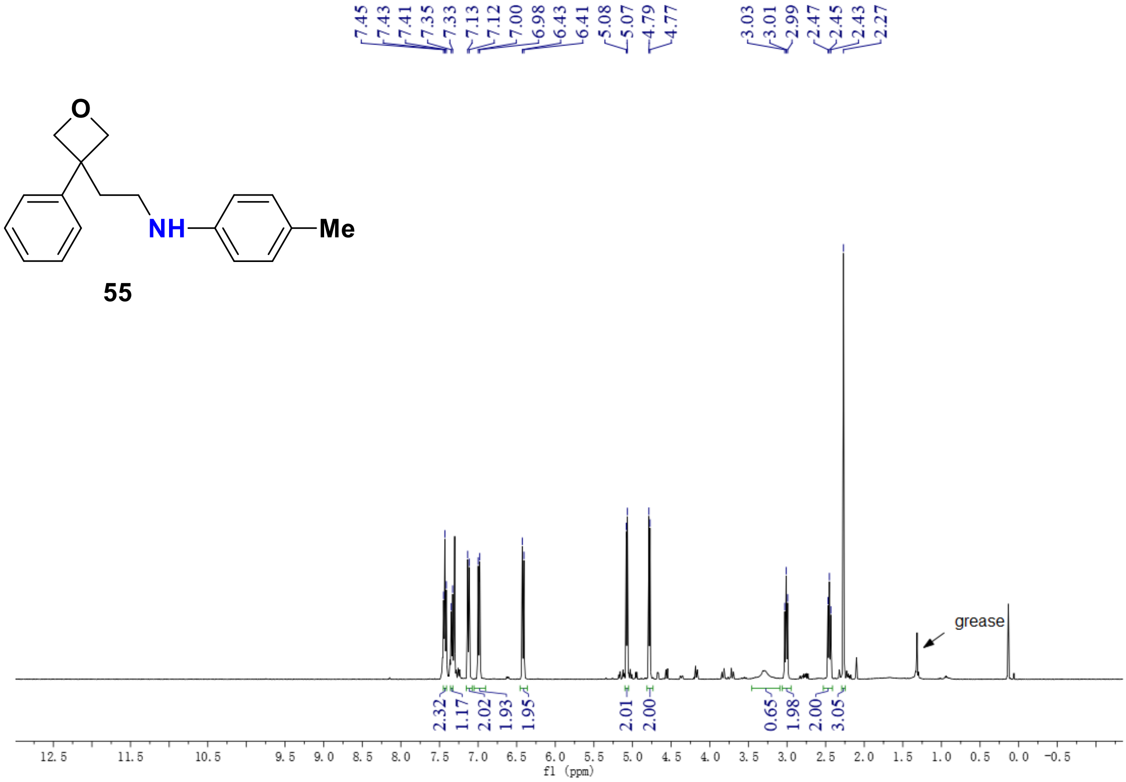

Supplementary Figure 69. **^{1}H NMR (400 MHz, $CDCl_3$)** spectrum of **55.**

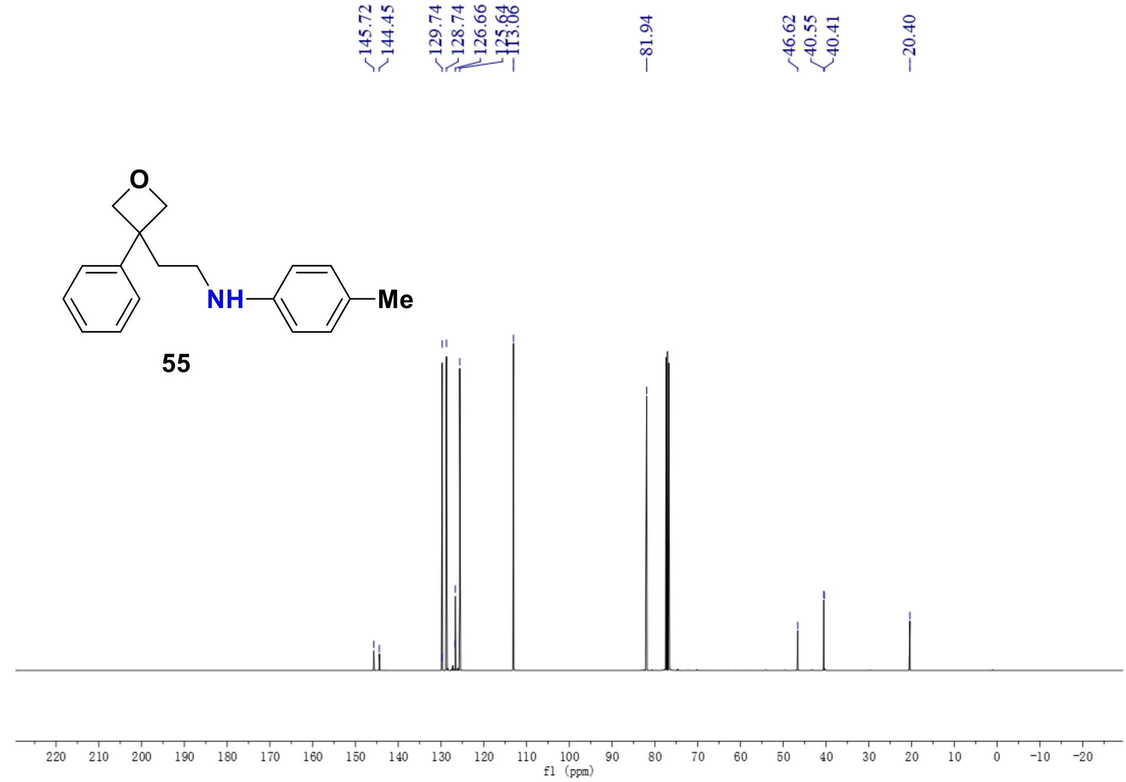

Supplementary Figure 70. **^{13}C NMR (101 MHz, $CDCl_3$)** spectrum of **55.**

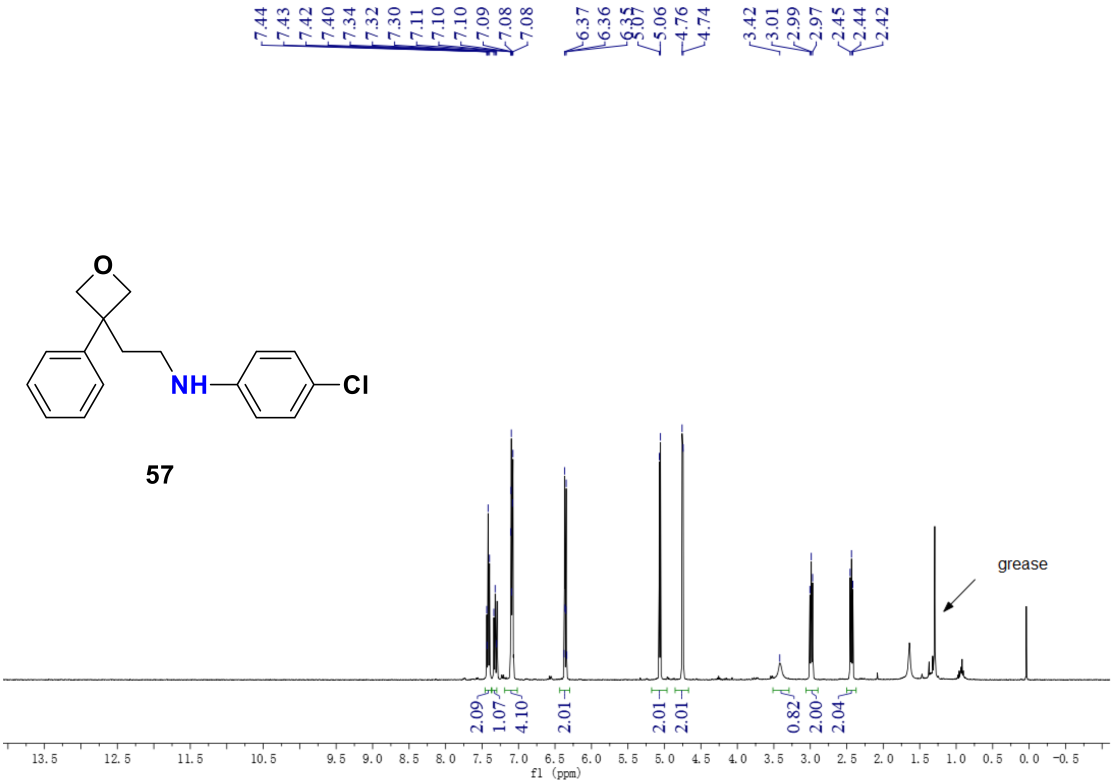

Supplementary Figure 71. **^{1}H NMR (400 MHz, $CDCl_3$)** spectrum of **57.**

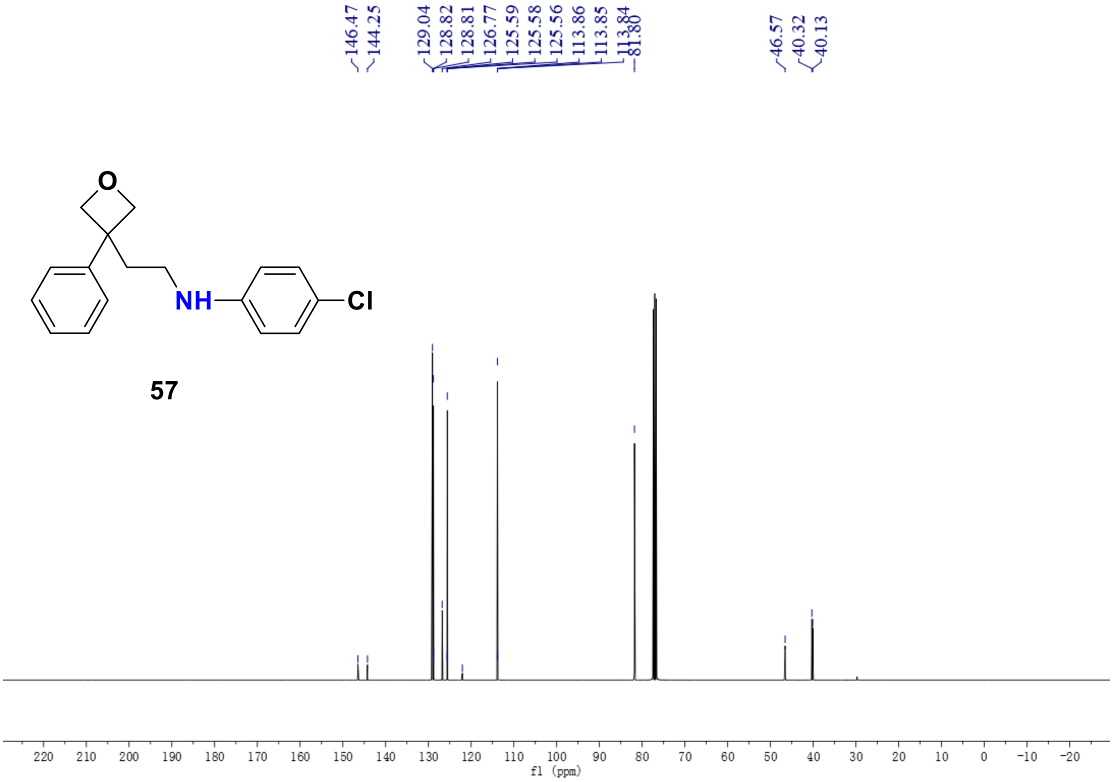

Supplementary Figure 72. **^{13}C NMR (101 MHz, $CDCl_3$)** spectrum of **57.**

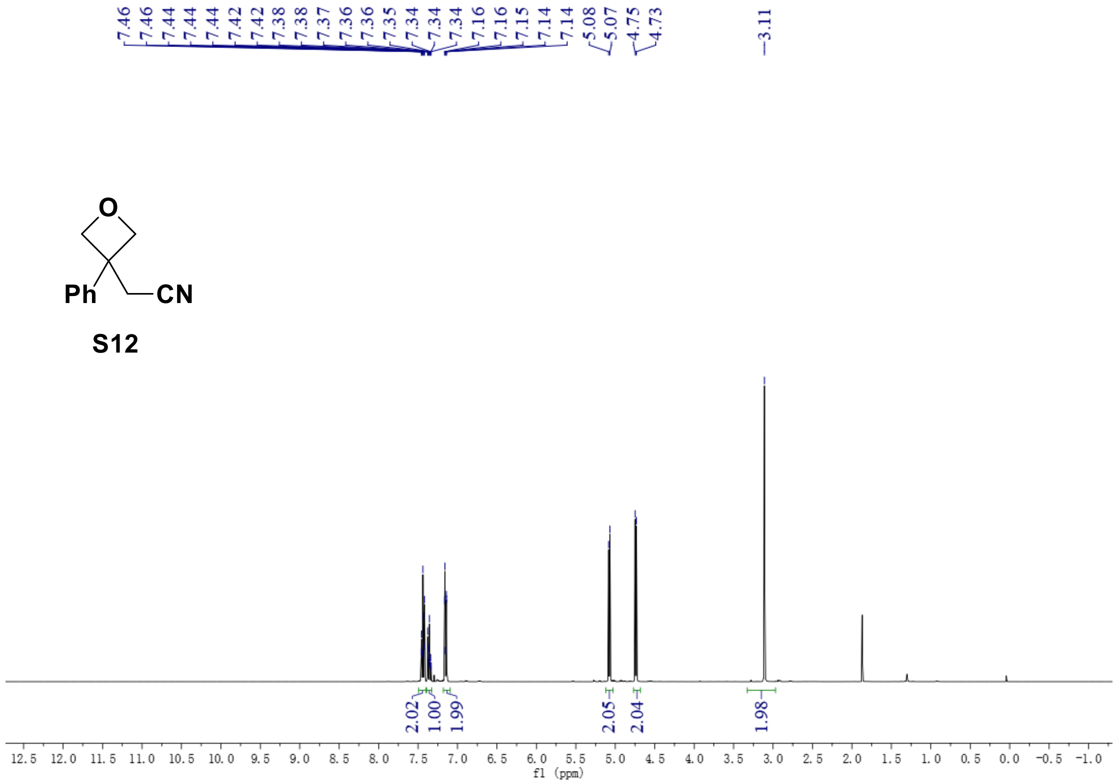

Supplementary Figure 73. **^{1}H NMR (400 MHz, $CDCl_3$)** spectrum of **S12.**

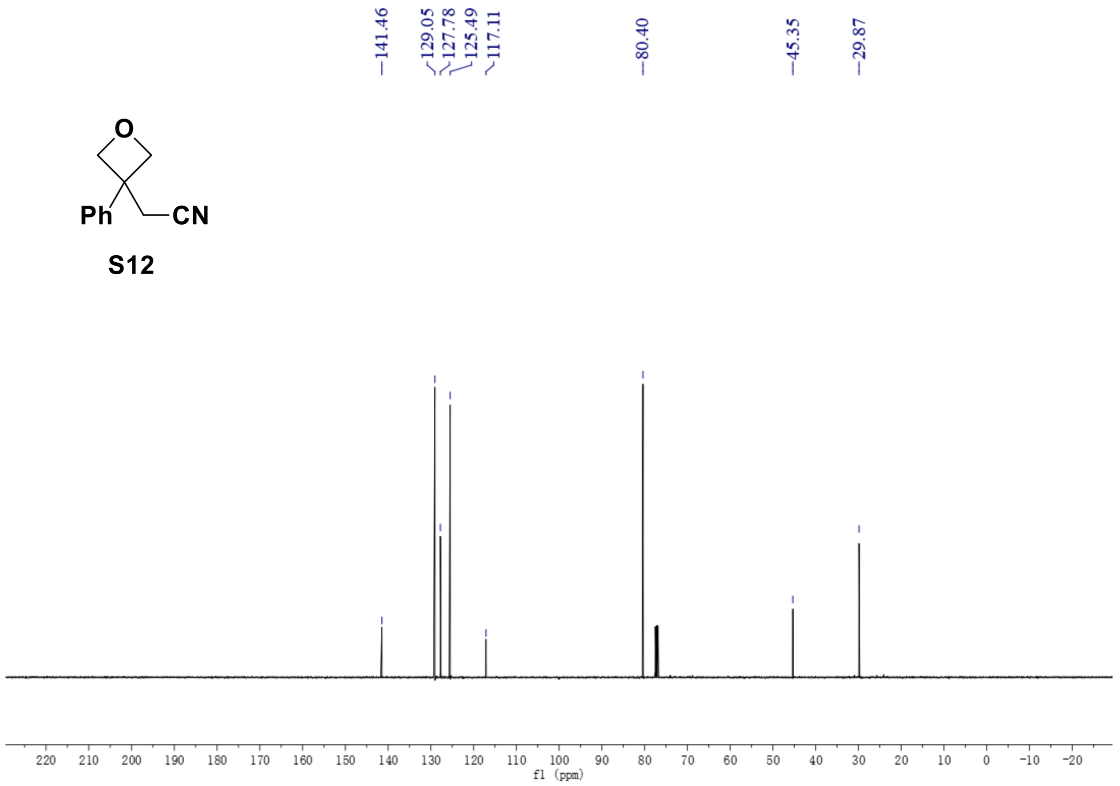

Supplementary Figure 74. **^{13}C NMR (101 MHz, $CDCl_3$)** spectrum of **S12.**

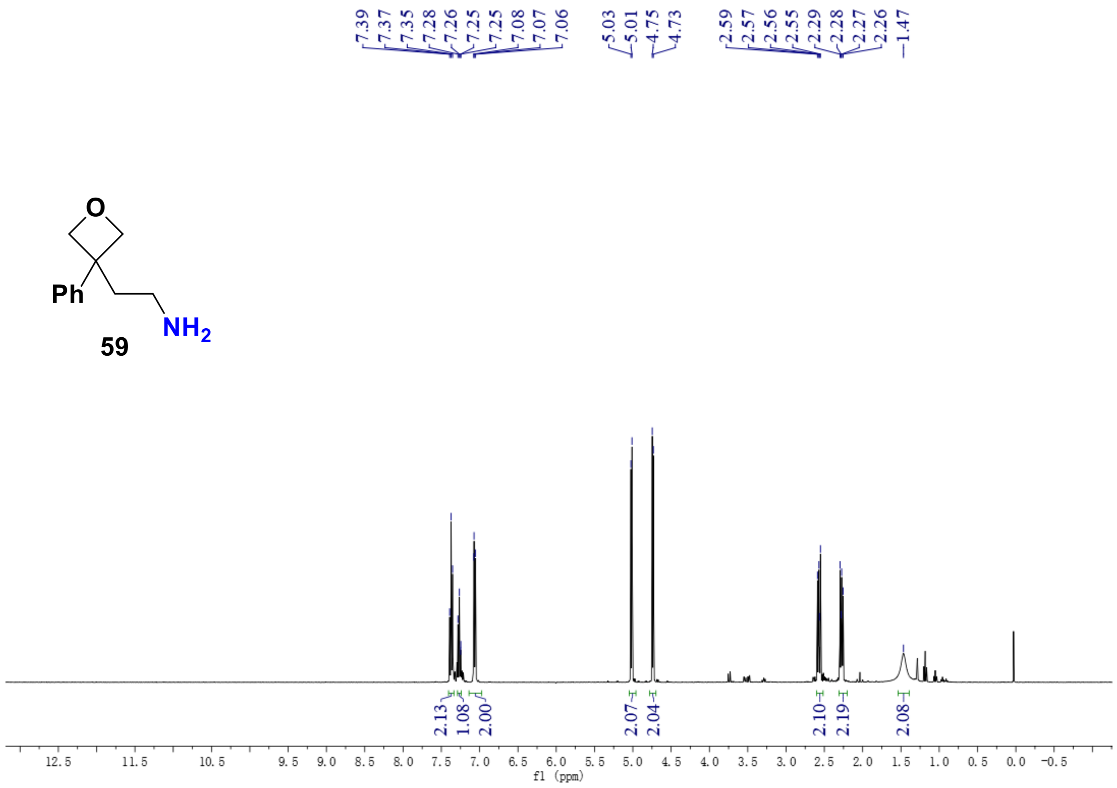

Supplementary Figure 75. ^{1}**H NMR (400 MHz, CDCl$_3$)** spectrum of **59.**

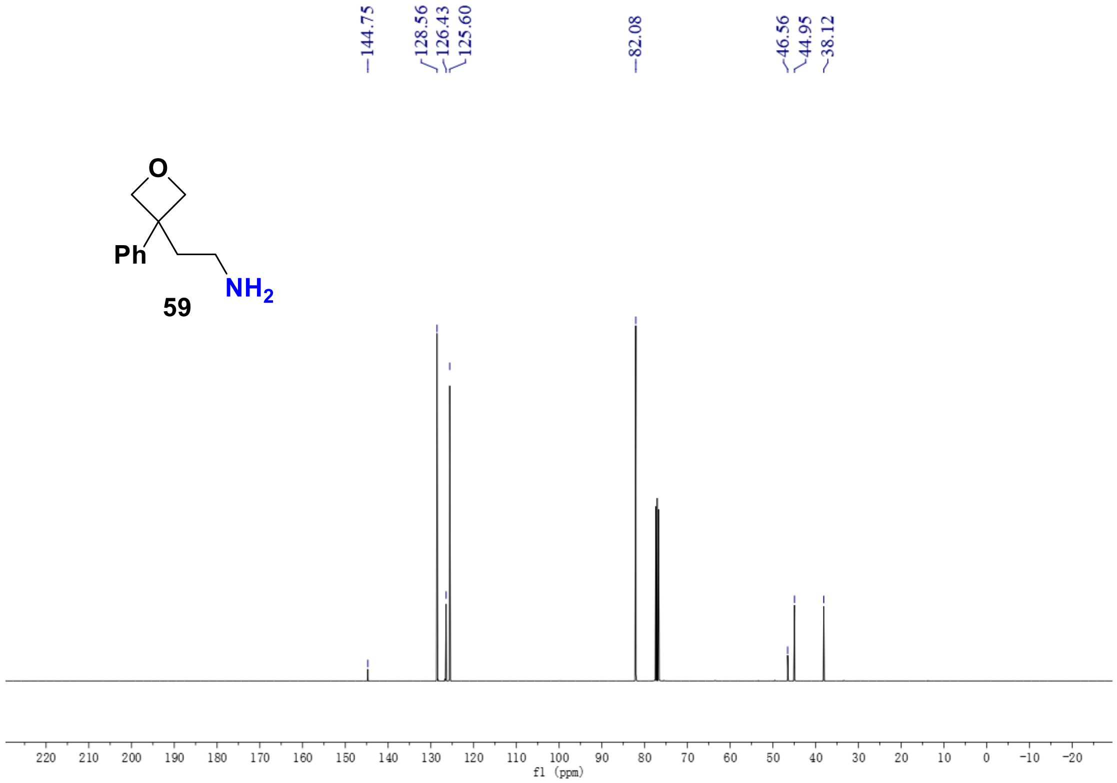

Supplementary Figure 76. ^{13}**C NMR (101 MHz, CDCl$_3$)** spectrum of **59.**

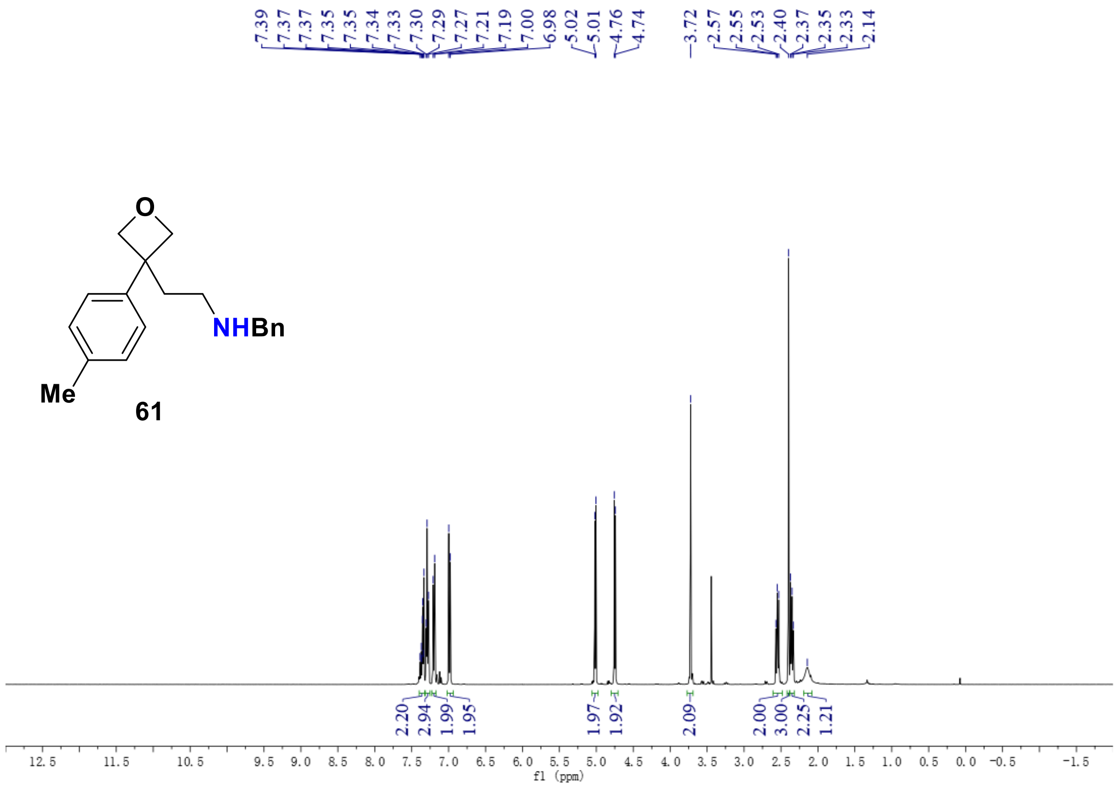

Supplementary Figure 77. ^{1}H NMR (400 MHz, $CDCl_3$) spectrum of **61.**

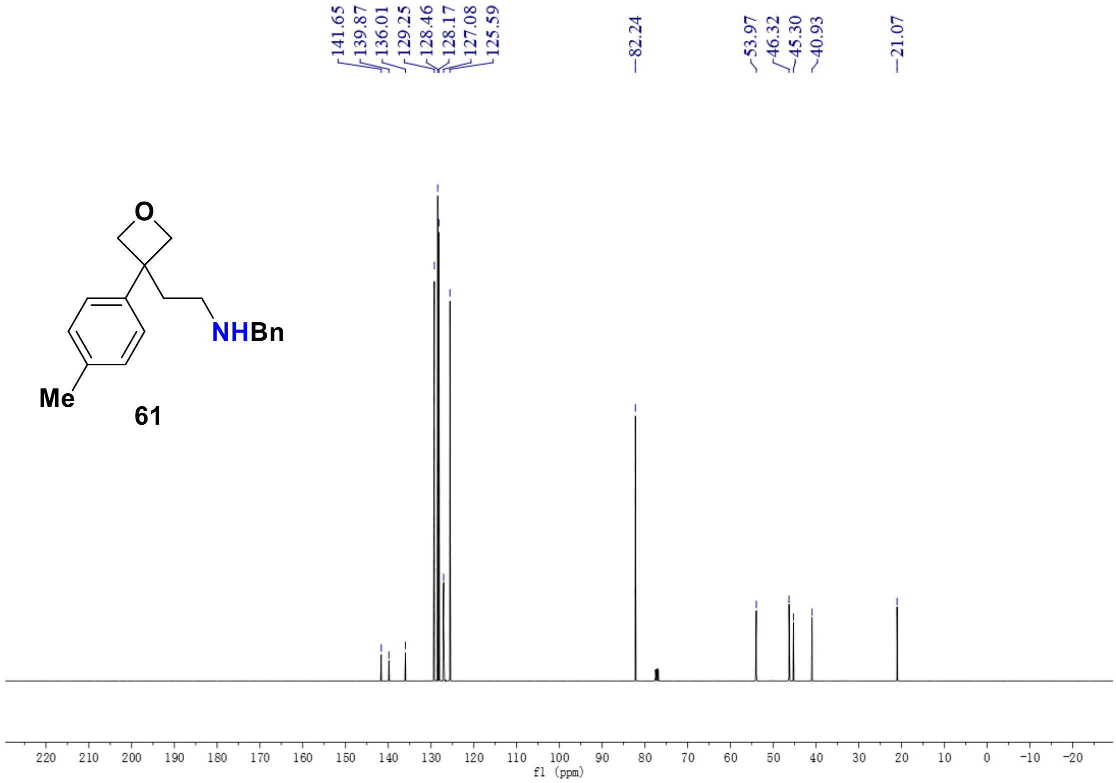

Supplementary Figure 78. ^{13}C NMR (101 MHz, $CDCl_3$) spectrum of **61.**

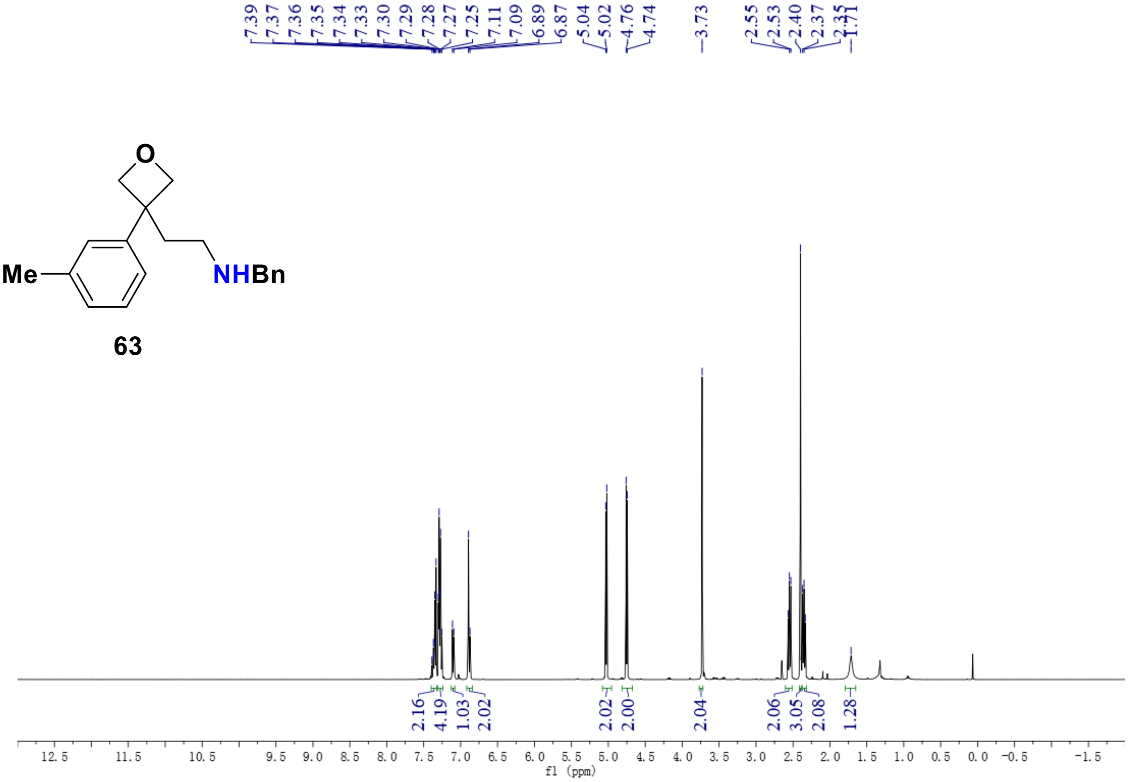

Supplementary Figure 79. **^{1}H NMR (400 MHz, $CDCl_3$)** spectrum of **63.**

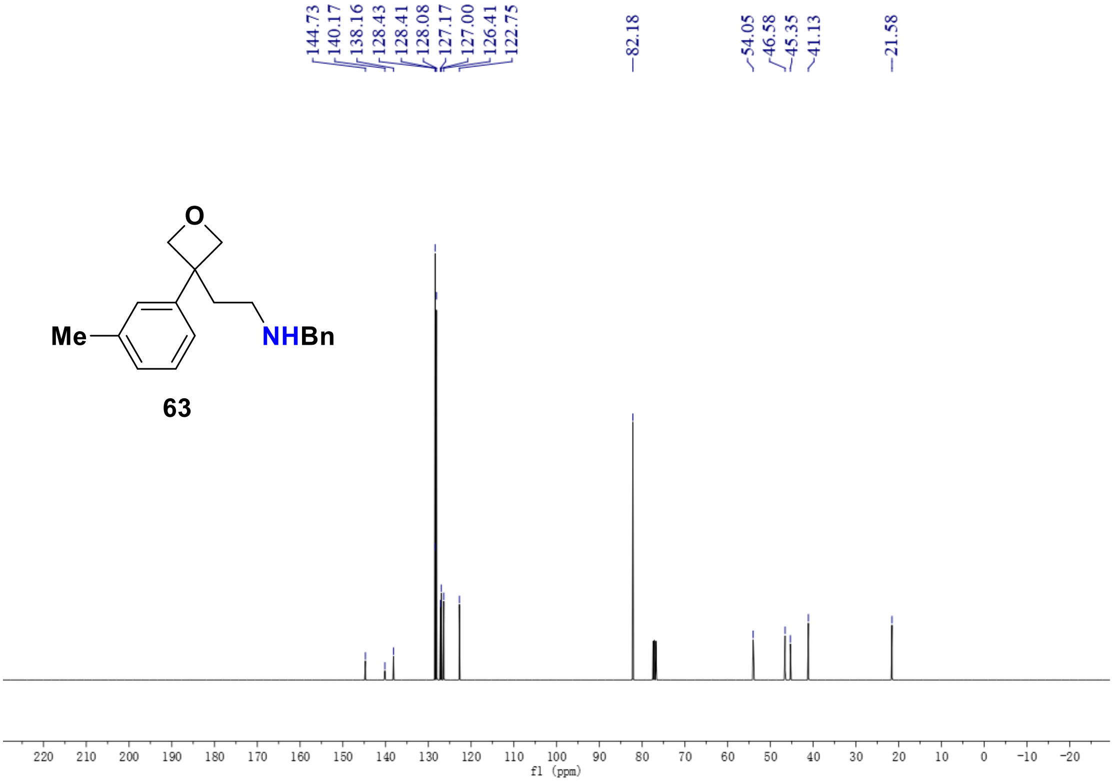

Supplementary Figure 80. **^{13}C NMR (101 MHz, $CDCl_3$)** spectrum of **63.**

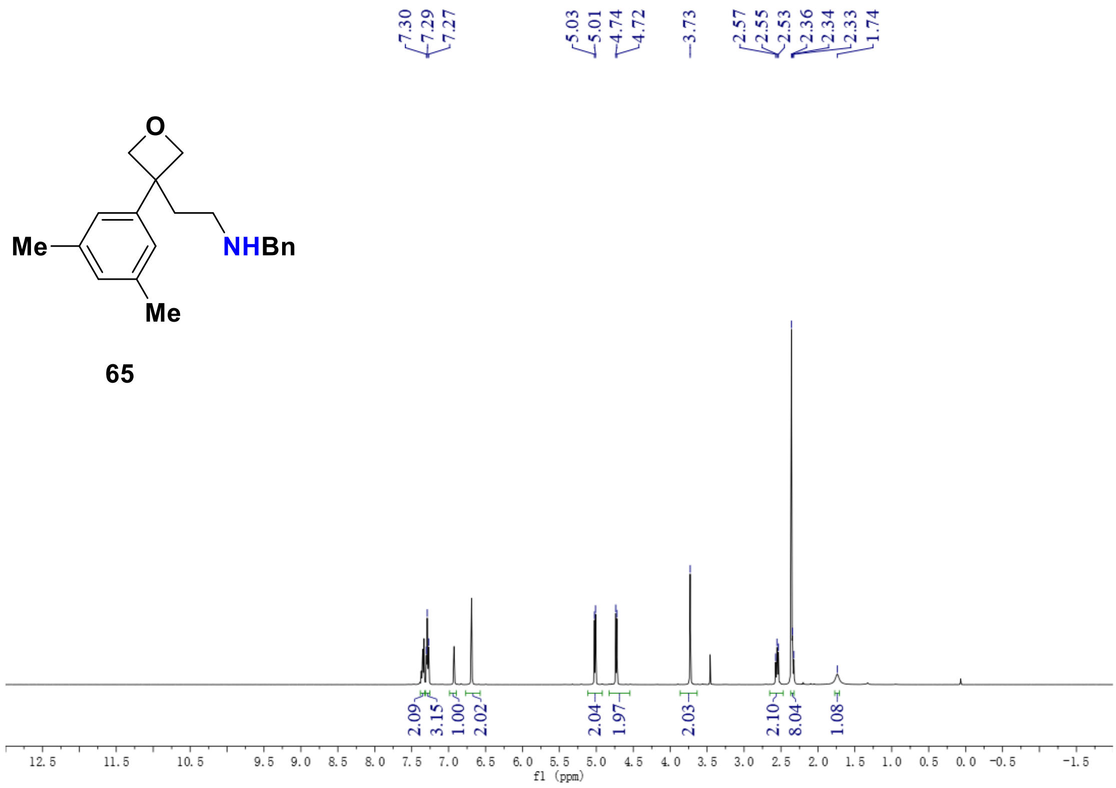

Supplementary Figure 81. ^{1}**H NMR (400 MHz, CDCl$_3$)** spectrum of **65.**

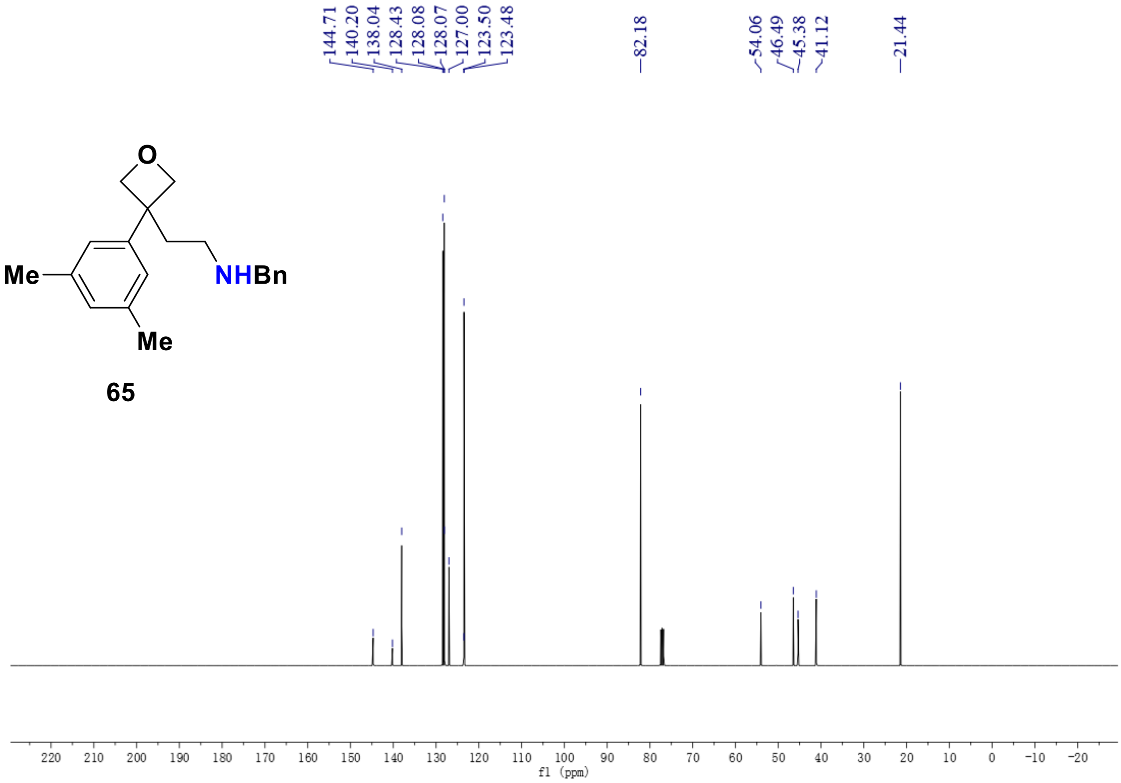

Supplementary Figure 82. ^{13}**C NMR (101 MHz, CDCl$_3$)** spectrum of **65.**

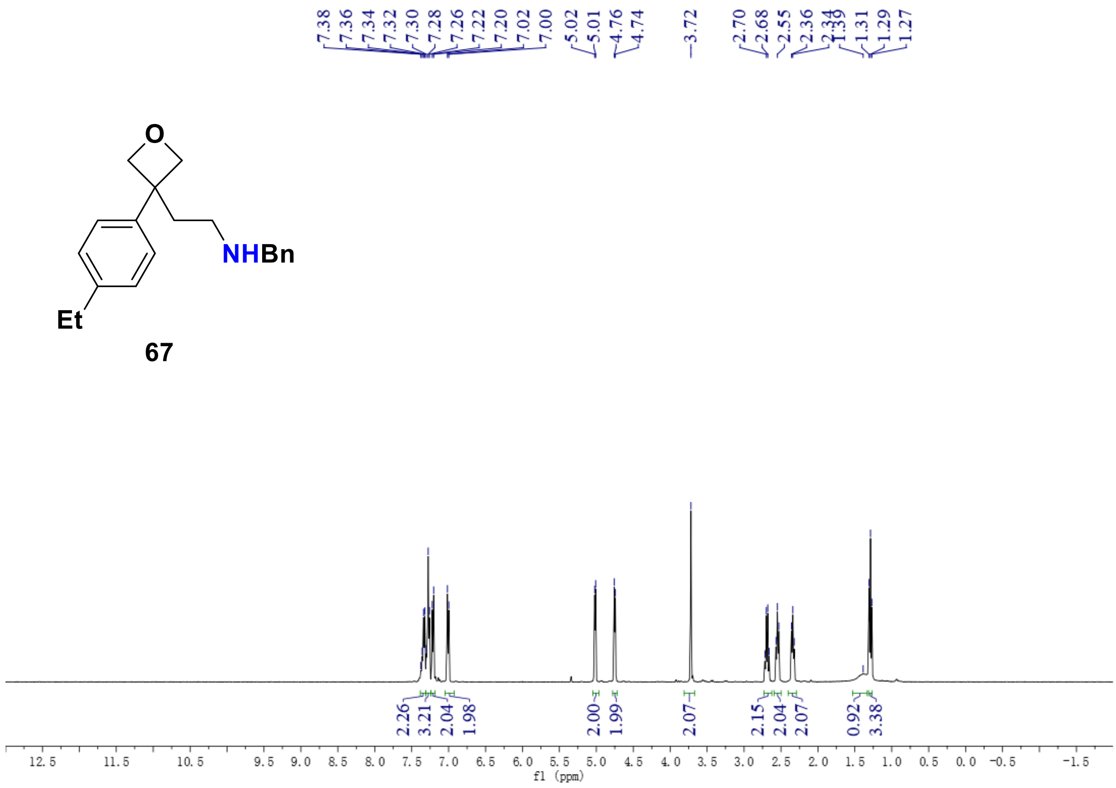

Supplementary Figure 83. ^{1}H NMR (400 MHz, $CDCl_3$) spectrum of 67.

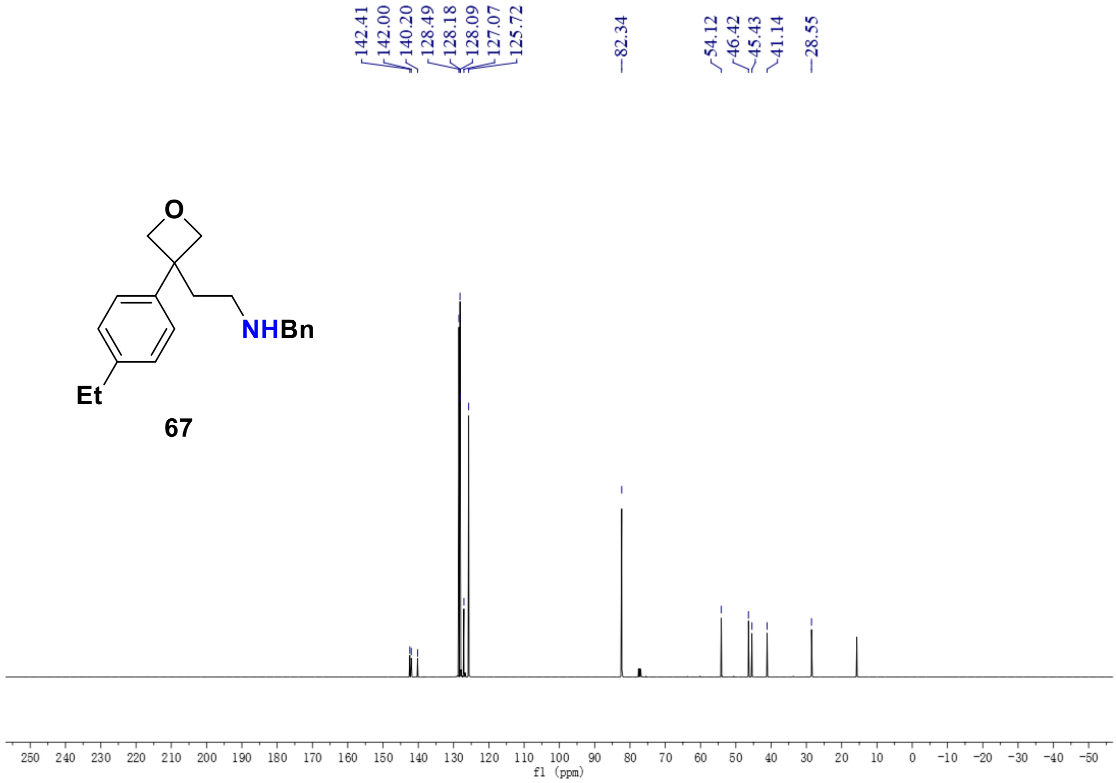

Supplementary Figure 84. ^{13}C NMR (101 MHz, $CDCl_3$) spectrum of 67.

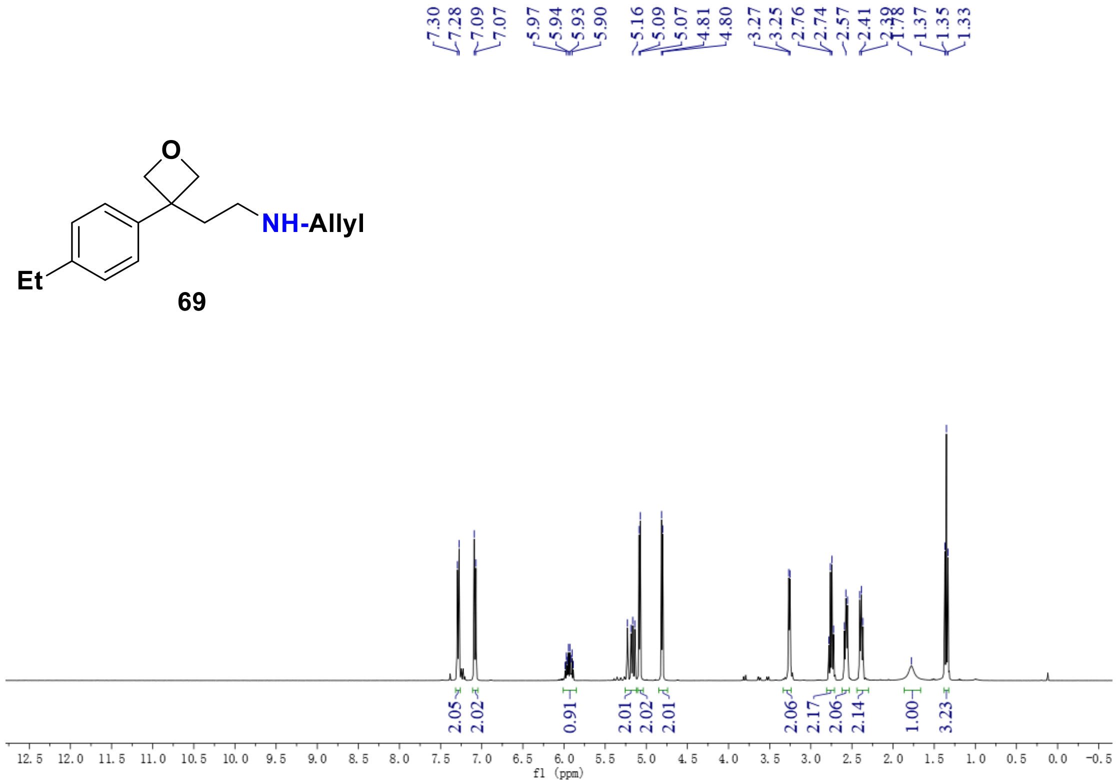

Supplementary Figure 85. **^{1}H NMR (400 MHz, $CDCl_3$)** spectrum of **69.**

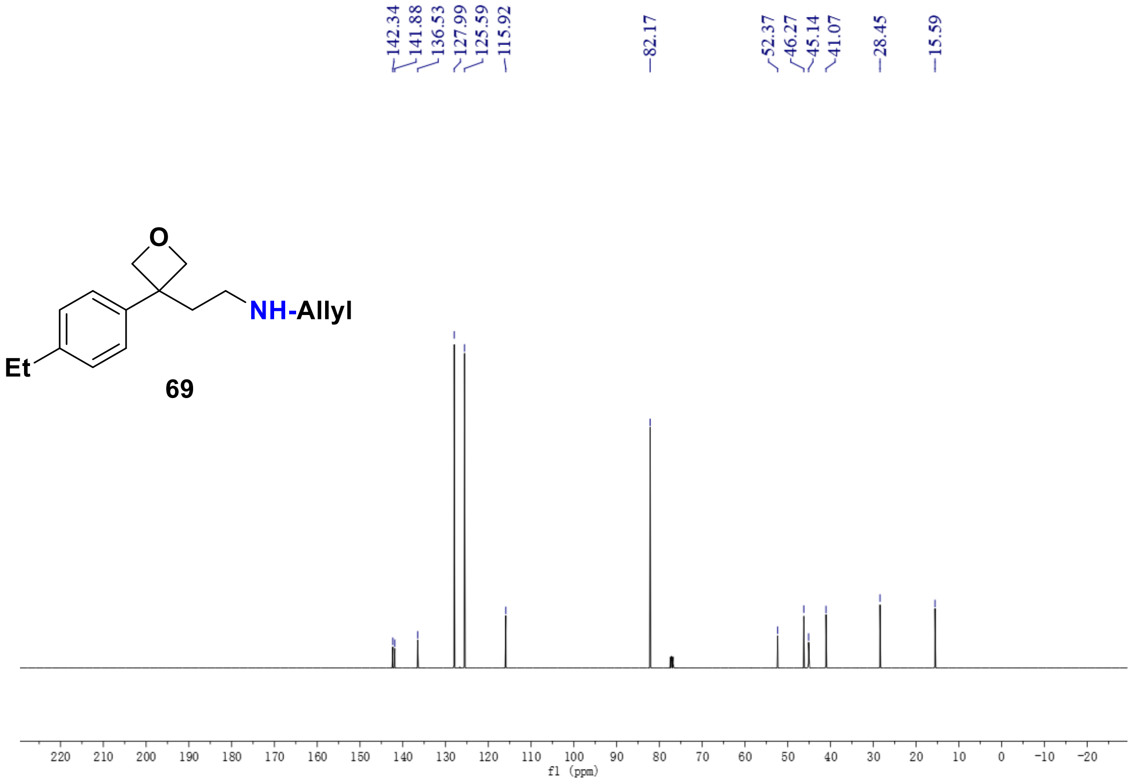

Supplementary Figure 86. **^{13}C NMR (101 MHz, $CDCl_3$)** spectrum of **69.**

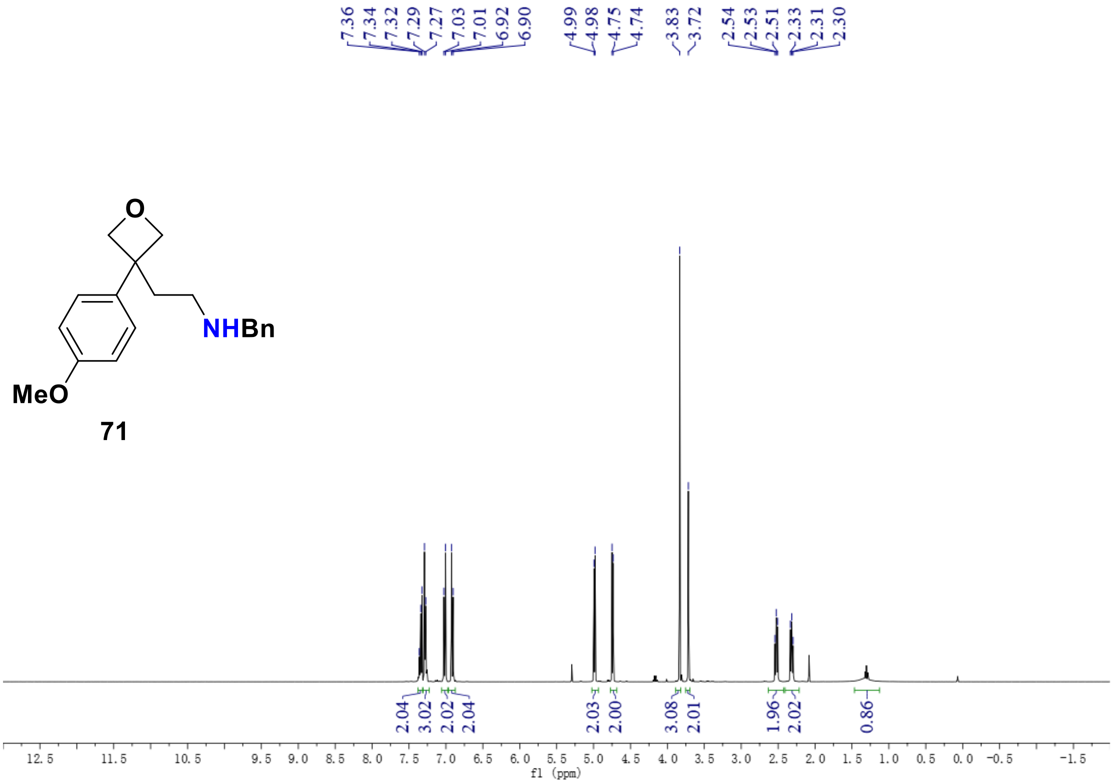

Supplementary Figure 87. ^{1}**H NMR (400 MHz, CDCl$_3$)** spectrum of **71.**

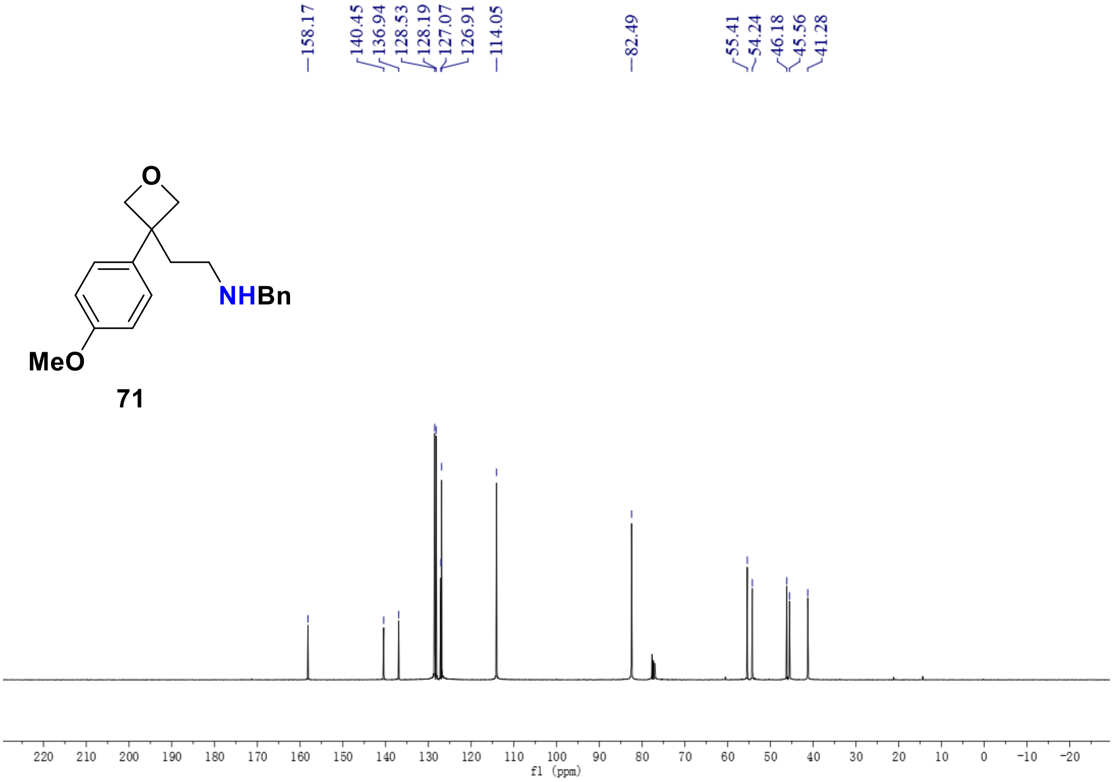

Supplementary Figure 88. ^{13}**C NMR (101 MHz, CDCl$_3$)** spectrum of **71.**

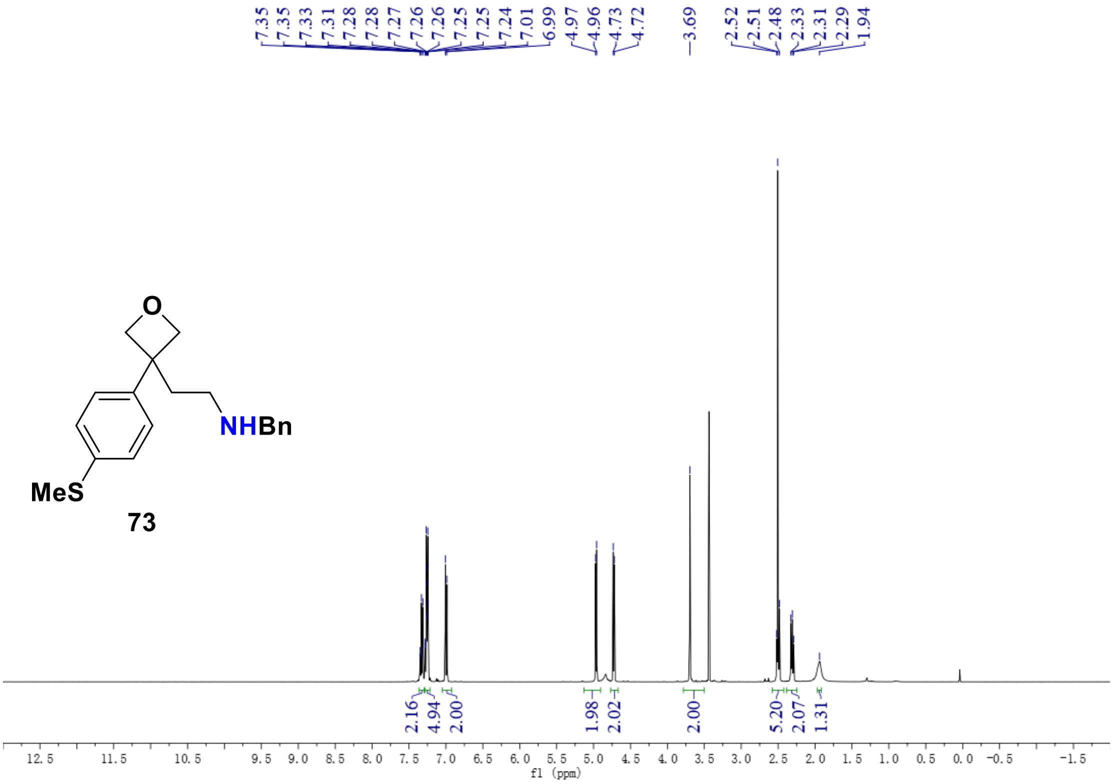

Supplementary Figure 89. **^{1}H NMR (400 MHz, $CDCl_3$)** spectrum of **73.**

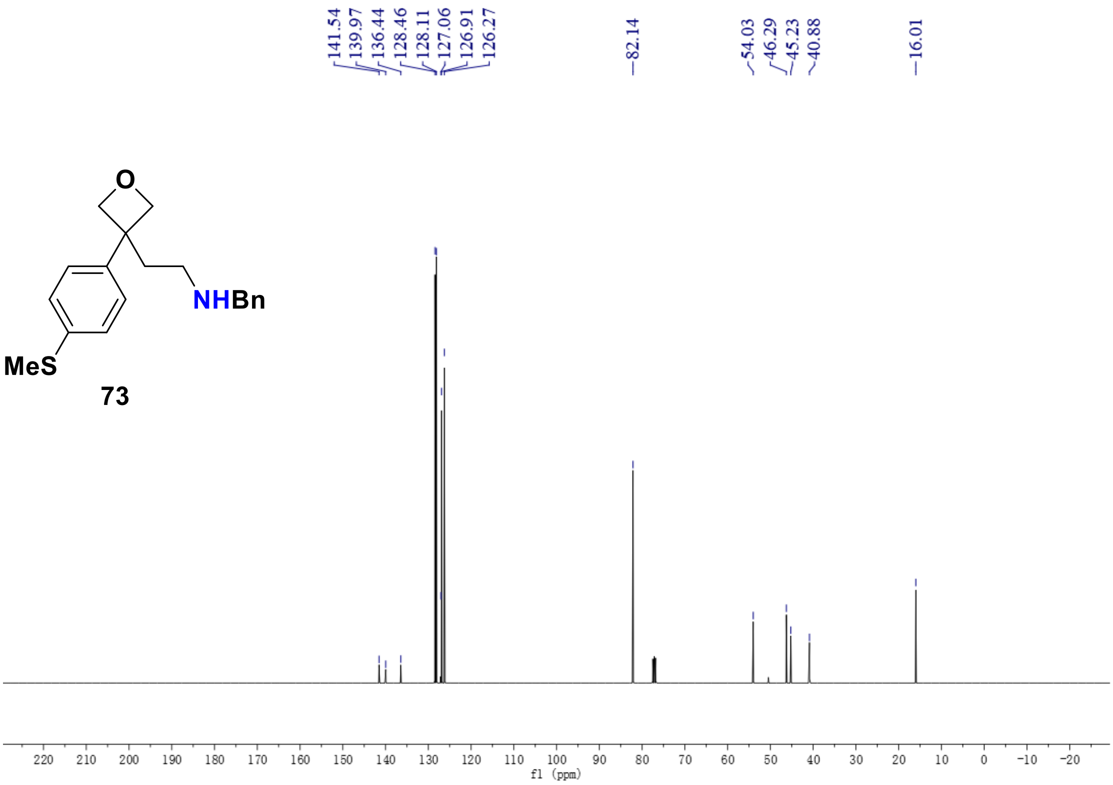

Supplementary Figure 90. **^{13}C NMR (101 MHz, $CDCl_3$)** spectrum of **73.**

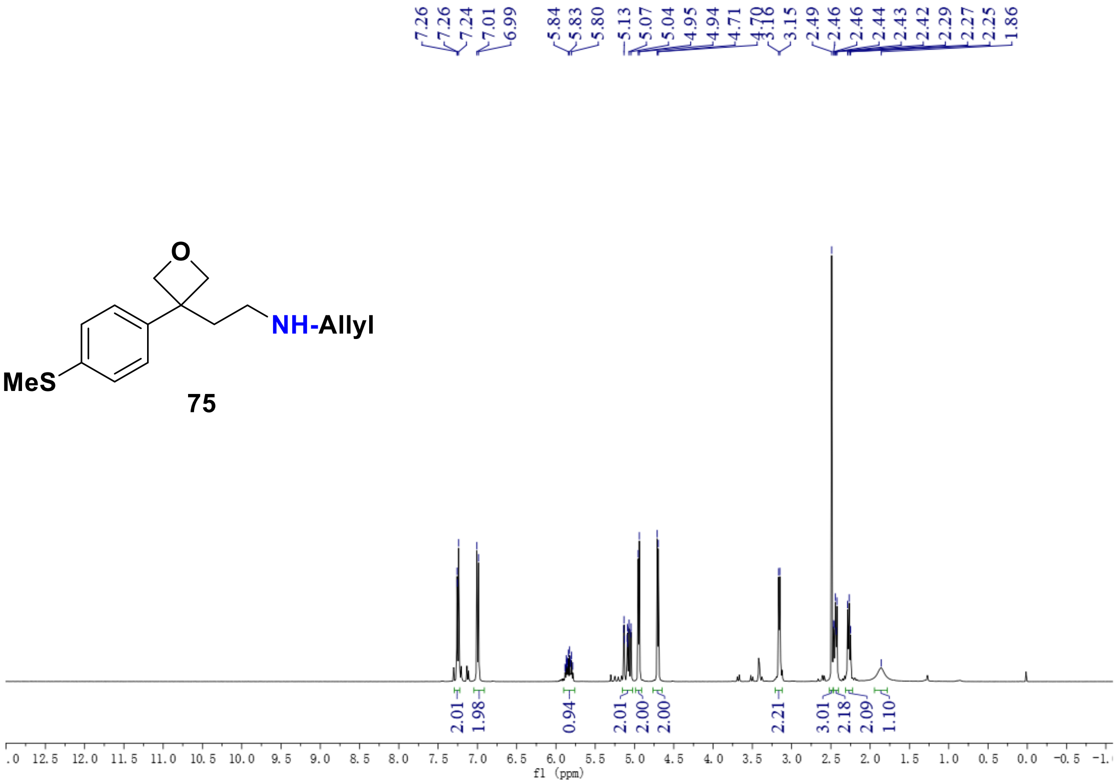

Supplementary Figure 91. ¹H NMR (400 MHz, CDCl₃) spectrum of **75.**

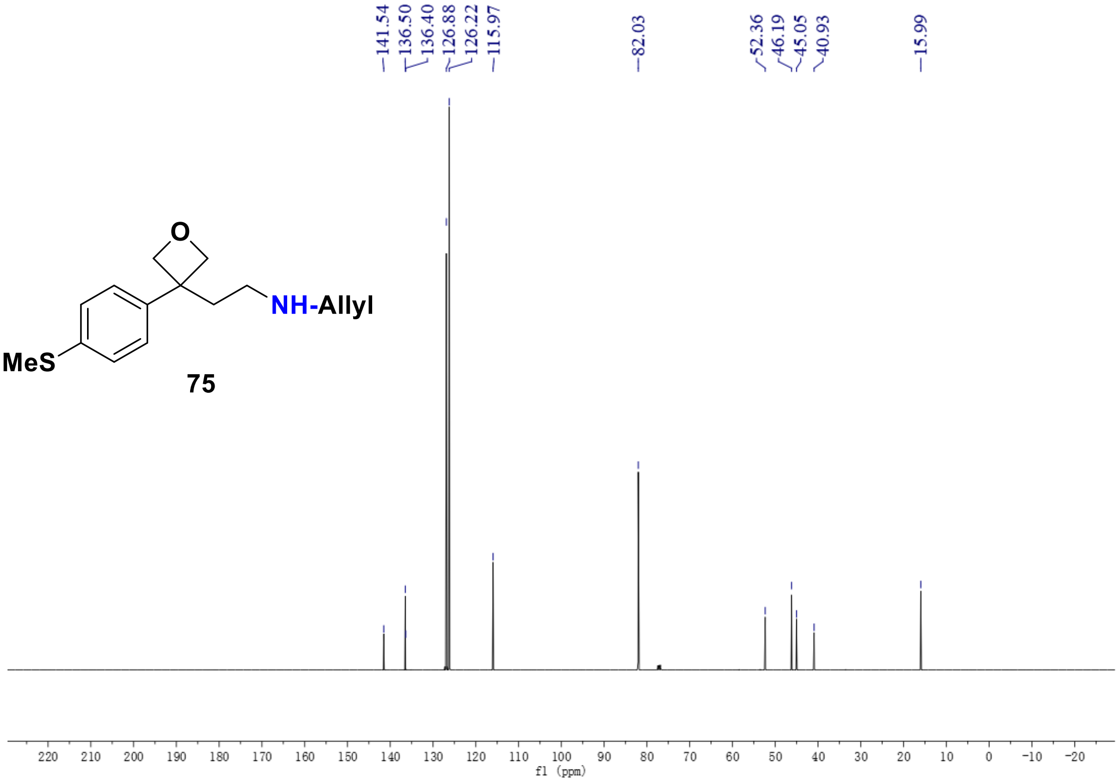

Supplementary Figure 92. ¹³C NMR (101 MHz, CDCl₃) spectrum of **75.**

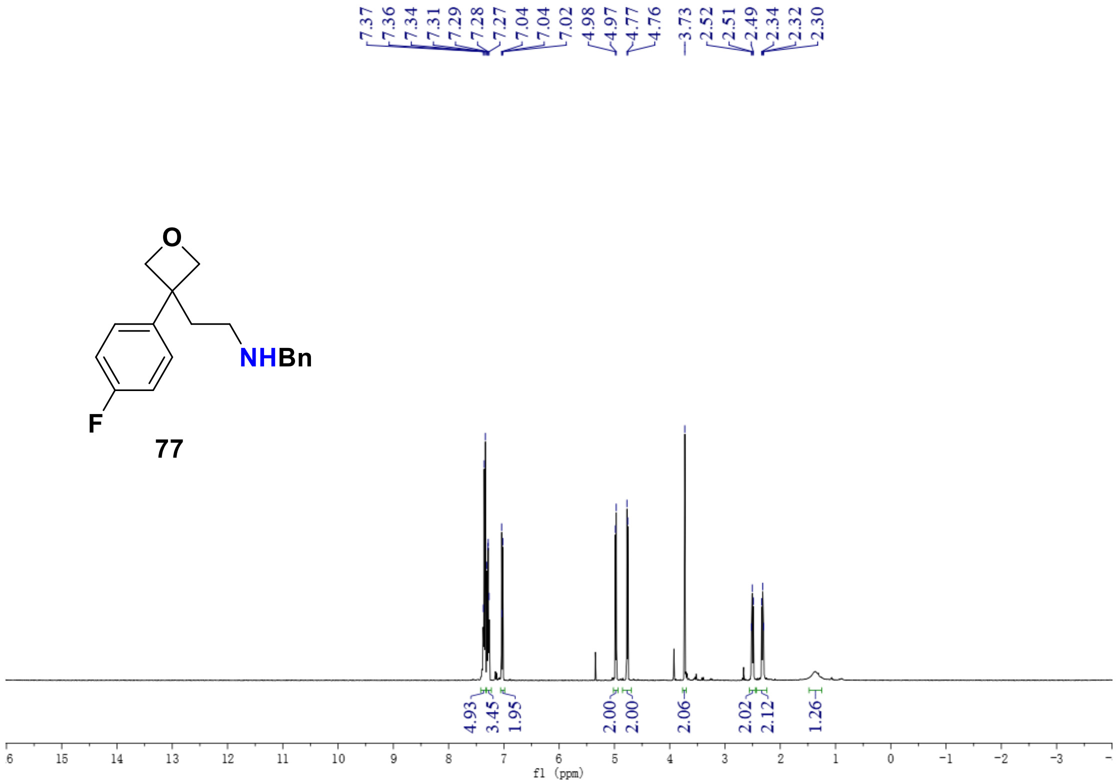

Supplementary Figure 93. ^{1}H NMR (400 MHz, $CDCl_3$) spectrum of **77.**

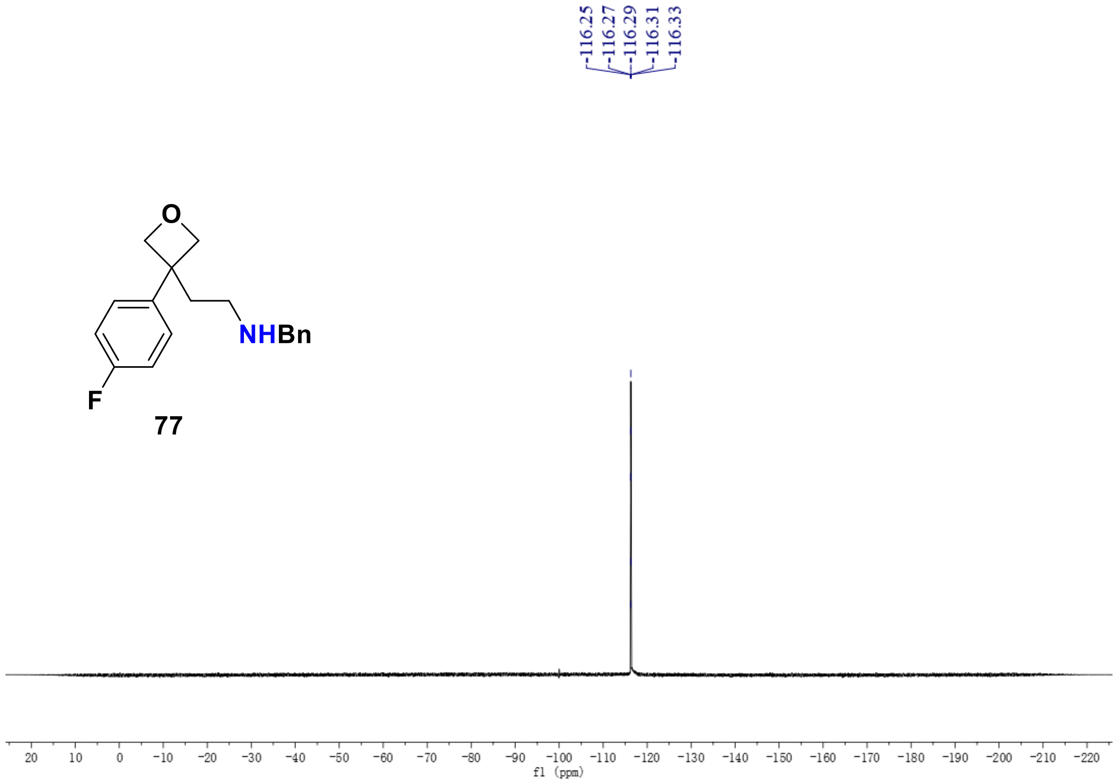

Supplementary Figure 94. ^{19}F NMR (376 MHz, $CDCl_3$) spectrum of **77.**

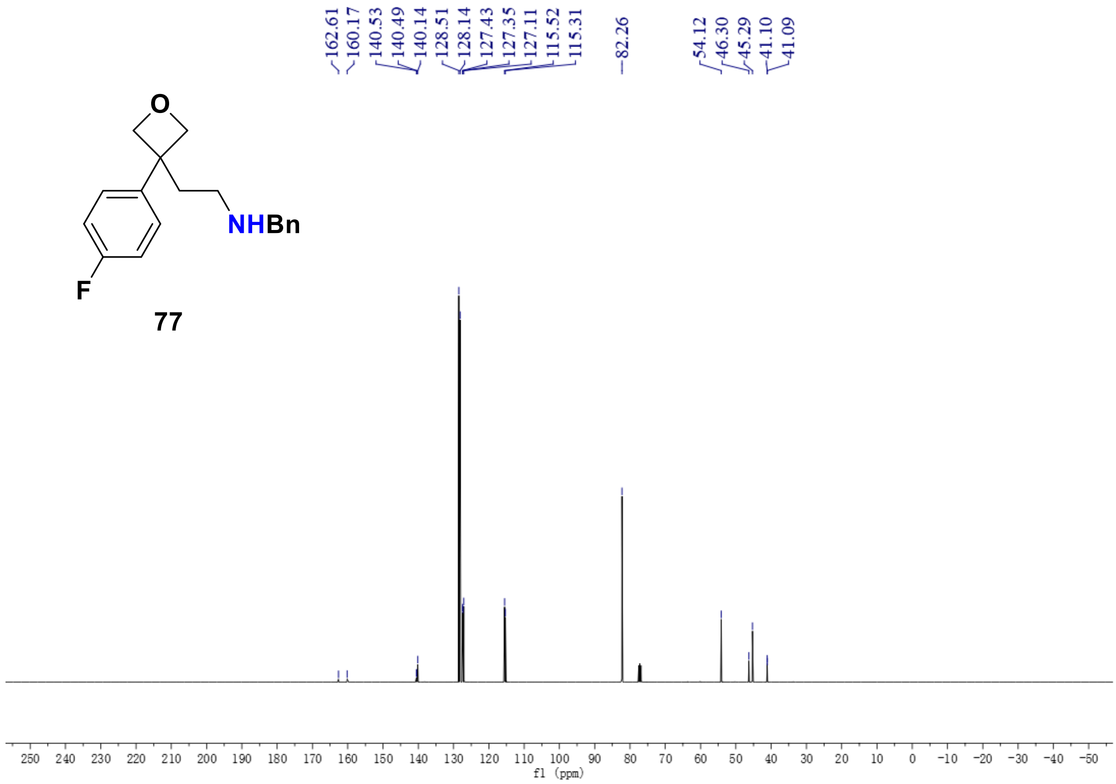

Supplementary Figure 95. ^{13}C NMR (101 MHz, CDCl$_3$) spectrum of **77**.

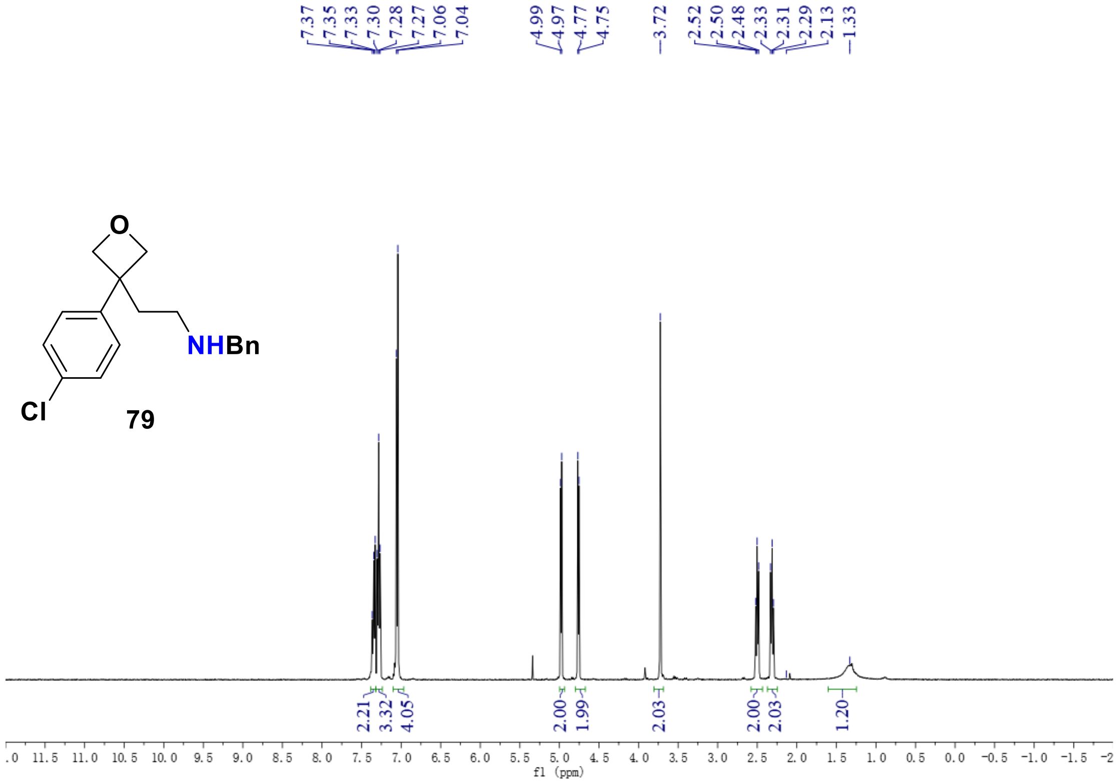

Supplementary Figure 96. ^{1}H NMR (400 MHz, CDCl$_3$) spectrum of **79**.

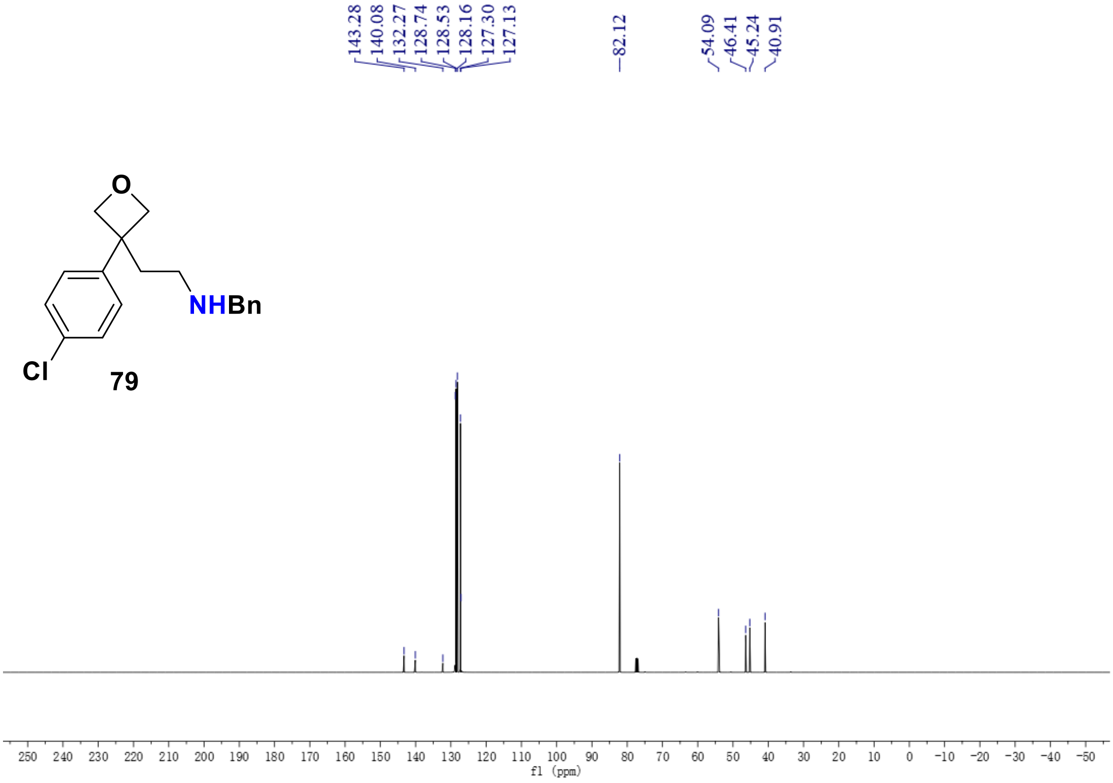

Supplementary Figure 97. ^{13}**C NMR (101 MHz, $CDCl_3$)** spectrum of **79**.

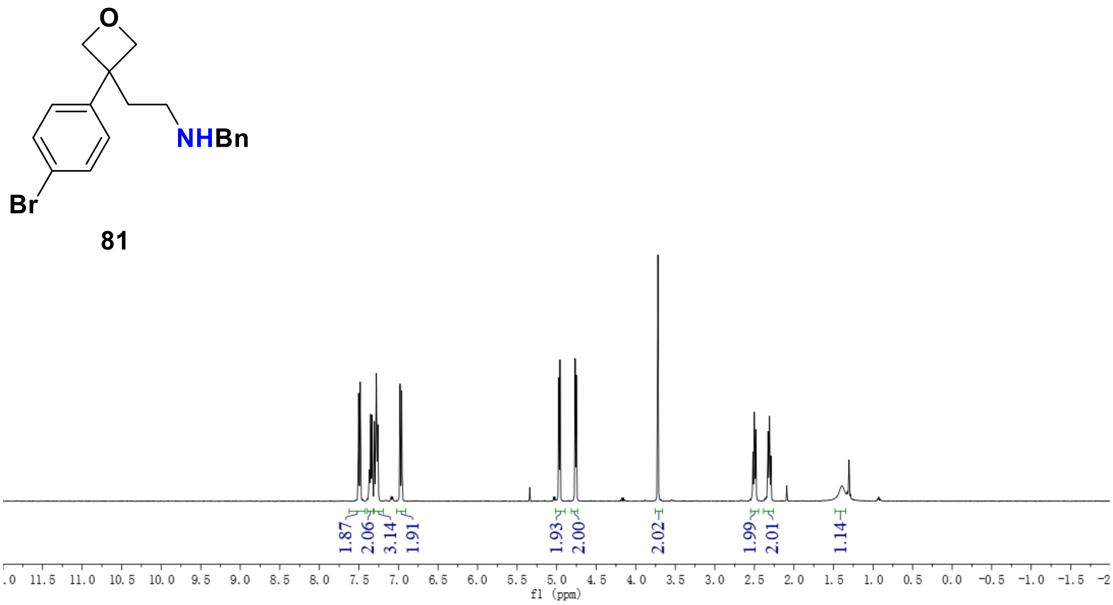

Supplementary Figure 98. ^{1}**H NMR (400 MHz, $CDCl_3$)** spectrum of **81.**

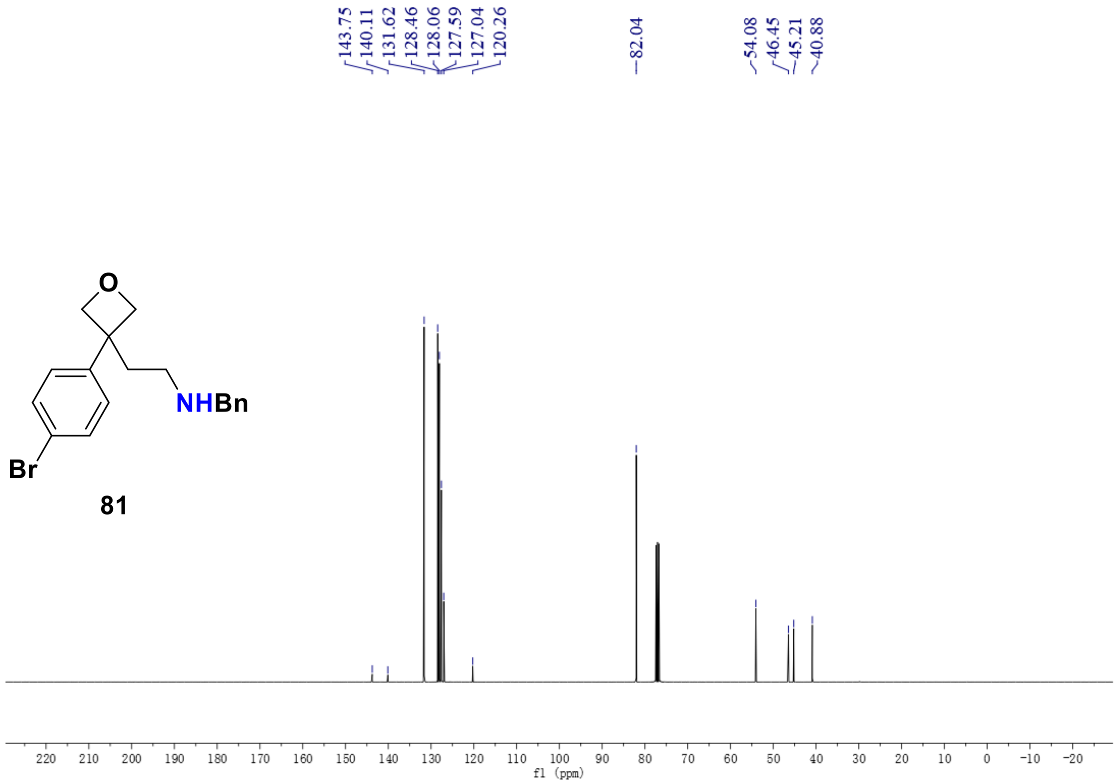

Supplementary Figure 99. ^{13}C NMR (101 MHz, $CDCl_3$) spectrum of **81.**

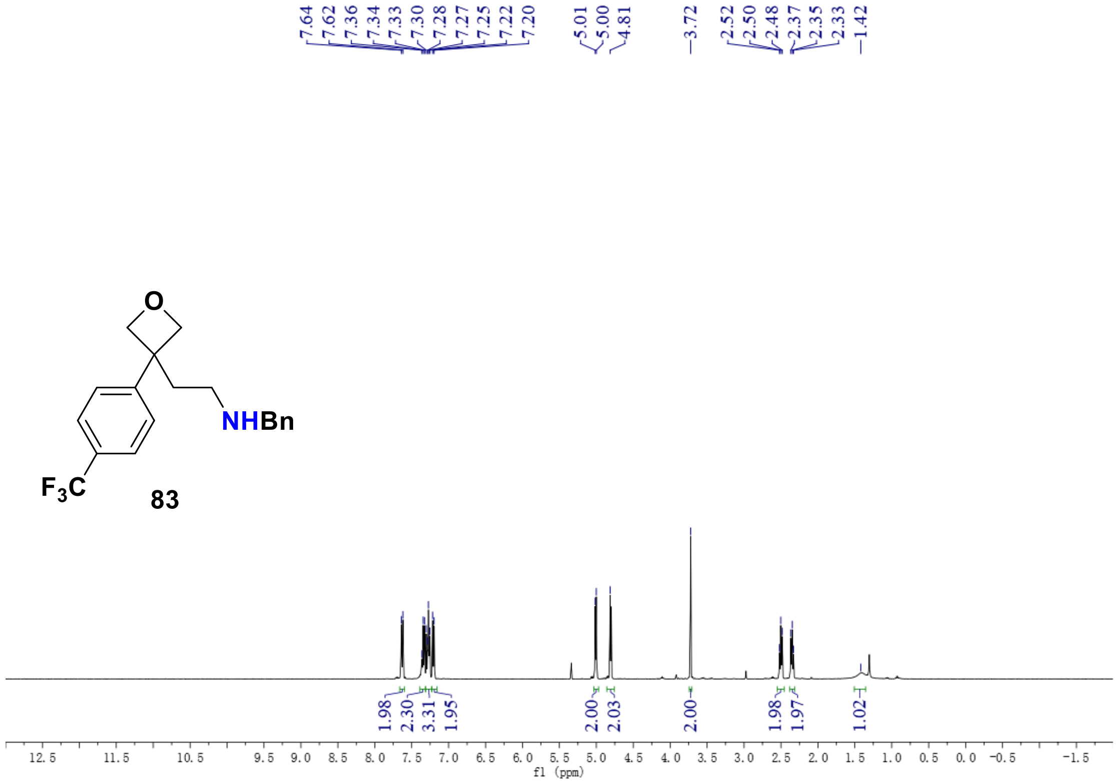

Supplementary Figure 100. ^{1}H NMR (400 MHz, $CDCl_3$) spectrum of **83.**

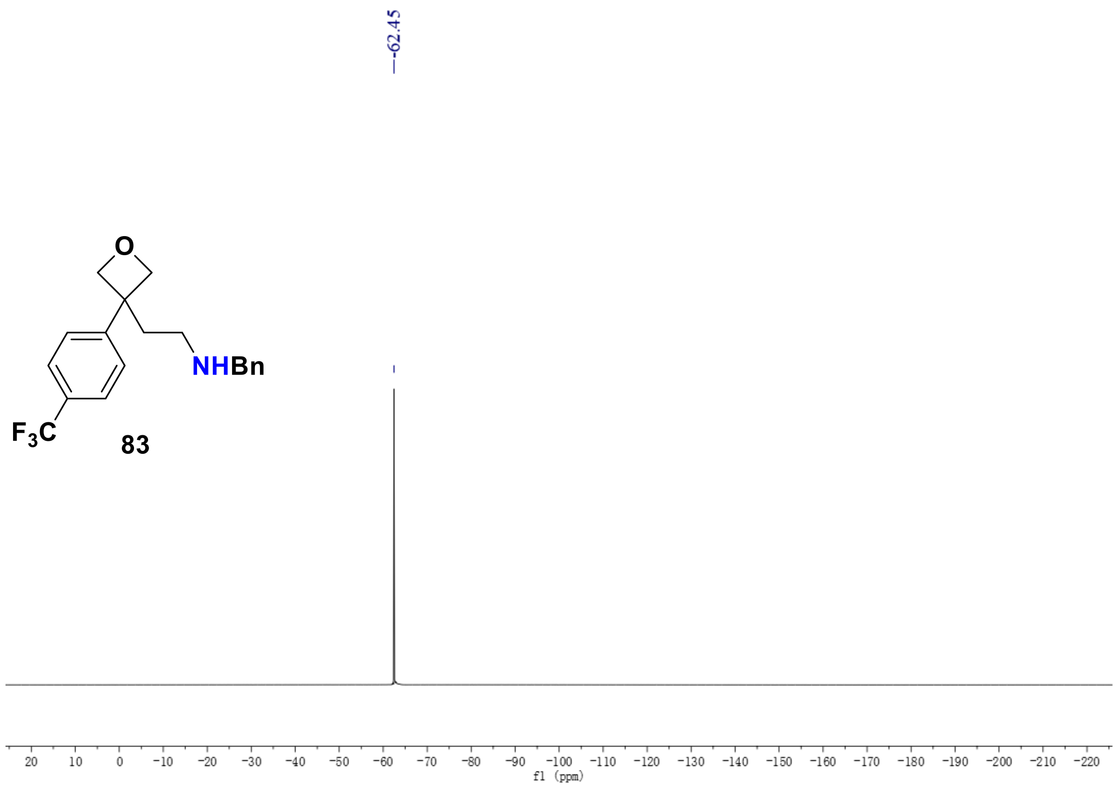

Supplementary Figure 101. **^{19}F NMR (400 MHz, $CDCl_3$)** spectrum of **83.**

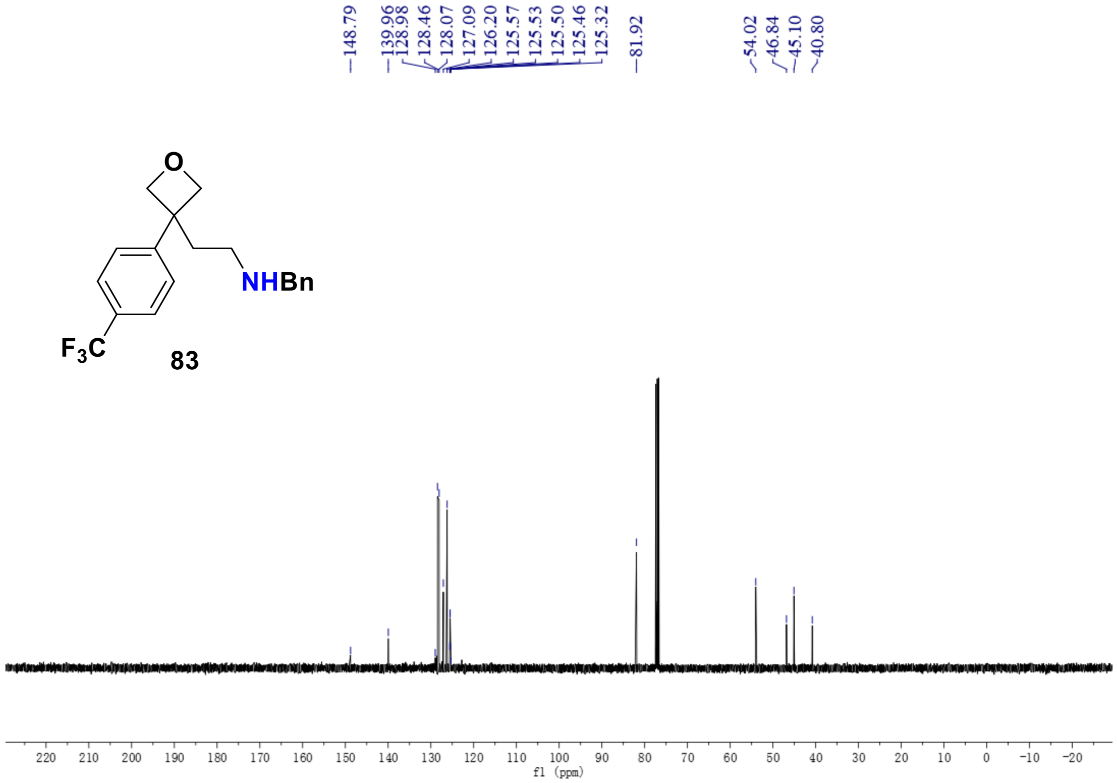

Supplementary Figure 102. **^{13}C NMR (101 MHz, $CDCl_3$)** spectrum of **83.**

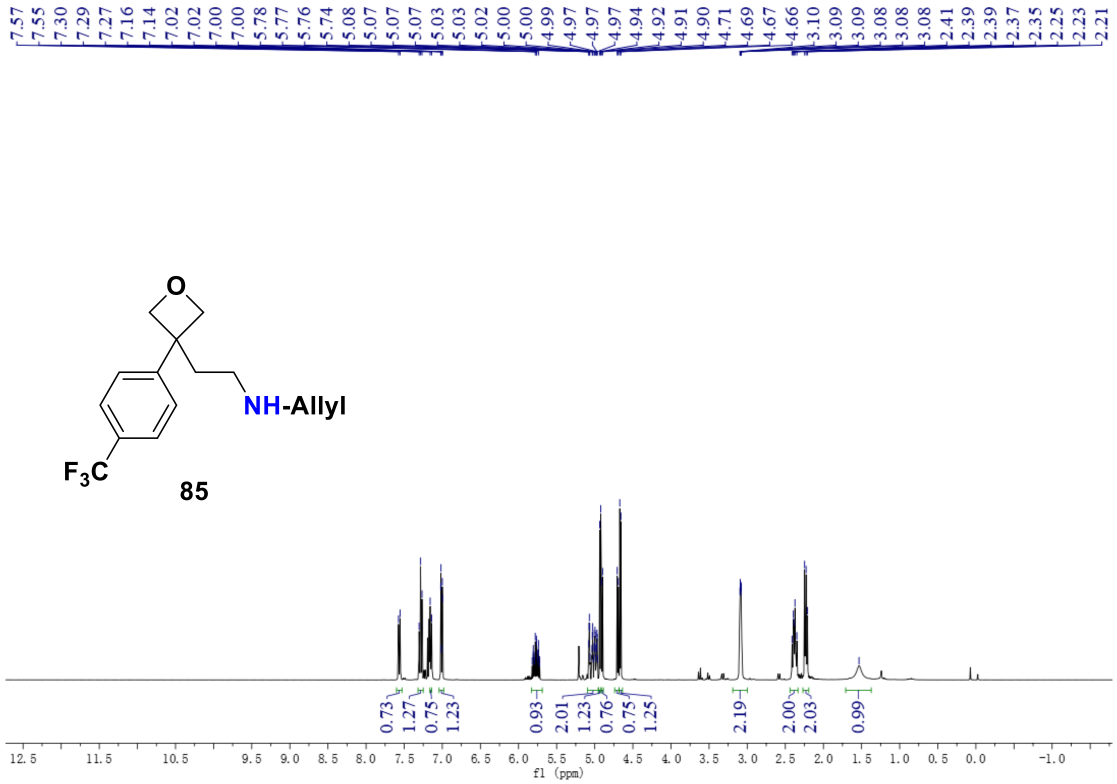

Supplementary Figure 103. ^{1}**H NMR (400 MHz, CDCl₃)** spectrum of **85.**

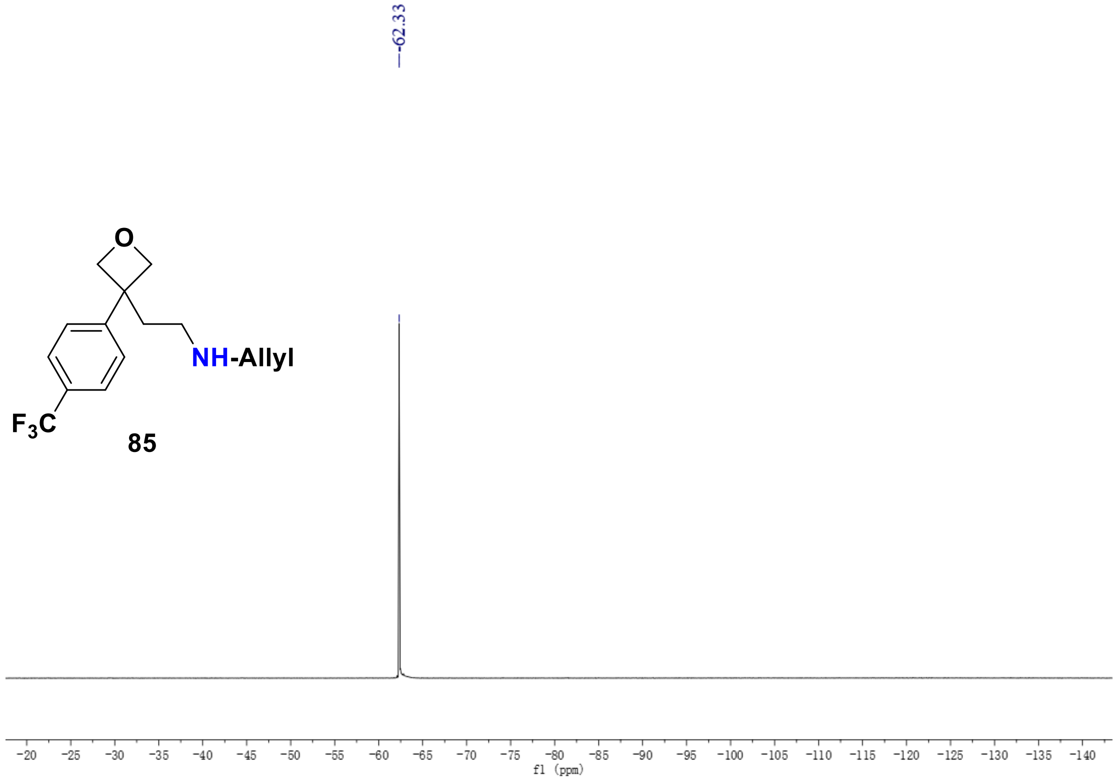

Supplementary Figure 104. ^{13}**F NMR (376 MHz, CDCl₃)** spectrum of **85.**

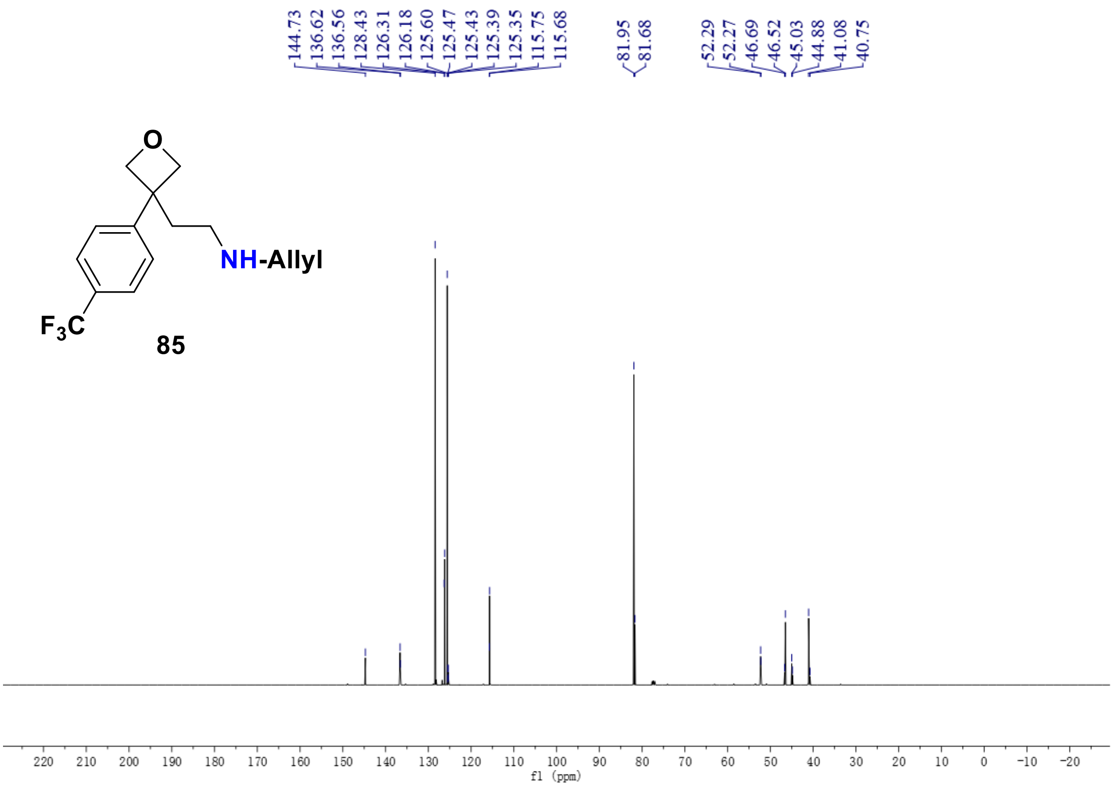

Supplementary Figure 105. ^{13}**C NMR (101 MHz,** $CDCl_3$**)** spectrum of **85.**

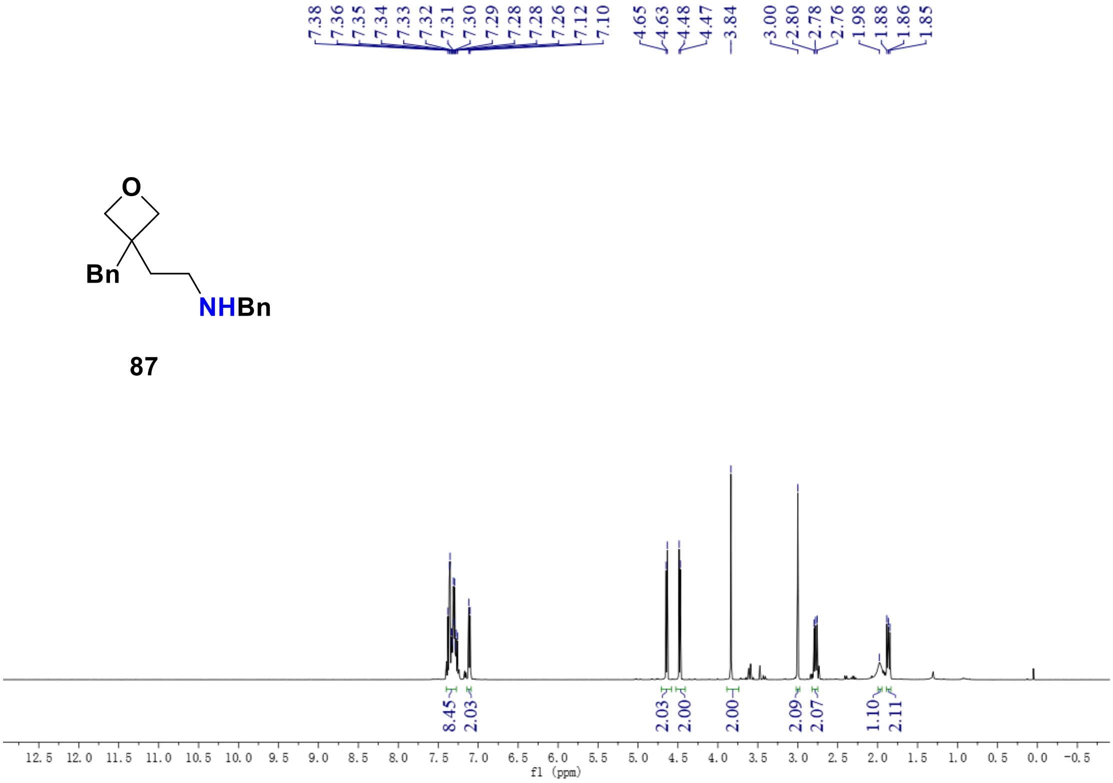

Supplementary Figure 106. ^{1}**H NMR (400 MHz,** $CDCl_3$**)** spectrum of **87.**

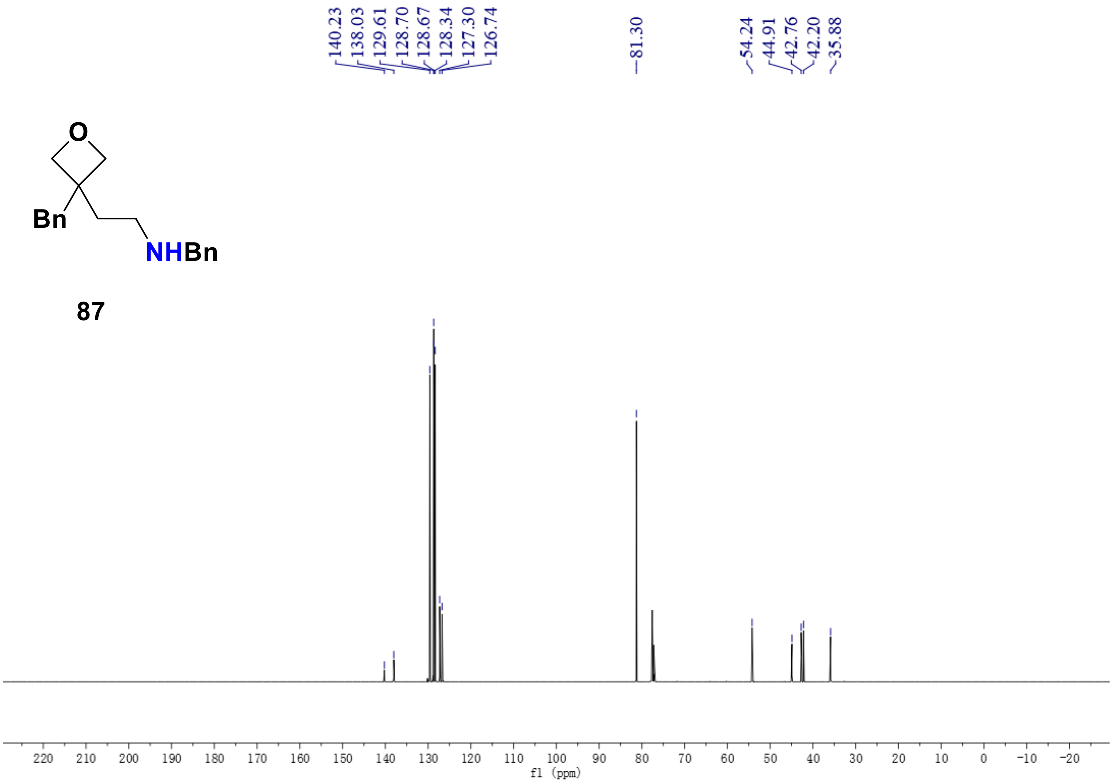

Supplementary Figure 107. ^{13}C NMR (101 MHz, CDCl$_3$) spectrum of **87.**

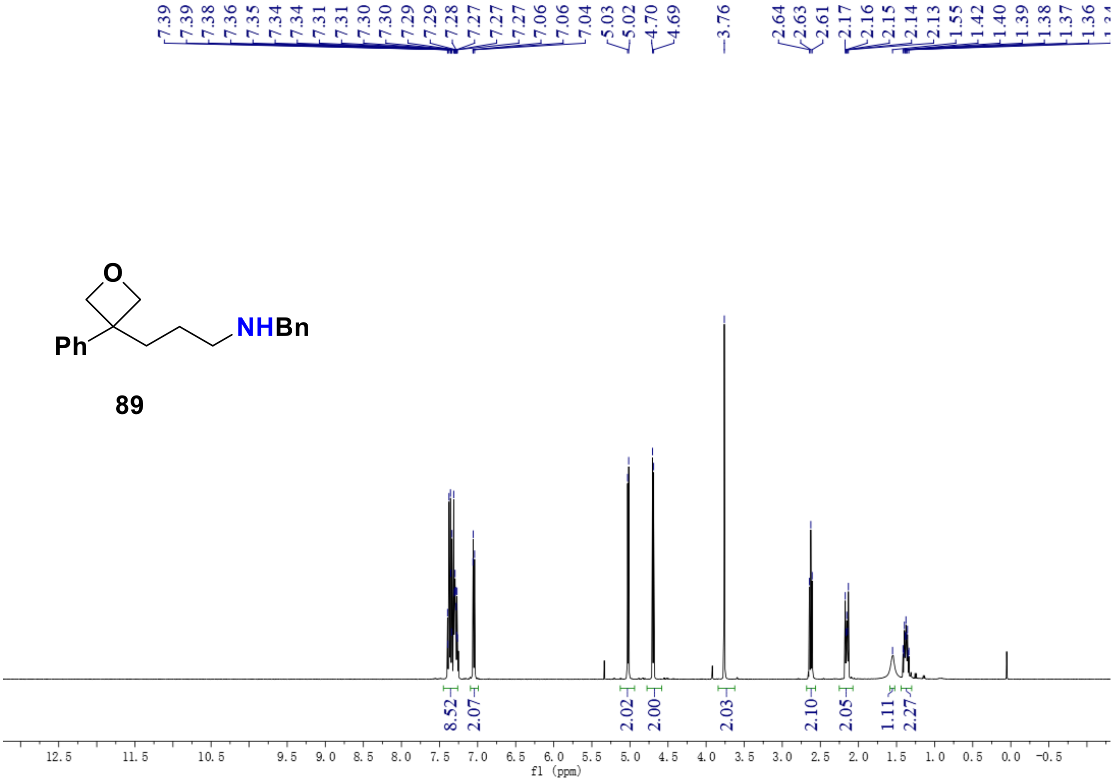

Supplementary Figure 108. ^{1}H NMR (400 MHz, CDCl$_3$) spectrum of **89.**

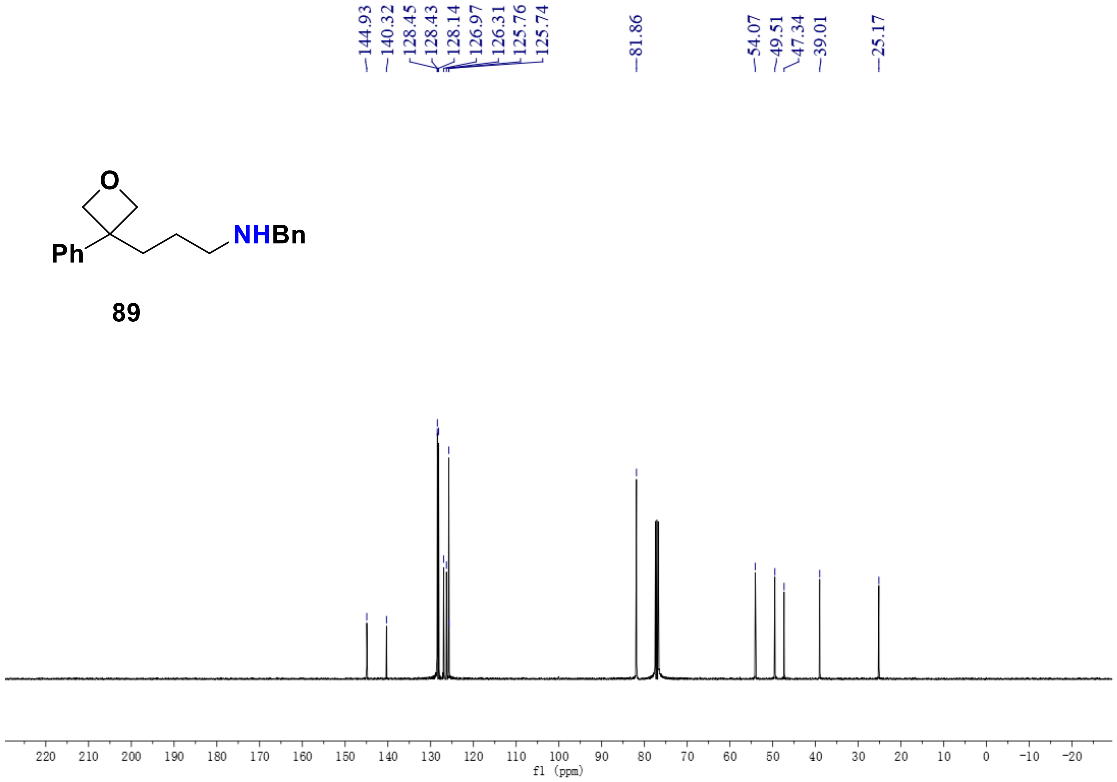

Supplementary Figure 109. ^{13}**C NMR (101 MHz, CDCl$_3$)** spectrum of **89.**

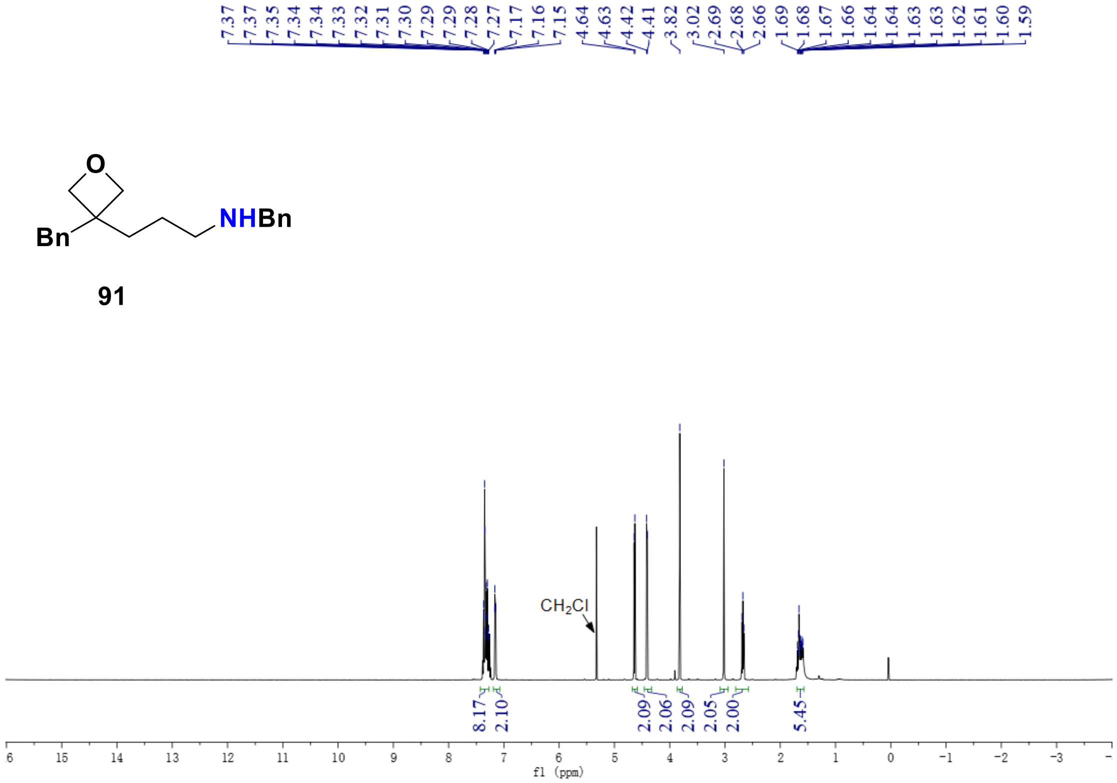

Supplementary Figure 110. ^{1}**H NMR (400 MHz, CDCl$_3$)** spectrum of **91.**

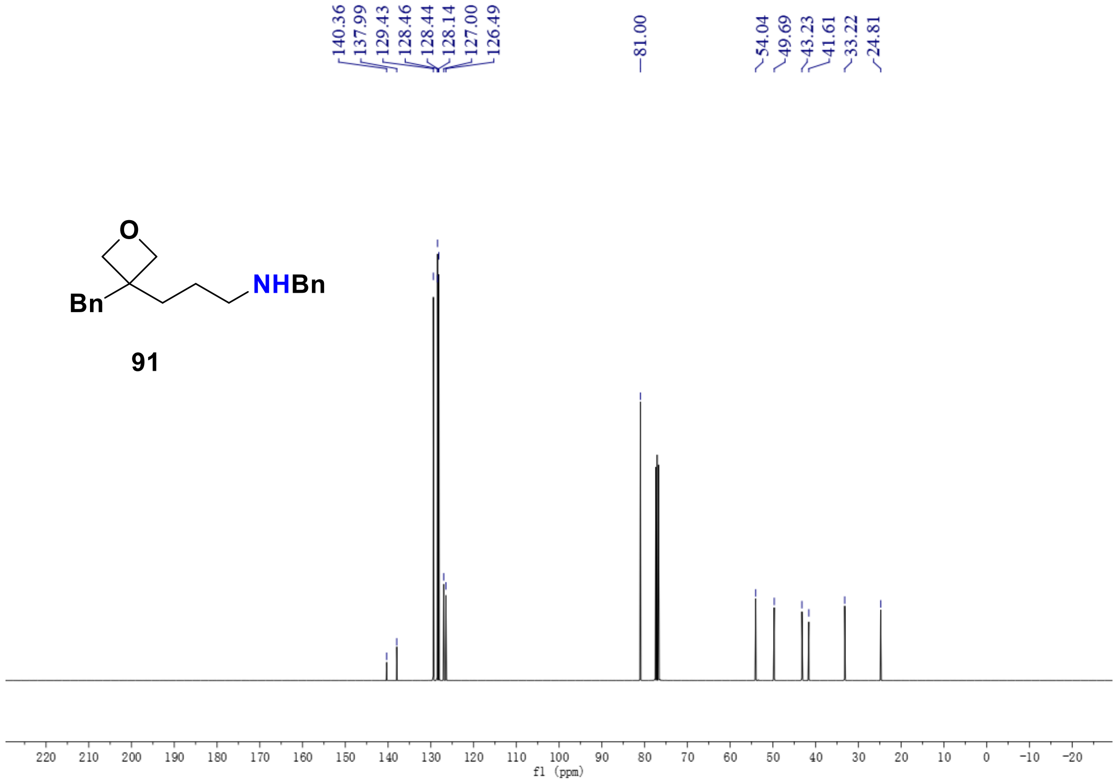

Supplementary Figure 111. ^{13}C NMR (101 MHz, $CDCl_3$) spectrum of **91.**

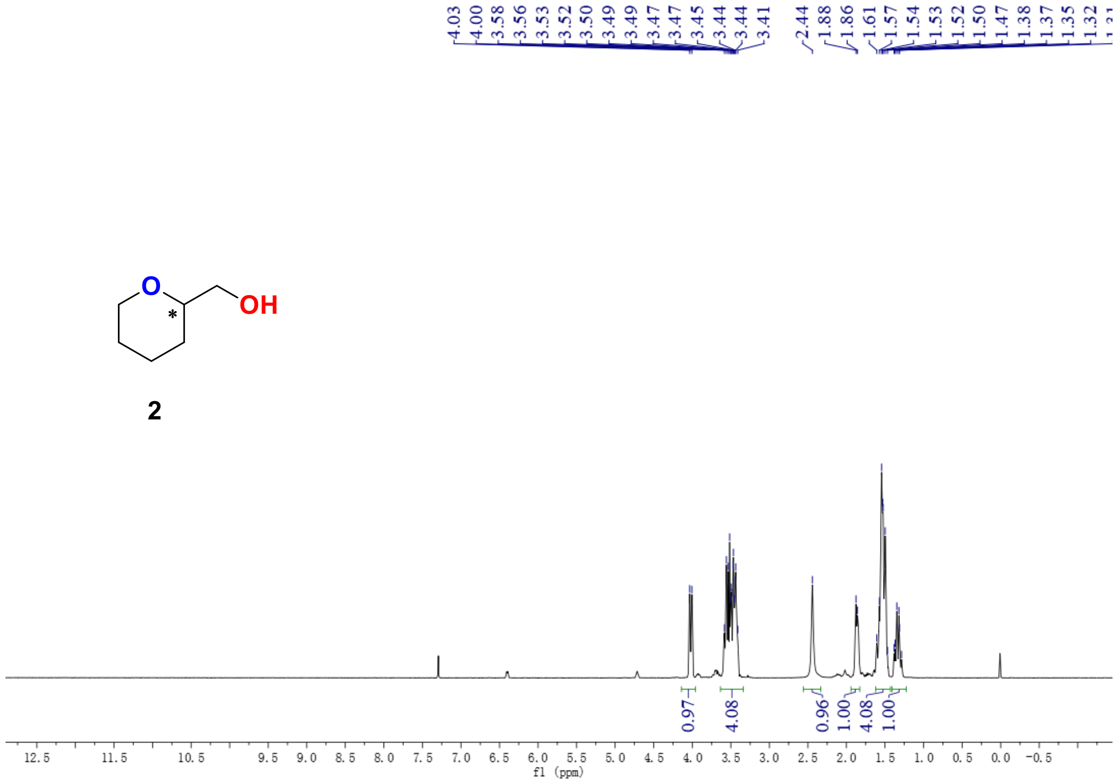

Supplementary Figure 112. ^{1}H NMR spectrum (400 MHz, $CDCl_3$) of 2.

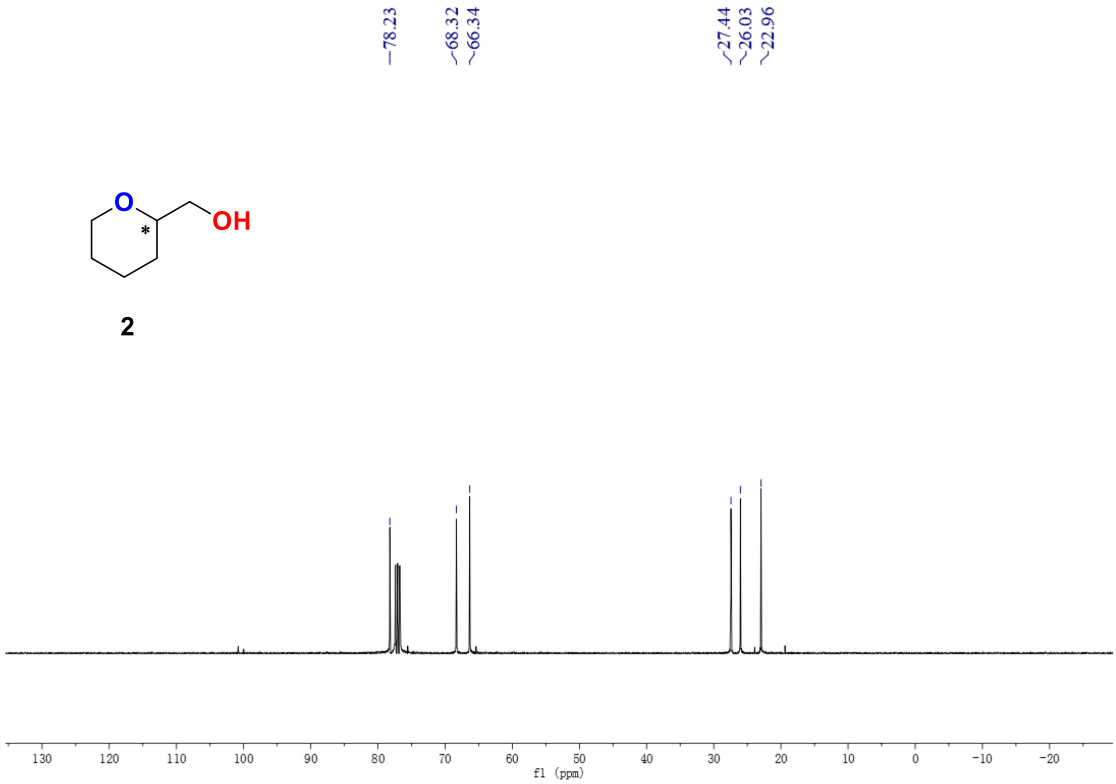

Supplementary Figure 113. ^{13}C NMR (101 MHz, $CDCl_3$) spectrum of **2.**

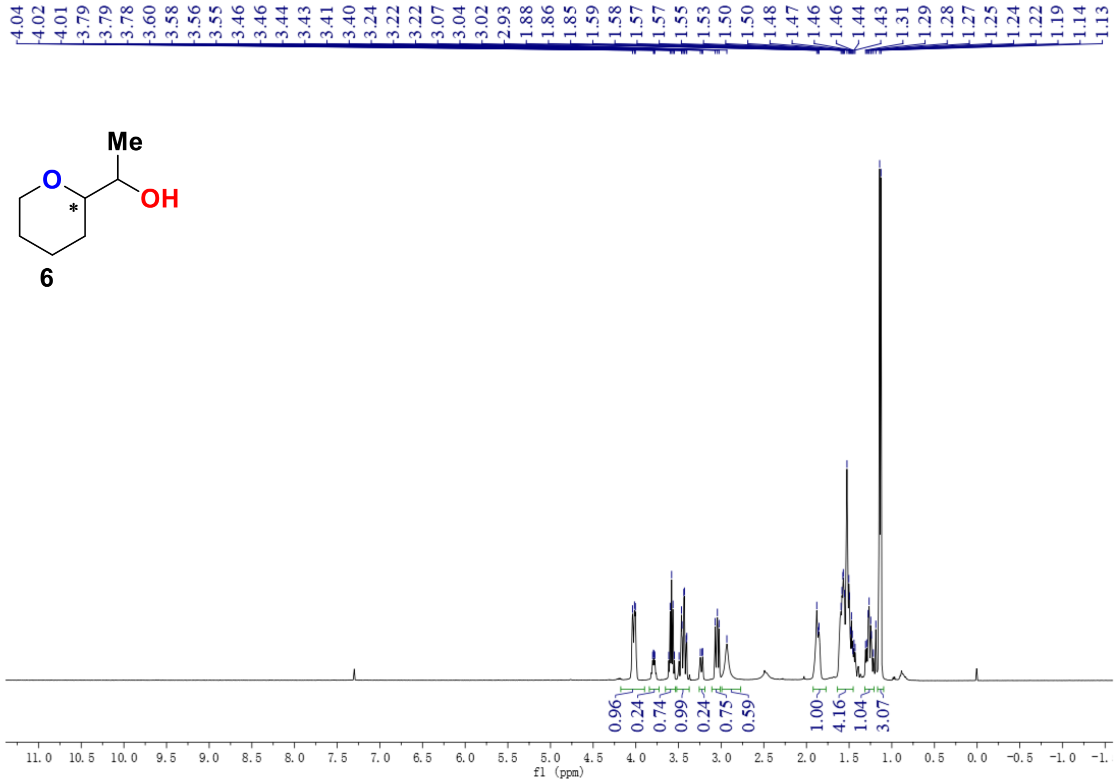

Supplementary Figure 114. ^{1}H NMR (400 MHz, $CDCl_3$) spectrum of **6.**

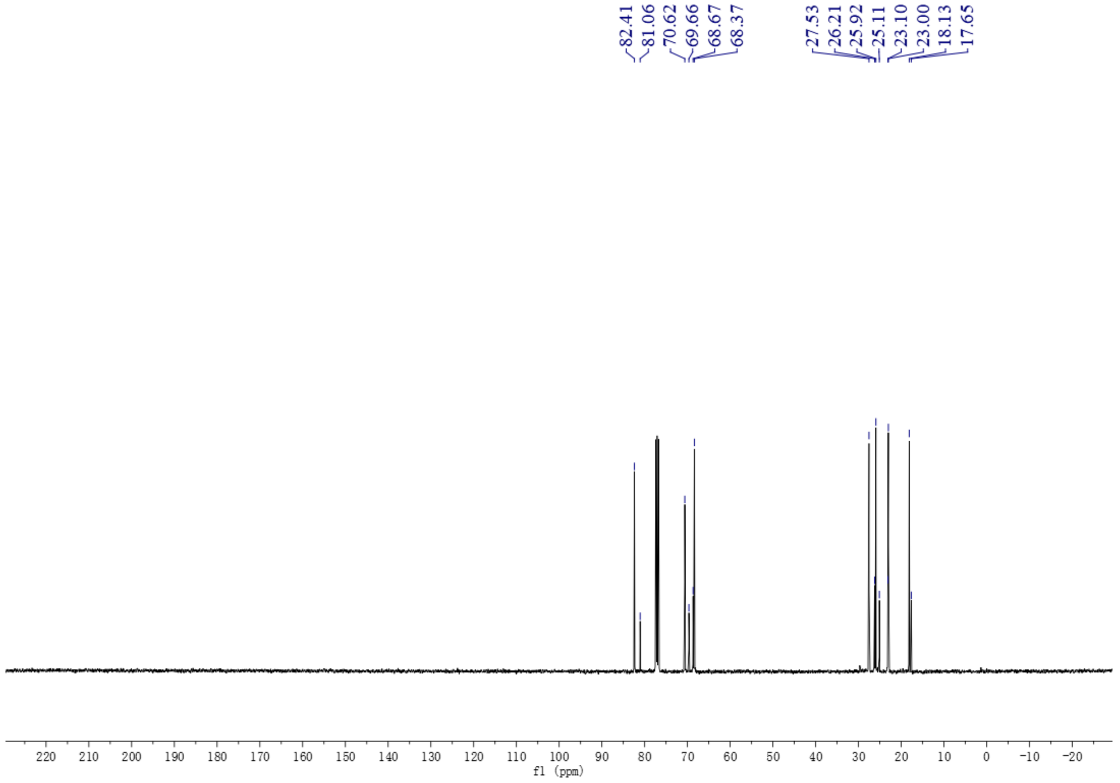

Supplementary Figure 115. ^{13}C NMR (101 MHz, $CDCl_3$) spectrum of **6.**

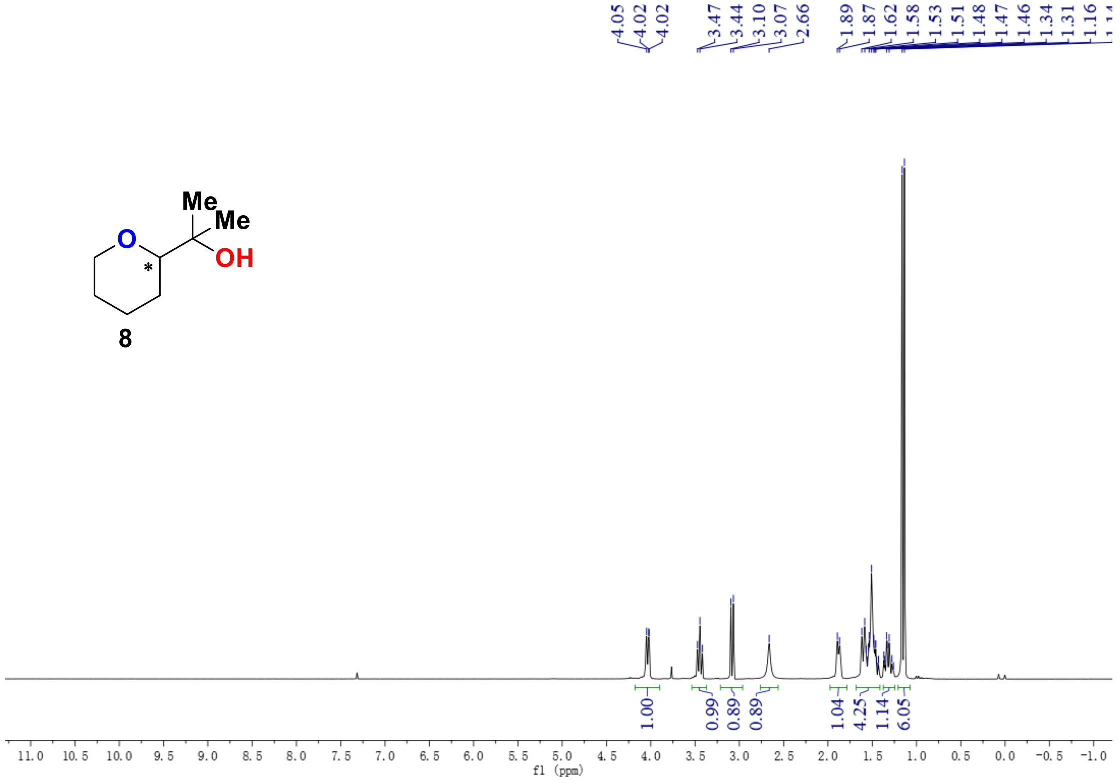

Supplementary Figure 116. ^{1}H NMR (400 MHz, $CDCl_3$) spectrum of **8.**

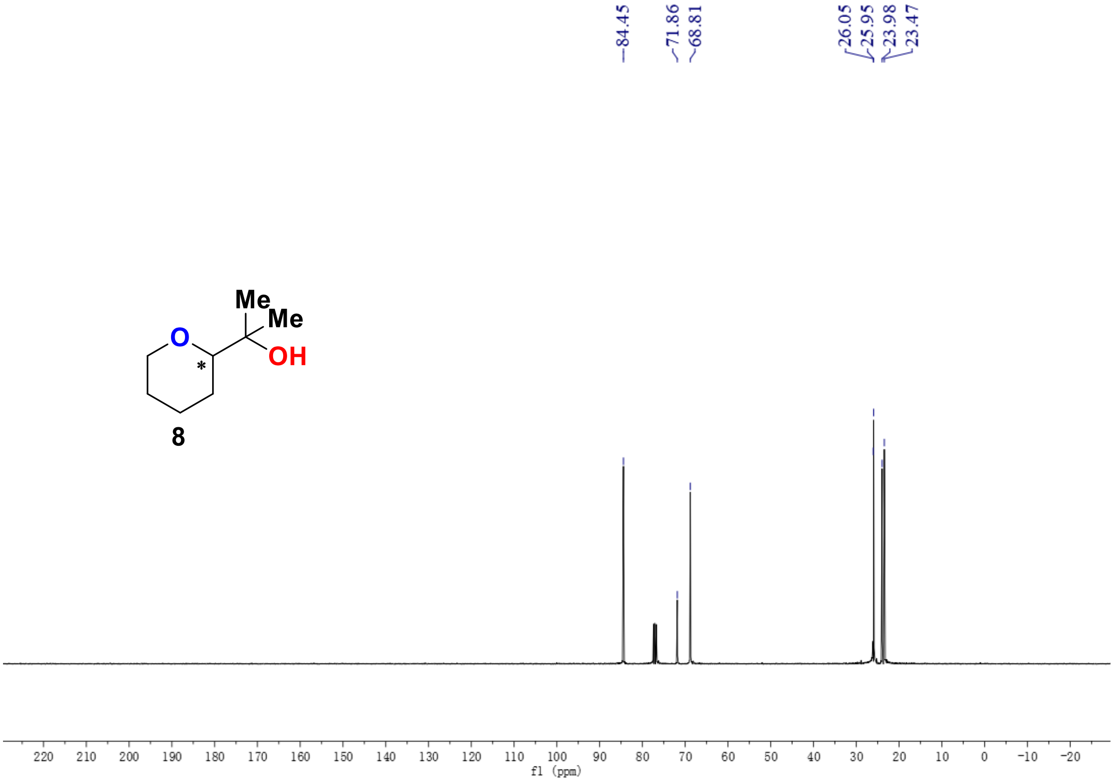

Supplementary Figure 117. **^{13}C NMR (101 MHz, CDCl$_3$)** spectrum of **8.**

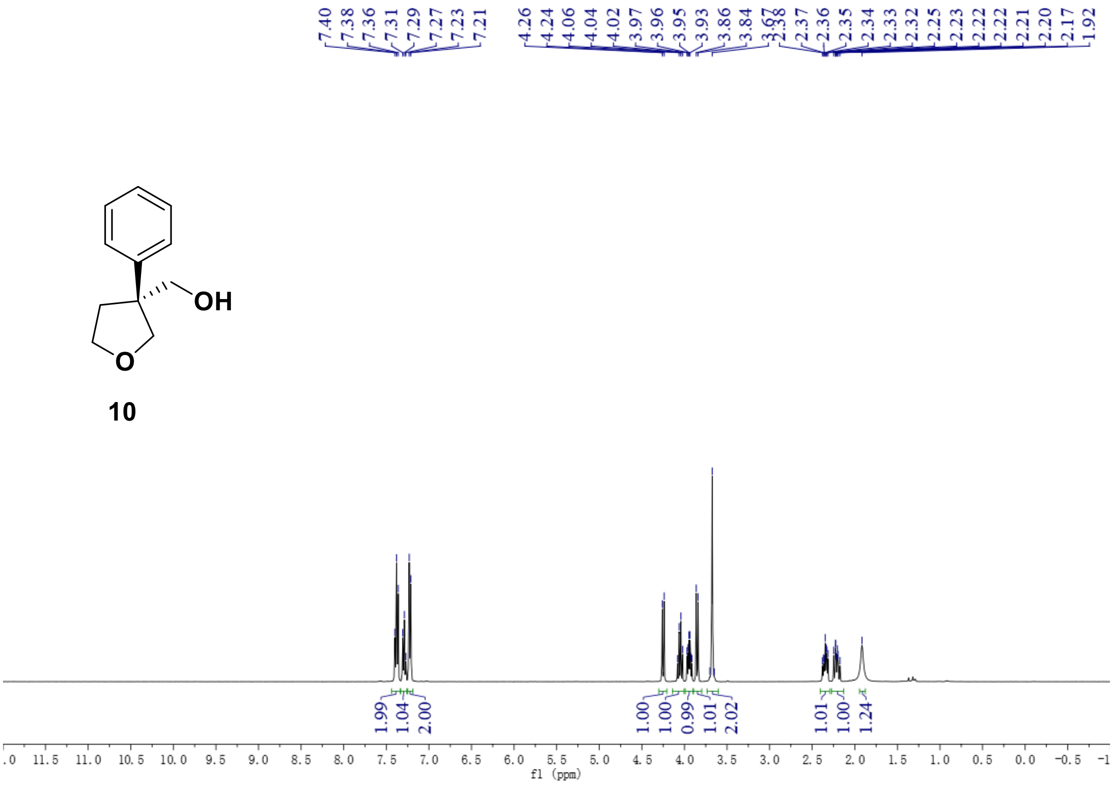

Supplementary Figure 118. **^{1}H NMR (400 MHz, CDCl$_3$)** spectrum of **10.**

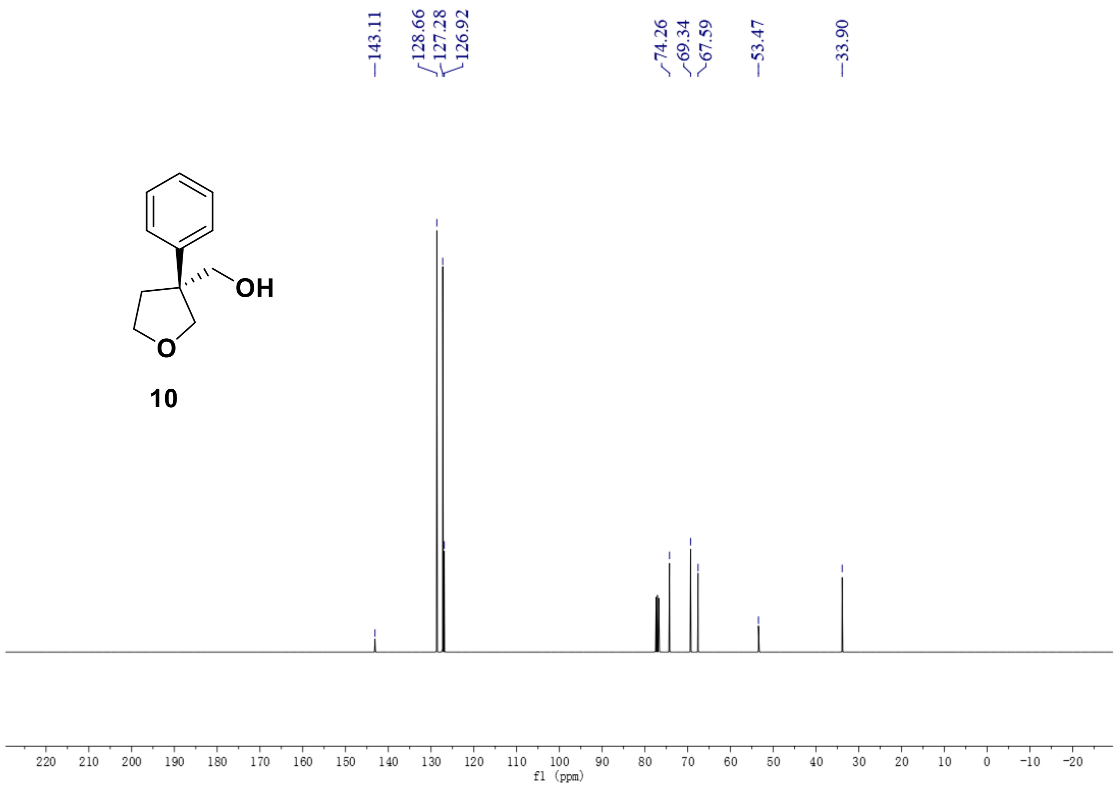

Supplementary Figure 119. **^{13}C NMR (101 MHz, $CDCl_3$)** spectrum of **10.**

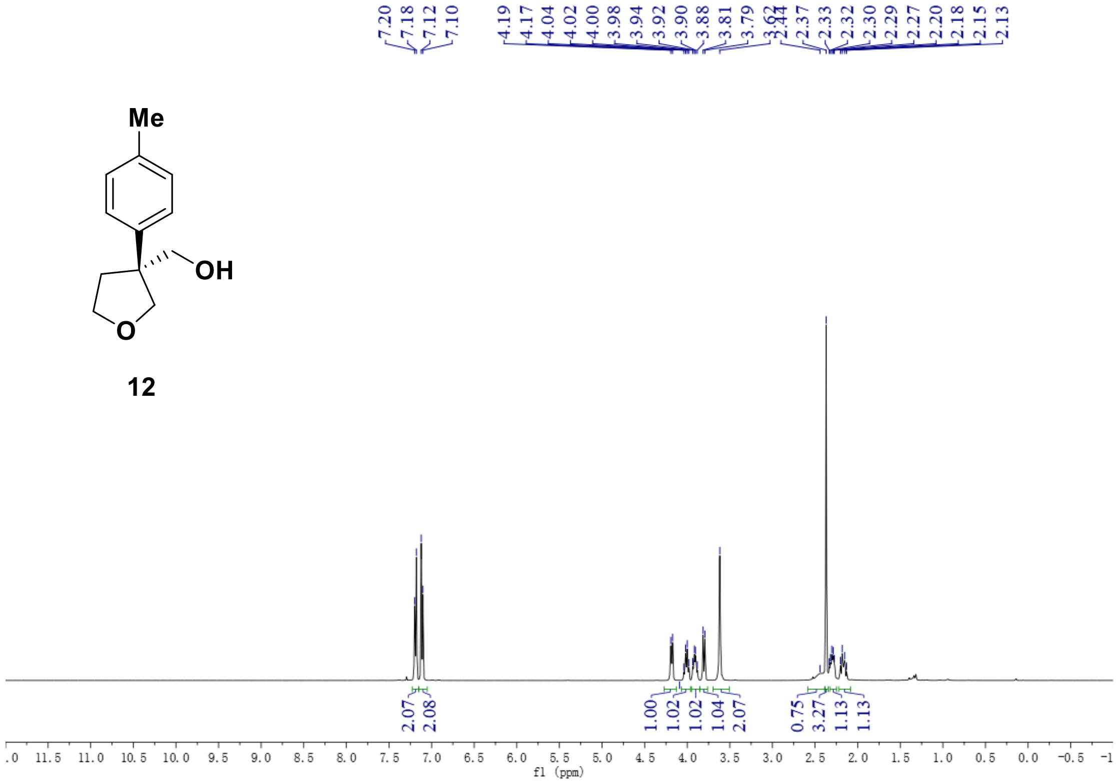

Supplementary Figure 120. **^{1}H NMR (400 MHz, $CDCl_3$)** spectrum of **12.**

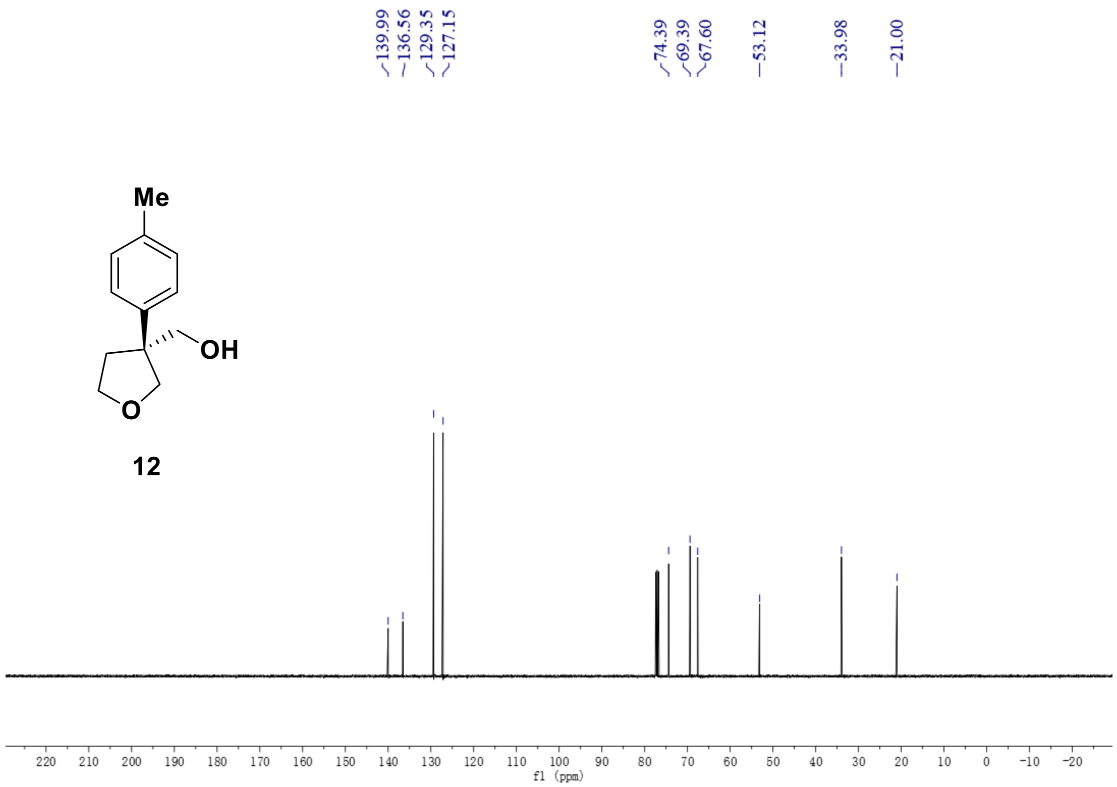

Supplementary Figure 121. **^{13}C NMR (101 MHz, $CDCl_3$)** spectrum of **12.**

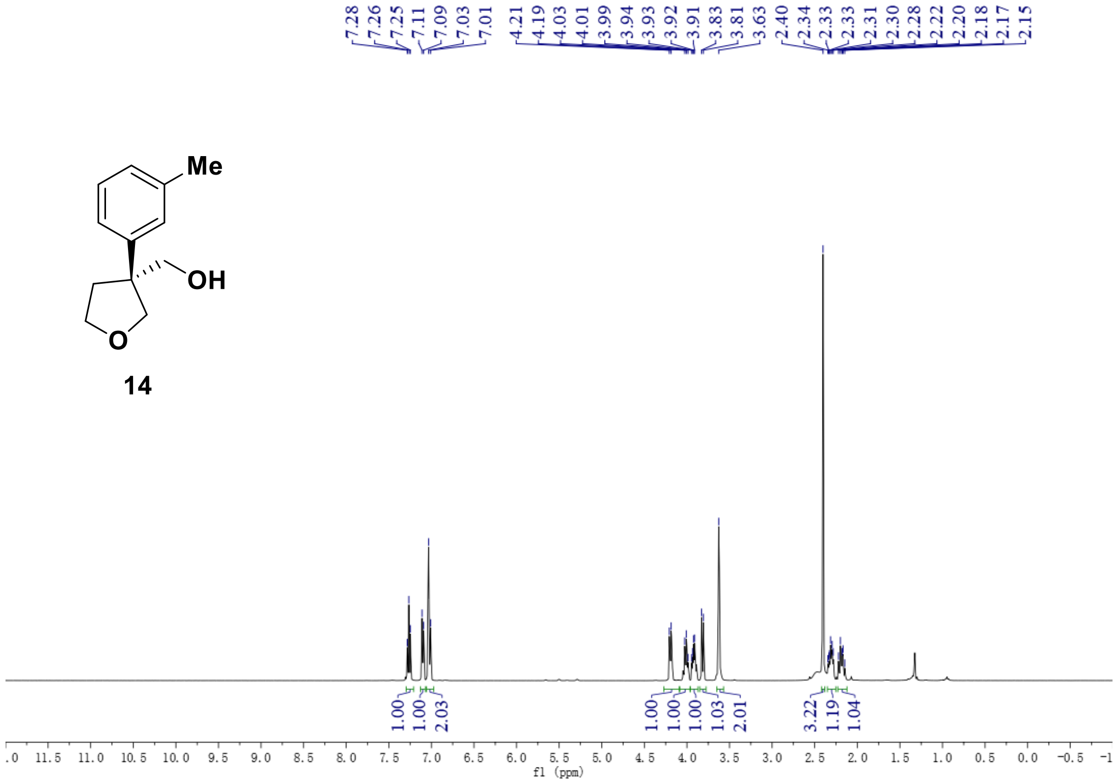

Supplementary Figure 122. **^{1}H NMR (400 MHz, $CDCl_3$)** spectrum of **14.**

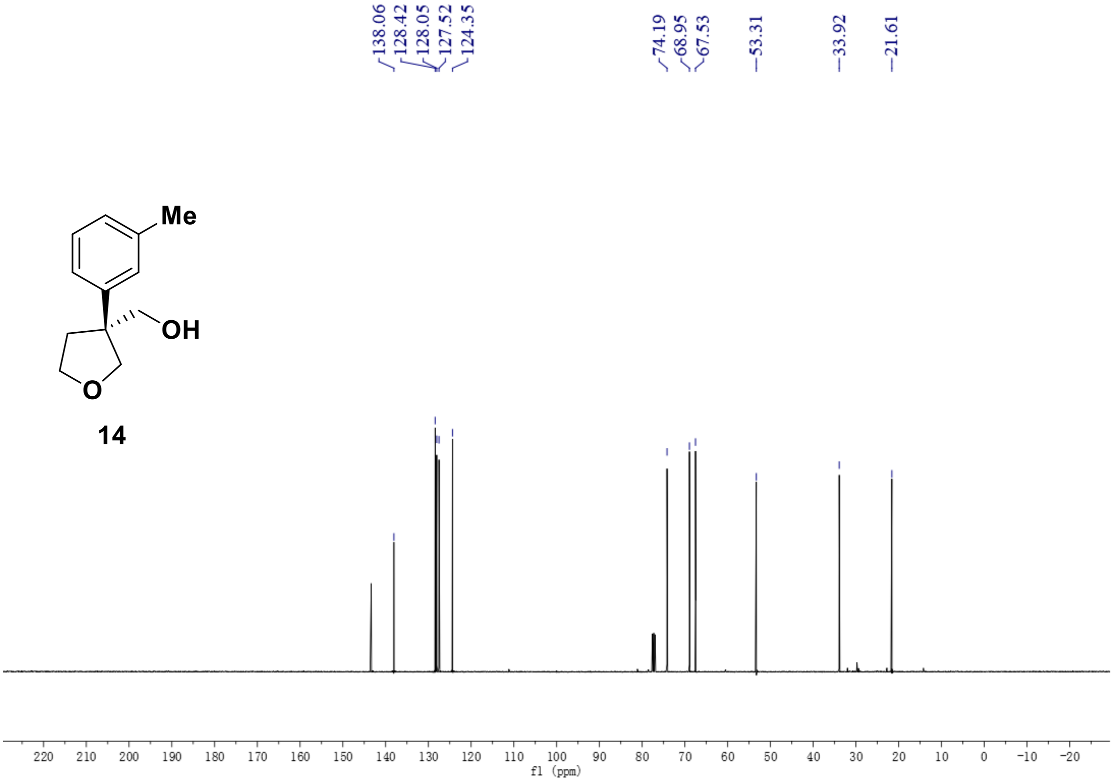

Supplementary Figure 123. ^{13}C NMR (101 MHz, CDCl$_3$) spectrum of **14.**

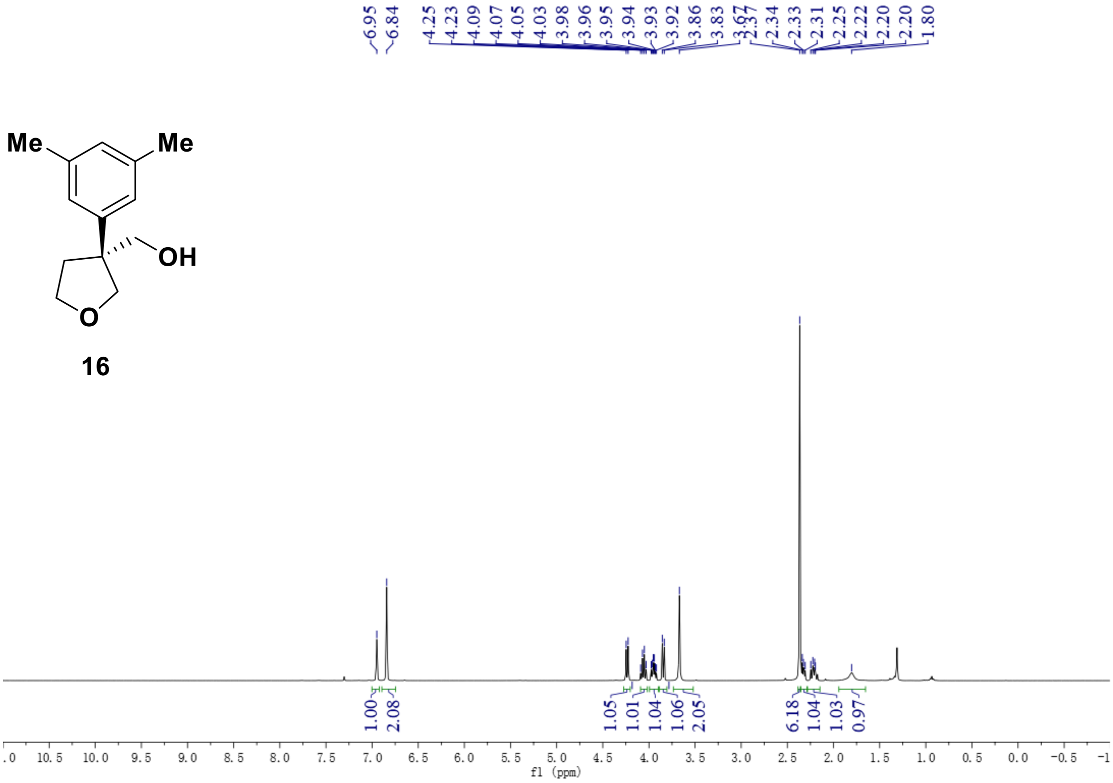

Supplementary Figure 124. ^{13}C NMR (101 MHz, CDCl$_3$) spectrum of **16.**

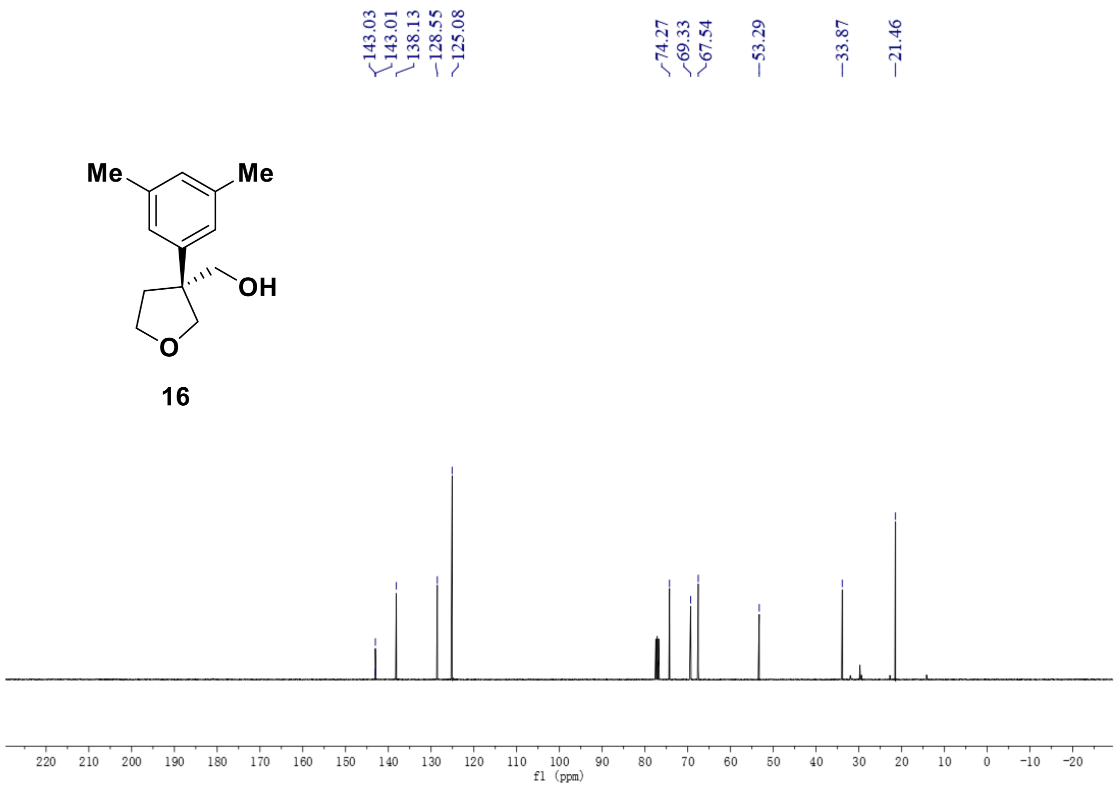

Supplementary Figure 125. ^{13}**C NMR (101 MHz, CDCl$_3$)** spectrum of **16.**

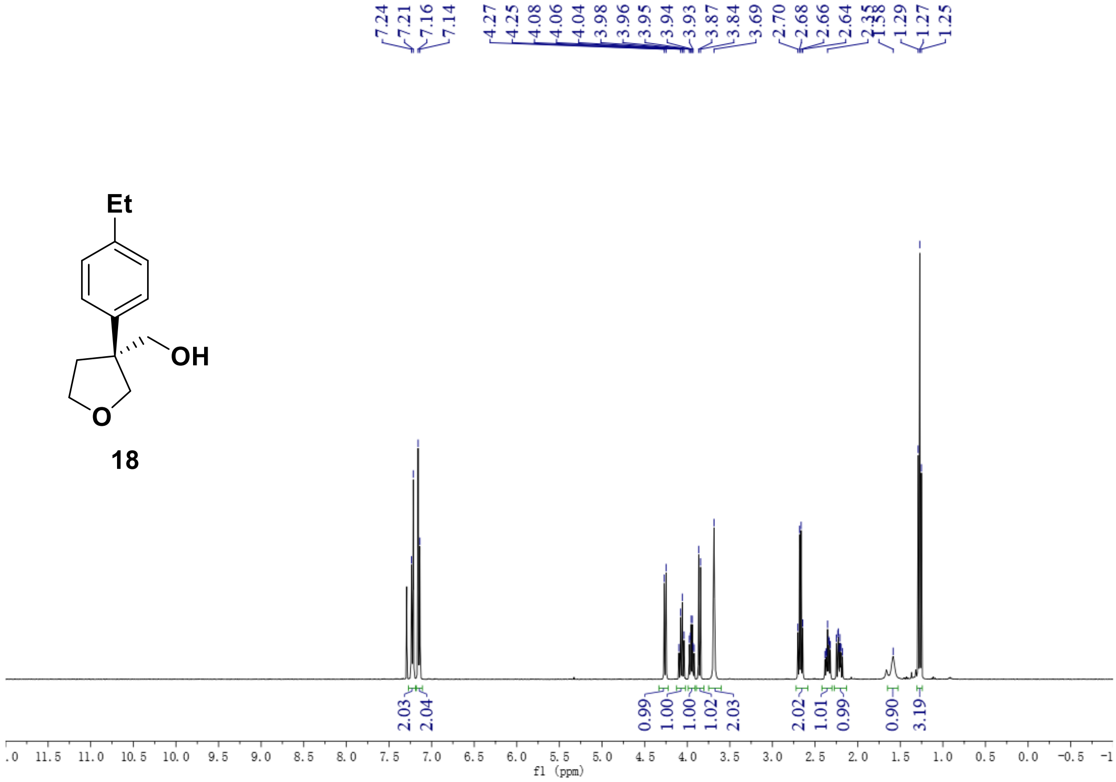

Supplementary Figure 126. ^{1}**H NMR (400 MHz, CDCl$_3$)** spectrum of **18.**

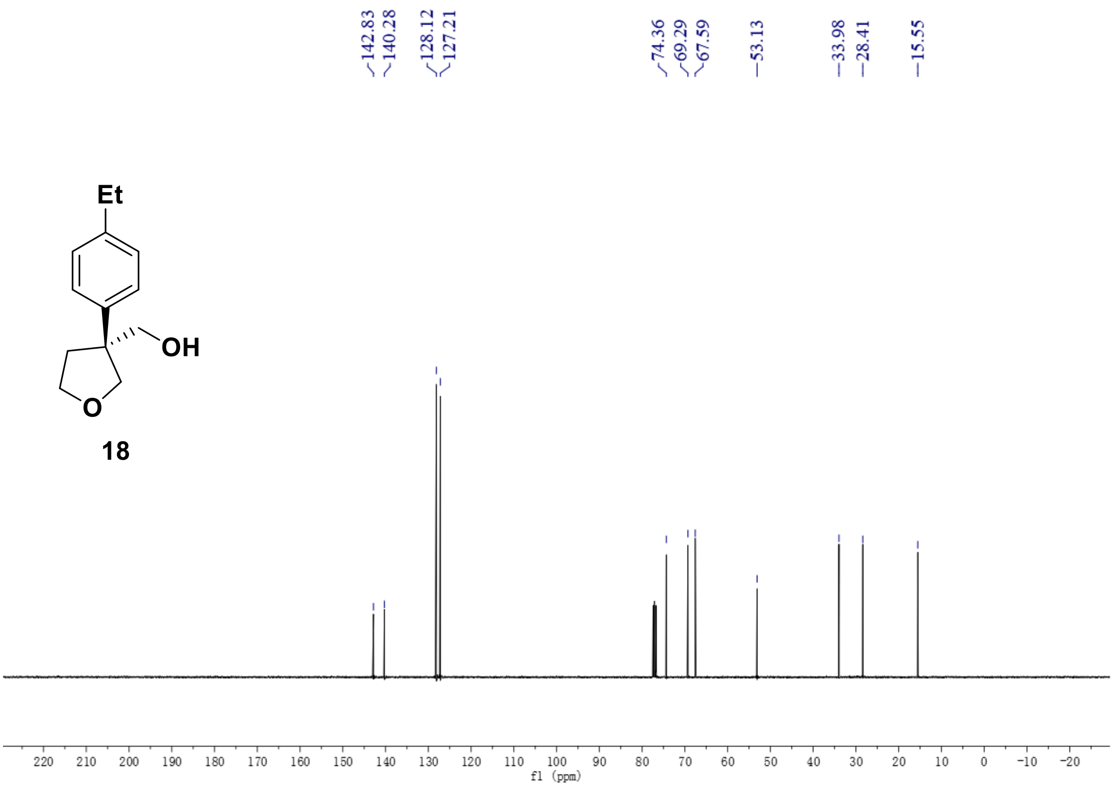

Supplementary Figure 127. **^{13}C NMR (101 MHz, $CDCl_3$)** spectrum of **18.**

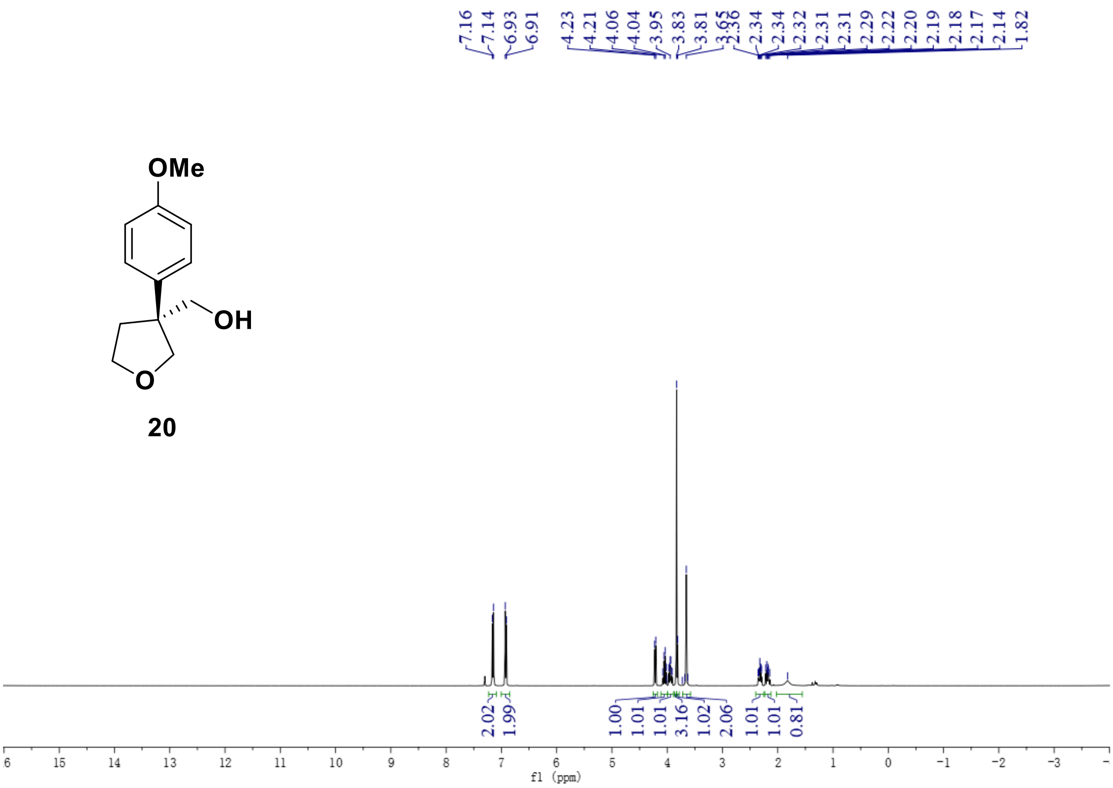

Supplementary Figure 128. **^{1}H NMR (400 MHz, $CDCl_3$)** spectrum of **20.**

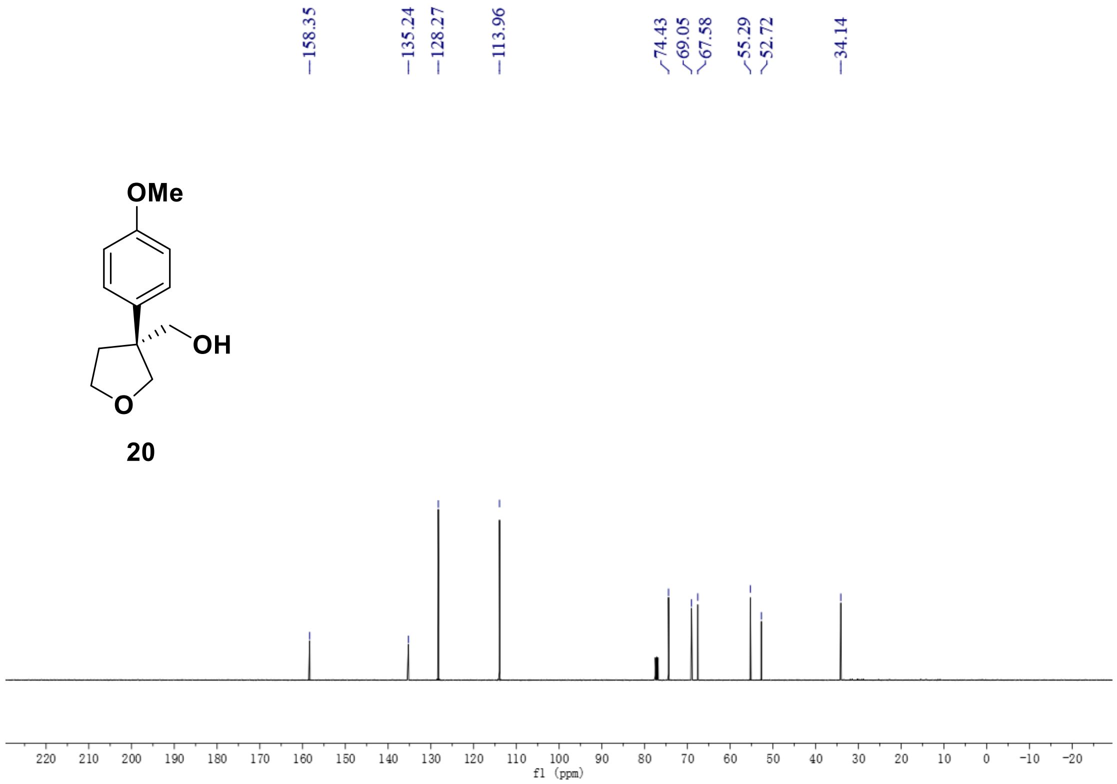

Supplementary Figure 129. ^{13}**C NMR (101 MHz, CDCl$_3$)** spectrum of **20.**

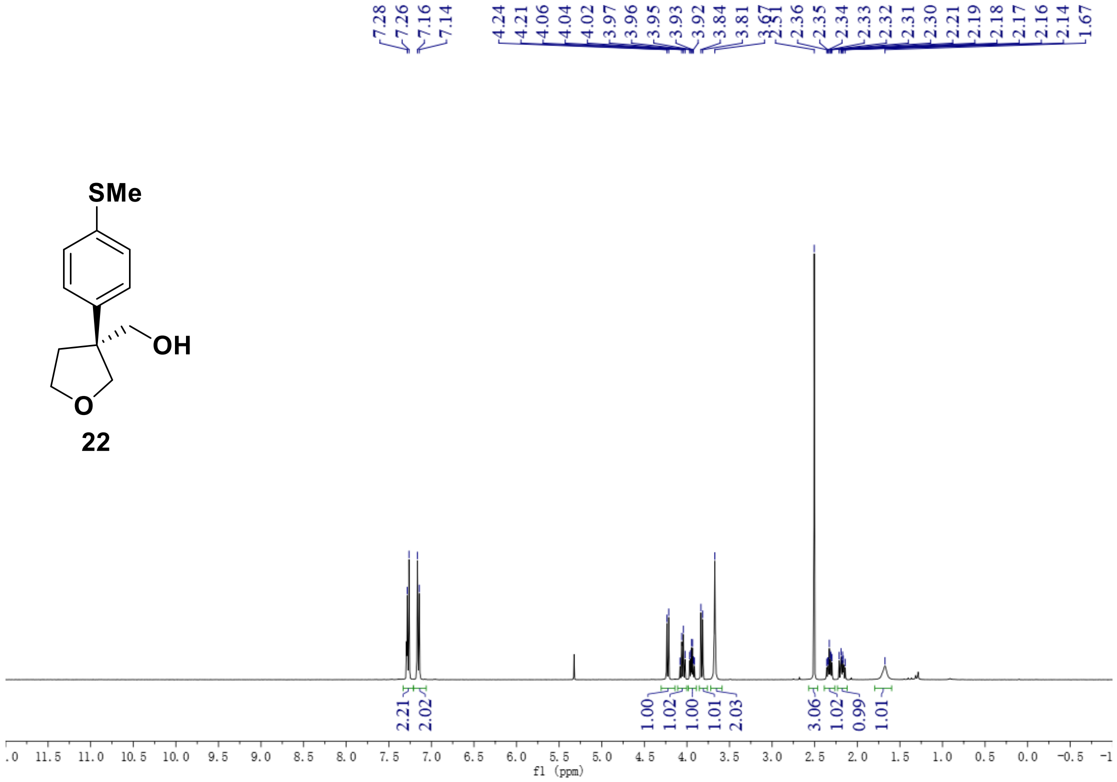

Supplementary Figure 130. ^{1}**H NMR (400 MHz, CDCl$_3$)** spectrum of **22.**

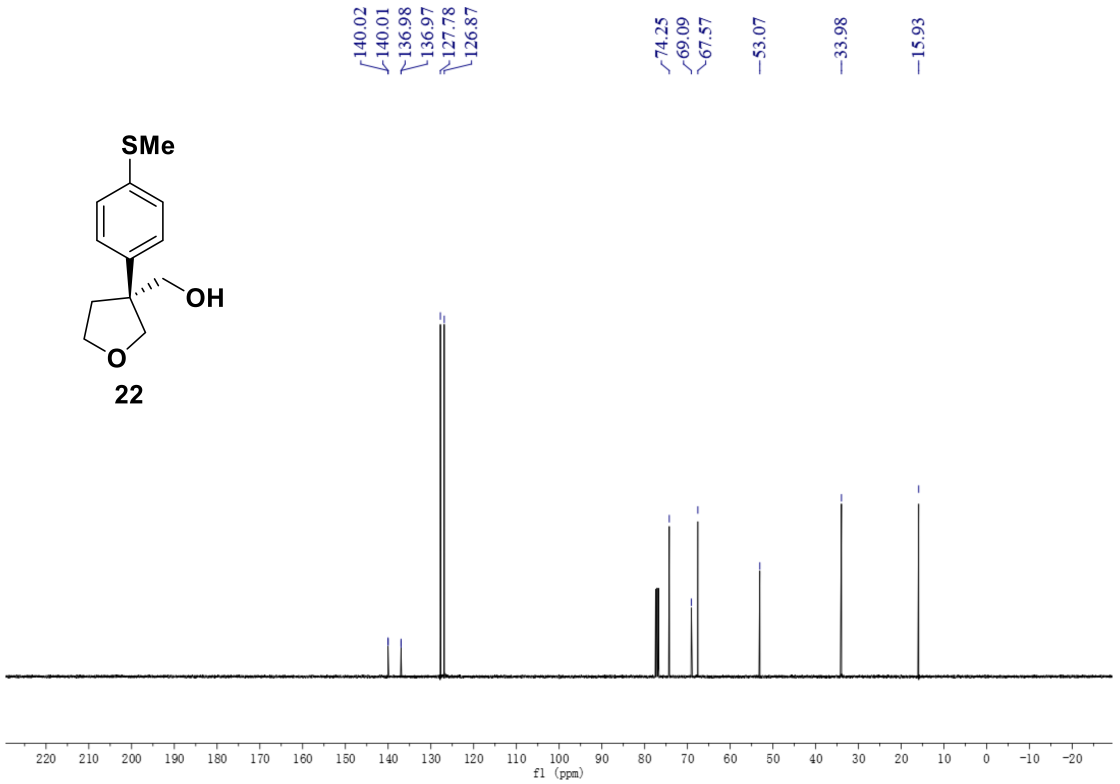

Supplementary Figure 131. ^{13}**C NMR (101 MHz, CDCl$_3$)** spectrum of **22.**

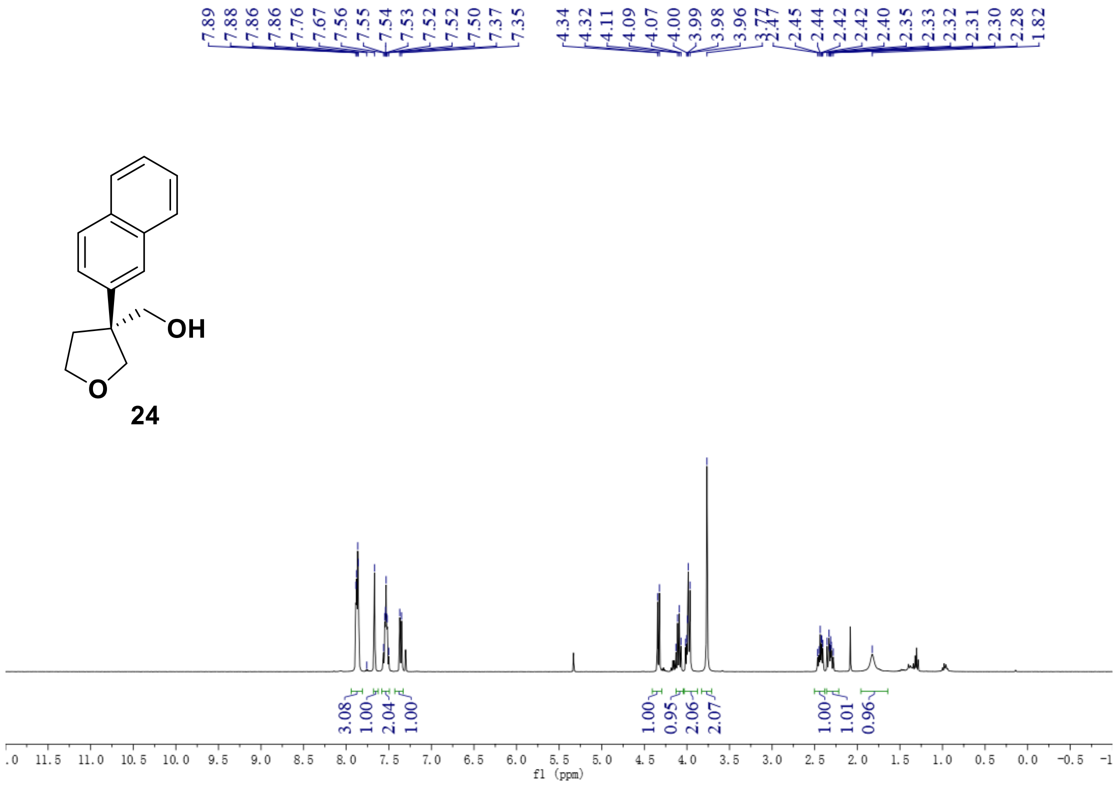

Supplementary Figure 132. ^{1}**H NMR (400 MHz, CDCl$_3$)** spectrum of **24.**

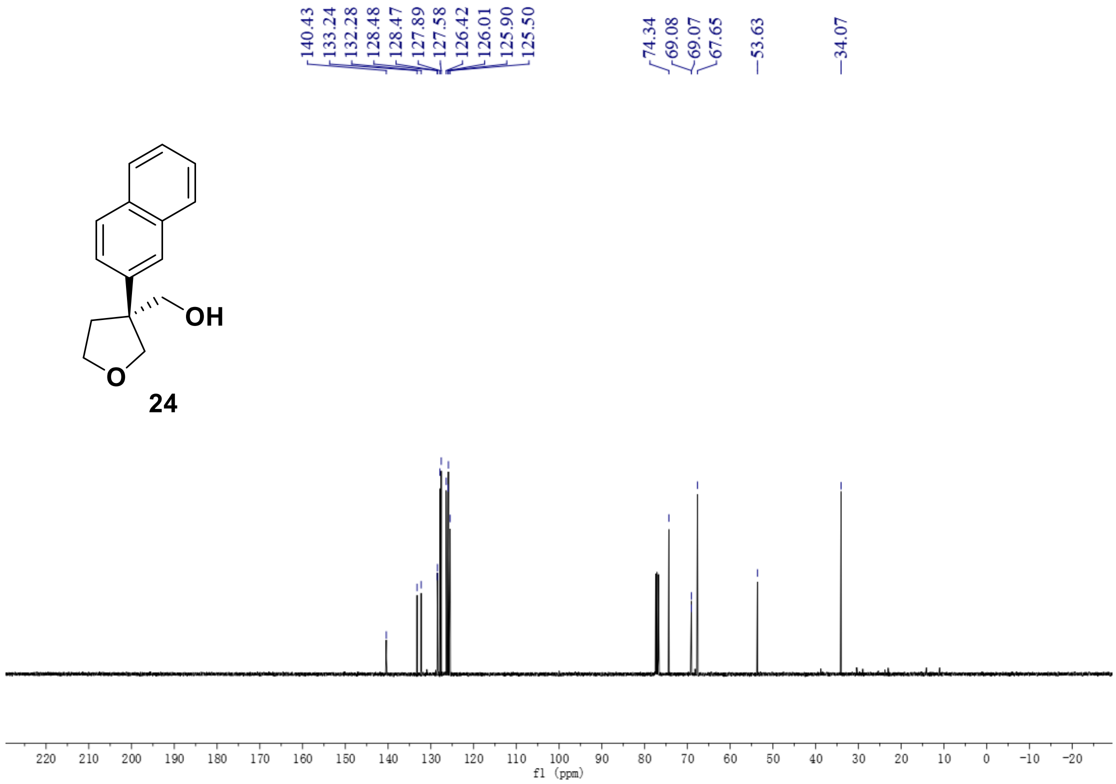

Supplementary Figure 133. ^{13}**C NMR (101 MHz,** $CDCl_3$**)** spectrum of **24.**

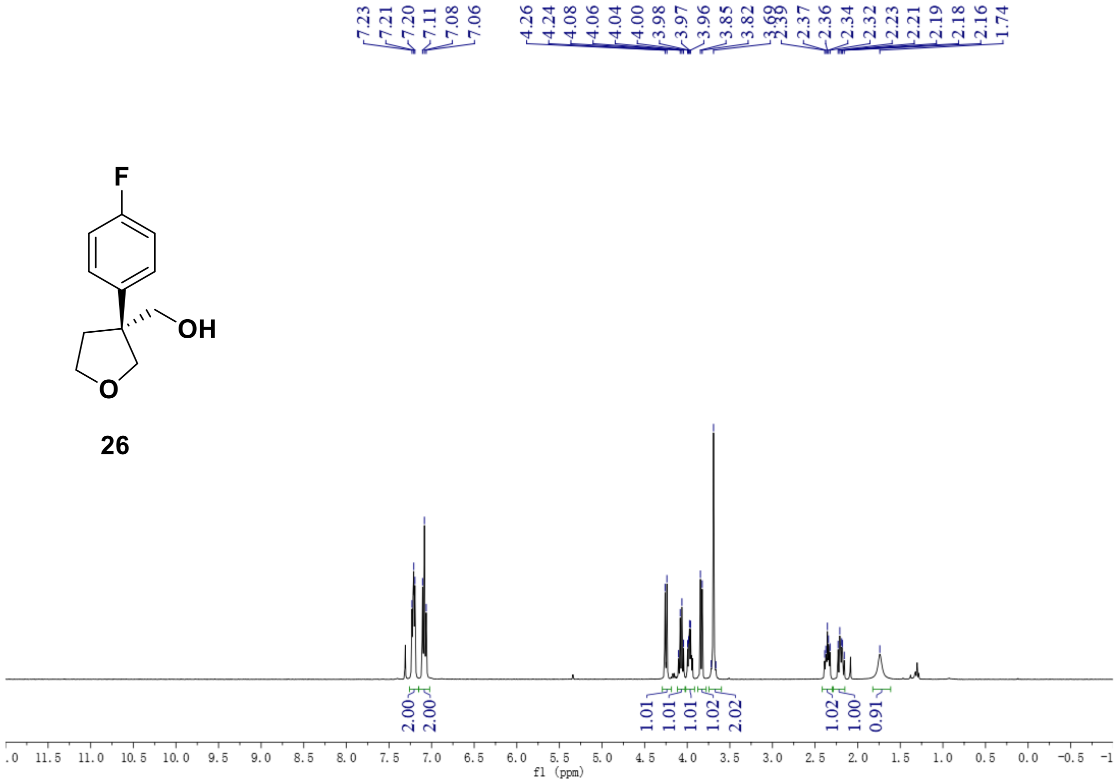

Supplementary Figure 134. ^{1}**H NMR (400 MHz,** $CDCl_3$**)** spectrum of **26.**

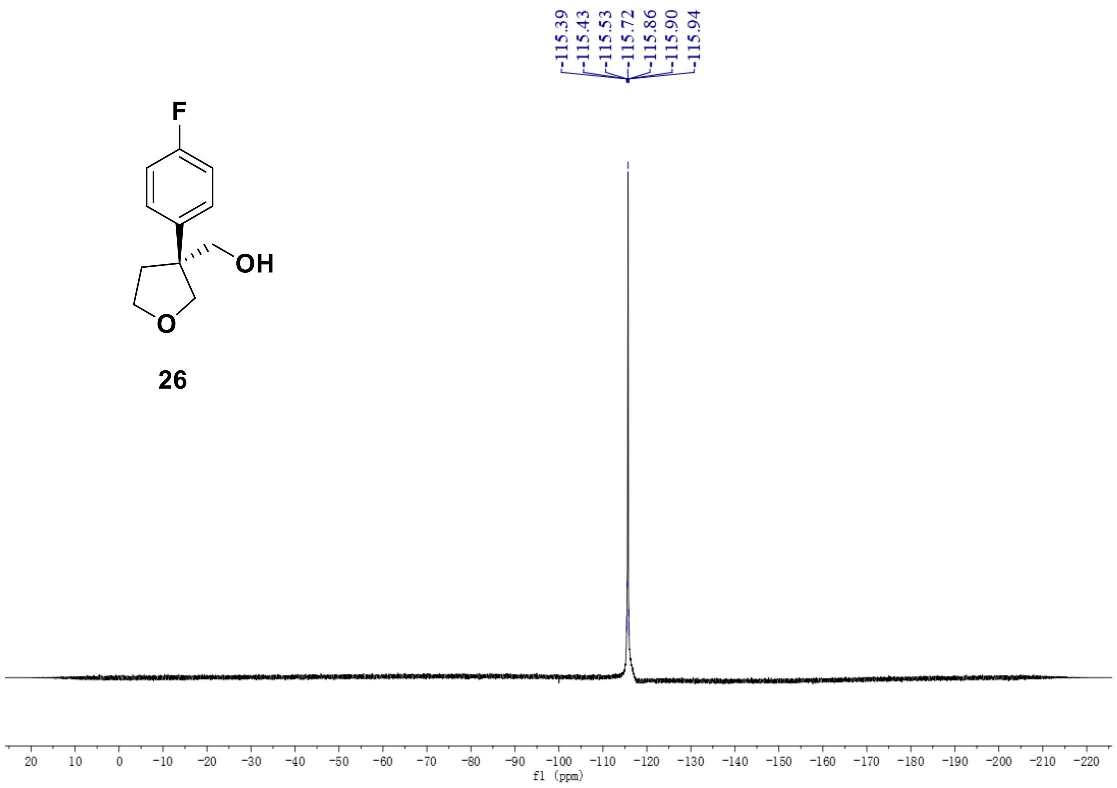

Supplementary Figure 135. ^{19}**F NMR (376 MHz, CDCl$_3$)** spectrum of **26.**

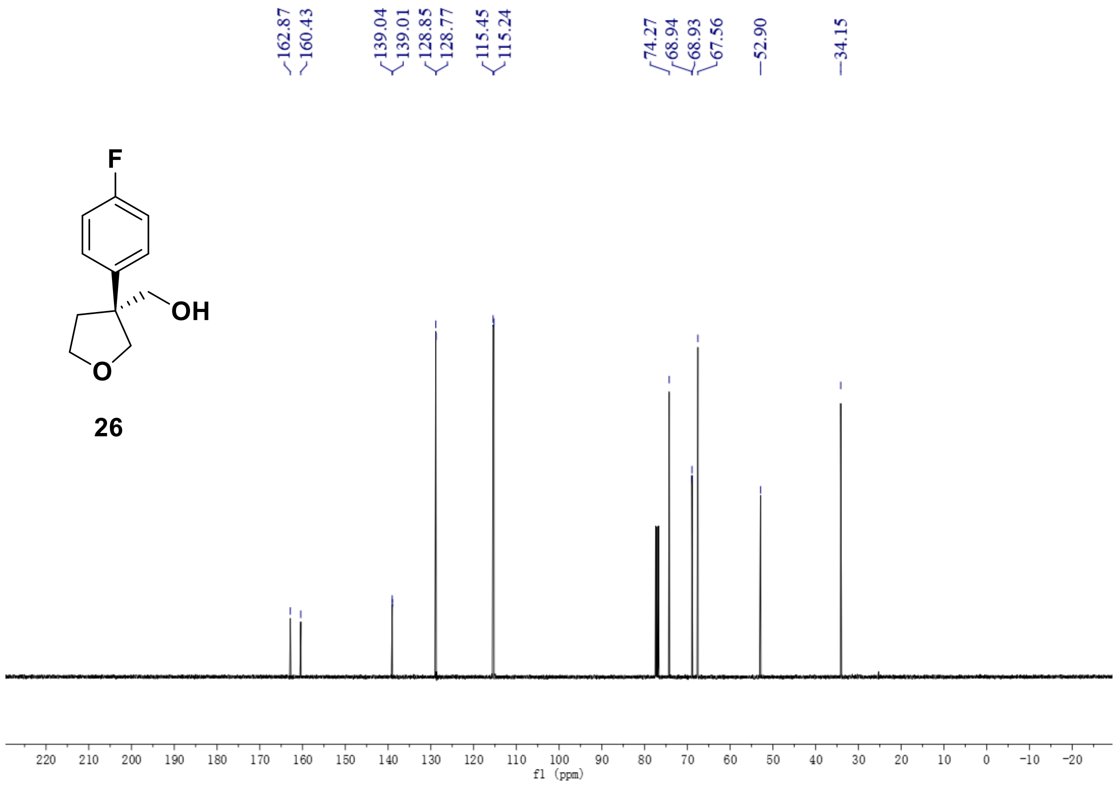

Supplementary Figure 136. ^{13}**C NMR (101 MHz, CDCl$_3$)** spectrum of **26.**

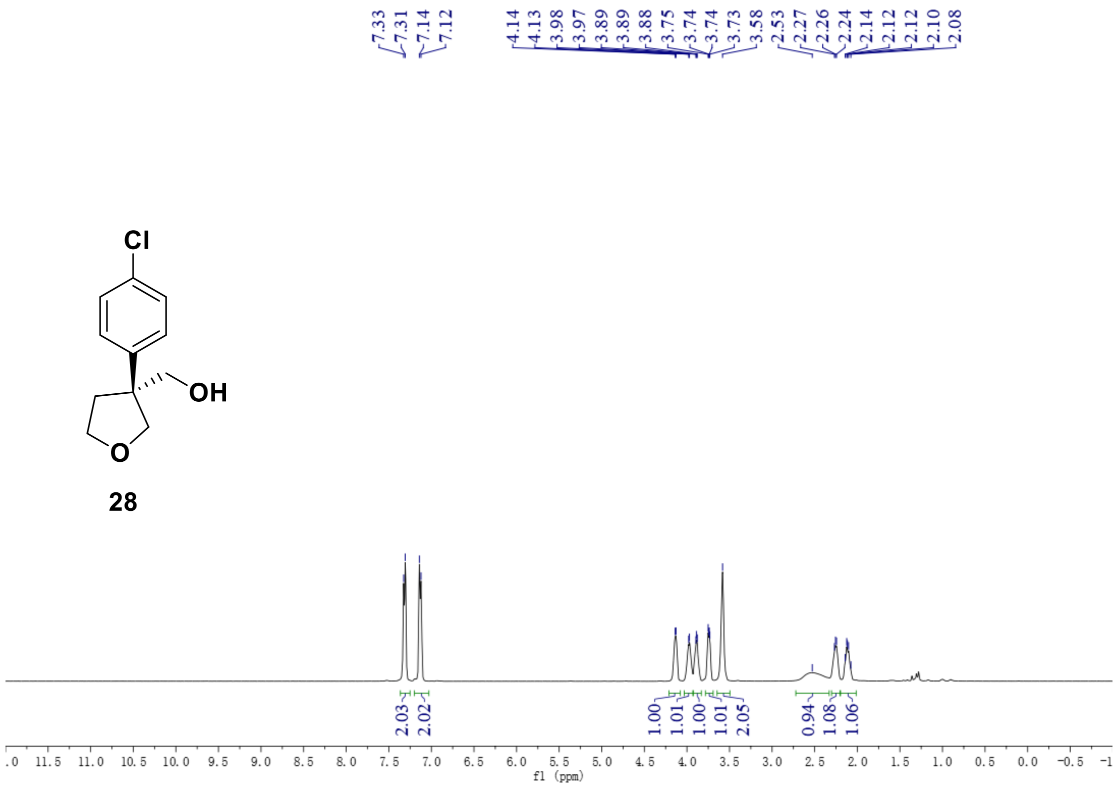

Supplementary Figure 137. ^{1}H NMR (400 MHz, $CDCl_3$) spectrum of **28.**

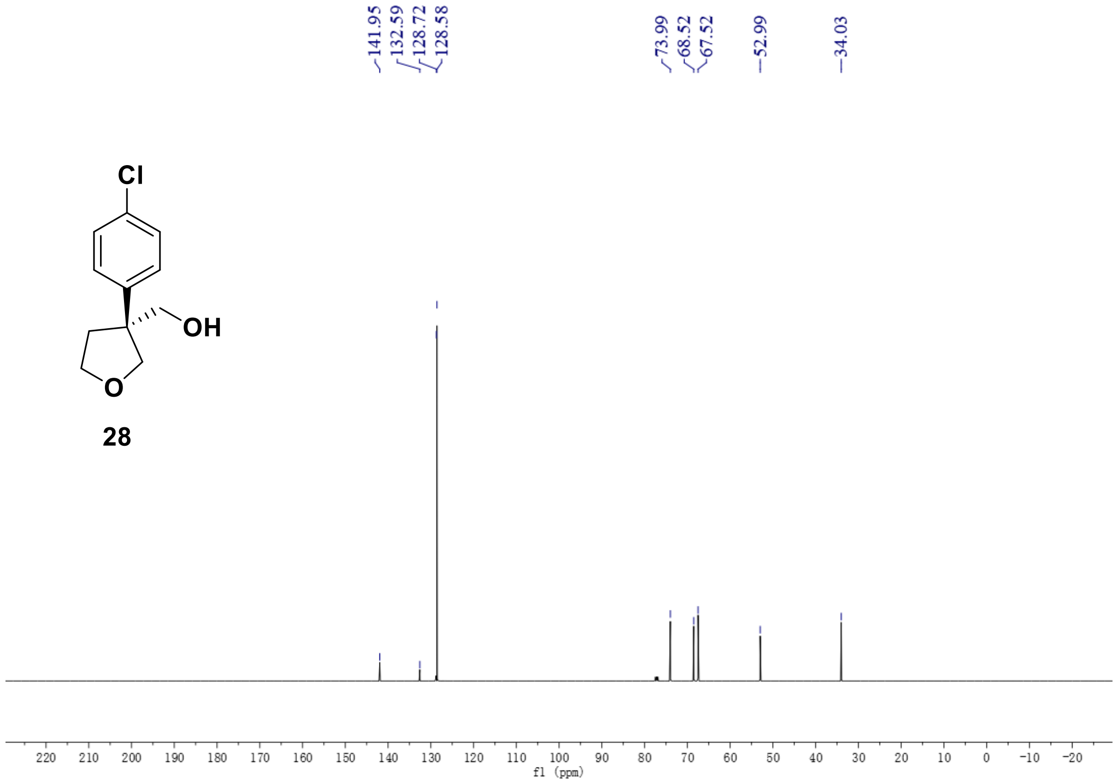

Supplementary Figure 138. ^{13}C NMR (101 MHz, $CDCl_3$) spectrum of **28.**

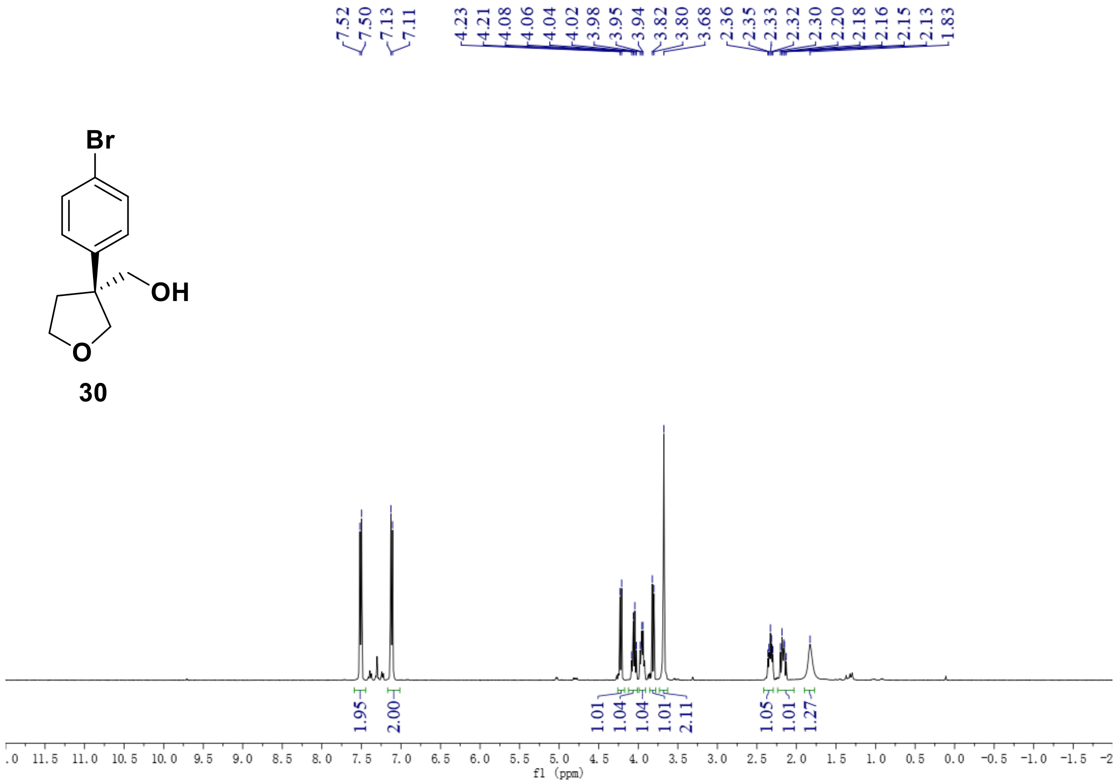

Supplementary Figure 139. ^{1}H NMR (400 MHz, $CDCl_3$) spectrum of 30.

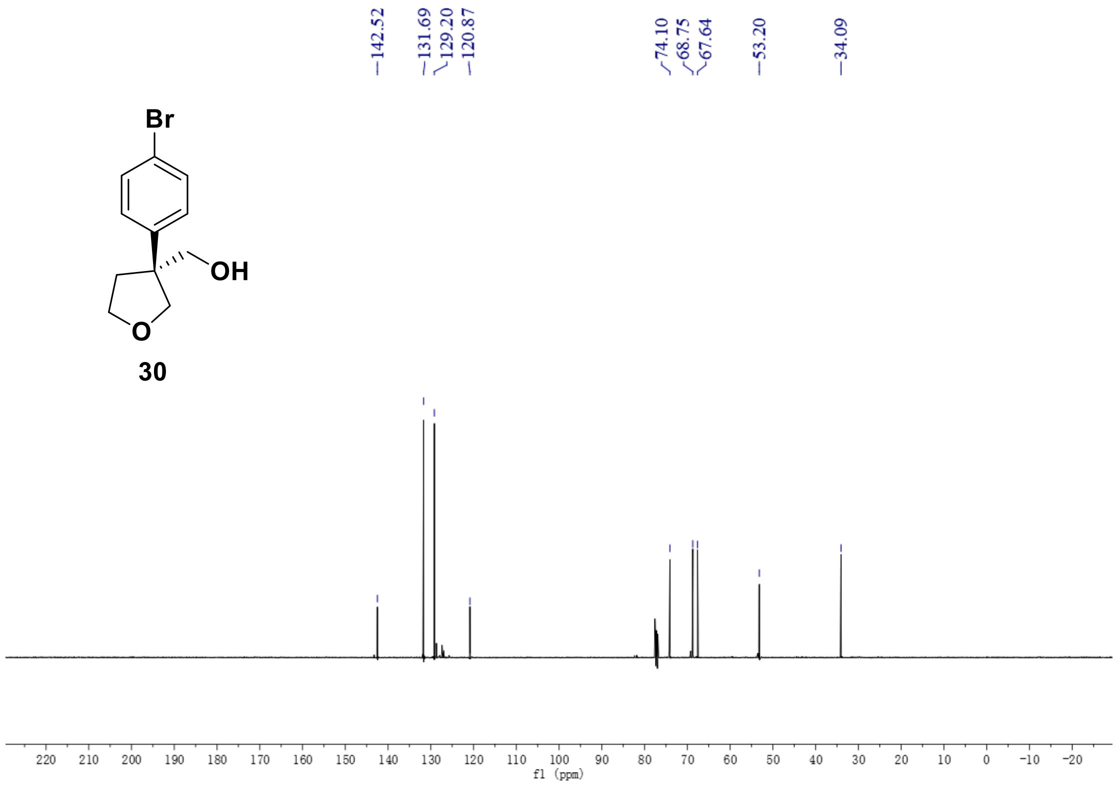

Supplementary Figure 140. ^{13}C NMR (101 MHz, $CDCl_3$) spectrum of 30.

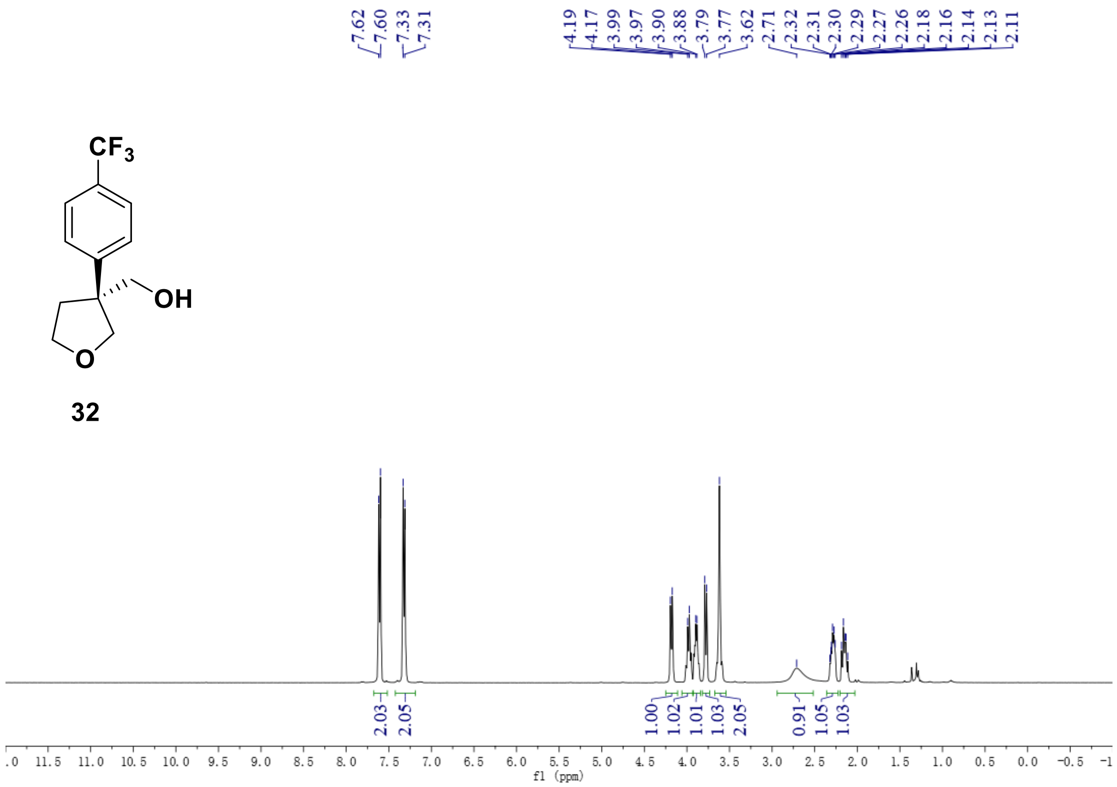

32

Supplementary Figure 141. ^{1}H NMR (400 MHz, $CDCl_3$) spectrum of **32.**

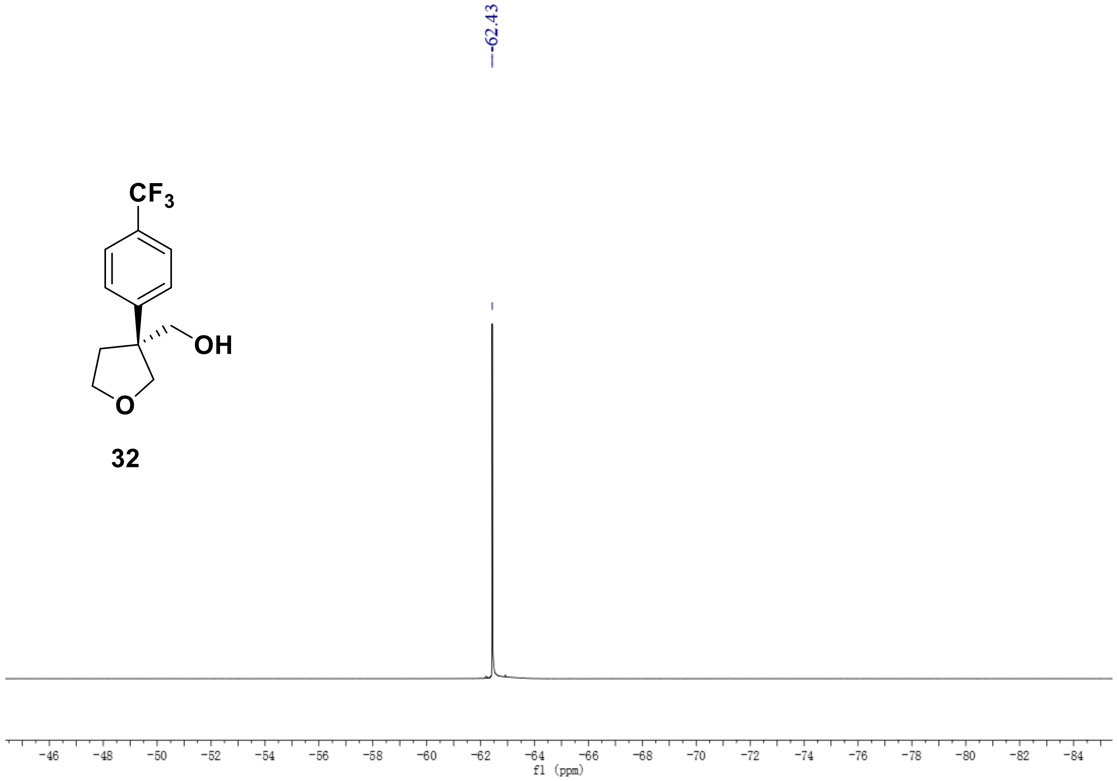

32

Supplementary Figure 142. ^{19}F NMR (376 MHz, $CDCl_3$) spectrum of **32.**

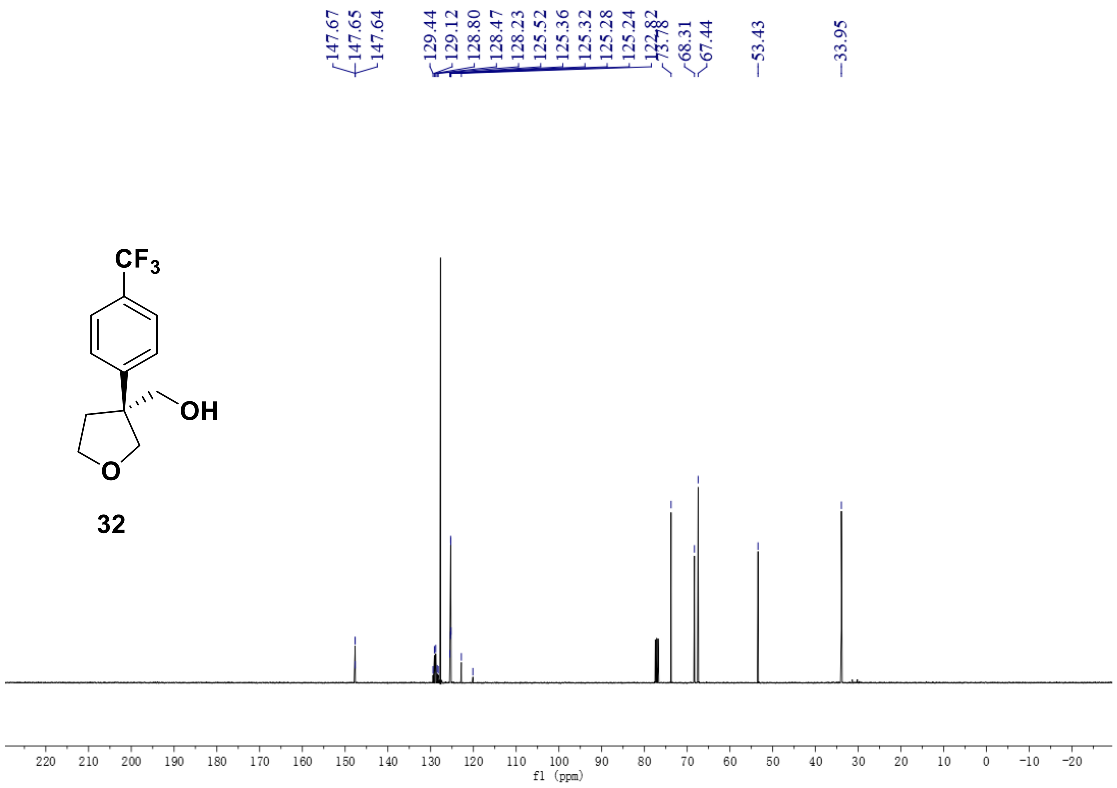

Supplementary Figure 143. ^{13}**C NMR (101 MHz, CDCl**$_3$**)** spectrum of **32.**

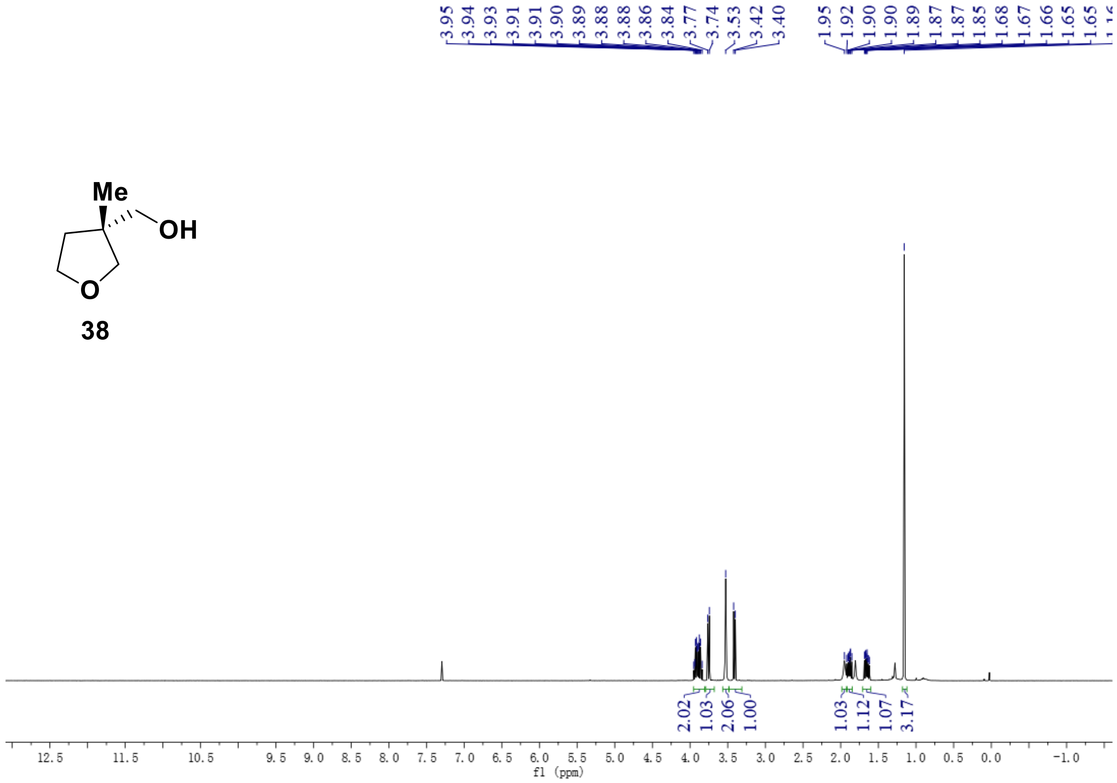

Supplementary Figure 144. ^{1}**H NMR (400 MHz, CDCl**$_3$**)** spectrum of **38.**

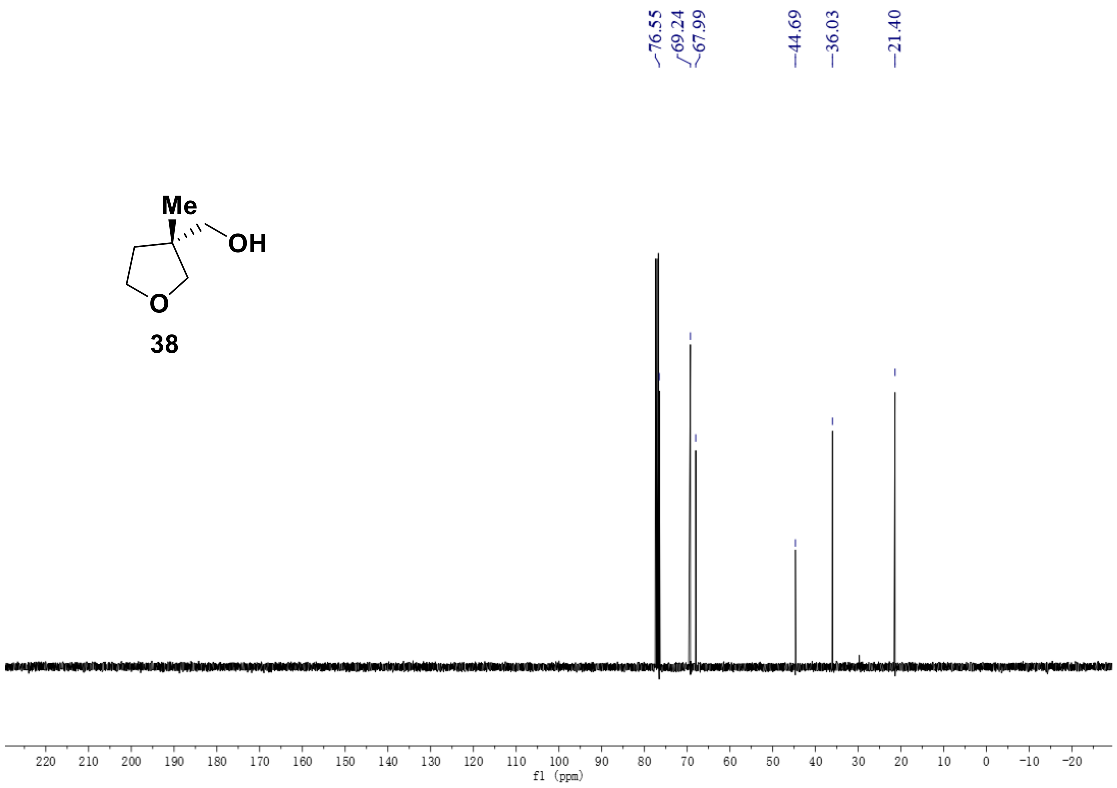

Supplementary Figure 145. **^{13}C NMR (101 MHz, CDCl$_3$)** spectrum of **38.**

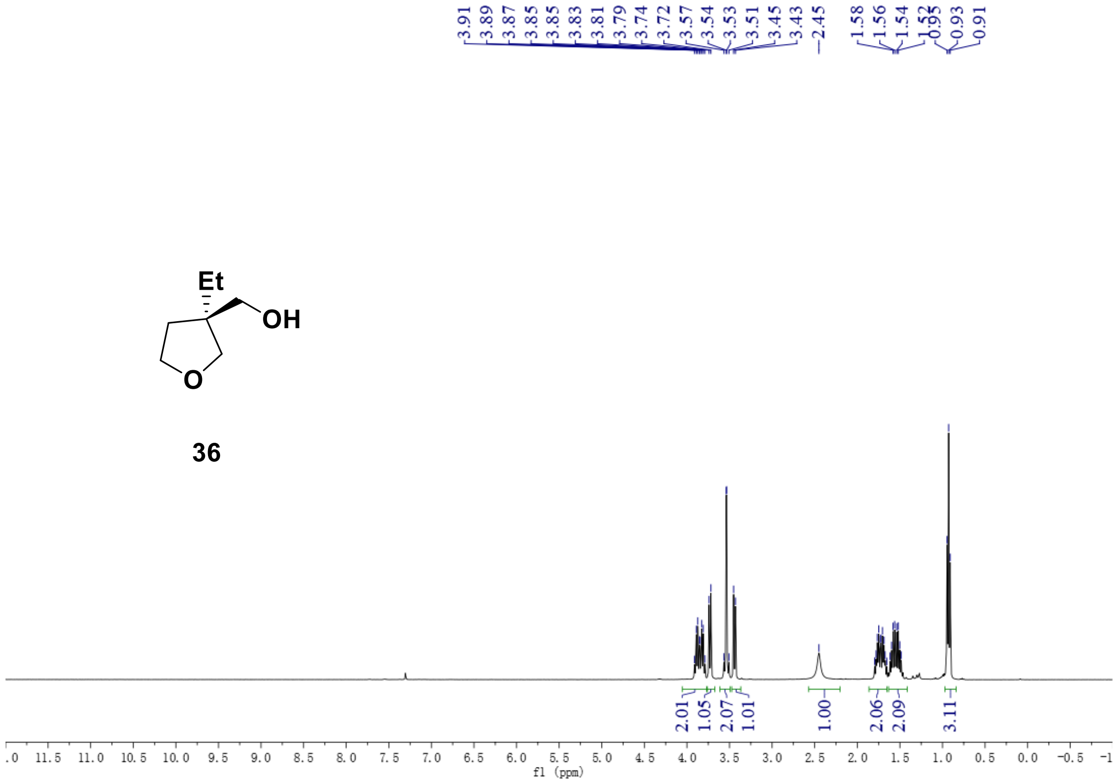

Supplementary Figure 146. **^{1}H NMR (400 MHz, CDCl$_3$)** spectrum of **36.**

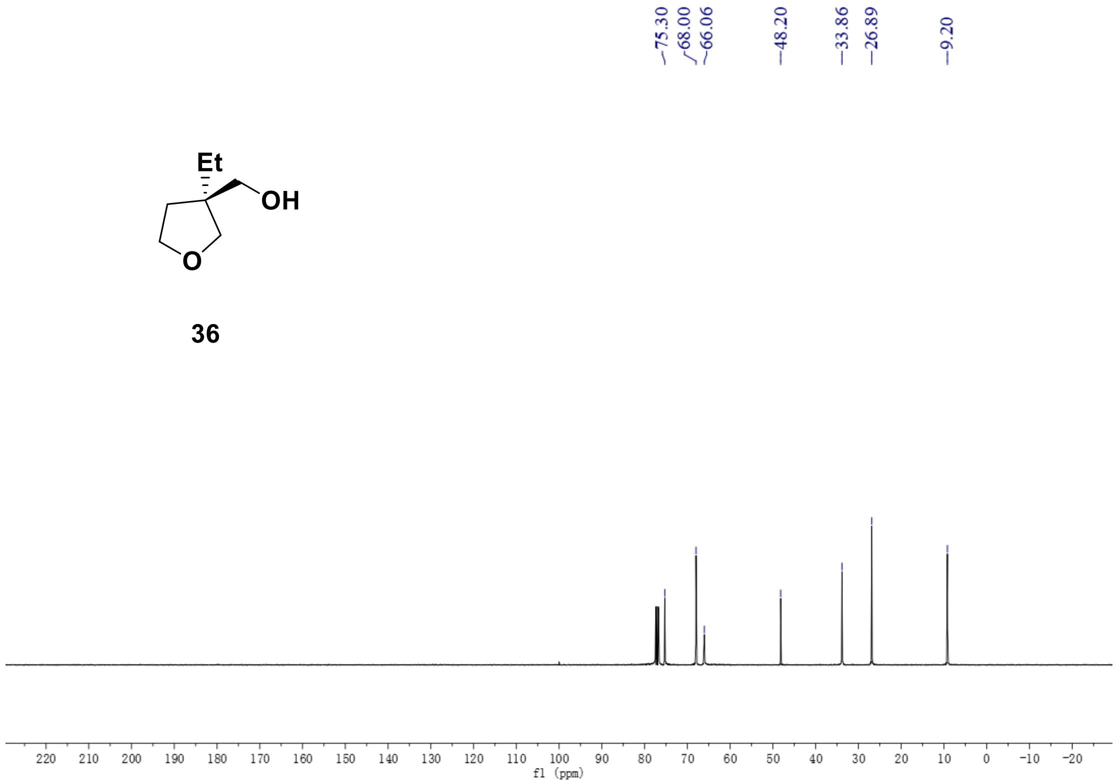

Supplementary Figure 147. **^{13}C NMR (101 MHz, $CDCl_3$)** spectrum of **36.**

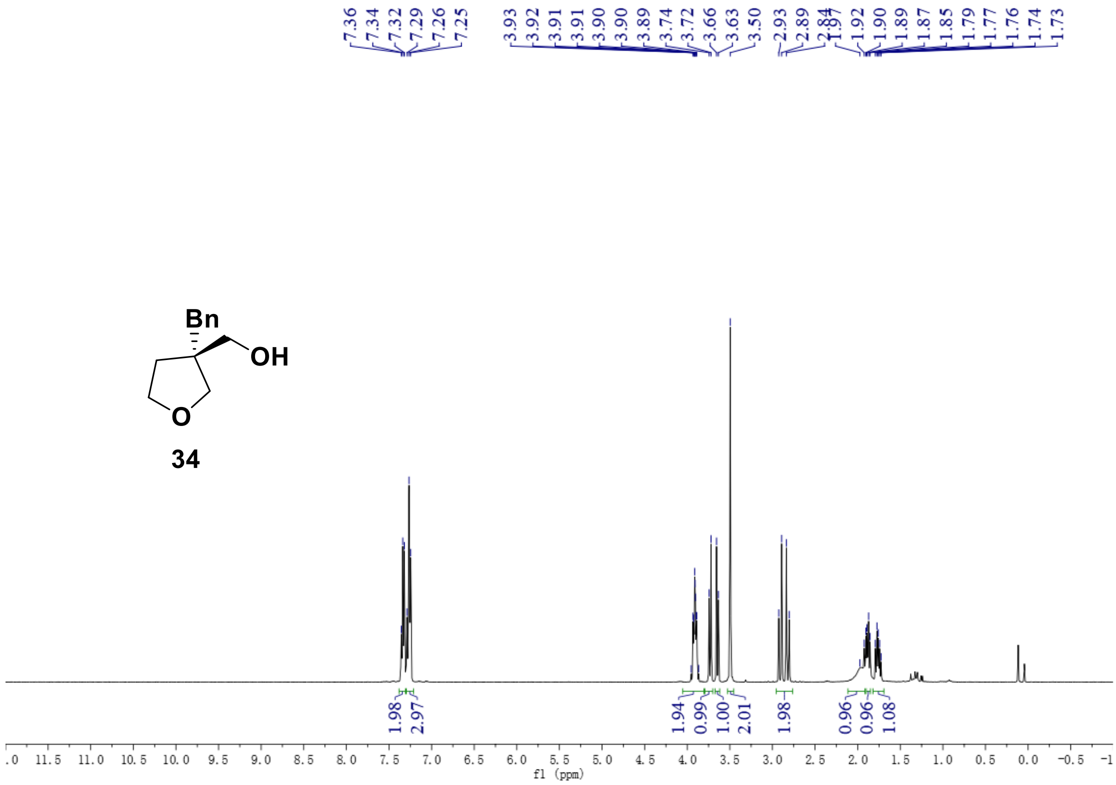

Supplementary Figure 148. **^{1}H NMR (400 MHz, $CDCl_3$)** spectrum of **34.**

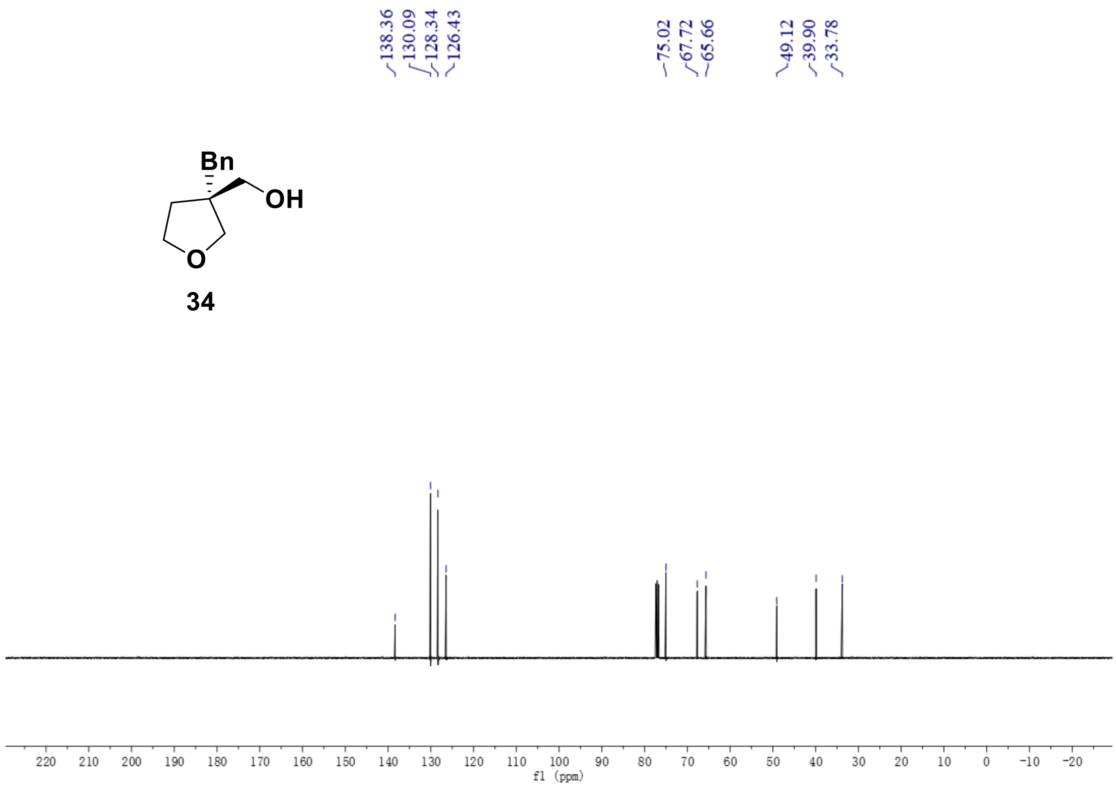

Supplementary Figure 149. ^{13}**C NMR (101 MHz,** $CDCl_3$**)** spectrum of **34.**

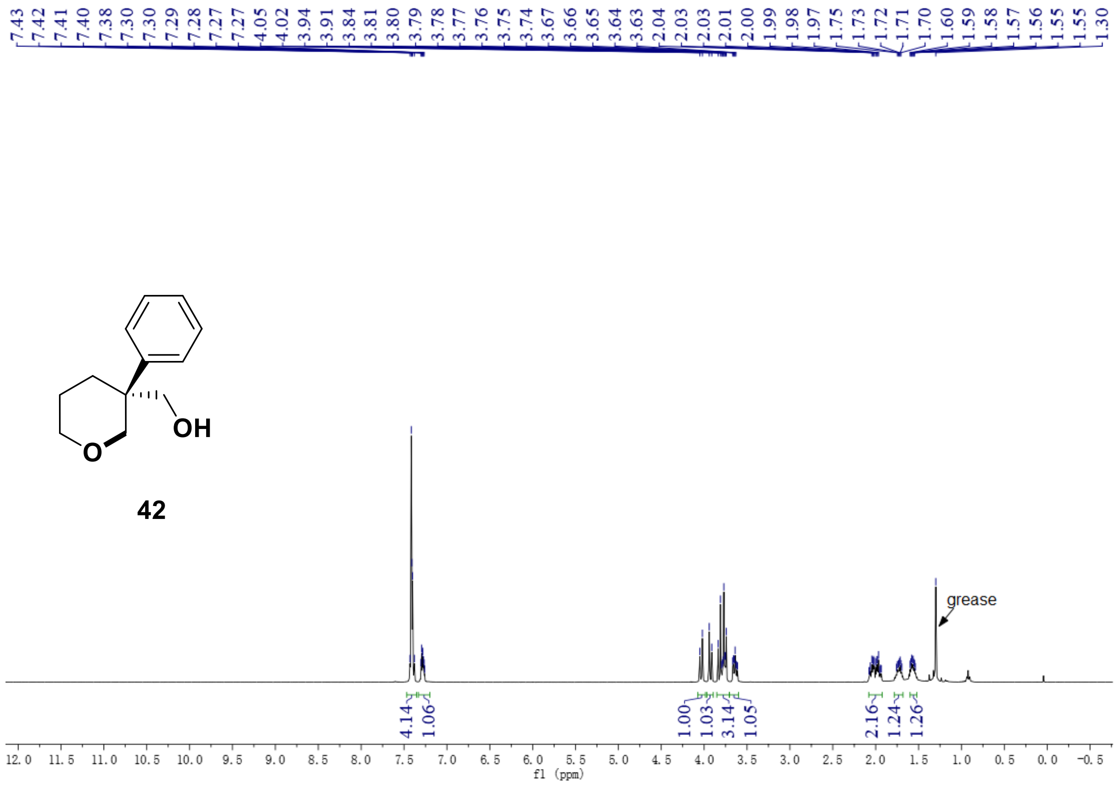

Supplementary Figure 150. ^{1}**H NMR (400 MHz,** $CDCl_3$**)** spectrum of **42.**

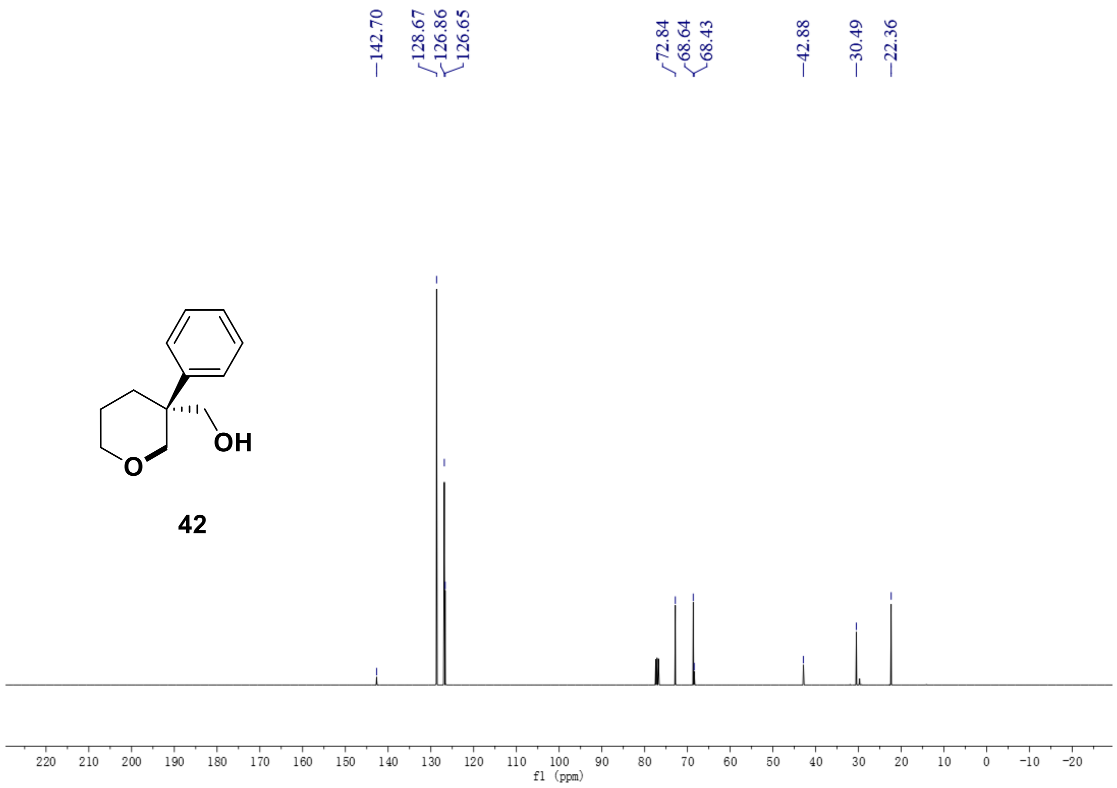

Supplementary Figure 151. ^{13}**C NMR (101 MHz, CDCl$_3$)** spectrum of **42.**

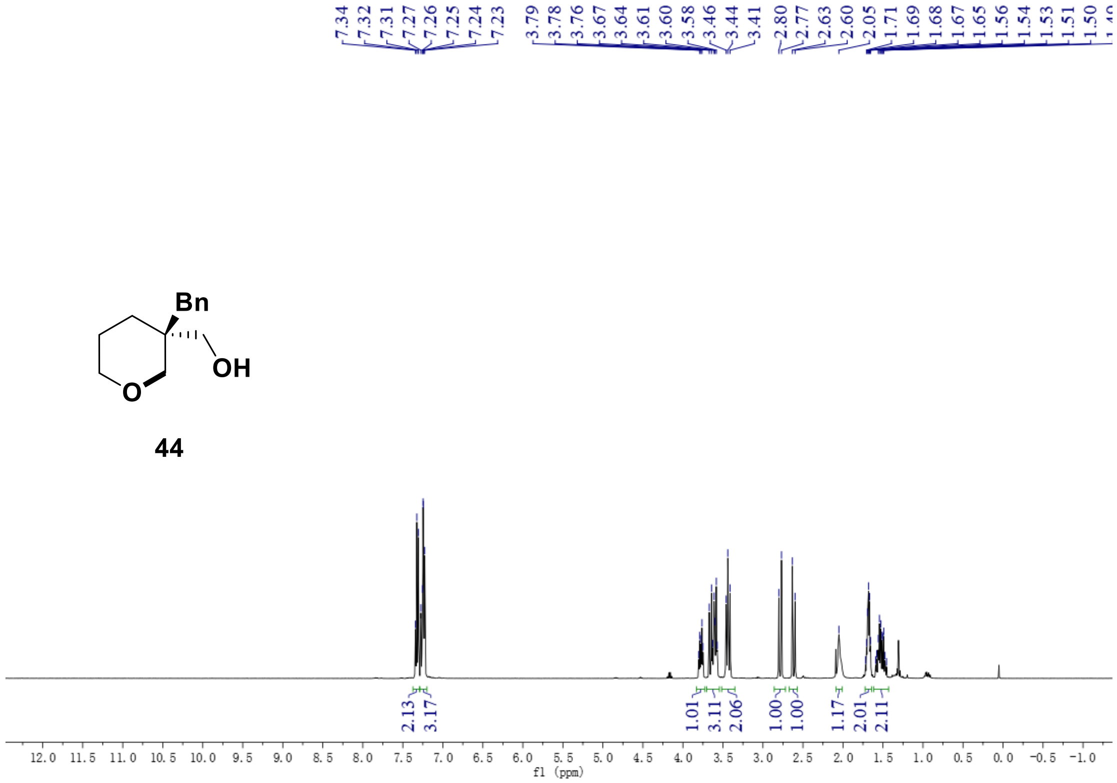

Supplementary Figure 152. ^{1}**H NMR (400 MHz, CDCl$_3$)** spectrum of **44.**

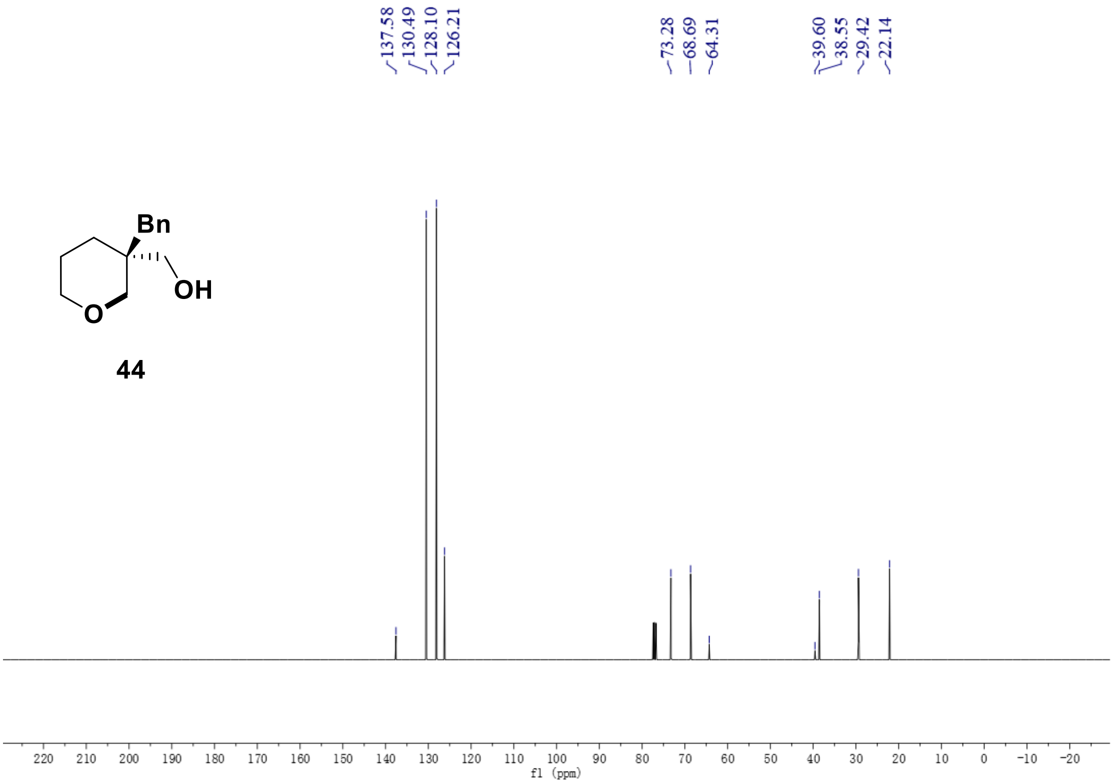

Supplementary Figure 153. ^{13}C NMR (101 MHz, $CDCl_3$) spectrum of **44.**

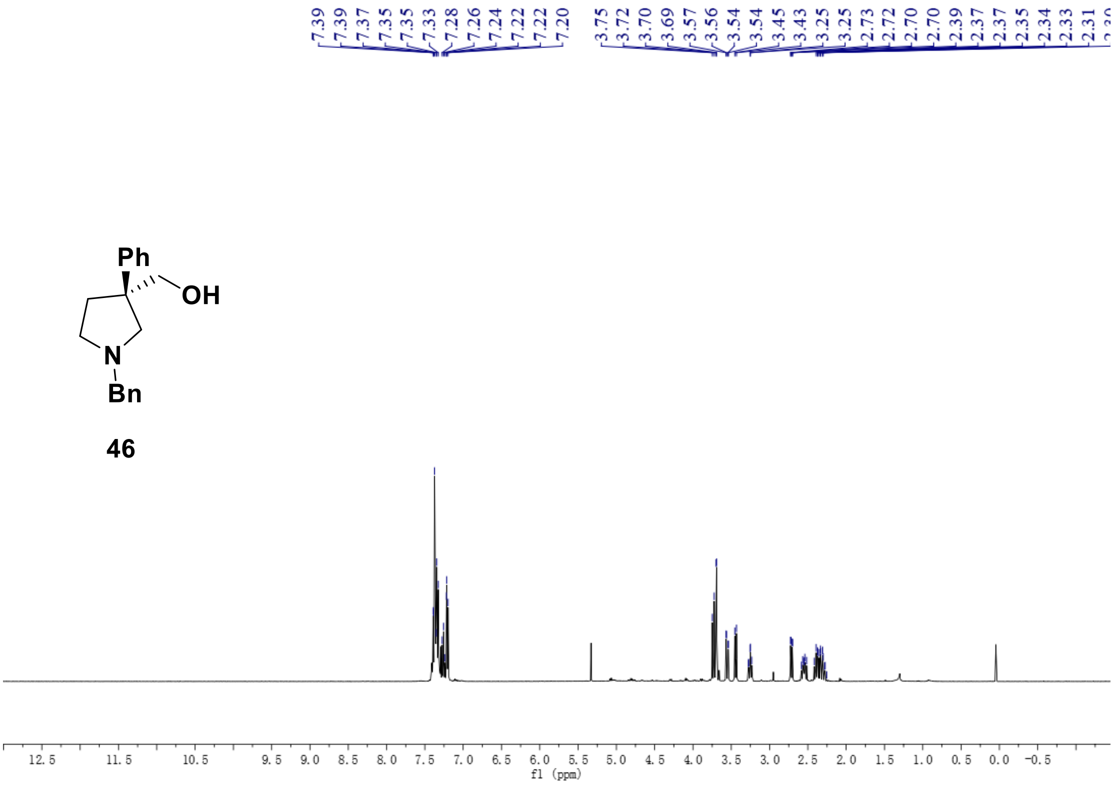

Supplementary Figure 154. ^{1}H NMR (400 MHz, $CDCl_3$) spectrum of **46.**

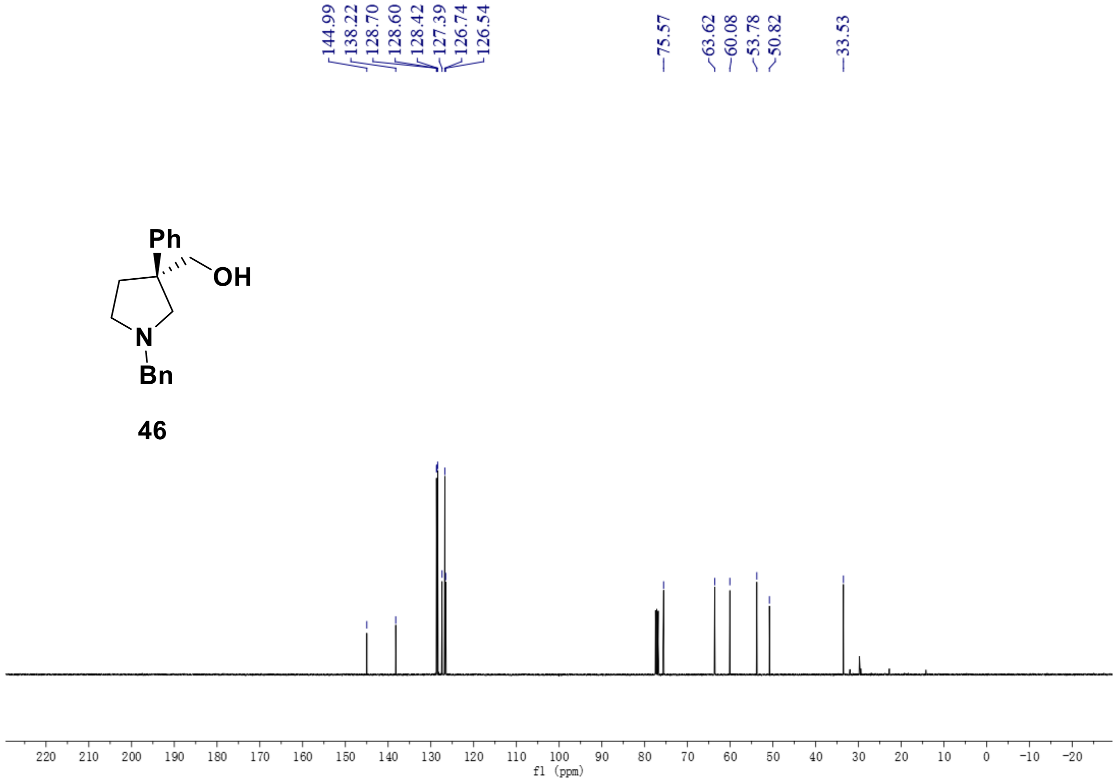

Supplementary Figure 155. ^{13}C NMR (101 MHz, $CDCl_3$) spectrum of **46.**

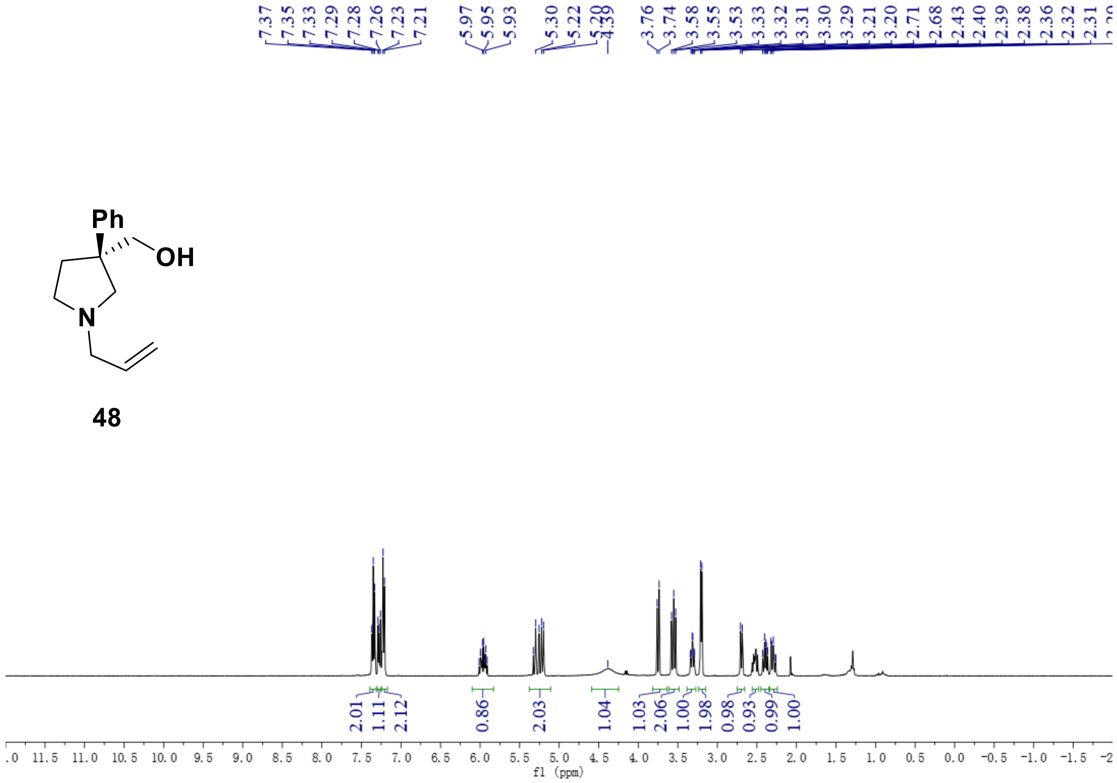

Supplementary Figure 156. ^{1}H NMR (400 MHz, $CDCl_3$) spectrum of **48.**

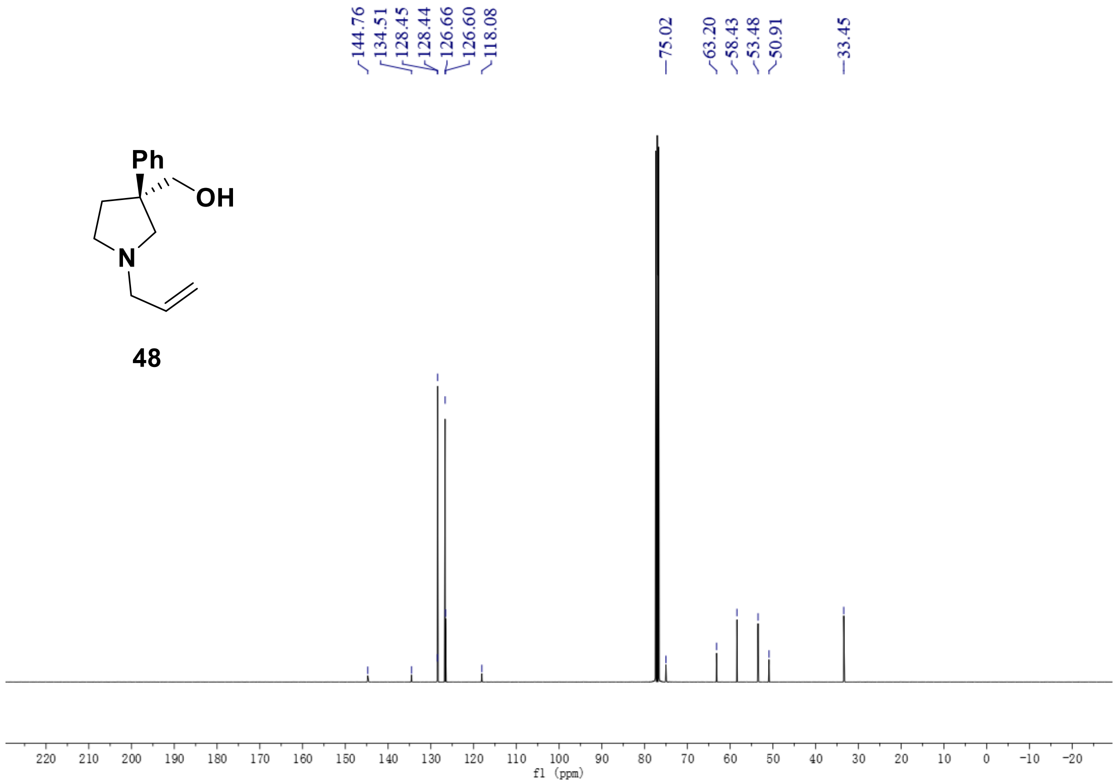

Supplementary Figure 157. ^{13}C NMR (400 MHz, $CDCl_3$) spectrum of **48.**

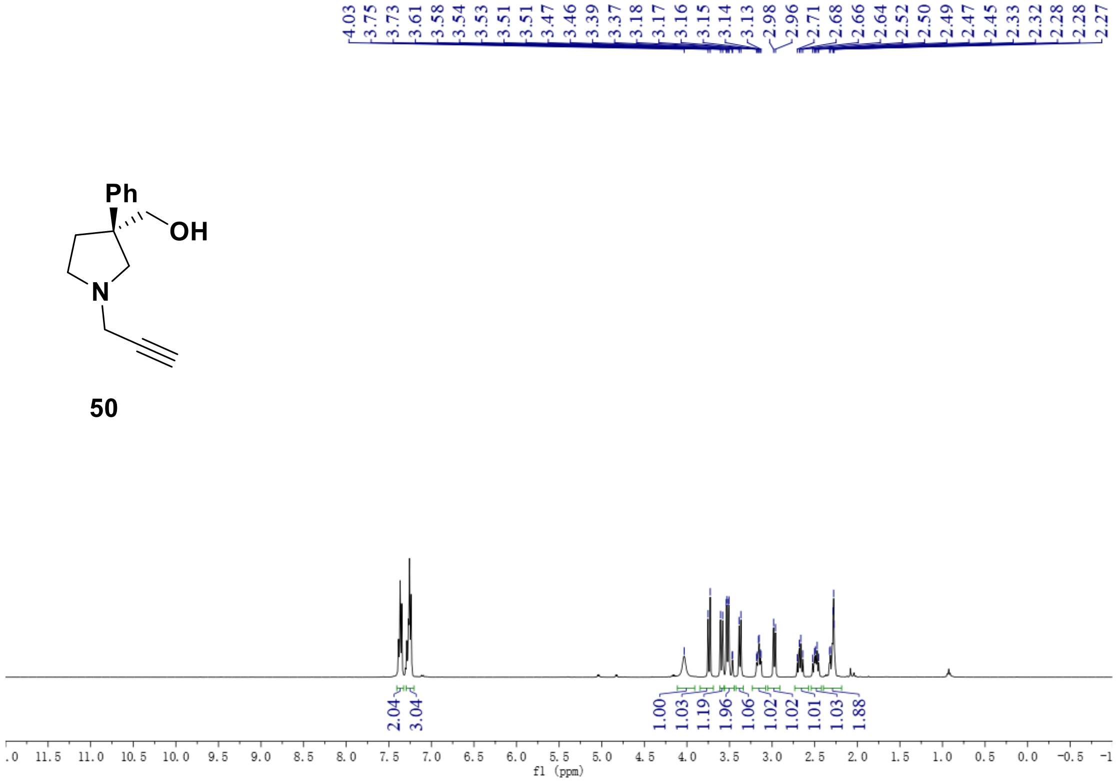

Supplementary Figure 158. ^{1}H NMR (400 MHz, $CDCl_3$) spectrum of **50.**

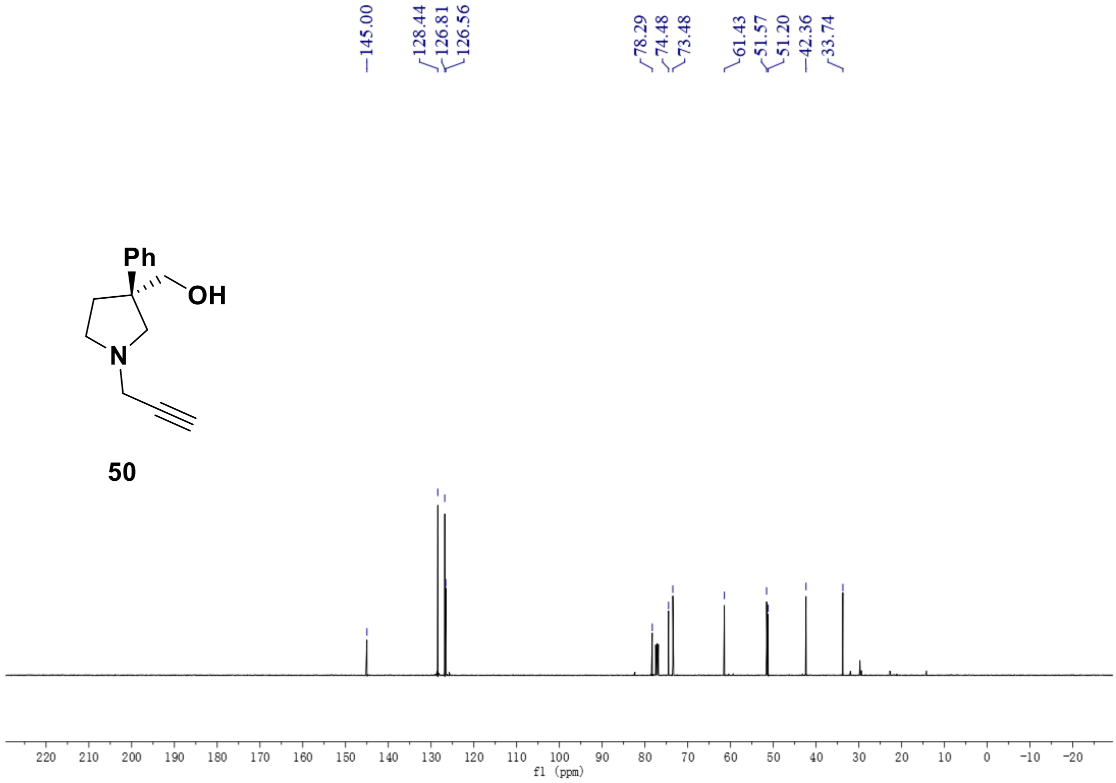

Supplementary Figure 159. ^{13}C NMR (101 MHz, CDCl$_3$) spectrum of **50**.

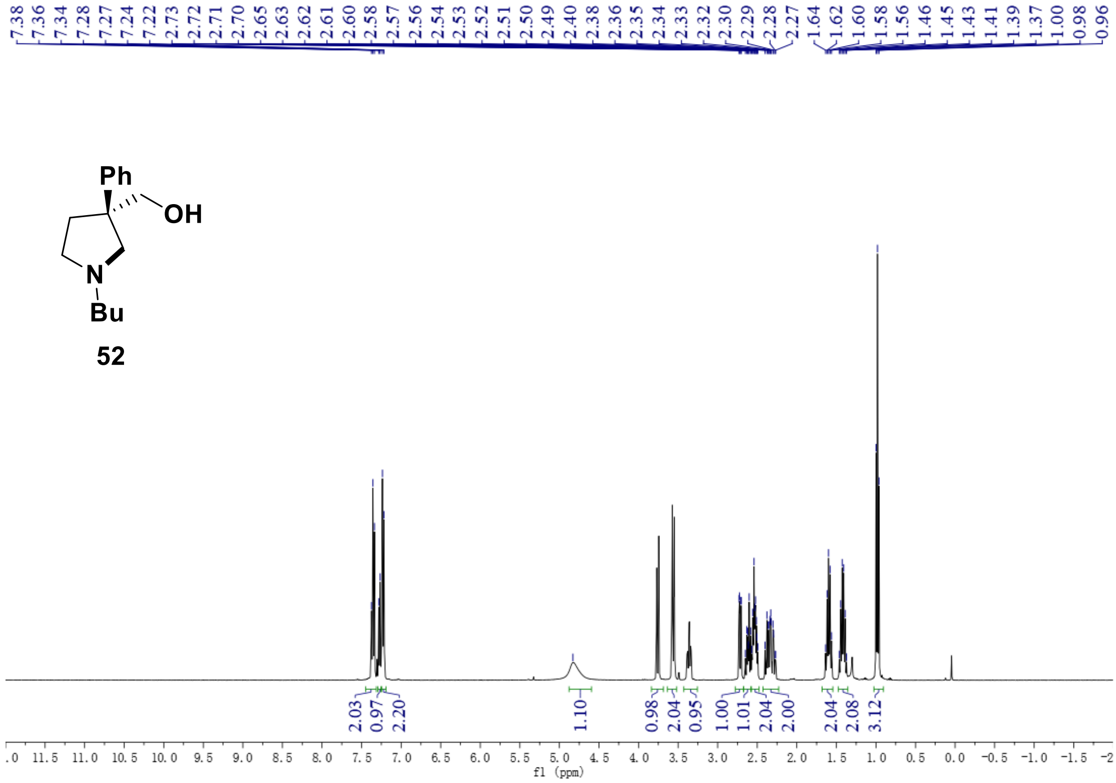

Supplementary Figure 160. ^{1}H NMR (400 MHz, CDCl$_3$) spectrum of **52**.

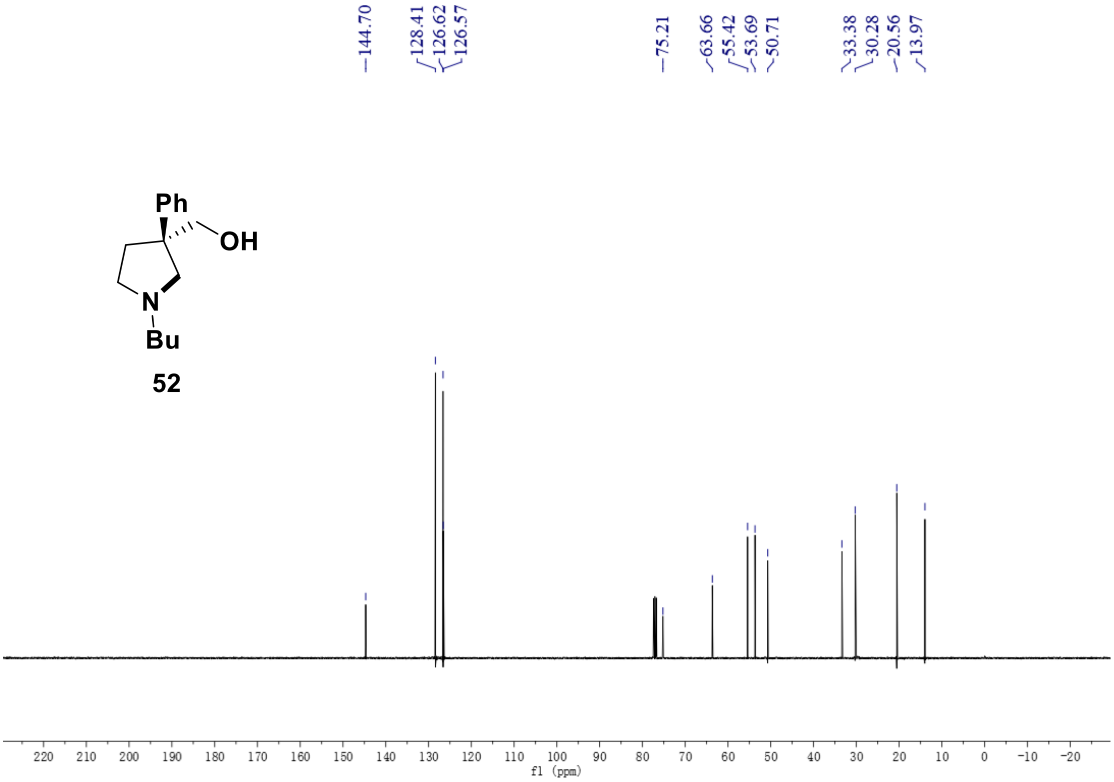

Supplementary Figure 161. ^{13}**C NMR (101 MHz, CDCl**$_3$**)** spectrum of **52.**

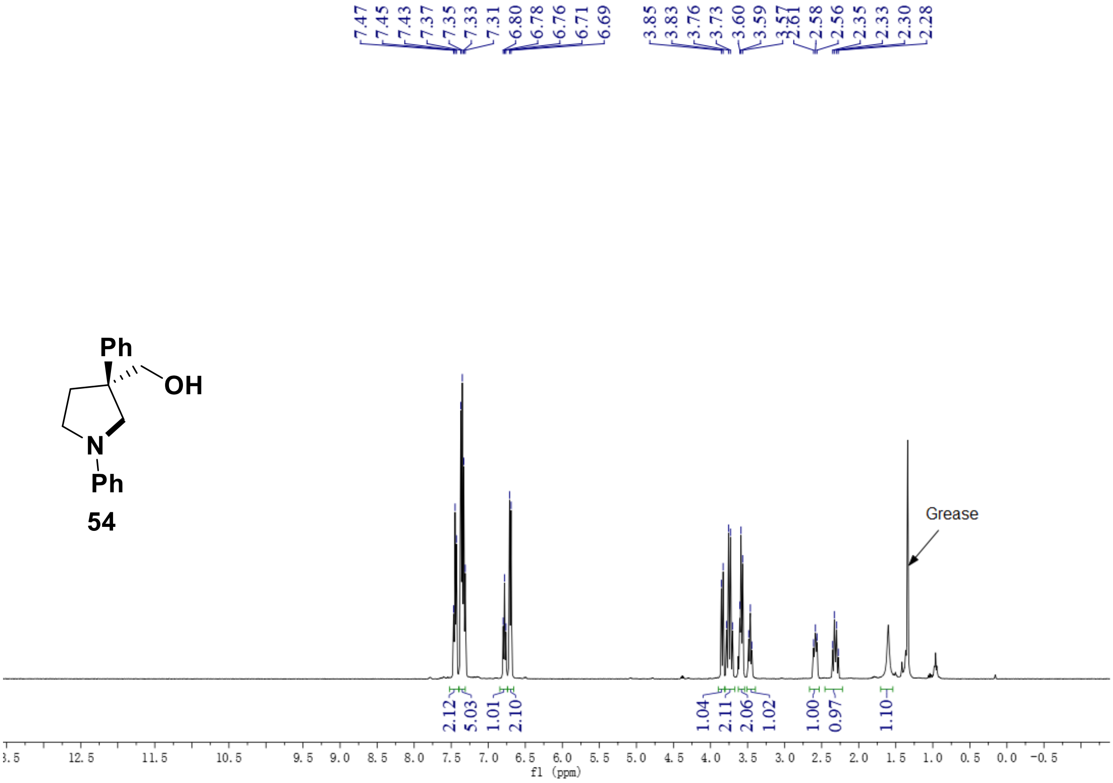

Supplementary Figure 162. ^{1}**H NMR (400 MHz, CDCl**$_3$**)** spectrum of **54.**

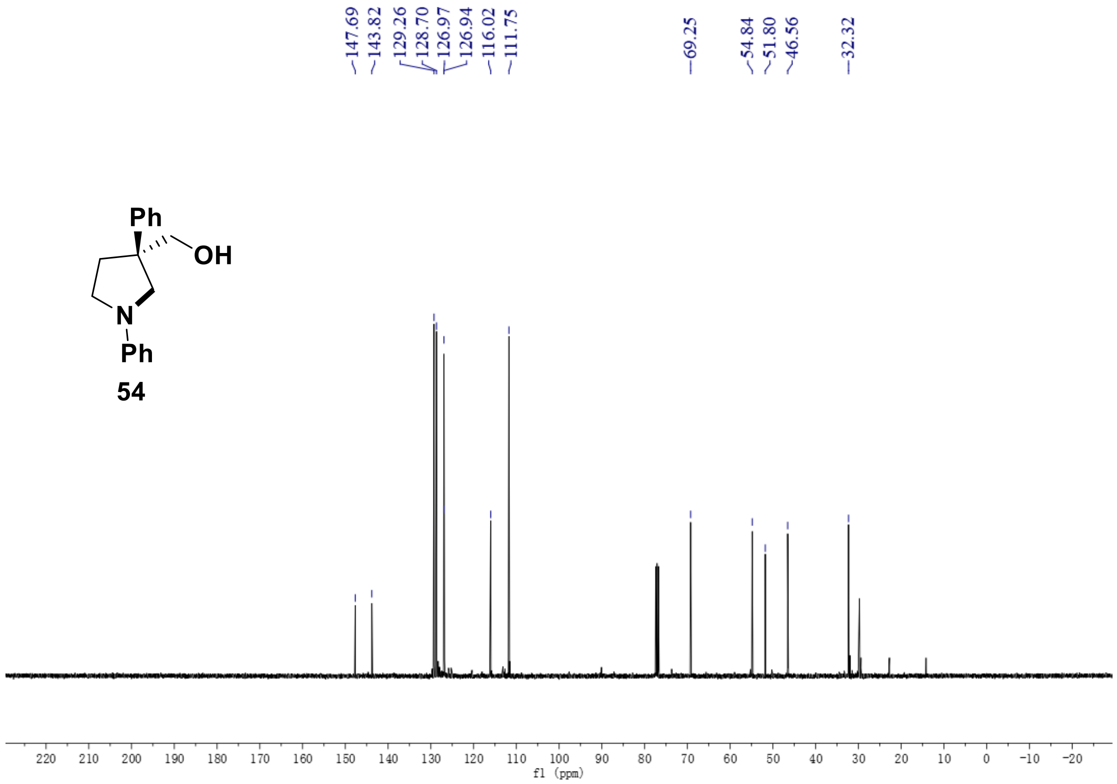

Supplementary Figure 163. ^{13}**C NMR (101 MHz, CDCl**$_3$**)** spectrum of **54.**

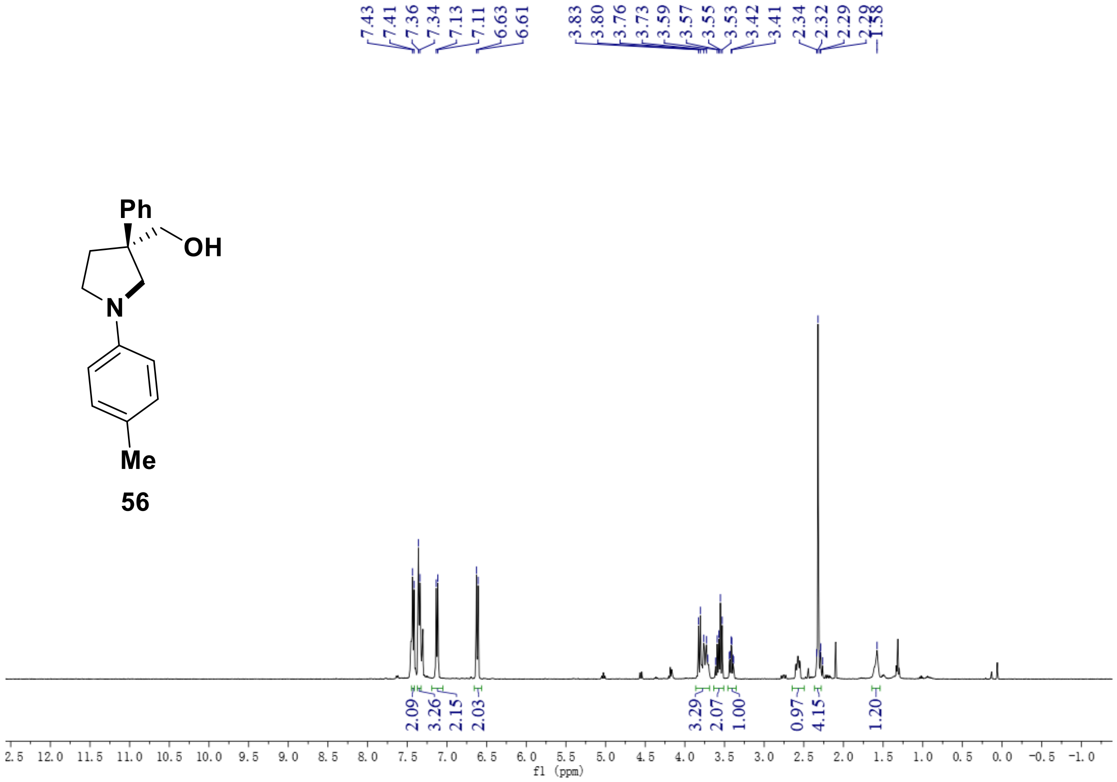

Supplementary Figure 164. ^{1}**H NMR (400 MHz, CDCl**$_3$**)** spectrum of **56.**

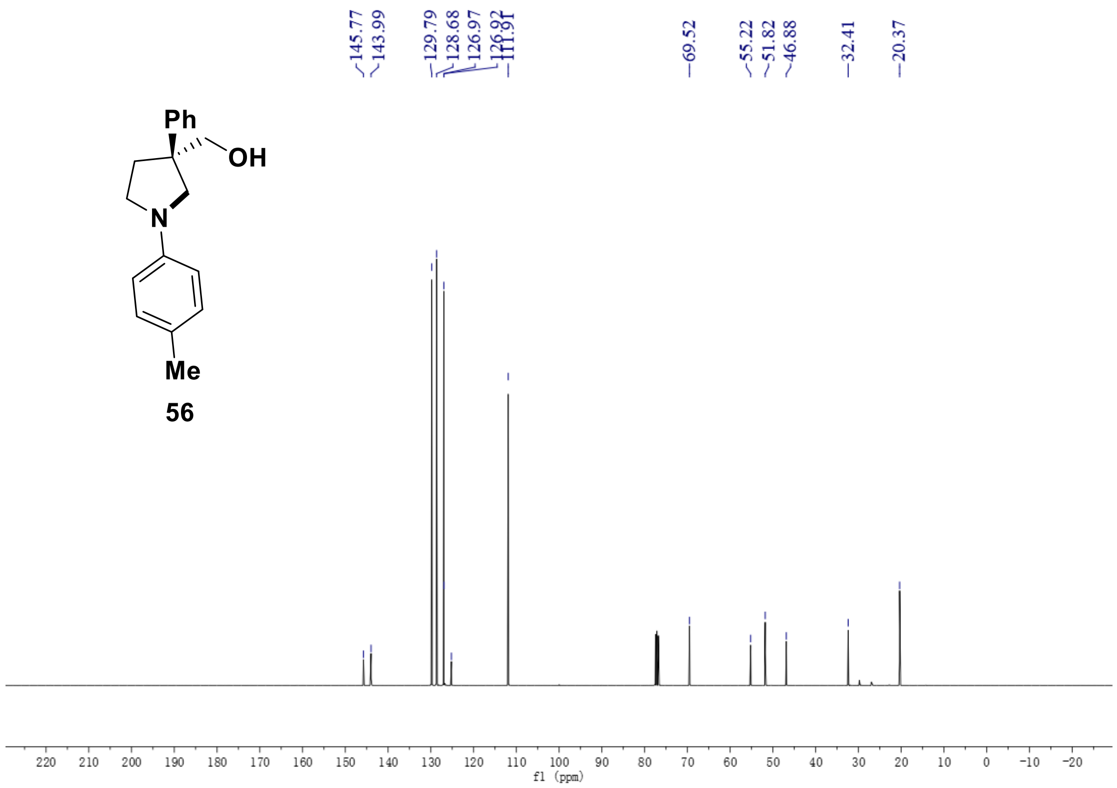

Supplementary Figure 165. ^{13}C NMR (101 MHz, $CDCl_3$) spectrum of **56.**

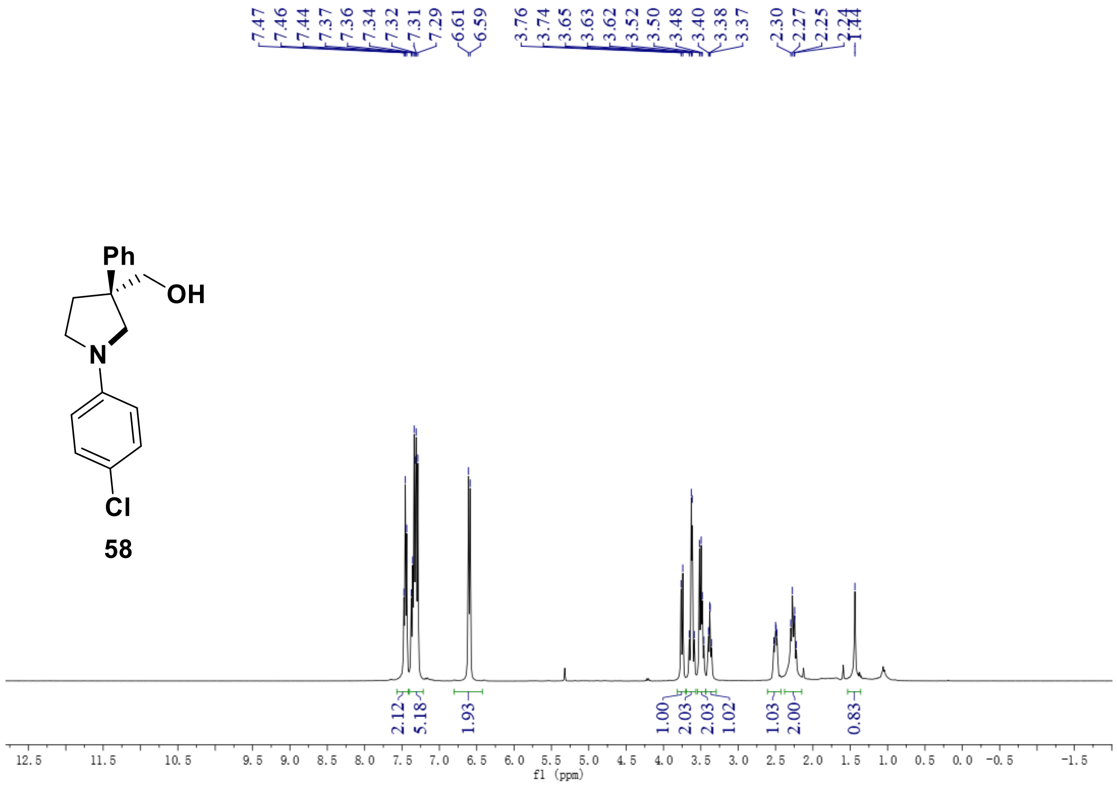

Supplementary Figure 166. ^{1}H NMR (400 MHz, $CDCl_3$) spectrum of **58.**

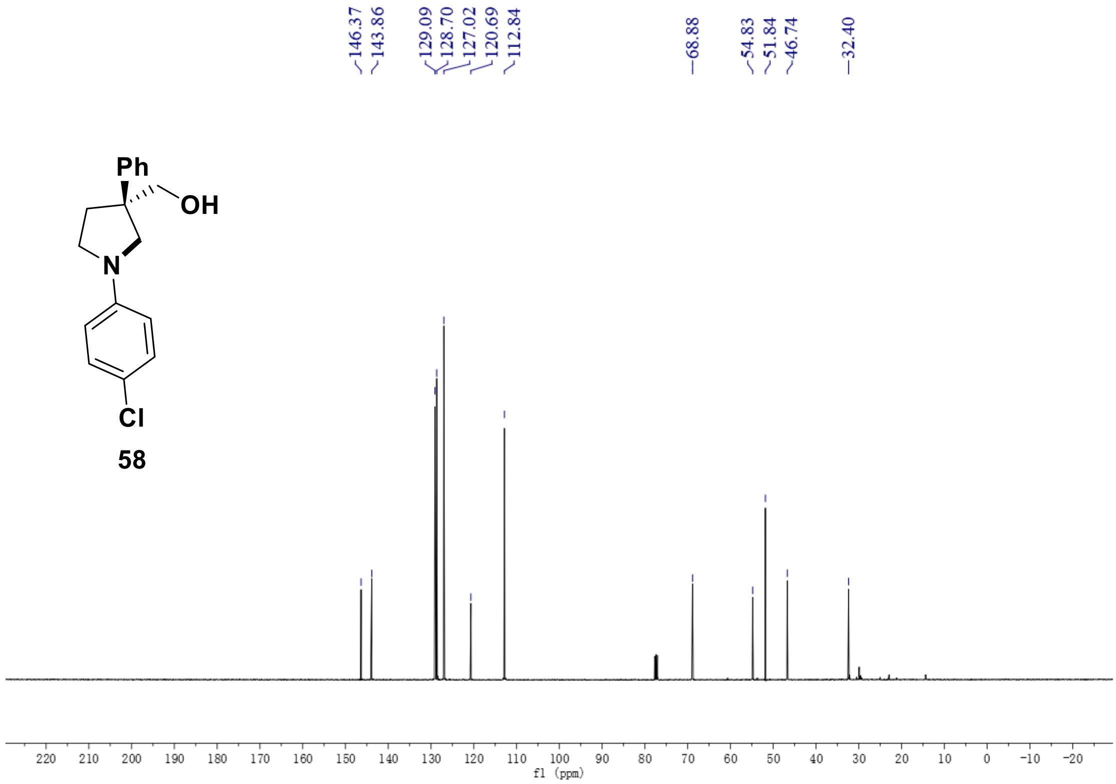

Supplementary Figure 167. ^{13}C NMR (101 MHz, $CDCl_3$) spectrum of **58.**

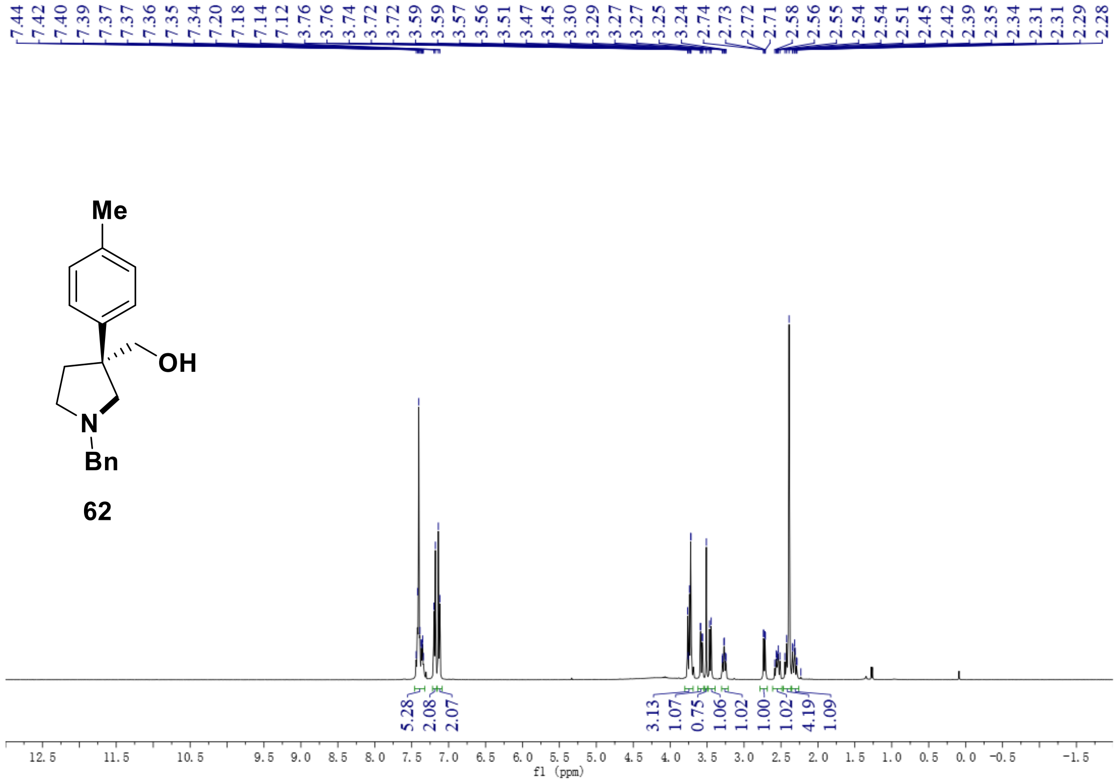

Supplementary Figure 168. ^{1}H NMR (400 MHz, $CDCl_3$) spectrum of **62.**

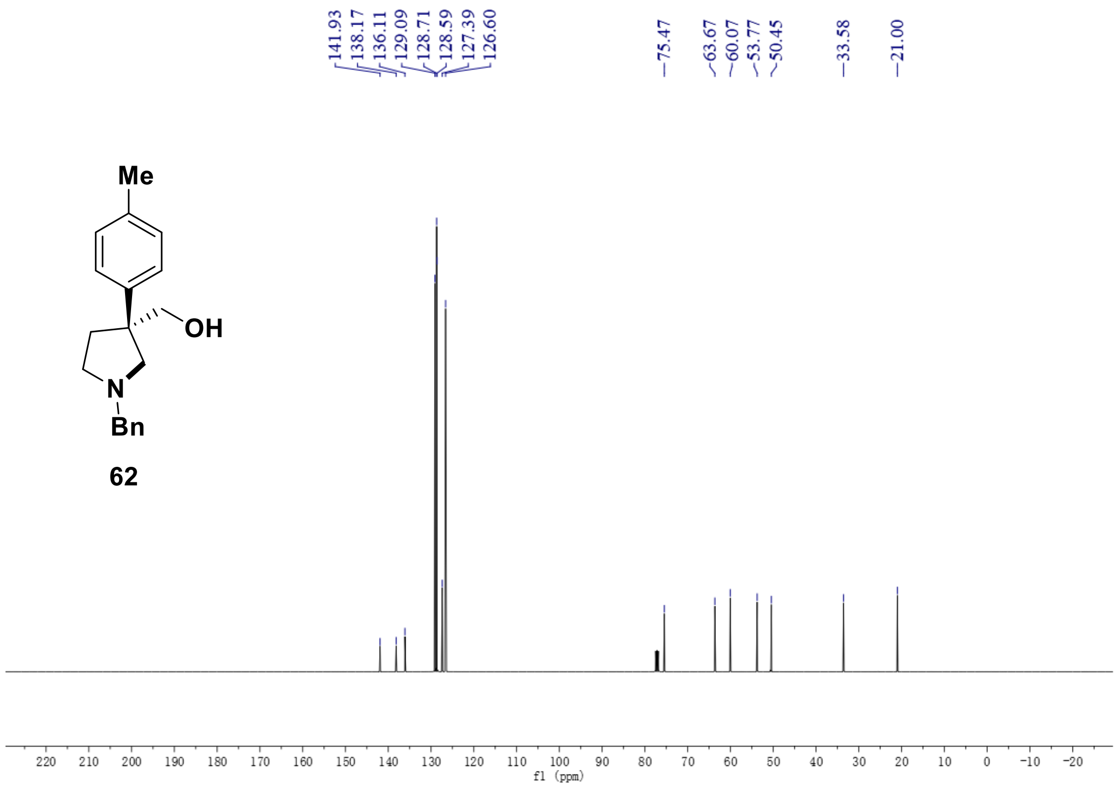

Supplementary Figure 169. **^{13}C NMR (101 MHz, $CDCl_3$)** spectrum of **62.**

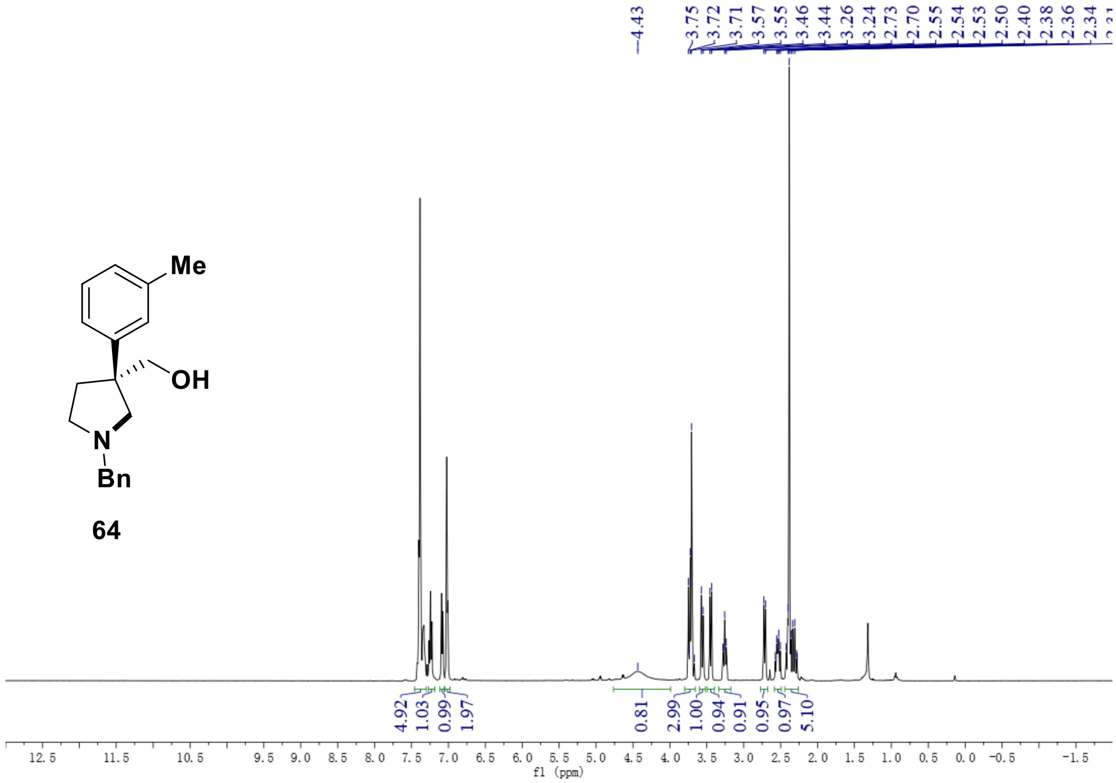

Supplementary Figure 170. **^{1}H NMR (400 MHz, $CDCl_3$)** spectrum of **64.**

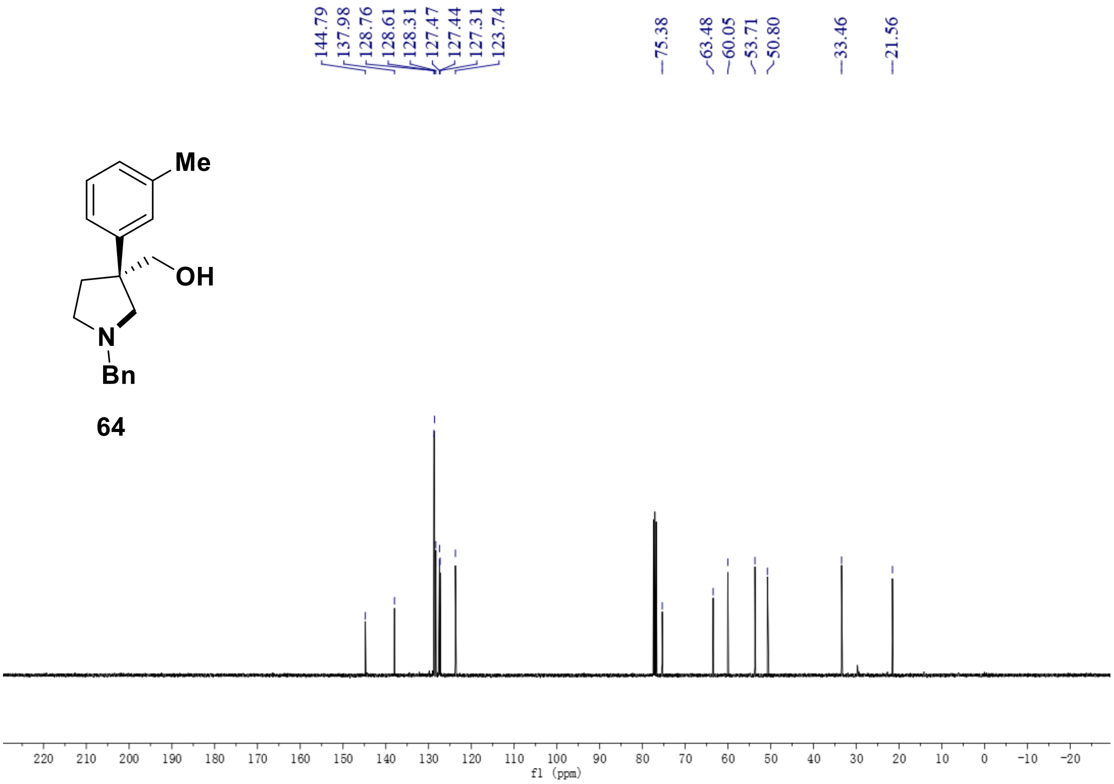

Supplementary Figure 171. **^{13}C NMR (101 MHz, $CDCl_3$)** spectrum of **64.**

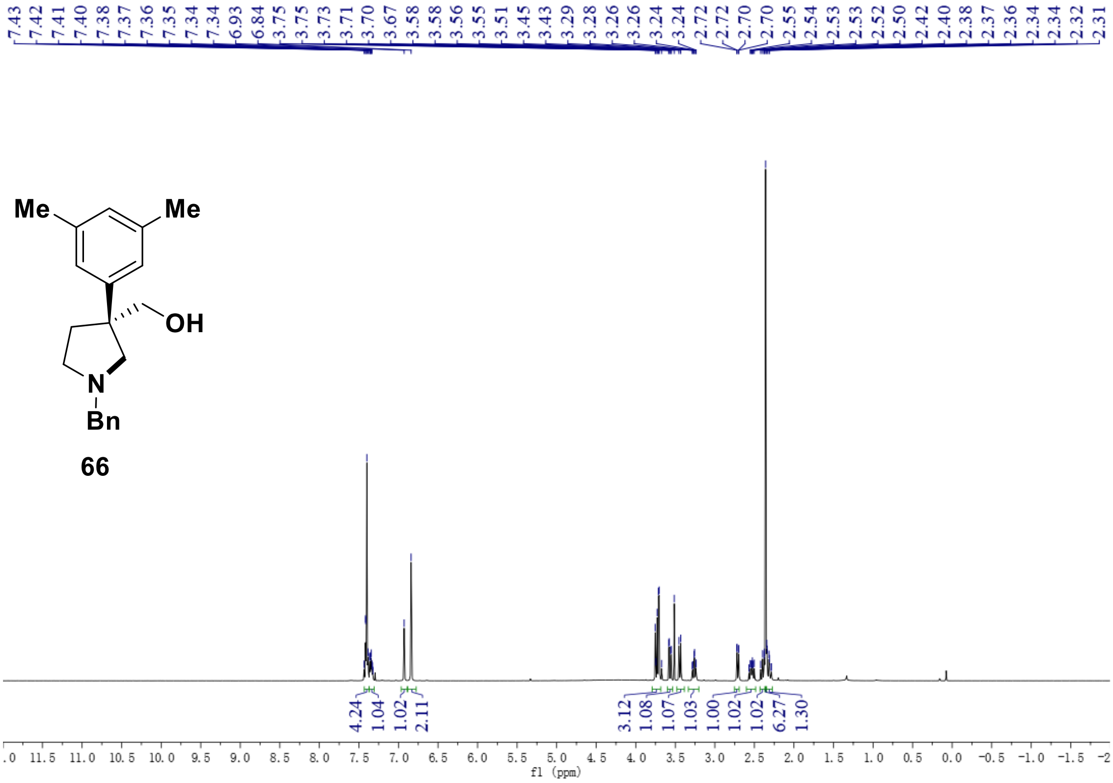

Supplementary Figure 172. **^{1}H NMR (400 MHz, $CDCl_3$)** spectrum of **66.**

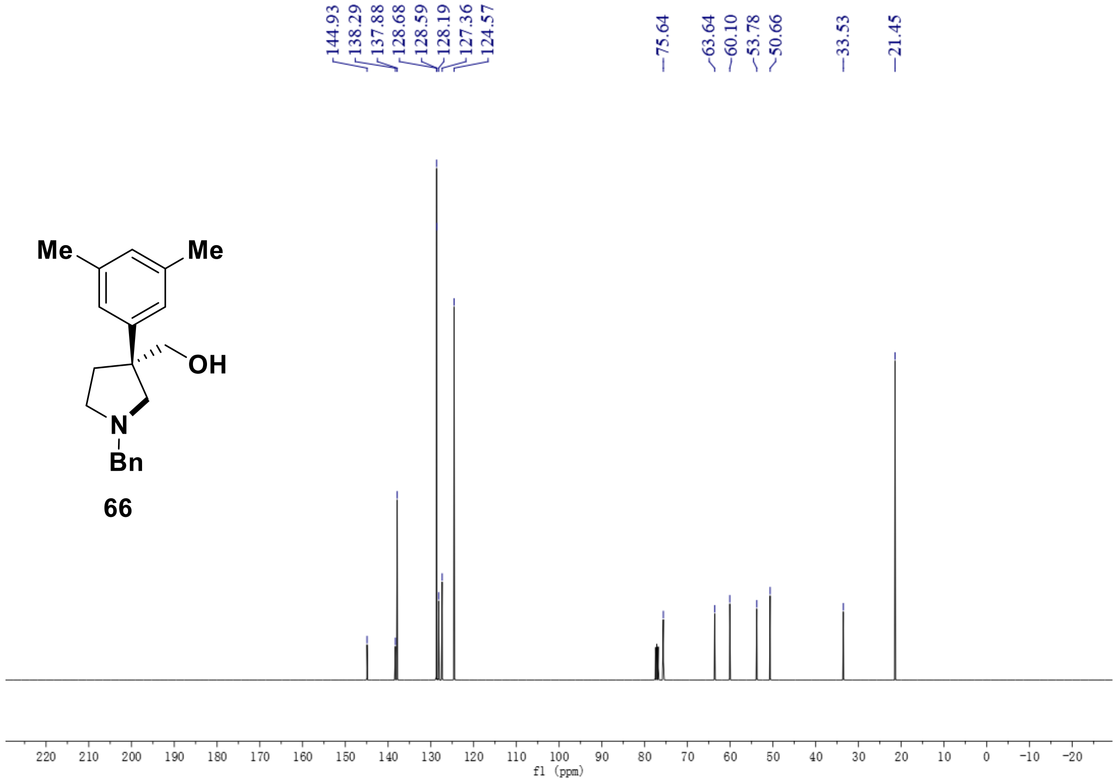

Supplementary Figure 173. ^{13}**C NMR (101 MHz, CDCl$_3$)** spectrum of **66.**

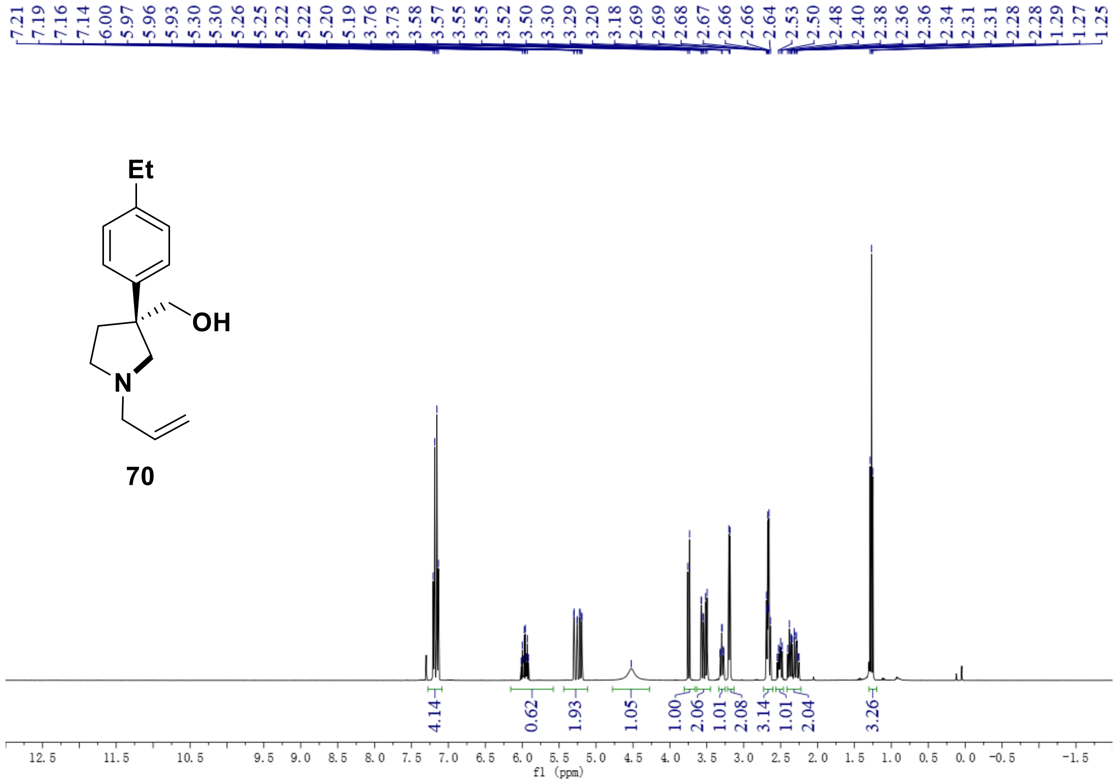

Supplementary Figure 174. ^{1}**H NMR (400 MHz, CDCl$_3$)** spectrum of **70.**

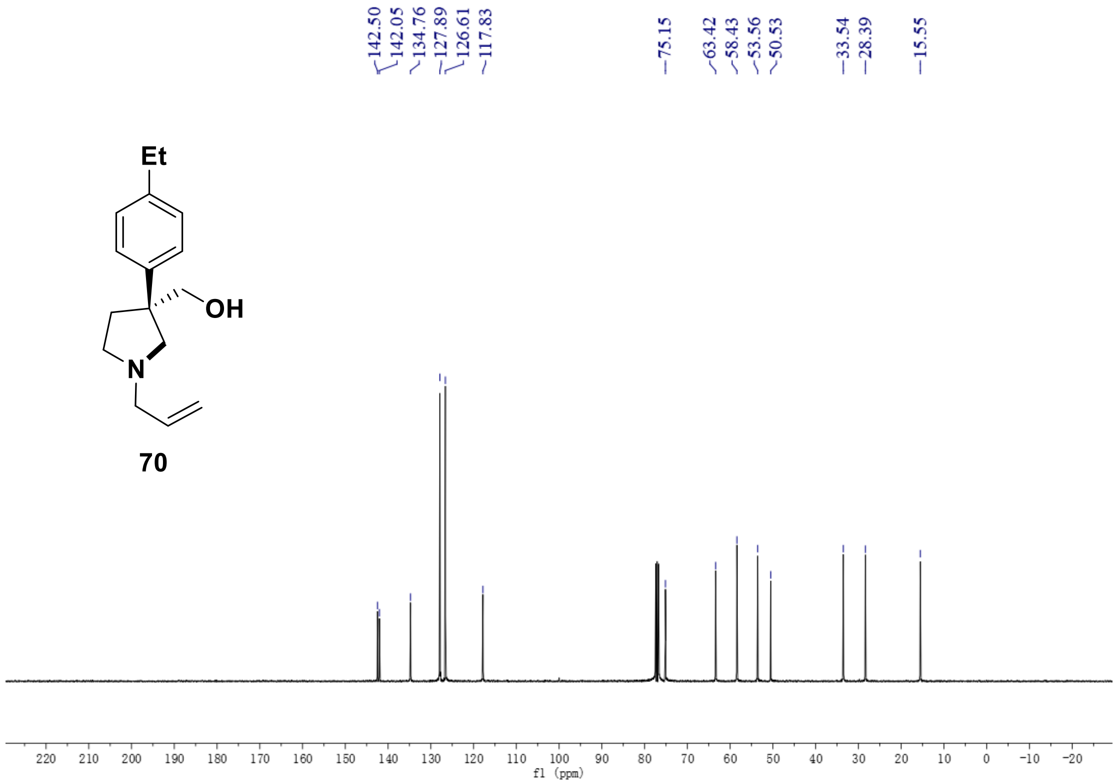

Supplementary Figure 175. ^{13}**C NMR (101 MHz, CDCl$_3$)** spectrum of **70.**

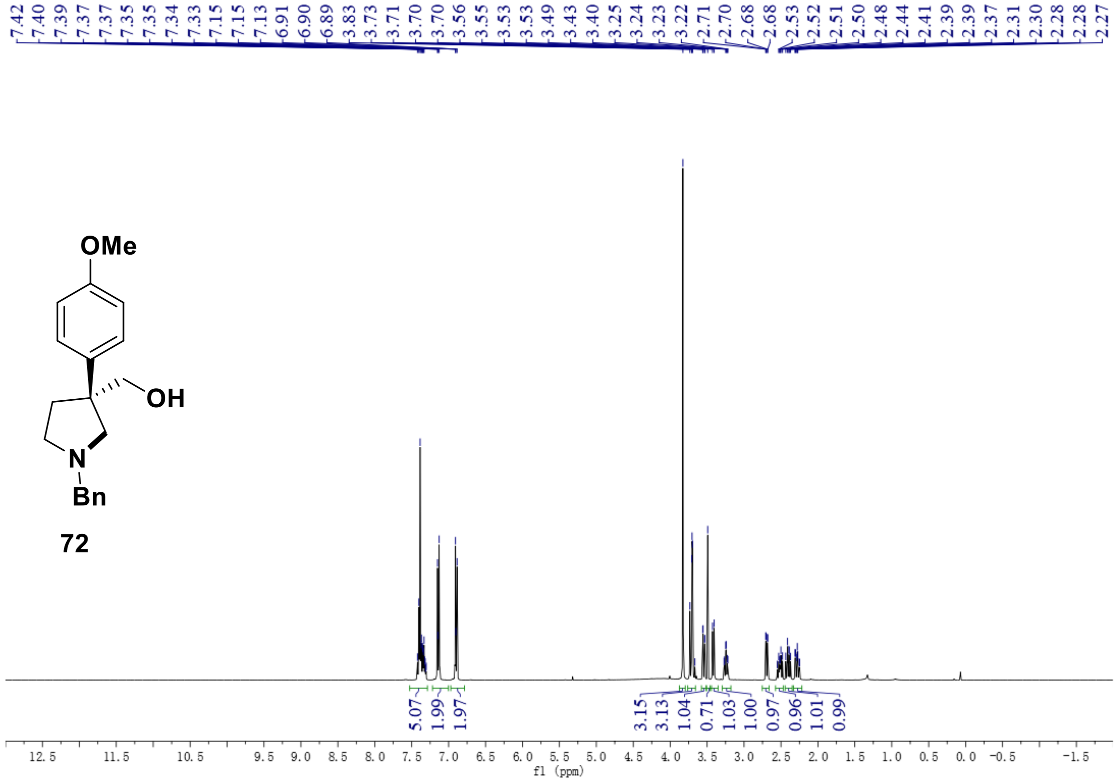

Supplementary Figure 176. ^{1}**H NMR (400 MHz, CDCl$_3$)** spectrum of **72.**

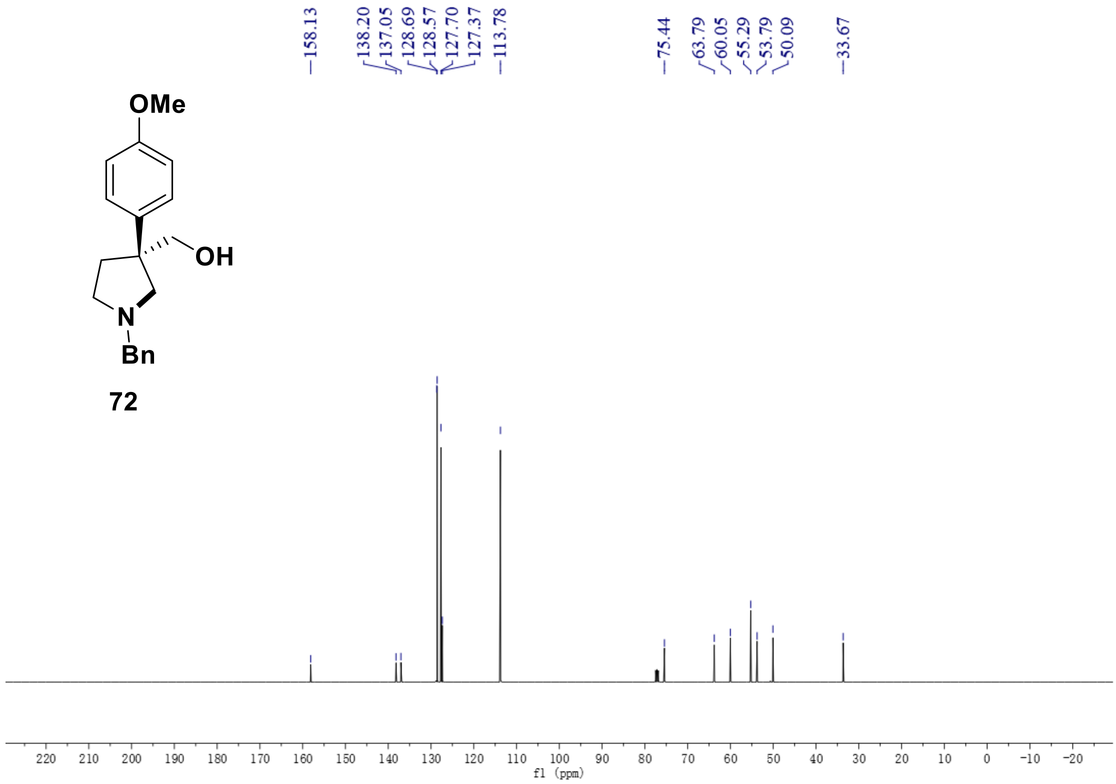

Supplementary Figure 177. ^{13}**C NMR (101 MHz, CDCl$_3$)** spectrum of **72.**

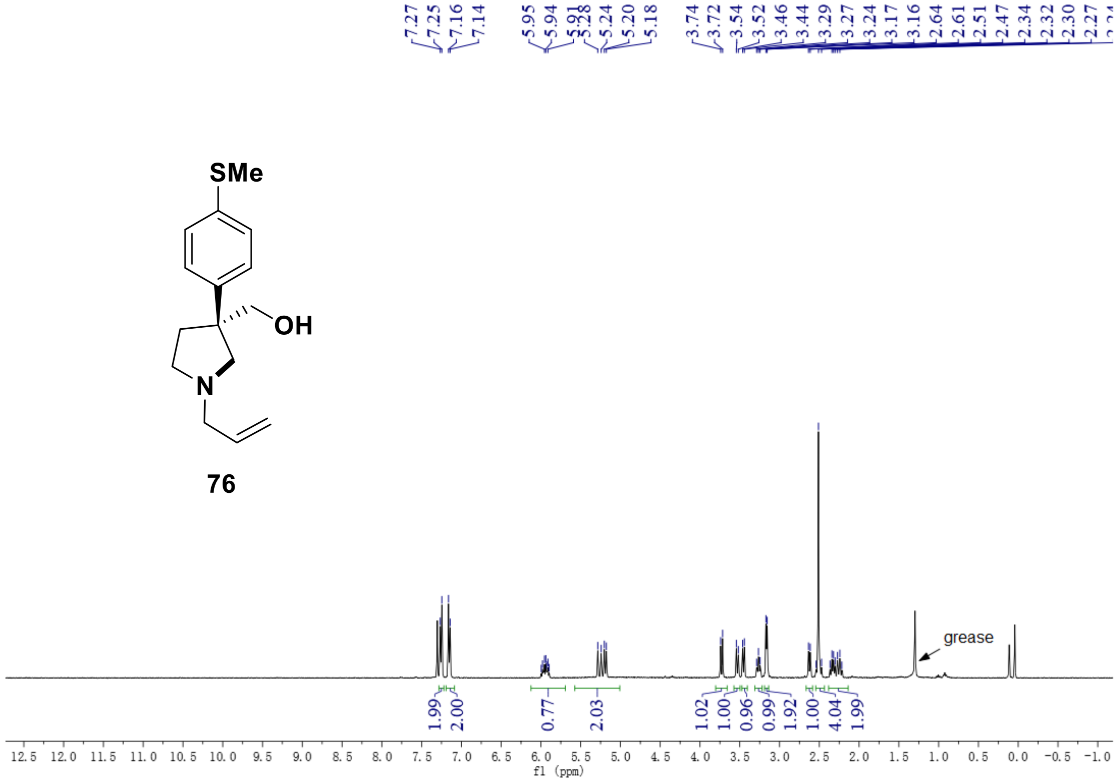

Supplementary Figure 178. ^{1}**H NMR (400 MHz, CDCl$_3$)** spectrum of **76.**

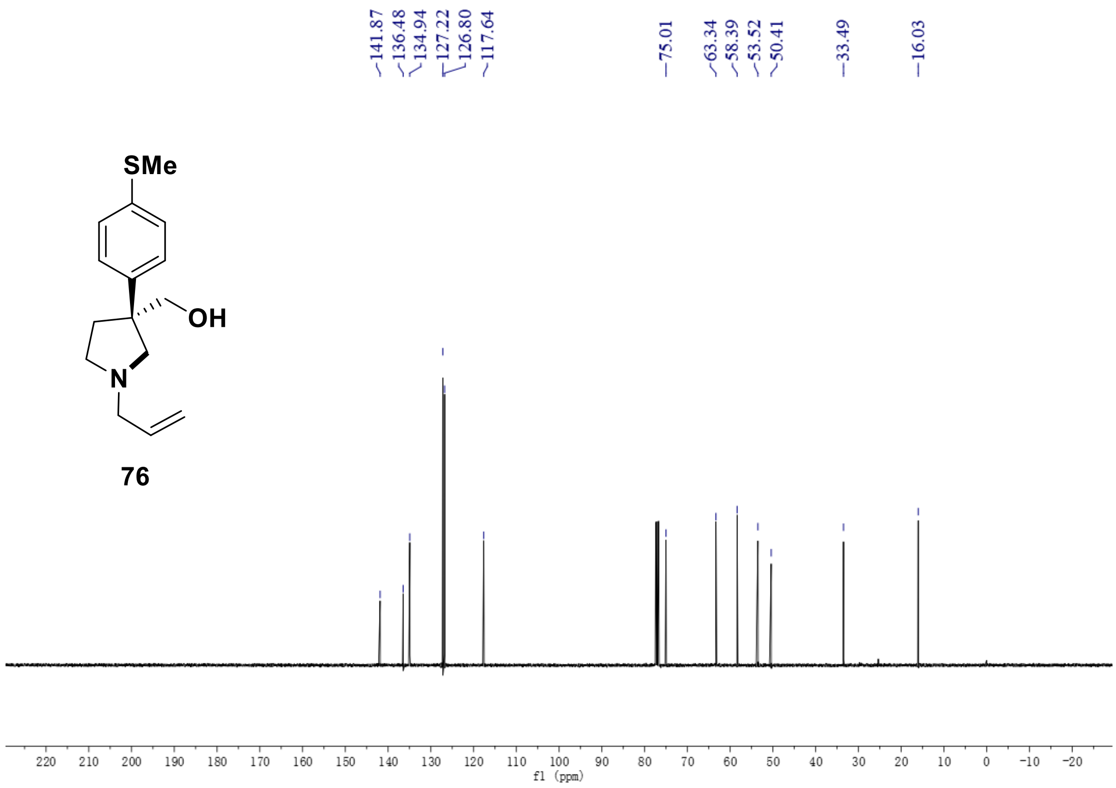

Supplementary Figure 179. ^{13}**C NMR (101 MHz, CDCl$_3$)** spectrum of **76.**

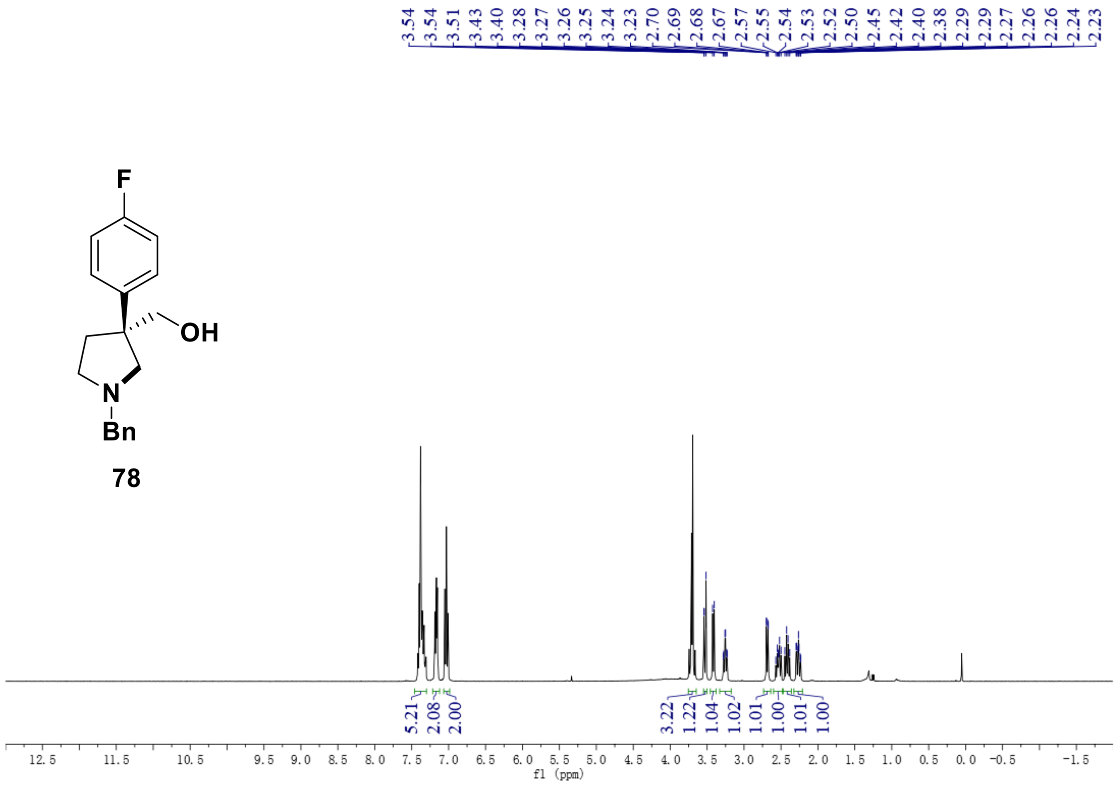

Supplementary Figure 180. ^{1}**H NMR (400 MHz, CDCl$_3$)** spectrum of **78.**

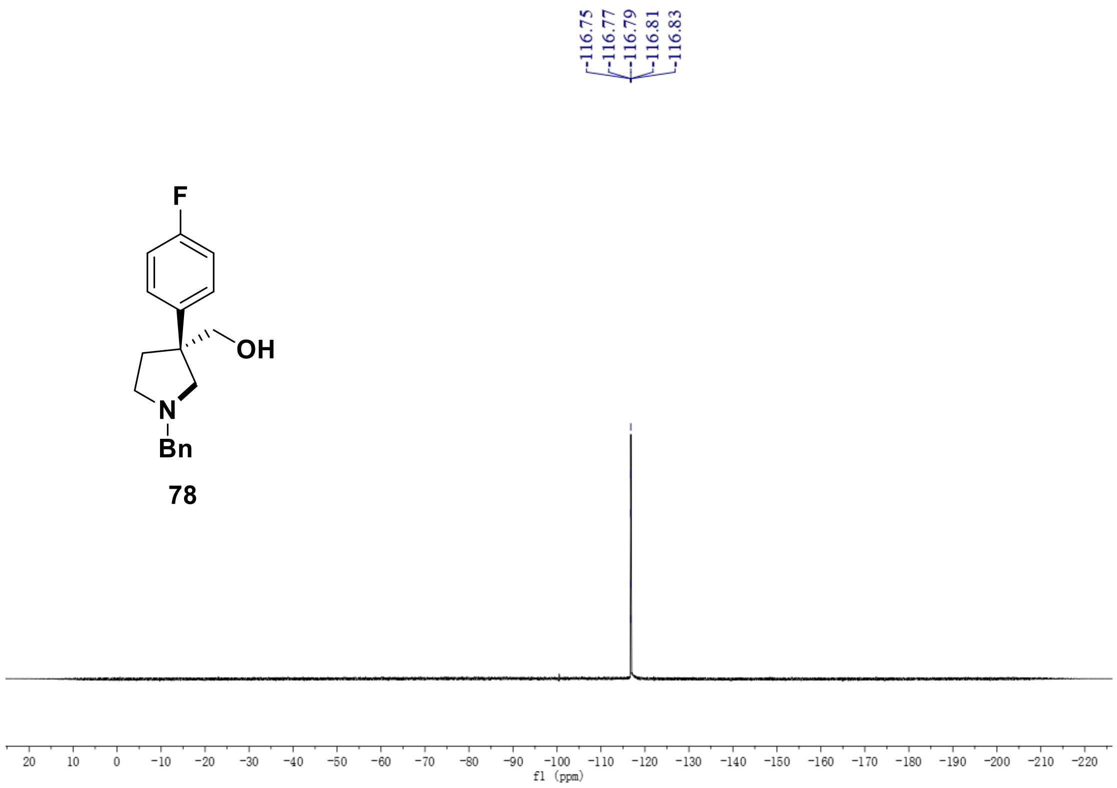

Supplementary Figure 181. ^{19}**F NMR (376 MHz, CDCl$_3$)** spectrum of **78.**

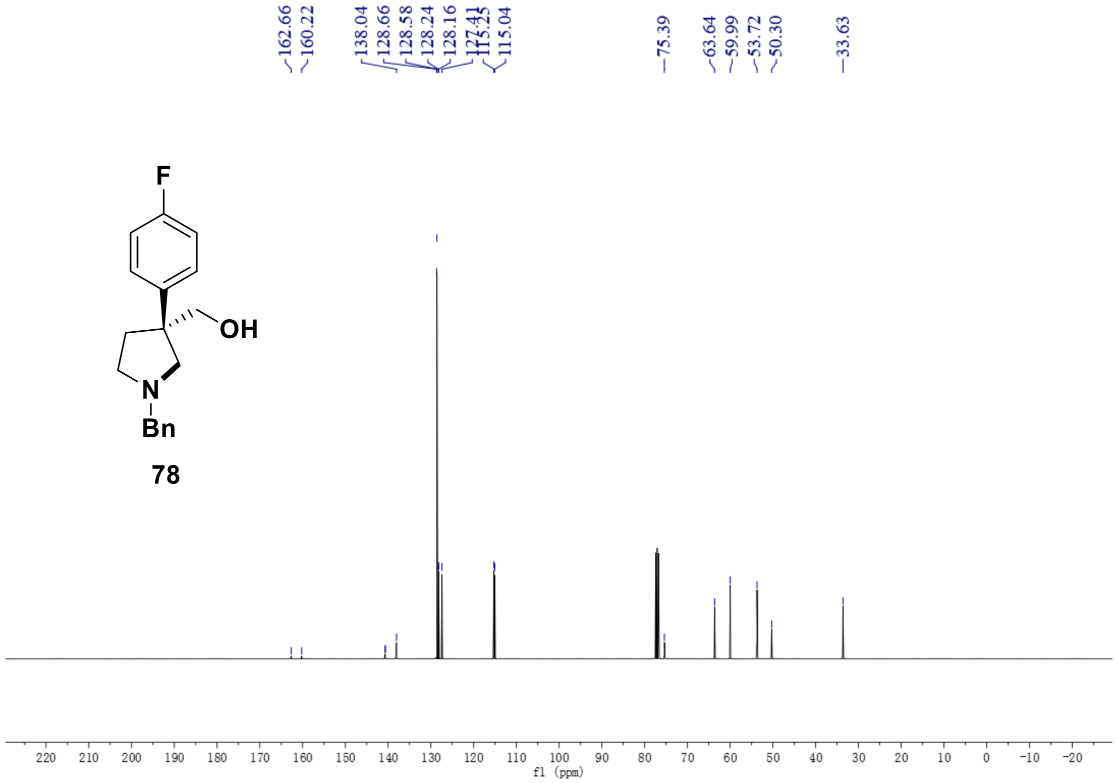

Supplementary Figure 182. ^{13}**C NMR (101 MHz, CDCl$_3$)** spectrum of **78.**

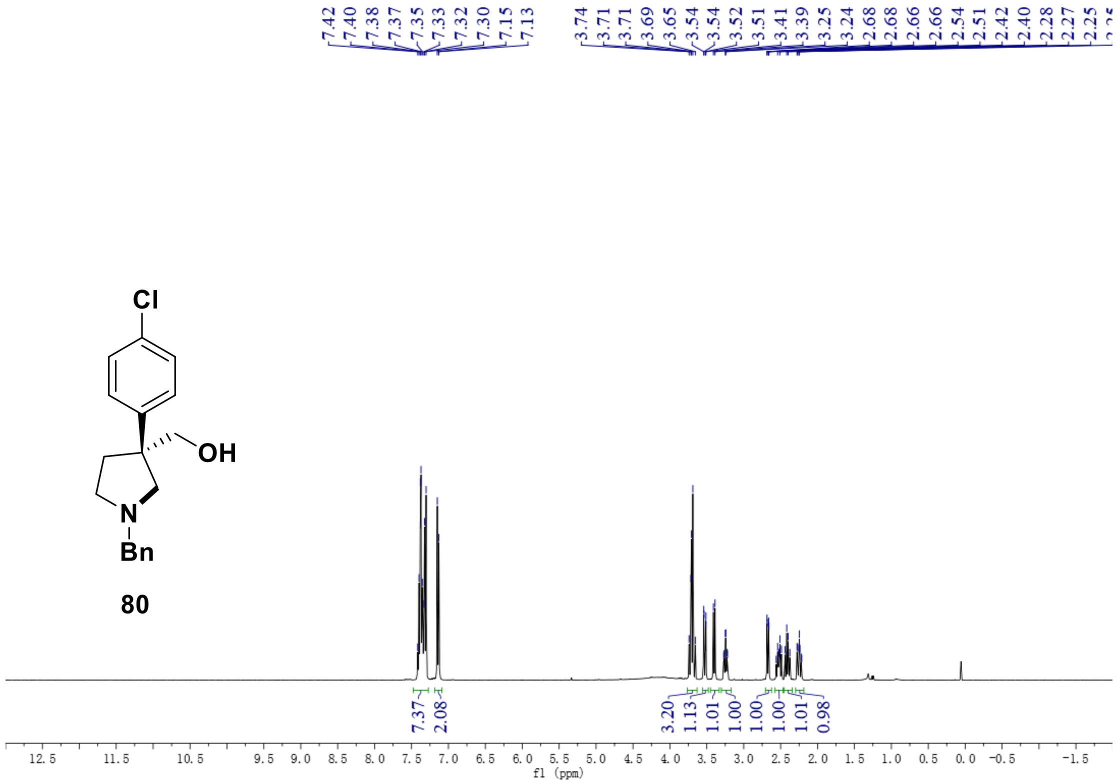

Supplementary Figure 183. **^{1}H NMR (400 MHz, $CDCl_3$)** spectrum of **80.**

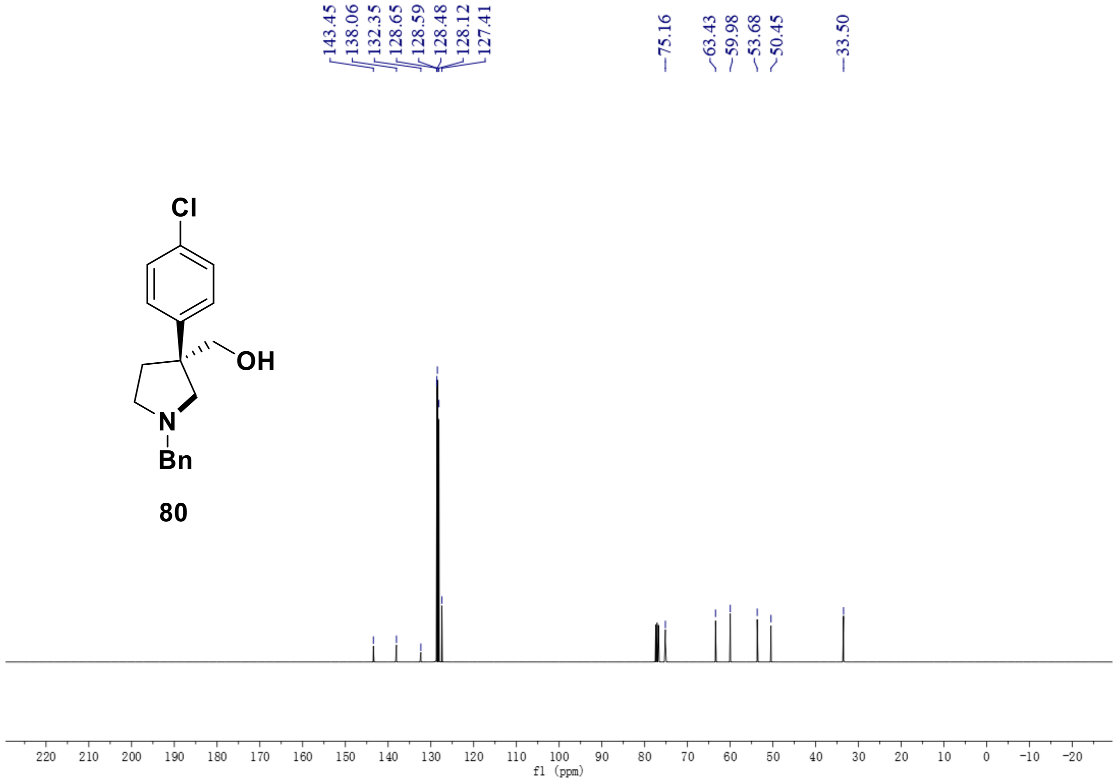

Supplementary Figure 184. **^{13}C NMR (101 MHz, $CDCl_3$)** spectrum of **80.**

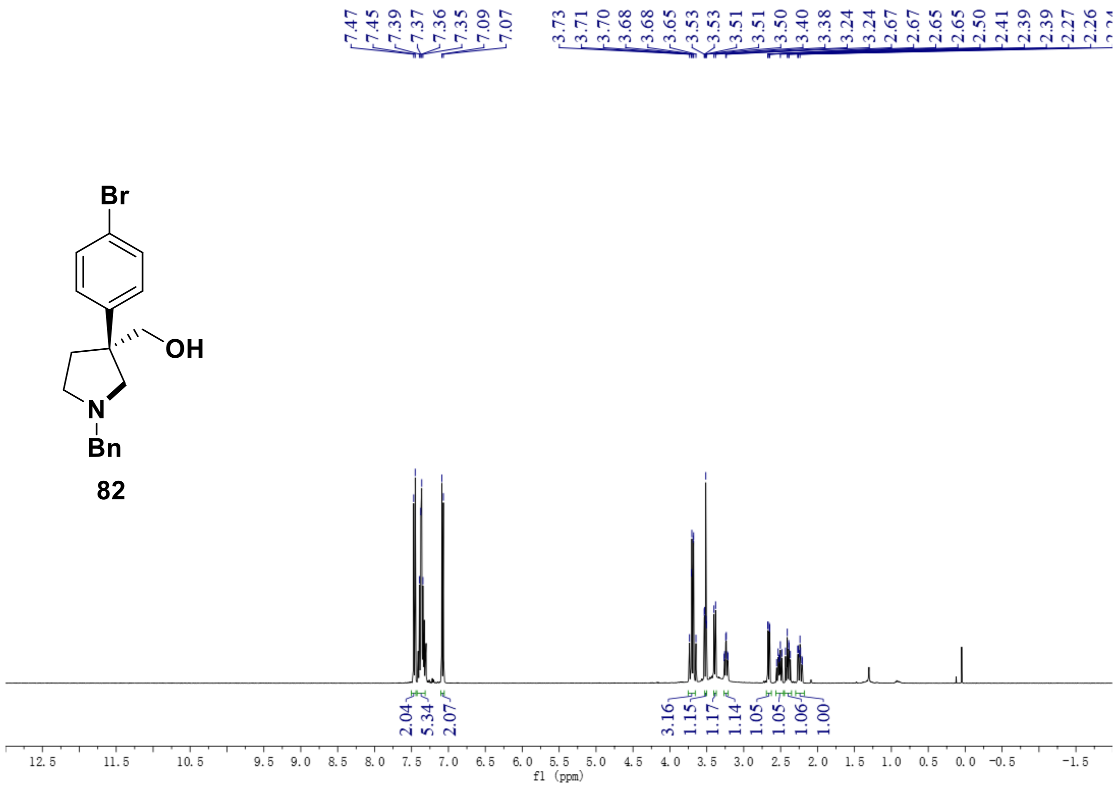

Supplementary Figure 185. ^{1}H NMR (400 MHz, $CDCl_3$) spectrum of **82.**

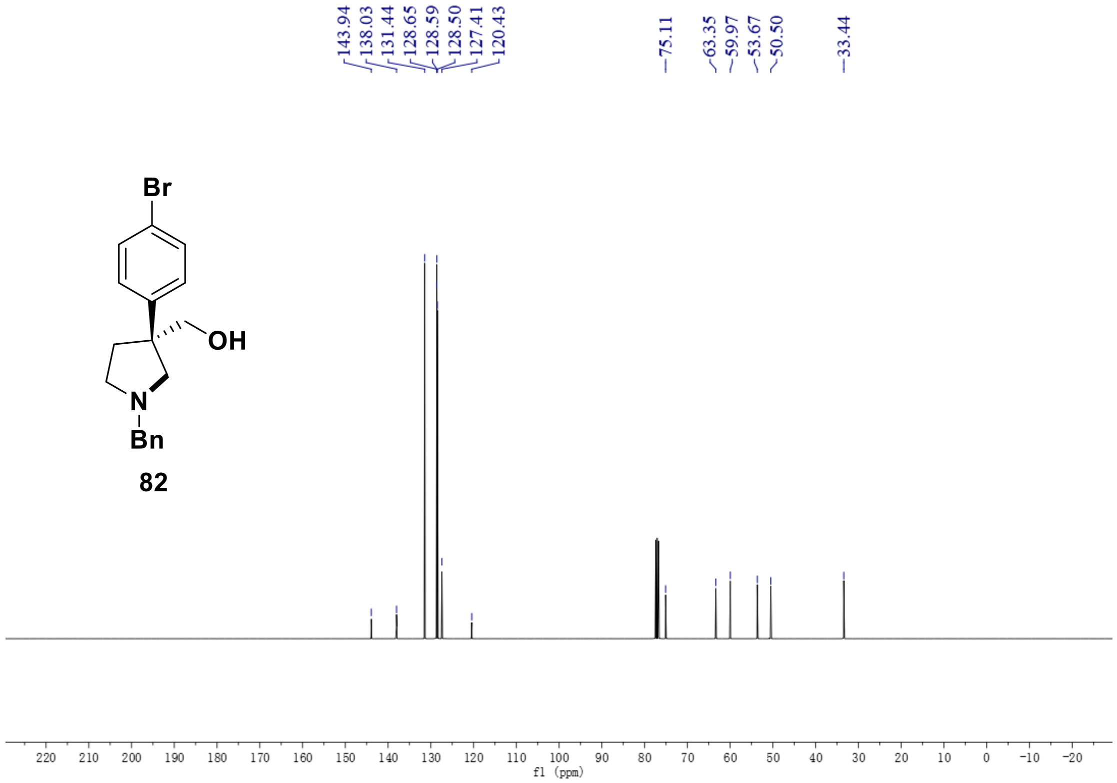

Supplementary Figure 186. ^{13}C NMR (101 MHz, $CDCl_3$) spectrum of **82.**

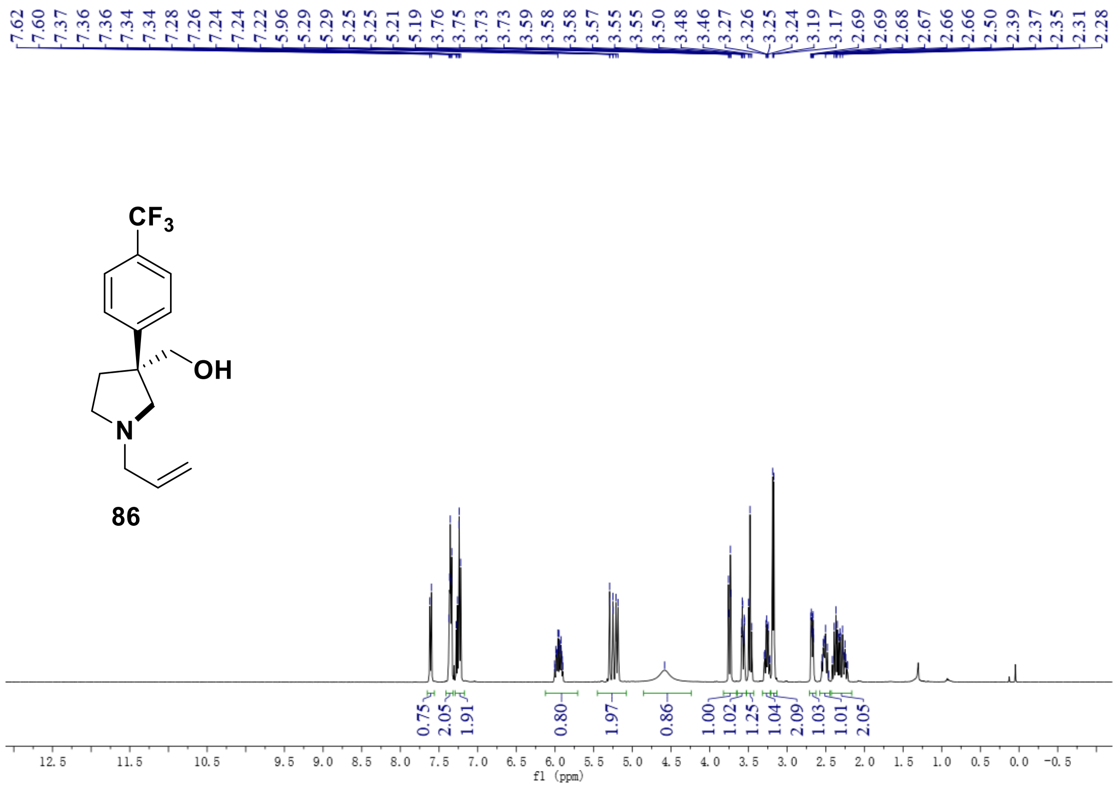

Supplementary Figure 187. ^{1}H NMR (400 MHz, $CDCl_3$) spectrum of **86.**

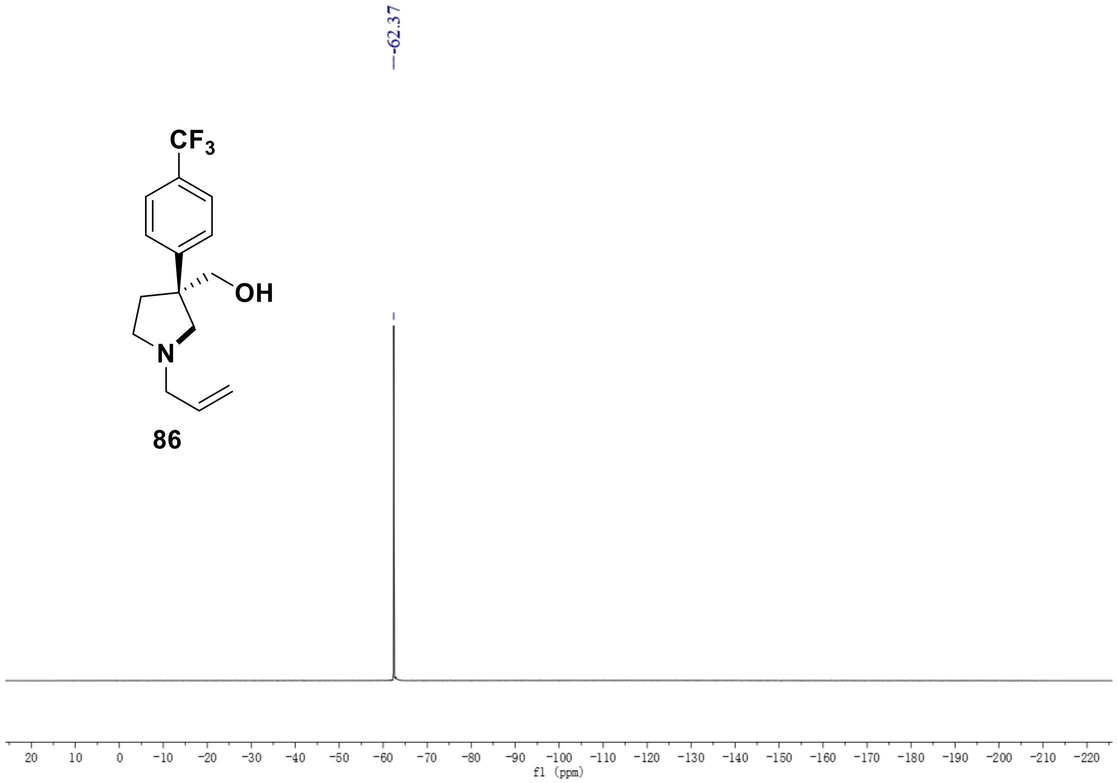

Supplementary Figure 188. ^{19}F NMR (400 MHz, $CDCl_3$) spectrum of **86.**

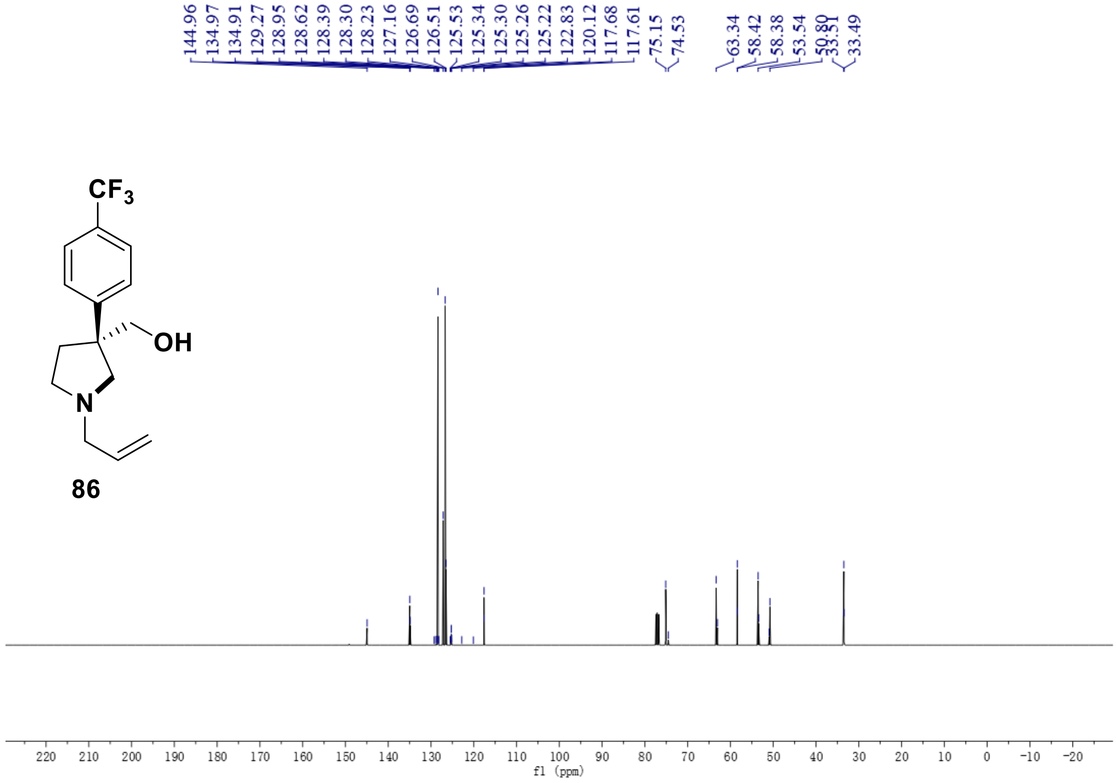

Supplementary Figure 189. ^{13}C NMR (101 MHz, CDCl$_3$) spectrum of **86.**

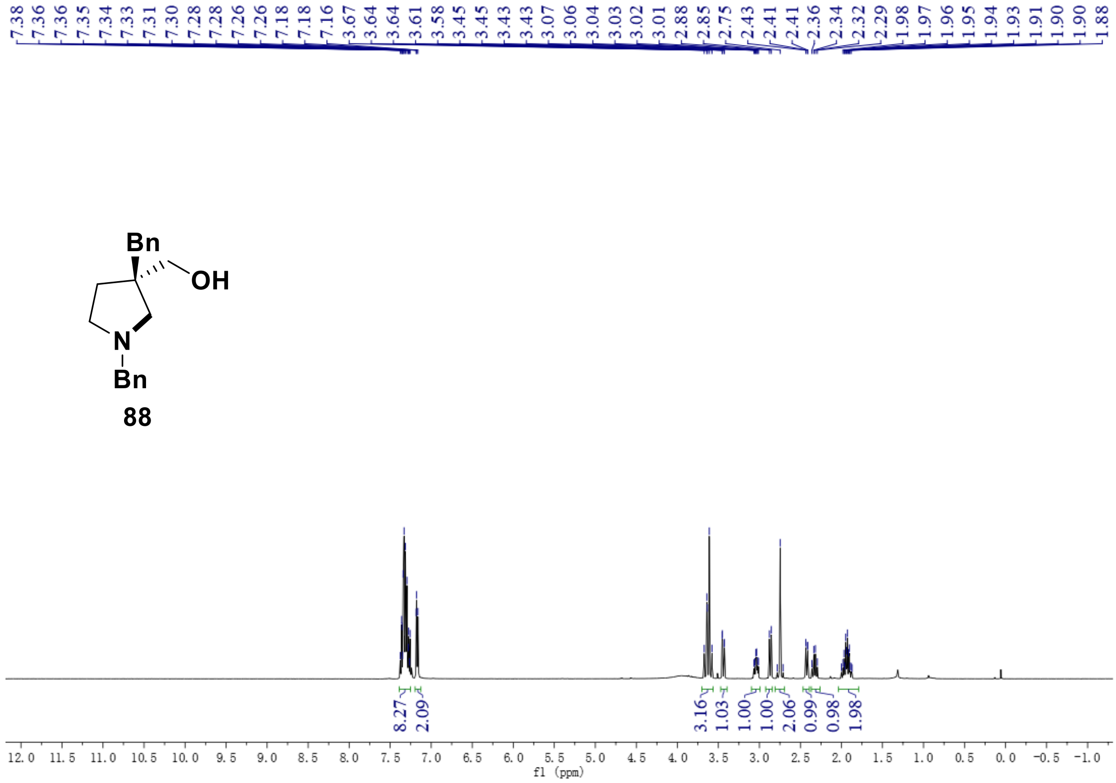

Supplementary Figure 190. ^{1}H NMR (400 MHz, CDCl$_3$) spectrum of **88.**

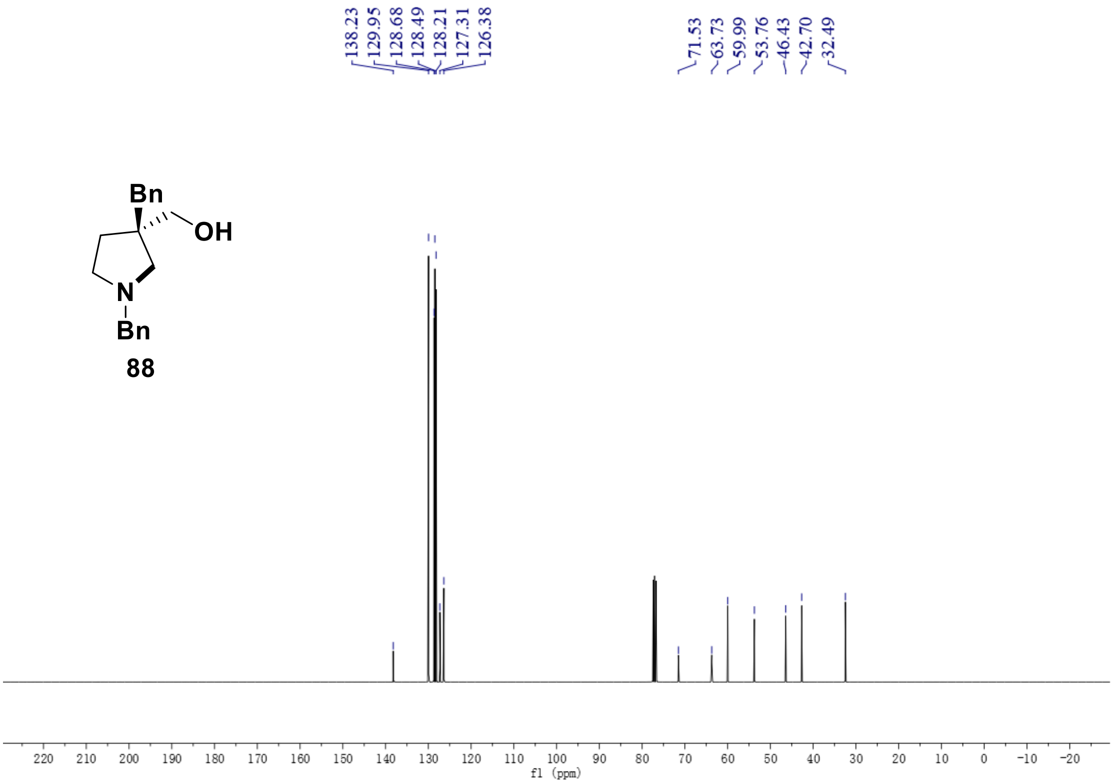

Supplementary Figure 191. ^{13}**C NMR (101 MHz, CDCl$_3$)** spectrum of **88.**

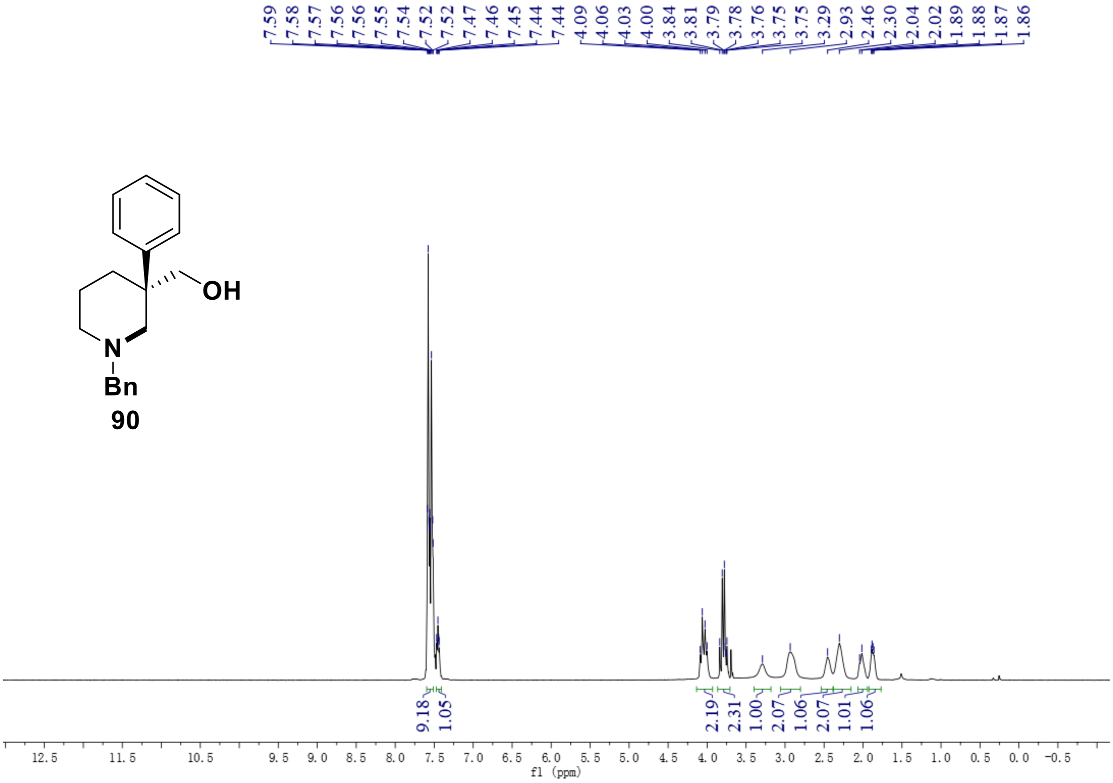

Supplementary Figure 192. ^{1}**H NMR (400 MHz, CDCl$_3$)** spectrum of **90.**

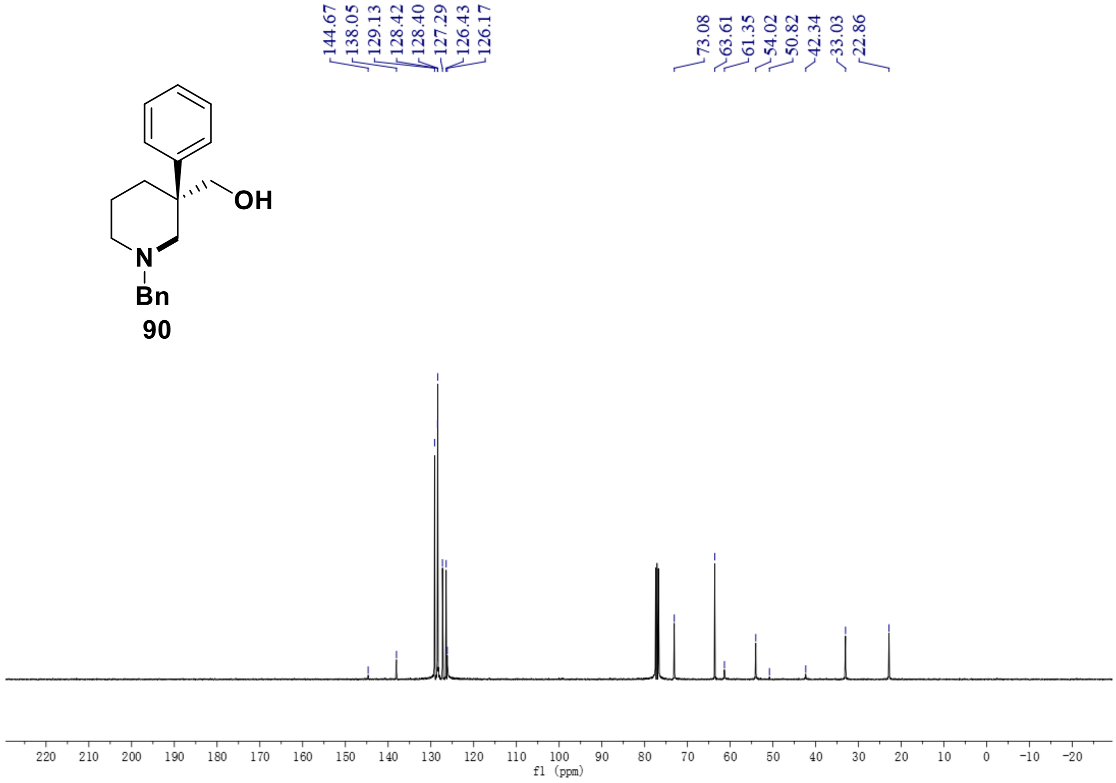

90

Supplementary Figure 193. ^{13}C NMR (101 MHz, $CDCl_3$) spectrum of **90.**

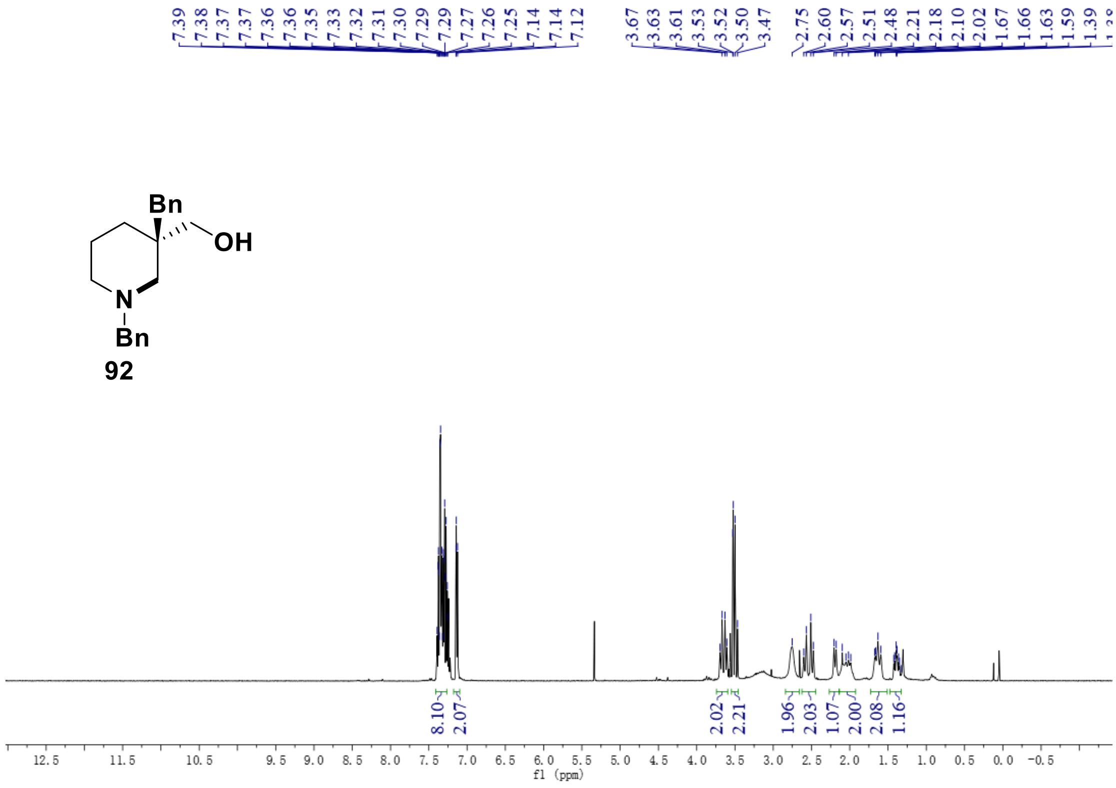

92

Supplementary Figure 194. ^{1}H NMR (400 MHz, $CDCl_3$) spectrum of **92**

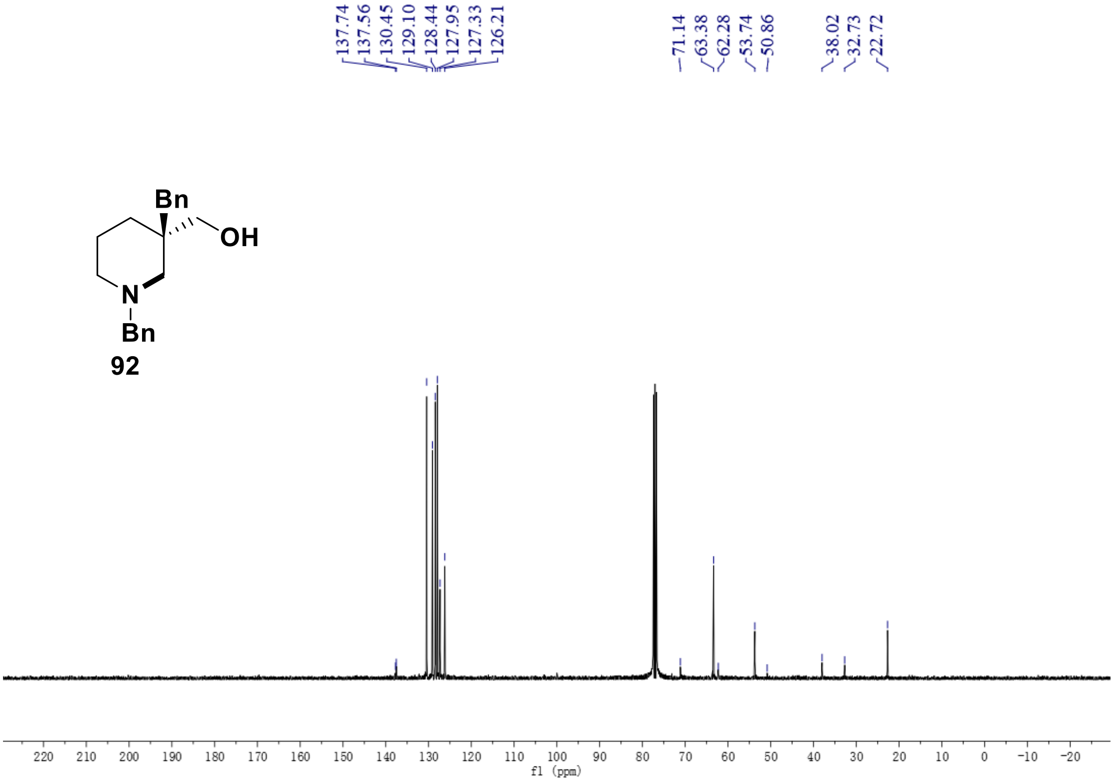

Supplementary Figure 195. ^{13}C NMR (101 MHz, $CDCl_3$) spectrum of **92.**

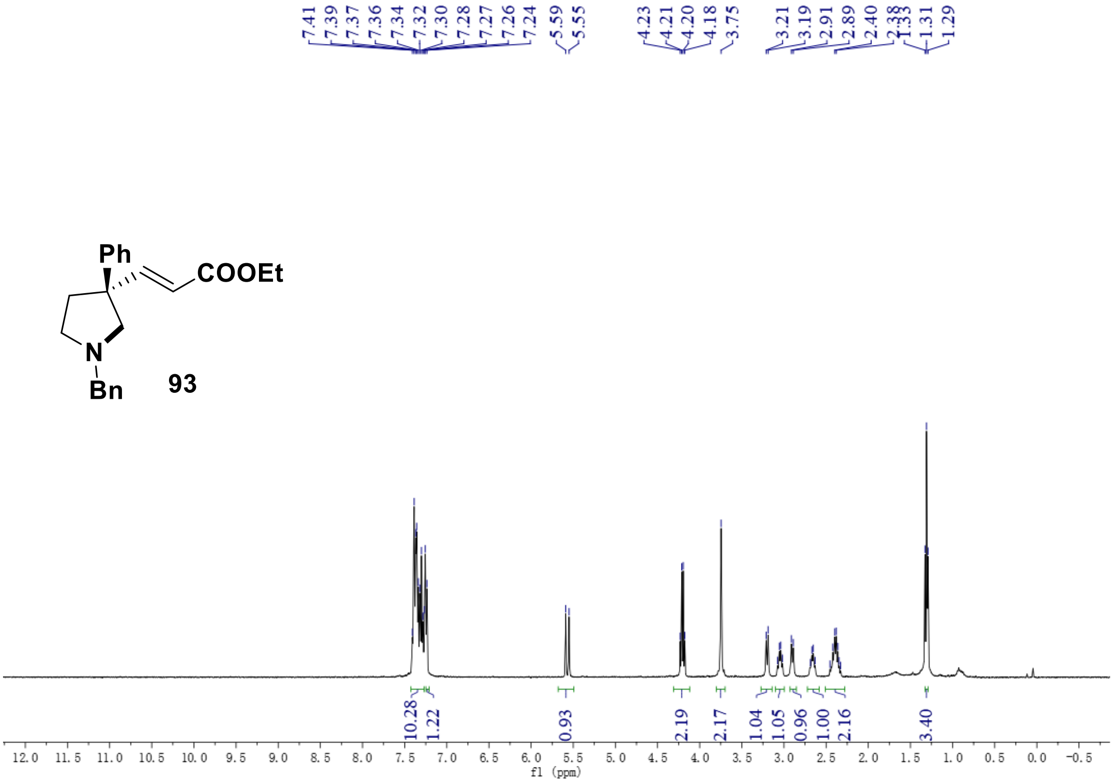

Supplementary Figure 196. ^{1}H NMR (400 MHz, $CDCl_3$) spectrum of **93.**

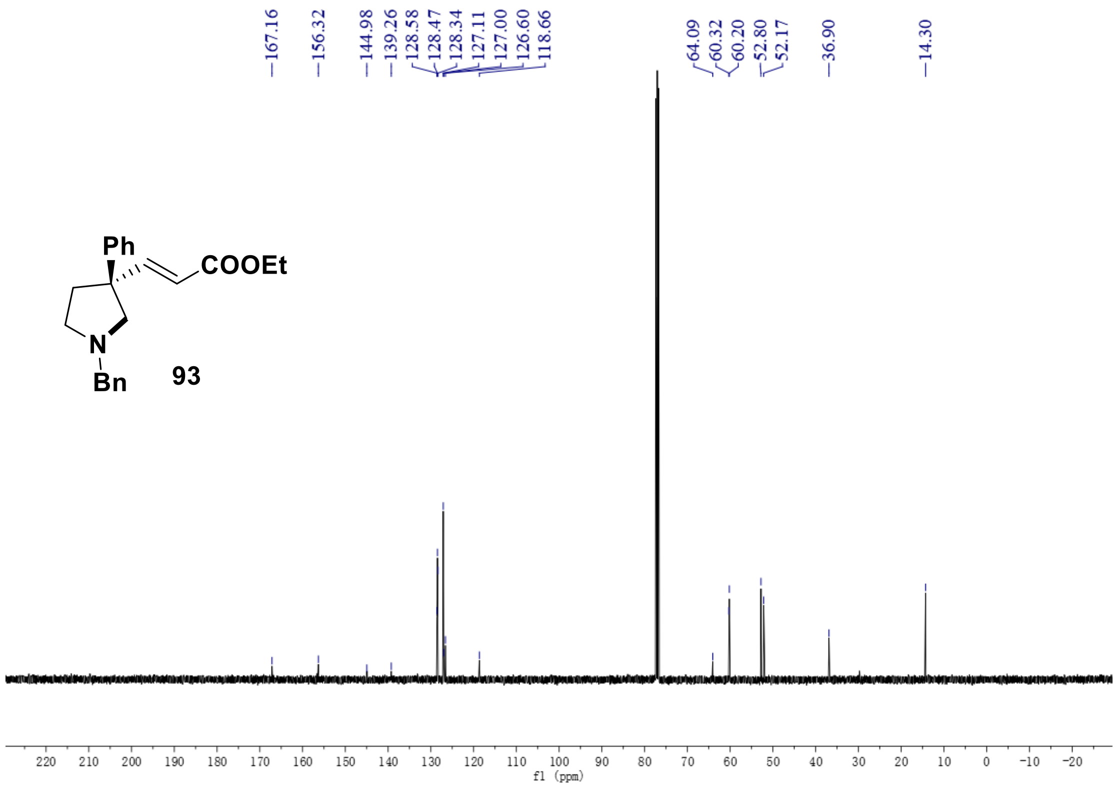

Supplementary Figure 197. ^{13}C NMR (101 MHz, $CDCl_3$) spectrum of **93.**

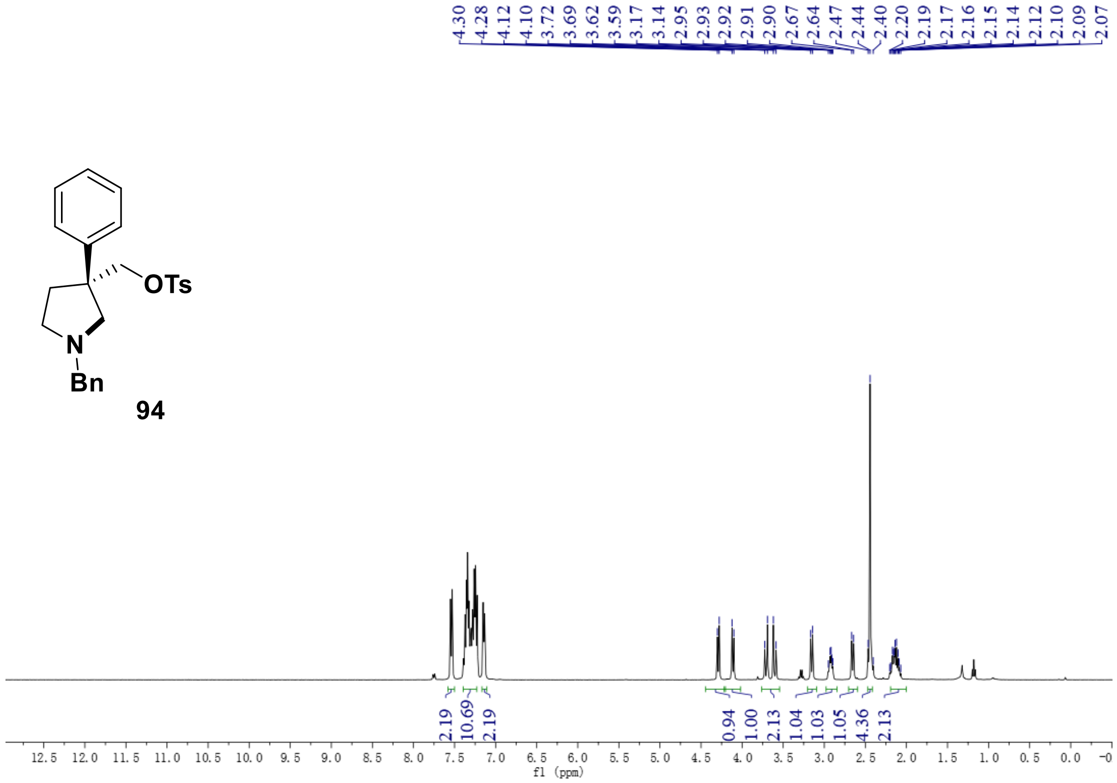

Supplementary Figure 198. ^{1}H NMR (400 MHz, $CDCl_3$) spectrum of **94.**

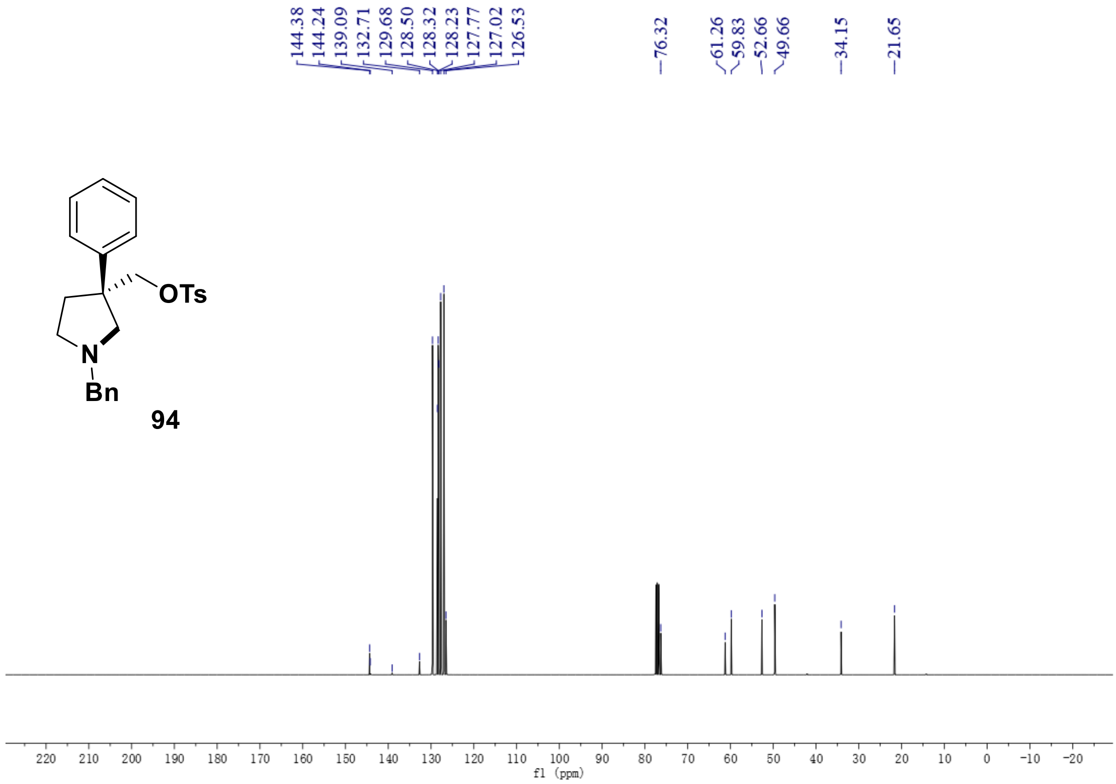

Supplementary Figure 199. ^{13}**C NMR (101 MHz, CDCl$_3$)** spectrum of **94.**

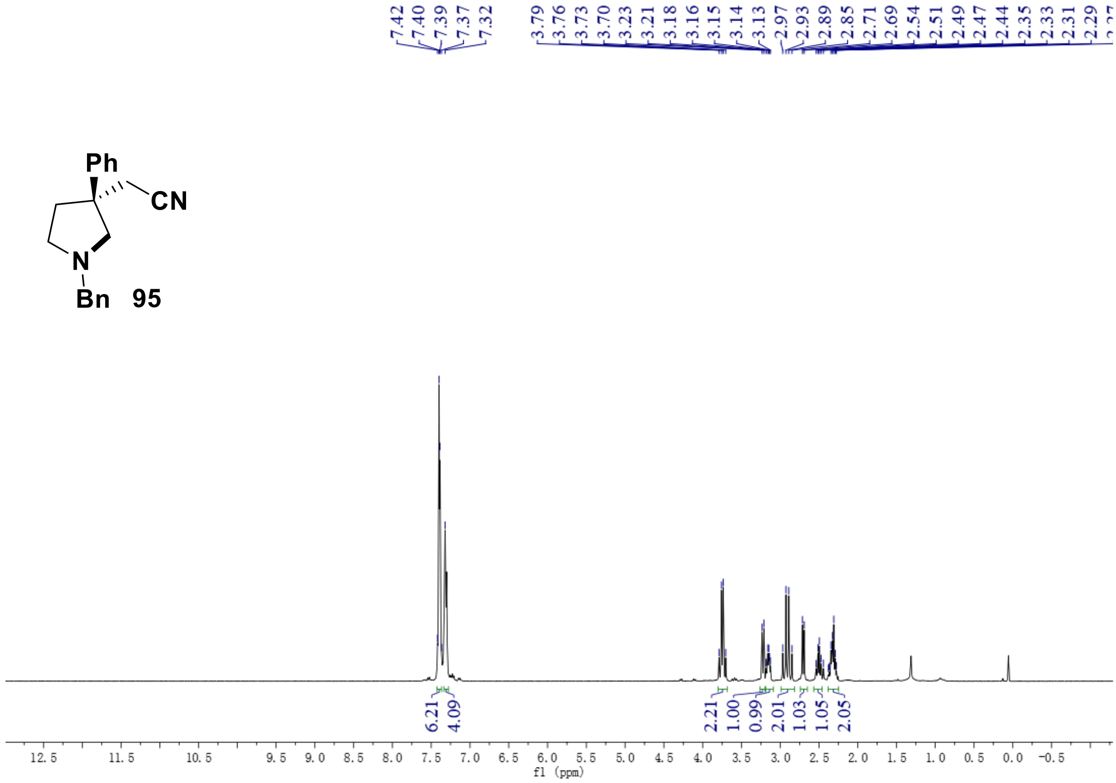

Supplementary Figure 200. ^{1}**H NMR (400 MHz, CDCl$_3$)** spectrum of **95.**

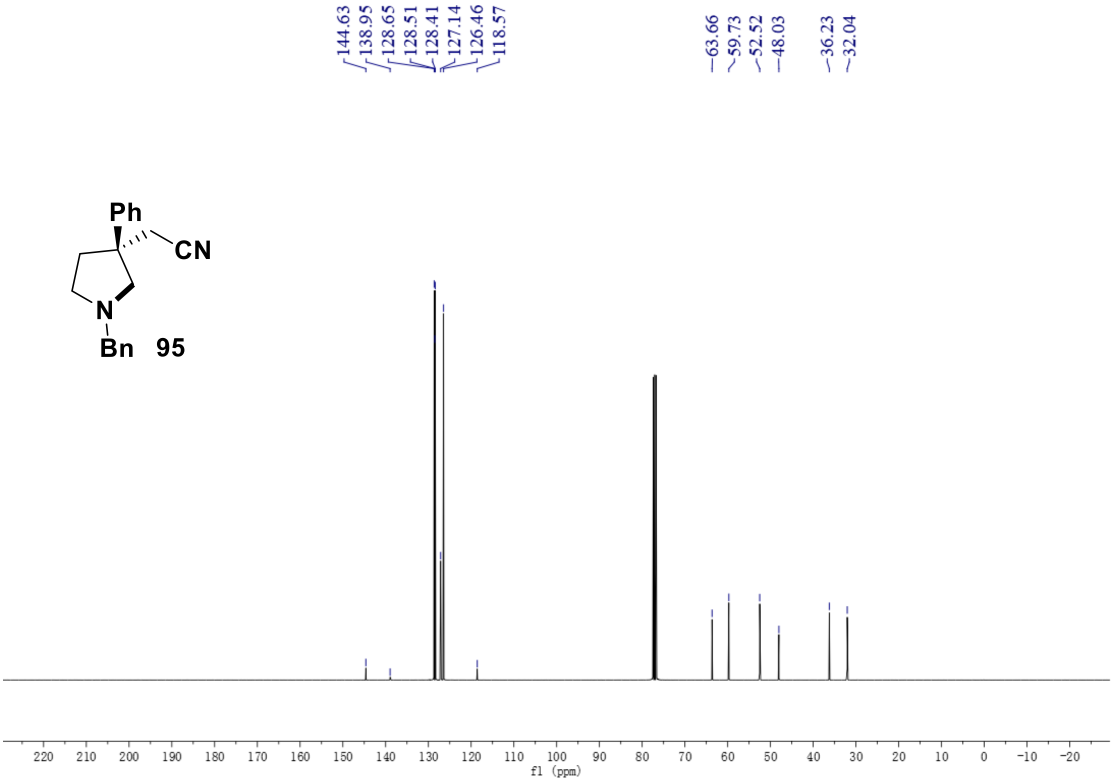

Supplementary Figure 201. **^{13}C NMR (101 MHz, CDCl$_3$)** spectrum of **95.**

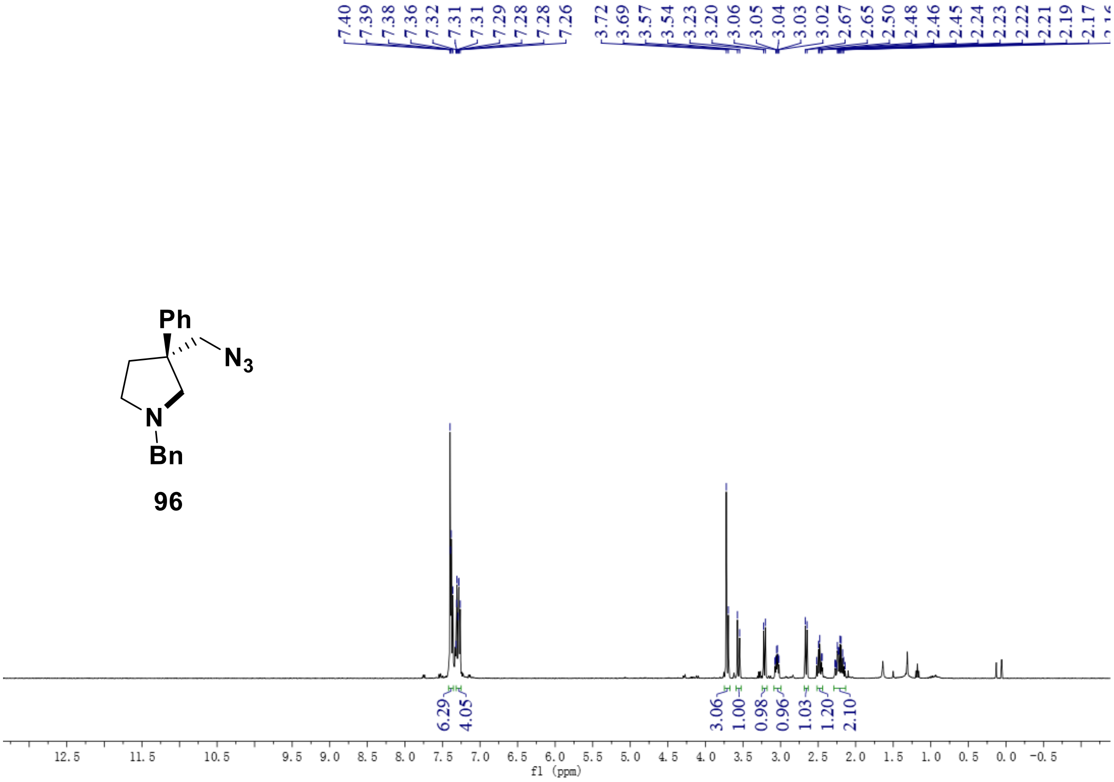

Supplementary Figure 202. **^{1}H NMR (400 MHz, CDCl$_3$)** spectrum of **96.**

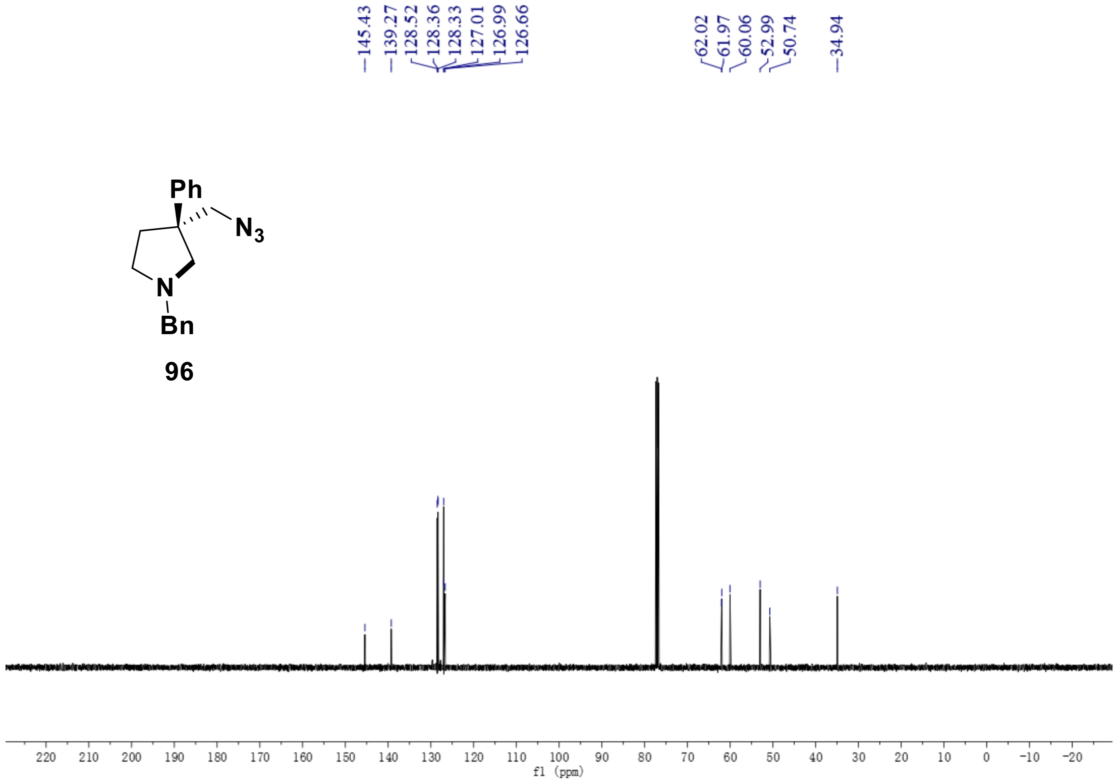

Supplementary Figure 203. **^{13}C NMR (101 MHz, $CDCl_3$)** spectrum of **96.**

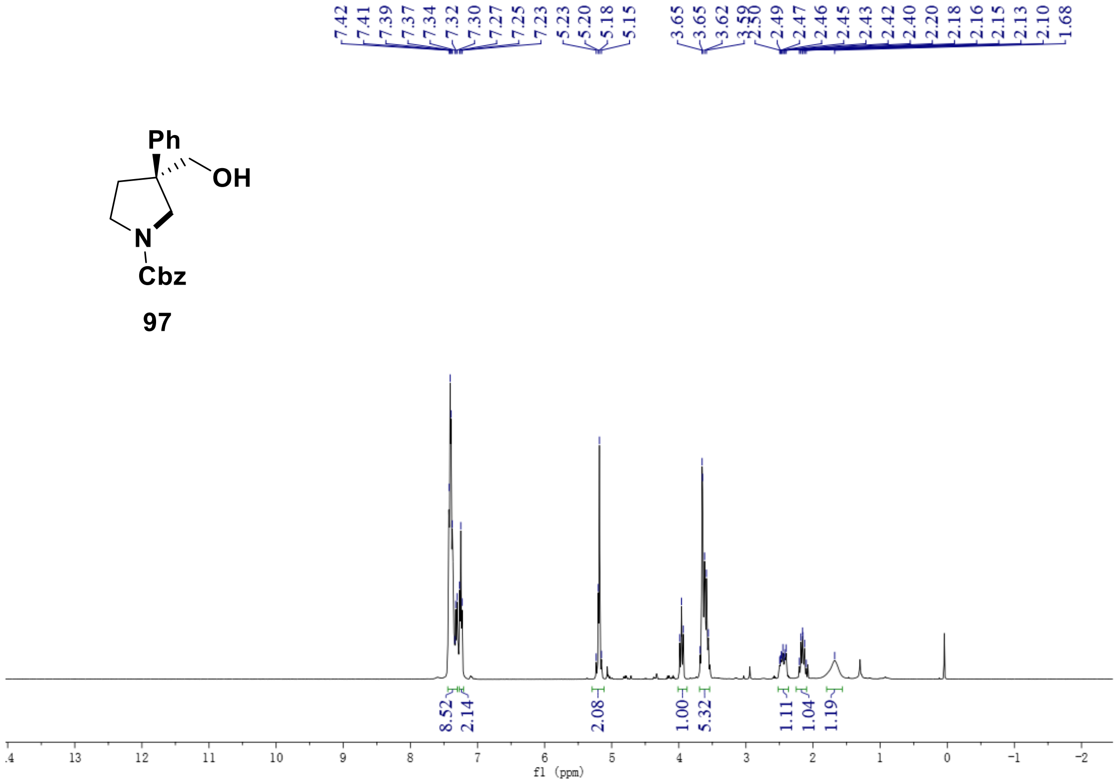

Supplementary Figure 204. **^{1}H NMR (400 MHz, $CDCl_3$)** spectrum of **97.**

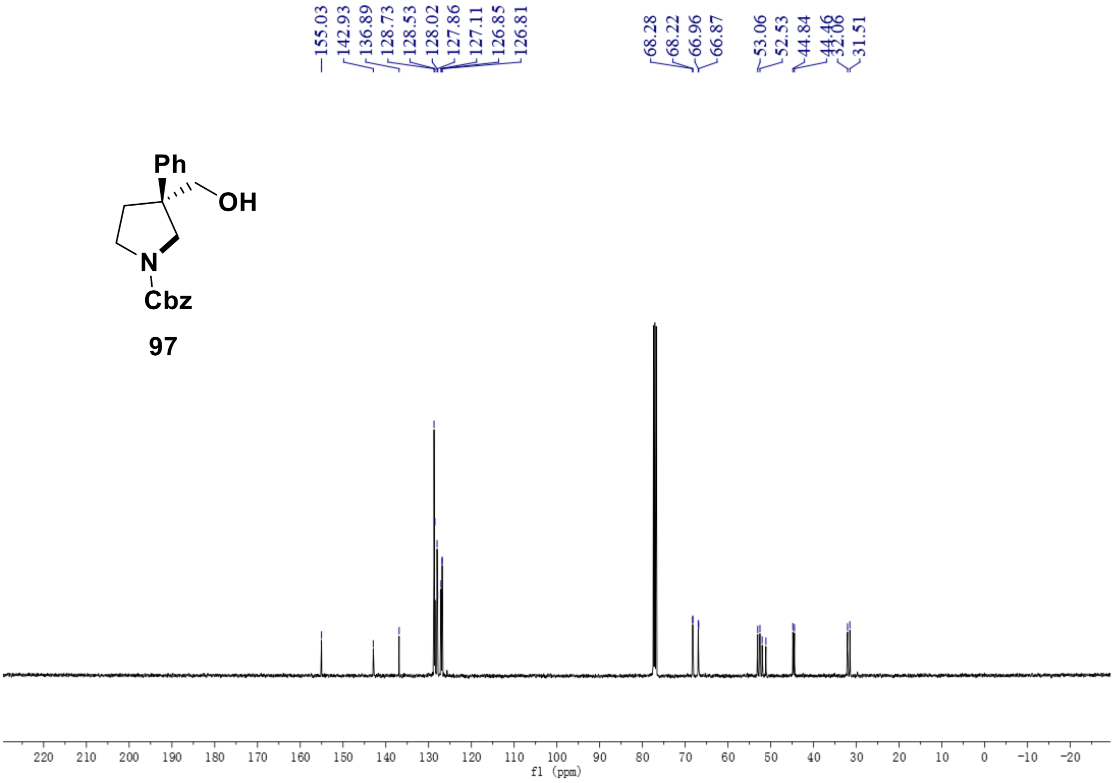

Supplementary Figure 205. ^{13}C NMR (101 MHz, CDCl$_3$) spectrum of **97.**

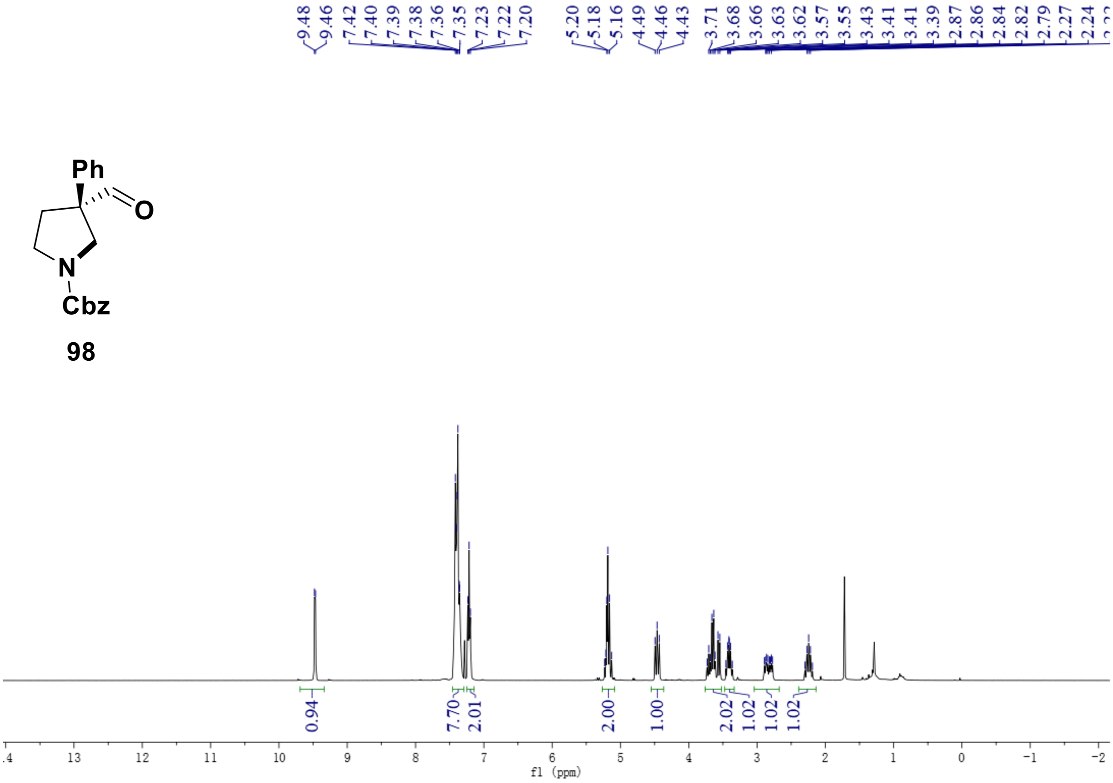

Supplementary Figure 206. ^{1}H NMR (400 MHz, CDCl$_3$) spectrum of **98.**

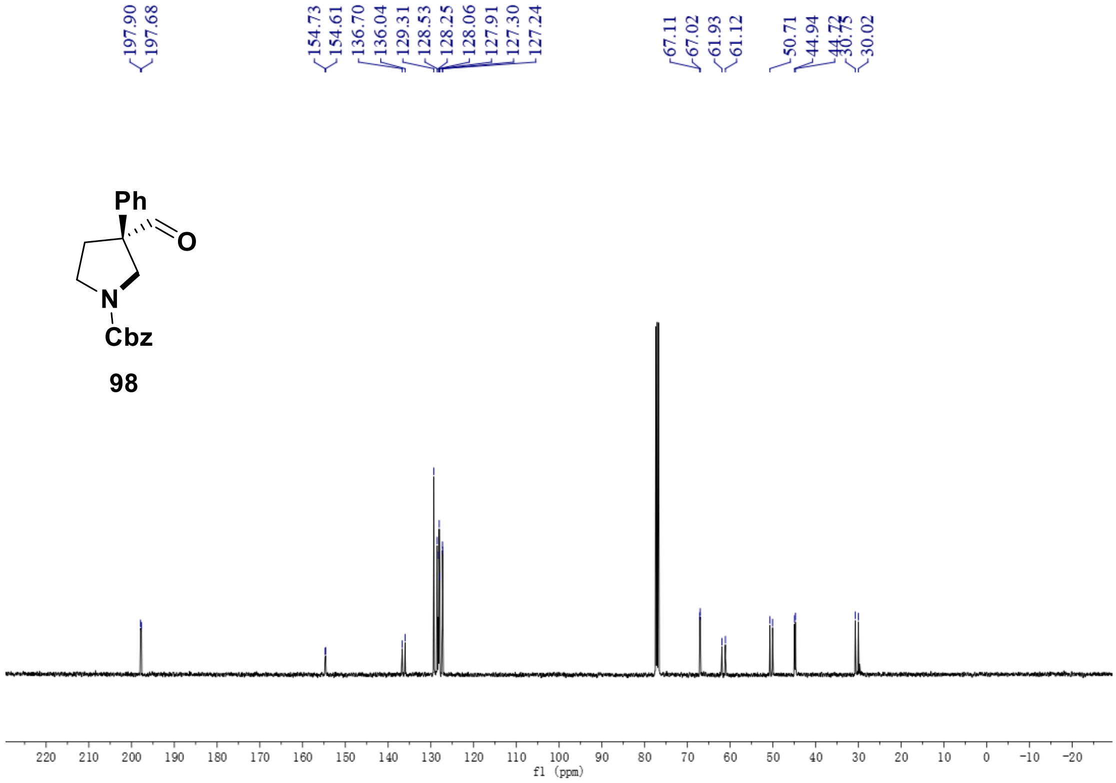

Supplementary Figure 207. ^{13}C NMR (101 MHz, $CDCl_3$) spectrum of **98.**

3.5 HPLC chromatograms

(*rac*)-1-(Tetrahydro-2*H*-pyran-2-yl)ethan-1-ol

Chiral GC, Hydrodex-*β*-TBDAc, 25 m x 0.25 mm ID as the follows condition: 80 °C, 5 °C/min, 110 °C, hold 2 min, 5 °C/min, 135 °C, hold 2 min, 20 °C/min, 220 °C hold 2 min. N_2: 1.5 bar.

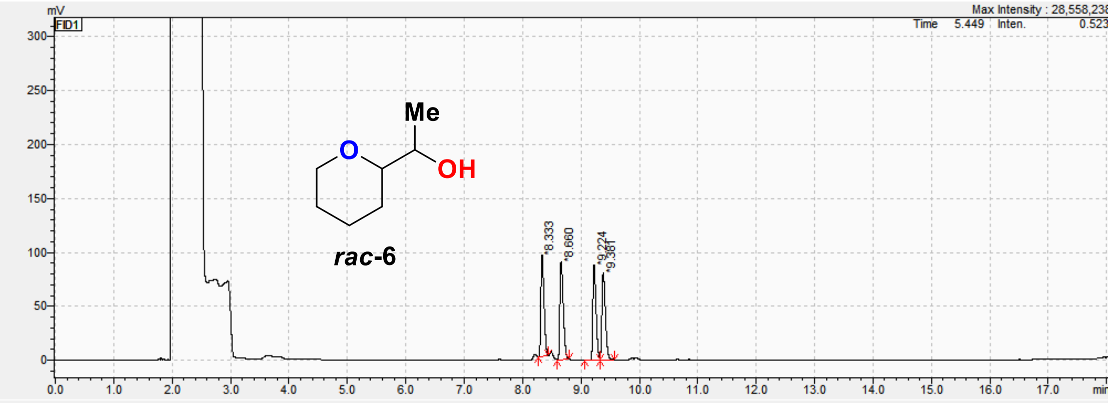

Peak #	Ret. Time [min]	Area mAu*s	Hight mAu	Width [min]	Area %
1	8.333	355073	93937	-	25.623
2	8.660	365049	90268	-	26.343
3	9.224	331140	87643	-	23.896
4	9.381	334472	80033	-	24.137

Supplementary Figure 208. GC profile of ***rac*-6** standard**.**

SZ612: (*S*)-1-(Tetrahydro-2H-pyran-2-yl)ethan-1-ol (6)

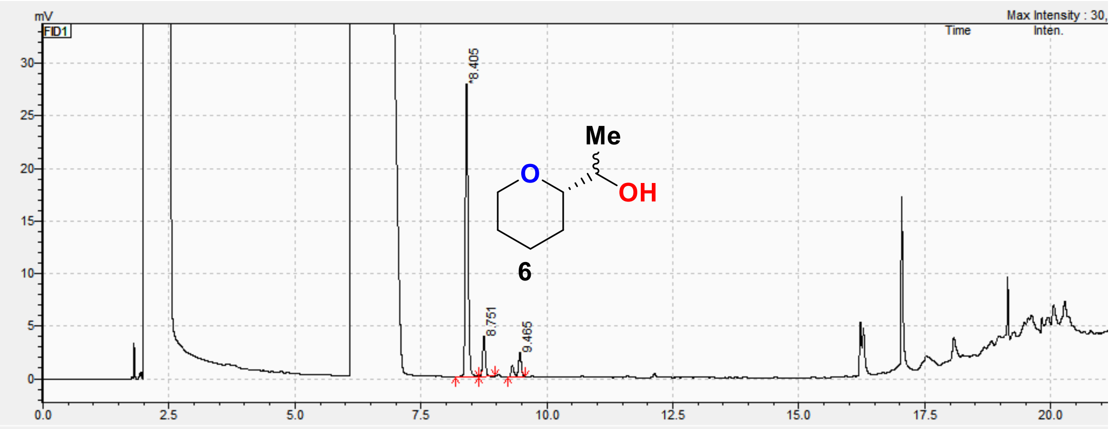

Peak #	Ret. Time [min]	Area mAu*s	Hight mAu	Width [min]	Area %
1	8.405	107551	27753	-	80.134
2	8.751	14007	3766	-	10.436
3	9.465	12656	2317	-	9.430

Supplementary Figure 209. GC profiles of the crude product **(*S*)-6** catalyzed by mutant SZ612**.**

SZ616: 1-(Tetrahydro-2H-pyran-2-yl)ethan-1-ol (6)

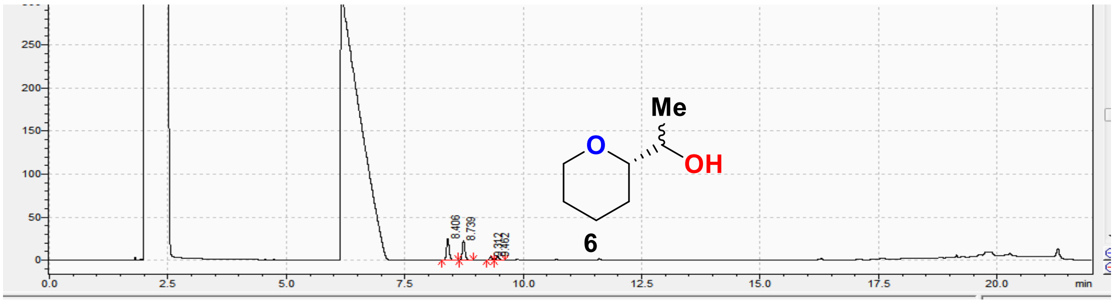

Peak #	Ret. Time [min]	Area mAu*s	Hight mAu	Width [min]	Area %
1	8.406	95050	24601	-	44.621
2	8.739	87941	22879	-	41.284
3	9.312	13594	3644	-	6.382
4	9.462	16430	4290	-	7.713

Supplementary Figure 210. GC profile of the crude product **6** catalyzed by mutant SZ612**.**

(*rac*)-2-(Tetrahydro-2H-pyran-2-yl)propan-2-ol (8)

Chiral GC, LIPODEX-E, 25 m x 0.25 mm ID as the follows condition: 80 °C, hold 1 min. 3 °C/min, 100 °C, hold 2 min, 20 °C/min, 175 °C hold 2 min. N_2: 1.5 bar.

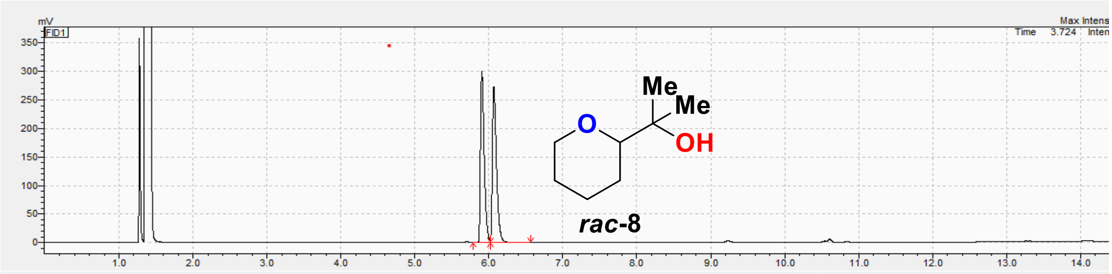

Peak #	Ret. Time [min]	Area mAu*s	Hight mAu	Width [min]	Area %
1	5.905	1057409	299335	-	49.872
2	6.068	1062844	271330	-	50.128

Supplementary Figure 211. GC profile of ***rac*-8** standard**.**

(*S*)-2-(Tetrahydro-2H-pyran-2-yl)propan-2-ol (8)

SZ532: Chiral GC, LIPODEX-E, 25 m x 0.25 mm ID as the follows condition: 80 °C, hold 1 min. 3 °C/min, 100 °C, hold 2 min, 20 °C/min, 175 °C hold 2 min. N_2: 1.5 bar.

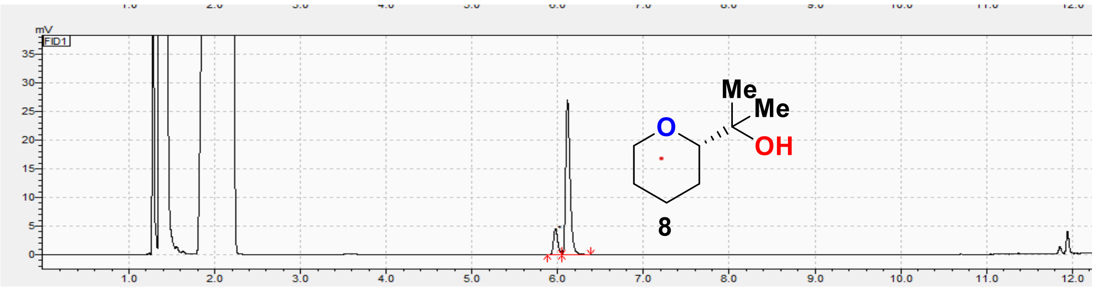

Peak #	Ret. Time [min]	Area mAu*s	Hight mAu	Width [min]	Area %
1	5.973	15441	4525	-	13.843
2	6.114	96108	26828	-	86.157

Supplementary Figure 212. GC profile of the crude product **(*S*)-8** catalyzed by mutant SZ532**.**

SZ612: Chiral GC, LIPODEX-E, 25 m x 0.25 mm ID as the follows condition: 80 °C, hold 1 min. 3 °C/min, 100 °C, hold 2 min, 20 °C/min, 175 °C hold 2 min. N_2: 1.5 bar.

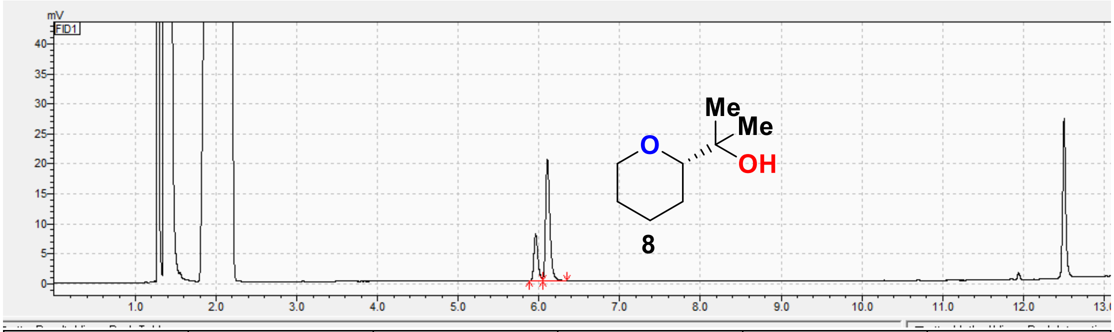

Peak #	Ret. Time [min]	Area mAu*s	Hight mAu	Width [min]	Area %
1	5.958	27024	7872	-	26.511
2	6.104	74912	20137	-	73.489

Supplementary Figure 213. GC profile of the crude product **(*S*)-8** catalyzed by mutant SZ612**.**

SZ616: Chiral GC, LIPODEX-E, 25 m x 0.25 mm ID as the follows condition: 80 °C, hold 1 min. 3 °C/min, 100 °C, hold 2 min, 20 °C/min, 175 °C hold 2 min. N_2: 1.5 bar.

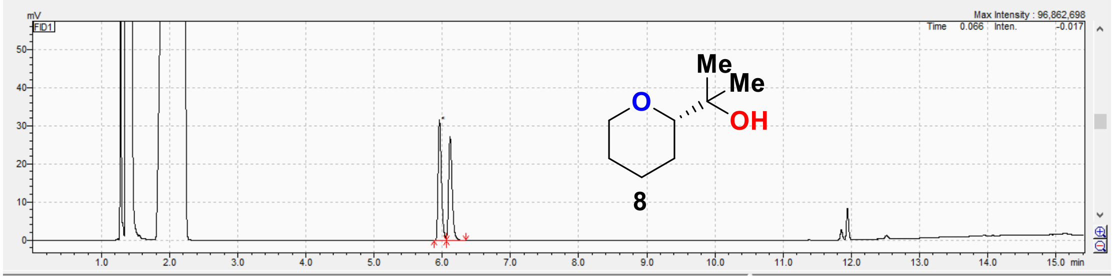

Peak #	Ret. Time [min]	Area mAu*s	Hight mAu	Width [min]	Area %
1	5.958	109386	31775	-	52.426
2	6.114	99262	27224	-	47.574

Supplementary Figure 214. GC profile of the crude product **(*S*)-8** catalyzed by mutant SZ616**.**

(*rac*)-(3-Phenyltetrahydrofuran-3-yl)methanol (10)

HPLC (ChiralPak AD-H, hexane : isopropanol=97 : 3, 1 mL/min, 220 nm

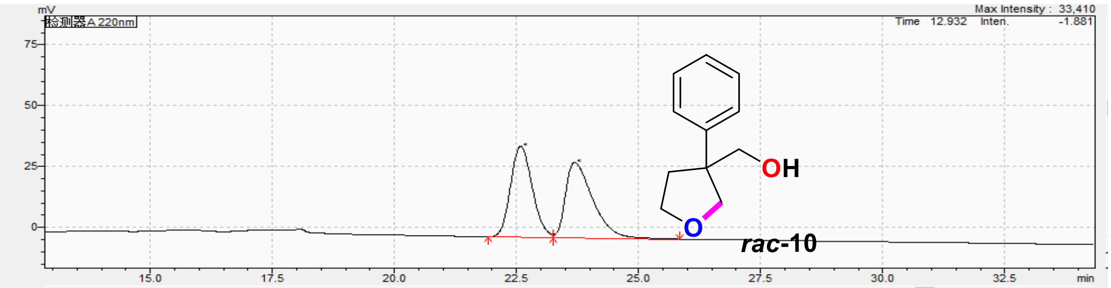

Peak #	Ret. Time [min]	Area mAu*s	Hight mAu	Width [min]	Area %
1	22.585	1154511	37349	0.483	49.129
2	24.693	1195457	30939	0.584	50.871

Supplementary Figure 215. HPLC profile of ***rac*-10** standard**.**

(*S*)-(3-Phenyltetrahydrofuran-3-yl)methanol (10)

SZ616: HPLC (ChiralPak AD-H, hexane : isopropanol=97 : 3, 1 mL/min, 220 nm

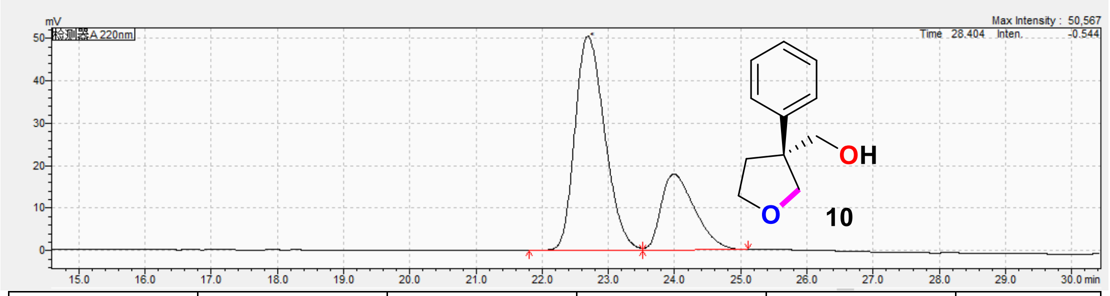

Peak #	Ret. Time [min]	Area mAu*s	Hight mAu	Width [min]	Area %
1	22.758	1599032	49637	0.502	70.948
2	24.054	654769	17701	0.571	29.052

Supplementary Figure 216. HPLC profile of the crude product **(*S*)-10** catalyzed by mutant SZ616**.**

SZ621: HPLC (ChiralPak AD-H, hexane : isopropanol=97 : 3, 1 mL/min, 220 nm)

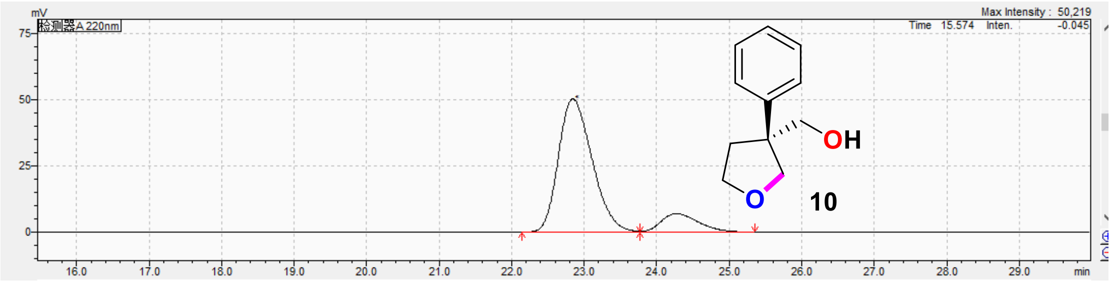

Peak #	Ret. Time [min]	Area mAu*s	Hight mAu	Width [min]	Area %
1	22.844	1622039	50180	0.504	86.752
2	24.273	247708	6902	0.559	13.248

Supplementary Figure 217. HPLC profile of the crude product **(*S*)-10** catalyzed by mutant SZ621**.**

(*rac*)-(3-(4-Tolyl)tetrahydrofuran-3-yl)methanol (12)

HPLC (ChiralPak AD-H, hexane : isopropanol=97 : 3, 1 mL/min, 220 nm)

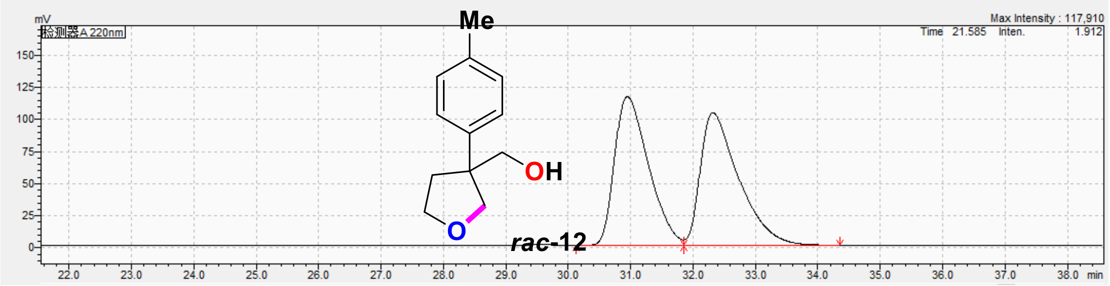

Peak #	Ret. Time [min]	Area mAu*s	Hight mAu	Width [min]	Area %
1	30.946	4393662	116078	0.596	49.827
2	32.319	4424138	103568	0.643	50.173

Supplementary Figure 218. HPLC profile of ***rac*-12** standard.

(*S*)-(3-(4-Tolyl)tetrahydrofuran-3-yl)methanol (12)

SZ616: HPLC (ChiralPak AD-H, hexane : isopropanol=97 : 3, 1 mL/min, 220 nm)

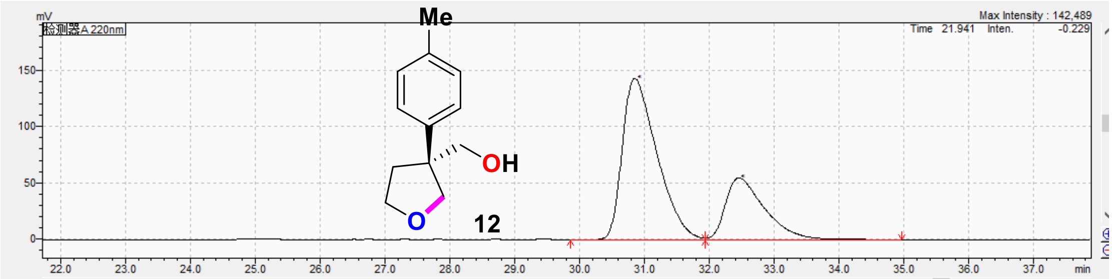

Peak #	Ret. Time [min]	Area mAu*s	Hight mAu	Width [min]	Area %
1	30.852	5302373	142792	0.578	69.951
2	32.458	2277714	54739	0.624	30.049

Supplementary Figure 219. HPLC profile of the crude product **(*S*)-12** catalyzed by mutant SZ616**.**

SZ621: HPLC (ChiralPak AD-H, hexane : isopropanol=97 : 3, 1 mL/min, 220 nm)

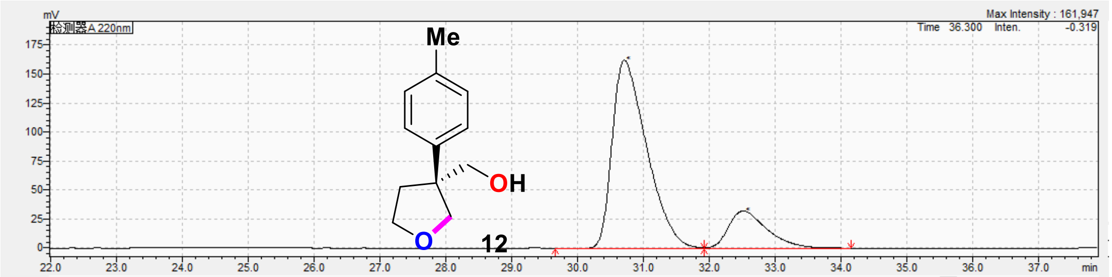

Peak #	Ret. Time [min]	Area mAu*s	Hight mAu	Width [min]	Area %
1	30.706	5918349	162275	0.567	81.846
2	32.513	1312707	32186	0.615	18.154

Supplementary Figure 220. HPLC profile of the crude product **(*S*)-12** catalyzed by mutant SZ621**.**

(*rac*)-(3-(3-Tolyl)tetrahydrofuran-3-yl)methanol (14)

HPLC (ChiralPak AD-H, hexane : isopropanol=95 : 5, 1 mL/min, 220 nm)

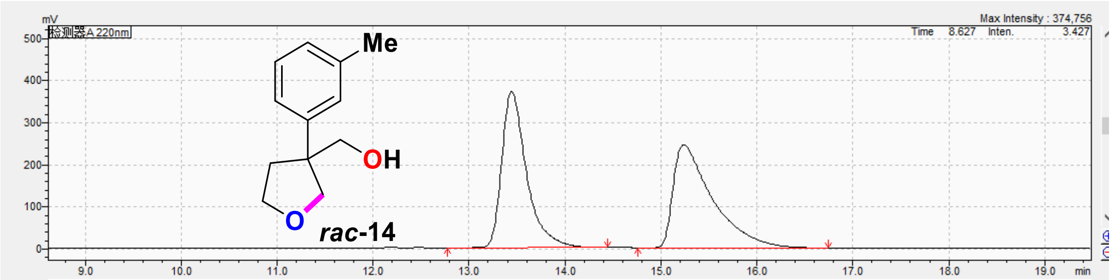

Peak #	Ret. Time [min]	Area mAu*s	Hight mAu	Width [min]	Area %
1	13.445	6717355	371925	0.260	49.161
2	15.238	6946609	244654	0.407	50.839

Supplementary Figure 221. HPLC profile of ***rac*-14** standard.

(*S*)-(3-(*m*-Tolyl)tetrahydrofuran-3-yl)methanol (14)

SZ616: HPLC (ChiralPak AD-H, hexane : isopropanol=95 : 5, 1 mL/min, 220 nm)

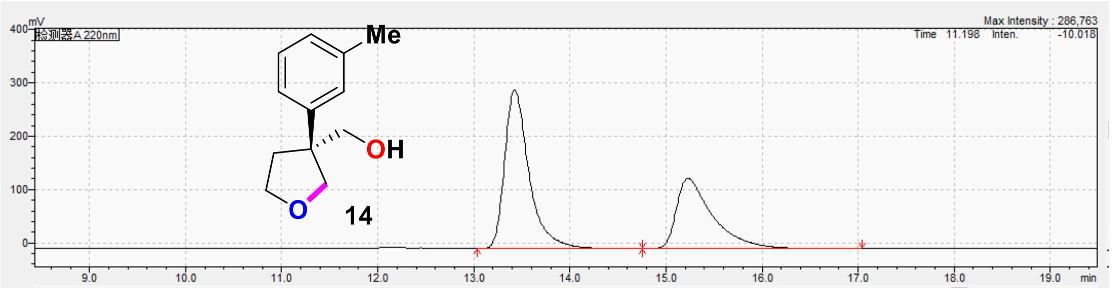

Peak #	Ret. Time [min]	Area mAu*s	Hight mAu	Width [min]	Area %
1	13.425	5245586	297413	0.254	60.408
2	15.230	3437961	131153	0.371	39.592

Supplementary Figure 222. HPLC profile of the crude product **(*S*)-14** catalyzed by mutant SZ616**.**

SZ621: HPLC (ChiralPak AD-H, hexane : isopropanol=95 : 5, 1 mL/min, 220 nm)

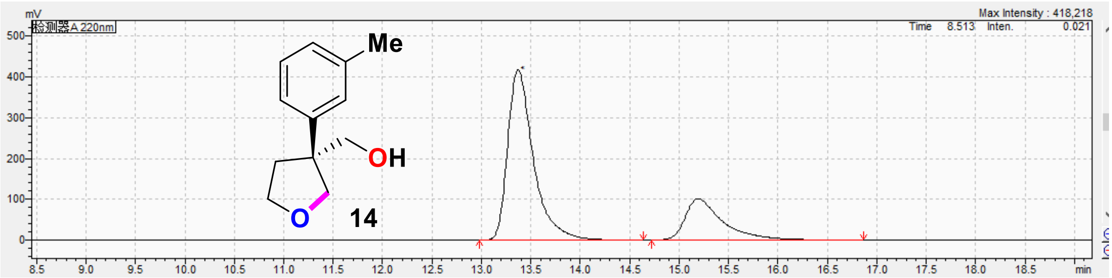

Peak #	Ret. Time [min]	Area mAu*s	Hight mAu	Width [min]	Area %
1	13.370	7590796	418602	0.262	74.024
2	15.193	2663727	101396	0.362	25.976

Supplementary Figure 223. HPLC profile of the crude product **(*S*)-14** catalyzed by mutant SZ621**.**

(*rac*)-(3-(3,5-Dimethylphenyl)tetrahydrofuran-3-yl)methanol (16)

HPLC (ChiralPak AD-H, hexane : isopropanol=98 : 2, 1 mL/min, 220 nm)

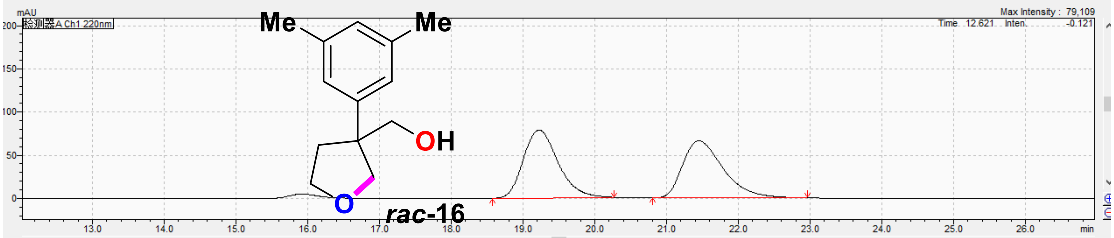

Peak #	Ret. Time [min]	Area mAu*s	Hight mAu	Width [min]	Area %
1	19.222	2635943	78525	0.516	50.240
2	21.450	2610735	66235	0.598	49.760

Supplementary Figure 224. HPLC profile of ***rac*-16** standard.

(*S*)-(3-(3,5-Dimethylphenyl)tetrahydrofuran-3-yl)methanol (16)

SZ616: HPLC (ChiralPak AD-H, hexane : isopropanol=98 : 2, 1 mL/min, 220 nm)

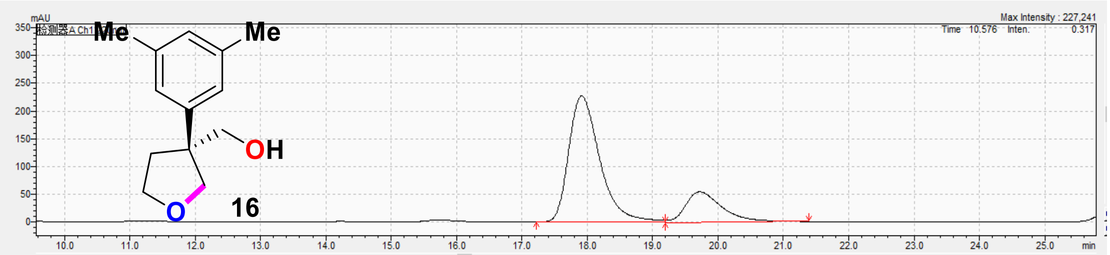

Peak #	Ret. Time [min]	Area mAu*s	Hight mAu	Width [min]	Area %
1	17.914	7444160	227358	0.491	78.053
2	19.720	2093118	54844	0.570	21.947

Supplementary Figure 225. HPLC profile of the crude product **(*S*)-16** catalyzed by mutant SZ616**.**

SZ621: HPLC (ChiralPak AD-H, hexane : isopropanol=98 : 2, 1 mL/min, 220 nm)

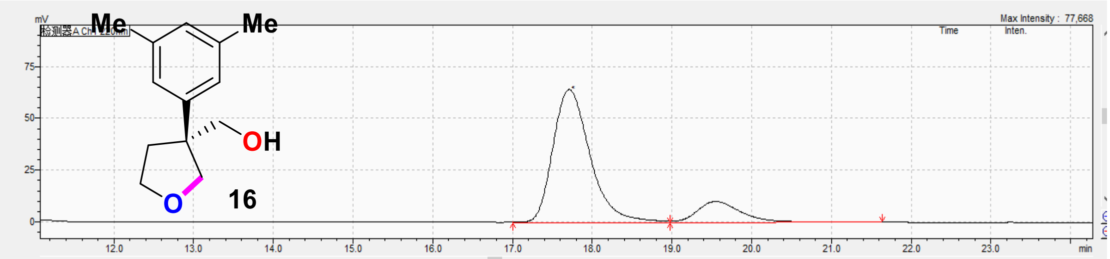

Peak #	Ret. Time [min]	Area mAu*s	Hight mAu	Width [min]	Area %
1	17.711	2076871	64198	0.487	84.301
2	19.550	386768	10066	0.567	15.699

Supplementary Figure 226. HPLC profile of the crude product **(*S*)-16** catalyzed by mutant SZ621**.**

(*rac*)-(3-(4-Ethylphenyl)tetrahydrofuran-3-yl)methyl benzoate

HPLC (ChiralPak IC, hexane : EtOH=97 : 3, 1 mL/min, 220 nm)

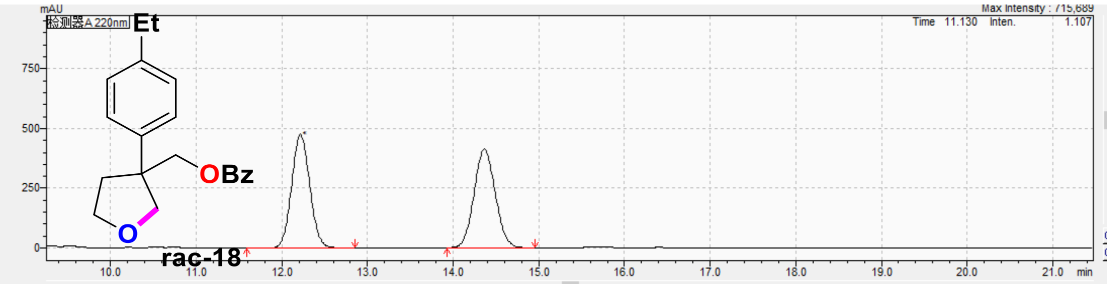

Peak #	Ret. Time [min]	Area mAu*s	Hight mAu	Width [min]	Area %
1	12.217	7029115	475341	0.229	49.472
2	14.365	7179211	413550	0.269	50.528

Supplementary Figure 227. HPLC profile of the derivative of ***rac*-18** standard**.**

(*R*)-(3-(4-Ethylphenyl)tetrahydrofuran-3-yl)methyl benzoate (18)

SZ616: HPLC (ChiralPak IC, hexane : EtOH=97 : 3, 1 mL/min, 220 nm)

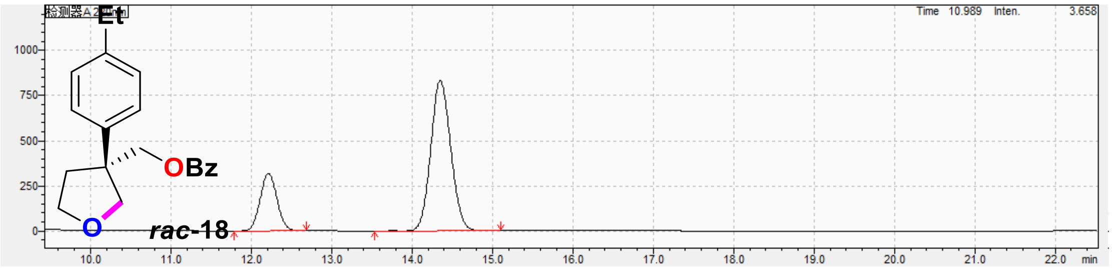

Peak #	Ret. Time [min]	Area mAu*s	Hight mAu	Width [min]	Area %
1	12.214	4640432	318211	0.227	24.337
2	14.353	14427285	833253	0.269	75.663

Supplementary Figure 228. HPLC profile of the derivative of **(*S*)-18** catalyzed by mutant SZ616**.**

SZ621: HPLC (ChiralPak IC, hexane : EtOH=97 : 3, 1 mL/min, 220 nm)

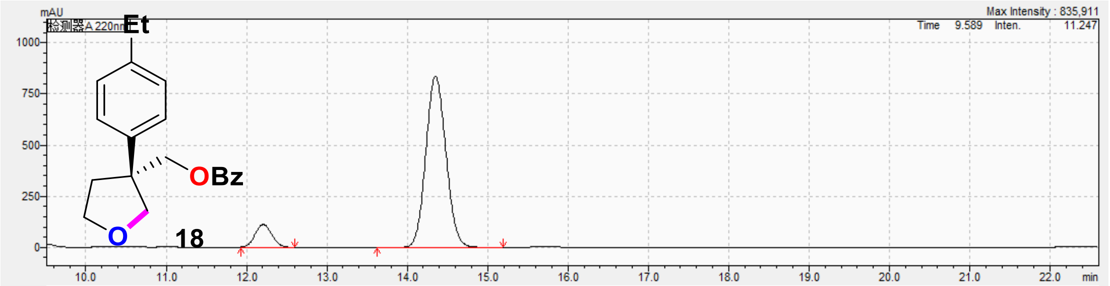

Peak #	Ret. Time [min]	Area mAu*s	Hight mAu	Width [min]	Area %
1	12.208	1604607	110997	0.227	9.971
2	14.351	14488105	835181	0.270	90.029

Supplementary Figure 229. HPLC profile of the the derivative of **(*S*)-18** catalyzed by mutant SZ621**.**

(*rac*)-(3-(4-Methoxyphenyl)tetrahydrofuran-3-yl)methanol

HPLC (ChiralPak AD-H, hexane : isopropanol=98:2, 1 mL/min, 220 nm)

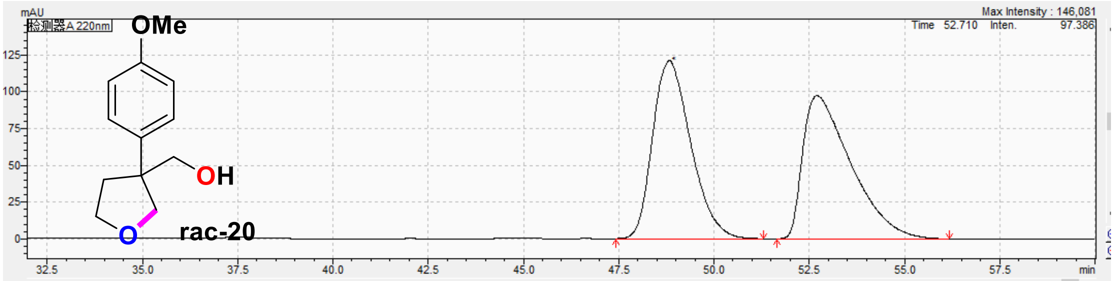

Peak #	Ret. Time [min]	Area mAu*s	Hight mAu	Width [min]	Area %
1	48.831	8433679	120996	1.083	49.679
2	52.708	8542754	97248	1.354	50.321

Supplementary Figure 230. HPLC profile of ***rac*-20** standard**.**

(*S*)-(3-(4-Methoxyphenyl)tetrahydrofuran-3-yl)methanol（20）

SZ616: HPLC (ChiralPak AD-H, hexane : isopropanol=98:2, 1 mL/min, 220 nm)

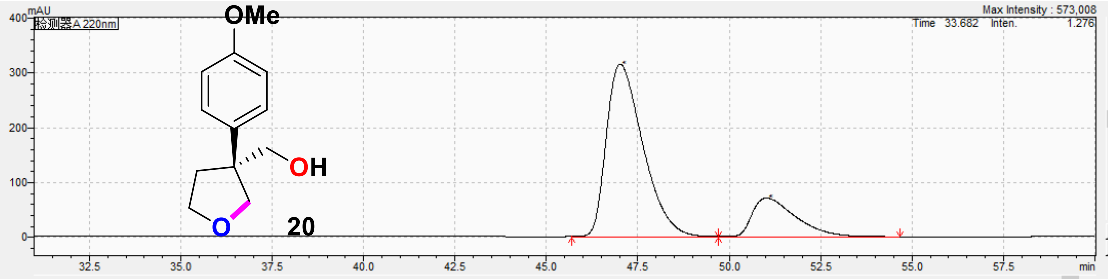

Peak #	Ret. Time [min]	Area mAu*s	Hight mAu	Width [min]	Area %
1	47.019	21913886	315295	1.076	79.054
2	51.013	5806383	70685	1.258	20.946

Supplementary Figure 231. HPLC profile of the crude product **(*S*)-20** catalyzed by mutant SZ616**.**

SZ621:HPLC (ChiralPak AD-H, hexane : isopropanol=98:2, 1 mL/min, 220 nm)

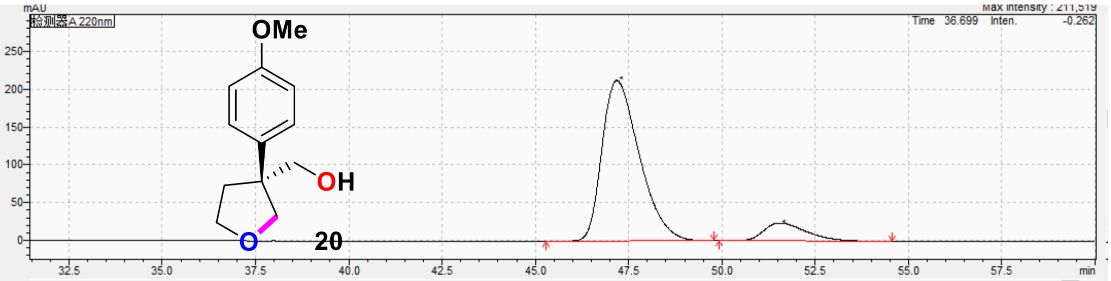

Peak #	Ret. Time [min]	Area mAu*s	Hight mAu	Width [min]	Area %
1	47.181	14796979	212176	1.082	88.648
2	51.563	1894875	23828	1.220	11.352

Supplementary Figure 232. HPLC profile of the crude product **(*S*)-20** catalyzed by mutant SZ621**.**

(*rac*)-(3-(4-(Methylthio)phenyl)tetrahydrofuran-3-yl)methanol

HPLC (ChiralPak AD-H, hexane : EtOH =97:3, 1 mL/min, 220 nm)

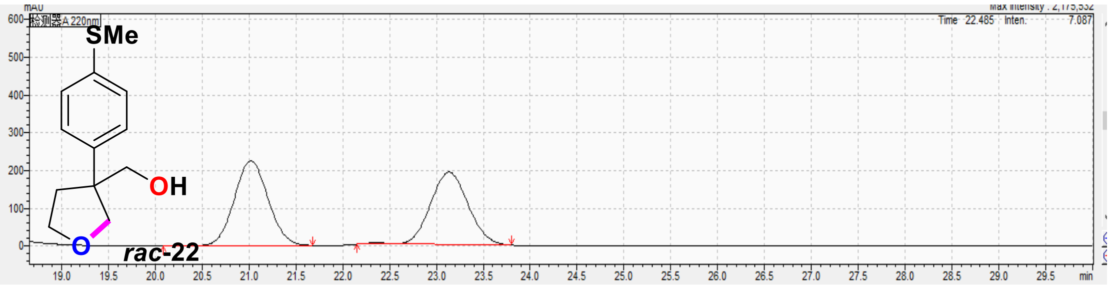

Peak #	Ret. Time [min]	Area mAu*s	Hight mAu	Width [min]	Area %
1	21.020	5630795	224649	0.391	50.840
2	23.134	5444700	192436	0.437	49.160

Supplementary Figure 233. HPLC profile of ***rac*-22** standard.

(*S*)-(3-(4-(Methylthio)phenyl)tetrahydrofuran-3-yl)methanol (22)

SZ616: HPLC (ChiralPak AD-H, hexane : EtOH =97:3, 1 mL/min, 220 nm)

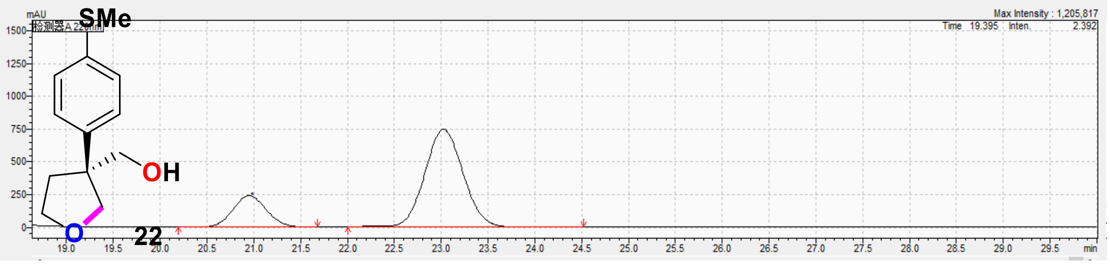

Peak #	Ret. Time [min]	Area mAu*s	Hight mAu	Width [min]	Area %
1	20.949	6003442	240920	0.387	22.041
2	23.025	21234126	746848	0.440	77.959

Supplementary Figure 234. HPLC profile of the crude product **(*S*)-22** catalyzed by mutant SZ616**.**

SZ621: HPLC (ChiralPak AD-H, hexane : EtOH =97:3, 1 mL/min, 220 nm)

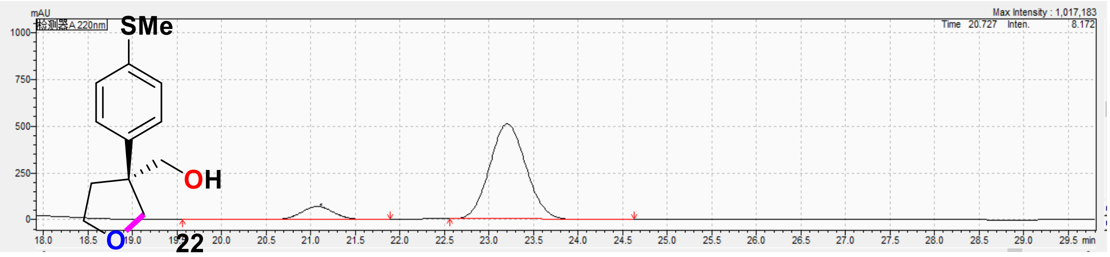

Peak #	Ret. Time [min]	Area mAu*s	Hight mAu	Width [min]	Area %
1	21.073	1832425	71042	0.398	11.297
2	23.207	14387744	508845	0.444	88.703

Supplementary Figure 235. HPLC profile of the crude product **(*S*)-22** catalyzed by mutant SZ621**.**

(*rac*)-(3-(Naphthalen-2-yl)tetrahydrofuran-3-yl)methanol

HPLC (ChiralPak IC, hexane : EtOH =97:3, 1 mL/min, 220 nm)

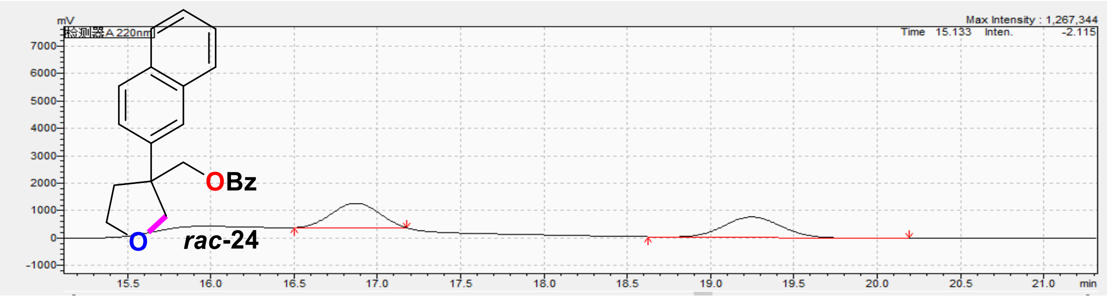

Peak #	Ret. Time [min]	Area mAu*s	Hight mAu	Width [min]	Area %
1	16.870	17535610	891971	0.321	50.569
2	19.246	17141091	740603	0.370	49.431

Supplementary Figure 236. HPLC profile of the derivative of ***rac*-24** standard.

(*R*)-(3-(Naphthalen-2-yl)tetrahydrofuran-3-yl)methanol (24)

SZ616:HPLC (ChiralPak IC, hexane : EtOH =97:3, 1 mL/min, 220 nm)

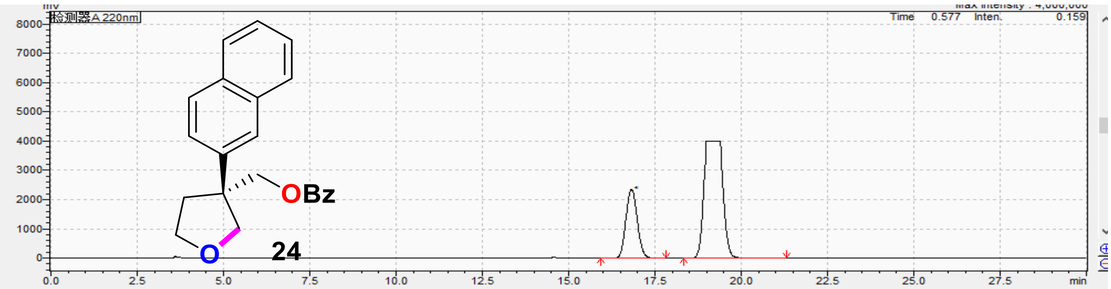

Peak #	Ret. Time [min]	Area mAu*s	Hight mAu	Width [min]	Area %
1	16.818	53984734	2351236	0.361	26.318
2	18.988	151141845	4000505	0.598	73.682

Supplementary Figure 237. HPLC profile of the derivative **(*S*)-24** catalyzed by mutant SZ616.

SZ621:HPLC (ChiralPak IC, hexane : EtOH =97:3, 1 mL/min, 220 nm)

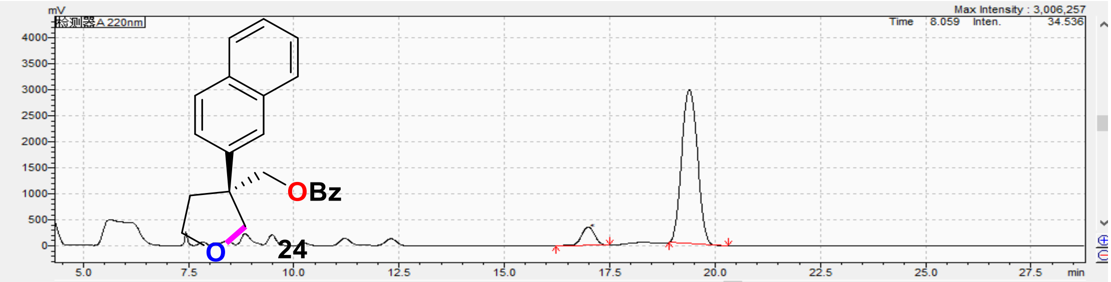

Peak #	Ret. Time [min]	Area mAu*s	Hight mAu	Width [min]	Area %
1	16.987	7778691	357967	0.336	9.175
2	19.391	77005777	2961119	0.419	90.825

Supplementary Figure 238. HPLC profile of the derivative **(*S*)-24** catalyzed by mutant SZ621.

(*rac*)-(3-(4-Fluorophenyl)tetrahydrofuran-3-yl)methanol

HPLC (ChiralPak IC, hexane: isopropanol =93:7, 1 mL/min, 220nm)

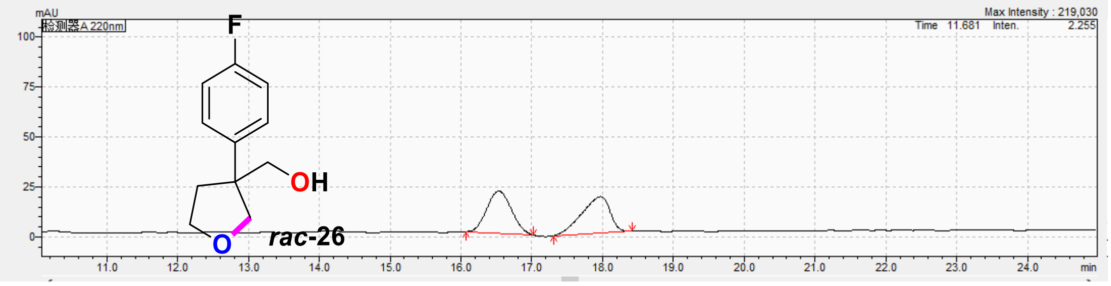

Peak #	Ret. Time [min]	Area mAu*s	Hight mAu	Width [min]	Area %
1	16.535	510210	21047	0.387	50.052
2	17.970	509141	18248	0.446	49.948

Supplementary Figure 239. HPLC profile of ***rac*-26** standard.

(*S*)-(3-(4-Fluorophenyl)tetrahydrofuran-3-yl)methanol (26)

SZ616: HPLC (ChiralPak IC, hexane: isopropanol =93:7, 1 mL/min, 220nm)

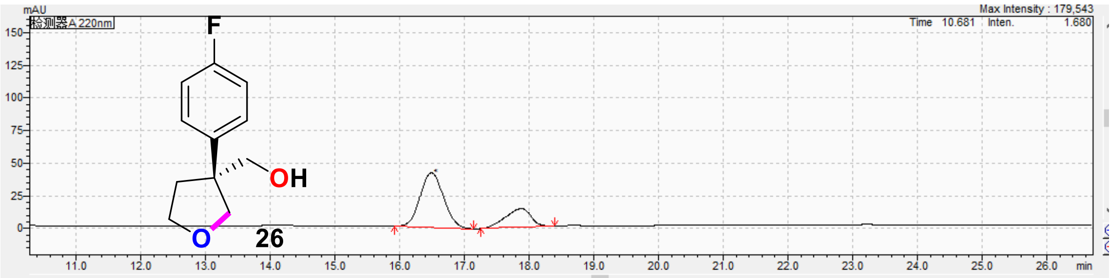

Peak #	Ret. Time [min]	Area mAu*s	Hight mAu	Width [min]	Area %
1	16.491	1047431	41545	0.396	72.877
2	17.881	389824	14214	0.436	27.123

Supplementary Figure 240. HPLC profile of the crude product **(*S*)-26** catalyzed by mutant SZ616**.**

SZ621: HPLC (ChiralPak IC, hexane: isopropanol =93:7, 1 mL/min, 220nm)

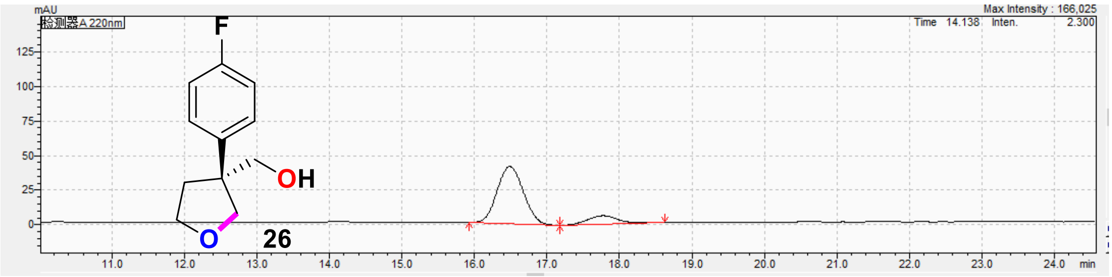

Peak #	Ret. Time [min]	Area mAu*s	Hight mAu	Width [min]	Area %
1	16.486	1042215	41376	0.398	85.870
2	17.774	171494	5990	0.437	14.130

Supplementary Figure 241. HPLC profile of the crude product **(*S*)-26** catalyzed by mutant SZ621**.**

(*rac*)-(3-(4-Chlorophenyl)tetrahydrofuran-3-yl)methanol

HPLC (ChiralPak IC, hexane: isopropanol =93:7, 1 mL/min, 220nm)

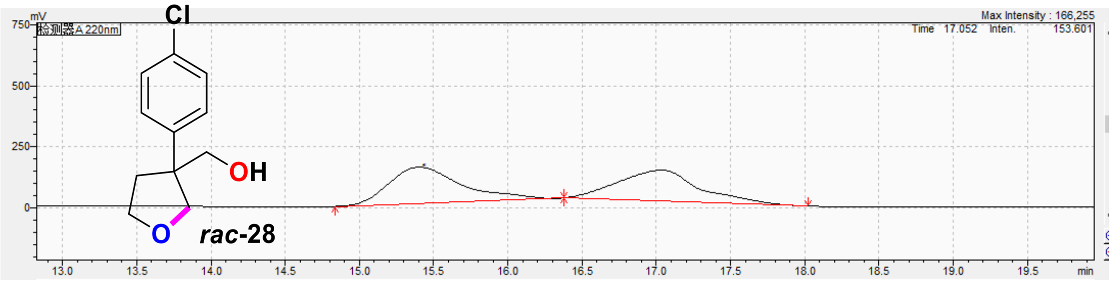

Peak #	Ret. Time [min]	Area mAu*s	Hight mAu	Width [min]	Area %
1	15.404	4989680	147873	0.492	50.611
2	17.032	4869205	127390	0.552	49.389

Supplementary Figure 242. HPLC profile of ***rac*-28** standard**.**

(*S*)-(3-(4-Chlorophenyl)tetrahydrofuran-3-yl)methanol (28)

SZ616: HPLC (ChiralPak IC, hexane: isopropanol =93:7, 1 mL/min, 220nm)

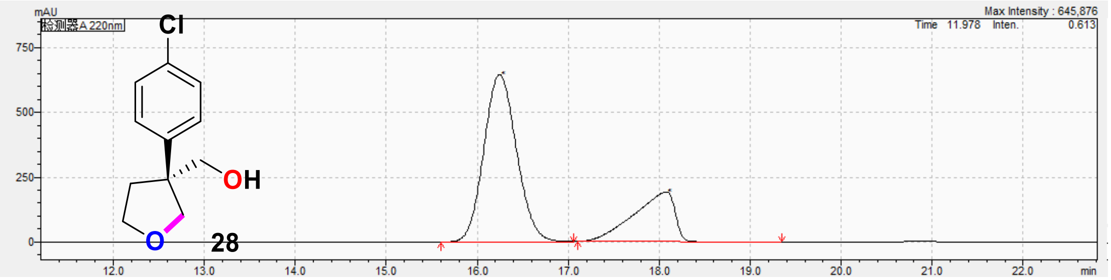

Peak #	Ret. Time [min]	Area mAu*s	Hight mAu	Width [min]	Area %
1	16.245	16143604	645409	0.390	73.014
2	18.072	5966800	192051	0.497	26.986

Supplementary Figure 243. HPLC profile of the crude product **(*S*)-28** catalyzed by mutant SZ616**.**

SZ621: HPLC (ChiralPak IC, hexane: isopropanol =93:7, 1 mL/min, 220nm)

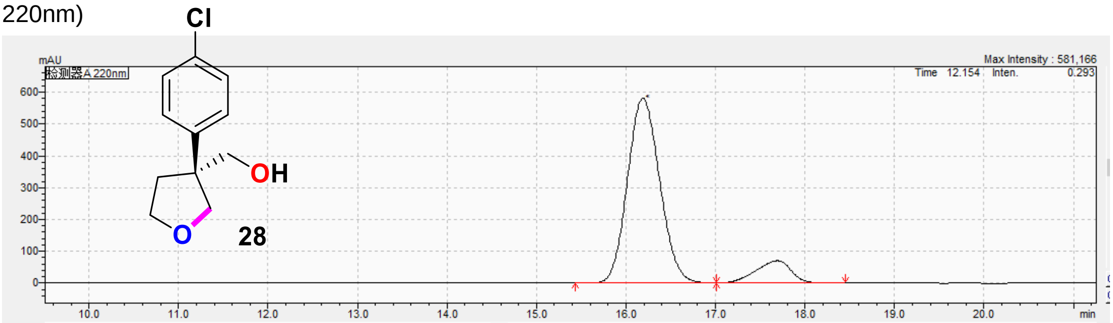

Peak #	Ret. Time [min]	Area mAu*s	Hight mAu	Width [min]	Area %
1	16.189	14565974	581106	0.392	88.239
2	17.690	1941524	70395	0.433	11.761

Supplementary Figure 244. HPLC profile of the crude product **(*S*)-28** catalyzed by mutant SZ621**.**

(*rac*)-(3-(4-Bromophenyl)tetrahydrofuran-3-yl)methanol

HPLC (ChiralPak IC, hexane: isopropanol =93:7, 1 mL/min, 220nm)

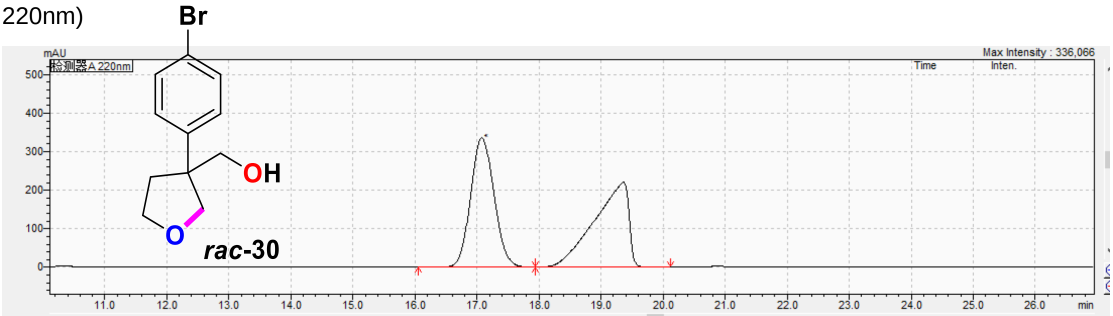

Peak #	Ret. Time [min]	Area mAu*s	Hight mAu	Width [min]	Area %
1	17.080	8822593	335573	0.411	50.885
2	19.358	8515643	220092	0.623	49.115

Supplementary Figure 245. HPLC profile of ***rac*-30** standard**.**

(*S*)-(3-(4-Bromophenyl)tetrahydrofuran-3-yl)methanol (30)

SZ616: HPLC (ChiralPak IC, hexane: isopropanol =93:7, 1 mL/min, 220nm)

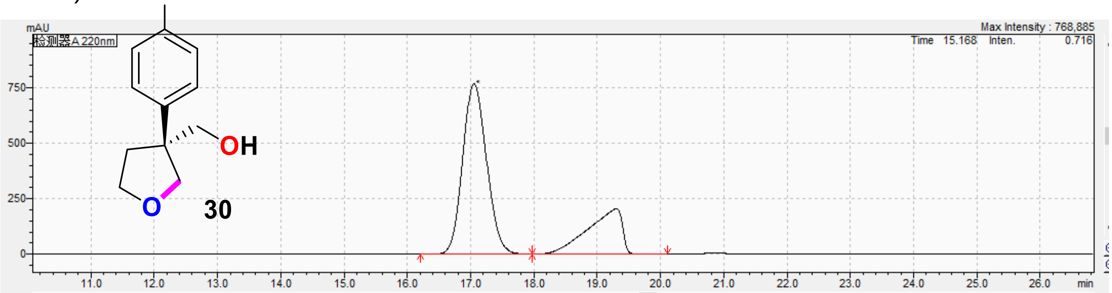

Peak #	Ret. Time [min]	Area mAu*s	Hight mAu	Width [min]	Area %
1	17.051	20225677	768486	0.411	72.355
2	19.299	7727578	204124	0.610	27.645

Supplementary Figure 246. HPLC profile of the crude product **(*S*)-30** catalyzed by mutant SZ616**.**

SZ621: HPLC (ChiralPak IC, hexane: isopropanol =93:7, 1 mL/min, 220nm)

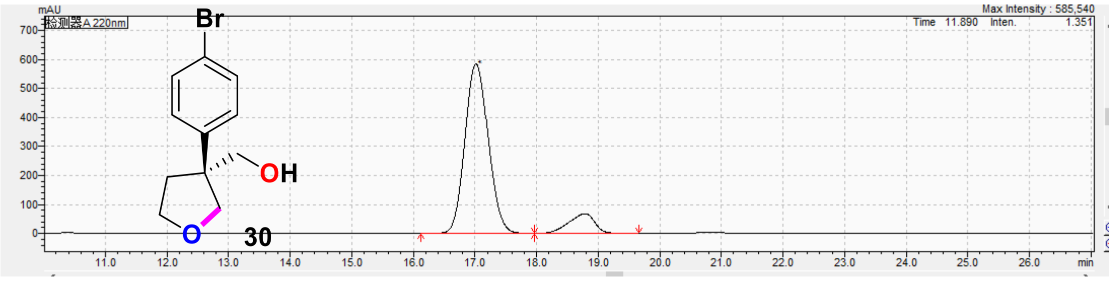

Peak #	Ret. Time [min]	Area mAu*s	Hight mAu	Width [min]	Area %
1	17.017	15442141	585046	0.412	88.516
2	18.785	2003534	67846	0.463	11.484

Supplementary Figure 247. HPLC profile of the crude product **(*S*)-30** catalyzed by mutant SZ621**.**

(*rac*)-(3-(4-(Trifluoromethyl)phenyl)tetrahydrofuran-3-yl)methanol

HPLC (ChiralPak IC, hexane:EtOH=93:7, 1 mL/min, 220nm)

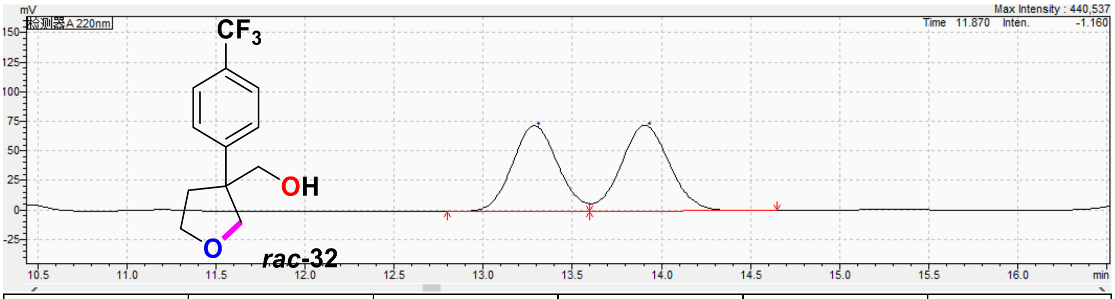

Peak #	Ret. Time [min]	Area mAu*s	Hight mAu	Width [min]	Area %
1	13.288	1275418	72363	0.275	48.572
2	13.907	1350429	72397	0.289	51.428

Supplementary Figure 248. HPLC profile of ***rac*-32** standard.

(*S*)-(3-(4-(Trifluoromethyl)phenyl)tetrahydrofuran-3-yl)methanol (32)

SZ616: HPLC (ChiralPak IC, hexane:EtOH=93:7, 1 mL/min, 220nm)

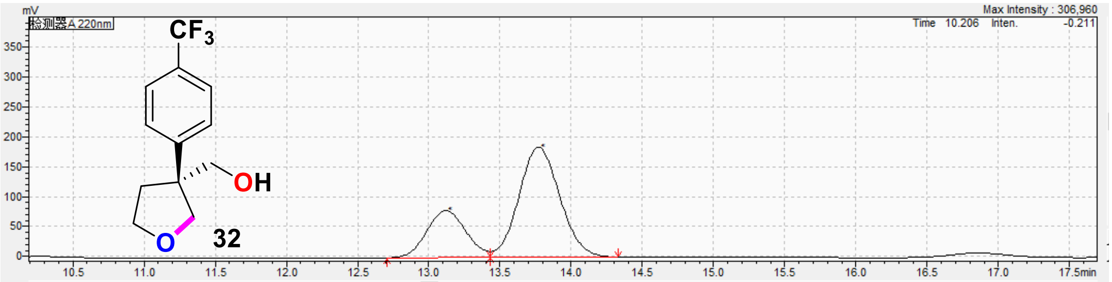

Peak #	Ret. Time [min]	Area mAu*s	Hight mAu	Width [min]	Area %
1	13.120	1506023	78789	0.300	28.959
2	13.771	3694514	184718	0.310	71.041

Supplementary Figure 249. HPLC profile of the crude product **(*S*)-32** catalyzed by mutant SZ616**.**

SZ621: HPLC (ChiralPak IC, hexane:EtOH=93:7, 1 mL/min, 220nm)

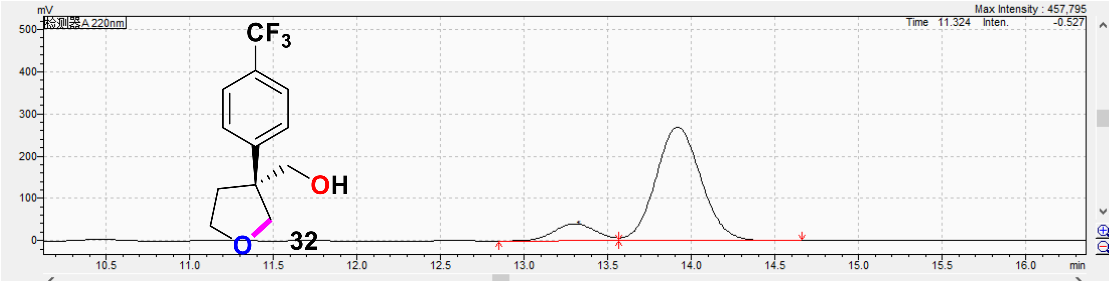

Peak #	Ret. Time [min]	Area mAu*s	Hight mAu	Width [min]	Area %
1	13.302	707422	40590	0.274	12.280
2	13.918	5053384	269663	0.291	87.720

Supplementary Figure 250. HPLC profile of the crude product **(*S*)-32** catalyzed by mutant SZ621**.**

(*rac*)-(3-Benzyltetrahydrofuran-3-yl)methyl benzoate

HPLC (ChiralPak IC, hexane : EtOH=97 : 3, 1 mL/min, 220 nm)

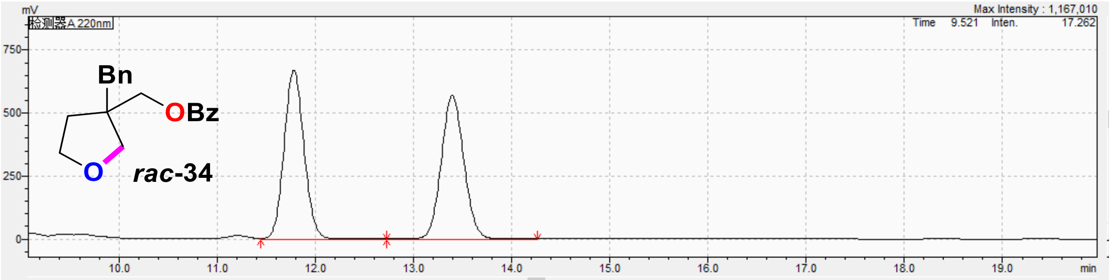

Peak #	Ret. Time [min]	Area mAu*s	Hight mAu	Width [min]	Area %
1	11.782	9484461	671372	0.218	50.810
2	13.396	9182203	570866	0.247	49.190

Supplementary Figure 251. HPLC profile of the derivative of ***rac*-34** standard**.**

(*S*)-(3-Benzyltetrahydrofuran-3-yl)methyl benzoate (34)

SZ616: HPLC (ChiralPak IC, hexane : EtOH=97 : 3, 1 mL/min, 220 nm)

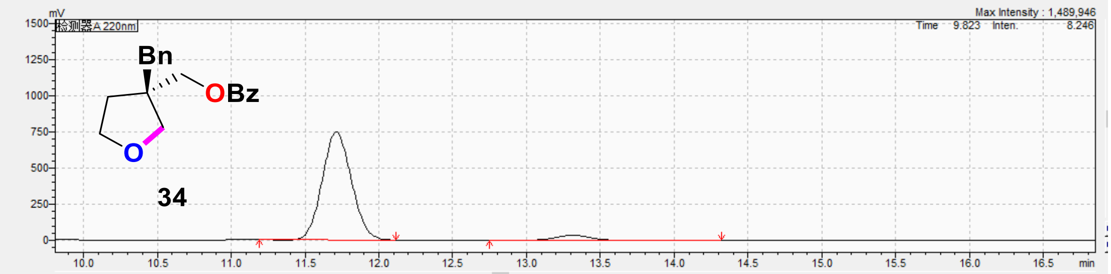

Peak #	Ret. Time [min]	Area mAu*s	Hight mAu	Width [min]	Area %
1	11.712	10126295	744165	0.214	95.187
2	13.310	511992	33356	0.241	4.813

Supplementary Figure 252. HPLC profile of the crude product **(*S*)-34** catalyzed by mutant SZ616**.**

SZ621: HPLC (ChiralPak IC, hexane : EtOH=97 : 3, 1 mL/min, 220 nm)

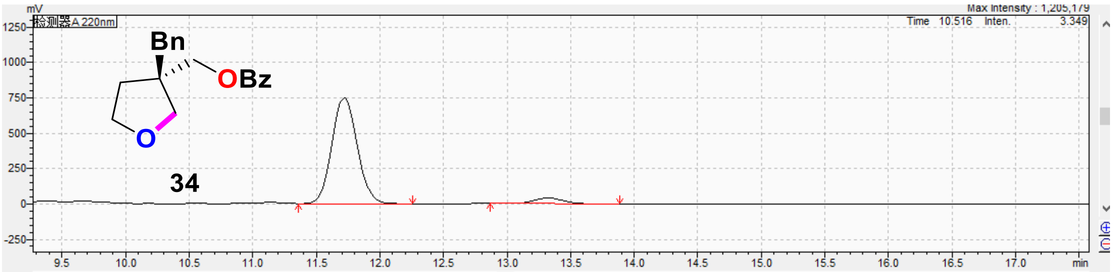

Peak #	Ret. Time [min]	Area mAu*s	Hight mAu	Width [min]	Area %
1	11.719	10373937	749278	0.215	94.518
2	13.324	601667	40284	0.240	5.482

Supplementary Figure 253. HPLC profile of the crude product **(*S*)-34** catalyzed by mutant SZ621**.**

(*rac*)-(3-Ethyltetrahydrofuran-3-yl)methanol

GC (CP7503, 25 m x 0.25 mm ID as the follows condition: 90°C, hold 2 min ; 3 °C/min, 140 °C; hold 3 min; 20 °C/min, 200 °C hold 2 min. N_2: 1.5 bar.)

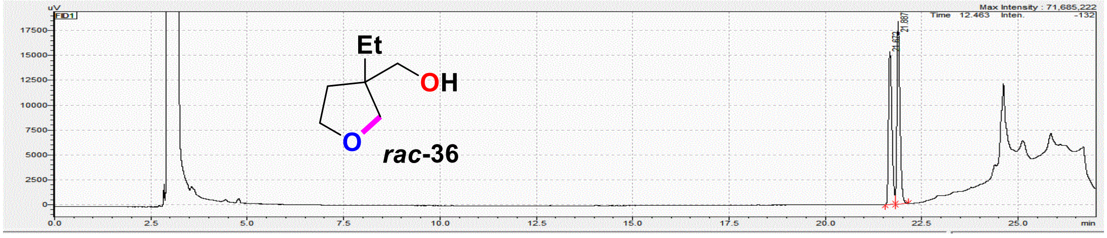

Peak #	Ret. Time [min]	Area mAu*s	Hight mAu	Width [min]	Area %
1	21.672	95943	15385	-	49.197
2	21.887	99075	18301	-	50.803

Supplementary Figure 254. GC profile of ***rac*-36** standard**.**

(*R*)-(3-Ethyltetrahydrofuran-3-yl)methanol (36)

SZ616: GC (CP7503, 25 m x 0.25 mm ID as the follows condition: 90°C, hold 2 min; 3 °C/min, 140 °C; hold 3 min; 20 °C/min, 200 °C hold 2 min. N_2: 1.5 bar.)

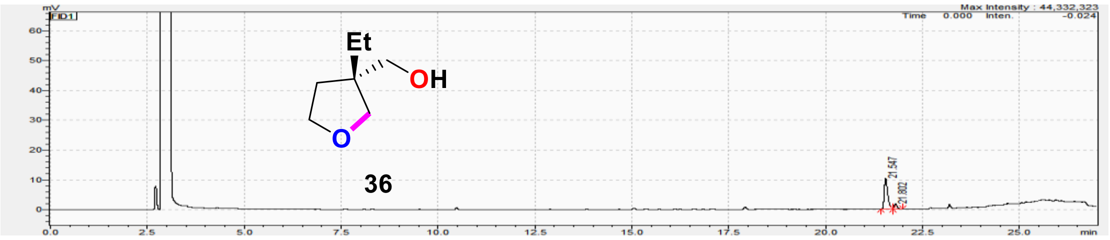

Peak #	Ret. Time [min]	Area mAu*s	Hight mAu	Width [min]	Area %
1	21.547	63812	10309	-	86.686
2	21.802	9801	1734	-	13.314

Supplementary Figure 255. GC profile of the crude product **(*S*)-36** catalyzed by mutant SZ616**.**

SZ621: GC (CP7503, 25 m x 0.25 mm ID as the follows condition: 90°C, hold 2 min; 3 °C/min, 140 °C; hold 3 min; 20 °C/min, 200 °C hold 2 min. N_2: 1.5 bar.)

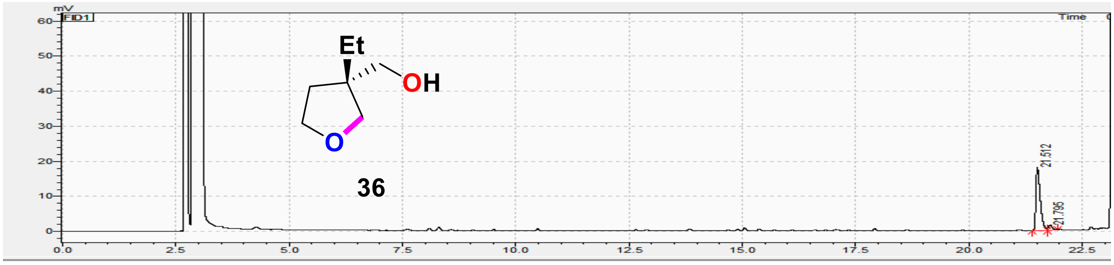

Peak #	Ret. Time [min]	Area mAu*s	Hight mAu	Width [min]	Area %
1	21.512	118359	17961	-	92.025
2	21.795	10257	1520	-	7.975

Supplementary Figure 256. GC profile of the crude product **(*S*)-36** catalyzed by mutant SZ621**.**

(*rac*)-(3-Methyltetrahydrofuran-3-yl)methanol

GC:(Hydrodex-*β*-TBDAc, 25 m x 0.25 mm ID, 25 m x 0.25 mm ID as the follows condition: 110 °C, 5 °C/min, 135 °C; hold 2 min; 20 °C/min, 200 °C hold 4 min. N_2: 1.5 bar.)

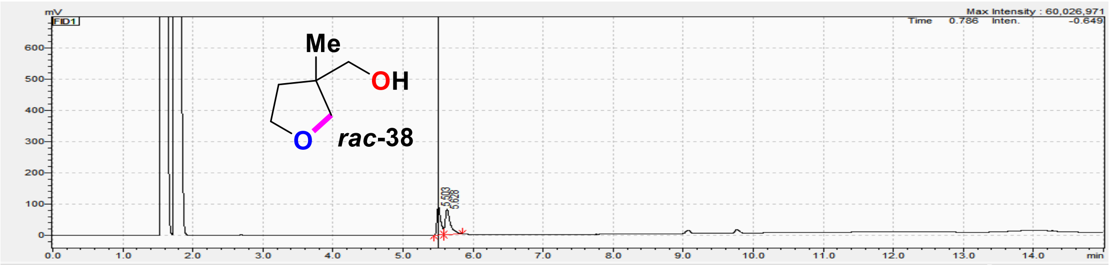

Peak #	Ret. Time [min]	Area mAu*s	Hight mAu	Width [min]	Area %
1	5.503	325842	89124	-	49.041
2	5.628	381872	78556	-	50.959

Supplementary Figure 257. GC profile of ***rac*-38** standard**.**

(*R*)-(3-Methyltetrahydrofuran-3-yl)methanol (38)

SZ616:GC (Hydrodex-*β*-TBDAc, 25 m x 0.25 mm ID, 25 m x 0.25 mm ID as the follows condition: 110 °C, 5 °C/min, 135 °C; hold 2 min; 20 °C/min, 200 °C hold 4 min. N_2: 1.5 bar.)

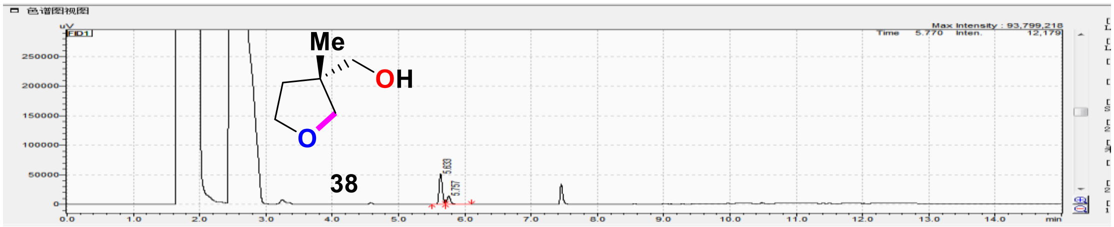

Peak #	Ret. Time [min]	Area mAu*s	Hight mAu	Width [min]	Area %
1	5.633	173631	51184		76.342
2	5.757	53808	13956		23.658

Supplementary Figure 258. GC profile of the crude product **(*S*)-38** catalyzed by mutant SZ616**.**

(*rac*)-(Tetrahydrofuran-3-yl)methanol

GC (Hydrodex-*β*-TBDAc, 25 m x 0.25 mm ID, 25 m x 0.25 mm ID as the follows condition: 110 °C, 5 °C/min, 135 °C; hold 2 min; 20 °C/min, 200 °C hold 4 min. N_2: 1.5 bar.)

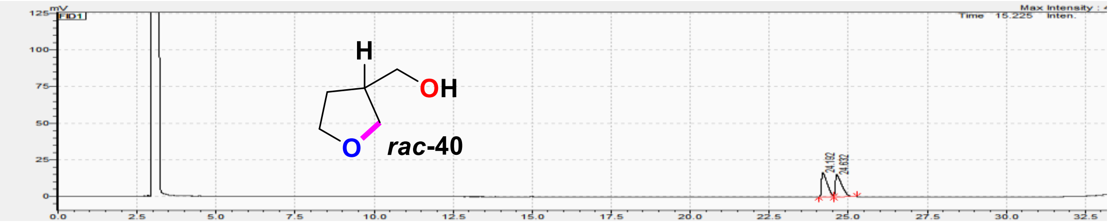

Peak #	Ret. Time [min]	Area mAu*s	Hight mAu	Width [min]	Area %
1	24.192	207572	16636	49.531	49.531
2	24.632	211499	15205	50.469	50.469

Supplementary Figure 259. GC profile of ***rac*-40** standard**.**

(*R*)-(Tetrahydrofuran-3-yl)methanol (40)

SZ616: GC (Hydrodex-*β*-TBDAc, 25 m x 0.25 mm ID, 25 m x 0.25 mm ID) as the follows condition: 110 °C, 5 °C/min, 135 °C; hold 2 min; 20 °C/min, 200 °C hold 4 min. N_2: 1.5 bar.)

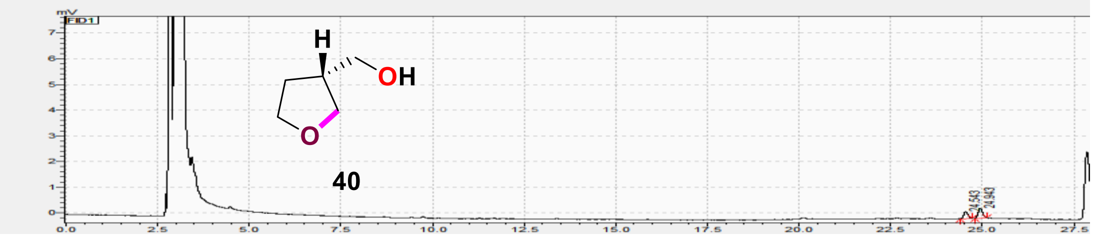

Peak #	Ret. Time [min]	Area mAu*s	Hight mAu	Width [min]	Area %
1	24.543	2217	274	39.971	39.971
2	24.943	3330	394	60.029	60.029

Supplementary Figure 260. GC profile of the crude product **(*R*)-40** catalyzed by mutant SZ616**.**

(*rac*)-(3-Phenyltetrahydro-2*H*-pyran-3-yl)methanol

HPLC (ChiralPak AD-H, hexane: isopropanol = 98.5:1.5, 1 mL/min, 220 nm)

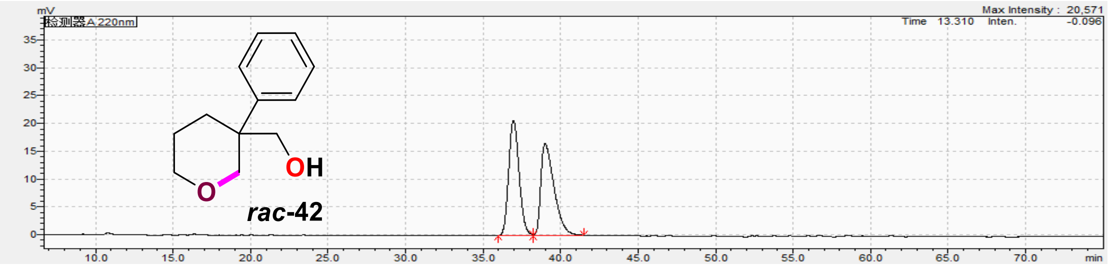

Peak #	Ret. Time [min]	Area mAu*s	Hight mAu	Width [min]	Area %
1	36.963	972095	20743	0.726	49.781
2	39.005	980638	16568	0.908	50.219

Supplementary Figure 261. HPLC profile of ***rac*-42** standard**.**

(*S*)-(3-Phenyltetrahydro-2*H*-pyran-3-yl)methanol (42)

SZ616: HPLC (ChiralPak AD-H, hexane: isopropanol = 98.5:1.5, 1 mL/min, 220 nm)

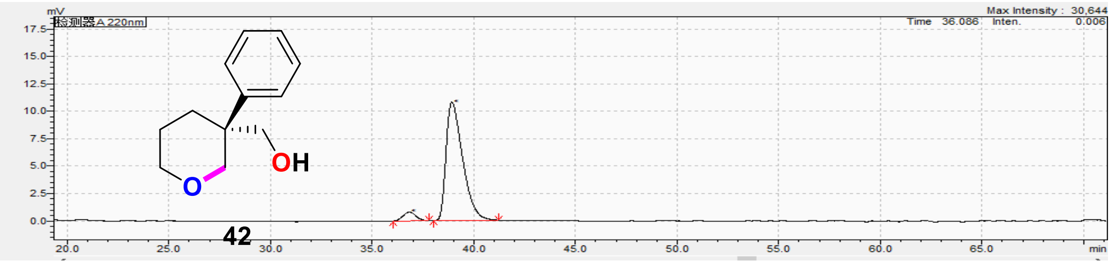

Peak #	Ret. Time [min]	Area mAu*s	Hight mAu	Width [min]	Area %
1	36.830	35776	811	0.698	5.511
2	38.923	613426	10805	0.872	94.489

Supplementary Figure 262. HPLC profile of the crude product **(*S*)-42** catalyzed by mutant SZ616**.**

(*rac*)-(3-Benzyltetrahydro-2*H*-pyran-3-yl)methanol

HPLC (ChiralPak AD-H, hexane: isopropanol = 90:10, 1 mL/min, 220 nm)

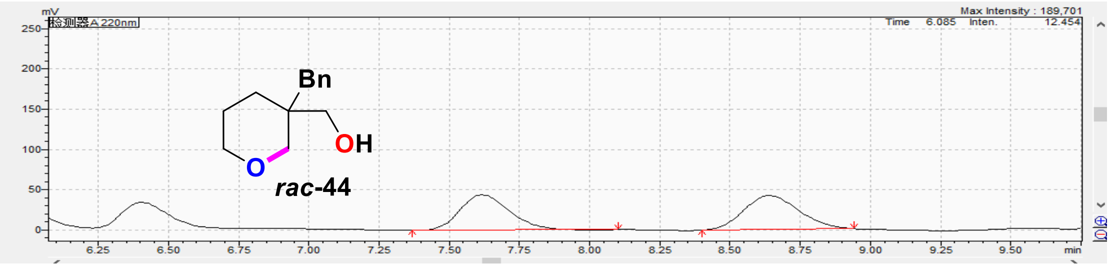

Peak #	Ret. Time [min]	Area mAu*s	Hight mAu	Width [min]	Area %
1	7.617	538084	43872	0.190	48.384
2	8.639	574033	42123	0.215	51.616

Supplementary Figure 263. HPLC profile of ***rac*-44** standard**.**

(*S*)-(3-Benzyltetrahydro-2*H*-pyran-3-yl)methanol (44)

SZ616: HPLC (ChiralPak AD-H, hexane: isopropanol = 90:10, 1 mL/min, 220 nm)

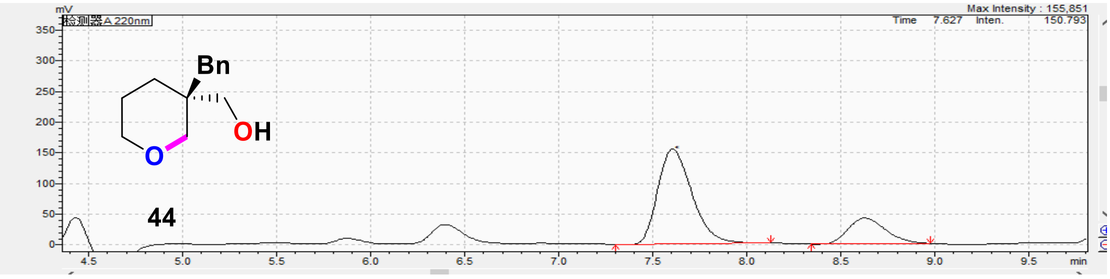

Peak #	Ret. Time [min]	Area mAu*s	Hight mAu	Width [min]	Area %
1	7.605	1948549	154060	0.195	77.088
2	8.628	579135	42053	0.215	22.912

Supplementary Figure 264. HPLC profile of the crude product **(*S*)-44** catalyzed by mutant SZ616**.**

(*rac*)-(1-Benzyl-3-phenylpyrrolidin-3-yl)methanol

HPLC (ChiralPak AD-H, hexane: isopropanol = 98:2, 1 mL/min, 220nm)

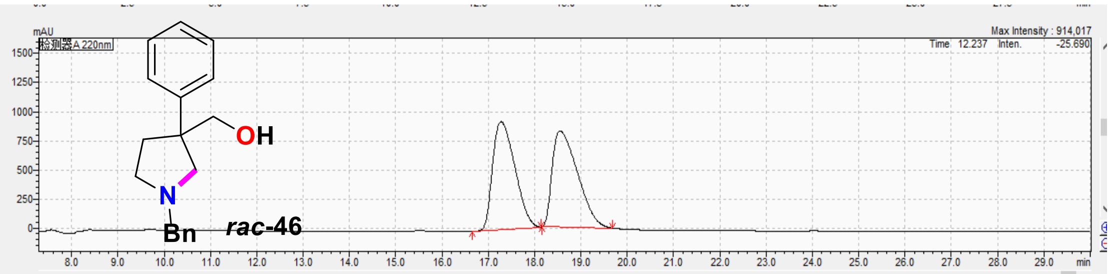

Peak #	Ret. Time [min]	Area mAu*s	Hight mAu	Width [min]	Area %
1	17.275	30934019	925964	0.543	49.549
2	18.551	31497110	828281	0.615	50.451

Supplementary Figure 265. HPLC profile of ***rac*-46** standard**.**

(*R*)-(1-Benzyl-3-phenylpyrrolidin-3-yl)methanol (46)

SZ611: HPLC (ChiralPak AD-H, hexane: isopropanol = 98:2, 1 mL/min, 220nm)

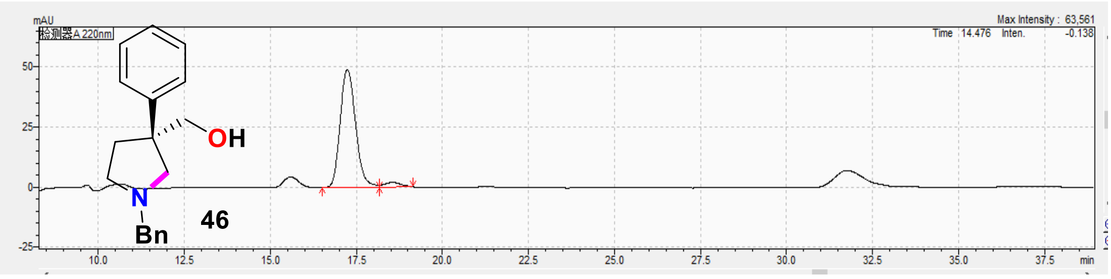

Peak #	Ret. Time [min]	Area mAu*s	Hight mAu	Width [min]	Area %
1	17.237	1522429	49087	0.481	95.865
2	18.553	65664	1903	0.577	4.135

Supplementary Figure 266. HPLC profile of the crude product **(*S*)-46** catalyzed by mutant SZ611**.**

(*rac*)-(1-Allyl-3-phenylpyrrolidin-3-yl)methanol

HPLC (ChiralPak AD-H, hexane: isopropanol = 98:2, 1 mL/min, 220nm)

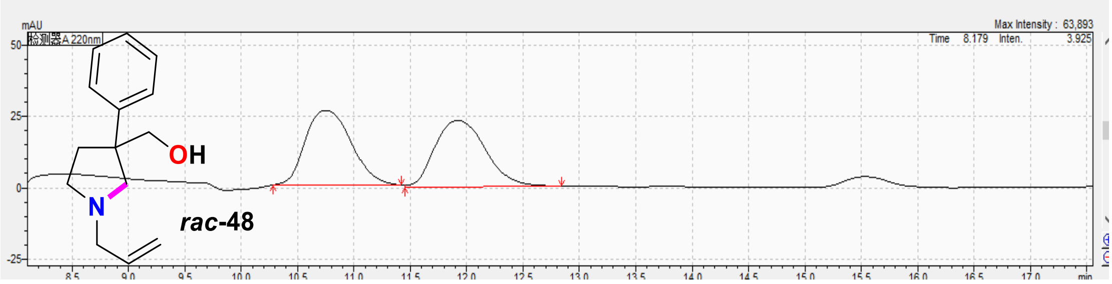

Peak #	Ret. Time [min]	Area mAu*s	Hight mAu	Width [min]	Area %
1	10.750	743193	26234	0.455	50.770
2	11.923	720651	23258	0.494	49.230

Supplementary Figure 267. HPLC profile of ***rac*-48** standard.

(*R*)-(1-Allyl-3-phenylpyrrolidin-3-yl)methanol (48)

SZ611: HPLC (ChiralPak AD-H, hexane: isopropanol = 98:2, 1 mL/min, 220

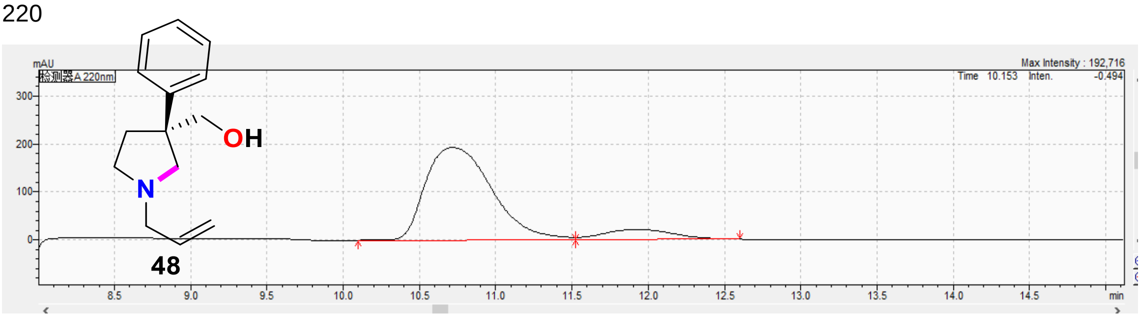

Peak #	Ret. Time [min]	Area mAu*s	Hight mAu	Width [min]	Area %
1	10.719	5824293	192863	0.477	89.730
2	11.925	666611	20648	0.515	10.270

Supplementary Figure 268. HPLC profile of the crude product **(*R*)-48** catalyzed by mutant SZ611**.**

(*rac*)-(3-Phenyl-1-(prop-2-yn-1-yl)pyrrolidin-3-yl)methanol

HPLC (ChiralPak AD-H, hexane: isopropanol = 98:2, 1 mL/min, 220nm)

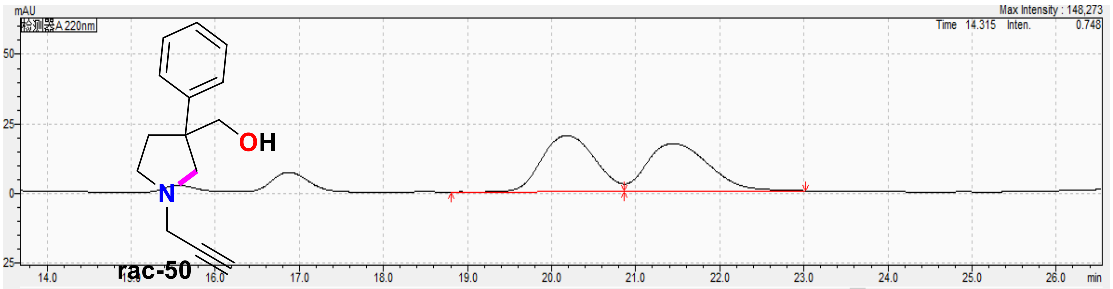

Peak #	Ret. Time [min]	Area mAu*s	Hight mAu	Width [min]	Area %
1	20.181	878497	20061	0.708	50.302
2	21.443	867961	17103	0.806	49.698

Supplementary Figure 269. HPLC profile of ***rac*-50** standard.

(*R*)-(3-Phenyl-1-(prop-2-yn-1-yl)pyrrolidin-3-yl)methanol (50)

HPLC (ChiralPak AD-H, hexane: isopropanol = 98:2, 1 mL/min, 220nm)

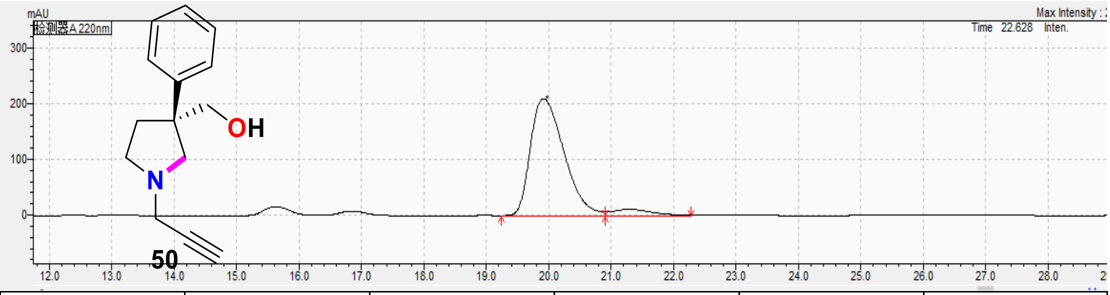

Peak #	Ret. Time [min]	Area mAu*s	Hight mAu	Width [min]	Area %
1	19.915	7877383	210097	0.587	93.708
2	21.288	528964	11530	0.364	6.292

Supplementary Figure 270. HPLC profile of the crude product **(*R*)-50** catalyzed by mutant SZ611**.**

(*rac*)-(1-Butyl-3-phenylpyrrolidin-3-yl)methanol

HPLC (ChiralPak IC, hexane: isopropanol = 97:3, 1 mL/min, 220nm)

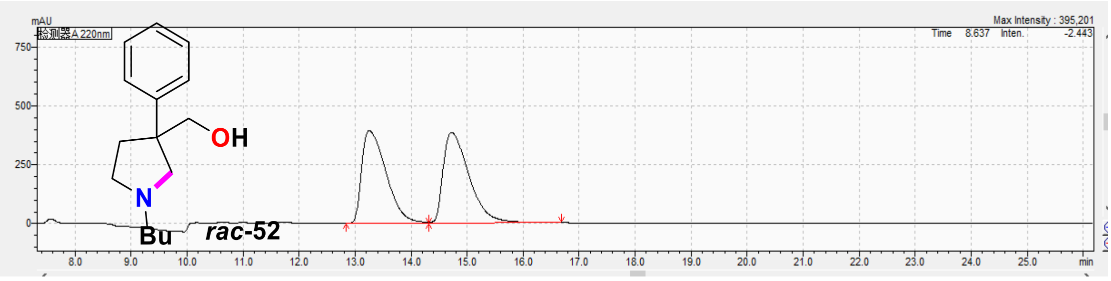

Peak #	Ret. Time [min]	Area mAu*s	Hight mAu	Width [min]	Area %
1	13.257	11917845	393359	0.480	50.080
2	14.724	11879932	380730	0.488	49.920

Supplementary Figure 271. HPLC profile of ***rac*-52** standard**.**

(*R*)-(1-Butyl-3-phenylpyrrolidin-3-yl)methanol (52)

HPLC (ChiralPak IC, hexane: isopropanol = 97:3, 1 mL/min, 220nm)

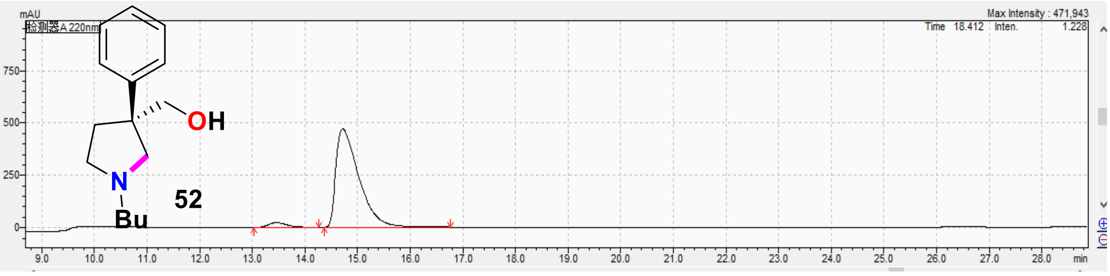

Peak #	Ret. Time [min]	Area mAu*s	Hight mAu	Width [min]	Area %
1	13.459	568627	22908	0.378	3.835
2	14.718	14258151	469985	0.461	96.165

Supplementary Figure 272. HPLC profile of the crude product **(*R*)-52** catalyzed by mutant SZ611**.**

(*rac*)-(1,3-Diphenylpyrrolidin-3-yl)methanol

HPLC (ChiralPak AD-H, hexane: isopropanol = 98:2, 1 mL/min, 220nm)

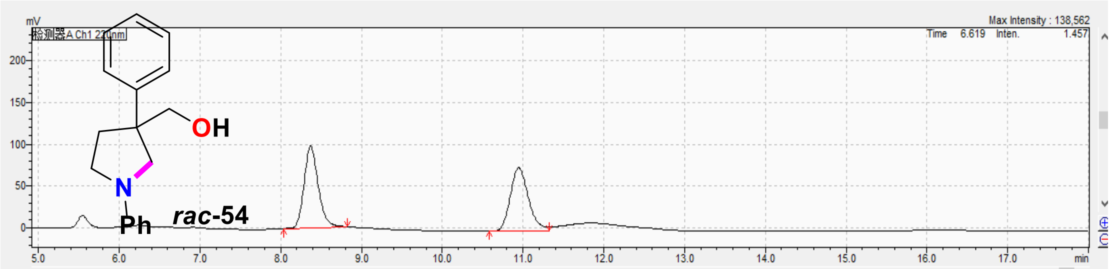

Peak #	Ret. Time [min]	Area mAu*s	Hight mAu	Width [min]	Area %
1	8.371	1183179	97188	0.184	49.585
2	10.947	1202996	75978	0.238	50.415

Supplementary Figure 273. HPLC profile of ***rac*-54** standard.

(*R*)-(1,3-Diphenylpyrrolidin-3-yl)methanol (54)

HPLC (ChiralPak AD-H, hexane: isopropanol =90:10, 1 mL/min, 220nm)

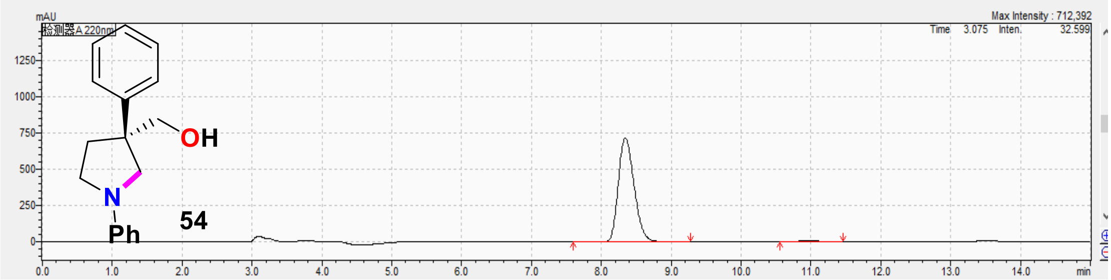

Peak #	Ret. Time [min]	Area mAu*s	Hight mAu	Width [min]	Area %
1	8.343	11189524	714469	0.246	98.567
2	10.963	162698	9166	0.276	1.433

Supplementary Figure 274. HPLC profile of the crude product **(*R*)-54** catalyzed by mutant SZ611**.**

(*rac*)-(3-Phenyl-1-(*p*-tolyl)pyrrolidin-3-yl)methanol

HPLC (ChiralPak AD-H, hexane: isopropanol =90:10, 1 mL/min, 220nm)

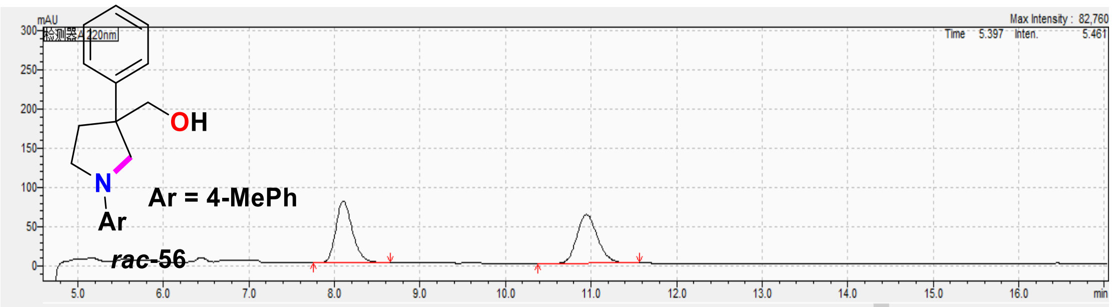

Peak #	Ret. Time [min]	Area mAu*s	Hight mAu	Width [min]	Area %
1	8.104	1052669	78448	0.205	50.297
2	10.948	1040243	62101	0.258	49.703

Supplementary Figure 275. HPLC profile of ***rac*-56** standard.

(*R*)-(3-Phenyl-1-(*p*-tolyl)pyrrolidin-3-yl)methanol (56)

HPLC (ChiralPak AD-H, hexane: isopropanol = 90:10, 1 mL/min, 220nm)

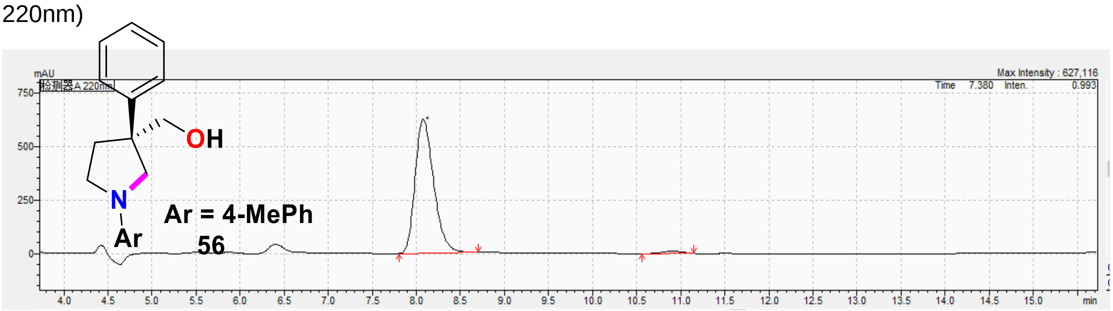

Peak #	Ret. Time [min]	Area mAu*s	Hight mAu	Width [min]	Area %
1	8.077	9129011	624752	0.225	98.131
2	10.911	173903	11072	0.255	1.869

Supplementary Figure 276. HPLC profile of the crude product **(*R*)-56** catalyzed by mutant SZ611**.**

(*rac*)-(1-(4-Chlorophenyl)-3-phenylpyrrolidin-3-yl)methanol

HPLC (ChiralPak AD-H, hexane: isopropanol =90:10, 1 mL/min, 220nm)

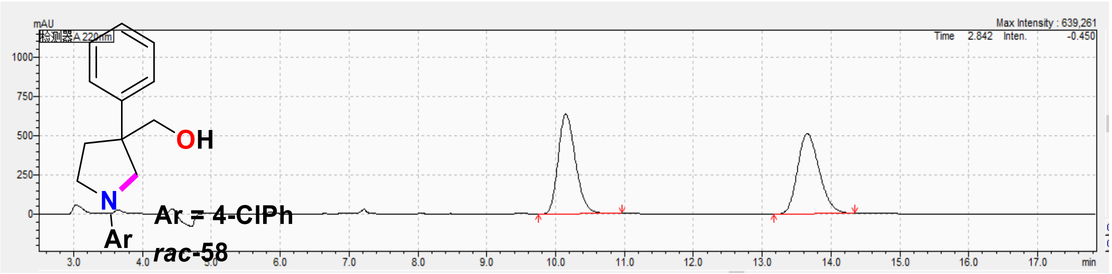

Peak #	Ret. Time [min]	Area mAu*s	Hight mAu	Width [min]	Area %
1	10.149	11043076	634807	0.270	49.634
2	13.655	11205965	511239	0.341	50.366

Supplementary Figure 277. HPLC profile of ***rac*-58** standard**.**

(*R*)-(1-(4-Chlorophenyl)-3-phenylpyrrolidin-3-yl)methanol (58)

HPLC (ChiralPak AD-H, hexane: isopropanol =90:10, 1 mL/min, 220nm)

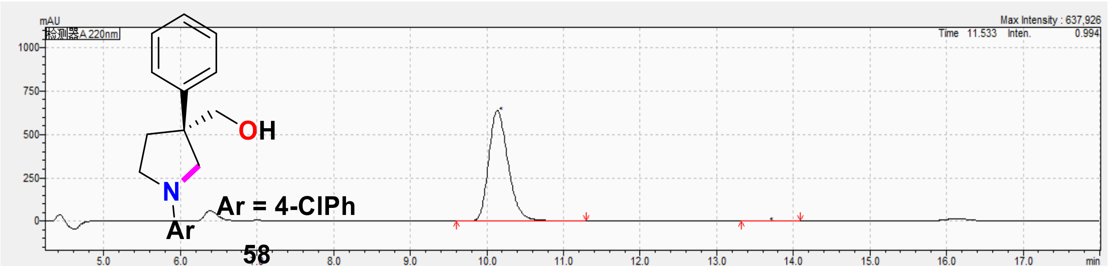

Peak #	Ret. Time [min]	Area mAu*s	Hight mAu	Width [min]	Area %
1	10.135	11179283	636565	0.269	99.261
2	13.666	83285	4318	0.309	0.739

Supplementary Figure 278. HPLC profile of the crude product **(*R*)-58** catalyzed by mutant SZ611**.**

(*rac*)-(1-Benzyl-3-(*p*-tolyl)pyrrolidin-3-yl)methanol

HPLC (ChiralPak AD-H, hexane: isopropanol =90:10, 1 mL/min, 220nm)

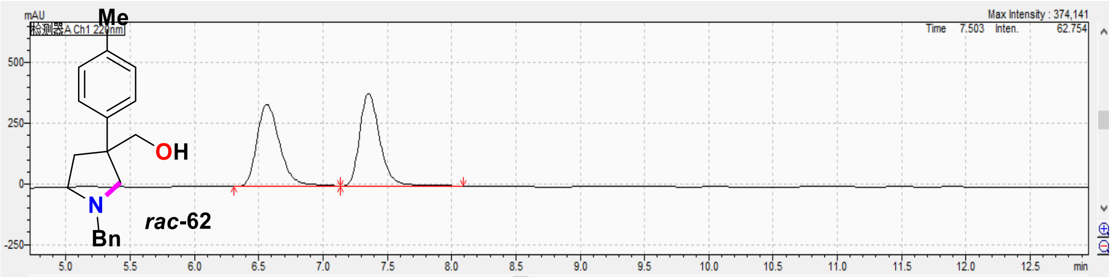

Peak #	Ret. Time [min]	Area mAu*s	Hight mAu	Width [min]	Area %
1	6.564	4211228	338834	0.188	50.106
2	7.354	4193404	383367	0.164	49.894

Supplementary Figure 279. HPLC profile of ***rac*-62** standard.

(*R*)-(1-Benzyl-3-(*p*-tolyl)pyrrolidin-3-yl)methanol (62)

SZ611: HPLC (ChiralPak AD-H, hexane: isopropanol =90:10, 1 mL/min, 220nm)

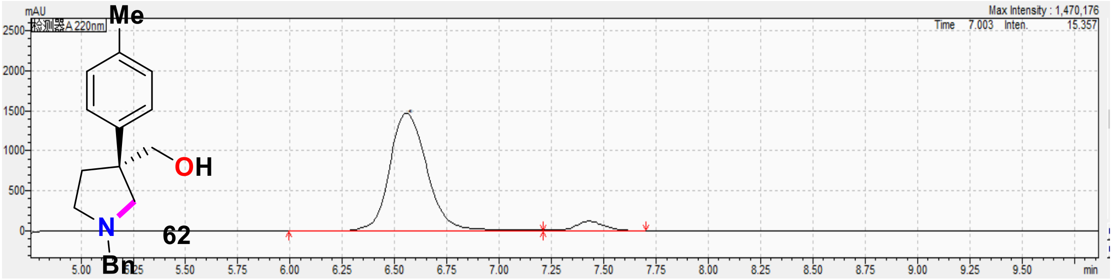

Peak #	Ret. Time [min]	Area mAu*s	Hight mAu	Width [min]	Area %
1	6.557	18182267	1469351	0.187	94.110
2	7.428	1137919	118380	0.142	5.890

Supplementary Figure 280. HPLC profile of the crude product **(*R*)-62** catalyzed by mutant SZ611**.**

(*rac*)-(1-Benzyl-3-(*m*-tolyl)pyrrolidin-3-yl)methanol

HPLC (ChiralPak AD-H, hexane: isopropanol =90:10, 1 mL/min, 220nm)

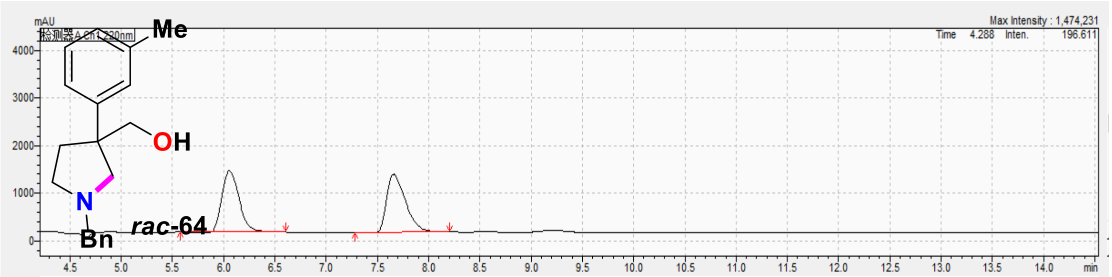

Peak #	Ret. Time [min]	Area mAu*s	Hight mAu	Width [min]	Area %
1	6.056	14992217	1286165	0.183	49.316
2	7.658	15407966	1217543	0.198	50.684

Supplementary Figure 281. HPLC profile of ***rac*-64** standard**.**

(*R*)-(1-Benzyl-3-(*m*-tolyl)pyrrolidin-3-yl)methanol (64)

SZ611: HPLC (ChiralPak AD-H, hexane: isopropanol =90:10, 1 mL/min, 220nm)

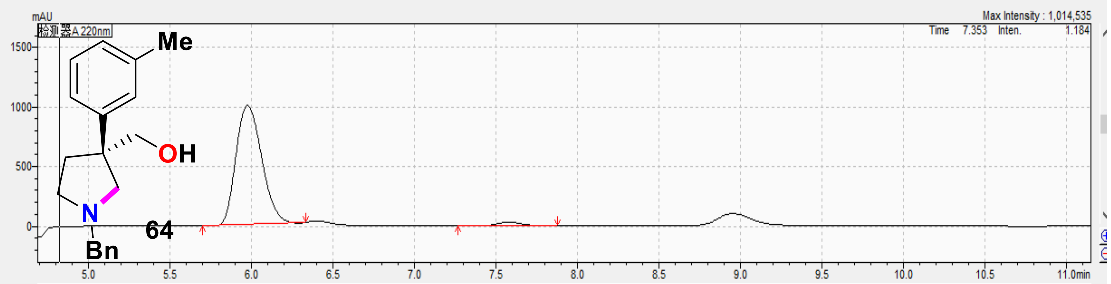

Peak #	Ret. Time [min]	Area mAu*s	Hight mAu	Width [min]	Area %
1	5.975	10955269	999736	0.174	97.319
2	7.577	301756	31876	0.158	2.681

Supplementary Figure 282. HPLC profile of the crude product **(*R*)-64** catalyzed by mutant SZ611**.**

(*rac*)-(1-Benzyl-3-(3,5-dimethylphenyl)pyrrolidin-3-yl)methanol

HPLC (ChiralPak AD-H, hexane: isopropanol = 90:10, 1 mL/min, 220nm)

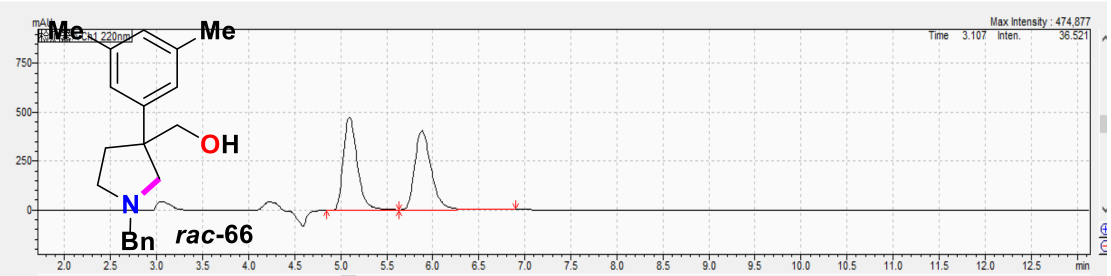

Peak #	Ret. Time [min]	Area mAu*s	Hight mAu	Width [min]	Area %
1	5.094	5010014	475978	0.160	49.968
2	5.882	5016409	405299	0.189	50.032

Supplementary Figure 283. HPLC profile of ***rac*-66** standard**.**

(*R*)-(1-Benzyl-3-(3,5-dimethylphenyl)pyrrolidin-3-yl)methanol (66)

HPLC (ChiralPak AD-H, hexane: isopropanol = 90:10, 1 mL/min, 220nm)

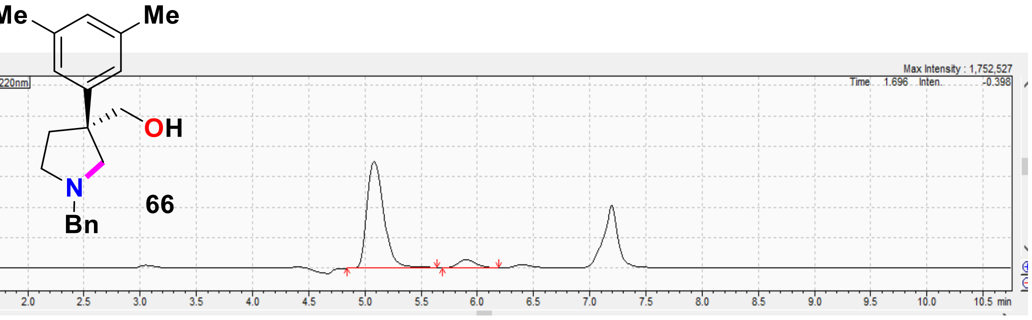

Peak #	Ret. Time [min]	Area mAu*s	Hight mAu	Width [min]	Area %
1	5.083	18420082	1750013	0.163	92.821
2	5.900	1424577	132573	0.168	7.179

Supplementary Figure 284. HPLC profile of the crude product **(*R*)-66** catalyzed by mutant SZ611**.**

(*rac*)-(1-Allyl-3-(4-ethylphenyl)pyrrolidin-3-yl)methanol

HPLC (ChiralPak AD-H, hexane: isopropanol =98:2, 1 mL/min, 220nm)

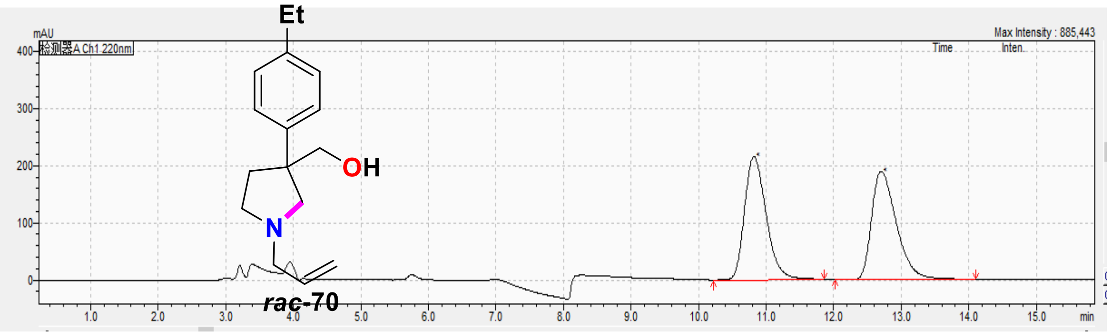

Peak #	Ret. Time [min]	Area mAu*s	Hight mAu	Width [min]	Area %
1	10.820	4847269	216434	0.342	50.167
2	12.703	4815027	189189	0.390	49.833

Supplementary Figure 285. HPLC profile of ***rac*-70** standard**.**

(*R*)-(1-Allyl-3-(4-ethylphenyl)pyrrolidin-3-yl)methanol (70)

SZ611: HPLC (ChiralPak AD-H, hexane: isopropanol =98:2, 1 mL/min, 220nm)

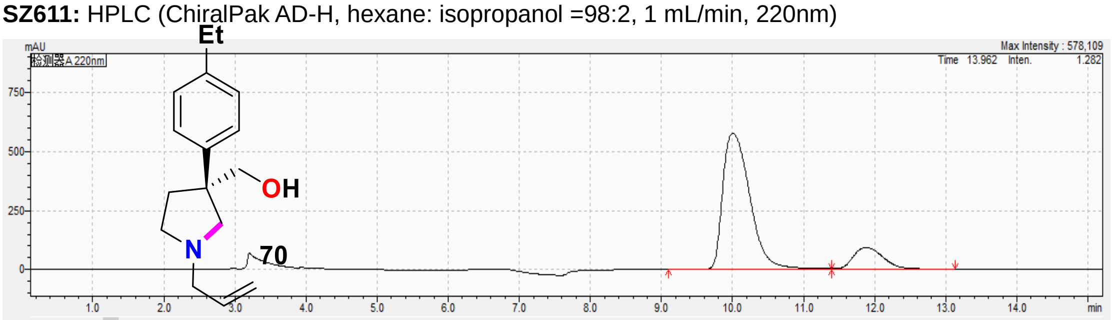

Peak #	Ret. Time [min]	Area mAu*s	Hight mAu	Width [min]	Area %
1	10.006	15336896	577557	0.412	84.877
2	11.877	2731539	91575	0.462	15.117

Supplementary Figure 286. HPLC profile of the crude product **(*R*)-70** catalyzed by mutant SZ611**.**

(*rac*)-(1-Benzyl-3-(4-methoxyphenyl)pyrrolidin-3-yl)methanol

HPLC (ChiralPak AD-H, hexane: isopropanol =90:10, 1 mL/min, 220nm)

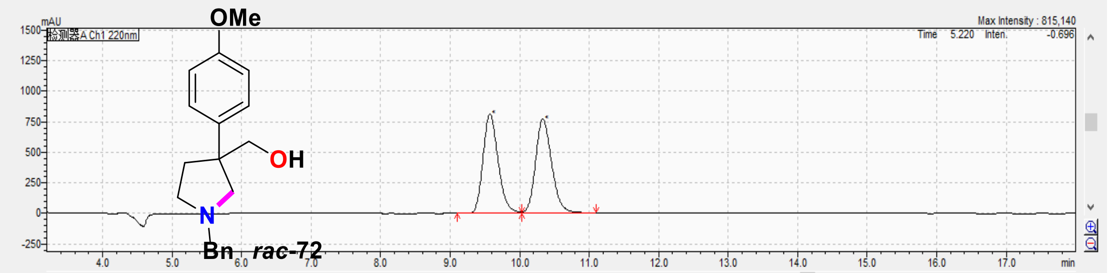

Peak #	Ret. Time [min]	Area mAu*s	Hight mAu	Width [min]	Area %
1	9.572	12310556	813835	0.232	49.663
2	10.333	12477557	772774	0.246	50.337

Supplementary Figure 287. HPLC profile of ***rac*-72** standard**.**

(*R*)-(1-Benzyl-3-(4-methoxyphenyl)pyrrolidin-3-yl)methanol (72)

SZ611: HPLC (ChiralPak AD-H, hexane: isopropanol =90:10, 1 mL/min, 220nm)

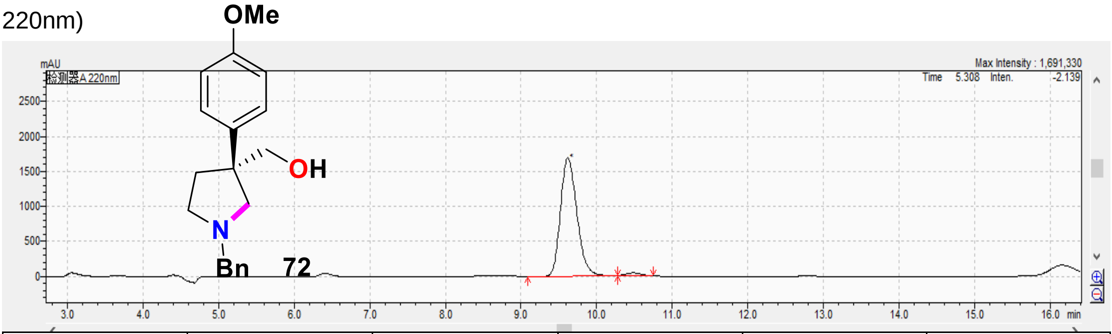

Peak #	Ret. Time [min]	Area mAu*s	Hight mAu	Width [min]	Area %
1	9.620	25300138	1685804	0.232	97.833
2	10.483	560388	40749	0.223	2.167

Supplementary Figure 288. HPLC profile of the crude product **(*R*)-72** catalyzed by mutant SZ611**.**

(*rac*)-(1-Allyl-3-(4-(methylthio)phenyl)pyrrolidin-3-yl)methanol

HPLC (ChiralPak AD-H, hexane: isopropanol =98:2, 1 mL/min, 220nm)

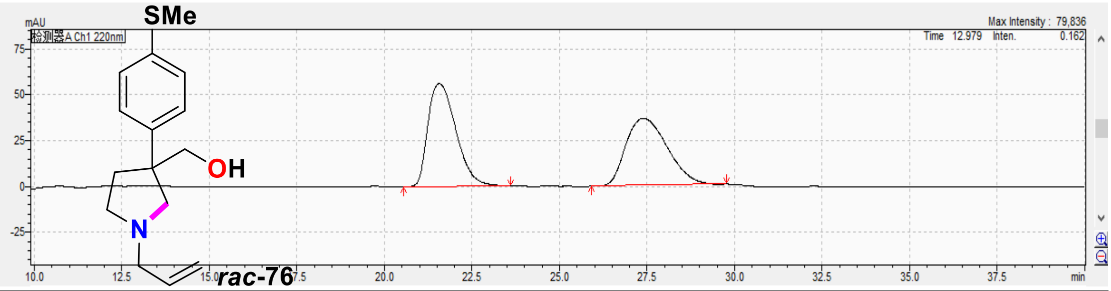

Peak #	Ret. Time [min]	Area mAu*s	Hight mAu	Width [min]	Area %
1	21.560	3104365	56087	0.873	50.948
2	27.393	2988859	36578	1.319	49.052

Supplementary Figure 289. HPLC profile of ***rac*-76** standard**.**

(*R*)-(1-Allyl-3-(4-(methylthio)phenyl)pyrrolidin-3-yl)methanol (76)

SZ611: HPLC (ChiralPak AD-H, hexane: isopropanol =90:10, 1 mL/min, 220nm)

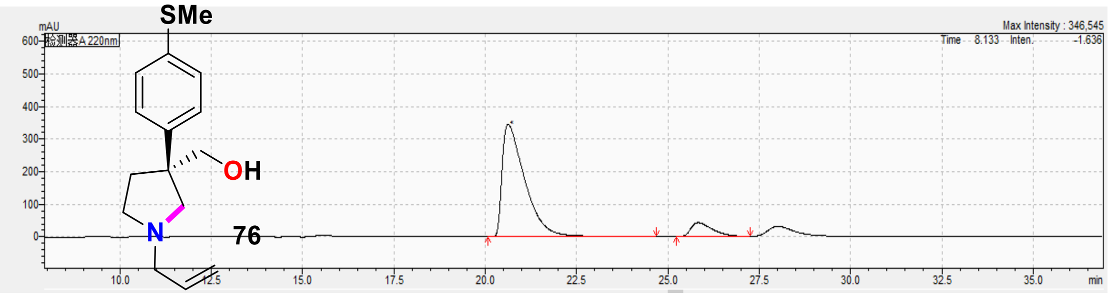

Peak #	Ret. Time [min]	Area mAu*s	Hight mAu	Width [min]	Area %
1	20.629	14961113	346732	0.640	89.022
2	25.830	1845012	43409	0.644	10.978

Supplementary Figure 290. HPLC profile of the crude product **(*R*)-76** catalyzed by mutant SZ611**.**

(*rac*)-(1-Benzyl-3-(4-fluorophenyl)pyrrolidin-3-yl)methanol

HPLC (ChiralPak IC, hexane: isopropanol =97:3, 1 mL/min, 220nm)

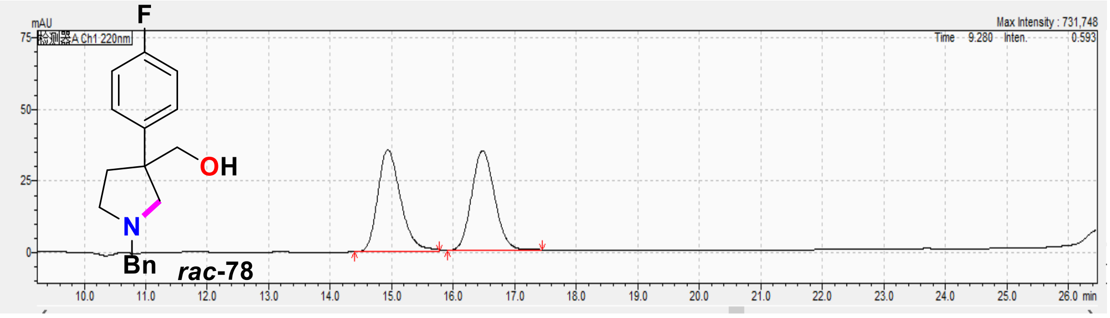

Peak #	Ret. Time [min]	Area mAu*s	Hight mAu	Width [min]	Area %
1	14.936	918694	35460	0.393	50.279
2	16.477	908491	34818	0.404	49.721

Supplementary Figure 291. HPLC profile of ***rac*-78** standard.

(*R*)-(1-Benzyl-3-(4-fluorophenyl)pyrrolidin-3-yl)methanol (78)

SZ611: HPLC (ChiralPak IC, hexane: isopropanol = 97:3, 1 mL/min, 220nm)

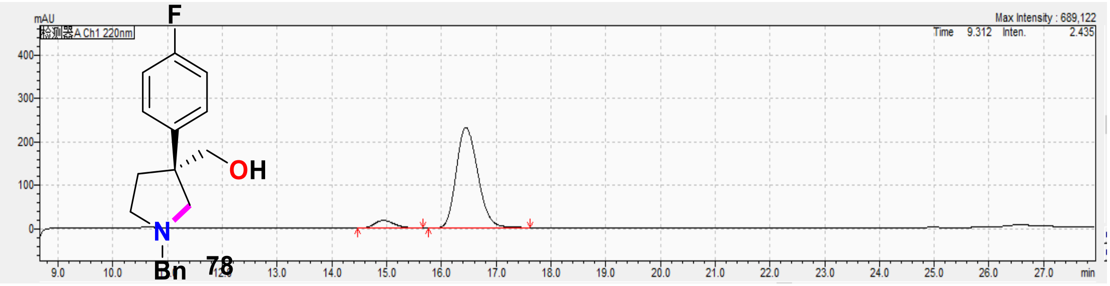

Peak #	Ret. Time [min]	Area mAu*s	Hight mAu	Width [min]	Area %
1	14.940	426248	17353	0.385	6.418
2	16.450	6215010	230525	0.420	93.582

Supplementary Figure 292. HPLC profile of the crude product **(*R*)-78** catalyzed by mutant SZ611**.**

(*rac*)-(1-Benzyl-3-(4-chlorophenyl)pyrrolidin-3-yl)methanol

HPLC (ChiralPak IC, hexane: isopropanol = 97:3, 1 mL/min, 220nm)

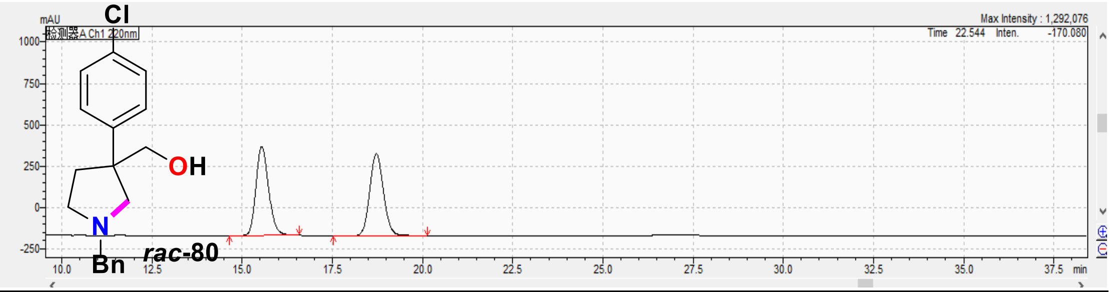

Peak #	Ret. Time [min]	Area mAu*s	Hight mAu	Width [min]	Area %
1	15.546	13118577	536647	0.375	50.022
2	18.718	13107090	495097	0.409	49.978

Supplementary Figure 293. HPLC profile of ***rac*-80** standard.

(*R*)-(1-Benzyl-3-(4-chlorophenyl)pyrrolidin-3-yl)methanol (80)

SZ611: HPLC (ChiralPak IC, hexane: isopropanol = 97:3, 1 mL/min, 220nm)

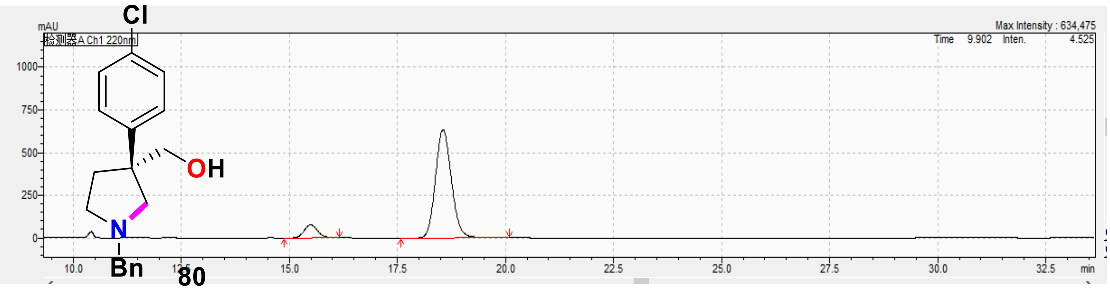

Peak #	Ret. Time [min]	Area mAu*s	Hight mAu	Width [min]	Area %
1	15.489	1568399	75035	0.326	8.782
2	18.555	16290836	633964	0.394	91.218

Supplementary Figure 294. HPLC profile of the crude product **(*R*)-80** catalyzed by mutant SZ611**.**

(*rac*)-(1-Benzyl-3-(4-bromophenyl)pyrrolidin-3-yl)methanol

HPLC (ChiralPak AD-H, hexane: isopropanol = 90:10, 1 mL/min, 220nm)

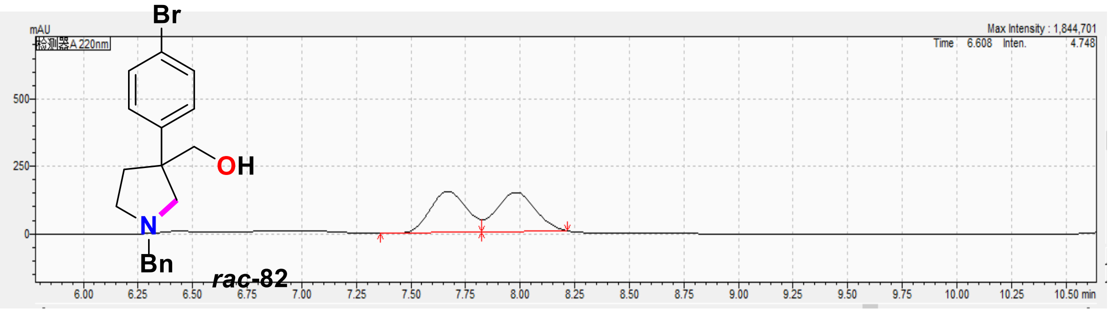

Peak #	Ret. Time [min]	Area mAu*s	Hight mAu	Width [min]	Area %
1	7.669	1749743	151452	0.187	49.564
2	7.980	1780521	145358	0.197	50.436

Supplementary Figure 295. HPLC profile of ***rac*-82** standard**.**

(*R*)-(1-Benzyl-3-(4-bromophenyl)pyrrolidin-3-yl)methanol (82)

SZ611: HPLC (ChiralPak AD-H, hexane: isopropanol = 90:10, 1 mL/min, 220nm)

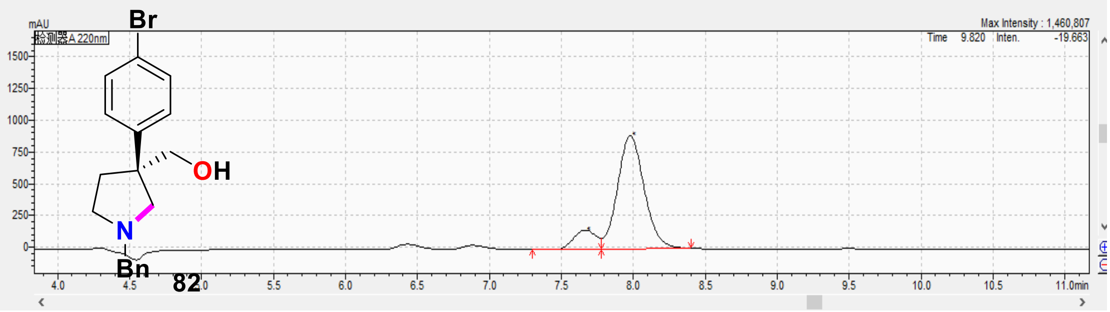

Peak #	Ret. Time [min]	Area mAu*s	Hight mAu	Width [min]	Area %
1	7.666	1625354	150633	--	12.371
2	7.979	11512648	890350	0.198	87.629

Supplementary Figure 296. HPLC profile of the crude product **(*R*)-82** catalyzed by mutant SZ611**.**

(*rac*)-(1-Allyl-3-(4-(trifluoromethyl)phenyl)pyrrolidin-3-yl)methanol

HPLC (ChiralPak AD-H, hexane: isopropanol = 98:2, 1 mL/min, 220nm)

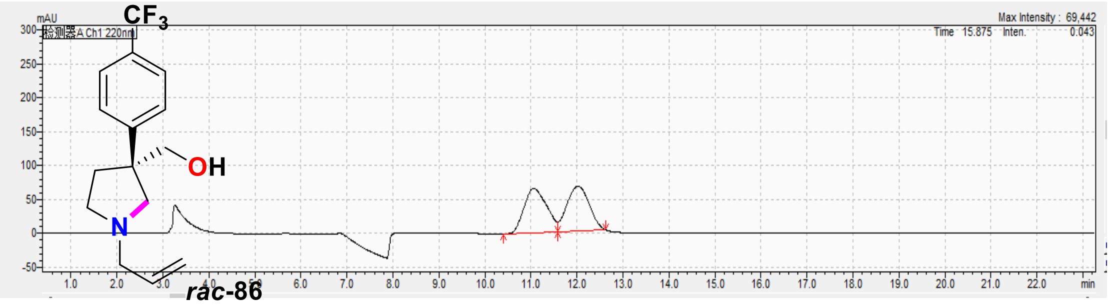

Peak #	Ret. Time [min]	Area mAu*s	Hight mAu	Width [min]	Area %
1	11.060	2228137	65639	0.568	49.897
2	12.022	2237294	65907	0.557	50.103

Supplementary Figure 297. HPLC profile of ***rac*-86** standard**.**

(*R*)-(1-Allyl-3-(4-(trifluoromethyl)phenyl)pyrrolidin-3-yl)methanol (86)

SZ611: HPLC (ChiralPak AD-H, hexane: isopropanol = 98:2, 1 mL/min, 220nm)

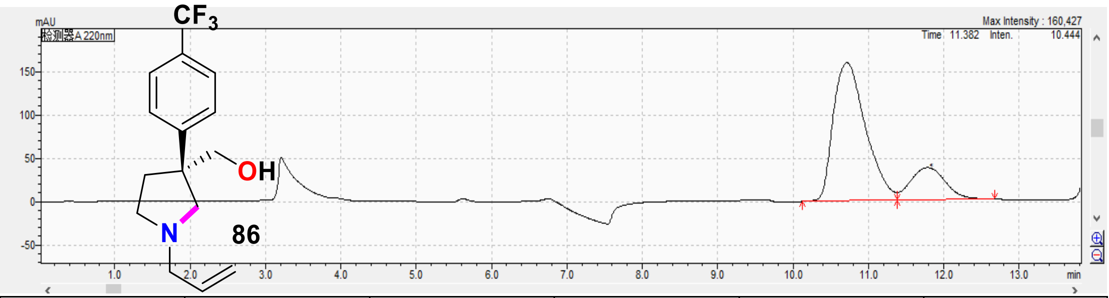

Peak #	Ret. Time [min]	Area mAu*s	Hight mAu	Width [min]	Area %
1	10.716	4780217	159655	0.468	79.781
2	11.787	1211488	37402	0.514	20.219

Supplementary Figure 298. HPLC profile of the crude product **(*R*)-86** catalyzed by mutant SZ611**.**

(*rac*)-(1,3-Dibenzylpyrrolidin-3-yl)methanol

HPLC (ChiralPak AD-H, hexane: isopropanol = 98:2, 1 mL/min, 220nm)

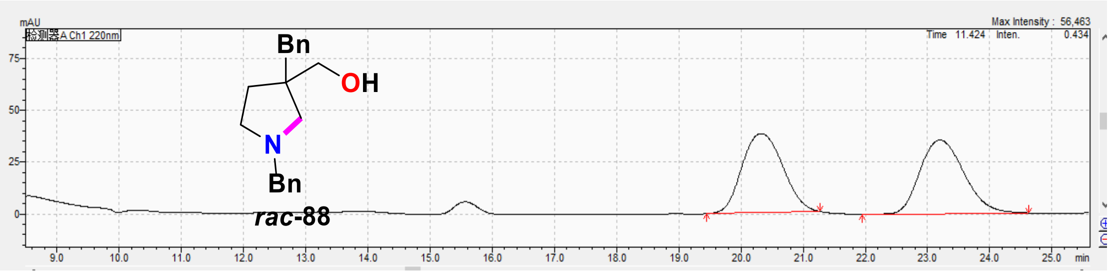

Peak #	Ret. Time [min]	Area mAu*s	Hight mAu	Width [min]	Area %
1	20.322	1740079	38166	0.734	49.928
2	23.199	1745082	35512	0.776	50.072

Supplementary Figure 299. HPLC profile of ***rac*-88** standard**.**

(*S*)-(1,3-Dibenzylpyrrolidin-3-yl)methanol (88)

SZ611: HPLC (ChiralPak AD-H, hexane: isopropanol = 98:2, 1 mL/min, 220nm)

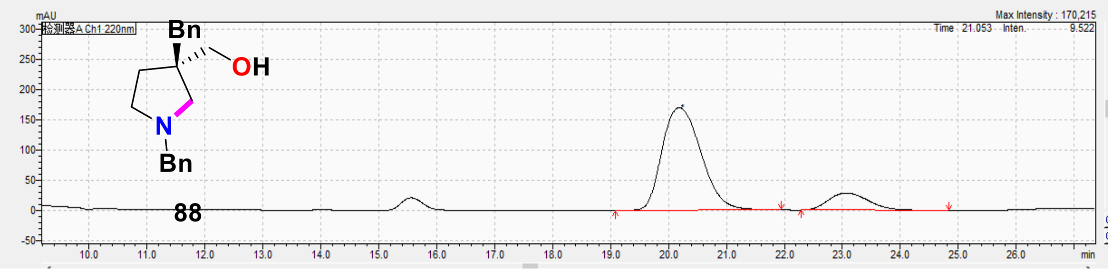

Peak #	Ret. Time [min]	Area mAu*s	Hight mAu	Width [min]	Area %
1	20.183	7957893	170081	0.742	85.339
2	23.071	1367163	28256	0.765	14.661

Supplementary Figure 300. HPLC profile of the crude product **(*R*)-88** catalyzed by mutant SZ611**.**

(*rac*)-(1-Benzyl-3-phenylpiperidin-3-yl)methanol

HPLC (ChiralPak AD-H, hexane: isopropanol = 98:2, 1 mL/min, 220nm)

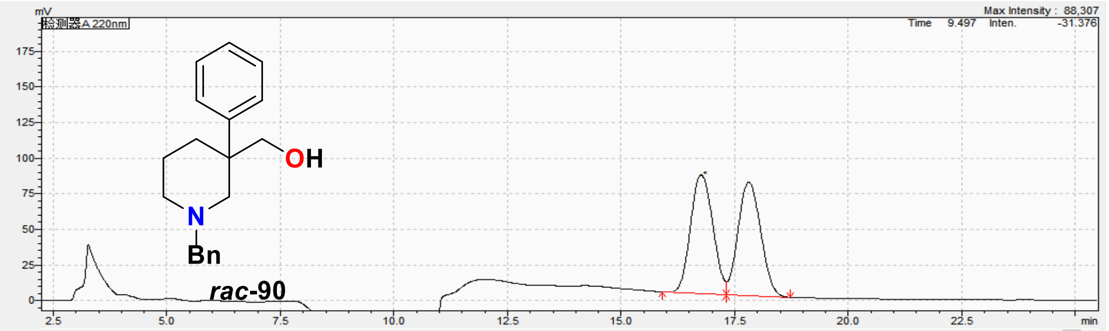

Peak #	Ret. Time [min]	Area mAu*s	Hight mAu	Width [min]	Area %
1	16.760	2858993	83498	0.549	50.684
2	17.809	2781853	79650	0.557	49.316

Supplementary Figure 301. HPLC profile of ***rac*-90** standard**.**

(*R*)-(1-Benzyl-3-phenylpiperidin-3-yl)methanol (90)

SZ611: HPLC (ChiralPak AD-H, hexane: isopropanol = 98:2, 1 mL/min, 220nm)

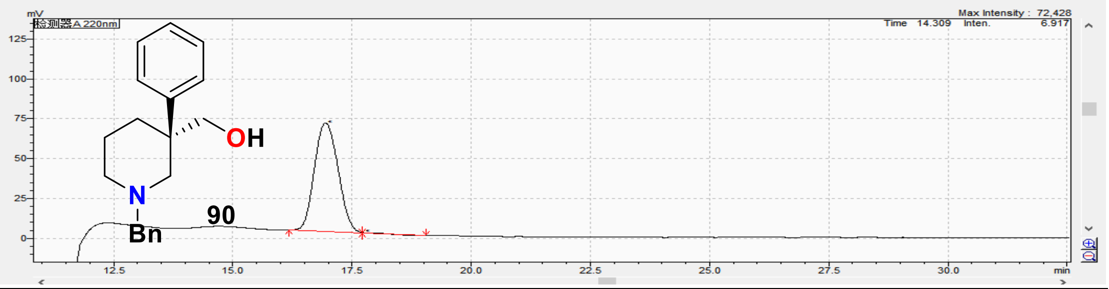

Peak #	Ret. Time [min]	Area mAu*s	Hight mAu	Width [min]	Area %
1	16.942	2343460	68092	0.553	99.812
2	17.729	4411	259	-	0.188

Supplementary Figure 302. HPLC profile of the crude product **(*R*)-90** catalyzed by mutant SZ611**.**

(*rac*)-(1,3-Dibenzylpiperidin-3-yl)methanol

HPLC (ChiralPak AD-H, hexane: isopropanol =90：10, 1 mL/min, 220nm)

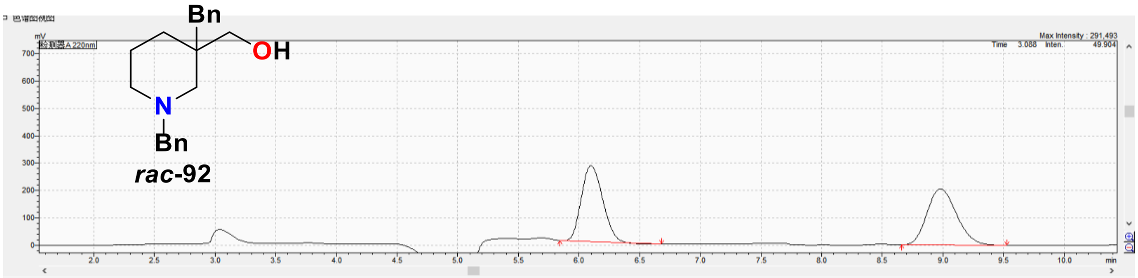

Peak #	Ret. Time [min]	Area mAu*s	Hight mAu	Width [min]	Area %
1	6.099	3385732	277628	0.194	49.531
2	8.979	3449832	204774	0.264	50.469

Supplementary Figure 303. HPLC profile of ***rac*-92** standard**.**

(*R*)-(1,3-Dibenzylpiperidin-3-yl)methanol

SZ611: HPLC (ChiralPak AD-H, hexane: isopropanol =90：10, 1 mL/min, 220nm)

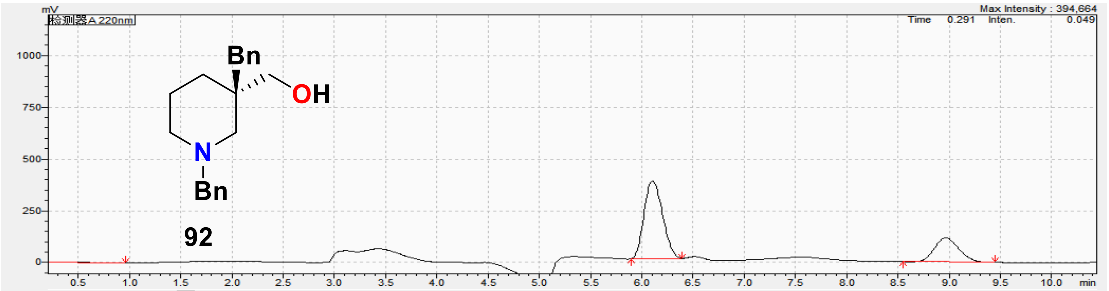

Peak #	Ret. Time [min]	Area mAu*s	Hight mAu	Width [min]	Area %
1	6.104	4721854	376277	0.201	70.940
2	8.966	1934229	116162	0.262	29.060

Supplementary Figure 304. HPLC profile of the crude product **(*R*)-92** catalyzed by mutant SZ611**.**

Ethyl (*E*)-3-(1-benzyl-3-phenylpyrrolidin-3-yl)acrylate (93)

HPLC (ChiralPak OJ-H, hexane: isopropanol = 97:3, 1 mL/min, 220nm)

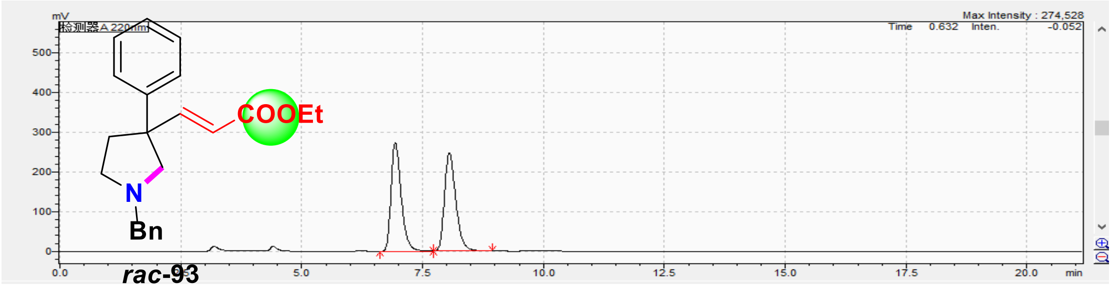

Peak #	Ret. Time [min]	Area mAu*s	Hight mAu	Width [min]	Area %
1	6.936	4055554	274874	0.221	50.223
2	8.053	4019506	247521	0.246	49.777

Supplementary Figure 305. HPLC profile of ***rac*-93** standard.

Ethyl (*R*, *E*)-3-(1-benzyl-3-phenylpyrrolidin-3-yl)acrylate (93)

HPLC (ChiralPak OJ-H, hexane: isopropanol = 97:3, 1 mL/min, 220nm)

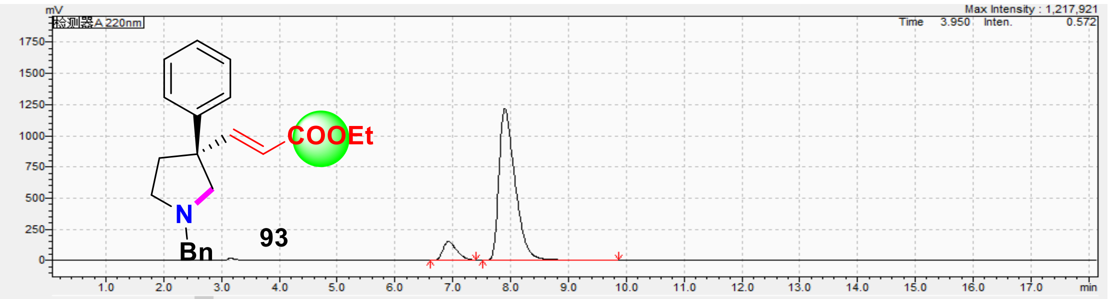

Peak #	Ret. Time [min]	Area mAu*s	Hight mAu	Width [min]	Area %
1	6.926	2507366	150684	0.257	9.602
2	7.900	23605709	1216291	0.295	90.398

Supplementary Figure 306. HPLC profile of the crude product **(*R*)-93.**

(*rac*)-(1-Benzyl-3-phenylpyrrolidin-3-yl)methyl 4-methylbenzenesulfonate（94）

HPLC (ChiralPak AD-H, hexane: isopropanol = 98:2, 1 mL/min, 220nm)

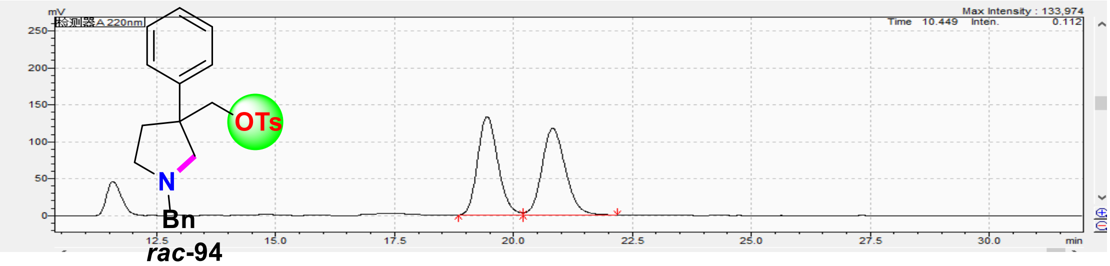

Peak #	Ret. Time [min]	Area mAu*s	Hight mAu	Width [min]	Area %
1	19.443	3964049	133040	0.458	49.507
2	20.824	4042961	117412	0.527	50.493

Supplementary Figure 307. HPLC profile of ***rac*-94** standard.

(*R*)-(1-Benzyl-3-phenylpyrrolidin-3-yl)methyl 4-methylbenzenesulfonate (94)

HPLC (ChiralPak AD-H, hexane: isopropanol = 98:2, 1 mL/min, 220nm)

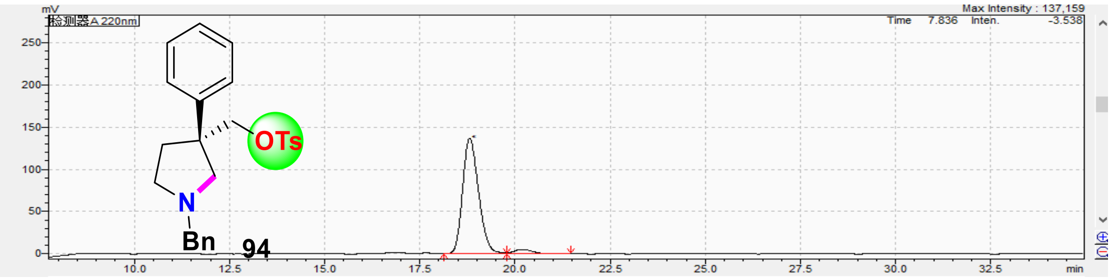

Peak #	Ret. Time [min]	Area mAu*s	Hight mAu	Width [min]	Area %
1	18.811	3975937	136832	0.449	96.228
2	20.193	155839	4701	0.520	3.772

Supplementary Figure 308. HPLC profile of the crude product **(*R*)-94.**

(*rac*)-3-(Azidomethyl)-1-benzyl-3-phenylpyrrolidine (95)

HPLC (ChiralPak AS-H, hexane: isopropanol = 99:1, 1 mL/min, 220nm)

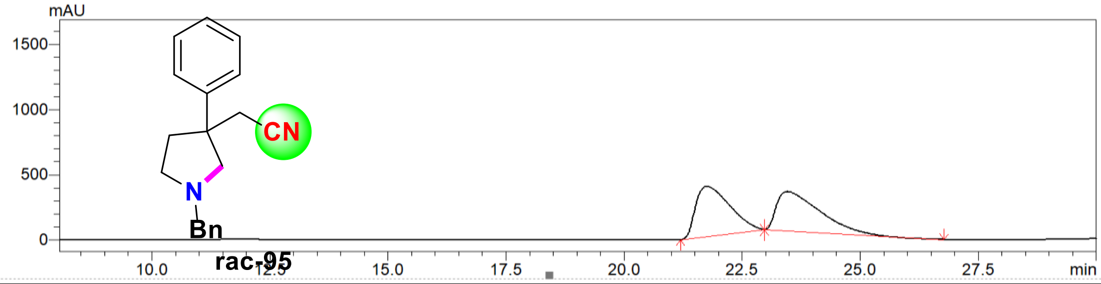

Peak #	Ret. Time [min]	Area mAu*s	Hight mAu	Width [min]	Area %
1	21.763	19358490	50.330	383587	50.330
2	23.470	19104335	49.670	300905	49.670

Supplementary Figure 309. HPLC profile of ***rac*-95** standard.

(*R*)-3-(Azidomethyl)-1-benzyl-3-phenylpyrrolidine (95)

HPLC (ChiralPak AS-H, hexane: isopropanol = 99:1, 1 mL/min, 220nm)

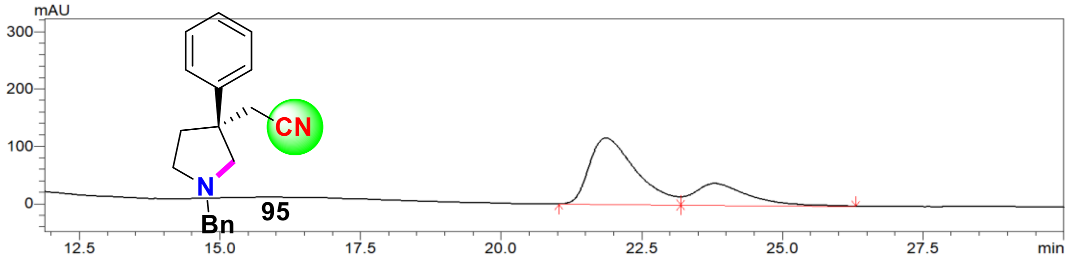

Peak #	Ret. Time [min]	Area mAu*s	Hight mAu	Width [min]	Area %
1	21.867	6730370	73.009	116226	73.009
2	23.792	2488214	26.991	37821	26.991

Supplementary Figure 310. HPLC profile of the crude product **(*R*)-95.**

(*rac*)-2-(1-Benzyl-3-phenylpyrrolidin-3-yl)acetonitrile (96)

HPLC (ChiralPak OJ-H, hexane: isopropanol =98:2, 1 mL/min, 220nm)

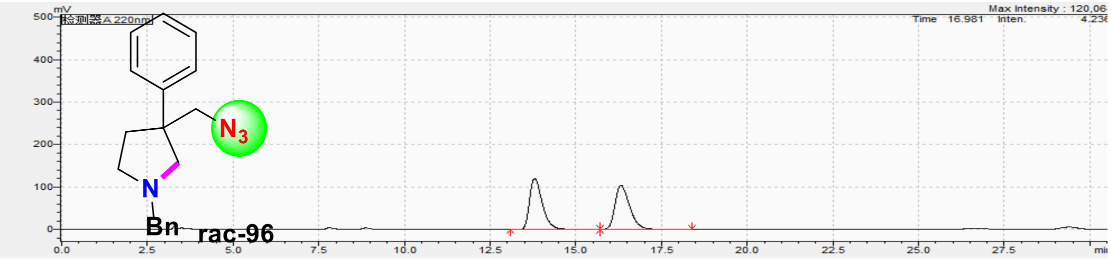

Peak #	Ret. Time [min]	Area mAu*s	Hight mAu	Width [min]	Area %
1	13.798	3132079	120378	0.390	50.184
2	16.316	3109135	104494	0.452	49.816

Supplementary Figure 311. HPLC profile of ***rac*-96** standard.

(*R*)-2-(1-Benzyl-3-phenylpyrrolidin-3-yl)acetonitrile (96)

HPLC (ChiralPak AD-H, hexane: isopropanol =98:2, 1 mL/min, 220nm)

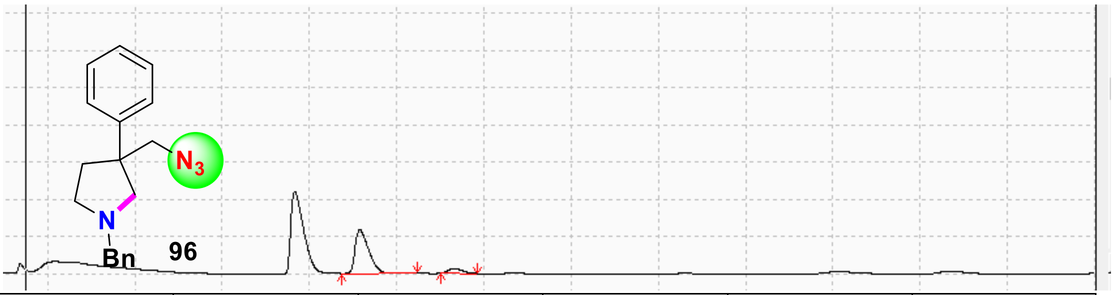

Peak #	Ret. Time [min]	Area mAu*s	Hight mAu	Width [min]	Area %
1	13.941	7924453	293416	0.419	89.731
2	16.643	906874	32542	0.443	10.269

Supplementary Figure 312. HPLC profile of the crude product **(*R*)-96.**

(*rac*)-Benzyl-3-(hydroxymethyl)-3-phenylpyrrolidine-1-carboxylate (97)

HPLC (ChiralPak AD-H, hexane: isopropanol =98:2, 1 mL/min, 220nm)

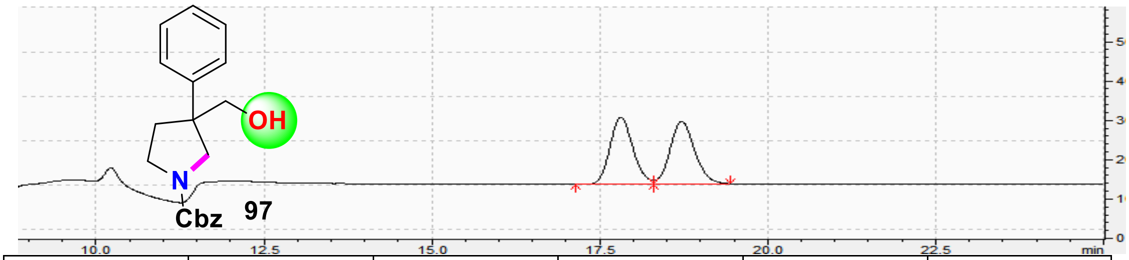

Peak #	Ret. Time [min]	Area mAu*s	Hight mAu	Width [min]	Area %
1	17.813	896710	37740	0.367	50.015
2	18.719	896170	35175	0.391	49.985

Supplementary Figure 313. HPLC profile of ***rac*-97** standard.

Benzyl (*R*)-3-(hydroxymethyl)-3-phenylpyrrolidine-1-carboxylate (97)

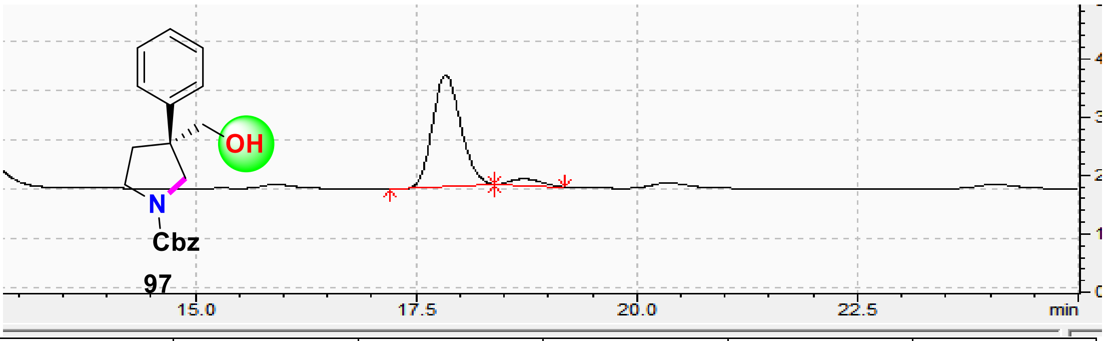

Peak #	Ret. Time [min]	Area mAu*s	Hight mAu	Width [min]	Area %
1	17.836	1257405	56379	0.349	94.123
2	18.719	78509	3276	0.395	5.877

Supplementary Figure 314. HPLC profile of the crude product **(*R*)-97.**

(*rac*)-Benzyl -3-formyl-3-phenylpyrrolidine-1-carboxylate (98)

HPLC (ChiralPak OD-H, hexane: isopropanol =90:10, 1 mL/min, 220nm)

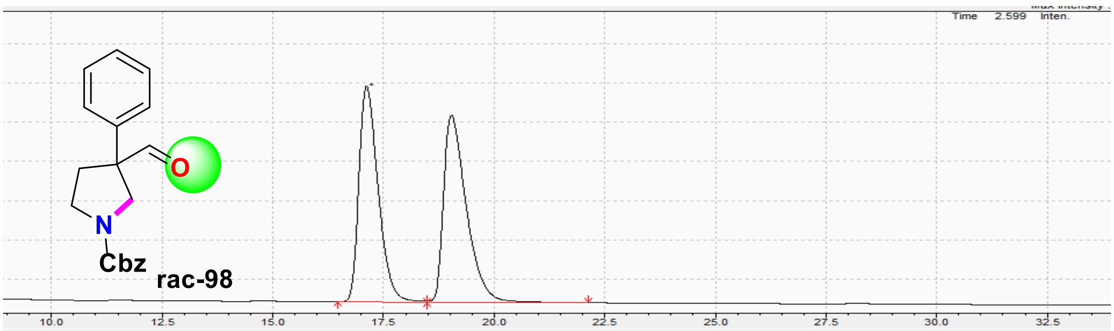

Peak #	Ret. Time [min]	Area mAu*s	Hight mAu	Width [min]	Area %
1	17.118	16534541	548719	0.461	49.274
2	19.038	17021668	474892	0.539	50.726

Supplementary Figure 315. HPLC profile of ***rac*-98** standard**.**

Benzyl (*R*)-3-formyl-3-phenylpyrrolidine-1-carboxylate (98)

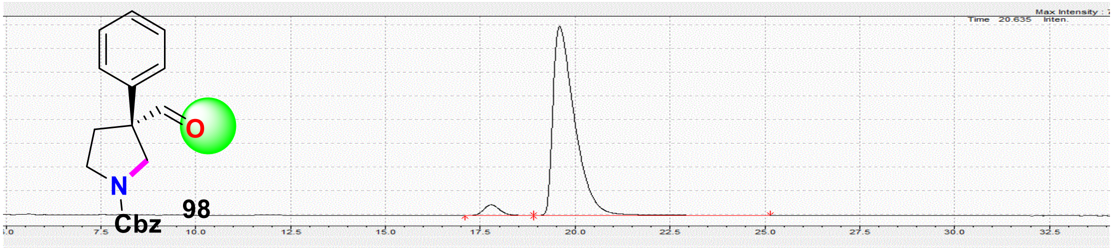

Peak #	Ret. Time [min]	Area mAu*s	Hight mAu	Width [min]	Area %
1	17.783	1333431	45039	0.453	4.025
2	19.593	31793289	796685	0.596	95.975

Supplementary Figure 316. HPLC profile of the crude product **(*R*)-98.**

4. Supplementary References

1. Wu, M. H., Hansen, K. B. & Jacobsen, E. N. Regio- and enantioselective cyclization of epoxy alcohols catalyzed by a [Co^{III}(salen)] complex. *Angew. Chem. Int. Ed.* **38**, 2012-2014 (1999).

2. Zhang, X. et al. Primary anion−π catalysis and autocatalysis. *J. Am. Chem. Soc.* **140**, 17867-17871 (2018).

3. Paraja, M. & Matile, S. Primary anion–π catalysis of epoxide-opening ether cyclization into rings of different sizes: access to new reactivity. *Angew. Chem. Int. Ed*. **59**, 6273-6277 (2020).

4. Zhang, R., Guo, W., Duan, M., Houk, K. N. & Sun, J. Asymmetric desymmetrization of oxetanes for the synthesis of chiral tetrahydrothiophenes and tetrahydroselenophenes. *Angew. Chem. Int. Ed.* **58**, 18055-18060 (2019).

5. Loy, R. N. & Jacobsen, E. N. Enantioselective intramolecular openings of oxetanes catalyzed by (salen)Co (III) complexes: access to enantioenriched tetrahydrofurans. *J. Am. Chem. Soc.***131**, 2786-2787 (2009).

6. Kuriyama, Y. et al. Highly regioselective 5-endo-tet cyclization of 3,4-epoxy amines into 3-hydroxypyrrolidines catalyzed by $La(OTf)_3$. *Chem. Eur. J.* **27**, 1961-1965 (2021).

7. Chang M-Y, Pai C-L, Kung Y-H. Synthesis of (±)-coerulescine and a formal synthesis of (±)-horsfiline . *Tetrahedron Letters*, **46**, 8463-8465 (2005).

8. Yamamoto Y, Shimizu E, Ban K, et al. Facile Hydrogenative Deprotection of *N*-Benzyl Groups Using a Mixed Catalyst of Palladium and Niobic Acid-on-Carbon. *ACS Omega*, **5**, 2699-2709 (2020).
