# Supplementary material for: Rational enzyme design for enabling biocatalytic Baldwin cyclization and asymmetric synthesis of chiral heterocycles
Source: Nat Commun. 2022 Dec 19;13:7813. doi: 10.1038/s41467-022-35468-y (PMC9763437; doi:10.1038/s41467-022-35468-y)
Supplement: Supplementary file 3 — Description of Additional Supplementary Files [file 41467_2022_35468_MOESM3_ESM.pdf]

Description of Additional Supplementary Files

Supplementary Movie 1. The bond forming and breaking in the SZ612- catalyzed transformation of substrate (*R*)-1 to Baldwin product (*S*)-**2**.

Supplementary Movie 2. The bond forming and breaking in the SZ612- catalyzed transformation of substrate (*R*)-1 to anti-Baldwin product (*R*)-**3**.

Supplementary Movie 3. The bond forming and breaking in the SZ616- catalyzed transformation of substrate (*R*)-1 to Baldwin product (*S*)-**2**.

Supplementary Movie 4. The bond forming and breaking in the SZ616- catalyzed transformation of substrate (*R*)-1 to anti-Baldwin product (*R*)-**3**.
