# Supplementary material for: Rational enzyme design for enabling biocatalytic Baldwin cyclization and asymmetric synthesis of chiral heterocycles
Source: Nat Commun. 2022 Dec 19;13:7813. doi: 10.1038/s41467-022-35468-y (PMC9763437; doi:10.1038/s41467-022-35468-y)
Supplement: Supplementary file 8 — Reporting Summary [file 41467_2022_35468_MOESM8_ESM.pdf]

nature portfolio

Corresponding author(s): Jun-An Ma, Manfred T. Reetz and Zhoutong Sun

Last updated by author(s): Nov 12, 2022

Reporting Summary

Nature Portfolio wishes to improve the reproducibility of the work that we publish. This form provides structure for consistency and transparency in reporting. For further information on Nature Portfolio policies, see our Editorial Policies and the Editorial Policy Checklist.

Statistics

For all statistical analyses, confirm that the following items are present in the figure legend, table legend, main text, or Methods section.

n/a	Confirmed	
☒	☐	The exact sample size (*n*) for each experimental group/condition, given as a discrete number and unit of measurement
☒	☐	A statement on whether measurements were taken from distinct samples or whether the same sample was measured repeatedly
☒	☐	The statistical test(s) used AND whether they are one- or two-sided<br>*Only common tests should be described solely by name; describe more complex techniques in the Methods section.*
☒	☐	A description of all covariates tested
☒	☐	A description of any assumptions or corrections, such as tests of normality and adjustment for multiple comparisons
☒	☐	A full description of the statistical parameters including central tendency (e.g. means) or other basic estimates (e.g. regression coefficient) AND variation (e.g. standard deviation) or associated estimates of uncertainty (e.g. confidence intervals)
☒	☐	For null hypothesis testing, the test statistic (e.g. *F*, *t*, *r*) with confidence intervals, effect sizes, degrees of freedom and *P* value noted<br>*Give P values as exact values whenever suitable.*
☒	☐	For Bayesian analysis, information on the choice of priors and Markov chain Monte Carlo settings
☒	☐	For hierarchical and complex designs, identification of the appropriate level for tests and full reporting of outcomes
☒	☐	Estimates of effect sizes (e.g. Cohen's *d*, Pearson's *r*), indicating how they were calculated

Our web collection on statistics for biologists contains articles on many of the points above.

Software and code

Policy information about availability of computer code

Data collection	No novel software or code was established in this work. Schrödinger Maestro software (release 2016-2) was used to prepare protein and ligand structures. Molecular dynamics (MD) simulations and QM/MM calculations were performed by using the commercial software Amber (GPU version 2016) and open-source GTKDynamo tool (1.9.0). X-ray data were collected by using BL17B1 beamline of Shanghai Synchrotron Radiation Facility
Data analysis	Crystallographic data sets were processed by using the HKL-2000 package, the refinement was analyzed with PHENIX (version 1.11.1_2575), and the models were rebuilt manually with Coot (0.9.8.1). The MD and QM/MM data were analyzed using Amber (GPU version 2016, embedded with SHAKE algorithm) and GTKDynamo tool (1.9.0).

For manuscripts utilizing custom algorithms or software that are central to the research but not yet described in published literature, software must be made available to editors and reviewers. We strongly encourage code deposition in a community repository (e.g. GitHub). See the Nature Portfolio guidelines for submitting code & software for further information.

Data

Policy information about availability of data

All manuscripts must include a data availability statement. This statement should provide the following information, where applicable:

- Accession codes, unique identifiers, or web links for publicly available datasets
- A description of any restrictions on data availability
- For clinical datasets or third party data, please ensure that the statement adheres to our policy

The authors declare that all the data supporting the findings of this study are available within the main text, and Supplementary Information documents. The X-ray structural data have been deposited in the Protein Data Bank (www.rcsb.org) with accession codes 7VWD, 7VX2, 7VWM, 7XEE and 7XEF respectively. Crystallographic data for the structures reported in this Article have been deposited at the Cambridge Crystallographic Data Centre, under deposition numbers: 2162958 (Derivative of the product 54). This data can be obtained free of charge from The Cambridge Crystallographic Data Center via www.ccdc.cam.ac.uk/data_request/cif. Additional data supporting the findings of this study are available from the corresponding author on request.

Human research participants

Policy information about studies involving human research participants and Sex and Gender in Research.

Reporting on sex and gender	n/a
Population characteristics	n/a
Recruitment	n/a
Ethics oversight	n/a

Note that full information on the approval of the study protocol must also be provided in the manuscript.

Field-specific reporting

Please select the one below that is the best fit for your research. If you are not sure, read the appropriate sections before making your selection.

[x] Life sciences   [ ] Behavioural & social sciences   [ ] Ecological, evolutionary & environmental sciences

For a reference copy of the document with all sections, see nature.com/documents/nr-reporting-summary-flat.pdf

Life sciences study design

All studies must disclose on these points even when the disclosure is negative.

Sample size	No sample size calculations were performed for this study. Sample sizes are indicated for each experiment and were chosen based on similar studies. Experiments were performed in triplicates (n=3).
Data exclusions	No mutational data was excluded.
Replication	The positive hits obtained from screening were evaluated in triplicate and showed similar results. All attempts at replication are successful.
Randomization	n/a, no claims on randomization, this work does not involve animal or human subjects or group allocation.
Blinding	n/a, no claims on blinding, this work does not involve animal or human subjects or group allocation.

Reporting for specific materials, systems and methods

We require information from authors about some types of materials, experimental systems and methods used in many studies. Here, indicate whether each material, system or method listed is relevant to your study. If you are not sure if a list item applies to your research, read the appropriate section before selecting a response.

Materials & experimental systems

n/a	Involved in the study	
☒	☐	Antibodies
☒	☐	Eukaryotic cell lines
☒	☐	Palaeontology and archaeology
☒	☐	Animals and other organisms
☒	☐	Clinical data
☒	☐	Dual use research of concern

Methods

n/a	Involved in the study	
☒	☐	ChIP-seq
☒	☐	Flow cytometry
☒	☐	MRI-based neuroimaging
